# New predicted dual CDK-2/CDK-1 inhibitors from *Aspergillus unguis* isolate SP51-EGY with relative selectivity for colorectal cancer cells: a computational and experimental approach

**DOI:** 10.1038/s41598-026-41120-2

**Published:** 2026-04-11

**Authors:** Ahmed A. El-Rashedy, Amal Mosad Ibrahim, Mohamed S. Abdel-Aziz, Walid Fayad, Faten K. Abd EL-Hady

**Affiliations:** 1 Chemistry of Natural and Microbial Products Department, Pharmaceutical and Drug Industries Research Institute, National Research Centre, 33 El Buhouth St., Dokki-Giza, 12622 Egypt; 2 Department of Organic and Medicinal Chemistry, Faculty of Pharmacy, University of Sadat City, Monofia, 32897 Egypt; 3 Department of Microbial Chemistry, National Research Centre, Giza, 12622 Egypt; 4 Drug Bioassay-Cell Culture Laboratory, Pharmacognosy Department, National Research Centre, Giza, 12622 Egypt

**Keywords:** *Aspergillus unguis* isolate SP51-EGY, HCT116 cancer, In silico study, CDK-2 and CDK-1 inhibitors, Cancer, Computational biology and bioinformatics, Drug discovery

## Abstract

Colorectal cancer is one of the deadliest cancers in the world. The main problem with cancer treatments is that they need to protect healthy cells. Cyclin-dependent kinases (CDKs), especially CDK-2 and CDK-1, are essential for regulating the cell cycle, cell growth, and tumor genesis. Their dysregulation is frequently detected in colorectal cancer, resulting in uncontrolled cell division and resistance to apoptosis, making them attractive targets for anticancer therapy. The fungal "*Aspergillus unguis* isolate SP51-EGY" “Sh cell” (shake mycelia) extract had the most significant cytotoxic action against HCT116 cancer cells (IC_50_ = 3.49 µg/mL) and a selectivity index (SI = 23.33), indicating relative cytotoxicity against cancer cells. Computational modeling and cellular phenotypic data suggest a potential mechanism of dual CDK2/1 inhibition, forming a testable hypothesis that requires direct enzymatic validation. Compounds corresponding to peaks [7] and [14] have been tentatively assigned as promising CDK inhibitors based on molecular docking studies; due to their: (1) strong binding affinities (-13.23 to -46.05 kcal/mol) confirmed by stable molecular dynamics (RMSD < 2.0 Å); (2) cell cycle arrest mediated by dual CDK2/1 inhibition; and (3) favorable drug-like properties, such as molecular weight < 500 Da, good intestinal absorption, and minimal toxicity. Critical interactions with CDK2 (Tyr16, Phe81) and CDK1 (Met88) active site residues were identified by structural analysis. These interactions competed with ATP to disrupt the CDK2 cyclin A and CDK1/cyclin A/B complexes, which resulted in both G1/S and G2/M cell cycle arrests. These integrated computational and cellular findings suggest that the fungal-derived compounds are promising candidates for novel dual CDK2/CDK1 inhibitors, warranting further experimental validation through biochemical kinase assays.

**Supplementary Information:**

The online version contains supplementary material available at 10.1038/s41598-026-41120-2.

## Introduction

Cancer remains a leading cause of global mortality, with an estimated 20 million new cases and 9.7 million deaths reported worldwide in 2022, underscoring the persistent demand for effective and safe therapies^1^. Current chemotherapeutic regimens are frequently hindered by significant challenges, including multidrug resistance, poor solubility, and detrimental side effects on healthy cells^2^ . This is particularly critical for colorectal cancer (CRC), which is projected to be the third most diagnosed cancer globally, with an anticipated 1.93 million new cases annually by 2025^3^. Despite treatment advances, the 5-year survival rate for CRC patients remains around 60%^4^, and the incidence is expected to rise from 1.15 million in 2020 to 1.92 million by 2040^5^. A central driver of this malignancy—and a key reason for treatment failure—is the dysregulation of core cell cycle machinery, leading to uncontrolled proliferation and resistance to apoptosis. This makes critical regulators of the cell cycle, particularly cyclin-dependent kinases (CDKs), prime therapeutic targets. Among CDKs, CDK-2 and CDK-1 are especially pivotal: CDK-2 governs the G1/S phase transition, while CDK-1 is essential for the G2/M transition. Their frequent dysregulation in CRC contributes directly to the unchecked cell division that characterizes the disease. Therefore, the dual inhibition of CDK2 and CDK1 represents a promising strategy to halt CRC progression by simultaneously inducing cell cycle arrest at multiple checkpoints.

Somatic mutations in the adenomatosis polyposis coli (APC) gene, a crucial tumor suppressor, enhance an individual’s susceptibility to genetic vulnerabilities. Chronic inflammation, which leads to oxidative cellular damage, is thought to play a significant role in the development of colorectal cancer^6^. Moreover, two major challenges in cancer treatment are drug resistance in cancer cells and toxicity to healthy cells. Currently, chemotherapeutic agents provide cancer patients with short-term relief and improved life expectancy. However, the high costs, lack of selectivity, and adverse side effects of these medications adversely affect patients’ quality of life. As a result, millions of individuals in low-income countries are unable to afford these essential treatments.

Although direct cost-efficiency comparisons with established chemotherapeutics such as doxorubicin are premature, marine fungal extracts may offer long-term economic advantages owing to renewable production, scalable fermentation, and potential reductions in treatment-associated toxicity.As stated by Blagosklonny^7^, the main obstacle is ensuring that normal cell protection is selective.

There is an ever-increasing need for novel, compelling, and selective compounds that can alleviate and assist many aspects of human disease. Therefore, finding new medicines in nature has been the primary goal of research.

Colorectal cancer (CRC) ranks among the most common and fatal cancers globally, necessitating the urgent discovery of new treatment approaches. CDK-2 and CDK-1 are cyclin-dependent kinases (CDKs) that are extremely important for cell cycle control, proliferation, and tumor development. When kinases are not adequately regulated, it leads to uncontrolled cell division and resistance to cell death, which are often seen in colorectal cancer^8^.

Attractive targets for anticancer therapy include CDK-2, which is crucial for the G1/S transition, and CDK-1, which governs the G2/M phase. One potential strategy for treating colorectal cancer is to inhibit these kinases, which can cause cancer cells to undergo cell cycle arrest and even death. Investigations into small-molecule CDK inhibitors abound, but increasing their efficacy and selectivity has been a formidable challenge^8^. Key players in the cell cycle transitions are the CDK2-Cyclin A, CDK2-Cyclin E, CDK1-Cyclin A, and CDK1-Cyclin B complexes. When the CDK-cyclin complexes are not working correctly, colon cancer cells can resist cell death, have unstable DNA, and grow uncontrollably.

A possible way to treat colorectal cancer is to use small-molecule inhibitors (like CDK1/2 inhibitors) or combination therapies to target the CDK-cyclin complexes; these complexes play crucial roles in the course of the disease^9^.

One efficient and cost-effective method to find possible inhibitors of CDK-2 and CDK-1 is to conduct in silico investigations, which include molecular docking, dynamics simulations, and virtual screening^10^.

There is an ever-increasing need for novel, compelling, and selective compounds that can alleviate and assist many aspects of human disease. Therefore, exploring biologically active natural products from underexplored sources, such as marine fungi, remains a primary goal in anticancer drug discovery. Recent analyses highlight the continued potential of bioactive natural-product scaffolds in developing new therapeutic agents against challenging targets like cyclin-dependent kinases^11^ In this context, the fungal *'Aspergillus unguis* isolate SP51-EGY’ represents a promising, underexplored source for such discovery.

It is widely recognized that microorganisms from the Red Sea can produce a diverse array of physiologically active metabolites with distinctive structures, representing a promising source for drug discovery^12,13^. While the specific metabolites from *Aspergillus unguis* isolate SP51-EGY have not been previously characterized for CDK inhibition, marine fungi in general are known to yield compounds with significant anti-tumor and other pharmacological activities^14^. This broad precedent supports our investigation of this underexplored fungal isolate as a potential source of novel CDK inhibitors.

Rapid breakthroughs in anticancer therapy have accelerated the creation of numerous effective therapeutic compounds that target the most essential mechanisms driving neoplastic progression. The overexpression of cyclin-dependent kinases (CDKs) is one of the factors that has garnered significant attention from researchers.

In this study, we adopt a hybrid in silico/in vitro strategy to discover new CDK1/2 inhibitors.

The development of CDK inhibitors derived from fungi offers hope for the treatment of colon cancer, especially in cases where cell cycle dysregulation is the focus. New supplementary or alternative therapies may emerge from future investigations into the mechanisms of action of particular bioactive substances.

Our goal is to discover novel, relatively selective marine fungal extracts that alleviate colorectal cancer (CRC). This represents a significant and urgent challenge to finding a safe and effective drug for clinical trials.

This study aims to discover anticancer drugs derived from fungal extracts that selectively exhibit cytotoxic effects.

Researchers have studied several small-molecule CDK inhibitors, but ensuring selective and effective normal cell protection remains a challenge.

## Results and discussion

### Effect of fungal extracts on cell cytotoxicity

**Fig. 1:**
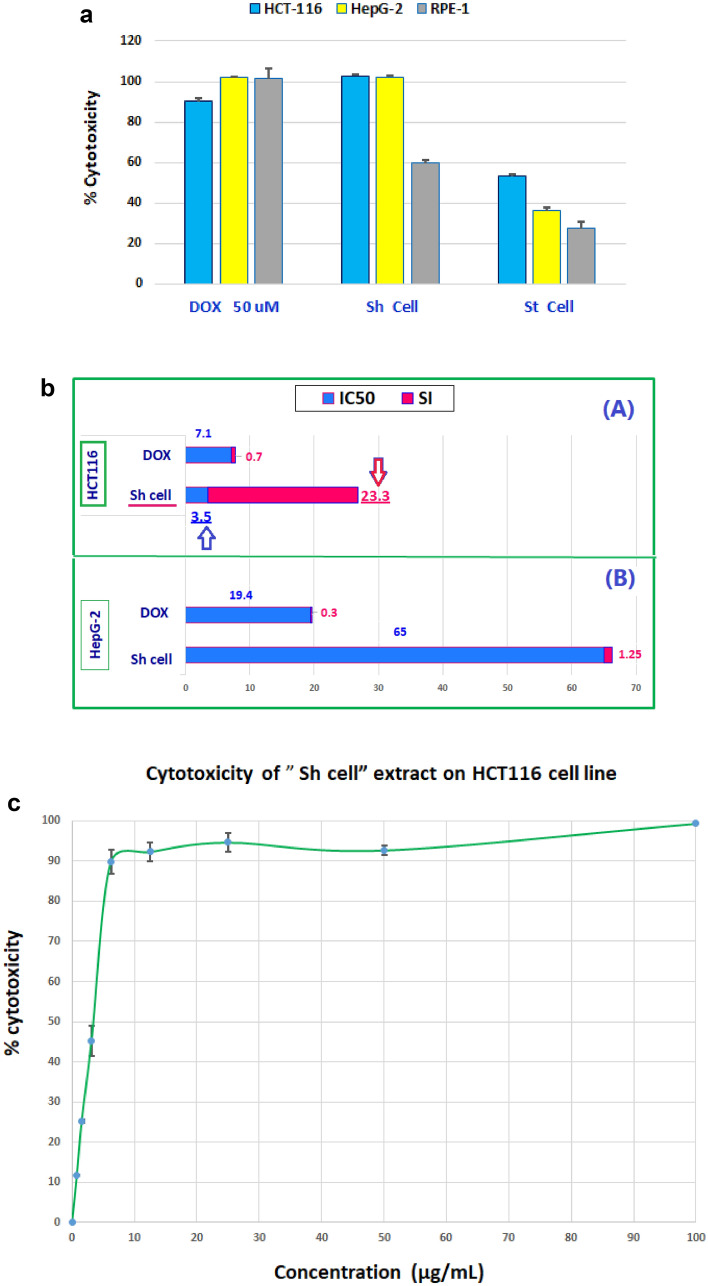
a Average % cytotoxicity of the fungus “*Aspergillus unguis* isolate SP51-EGY”, different extracts, and doxorubicin (DOX) on cell viability/proliferation against HCT-116, HepG2, and RPE1 cell lines after 48 h, evaluated by MTT assay. Values are expressed as mean ± SD, n = 3 at a concentration of 100 µg/mL. Mainly, DOX expressed the same effect against cancer and normal “RPE1 “cells; meanwhile, the fungal extracts showed low cytotoxic activity against the normal cells. b. It shows the cytotoxic activity against the HCT-116 cancer cell line (**A**) of the most significant and highly selective " Sh cell" extract [IC_50_ = 3.5 µg/mL & SI = 23.3]. The HepG-2 cancer cell line was not cytotoxically affected by the “Sh cell” extract [IC_50_ = 65.0 µg/ml & SI = 1.25] (**B**). DOX demonstrated very low SI [0.7, 0.3] and low IC_50_ [7.1, 19.4 µg/mL] against HCT-116 and HepG-2 cancer cell lines. DOX was harmful to normal cells but had a low IC_50_ on the tested cancer cell lines. (**C**). The dose-response curve for the most effective” Sh cell” extract showed that the suppression of HCT116 cell growth was dose-dependent

The fungus "*Aspergillus unguis* isolate SP51-EGY" was cultured in a broth medium, producing Sh cell (shake mycelia) and St cell (static mycelia) extracts; conditions were evaluated for their in vitro cytotoxic effects against HCT116, HepG2, and RPE1 cell lines after 48 h at a concentration of 100 µg/mL using the MTT assay (Table 1, Fig. 1a).

**Table 1:** Inhibitory concentration 50 (IC_50_) and selectivity index (SI) of the active cytotoxic extracts of the fungus “*Aspergillus unguis* isolate SP51-EGY” against HCT-116, HepG2, and RPE-1 cell lines.

Extracts	HCT-116 cell line			HepG2 cell line			RPE-1 cell lines	
	% cytotoxicity at 100 µg/mL	IC_50_ µg/mL	SI	% cytotoxicity at 100 µg/mL	IC_50_ µg/mL	SI	% cytotoxicity at 100 µg/mL	IC_50_ µg/mL
Sh cell	99.4 ± 0.29	**3.49 ± 0.60**	**23.34**	99.4 ± 0.6	**65 ± 0.55**	**1.25**	59.6 ± 1.7	81.48 ± 1.7
St cell	53.1 ± 1.1	63.5 ± 1.13	>1.57	36.1 ± 1.5	**–**	**–**	27.37 ± 3.4	>100
DOX (50 Um)	90.2 ± 1.3	7.1 ± 0.23	0.70	99.4 ± 0.46	19.4 ± 1.9	0.3	98.4 ± 1.4	5.3 ± 0.14

Doxorubicin (DOX), the standard treatment agent, acted as the positive control, while 0.5% DMSO served as the negative control. The “Sh cell” fungal extract exhibited the most significant cytotoxic effect against HCT-116, while showing relatively lower cytotoxicity towards normal RPE1 cells compared to cancer cells.

The cytotoxic effects were quantified as IC_50_ and selectivity index (SI) for the most potent fungal extract against HCT-116 and HepG-2 cancer cell lines (Table 1, Fig. 1b). According to prior research, an SI value exceeding two is deemed safe^15^.

The “Sh cell” extract had the most significant cytotoxic activity against HCT116 cancer cells, exhibiting a high safety profile for normal cells [IC_50_ = 3.49 µg/mL & SI = 23.33], while showing no cytotoxicity against HepG-2 cells [IC_50_ = 65.0 µg/mL & SI = 1.25] (Table 1, Fig. 1b).

The dose–response curve for Sh cell extract demonstrated that the inhibition of HCT-116 cell proliferation was dose-dependent (Fig. 1c).

DOX exhibited a low IC_50_ in the assessed cancer cell lines; however, it also showed toxicity to normal cells. This indicates that existing chemotherapeutic agents may not be inherently safe for normal cells, and that novel, safer pharmaceuticals can be discovered.

### GC/MS analysis

The composition of the highly cytotoxic extract known as "Sh cell," with an IC_50_ of 3.49 µg/mL and a selectivity index (SI) of 23.33, was analyzed via GC/MS (Table 2). The GC/MS analysis revealed 17 chemicals, none of which exhibited previously documented anticancer properties. The compounds exhibiting the highest area percentage are 2,4-diphenyl-glutaronitrile (1.33%); 7,9-diethyl-2,4-bis(dimethylamino)-10-imino-8-thio-1,7,9-triazaspiro[4.5]-1,3-decadiene-6,8-dione (4.25%); hexane-2,5-dihydroxy (6.99%); 2,13-dithia(3)meta-cyclo-(3)naphthalenophane (7.74%); 2,6-dibromophenyl 3-phenylpropa -noate (0.61%); butanoic acid heptafluoromethyl ester (0.4%) and 10,13-octadecadiynoic acid methyl ester (0.2%). The GC/MS analysis yielded a qualitative profile and relative abundance (Area %) for the components present in the intricate ‘Sh cell’ extract. Nevertheless, the precise identification of individual compounds is not achievable without prior isolation and purification. Consequently, the structural assignments, particularly for the complex heterocycle [7], are tentative and require definitive confirmation through isolation followed by comprehensive spectroscopic analysis (e.g., NMR, HRMS). Moreover, the extract’s cytotoxic activity (IC_50_ = 3.49 µg/mL) cannot be definitively linked to any specific component solely on the basis of this data.

**Table 2:** GC/MS Chemical analysis of [Sh cell] bioactive extract of the fungus “*Aspergillus unguis* isolate SP51-EGY”. Rt = Retention time, MW = Molecular weight, MF = Molecular Formula.

No	Rt	Compound	M W	M F	Area %
1	18.43	4,6,6-Trimethyl-2-(3-methylbuta-1,3-dienyl)-3-oxatricyclo- [5.1.0.0(2,4)]octane	218	C_15_H_22_O	0.86
2	19.82	4-Methyl(trimethylene)silyloxyoctane	214	C_12_H_26_OSi	0.99
3	22.38	1-Nitro-á-d-arabinofuranose, tetraacetate	363	C_13_H_17_NO_11_	0.35
4	26.93	1-(2'-Quinolyl)-3-methylazulene	296	C_20_H_15_N	0.58
5	27.80	19,19-Dimethoxy-3-oxoandrost-1-en-17-yl acetate	390	C_23_H_34_O_5_	0.16
6	30.24	2,4-diphenyl-glutaronitrile	246	C_17_H_14_N_2_	1.33
**[7]**	31.39	**7,9-Diethyl-2,4-bis(dimethylamino)-10-imino-8-thio-1,7,9-triazaspiro [4.5]-1,3-decadiene-6,8-dione**	**336**	**C**_**15**_**H**_**24**_**N**_**6**_**OS**	**4.24**
8	34.92	Hexane-2,5- -dihydroxy	262	C_12_H_30_O_2_Si_2_	6.99
9	38.23	6-Phenyldodecane	246	C_18_H_30_	0.53
10	38.88	4-Phenyldodecane	246	C_18_H_30_	0.72
11	39.74	2,13-Dithia(3)metacyclo(3)naphthalenophane	322	C_20_H_18_S_2_	7.74
12	42.45	2,6-Dibromophenyl 3-phenylpropanoate	382	C_15_H_12_Br_2_O_2_	0.61
13	43.64	Butanoic acid-heptafluoro-methyl ester	228	C_5_H_3_F_7_O_2_	0.4
**[14]**	44.85	**10,13-Octadecadiynoic acid, methyl ester**	**290**	**C**_**19**_**H**_**30**_**O**_**2**_	**0.2**
15	48.27	Glycocholic acid methyl ester	695	C_36_H_69_NO_6_Si_3_	0.13
16	51.08	Phenanthrene-9-dodecyltetradecahydro	360	C_26_H_48_	0.17
17	62.95	3',8,8'-Trimethoxy-3-piperidin-1-yl-2,2'-binaphthyl-1,1',4,4'-tetrone	487	C_28_H_25_NO_7_	0.21

The observed significant activity likely stems from the collective influence of the mixture, potentially involving synergistic interactions. The suggested structures, especially for the complex heterocycle [7], await conclusive verification through isolation, followed by NMR (1H, 13C, 2D) and HRMS analyses.

Compounds identification were performed using computational searches against user-constructed reference libraries (mainlib and Wiley9) that contained mass spectra. The seventeen compounds tentatively identified within the bioactive ‘Sh cell’ extract underwent an initial in silico assessment.

In silico studies, encompassing molecular docking, dynamic simulations, and virtual screening, offer a cost-effective and efficient approach to identify potential inhibitors^16^.

Despite our extract showing promising anticancer activity, the exact molecular mechanism by which it exerts its effect is not clearly understood. Potential drug target prediction was then carried out using a pharmacophore-mapping approach^17^. To generate a testable hypothesis regarding the extract’s mechanism of action, the compounds corresponding to the GC/MS peaks were investigated in silico for their potential binding affinity to CDK receptors.

Binding properties for identified compounds on potential targets were estimated by a reverse pharmacophore mapping server^18^.

The compounds corresponding to the GC/MS peaks were investigated for their binding affinity to CDK receptors:

Cyclin-dependent kinases (CDKs), particularly CDK-2 and CDK-1, significantly influence the control of the cell cycle, cellular proliferation, and cancer development.

Dysregulation of these kinases is commonly detected in Colorectal cancer, which results in unregulated cell proliferation and resistance to apoptosis^8^.

**Fig. 2:**
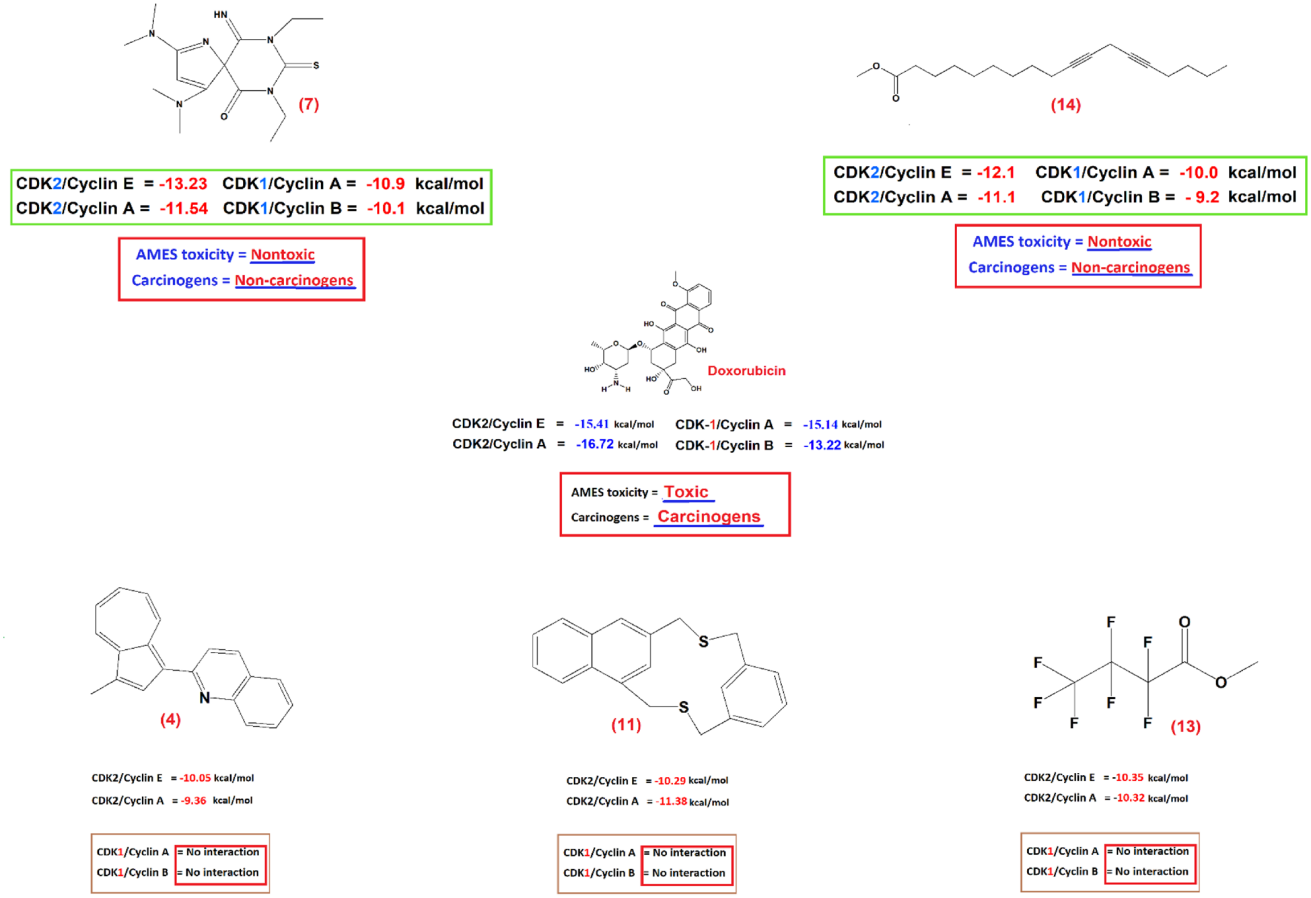
Structures of the NEW anti-cancer compounds identified by GC/MS analysis and a summary of AutoDock vina was used to evaluate the interaction free energies of these compounds with the targeted CDK receptors.

- Cyclin-dependent kinase-2 (CDK-2), in complex with cyclins E and A, is essential for cell cycle progression, particularly during the G1/S and S/G2 transitions. The dysregulation of CDK-2 activity is frequently observed in colorectal cancer (CRC), rendering it an attractive therapeutic target.
- Cyclin-dependent kinase-1 (CDK-1), in complex with cyclins A and B, plays a crucial role in the cell cycle, especially during the G2/M transition. The improper functioning of CDK-1-cyclin complexes is linked to uncontrolled growth in colorectal cancer (CRC), making them interesting targets for treatment.
- AutoDock vina was used to evaluate the interaction free energies of these compounds with the targeted CDK receptors:
- The docking results obtained via AutoDock Vina demonstrate that CDK2/Cyclin E/A of the compounds 7,9-diethyl-2,4-bis(dimethylamino)-10-imino-8-thio-1,7,9-triazaspiro[4.5]-1,3-decadiene-6,8-dione [7] (-13.23, -11.54 kcal/mol) and 10,13-octadecadiynoic acid, methyl ester [14] (-12.07, -11.07 kcal/mol) exhibit the highest docking scores (Table 3, Fig. 2).
- CDK1/Cyclin A/B of the chemical 7,9-diethyl-2,4-bis(dimethylamino)-10-imino-8-thio-1,7,9-triazaspiro[4.5] -1,3-decadiene-6,8-dione [7] (-10.93, -10.13 kcal/mol) and 10,13-octadecadiynoic acid, methyl ester [14>] (-10.02, -9.17 kcal/mol) produce the highest docking scores (Table 4, Fig. 2).
- The compounds; 1-(2'-Quinolyl) -3-methylazulene [4] (-9.36, -10.05 kcal/mol), 2,13-Dithia(3)metacyclo- (3)naphthalenophane [11] (-11.38, -10.29 kcal/mol), and Butanoic acid-heptafluoro-methyl ester [13] (-10.32, -10.35 kcal/mol) exhibit elevated docking scores in CDK2/Cyclin E/A, with no impact on CDK1/Cyclin A/B (Tables 3, 4; Fig. 2).
- Doxorubicin exhibited binding energies of (-16.72, -15.41 kcal/mol) in CDK2/Cyclin E/A and (-13.22, -15.14 kcal/mol) in CDK1/Cyclin A/B. However, in vitro analysis revealed an IC_50_ of 7.1, which is higher than that of the extract (IC_50_ = 3.49), and a significantly lower selectivity index (SI = 0.7) compared to the extract (SI = 23.34), in addition to its toxicity to normal cells (Tables 3, 4, Fig. 2).

**Table 3:** AutoDock vina flexible docking results for the compounds corresponding to the GC/MS peaks docked into CDK2/Cyclin E and CDK2/Cyclin A receptors.

	CDK2/Cyclin E						CDK2/Cyclin A					
	Hydrogen bonds between atoms of compounds and amino acids of receptor					S- score (binding energy) (kcal/mol)	Hydrogen bonds between atoms of compounds and amino acids of receptor					S- score (binding energy) (kcal /mol)
	Compounds	Receptor		Type	Distance (Å)		Compounds	Receptor		Type	Distance (Å)	
	Atoms	Atoms	Residues				Atoms	Atoms	Residues			
DOX	H9332	O853	Ser53	H-donor	1.98	**-16.72**	H 4743	O 1188	LEU 83	H-donor	1.84	**-15.41**
	H9334	O864	Leu54	H-donor	2.18		H 9091	O2085	Gln131	H-donor	2.14	
	H9330	OG7201	Ser227	H-donor	1.89		H 9064	OD2309	Asp145	H-donor		
	H9356	O7207	Pro228	H-donor	3.45		O9032	OH252	TYR15	H-donor	3.18	
	O9299	NZ5201	Lys108	H-donor	3.10		O9065	NZ540	LYS 33	H-donor	1.06	
	O9321	OG7294	Ser233	H-donor	3.05							
1	H9494	O2529	Pro155	H-donor	3.24	-8.43	–	–	–	–	–	–
2	H9305	NH2573	Arg157	H-acceptor	2.19	-8.12	–	–	–	–	–	–
3	O9316	OH2613	Tyr159	H-donor	3.03	-9.74	–	–	–	–	–	–
4	O9304	OH2612	Tyr159	H-acceptor	2.16	**-9.36**	O 9044	OH252	Tyr15	H-acceptor	2.92	**-10.05**
5	H9337	O2529	Pro155	H-donor	2.40	-9.66	O9033	Nz540	Lys33	H-acceptor	2.67	8.39
6	–	–	–	–	–	–	O9048	NZ540	Lys33	H-acceptor	2.02	-9.87
[7]	O9337	OH2953	Tyr179	H-donor	2.73	**-13.23**	H9106	OD1346	Asp 86	H-donor	2.99	**-11.54**
	O9337	O9008	Glu340	H-donor	2.81		O9060	NZ2062	Lys129	H-acceptor	3.09	
8	–	–	–	–	–	–	–	–	–	–	–	–
9	–	–	–	–	–	–	–	–	–	–	–	–
10	–	–	–	–	–	–	–	–	–	–	–	–
11	H9299	OE6571	Glu188	H-donor	2.57	**-11.38**	O 9040	OH252	Tyr15	H-acceptor	2.65	**-10.29**
12	–	–	–	–	–	–	–	–	–	H-acceptor	–	–
13	O9310	OH2612	Tyr159	H-donor	2.45	**-10.32**	N9045	N1335	Asp86	H-acceptor	2.81	**-10.35**
[14]	O9320	NH2466	Arg150	H-acceptor	3.13	**-12.07**	N9045	NE1331	Gln85	H-acceptor	3.05	**-11.07**
	O9319	OH2612	Tyr159	H-acceptor	2.77		N9046	N1335	Asp86	H-acceptor	2.78	
							N9045	NZ1406	Lys89	H-acceptor	2.43	
15	–	–	–	–	–	–	O9034	OH252	Tyr15	H-acceptor	2.54	-9.15
16	H9302	OE6570	Glu188	H-donor	3.86	-7.78	O9036	NZ540	Lys33	H-acceptor	2.74	-7.88
17	H9302	OE5931	Glu149	H-donor	2.08	-9.66	–	–	–	–	–	–

**Table 4:** AutoDock vina flexible docking results for the compounds corresponding to the GC/MS peaks docked into CDK1/Cyclin A and CDK1/Cyclin B receptors.

	CDK1/Cyclin A						CDK1/Cyclin B					
	Hydrogen bonds between atoms of compounds and amino acids of receptor					S- score (binding energy) (kcal/mol)	Hydrogen bonds between atoms of compounds and amino acids of receptor					S- score (binding energy) (kcal/mol)
	Compounds	Receptor		Type	Distance (Å)		Compounds	Receptor		Type	Distance (Å)	
	Atoms	Atoms	Residues				Atoms	Atoms	Residues			
DOX	H10318	OD2444	Asp146	H-don	1.75	**-13.22**	O10401	N2433	Asp146	H-acc	2.74	**-15.14**
	H10346	OD2444	Asp14	H-don	2.50							
	O10286	N238	Gly13	H-acc	2.93							
	O10316	NE2221	Gln132	H-acc	2.07							
1	O10367	OH278	Tyr15	H-donor	1.97	-7.71	O10283	NE2221	Gln132	H-acceptor	3.08	-8.08
2	H1073	O2213	Gln132	H-donor	1.77	-6.79	H10293	O2213	Gln132	H-donor	1.25	-6.71
3	O10384	OH278	Tyr15	H-donor	2.90	-9.40	H10304	OD1444	Asp86	H-donor	1.90	-7.63
4	–	–	–	–	–	–	–	–	–	–	–	–
5	–	–	–	–	–	–	–	–	–	–	–	–
6	–	–	–	–	–	–	–	–	–	–	–	–
[7]	O10407	OE2221	Gln 132	H-donor	2.24	**-10.93**	H10281	OE2224	Gln 132	H-donor	1.75	**-10.13**
							O10326	OD2444	Asp 146	H-donor	2.98	
8	–	–	–	–	–	–	–	–	–	–	–	–
9	O10361	O228	Glu12	H-donor	2.56	-9.36	–	–	–	–	–	–
10	H10374	O2213	Gln132	H-donor	2.13	-8.13	–	–	–	–	–	–
11	–	–	–	–	–	–	–	–	–	–	–	–
12	–	–	–	–	–	–	–	–	–	–	–	–
13	–	–	–	–	–	–	–	–	–	–	–	–
[14]	C10376	OH278	Tyr15	H-acceptor	3.29	**-10.02**	O10309	N1386	Leu83	H-acceptor	3.14	**-9.17**
15	–	–	–	–	–	–	–	–	–	–	–	–
16	–	–	–	–	–	–	–	–	–	–	–	–
17	–	–	–	–	–	–	–	–	–	–	–	–

### Limitations of docking scores and contextualization with known CDK inhibitors

- AutoDock Vina provides quick binding affinity assessments, but molecular docking scoring systems have limitations. Thus, high docking scores suggest binding rather than proving binding affinity or inhibitory efficacy. However, docking scores alone cannot predict cellular effectiveness, selectivity, or pharmacokinetics.
- Geometric complementarity and simplified energy calculations determine these scores, which may neglect important physicochemical events. These include explicit solvation effects, entropy variations, protein flexibility, and ligand-induced conformational changes. Thus, high docking scores suggest binding rather than proving binding affinity or inhibitory efficacy.
- Comparing compounds [7] and [14] to clinically utilized CDK inhibitors helps explain their promising docking scores. The CDK2/1 inhibitor Dinaciclib (SCH 727,965) and CDK2-specific inhibitor AT-7519 exhibit low nanomolar, experimental IC_50_ values against their targets.
- Similar in silico docking investigations using AutoDock Vina show these reference inhibitors docking at CDK2 or CDK1 active sites with scores between -10.0 and -12.0 kcal/mol.
- Our lead compounds demonstrated similar or better anticipated affinities, such as compound [7] with -13.23 kcal/mol against CDK2/Cyclin E and -10.93 against CDK1/Cyclin A. Target involvement is likely with this positive contrast.
- However, docking scores alone cannot predict cellular effectiveness, selectivity, or pharmacokinetics. We added molecular dynamics (MD) simulations and computationally costly MM/GBSA binding free-energy calculations to docking experiments to circumvent these limits and better assess binding stability and energetics.
- The stable binding and favorable interaction energies of [7] and [14] were confirmed by solvation and dynamic flexibility analyses, validating the docking predictions and bolstering their identification as promising dual CDK2/1 inhibitors.

### Molecular dynamics and system stability

- A Molecular Dynamics Simulation was conducted to predict the performance of the identified compounds upon binding to the protein’s active site, as well as their interaction and stability through simulation^19,20^.
- The validation of system stability is essential to trace disrupted motions and avoid artifacts that may develop during the simulation.

The RMSD, RMSF , ROG, and SASA for the human cyclin-dependent kinases (CDK-2 Cyclin E/A), (CDK-1 Cyclin A/B) receptors for compounds [7] and [14] were measured.

- This study assessed Root-Mean-Square Deviation (RMSD) to measure the systems’ stability during the 100 ns simulations.
- During MD simulation, (RMSF) assessing protein structural flexibility upon ligand binding is critical for examining residue behavior and their connection with the ligand^21^. Protein residue fluctuations were evaluated using the Root-Mean-Square Fluctuation (RMSF) algorithm to evaluate the effect of inhibitor binding towards the respective targets over 100 ns simulations.
- ROG was determined to evaluate overall system compactness as well as stability upon ligand binding during MD simulation^22,23^.
- The compactness of the protein hydrophobic core was examined by calculating the protein’s solvent accessible surface area (SASA). This was performed by measuring the surface area of the protein visible to the solvent, which is essential for biomolecule stability^24^.

**Fig. 3:**
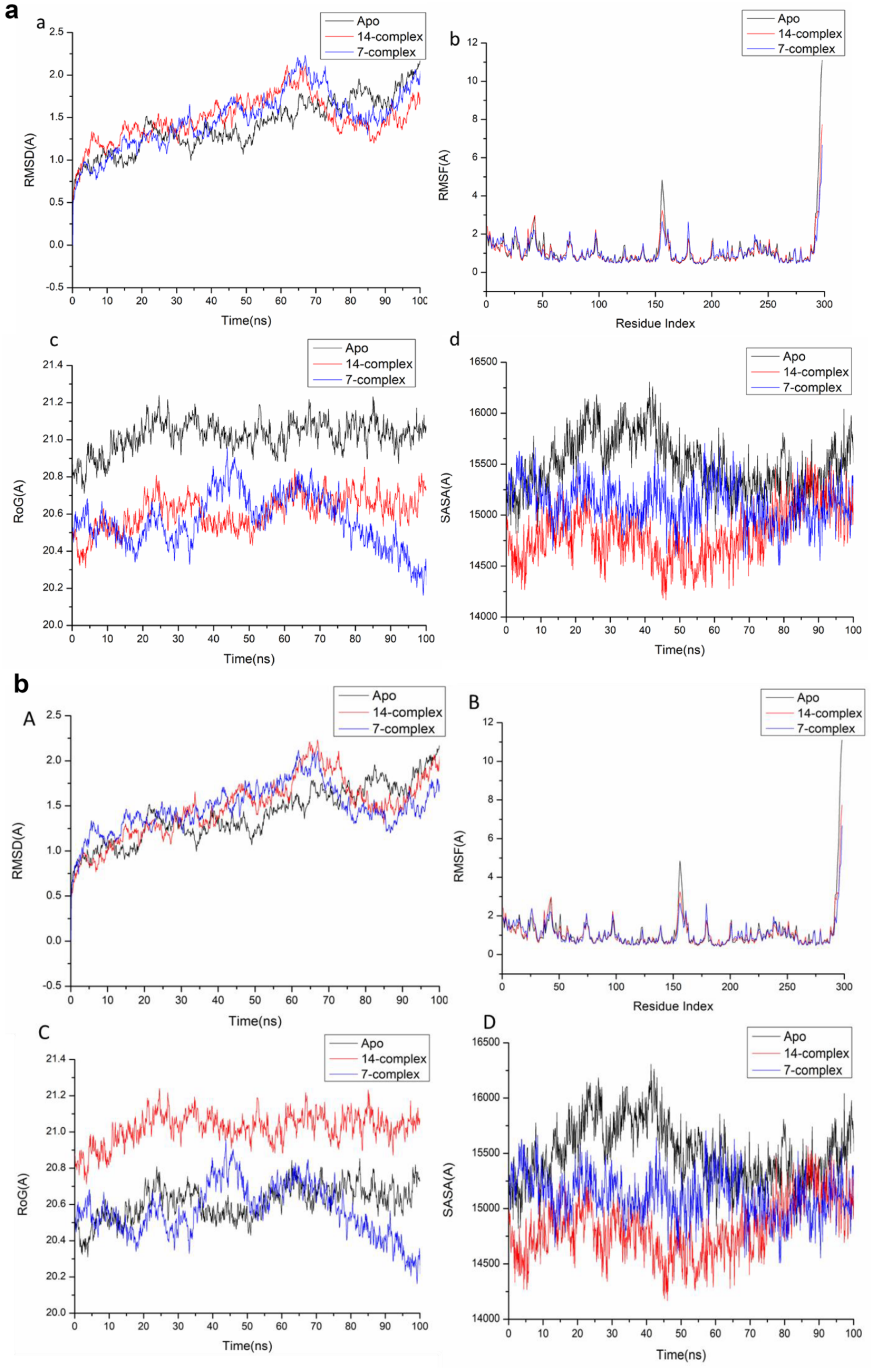

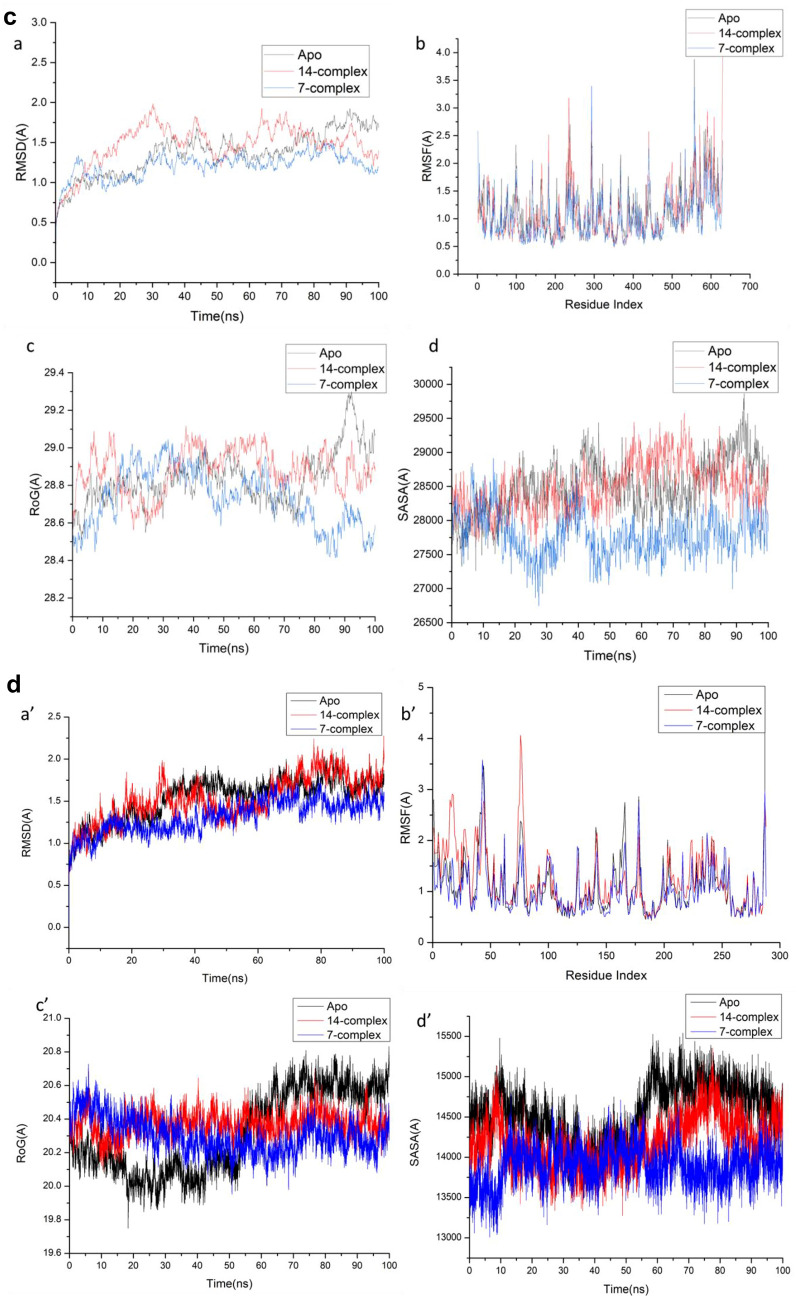
a (**a**) RMSD of Cα atoms of the protein backbone atoms. (**b**) RMSF of each residue of the protein backbone Cα atoms of protein residues (**c**) ROG of Cα atoms of protein residues; (**d**) solvent accessible surface area (SASA) of the C α of the backbone atoms relative (black) to the starting minimized over 100 ns for the ATP binding site of human cyclin-dependent kinase 2- Cyclin E (CDK2/ Cyclin E ) receptor with [7] (blue) and [14] (red). b. (**A**) RMSD of Cα atoms of the protein backbone atoms. (**B**) RMSF of each residue of the protein backbone Cα atoms of protein residues (**C**) ROG of Cα atoms of protein residues; (**D**) solvent accessible surface area (SASA) of the C α of the backbone atoms relative (black) to the starting minimized over 100 ns for the ATP binding site of human cyclin-dependent kinase 2- Cyclin A (CDK2 ) receptor with [7] (blue) and [14] (red). c. (**a**) RMSD of Cα atoms of the protein backbone atoms. (**b**) RMSF of each residue of the protein backbone Cα atoms of protein residues (**c**) ROG of Cα atoms of protein residues; (**d**) solvent accessible surface area (SASA) of the C α of the backbone atoms relative (black) to the starting minimized over 100 ns for the ATP binding site of human cyclin-dependent kinase 1 –cycline A (CDK1-cyclinA ) receptor with [7] (blue) and [14] (red). d. (**a**) RMSD of Cα atoms of the protein backbone atoms. (**b**) RMSF of each residue of the protein backbone Cα atoms of protein residues (**c**) ROG of Cα atoms of protein residues; (**d**) solvent accessible surface area (SASA) of the C α of the backbone atoms relative (black) to the starting minimized over 100 ns for the ATP binding site of human cyclin-dependent kinase 1 (CDK1 / CyclinB) receptor with [7] (blue) and [14] (red).

Table 5 and Fig. 3a–d in this study assessed (RMSD), (RMSF), (ROG), and (SASA) for CDK2/Cyclin E/A, and CDK1-cyclin A/B complexes.

**Table 5:** The RMSD, RMSF, ROG, and SASA for the human cyclin-dependent kinases (CDK2 Cyclin E/A), (CDK1 Cyclin A/B) receptors for compounds [7] and [14].

	Apo	7-complex	14-complex
CDK2/Cyclin E			
RMSD	1.54 ± 0.24 Å	1.32 ± 0.18Å	1.50 ± 0.27Å
RMSF	1.14 ± 0.44 Å,	1.02 ± 0.40 Å	1.10 ± 0.47 Å
ROG	20.97 ± 0.09 Å	20.46 ± 0.44Å	20.82 ± 0.22Å
SASA	15,442.44Å	15,177.77Å	15,036.96
CDK2/Cyclin A			
RMSD	1.41 ± 0.17 Å,	1.18 ± 0.11Å	1.39 ± 0.11Å
RMSF	0.99 ± 0.59Å	0.90 ± 0.44Å	0.91 ± 0.46Å
ROG	20.594 ± 0.08Å	20.186 ± 0.07Å	20.50 ± 0.08Å
SASA	14523Å	14,054.07 Å	14,284.97Å
CDK1-cyclin A			
RMSD	1.67 ± 0.30 Å	1.50 ± 0.26 Å	1.39 ± 0.23Å
RMSF	1.14 ± 0.44 Å	1.02 ± 0.40 Å	1.10 ± 0.47 Å
ROG	28.85 ± 0.11 Å	28.65 ± 0.15 Å	28.73 ± 0.19 Å
SASA	28,543 Å	28,615.9 Å	27,879.5 Å
CDK1-cyclin B			
RMSD	2.25 ± 0.82 Å	1.70 ± 0.34Å	1.87 ± 0.39Å
RMSF	1.23 ± 0.40 Å	1.21 ± 0.58Å	1.02 ± 0.46Å
ROG	20.40 ± 0.07Å	20.32 ± 0.24Å	20.00 ± 0.10Å
SASA	14,554.78 Å	13,896.71Å	14,196.39Å

The results of the system stability validation (Table 5, Fig. 3a-d) revealed that the compound [7]-bound protein complex system acquired a relatively more stable conformation, a lower residue fluctuation, and a highly stiff structure than the other studied systems.

The binding interaction mechanism based on binding free energy calculation:

A popular method for determining the free binding energies of small molecules to biological macromolecules is the molecular mechanics energy technique (MM/GBSA), which combines the generalized Born and surface area continuum solvation. It may be more trustworthy than docking scores^25^.

The MM-GBSA program in AMBER18 was used to calculate the binding free energies by extracting snapshots from the trajectories of the systems. As shown in Table 6, all the reported calculated energy components (except ΔG_solv_) gave high negative values, indicating favorable interactions.

**Table 6:** Shows the calculated energy binding for the target compounds against the target receptors.

	CDK2/CyclinE					CDK2/CyclinA				
Complex	ΔE_vdW_	ΔE_elec_	ΔG_gas_	ΔG_solv_	ΔG_bind_	ΔE_vdW_	ΔE_elec_	ΔG_gas_	ΔG_solv_	ΔG_bind_
Energy components (kcal/mol)										
7- CDK2	-40.17 ± 0.60	-5.25 ± 0.73	-45.42 ± 1.07	12.62 ± 0.68	-32.80 ± 0.52	-51.31 ± 0.40	-5.58 ± 0.40	-56.90 ± 0.49	14.89 ± 0.28	-42.01 ± 0.44
14- CDK2	-34.05 ± 0.40	-77.01 ± 0.21	-111.06 ± 0.22	95.98 ± 0.71	-15.07 ± 0.42	-38.96 ± 0.34	-9.86 ± 0.16	-48.82 ± 0.55	21.31 ± 0.41	-27.51 ± 0.37

	CDK1-cyclinA					CDK1-cyclin B				
Complex	ΔE_vdW_	ΔE_elec_	ΔG_gas_	ΔG_solv_	ΔG_bind_	ΔE_vdW_	ΔE_elec_	ΔG_gas_	ΔG_solv_	ΔG_bind_
7- CDK1	-48.35 ± 0.51	-13.49 ± 0.78	-61.85 ± 0.88	20.69 ± 0.64	-41.15 ± 0.44	-52.59 ± 0.85	-13.12 ± 0.65	-65.71 ± 0.88	19.21 ± 0.78	-46.50 ± 0.13
14- CDK1	-45.99 ± 0.34	-7.86 ± 0.74	-53.86 ± 0.76	15.34 ± 0.60	-38.51 ± 0.38	-33.34 ± 0.61	-13.90 ± 0.18	-47.24 ± 0.03	23.48 ± 0.77	-23.76 ± 0.06

∆E_vdW_ = van der Waals energy; ∆E_elec_ = electrostatic energy; ΔG_gas_ = gas free energy; ∆G_solv_ = solvation free energy; ∆G_bind_ = calculated total binding free energy.

The results indicated that binding affinity of [7]- CDK2/CyclinE and [14]- CDK2/CyclinE systems were -32.80 kcal/mol and -15.07 kcal/mol, respectively, Where the the binding affinity of [7]- CDK2/CyclinA and [14]- CDK2/CyclinA systems were -42.01 kcal/mol and -27.51 kcal/mol, respectively. Furthermore, the binding affinity of [7]-CDK1/CyclinA and [14]-CDK1/CyclinA systems was -41.15 kcal/mol and-38.51 kcal/mol, respectively. Finally, the binding affinity of [7]-CDK1/CyclinB and [14]-CDK1/CyclinB systems was -46.05 kcal/mol and -23.76 kcal/mol, respectively (Table 6).

An in-depth analysis of each energy contribution, which results in the reported binding free energies, demonstrates that the interactions between the [7] and [14] compounds and the target receptor protein residues are driven by the more positive van der Waals energy (Table 7).

**Table 7:** Drug likeliness properties of extract compounds.

	[7]	[14]	Doxorubicin		[7]	[14]	Doxorubicin
Molecular weight (g/mol)	336.46	290.44	532.52	Num. of H-bond donor	1	0	6
Num. of heavy atoms	23	21	39	Log Po/w	0.93	5.44	2.58
Num. of rotatable bonds	4	11	5	Num. of violations	1	1	3
Num. of H bond acceptor	3	2	12				

Identification of the critical residues responsible for ligand binding:

The total energy involved when compounds [7] and [14] bind these enzymes was further decomposed into the involvement of individual site residues to get more knowledge about essential residues involved in the inhibition of the ATP binding site of CDK2–Cyclin E/A and CDK1–Cyclin A/B receptors, resulting in G1/S and G2/M cell cycle arrests^26^.

**Fig. 4:**
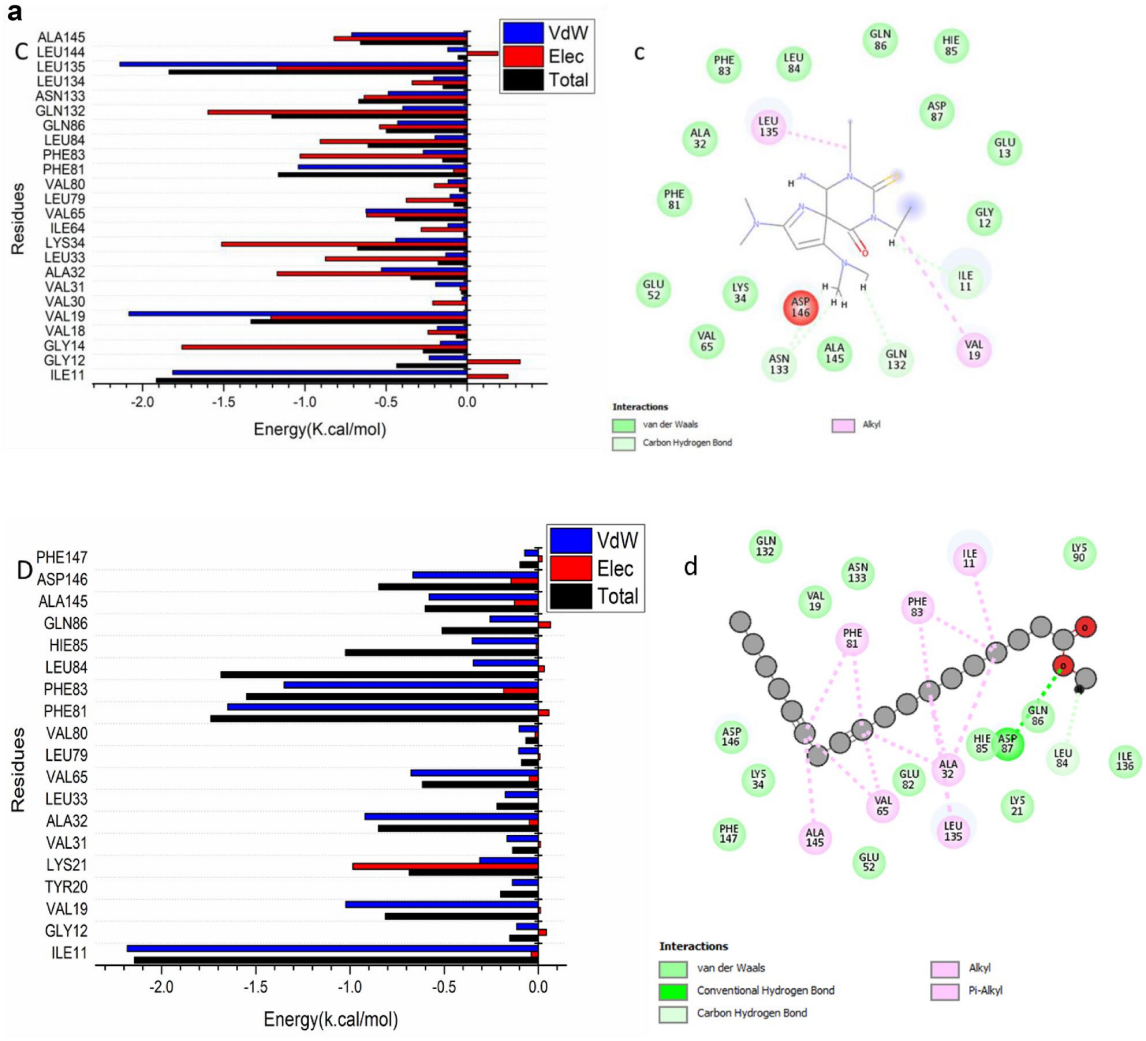

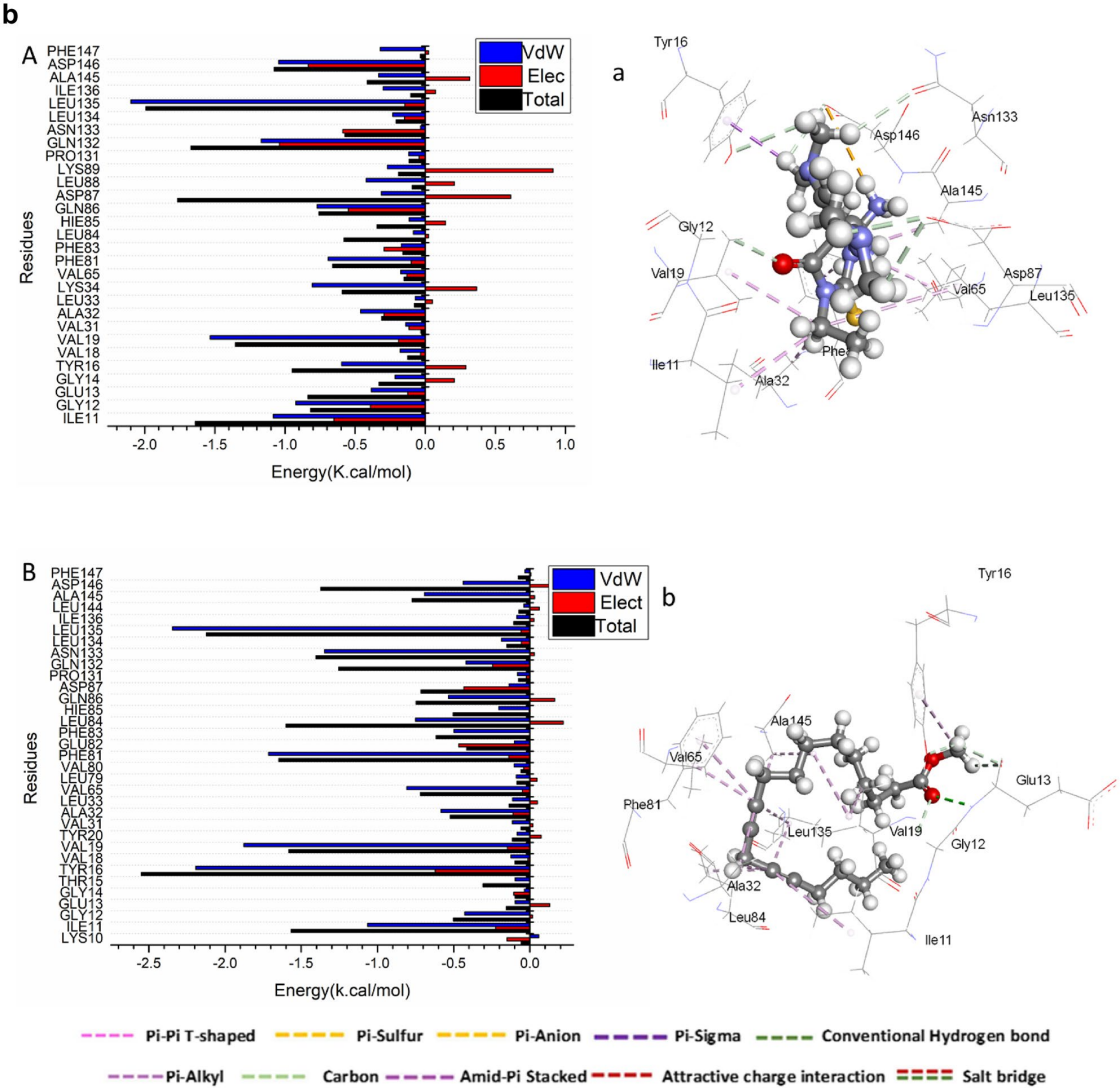

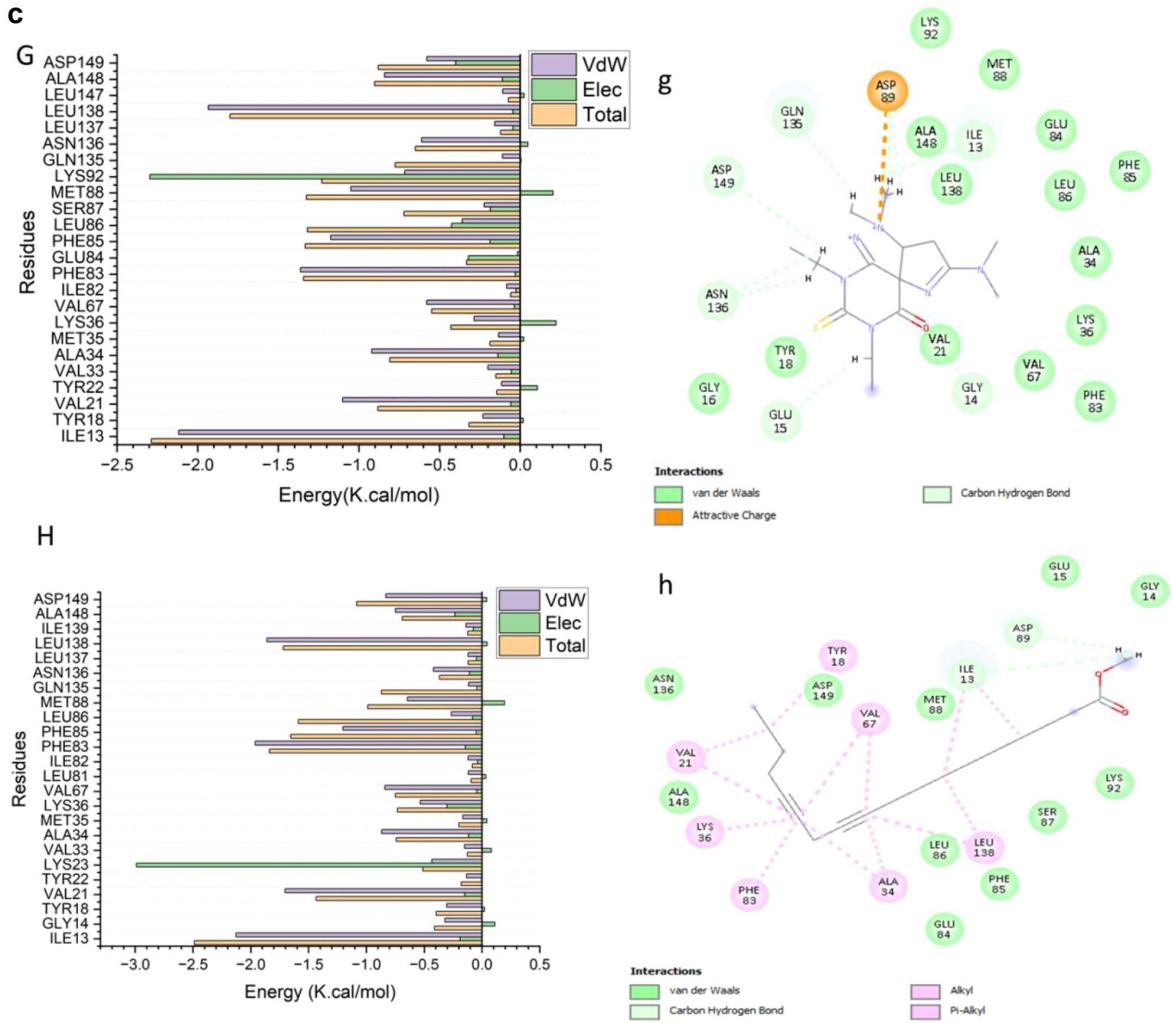

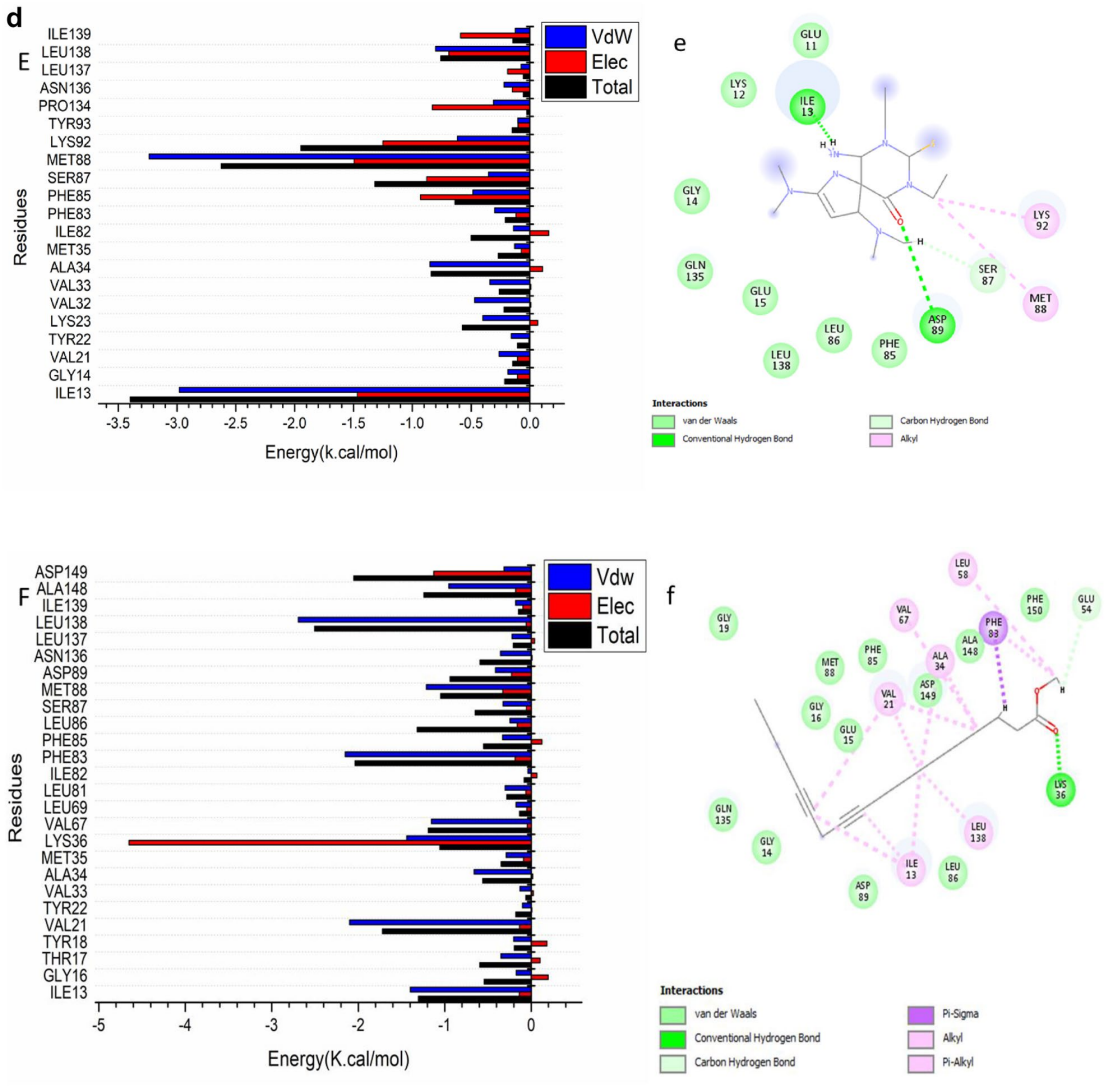
a: Per-residue decomposition plots showing the energy contributions to the binding and stabilization of [7] [**A**] ,and [14] [**B**] to the ATP binding site of CDK2- Cyclin E receptor . b.: Per-residue decomposition plots showing the energy contributions to the binding and stabilization of [7] [**A**], and [14] [**B**] to the ATP binding site of CDK2 – Cyclin A receptor . c. Per-residue decomposition plots showing the energy contributions to the binding and stabilization of [7] [**A**], and [14] [**B**] to the ATP binding site of CDK1/Cycline A receptor. d.: Per-residue decomposition plots showing the energy contributions to the binding and stabilization of [7] [**A**],and [14] [**B**] to the ATP binding site of CDK1/Cycline B receptor.

- From Fig. 4a[A], the significant favorable contribution of compound-[7] to the ATP binding site receptor of CDK2- CyclinE receptor is predominantly observed from residues Ile11 (-1.814 kcal/mol), Gly 12 (-0.234 kcal/mol), Val19 (-2.085 kcal/mol), Val31 (-0.194 kcal/mol), Ala32 ( -0.529 kcal/mol), Leu 33 ( -0.132 kcal/mol), Lys 34 ( -0.44 kcal/mol), , Ala32 ( -0.529 kcal/mol), Ile 64 ( -0.119 kcal/mol), Val 65 (-0.624 kcal/mol), Leu 79 (-0.105 kcal/mol), Val 80 (-0.117 kcal/mol), Phe 81 (-1.041 kcal/mol), Phe83 (-0.27 kcal/mol), Leu 84 (-0.198 kcal/mol), Gln 86 ( -0.428 kcal/mol), Gln 132 (-0.397 kcal/mol), Asn 133 (-0.486 kcal/mol), Leu 134 (-0.207 kcal/mol), Leu 135 (-2.14 kcal/mol), Leu 144 (-0.12 kcal/mol), and Ala 145 ( -0.712 kcal/mol).
- In addition, from Fig. 4a[B],.: the significant favorable contribution of compound-[14] to the ATP binding site receptor of CDK2- Cyclin E receptor is predominantly observed from residues Ile11 (-2.183 kcal/mol), Gly 12 (-0.115 kcal/mol), Val19 (-1.024 kcal/mol), Tyr 20 (-0.138 kcal/mol), Lys 21 (-0.311 kcal/mol), Val31 (-0.166 kcal/mol), Ala32 ( -0.921 kcal/mol), Leu 33 ( -0.176 kcal/mol), Val 65 (-0.676 kcal/mol), Leu 79 (-0.105 kcal/mol), Val 80 (-0.103 kcal/mol), Phe 81 (-1.649 kcal/mol), Phe83 (-1.349 kcal/mol), Leu 84 (-0.346 kcal/mol), Hie 85 (-0.351 kcal/mol), Gln 86 ( -0.256 kcal/mol), Ala 145 ( -0.58 kcal/mol), Asp 146 (-0.666 kcal/mol), and Phe147 (-0.346 kcal/mol).
- In addition, from Fig. 4b[A], the significant favorable contribution of compound-[7] to the ATP binding site receptor is predominantly observed from residues Ile 11(-1.084 kcal/mol), Gly 12 (-0.922 kcal/mol), Glu 13 (-0.385 kcal/mol), Gly14 ( -0.215 kcal/mol), Tyr16 (-0.597 kcal/mol), Ala32 (-0.461 kcal/mol), Lys34 (-0.805 kcal/mol), Phe 81 (-0.693 kcal/mol), Gln86 (-0.771 kcal/mol), Leu 88 (-0.42 kcal/mol), Gln132 (-1.17 kcal/mol), Leu135 (-2.099 kcal/mol), Ile136 (-0.301 kcal/mol), Ala145 (-0.332 kcal/mol), Asp 146 (-1.045 kcal/mol), and Phe147 (-0.32 kcal/mol).
- On the other hand, From Fig. 4b[B], the significant favorable contribution of compound-[14] to the ATP binding site receptor of CDK2- Cyclin A receptor is predominantly observed from residues Ile11 (-1.065 kcal/mol), Gly 12 (-0.427 kcal/mol), Tyr16 (-2.192 kcal/mol), Val19 (-1.877 kcal/mol), Ala32 ( -0.583 kcal/mol), Val 65 (-0.808 kcal/mol), Phe 81 (-1.715 kcal/mol), Phe83 (-0.498 kcal/mol), Leu 84 (-0.75 kcal/mol), His85 (-0.204 kcal/mol), Gln 86 ( -0.535 kcal/mol), Gln 132 (-0.419 kcal/mol), Asn 133 (-1.348 kcal/mol), Leu 135 (-2.345 kcal/mol), Ala 145 ( -0.691 kcal/mol), and Asp 146 (-0.437 kcal/mol).
- In addition, the significant favorable contribution of compound-[7] to the ATP binding site receptor of CDK1/Cycline A receptor from Fig. 4c[A], is predominantly observed from residues Ile13 (-2.117 kcal/mol), Tyr 18 (-0.231 kcal/mol), Val21 (-1.01 kcal/mol), Tyr 22 (-0.115 kcal/mol), Lys 23 (-0.4 kcal/mol), Val33 (-0.2 kcal/mol), Ala34 ( -0.92 kcal/mol), Met 35 ( -0.134 kcal/mol), Lys 36 ( -0.286 kcal/mol), Val 67 ( -0.58 kcal/mol), Phe 83 (-1.362 kcal/mol), Phe 85 (-1.175 kcal/mol), Ser 87 (-0.223 kcal/mol), Met 88 (-1.05 kcal/mol), Lys 92 (-0.716 kcal/mol), Asn 136 ( -0.612 kcal/mol), Ala 148 ( -0.841 kcal/mol), and Asp 149 (-0.579 kcal/mol).
- Finally, the significant favorable contribution of compound-[14] to the ATP binding site receptor of CDK1/Cycline A receptor from Fig. 4c[B],.: is predominantly observed from residues Ile13 (-2.128 kcal/mol), Gly 14 (-0.32 kcal/mol), Tyr 18 (-0.305 kcal/mol), Val21 (-1.703 kcal/mol), Tyr 22 (-0.134 kcal/mol), Val 33 (-0.151 kcal/mol), Ala34 ( -0.869 kcal/mol), Met 35 ( -0.166 kcal/mol), Lys 36 (-0.534 kcal/mol), Val 67 (-0.841 kcal/mol), Ser 87 (-0.325 kcal/mol), Phe 83 (-1.961 kcal/mol), Phe 85 (-1.202 kcal/mol), Leu 86 (-0.265 kcal/mol), Met 88 (-0.645 kcal/mol), Leu 138 ( -1.861 kcal/mol), Ile 139 (-0.139 kcal/mol), Ala 148 (-0.748 kcal/mol), and Asp 149 ( -0.831 kcal/mol).
- In addition, from Fig. 4d[A], the significant favorable contribution of compound-[7] to the ATP binding site receptor of CDK1/Cycline B receptor is predominantly observed from residues Ile13 (-2.981 kcal/mol), Gly 14 (-0.187 kcal/mol), Val21 (-0.262 kcal/mol), Tyr 22 (-0.158 kcal/mol), Lys 23 (-0.4 kcal/mol), Val32 (-0.47 kcal/mol), Val33 (-0.34 kcal/mol), Ala34 ( -0.85 kcal/mol), Met 35 ( -0.13 kcal/mol), Ile 82 (-0.14 kcal/mol), Phe 83 (-0.3 kcal/mol), Phe 85 (-0.485 kcal/mol), Ser 87 (-0.353 kcal/mol), Met 88 (-3.237 kcal/mol), Lys 92 (-0.617 kcal/mol), Pro 134 (-0.31 kcal/mol), Asn 136 ( -0.22 kcal/mol), Leu 138 ( -0.8 kcal/mol), and Ile 139 (-0.124 kcal/mol).
- Furthermore, the significant favorable contribution of compound-[14] to the ATP binding site receptor of CDK1/Cycline B receptor Fig. 4d[B],.: is predominantly observed from residues Ile13 (-1.398 kcal/mol), Gly 16 (-0.173 kcal/mol), Thr 17 (-0.35 kcal/mol), Tyr 18 (-0.203 kcal/mol), Val21 (-2.1 kcal/mol), Tyr 22 (-0.101 kcal/mol), Val 33 (-0.129 kcal/mol), Ala34 ( -0.66 kcal/mol), Met 35 ( -0.289 kcal/mol), Lys 36 (-1.439 kcal/mol), Val 67 (-1.154 kcal/mol), Leu 69 (-0.177 kcal/mol), Leu 81 (-0.301 kcal/mol), Phe 83 (-2.152 kcal/mol), Phe 85 (-0.33 kcal/mol), Leu 86 (-0.245 kcal/mol), Ser 87 (-0.325 kcal/mol), Asp 89 (-0.414 kcal/mol), Asn136 (-0.355 kcal/mol), Leu 137 (-0.223 kcal/mol), Leu 138 ( -2.692 kcal/mol), Ile 139 (-0.181 kcal/mol), Ala 148 (-0.953 kcal/mol), and Asp 149 ( -0.313 kcal/mol).

The unregulated growth of colorectal cancer (CRC) is linked to the overproduction of two crucial cell cycle regulators, CDK-2 and CDK-1. If these kinases are blocked, neoplastic cells may undergo cell cycle arrest and die.

- Numerous fungal drugs have shown dual inhibitory effects on these CDKs^8^. Pang et al.^27^ discovered that an excess of Cyclin E is associated with more advanced tumor stages and disease metastasis, resulting in a worse prognosis for patients with colorectal cancer (CRC).
- DNA replication and S-phase advancement are regulated by CDK2-Cyclin A. Patients with colorectal cancer who have greater levels of Cyclin A typically have worse outcomes and faster tumor progression, according to Bahnassy et al.^28^. CDK2 may be a potential therapeutic target, as demonstrated by Zaki et al. (2023), who found that CDK2 inhibition inhibits the growth of CRC cells^29^.
- In colorectal cancer (CRC), the G2/M transition and mitotic progression are strongly impacted by the CDK1/cyclin A and CDK1/cyclin B complexes. Deregulation promotes unchecked growth, metastasis, and genetic instability. There is evidence that CDK1 plays a role in tumor survival; colorectal cancer cells undergo apoptosis when CDK1 is inhibited^30^.
- Since cyclin-dependent kinases (CDKs) like CDK-2 and CDK-1 are essential for the development of colorectal cancer (CRC).

Fungal extracts have attracted considerable attention as potential sources of natural compounds with anticancer properties^31^. To contextualize our findings within the broader field, previous studies have identified other fungal metabolites with CDK-inhibitory activity.

- For example, roseopurpurin C from *Aspergillus nidulans* was shown to inhibit CDK2-cyclin E/A^32^, and fumitremorgin-type alkaloids from *A. fumigatus* inhibit CDK2 phosphorylation^31^. While these studies demonstrate the potential of *Aspergillus* species to produce CDK-inhibitory compounds.
- The metabolites and mechanism proposed here for *A. unguis* isolate SP51-EGY are distinct. This highlights the structural and functional diversity within fungal metabolites and underscores the novelty of the lead compounds identified in our study.

The levels of the cell-cycle regulatory protein CDK1 were markedly reduced by Asperteretal M, which is derived from a culture of the marine fungus *A. terreus*. This impact grew as the concentration of the compound rose^33^. According to the data, heptelidic acid caused cell cycle arrest, especially in the G2/M phase, which raised the levels of cyclin B1^34^. Butyrolactone-I, one of the earliest naturally occurring compounds known to block CDK1 and CDK2 enzymes, was produced by *Aspergillus terreus*. It has been discovered that this substance competes with ATP to disrupt the CDK1/cyclin B complex and prevent the phosphorylation of RB in the cells that are treated. As shown in Butyrolactone-I, which was found to suppress CDKN1A (p21WAF1) expression via a mechanism possibly involving cellular targets beyond CDK1, extended treatment has produced both G1 and G2/M cell cycle arrests and apoptotic induction, much like the trisubstituted purines. The expression of CDKN1A (p21WAF1) was shown to be significantly inhibited by butyrolactone-I^26^. These findings are consistent with prior reports that our extract reduces CRC cell proliferation (IC_50_ = 3.49 µg/mL), with notable safety towards normal cells with a selectivity index (SI = 23.33), indicating selective protection of normal cells.

-It was reported that CDK1/cyclin A and CDK1/cyclin B complexes substantially influence the G2/M transition and mitotic progression in colorectal cancer (CRC). Deregulation promotes genomic instability, metastasis, and uncontrolled proliferation^30^. The commencement of mitosis depends on CDK1-Cyclin B^8^. Evidence suggests that CDK1 plays a role in tumor survival; its inhibition triggers apoptosis in colorectal cancer cells^30^.

- A compound inhibits CDK2-cyclin E/A by binding to the ATP-binding pocket, thereby suppressing CRC proliferation^32^. *Aspergillus terreus* yielded Butyrolactone-I, one of the first natural chemicals known to inhibit CDK1 and CDK2 enzymes. This compound has been identified as a competitor of ATP, inhibiting the CDK1/cyclin B complex and preventing the phosphorylation of RB in the affected cells. Analogous to trisubstituted purines, extended treatment has led to G1 and G2/M cell cycle arrests and the induction of apoptosis^26^.
- Fungal factories can produce bioactive natural compounds. Endophyte *Aspergillus* TRL1 yielded pulchranin A. Pulchranin A showed in vitro cytotoxicity in breast (MCF-7), liver (Hep-G2), and colorectal (HCT) cell lines, with IC_50_ values of 63, 80, and 91 µg/mL. Additionally, it inhibited three cyclin-dependent kinases (CDK1, CDK2, and CDK4) in MCF-7 cells with IC_50_ values of 9.82, 15.6, and 2.7 µg/mL. In addition, in-silico molecular docking of pulchranin A with CDK1, CDK2, and CDK4 crystal structures showed favorable hydrogen bonding, hydrophobic contacts, and Pi-Pi stacking with certain amino acid residues. Pulchranin A may suppress breast cancer CDKs^35^.
- In human colon cancer cell lines, Flavopiridol (Alvocidib) from *Aspergillus* sp. inhibits CDK1, CDK2, CDK4/6, and CDK9 to stop the cell cycle and induce apoptosis^9^. Ganoderic acids from *Ganoderma lucidum* affect CDK-2 and CDK-1, causing colorectal cancer cell death^36^.
- In silico methods were used to evaluate the CDK1/Cks2 inhibitory potential of *Securigera securidaca* L. (S. securidaca) seeds, used in medicine for numerous disorders, including cancer^37,38^. Gingerone had 25.67% prevalence, while hippeastrine had 2%. Gingerol, eugenol, and α-curcumene made about 52% of the volatile extract composition in the essential extract. Hippeastrine and naringenin had drug-like characteristics and molecular interactions, with pharmacokinetic and toxicological profiles similar to dinaciclib, according to molecular docking and ADMET analyses targeting CDK1.
- The 100 ns molecular dynamics (MD) simulation showed that both drugs maintained stable conformations in the binding region, suggesting they might stabilize the protein structure by lowering CDK1 backbone flexibility.
- Hippeastrine and naringenin’s stability in CDK1 complexes was confirmed by MM-PBSA simulations. Hippeastrine and naringenin have CDK1 inhibitor potential due to their active site binding affinity and stability^39^.
- Polyphenols from mulberry leaf extract suppress tumor cell proliferation, enhance p53 phosphorylation, increase CDKN1A (p21) and CDKN1B (p27) expression, decrease CDK-2 and CDK-4 activity, and block RB phosphorylation^40^.
- According to various evaluations, *Brassicaceae* plants like cabbage, radishes, cauliflower, broccoli, Brussels sprouts, and daikon contain indole-3-carbinol, which inhibits breast, prostate, endometrial, colon, and leukemia tumor cell growth^41^. Indole-3-carbinol downregulates cyclins D1, E, CDK-2, -4, and -6 and upregulates CDKN1A (p21), 1B (p27), and 2B (p15) to stop the G1/S phase^41^.
- Silibinin, the most active component of milk thistle (*Silybum marianum*) crude seed extract, induces cell cycle arrest in the G2-M phase in human colon cancer FET and GEO cell lines and in the G1 phase in HCT116 cells^42^. Silibinin lowered B1, D1, and CDK-2 levels. Silibinin also upregulated cell cycle inhibitors CDKN1A (p21) and CDKN1B (p27)^42^.
- Wogonin, a *Scutellaria radix* monoflavonoid, inhibits colorectal HCT116 cell growth. Wogonin inhibited cyclin A, E, D1, CDK-2, and CDK-4^43^.
- Acetylbritannilactone, produced from *Inula britannica* L., arrested the cell cycle in G1 phase, reduced cyclins A, D1, and E, and CDK-2, -4, and -6, and increased CDKN1A (p21)^44^.
- Apigenin, a fruit and vegetable flavonoid, stops the G2/M phase in p53-mutant cancer cell lines HT-29 and MG63 and increases CDKN1A protein^45^. Apigenin elevated CDKN1A and 1B and decreased cyclins D1, D2, and E, as well as CDK-2, -4, and -6, to limit prostate tumor xenograft proliferation in athymic nude mice^46^. In human pancreatic and hepatoma cancer cell lines, apigenin stopped the cell cycle at G2/M, decreased cyclin A, B, phosphorylated CDC2, and CDC25, and increased CDKN1A^47^.
- The integrated computational and cellular data support a model in which compound [7] is predicted to antagonize ATP to block the CDK2/cyclin A, CDK1/cyclin A, and CDK1/cyclin B complexes, interrupting the G1/S and G2/M cell cycle.
- For general mechanistic comparison, a wide range of natural products from plants have been reported to modulate CDK activity or induce cell cycle arrest. These include roscovitine (a semisynthetic CDK2 inhibitor derived from a plant precursor), silibinin^43^, wogonin^43^, and apigenin^45–47^. These examples from diverse chemical classes illustrate the established principle that natural products can target cell cycle regulators.
- Our computational predictions align with this broader mechanistic paradigm, suggesting that the putative compounds from *A. unguis* may act via a similar strategy—competition with ATP at the kinase active site—to induce cell cycle arrest. This positions our fungal-derived leads within a known pharmacological framework while emphasizing their novel structural origin.
- In summary, while the cited literature provides precedents for CDK inhibition by fungal and plant-derived natural products, the specific compounds, structural motifs, and extract source (*A. unguis* SP51-EGY) reported here are novel. Our computational predictions align with established mechanisms of known CDK inhibitors, suggesting that compounds [7] and [14] may compete with ATP to block the CDK2/cyclin A and CDK1/cyclin A/B complexes, resulting in both G1/S and G2/M cell cycle arrests. This hypothesis, generated from our integrated in silico and cellular data, now requires direct biochemical validation specific to this extract and its purified constituents.

### Pharmacokinetics study

SWISSADME and admetSAR webservers were used to assess the identified compound-likeness characteristics, and ADME (Adsorption, Distribution, Metabolism, Excretion)^48,49^.

To comprehend Lipinski’s "rule of 5" and the quantity of free rotatable bonds, the drug likeness characteristics of the identified compound were assessed.

#### Drug likeliness

The Lipinski rule of five filters is used to assess the drug-likeness of the identified compounds that were chosen.

The rule of five states that a molecule is considered drug-like if it satisfies two or more of the following criteria:

- Its Lipophilicity (expressed as Log P) must be less than 5,
- Its molecular weight must be less than 500 Dalton,
- Its number of hydrogen donors must be less than 5, and so on^50^.
- The class of toxicity values was derived from the AdmetSAR data.

This analysis aids in the differentiation of molecules that resemble drugs from those that do not. Table 8 shows that all the identified compounds followed Lipinski’s rule of five.

- The molecular masses of [7, 14] are 336.46 and 290.44 g/mol, respectively.
- Compound [14] violates the LogP values by being assigned a value greater than 5, which indicates its hydrophobic nature. The high LogP value of compound [14] is because of the long alkyl chain, whereas in compound [7], the presence of hydrophilic amino groups supports the low LogP value.
- LogP value: It must be noted that at times, in the case of natural products, Lipophilicity does not directly relate to its physicochemical profile, violating the rule of five^51^.
- The number of hydrogen bond acceptors for [7] and [14] is 3 and 2, respectively.
- Hydrogen bond donors of [7] and [14] are 1 and 0, respectively.
- The number of rotatable bonds of the [7] and [14] molecules is 4 and 11, respectively, indicating the flexibility of some molecules compared to others.

**Table 8:** ADME profile prediction of the extracted compounds.

	[7]	[14]	Doxorubicin		[7]	[14]	Doxorubicin
Log S	-1.83	-5.38	-3.91	Metabolism (cytochrome P450)			
Solubility class	Very soluble	Moderately soluble	Soluble	2C9 SUB	Non-sub	Non-sub	Non-sub
Absorption				C2DR SUB	Non-sub	Non-sub	Non-sub
BBB	BBB + 0.5420	BBB + 0.542	0.9951	3A4 SUB	Sub	Sub	sub
HIA	(HIA) + 0.9540	(HIA) + 0.9548	0.8092	1A2 INH	Non-inhibitor	Non-inhibitor	Non-inhibitor
CaCo-2	caco2 + 0.4927	caco2 -0.568	0.7990	2C9 INH	Non-inhibitor	Non-inhibitor	Non-inhibitor
ROCT	Non-Inh	Non-Inh	Non-Inh	2D6 INH	Non-inhibitor	Non-inhibitor	Non-inhibitor
Distribution				2C19 INH	Non-inhibitor	Non-inhibitor	Non-inhibitor
p-glyco substrate	None	Inhibitor	Inhibitor	3A4 INH	Non-inhibitor	Non-inhibitor	Non-inhibitor
p-glyco Inhibitor	None	None	Yes				

### Implications of ADMET profiles for drug development

In silico ADMET profiling of drugs [7, 14] gives crucial preclinical drug candidate insights. Both compounds’ predicted human intestinal absorption (HIA +) and blood–brain barrier permeability (BBB +) suggest promising bioavailability and the potential to reach peripheral and central nervous system targets, which could treat colorectal cancer metastases.

The main difference is lipophilicity:

Compound [7] meets Lipinski’s Rule of Five with a balanced profile, moderate LogP (0.93), good solubility, and no projected toxicity. This profile matches successful oral medicines, suggesting a simple formulation and development approach.

Compound [14] is more complicated but not disqualifying. The high anticipated LogP (5.44) suggests hydrophobicity, which may explain its robust membrane permeability and potent target binding (as demonstrated in docking and MM/GBSA), but raises concerns. Probability of promiscuous off-target binding, metabolic clearance, and solubility-limited absorption increase with lipophilicity. However, its expected P-glycoprotein inhibitor status may cause drug-drug interactions with co-administered substrates. The absence of projected mutagenicity (AMES toxicity) or carcinogenicity for both chemicals is a strong predictor of safety, unlike doxorubicin’s predicted toxicity.

Synthesis and Forward Look: These studies catalog properties and guide strategic development. Compound [7] is a lead-like compound appropriate for quick study. The profile suggests compound [14] is a high-potency, high-permeability hit that could be developed by lead optimization, specifically synthetic modification to reduce LogP (e.g., by adding polar groups or shortening the aliphatic chain) while preserving its core inhibitory pharmacophore. The complementing characteristics of [7] and [14]—one “drug-like” and the other “potency-driven”—offer ideal beginning points for a medicinal chemistry campaign to create selective, bioavailable, and safe dual CDK1/2 inhibitors for colorectal cancer.

#### ADMET analysis

To understand the pharmacokinetic properties of the molecules, ADMET analysis was performed.

Solubility expressed as Log S is an essential parameter of a drug-like molecule, and its value ideally varies from − 0.5 to − 5.5^52^. The solubility of compounds [7] and [14] falls in the range mentioned above.

Once the drug is administered orally, it is absorbed in the intestine to reach specific targets. All molecules have shown positive results concerning human intestinal absorption (HIA). The parameters, such as blood–brain barrier (BBB) and colorectal carcinoma (Caco-2), have been studied to assess the permeability of the membrane^53^.

- The BBB and Caco-2 values for compound [7] show positive values, hence it can cross the barriers with ease.
- The studied molecules are found to be non-inhibitors of Renal Organic Cation Transporter (ROCT) and also shown to be substrates and inhibitors of P-glycoprotein, signifying the distribution ability of the drugs^52^.
- Due to the role of cytochrome P450 (CYP450) in Phase I drug metabolism, it is considered the main parameter to examine ADME of the drugs^54,55^. From Table 9, all molecules are shown to be non-substrates and non-inhibitors of CYP450.
- None of the molecules were found to show toxicity risks, such as carcinogenic, mutagenic, etc.
- Not all compounds were damaging to AMES, which means they were not hazardous (Table 9).

**Table 9:** In silico toxicity predictions.

	[7]	[14]	Doxorubicin		[7]	[14]	Doxorubicin
H ERG inhibitor	Weak inhibitor	Weak inhibitor	No	Carcinogens	Non carcinogens	Non carcinogens	Carcinogens
AMES toxicity	Non toxic	Non toxic	Toxic	Rate acute toxicity	-3.0746	2.7148	2.66

However, many successful natural product-derived medicines including kinase inhibitors have LogP values exceeding 5. Compound [14]’s promising in silico binding profile, selectivity in cellular assays (high SI), and anticipated permeability (BBB + , HIA +) suggest biological activity despite its hydrophobicity. Hydrophobic CDKs may help pinpoint engagement in their hydrophobic ATP-binding pocket. To optimize LogP while conserving core pharmacophore and efficacy, future lead optimization could use strategic alkyl chain alteration (e.g., polar groups, unsaturation, or cyclization).

## Conclusions

Colorectal cancer (CRC) remains a significant global health challenge due to uncontrolled cell proliferation driven by dysregulated cyclin-dependent kinases (CDKs), particularly CDK-2 and CDK-1.

In this study, the fungal extract of *Aspergillus unguis* isolate SP51-EGY (“Sh cell”) demonstrated potent cytotoxic activity against HCT116 cells (IC_50_ = 3.49 µg/mL) with relative selectivity (SI = 23.33), highlighting its potential to target cancer cells while sparing normal cells.

Computational analyses identified compounds [7] and [14] as promising dual inhibitors of CDK2/cyclin E/A and CDK1/cyclin A/B, exhibiting strong binding affinities and stability in molecular dynamics simulations. These compounds are predicted to compete with ATP, which would induce G1/S and G2/M cell cycle arrest (a mechanism critical for halting tumor progression).

The integrated computational and cellular findings generate a strong hypothesis that compounds [7] and [14] act as dual CDK2/CDK1 inhibitors. This proposed mechanism remains to be confirmed and requires direct experimental validation through in vitro kinase inhibition assays, which will be a critical focus of subsequent work through in vitro kinase inhibition assays. Such biochemical studies are essential to confirm target engagement and will be a critical focus of our subsequent work.

**Fig. 5:**
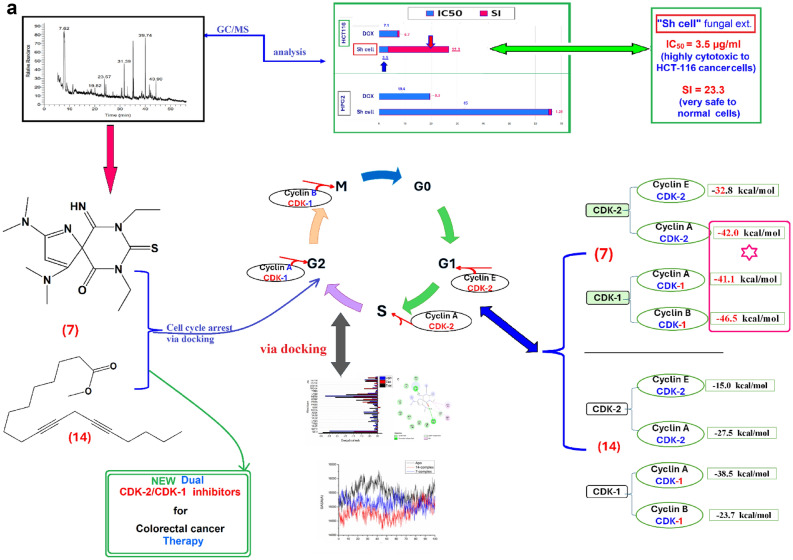

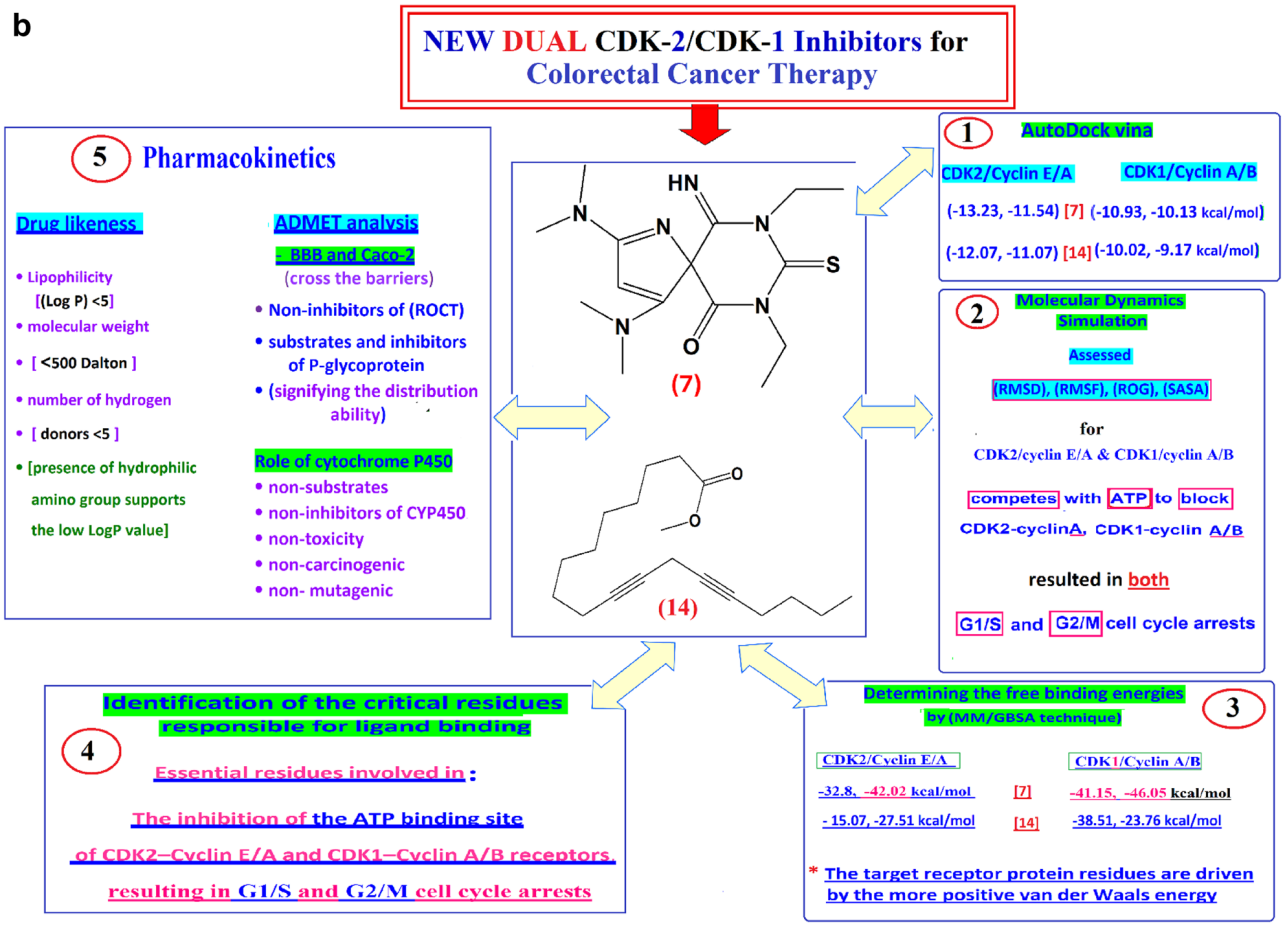
(**a**) A conclusive diagram showed that compound [7]* has been found to compete with ATP to block the CDK2 cyclin A, CDK1/cyclin A ,and CDK1/cyclin B complexes resulted in both G1/S and G2/M cell cycle arrests. (**b**) A conclusive diagram showed all the in Silico and Pharmacokinetic studies done for compounds [7] and [14].

Pharmacokinetic assessments confirmed drug-like properties, including favorable ADME profiles, compliance with Lipinski’s rule (except for LogP in [14]), and minimal toxicity risks. Notably, compound [7] displayed superior stability and binding energy compared to doxorubicin, which exhibited higher toxicity and lower selectivity. These integrated computational and cellular findings suggest that the fungal-derived compounds are promising candidates for novel dual CDK2/CDK1 inhibitors, warranting further experimental validation through biochemical kinase assays (Fig. 5a, b)^54,55^.

While previous studies have identified CDK-inhibitory activities in various natural products^9,26–49^, this work specifically highlights the potential of the previously unexplored *Aspergillus unguis* isolate SP51-EGY as a source of novel, structurally distinct leads with predicted dual CDK2/1 inhibitory activity and relative selectivity for colorectal cancer cells.

- These integrated computational and cellular findings generate a testable hypothesis that the putative compounds [7] and [14] may act as dual CDK2/CDK1 inhibitors. This proposed mechanism, based on tentative structural assignments, requires direct experimental validation through compound isolation, structural confirmation, and biochemical kinase inhibition assays.
- Further preclinical and clinical studies are warranted to optimize their efficacy and safety for future anticancer applications.

## Materials and methods

Chemicals, including Doxorubicin, DMEM, DMEM-F12, penicillin/streptomycin, trypsin solution, and fetal bovine serum, were procured from Lonza in Spain. MTT and N,O-bis(trimethylsilyl)trifluoroacetamide were obtained from Sigma-Aldrich. Pierce Biotechnology Inc. of Rockford, IL, supplied sodium acetate, Triton X-100, and p-nitrophenyl phosphate. All remaining reagents and substances were of analytical grade.

### Molecular identification of the fungal isolate

The fungus was identified as "Aspergillus unguis isolate SP51-EGY"^56,57^. The sequence has been deposited in GenBank with the name Aspergillus unguis isolate SP51-EGY and accession number KM203831.1.

### Screening medium and preparation of fungal extracts

Yeast Malt extract Broth medium (in static and shake) was selected for the cultivation of the isolated fungi. This medium exhibited the following ingredients (g/L): yeast extract (3), malt extract (17)^58^. Spore suspension (2%) with colony-forming units (10^6^ CFU) from each fungus was used to inoculate each culture medium, and the inoculated flasks (1L Erlenmeyer flasks each having 200 ml broth medium) were incubated at 30 °C under static and shaking (150 rpm) conditions for 12 days. The initial pH of all media was adjusted at 6.0.

Fungal cultures were prepared by cool centrifugation at 8000 rpm to separate fungal mycelia from the culture supernatant. The culture supernatant was extracted with the semi polar organic solvent ethyl acetate (3 × or till exhaustion), and then the solvent was evaporated under vacuum using *Heidolph* rotary evaporator. On the other hand, the fungal mycelia were first extracted using acetone (efficient in penetrating fungal cell walls), and the solvent was evaporated, and the residual water containing extract was re-extracted with ethyl acetate^59^. The obtained extracts were kept at 4 °C till use.

Secondary metabolite extracts from static and shake conditions were abbreviated as; Sh F (shake filtrate) extract, Sh Cell (shake mycelia) extract, St F (static filtrate) extract, and St Cell (static mycelia) extract.

### Cancer cell lines

The fungal extracts were evaluated for cytotoxicity against the human cancer cell lines HCT116 (colon), HepG2 (liver), and the RPE-1 human normal immortalized epithelial cell line. HCT116 cell cultures were maintained in DMEM, whereas the others were sustained in DMEM-F12 media supplemented with 10% fetal bovine serum, penicillin (100 U/mL), and streptomycin (2 mg/mL), incubated at 37 °C in 5% CO2 and 95% humidity. A 0.15% trypsin-versene solution was utilized for cell passage. Professor Stig Linder from the Oncology and Pathology department at the Karolinska Institute in Stockholm, Sweden, kindly supplied cell lines previously acquired from the American Type Culture Collection (ATCC).

### Cell proliferation assay

The cells were seeded into 96-well plates at a density of 20,000 cells per well for the HCT-116 and RPE-1 cell lines, and 10,000 cells per well for the HepG2 cell line. The media was aspirated 24 h post-seeding and substituted with serum-free culture media containing the extracts (100 g/mL). The cells underwent treatment in triplicate for a duration of 48 h. Doxorubicin (100 g/mL) served as the positive control, while dimethyl sulfoxide (DMSO) at 0.5% functioned as the negative control. The MTT [3-(4, 5-dimethylthiazol-2-yl)-2,5-diphenyltetrazolium bromide] assay was employed to assess cell survival following treatments^60^.

The cytotoxicity% % was determined using the following equation:

$$\% \, cytotoxicity \, = \left[ {1 - \left( {AVx \, /AVNC} \right)} \right] \times 100$$

where AVx denotes the average absorbance of the sample well and AVNC denotes the average absorbance of the negative control well measured at 595 nm with reference at 690 nm.

### Determination of IC_50_ values and selectivity index (SI)

Active extracts exhibiting cytotoxicity of 60% or greater on multiple cancer cell lines were selected for dose–response tests at varying concentrations. The final tested values, recorded in triplicate, were 100, 50, 25, 12.5, and 6.25 µg/mL, with a maximum of 0.78 µg/mL. GraphPad Prism® v6.0 (GraphPad Software Inc., San Diego, CA, USA) was utilized to derive the IC_50_ values by fitting the concentration–response curve to a nonlinear regression model of triplicate measurements at each concentration, and variability is reported as mean ± SD derived from the curve fitting.

The selectivity index (SI) is a value that indicates the selectivity (i.e., safety) of the fungal extract against cancer cells (HCT116 and HepG2) vs normal cells (RPE-1) and was calculated using the formula:

SI = IC_50_ of extract in RPE-1 normal cell line / IC_50_ of extract in the cancer cell line.

The greater the SI value, the safer the extract. Extracts with SI values larger than two are thought to have appropriate selectivity toward cancer cells^15^.

### GC/MS analysis

A dry extract (1.5 mg) was well combined with 20 μL of pyridine; thereafter, 30 μL of N,O-bis-(trimethylsilyl) trifluoroacetamide was added and heated at 80 °C for 30 min. GC/MS analysis was ultimately conducted^61,62^.

A Finnigan MAT SSQ 7000 mass spectrometer was connected to a Varian 3400 gas chromatograph. The DB-5 column possessed an internal diameter of 30 m × 0.32 mm; the carrier gas utilized was helium (He pressure, 20 MPa/cm^2^); the injector temperature was set at 310 °C; the gas chromatography temperature program ranged from 85 to 310 °C at a rate of 3 °C/minute with an initial hold of 10 min; and the electron impact mode operated at 70 eV. The scan repetition rate was 0.5 s across a mass range of 39 to 650 amu.

Identification relied on computational searches of user-constructed reference libraries (mainlib and Wiley9) containing mass spectra. Important Note on Compound Identification: GC/MS-based identifications, especially for novel or complex heterocycles such as compound [7], are provisional. The structures proposed here are based on spectral matching to reference libraries and should be considered putative until isolation and unambiguous structural elucidation (e.g., by NMR and HRMS) are performed. The subsequent in silico analyses (docking, dynamics, MM/GBSA) are therefore presented as a hypothesis-generating exercise to prioritize candidates for further experimental investigation, not as validation of the assigned structures or their mechanism. Peaks were confirmed for homogeneity using single-ion chromatographic reconstruction^62,63^.

#### In silico study

The identification of compounds was based on computational searches of reference mass spectral libraries (mainlib and Wiley9). It is important to note that for complex molecules, particularly novel or uncommon heterocycles like compound [7], GC/MS provides a tentative identification based on spectral matching. Definitive structural elucidation requires orthogonal techniques such as NMR spectroscopy and HRMS, which were not employed in this initial screening phase. Consequently, the structures presented, especially for [7], should be considered proposed identities pending future isolation and confirmatory analysis. The in silico studies conducted here—including molecular docking, dynamics simulations, and binding free energy calculations—are intended to prioritize leads and propose a plausible mechanism of action. They provide predictive insights that must be followed by biochemical validation.

### System preparation

The 3D structures of human Cyclin-dependent kinase 2-Cycline A (CDK2/CyclinA) (PDB code: 6gue)^64^, human Cyclin-dependent kinase 2-Cycline E (CDK2/Cyclin E)( PDB code: 1W98 )^65^, human Cyclin-dependent kinase 1-Cycline B (CDK1-cyclin B) (PDB code: 5LQF )^66^, and human Cyclin-dependent kinase 1-Cycline A (CDK1-cyclinA) (PDB code: 4Y72)^67^. Moreover, prepared using UCSF Chimera^68^. Using PROPKA, pH was fixed and optimized to 7.5^69^. The extracted 2D structure was drawn using ChemBioDraw Ultra 12.1^70^. The steepest descent approach and MMFF94 force field in Avogadro software^71^ were used to optimise the 2D structure for energy minimization. In preparation for docking, hydrogen atoms were removed using UCSF Chimera^68^.

### Docking protocol validation

To validate the accuracy and reliability of our molecular docking protocol using AutoDock Vina, we performed the following validation procedures:

1. Redocking and RMSD calculation for ligand-bound structures: For target complexes with co-crystallized ligands, we performed a redocking validation. The native ligand was extracted from each protein complex (CDK2/Cyclin A (PDB: 6GUE), CDK1/Cyclin A (PDB: 4Y72), and CDK1/Cyclin B (PDB: 5LQF)), then re-docked into its original binding site. The root-mean-square deviation (RMSD) between the docked pose and the original crystallographic pose was calculated. A successful validation is typically indicated by a heavy-atom RMSD of ≤ 2.0 Å, confirming the protocol’s ability to reproduce known binding modes. The calculated RMSD values for the redocked ligands were as follows: [0.40 Å for 6GUE, 1.32 Å for 4Y72, and 0.89 Å for 5LQF].
2. Validation for Apo structure (CDK2/Cyclin E—PDB: 1W98): As the 1W98 structure is in an apo (unliganded) state, redocking of a native ligand was not applicable. To ensure the docking grid for this target was appropriately centered on the canonical ATP-binding site, we cross-validated the binding pocket location by:
  Aligning the 1W98 structure with the ligand-bound CDK2 structure from 6GUE.
  Ensuring the defined docking grid encompassed the key catalytic residues (e.g., the DFG motif, hinge region) conserved across CDK2 structures.
  Docking a known CDK2 inhibitor to verify plausible pose generation in the expected site.
3. Control inhibitors: To further benchmark our docking scores and ensure they are within a plausible range, we included known CDK inhibitors as controls in our docking runs against all targets. Specifically, the CDK1/2 inhibitor Dinaciclib (SCH 727,965) and the CDK2 inhibitor AT-7519 were docked. The obtained docking scores for these reference compounds (e.g., between -10.0 and -12.0 kcal/mol) were consistent with values reported in similar computational studies and provided a relevant context for evaluating the scores of our candidate fungal compounds

### Molecular dynamics (MD) simulations

A production simulation of 100 ns was performed for each system. This timescale was selected as it is widely established in the literature for achieving stable protein–ligand conformational ensembles and for enabling subsequent MM/GBSA binding free energy calculations. Convergence to an equilibrium state was assessed by monitoring the stabilization of the backbone RMSD, RMSF, Rg, and SASA. The final 80 ns of each trajectory, where these metrics plateaued, was considered equilibrated and used for analysis. While longer simulations or independent replicates can provide additional statistical robustness, the consistent stabilization observed across all systems supports the reliability of the sampled conformational ensemble for the comparative analysis presented. The integration of Molecular dynamics (MD) simulations in biological systems’ study enables exploring the physical motion of atoms and molecules that cannot be easily accessed by any other means^72^. The insight extracted from performing this simulation provides an intricate perspective into the biological systems’ dynamical evolution, such as conformational changes and molecule associatio^72^. The MD simulations of all systems were performed using the GPU version of the PMEMD engine present in the AMBER 18 package^73^.

The partial atomic charge of each compound was calculated with ANTECHAMBER’s General Amber Force Field (GAFF) technique^74^. The Leap module of the AMBER 18 package implicitly solvated each system within an orthorhombic box of TIP3P water molecules within 10 Å of any box edge. The Leap module was used to neutralize each system by incorporating Na + and Cl- counter ions. A 2000-step initial minimization of each system was carried out in the presence of a 500 kcal/mol applied restraint potential, followed by a 1000-step full minimization using the conjugate gradient algorithm without restraints.

During the MD simulation, each system was gradually heated from 0 to 300 K over 500 ps, ensuring that all systems had the same number of atoms and volume. The system’s solutes were subjected to a 10 kcal/mol potential harmonic constraint and a 1 ps collision frequency. Following that, each system was heated and equilibrated for 500 ps at a constant temperature of 300 K.To simulate an isobaric-isothermal (NPT) ensemble, the number of atoms and pressure within each system for each production simulation were kept constant, with the system’s pressure maintained at 1 bar using the Berendsen barostat^75^.

For 100 ns, each system was MD-simulated. The SHAKE method was used to constrain the hydrogen bond atoms in each simulation. Each simulation used a 2 fs step size and integrated an SPFP precision model. An isobaric-isothermal ensemble (NPT) with randomised seeding, constant pressure of 1 bar, a pressure-coupling constant of 2 ps, a temperature of 300 K, and a Langevin thermostat with a collision frequency of 1 ps was used in the simulations.

The binding free energies (ΔG_bind) reported herein were calculated using the MM/GBSA method without the explicit entropy term (–TΔS). This is a standard approximation for high-throughput comparative analysis, as the computational cost of rigorous entropy calculation (e.g., via normal mode or quasi-harmonic analysis) is significant, and the relative ranking of ligands is often reliably reflected by the enthalpic component. We acknowledge that inclusion of entropic contributions could provide a more complete thermodynamic profile and is an important consideration for absolute binding free energy estimation.

### Post-MD analysis

After saving the trajectories obtained by MD simulations every 1 ps, the trajectories were analyzed using the AMBER18 suite’s CPPTRAJ^76^ module. The Origin^77^ data analysis program and Chimera. were used to create all graphs and visualizations.

### Thermodynamic calculation

The Poisson-Boltzmann or generalized Born and surface area continuum solvation (MM/PBSA and MM/GBSA) approach is helpful in the estimation of ligand-binding affinities^78^. The Protein–Ligand complex molecular simulations used by MM/GBSA and MM/PBSA compute rigorous statistical-mechanical binding free energy within a defined force field.

The binding free energy was averaged over 1000 snapshots extracted from the entire 100 ns trajectory. The estimation of the change in binding free energy (ΔG) for each molecular species (complex, ligand, and receptor) can be represented as follows^79^:

1 $$\Delta {\mathrm{G}}_{\mathrm{bind}}={\mathrm{G}}_{\mathrm{complex}}-{\mathrm{G}}_{\mathrm{receptor}}-{\mathrm{G}}_{\mathrm{ligand}}$$

2 $$\Delta {\mathrm{G}}_{\mathrm{bind}}={\mathrm{E}}_{\mathrm{gas}}+{\mathrm{G}}_{\mathrm{sol}}-\text{TS }$$

3 $${\mathrm{E}}_{\mathrm{gas}}={\mathrm{E}}_{\mathrm{int}}+{\mathrm{E}}_{\mathrm{vdw}}+{\mathrm{E}}_{\mathrm{ele}}$$

4 $${\mathrm{G}}_{\mathrm{sol}}={\mathrm{G}}_{\mathrm{GB}}+{\mathrm{G}}_{\mathrm{SA}}$$

5 $$G_{{SA}} = \gamma SASA$$

The terms Egas, Eint, Eele, and Evdw symbolize the gas-phase energy, internal energy, Coulomb energy, and van der Waals energy. The Egas was directly assessed from the FF14SB force field terms. Solvation-free energy (Gsol) was evaluated from the energy involved in the polar states (GGB) and non-polar states (G). The non-polar solvation free energy (GSA) was determined from the Solvent Accessible Surface Area (SASA)^80^ using a water probe radius of 1.4 Å. In contrast, solving the GB equation assessed the polar solvation (GGB) contribution. Items S and T symbolize the total entropy of the solute and temperature, respectively. The MM/GBSA-binding free energy method in Amber18 was used to calculate the contribution of each residue to the total binding free energy.

## Supplementary Information

Supplementary Information 1.

Supplementary Information 2.

Supplementary Information 3.

## Data Availability

The datasets used and/or analysed during the current study available from the corresponding author on reasonable request. **Correspondence and requests for materials should be addressed to:****Faten K. Abd EL-Hady** ; **email:** [fatenkamal@hotmail.com](mailto:fatenkamal@hotmail.com).

## References

[1] Bray, F. et al. Global cancer statistics 2022: GLOBOCAN estimates of incidence and mortality worldwide for 36 cancers in 185 countries. *CA. Cancer J. Clin.* **74**(3), 229–263 (2024). 38572751 10.3322/caac.21834

[2] Somu, P. & Paul, S. Supramolecular nano assembly of lysozyme and α-lactalbumin (apo α-LA) exhibits selective cytotoxicity and enhanced bioavailability of curcumin to cancer cells. *Colloids Surf. B Biointerfaces* **178**, 297–306. 10.1016/j.colsurfb.2019.03.016 (2019) (**Epub 2019 Mar 8. PMID: 30878804.**). 30878804 10.1016/j.colsurfb.2019.03.016

[3] IAR Cmarks Colorectal Cancer Awareness Month (2025).

[4] Sung, H. et al. Global cancer statistics 2020: GLOBOCAN estimates of incidence and mortality worldwide for 36 cancers in 185 countries. *CA. Cancer J. Clin.* **71**, 209–249 (2021). 33538338 10.3322/caac.21660

[5] Xi, Y. & Xu, P. Global colorectal cancer burden in 2020 and projections to 2040. *Transl Oncol*. 14(10), (2021). 10.1016/j.tranon.2021.101174. Epub Jul 6. PMID: 34243011; PMCID: PMC8273208. 10.1016/j.tranon.2021.101174 PMC8273208 34243011

[6] Mahadevappa, R. & Kwok, H. F. Phytochemicals - A novel and prominent source of anti-cancer drugs against colorectal cancer. *Comb. Chem. High Throughput Screen* **20**(5), 376–394. 10.2174/1386207320666170112141833 (2017) (**PMID: 28078982.**). 28078982 10.2174/1386207320666170112141833

[7] Blagosklonny, M. V. Selective protection of normal cells from chemotherapy, while killing drug-resistant cancer cells. *Oncotarget* **14**(1), 193–206. 10.18632/oncotarget.28382 (2023). 36913303 10.18632/oncotarget.28382 PMC10010629

[8] Pellarin, I., Dall’Acqua, A., Favero, A., Segatto, I., Rossi, V., Crestan, N., Karimbayli, J., Belletti, B., & Baldassarre, G. Cyclin-dependent protein kinases and cell cycle regulation in biology and disease. *Signal Transduct Target Ther* . 10, 11 (2025). 10.1038/s41392-024-02080-z PMC11734941 39800748

[9] Thoma, O.-M., Neurath, M. F. & Waldner, M. J. Cyclin-dependent kinase inhibitors and their therapeutic potential in colorectal cancer treatment. *Front. Pharmacol.* **12**, 757120. 10.3389/fphar.2021.757120 (2021). 35002699 10.3389/fphar.2021.757120 PMC8733931

[10] Ikwu, F. A., Isyaku, Y., Obadawo, B. S., Lawal, H. A. & Ajibowu, S. A. In silico design and molecular docking study of CDK2 inhibitors with potent cytotoxic activity against HCT116 colorectal cancer cell line. *J Genet EngBiotechnol*. 18(1), (2020). 10.1186/s43141-020-00066-2. PMID: 32930901; PMCID: PMC7492310. 10.1186/s43141-020-00066-2 PMC7492310 32930901

[11] Noori, R. et al. Microbial biofilm inhibition using magnetic cross-linked polyphenol oxidase aggregates. *ACS Appl. Bio Mater.* **7**(5), 3164–3178. 10.1021/acsabm.4c00175 (2024). 38722774 10.1021/acsabm.4c00175

[12] Bae, S. Y., Liao, L., Park, S. H., Kim, W. K., Shin, J., & Lee, S. K. Antitumor activity of Asperphenin A, a lipopeptidylbenzophenone from marine-derived Aspergillus sp. fungus, by inhibiting tubulin polymerization in colon cancer cells. *Marine drugs*. 18(2), 110 (2020). 10.3390/md18020110 PMC7073961 32069904

[13] Barzkar, N., Sukhikh, S. & Babich, O. Study of marine microorganism metabolites: New resources for bioactive natural products. *Front. Microbiol.* **14**, 1285902. 10.3389/fmicb.2023.1285902 (2024) (**PMID: 38260902; PMCID: PMC10800913.**). 38260902 10.3389/fmicb.2023.1285902 PMC10800913

[14] Tang, P. et al. Inhibitory effects and mechanism of the natural compound diaporthein B extracted from marine-derived fungi on colon cancer cells. *Molecules* **27**, 2944 (2022). 35566295 10.3390/molecules27092944 PMC9101636

[15] Nasr, T., Bondock, S., Youns, M., Fayad, W. & Zaghary, W. Synthesis, antitumor evaluation and microarray study of some new pyrazolo[3,4-d][1,2,3]triazine derivatives. *Eur. J. Med. Chem.* **141**, 603–614 (2017). 29107422 10.1016/j.ejmech.2017.10.016

[16] Zoete, V., Grosdidier, A. & Michielin, O. Docking, virtual high throughput screening and in silico fragment‐based drug design. *J. Cell. Mol. Med.* **13**(2), 238–248 (2009). 19183238 10.1111/j.1582-4934.2008.00665.x PMC3823351

[17] Liu, X., Ouyang, S., Yu, B., Liu, Y., Huang, K., Gong, J., Zheng, S., Li, Z., Li, H., & Jiang H. PharmMapper server: A Web server for potential drug target identification using pharmacophore mapping approach. *Nucleic. Acids Res*. 38,609- 614 (2010). [PMC free article] [PubMed]. 10.1093/nar/gkq300 PMC2896160 20430828

[18] Pharmmapper. Available online: http://lilab.ecust.edu.cn/pharmmapper/.

[19] Mirzaei, S., Eisvand, F., Hadizadeh, F., Mosaffa, F., Ghasemi, A., & Ghodsi, R. Design, synthesis and biological evaluation of novel 5,6,7-trimethoxy-N-aryl-2-styrylquinolin-4-amines as potential anticancer agents and tubulin polymerization inhibitors. *Bioorg. Chem*. 98,(2020). 10.1016/j.bioorg.2020.103711 32179282

[20] Hasanin, M., Hashem, A. H., El-Rashedy, A. A. & Kamel, S. Synthesis of novel heterocyclic compounds based on dialdehyde cellulose: Characterization, antimicrobial, antitumor activity, molecular dynamics simulation and target identification. *Cellulose* **28**, 8355–8374 (2021).

[21] Machaba, K. E., Mhlongo, N. N. & Soliman, M. E. S. Induced mutation proves a potential target for TB therapy: A molecular dynamics study on LprG. *Cell. Biochem. Biophys.* **76**, 345–356 (2018). 30073572 10.1007/s12013-018-0852-7

[22] Pan, L., Patterson, J. C., Deshpande, A., Cole, G. & Frautschy, S. Molecular dynamics study of Zn(Aβ) and Zn(Aβ)_2_. *PLoS ONE* **8**, 70681–70688 (2013). 10.1371/journal.pone.0070681 PMC3785486 24086248

[23] Wijffels, G., Dalrymple, B., Kongsuwan, K. & Dixon, N. Conservation of Eubacterialreplicases. *IUBMB Life* **57**, 413–419 (2005). 16012050 10.1080/15216540500138246

[24] Richmond, T. J. Solvent accessible surface area and excluded volume in proteins: Analytical equations for overlapping spheres and implications for the hydrophobic effect. *J. Mol. Biol.* **178**, 63–89 (1984). 6548264 10.1016/0022-2836(84)90231-6

[25] Cournia, Z., Allen, B., & Sherman, W. Relative Binding Free Energy Calculations in Drug Discovery: Recent Advances and Practical Considerations. *J. Chem. Inf. Model*. American Chemical Society (2017, December 26) (2017) . 10.1021/acs.jcim.7b00564 29243483

[26] Bailon-Moscoso, N., Cevallos-Solorzano, G., Romero-Benavides, J. C. & Orellana, M. I. Natural compounds as modulators of cell cycle arrest: Application for anticancer chemotherapies. *Curr. Genomics* **18**, 106–131 (2017). 28367072 10.2174/1389202917666160808125645 PMC5345333

[27] Pang, W., Li, Y., Guo, W. & Shen, H. Cyclin E: A potential treatment target to reverse cancer chemoresistance by regulating the cell cycle. *Am. J. Transl. Res.* **12**(9), 5170–5187 (2020). 33042412 PMC7540110

[28] Bahnassy, A.A., Zekri, AR.N. & El-Houssini, S. Cyclin A and cyclin D1 as significant prognostic markers in colorectal cancer patients. *BMC Gastroenterol* . 4, 22 (2004). 10.1186/1471-230X-4-22 10.1186/1471-230X-4-22 PMC524166 15385053

[29] Zaki, W. A. et al. Design, synthesis, in vitro, and in silico studies of new N5-Substituted-pyrazolo[3,4-d]pyrimidinone derivatives as anticancer CDK2 inhibitors. *Pharmaceuticals* **16**(11), 1593 (2023). 38004458 10.3390/ph16111593 PMC10674233

[30] Shen, J. et al. Identification and validation of CDK1 as a promising therapeutic target for Eriocitrin in colorectal cancer: A combined bioinformatics and experimental approach. *BMC Cancer* **25**, 76 (2025). 39806333 10.1186/s12885-025-13448-x PMC11731355

[31] Wong Chin, J. M., Jeewon, R., Fahad, A., A., Puchooa, D., Bahorun, T., & Neergheen, V. S. Marine-derived fungi from the genus *Aspergillus* (Ascomycota) and their anticancer properties. *Mycology*. 1–48 (2024). 10.1080/21501203.2024.2402309 PMC12096698 40415918

[32] Dunbar, K. L., Perlatti, B., Liu, N., Cornelius, *et al.* Resistance gene-guided genome mining reveals the roseopurpurins as inhibitors of cyclin-dependent kinases. *Proc Natl Acad Sci U S A*. 120 (48), e2310522120 (2023). 10.1073/pnas.2310522120.. 10.1073/pnas.2310522120 PMC10691236 37983497

[33] Zhang, G. Y. et al. Four sulfur-containing compounds with anti-colon cancer effect from marine-derived fungus *Aspergillus terreus*. *Fitoterapia* **175**, 105967 (2024). 38631597 10.1016/j.fitote.2024.105967

[34] Konishi, H., Isozaki, S. & Kashima, S. Probiotic *Aspergillus oryzae* produces anti-tumor mediator and exerts anti-tumor effects in pancreatic cancer through the p38 MAPK signaling pathway. *Sci Rep.* **11**, 11070 (2021). 34040123 10.1038/s41598-021-90707-4 PMC8154913

[35] Moussa, A. Y., Mostafa, N. M. & Singab, A. N. B. Pulchranin A: First report of isolation from an endophytic fungus and its inhibitory activity on cyclin dependent kinases. *Nat. Prod. Res.* **34**(19), 2715–2722. 10.1080/14786419.2019.1585846 (2019). 30887847 10.1080/14786419.2019.1585846

[36] Wu, G. S. et al. Ganoderic acid DM, a natural triterpenoid, induces DNA damage, G1 cell cycle arrest and apoptosis in human breast cancer cells. *Fitoterapia* **83**(2), 408–414. 10.1016/j.fitote.2011.12.004 (2012). 22178684 10.1016/j.fitote.2011.12.004

[37] Bailon-Moscoso, N., Cevallos-Solorzano, G., Romero-Benavides, J. C. & Orellana, M. I. Natural compounds as modulators of cell cycle arrest: Application for anticancer chemotherapies. *Curr. Genomics* **18**(2), 106–131. 10.2174/1389202917666160808125645 (2017). 28367072 10.2174/1389202917666160808125645 PMC5345333

[38] Shah, U., Shah, R., Acharya, S. & Acharya, N. Novel anticancer agents from plant sources. *Chin. J. Nat. Med.* **11**, 16–23 (2013).

[39] Abdelbagi, M. E. M. et al. Exploring Securigera securidaca Seeds as a Source of Potential CDK1 Inhibitors: Identification of Hippeastrine and Naringenin as Promising Hit Candidates. *Processes.* **11**, 1478. 10.3390/pr11051478 (2023).

[40] Chan, K. C. et al. Polyphenol-rich extract from mulberry leaf inhibits vascular smooth muscle cell proliferation involving upregulation of p53 and inhibition of cyclin-dependent kinase. *J. Agric. Food Chem.* **58**(4), 2536–2542 (2010). 20070102 10.1021/jf904293p

[41] Aggarwal, B. B. & Ichikawa, H. Molecular targets and anticancer potential of indole-3-carbinol and its derivatives. *Cell Cycle* **4**(9), 1201–1215 (2005). 16082211 10.4161/cc.4.9.1993

[42] Hogan, F. S., Krishnegowda, N. K., Mikhailova, M. & Kahlenberg, M. S. Flavonoid, silibinin, inhibits proliferation and promotes cellcycle arrest of human colon cancer. *J. Surg. Res.* **143**(1), 58–65 (2007). 17950073 10.1016/j.jss.2007.03.080

[43] Li, C. Y. et al. Molecular mechanisms of Lycorisaurea agglutinin-induced apoptosis and G2/ M cell cycle arrest in human lung adenocarcinoma A549 cells, both in vitro and in vivo. *Cell Prolif.* **46**(3), 272–282 (2013). 23692086 10.1111/cpr.12034 PMC6495545

[44] Liu, B. et al. Acetylbritannilactone induces G1 arrest and apoptosis in vascular smooth muscle cells. *Int. J. Cardiol.* **149**(1), 30–38 (2011). 20060605 10.1016/j.ijcard.2009.11.036

[45] Takagaki, N. et al. Apigenin induces cell cycle arrest and p21/WAF1 expres sion in a p53-independent pathway. *Int. J. Oncol.* **26**, 185–189 (2005). 15586239

[46] Shukla, S. & Gupta, S. Apigenin-induced cell cycle arrest is mediated by modulation of MAPK, PI3K-Akt, and loss of cyclin D1 associated retinoblastoma dephosphorylation in human prostate cancer cells. *Cell Cycle* **6**, 1102–1114 (2007). 17457054 10.4161/cc.6.9.4146

[47] Chiang, L. C., Ng, L. T., Lin, I. C., Kuo, P. L. & Lin, C. C. Antiproliferative effect of apigenin and its apoptotic induction in human Hep G2 cells. *Cancer Lett.* **237**, 207–214 (2006). 16023288 10.1016/j.canlet.2005.06.002

[48] Daina, A., Michielin, O. & Zoete, V. SwissADME: A free web tool to evaluate pharmacokinetics, drug-likeness and medicinal chemistry friendliness of small molecules. *Sci. Rep.* **717**, 1–13 (2017). 10.1038/srep42717 PMC5335600 28256516

[49] Cheng, F., Li, W. & Zhou, Y. AdmetSAR: A comprehensive source and free tool for assessment of chemical ADMET properties. *J. Chem. Inf. Model.* **52**(11), 3099–3105. 10.1021/ci300367a (2012). 23092397 10.1021/ci300367a

[50] Lipinski, C. A. Lead-and drug-like compounds: the rule-of-five revolution. *Drug Discov. Today Technol.* **1**, 337–341 (2004). 24981612 10.1016/j.ddtec.2004.11.007

[51] Lipinski, C. A. Rule of five in 2015 and beyond: Target and ligand structural limitations, ligand chemistry structure and drug discovery project decisions. *Adv. Drug Deliv. Rev.* **101**, 34–41 (2016). 27154268 10.1016/j.addr.2016.04.029

[52] Joshi, M. et al. Spanbert: Improving pre-training by representing and predicting spans. *Trans. Assoc. Comput. Linguist.* **8**, 64–77 (2020).

[53] Singh, J., & Singh, J. COVID-19 and its impact on society. *Electronic Research Journal of Social Sciences and Humanities*. 2(1), (2020).‏ Available at SSRN: https://ssrn.com/abstract=3567837.

[54] Paramashivam, S. K. et al. In silico pharmacokinetic and molecular docking studies of small molecules derived from *Indigofera aspalathoides* Vahl targeting receptor tyrosine kinases. *Bioinformation* **11**, 73–84 (2015). 25848167 10.6026/97320630011073 PMC4369682

[55] Malumbres, M. & Barbacid, M. Cell cycle, CDKs and cancer: A changing paradigm. *Nat. Rev. Cancer.* **9**, 153–166 (2009). 19238148 10.1038/nrc2602

[56] Aboutabl, M. E., Maklad, Y. A., Abdel-Aziz, M. S. & El-Hady, F. K. A. In vitro and in vivo studies of the antidiabetic potential of Red Sea sponge-associated fungus “*Aspergillus unguis*” isolate SP51-EGY with correlations to its chemical composition. *J. Appl. Pharm. Sci.* **12**(8), 165–178 (2022).

[57] Nasr, S., Dawood, A. S., Ibrahim, A. M., Abdel-Aziz, M. S., Fayad, W., Abdelnaser, A. & El-Hady, F. K. A. Anti-inflammatory potential of aspergillus unguis SP51-EGY: TLR4-dependent effects & chemical diversity via Q-TOF LC-HRMS. *BMC Biotechnol.* 24–62 (2024) 10.1186/s12896-024-00890-1 PMC11411751 39294631

[58] Kang, M. G., Yi, S. H. & Lee, J. S. Production and characterization of a new α-Glucosidase inhibitory peptide from *Aspergillus oryzae* N159-1. *Mycobiology* **41**(3), 149–154 (2013). 24198670 10.5941/MYCO.2013.41.3.149 PMC3817230

[59] Abd El-Hady, F. K., Abdel-Aziz, M. S., Shaker, K. H., El-Shahid, Z. A. & Ghani, M. A. Coral-derived fungi inhibit acetyl cholinesterase, superoxide anion radical, and microbial activities. *Int. J. Pharm. Sci. Rev. Res.* **26**(1), 301–308 (2014).

[60] El-Menshawi, B. S. et al. Screening of natural products for therapeutic activity against solid tumors. *Indian J. Exp. Biol.* **48**, 258–264 (2010). 21046978

[61] Salah, N. M., Mettwally, W. S. A., Afifi, A. H., Kamel, R. & Abd El-Hady, F. K. A. Anti-candida effect of Saudi nanoencapsulation. *Egypt. J. Chem.* **66**, 25–39 (2023).

[62] Abdelsalam, E. et al. Combating COVID-19 and its co-infection by *Aspergillus tamarii* SP73-EGY using in vitro and in silico studies. *Sci. Rep.*10.1038/s41598-024-77854-0 (2025). 39753574 10.1038/s41598-024-77854-0 PMC11698736

[63] Abd El-Hady, F. K. A. et al. Comparative correlation between chemical composition and cytotoxic potential of the coral-associated fungus Aspergillus sp. 2C1-EGY against human colon cancer cells. *Current microbiol.* **74**, 1294–1300 (2016). 10.1007/s00284-017-1316-9 28752341

[64] Wood, D. J. et al. Differences in the conformational energy landscape of CDK1 and CDK2 suggest a mechanism for achieving selective CDK inhibition. *Cell Chem. Biol.* **26**, 121–130 (2019). 30472117 10.1016/j.chembiol.2018.10.015 PMC6344228

[65] Honda, R. et al. The structure of cyclin E1/CDK2: Implications for CDK2 activation and CDK2-independent roles. *EMBO J.* **24**, 452–463 (2005). 15660127 10.1038/sj.emboj.7600554 PMC548659

[66] Coxon, C. R. et al. Cyclin-dependent kinase (CDK) inhibitors: Structure-activity relationships and insights into the CDK-2 selectivity of 6-substituted 2-arylaminopurines. *J. Med. Chem.* **60**, 1746–1767 (2017). 28005359 10.1021/acs.jmedchem.6b01254 PMC6111440

[67] Brown, N. R. et al. CDK1 structures reveal conserved and unique features of the essential cell cycle CDK. *Nat. Commun.* **616**, 1–12 (2015). 10.1038/ncomms7769 PMC4413027 25864384

[68] Pettersen, E. F. et al. UCSF Chimera--A visualization system for exploratory research and analysis. *J. Comput. Chem.* **25**, 1605–1612 (2004). 15264254 10.1002/jcc.20084

[69] Li, H., Robertson, A. D. & Jensen, J. H. Very fast empirical prediction and rationalization of protein pKa values. *Proteins* **61**, 704–721 (2005). 16231289 10.1002/prot.20660

[70] Halford, B. Reflections on ChemDraw. *Chem. Eng. News Arch.* **92**, 26–27 (2014).

[71] Hanwell, M. D. et al. Avogadro: An advanced semantic chemical editor, visualization, and analysis platform. *J. Cheminform.* **4**, 17 (2012). 22889332 10.1186/1758-2946-4-17 PMC3542060

[72] Hospital, A., Goñi, J. R., Orozco, M. & Gelpí, J. L. Molecular dynamics simulations: Advances and applications. *Adv. Appl. Bioinform. Chem.* **8**, 37–47 (2015). 26604800 10.2147/AABC.S70333 PMC4655909

[73] Lee, T. S. et al. GPU-accelerated molecular dynamics and free energy methods in Amber18: Performance enhancements and new features. *J. Chem. Inf. Model.* **58**, 2043–2050 (2018). 30199633 10.1021/acs.jcim.8b00462 PMC6226240

[74] Wang, J., Wang, W., Kollman, P. A. & Case, D. A. Automatic atom type and bond type perception in molecular mechanical calculations. *J. Mol. Graph. Model.* **25**, 247–260 (2006). 16458552 10.1016/j.jmgm.2005.12.005

[75] Berendsen, H. J. C., Postma, J. P. M., van Gunsteren, W. F., DiNola, A. & Haak, J. R. Molecular dynamics with coupling to an external bath. *J. Chem. Phys.* **81**, 3684–3690 (1984).

[76] Roe, D. R. & Cheatham, T. E. PTRAJ and CPPTRAJ: Software for processing and analysis of molecular dynamics trajectory data. *J. Chem. Theory Comput.* **9**, 3084–3095 (2013). 26583988 10.1021/ct400341p

[77] Seifert, E. OriginPro 9.1: Scientific data analysis and graphing software - software review. *J. Chem. Inf. Model.* **54**, 1552–1552 (2014). 24702057 10.1021/ci500161d

[78] Hayes , M., J., & Archontis, G. MM-GB(PB)SA calculations of protein-ligand binding free energies, in Molecular Dynamics - Studies of Synthetic and Biological Macromolecules. *InTech* , (2012).

[79] Hou, T., Wang, J., Li, Y. & Wang, W. Assessing the performance of the MM/PBSA and MM/GBSA methods. 1. The accuracy of binding free energy calculations based on molecular dynamics simulations. *J. Chem. Inf. Model.* **51**, 69–82 (2011). 21117705 10.1021/ci100275a PMC3029230

[80] Sitkoff, D., Sharp, K. A. & Honig, B. Accurate calculation of hydration free energies using macroscopic solvent models. *J. Phys. Chem.* **98**, 1978–1988 (1994).

