# Supplementary material for: New predicted dual CDK-2/CDK-1 inhibitors from Aspergillus unguis isolate SP51-EGY with relative selectivity for colorectal cancer cells: a computational and experimental approach
Source: Sci Rep. 2026 Apr 11;16:12181. doi: 10.1038/s41598-026-41120-2 (PMC13076678; doi:10.1038/s41598-026-41120-2)
Supplement: Supplementary file 1 — Supplementary Information 1. [file 41598_2026_41120_MOESM1_ESM.pdf]

Library Search Report

NRC-GC/EI-MS Lab

		Data File:	Faten-212
Original Data Path:	C:\XCALIBUR\DATA\SAMEH\GC-MS\2018\5	Current Data Path:	C:\Xcalibur\data\sameh\GC-MS\2018\5
Sample Type:	Unknown	Sample ID:	1
Sample Name:		Operator:	ISQ120602
Acquisition Date:	10/08/18 11:34:10 AM	Run Time(min):	85.00
Comments:		Vial:	1
Scans:	24996	Low Mass(m/z):	40.00000
High Mass(m/z):	800.03113	Sample Weight:	0.00
ISTD Amount:	0.000	Calibration Level:	
Dilution Factor:	1.00		

Instrument Method: C:\Xcalibur\methods\sameh\dr_faten.meth

Original Processing Method:

Current Processing Method: C:\Xcalibur\methods\sameh\Faten-150

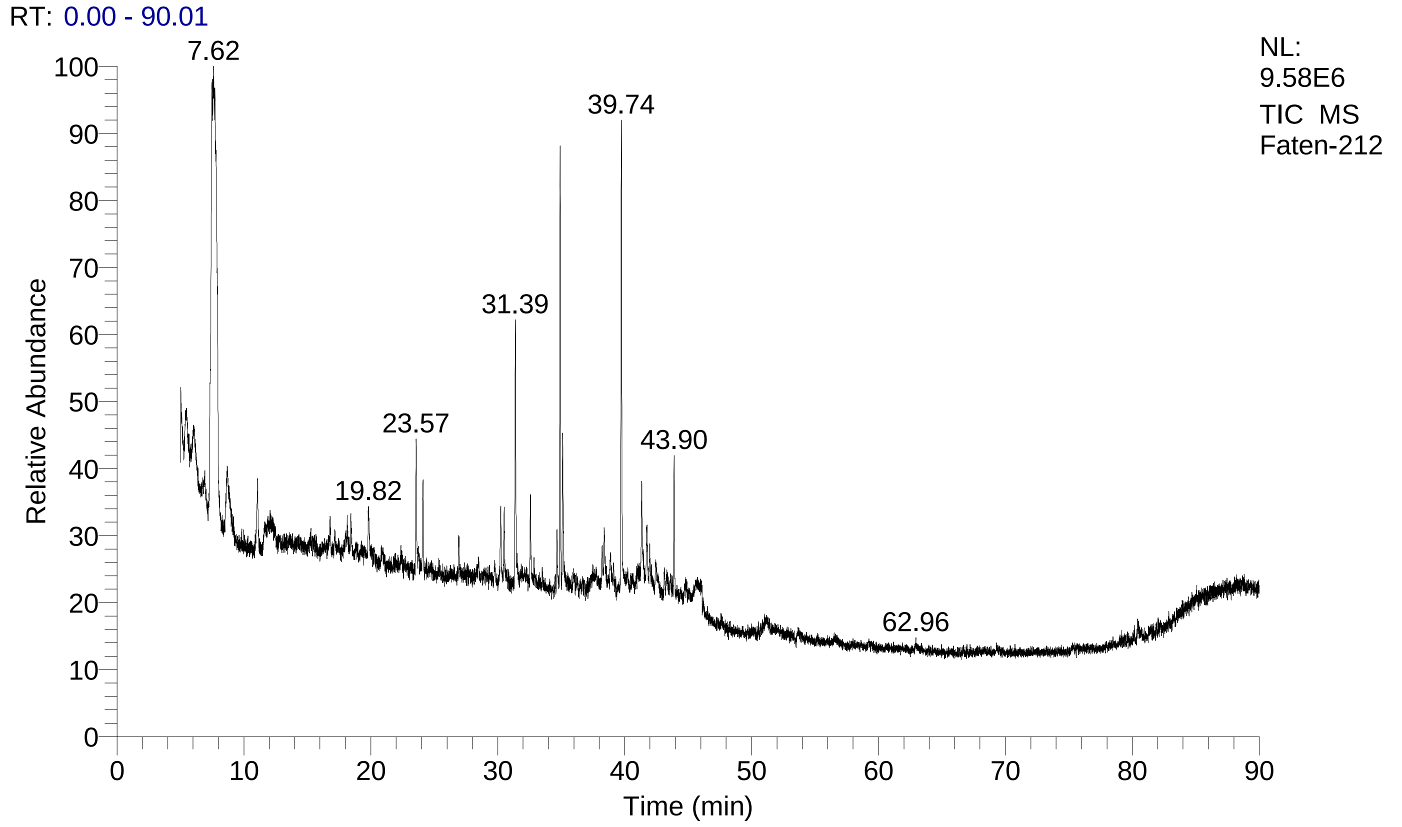

RT	Area %	Peak Area	Peak Height
5.03	0.61	1925499.43	720005.99
5.42	0.85	2703802.54	452242.90
5.48	0.57	1822562.63	438228.82
6.05	0.77	2459672.90	407419.66
6.09	0.32	1026114.65	406376.68
6.77	0.18	558797.44	135829.92
6.93	0.66	2108596.78	320496.51
7.33	0.28	895707.58	340570.38
7.50	7.82	24838879.42	4001928.56
7.62	10.15	32220813.98	4426330.37
7.71	7.12	22618652.74	4392044.09
8.69	2.32	7354905.43	659254.73
9.83	0.16	517700.02	167986.40
10.06	0.14	435252.33	144258.40
11.08	1.55	4920818.77	743719.08
11.64	0.50	1601053.00	192094.43
11.87	0.17	531428.00	117716.90

There is no signature data to report.

Library Search Report

RT	Area %	Peak Area	Peak Height
12.10	0.18	570728.42	151553.09
12.27	0.32	1009979.88	130189.53
12.49	0.16	504137.08	122516.14
12.84	0.23	719768.45	124394.84
13.63	0.19	594278.18	83420.72
15.22	0.17	534503.25	154172.93
15.57	0.26	824685.55	129825.91
16.79	0.90	2844860.84	429811.38
17.15	0.32	1024227.87	228182.39
17.91	0.19	597830.44	106767.82
18.03	0.37	1168927.66	222543.76
18.15	0.52	1640338.22	353605.23
18.43	0.86	2726093.57	462582.33
18.84	0.36	1132990.22	147115.26
18.93	0.13	421470.06	112814.13
19.26	0.17	534594.69	112601.88
19.82	0.99	3156981.79	594129.88
20.27	0.25	780831.26	176699.52
20.87	0.37	1178136.59	183642.78
20.96	0.22	706019.11	85043.89
21.50	0.13	428552.38	90712.11
22.38	0.35	1126679.55	227396.90
23.16	0.19	608833.83	110524.47
23.57	2.31	7334832.83	1700321.76
23.76	0.26	811517.49	216018.35
24.11	1.80	5721645.07	1167037.61
24.37	0.22	703282.39	147348.84
24.60	0.13	415483.86	86645.94
24.73	0.20	634161.36	103738.38
25.36	0.17	547913.23	170601.55
25.39	0.17	551581.58	109748.39
26.93	0.58	1843015.42	506653.17
26.98	0.14	443747.78	103543.69
27.40	0.14	456331.08	113062.42
27.61	0.21	675951.28	157623.41
27.80	0.16	519636.18	86153.71
28.39	0.23	733172.42	124308.95
28.49	0.48	1514889.24	275201.21
28.75	0.15	486563.25	112127.34
28.88	0.18	585438.82	107697.17
28.98	0.16	513527.98	129740.79
29.08	0.20	638965.57	135107.44
29.35	0.15	478532.45	140647.36
29.76	0.42	1336240.15	239995.50
30.24	1.33	4214125.25	894023.44
30.51	1.19	3792960.04	876313.10
31.39	4.24	13450336.03	3313927.04
31.78	0.15	473178.36	108529.94
31.86	0.17	528511.93	160464.31
32.12	0.14	457604.61	113975.45
32.27	0.32	1004912.30	155138.42
32.58	1.51	4786416.64	1138627.10
32.86	0.14	441735.06	135088.32
33.51	0.29	916758.17	222138.34
34.35	0.15	483966.48	89315.24
34.54	0.20	641881.22	156704.83
34.67	0.95	3009196.19	716023.97
34.92	6.99	22207131.85	5743117.57
35.12	2.63	8352797.36	1849094.81
35.96	0.54	1713825.63	226907.72
36.22	0.13	425012.15	143235.60
36.30	0.21	674855.80	135341.87
36.48	0.14	451042.43	91367.91

There is no signature data to report.

Library Search Report

RT	Area %	Peak Area	Peak Height
36.67	0.20	644617.83	143641.82
36.77	0.16	522606.05	106542.80
37.15	0.13	405047.80	121164.33
37.34	0.20	634692.82	111604.44
37.51	0.21	652926.52	128956.31
37.75	0.20	639754.56	117098.86
38.23	0.53	1686674.58	427324.06
38.39	0.75	2388254.24	553775.60
38.88	0.72	2278081.58	402636.58
39.12	0.31	984420.76	247926.92
39.53	0.15	469862.96	75643.40
39.74	7.74	24573453.27	5984017.57
40.24	0.28	896748.08	174932.75
40.49	0.16	497551.67	124421.72
40.60	0.15	469134.89	132500.68
40.94	0.18	574795.82	123713.00
41.03	0.28	878542.98	161037.73
41.34	2.00	6340349.45	1197366.50
41.75	1.16	3689071.10	657656.15
41.98	0.64	2023222.66	421115.67
42.45	0.61	1949300.89	321641.07
43.13	0.43	1357074.90	312895.51
43.32	0.50	1596754.63	232994.84
43.64	0.40	1259776.60	218386.39
43.90	2.22	7060952.45	1764634.07
44.41	0.23	732876.13	114240.95
44.79	0.24	763196.84	191563.81
44.85	0.20	634853.92	189523.76
45.06	0.13	408709.49	74013.99
45.58	0.16	506885.27	130571.37
45.73	0.19	608028.96	95437.12
45.99	0.28	898232.01	187038.62
46.05	0.27	868960.82	247669.26
46.22	0.21	676866.36	121291.23
46.53	0.13	417450.47	99090.99
47.59	0.13	411307.01	120070.91
48.27	0.13	403920.72	119211.85
50.15	0.16	495232.94	83738.76
50.24	0.16	505098.79	96958.82
51.08	0.17	547049.20	130377.83
51.19	0.29	914952.37	150511.97
51.26	0.30	956020.71	118778.57
53.10	0.13	418264.90	70948.99
53.59	0.16	495720.29	101193.77
55.16	0.14	437538.93	85602.03
56.50	0.13	417210.87	77504.80
59.25	0.14	432444.98	74972.30
62.95	0.21	677632.11	97419.03
64.42	0.13	413671.87	54029.23
67.94	0.13	400555.23	69884.87
79.44	0.16	522078.03	95022.32
79.62	0.16	511915.05	124823.21
80.43	0.25	788606.97	196431.51
80.50	0.14	442753.53	114799.91
81.37	0.14	433851.46	85131.34
81.91	0.19	606472.79	122696.63
82.21	0.16	499798.94	115641.29
82.88	0.15	473026.84	121746.99
83.31	0.15	474520.07	118967.15
83.47	0.13	411201.87	120639.13
84.43	0.15	463104.06	124597.81
84.77	0.16	503266.16	102675.37
85.77	0.13	421419.64	93639.80

There is no signature data to report.

Library Search Report

RT	Area %	Peak Area	Peak Height
87.46	0.15	484867.97	142326.66
87.68	0.14	454109.06	80418.47
88.69	0.16	497594.00	133980.63
88.82	0.18	580591.14	114463.07
89.34	0.20	628546.27	103553.74
89.74	0.18	577882.00	95073.82
89.94	0.15	478353.71	122729.99

There is no signature data to report.

Library Search Report

RT	Probability	Compound Name	SI	Area %	Area	Molecular Weight	Molecular Formula	Library
5.03	17.85	Cathine (CAS)	483	0.61	1925499.43	151	C9H13NO	Wiley9
5.03	11.88	Pregnane-3,11,20,21-tetrol, cyclic 20,21-(butyl boronate), (3à,5á,11á,20R)- (CAS)	471	0.61	1925499.43	418	C25H43BO4	Wiley9
5.03	6.13	d-Galactitol, 1-deoxy-, pentaacetate (CAS)	453	0.61	1925499.43	376	C16H24O10	Wiley9

Faten-212 #10 RT: 5.03 AV: 1 RF: 6.00, 3 NL: 1.90E5
F: {0,0} + c EI Full ms [40.00-800.00]

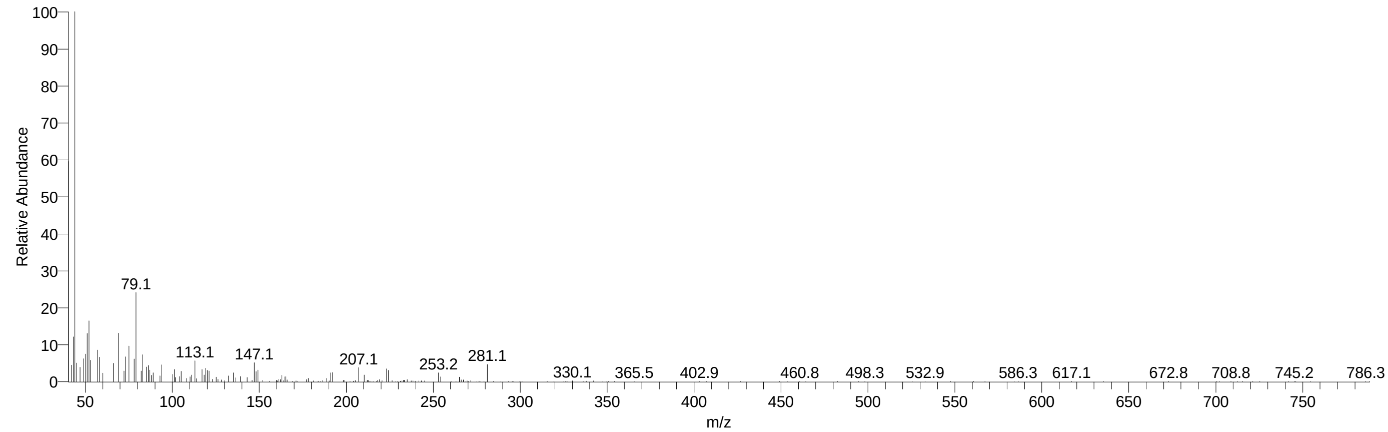

Hit Spectrum	Delta	Compound Structure

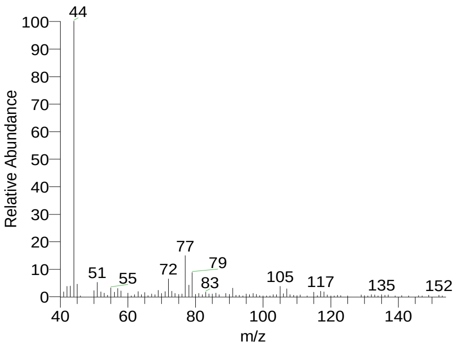

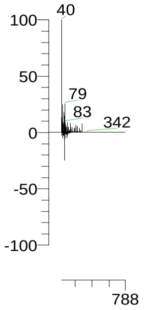

Cathine (CAS)
Formula C9H13NO, MW 151, CAS# 492-39-7, Entry# 52366
Benzenemethanol, à-(1-aminoethyl)-, [S-(R@,R@)]-

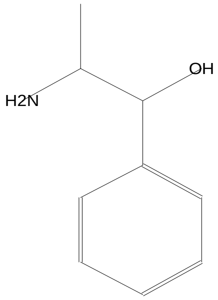

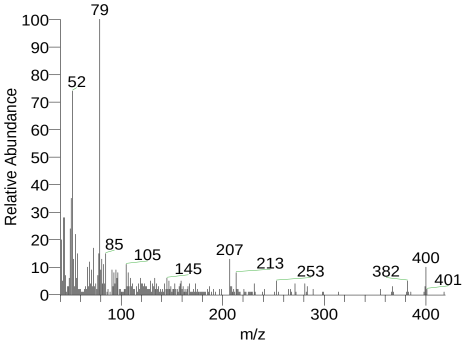

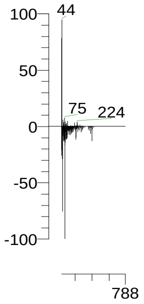

Pregnane-3,11,20,21-tetrol, cyclic 20,21-(butyl boronate), (3à,5á,11á,20R)- (CAS)
Formula C25H43BO4, MW 418, CAS# 55556-74-6, Entry# 562492
5á-PREGNAN-3à,11á,20à,21-TETROL BUTYL BORONATE

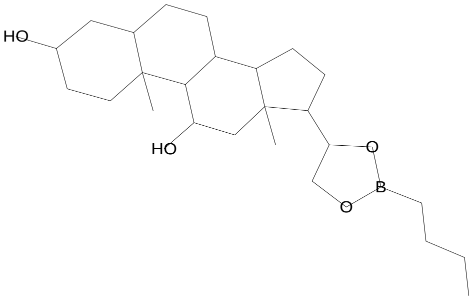

There is no signature data to report.

Library Search Report

Hit Spectrum	Delta	Compound Structure

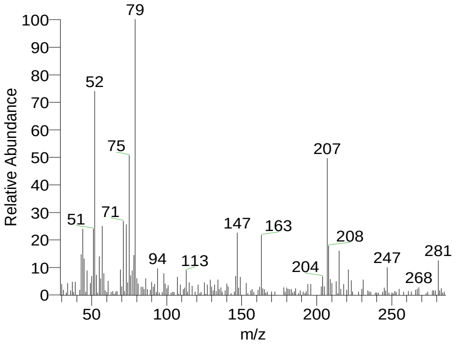

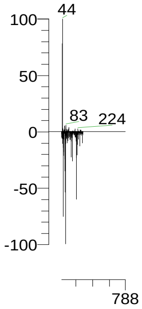

d-Galactitol, 1-deoxy-, pentaacetate (CAS)
Formula C16H24O10, MW 376, CAS# 7226-60-0, Entry# 511381
L-FUCITOL-1,2,3,4,5-PENTAACETATE

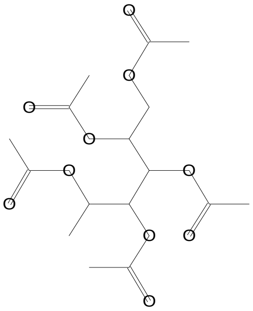

There is no signature data to report.

Library Search Report

RT	Probability	Compound Name	SI	Area %	Area	Molecular Weight	Molecular Formula	Library
5.42	38.77	3-Cyano-2H-benzo[f]chromene	689	0.85	2703802.54	207	C14H9NO	Wiley9
5.42	27.37	Ethyl 3-Butenyl Phosphite	679	0.85	2703802.54	316	C18H21O3P	Wiley9
5.42	19.32	Ethyl 2-Cyano-2-(p-tolyl)-2-phenylacetate	669	0.85	2703802.54	279	C18H17NO2	Wiley9

Faten-212 #123 RT: 5.42 AV: 1 RF: 6.00, 3 NL: 8.44E4
F: {0,0} + c EI Full ms [40.00-800.00]

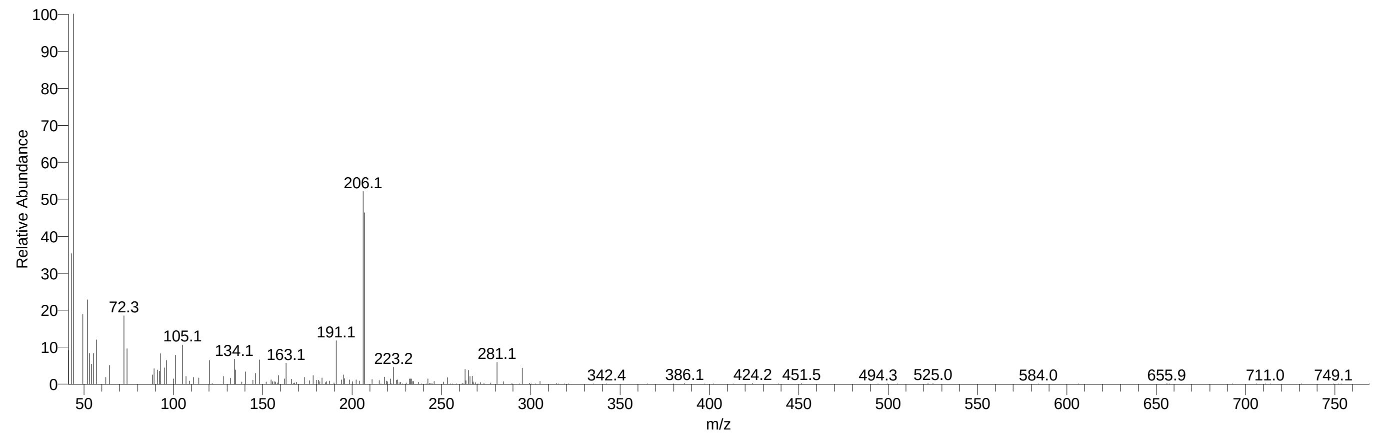

Hit Spectrum	Delta	Compound Structure

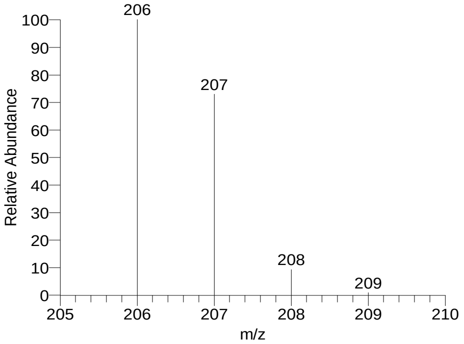

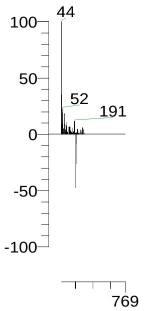

3-Cyano-2H-benzo[f]chromene
Formula C14H9NO, MW 207, CAS# NA, Entry# 158884

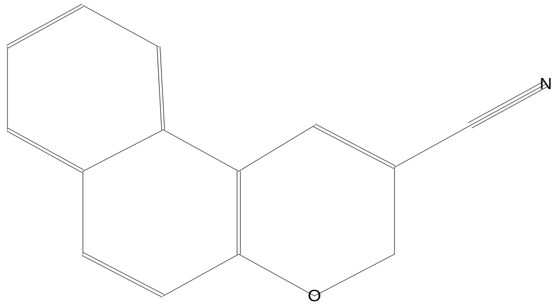

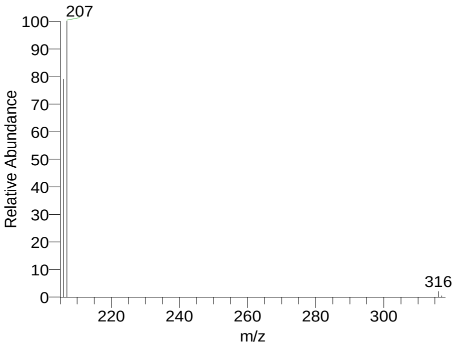

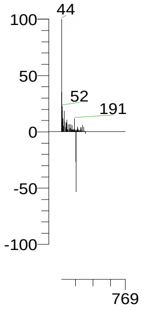

Ethyl 3-Butenyl Phosphite
Formula C18H21O3P, MW 316, CAS# NA, Entry# 408567

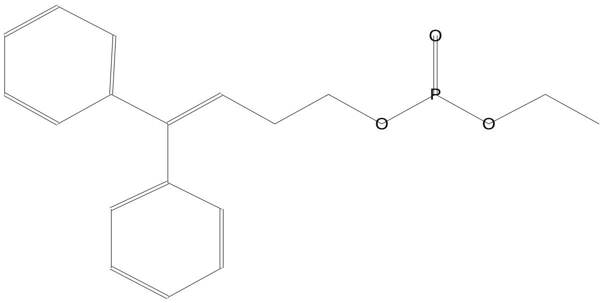

There is no signature data to report.

Library Search Report

Hit Spectrum | Delta | Compound Structure

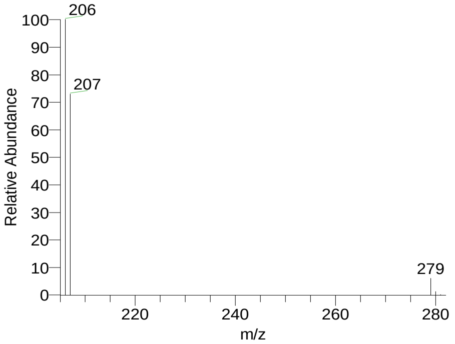

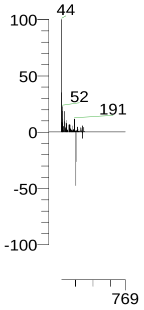

Ethyl 2-Cyano-2-(p-tolyl)-2-phenylacetate
Formula C18H17NO2, MW 279, CAS# NA, Entry# 327560

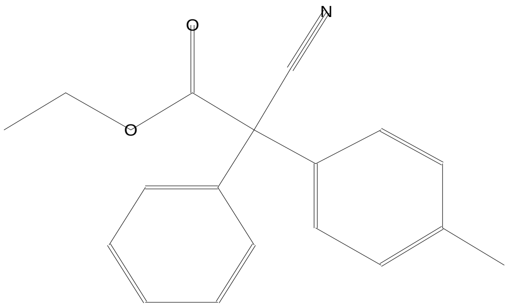

There is no signature data to report.

Library Search Report

RT	Probability	Compound Name	SI	Area %	Area	Molecular Weight	Molecular Formula	Library
5.48	30.07	2-(p-Methoxyphenyl)-4-(p-chlorophenyl)pyridine	533	0.57	1822562.63	295	C18H14ClNO	Wiley9
5.48	20.02	Methyl 9,10-epoxy-octadec-12-enoate	521	0.57	1822562.63	310	C19H34O3	Wiley9
5.48	9.12	EBURNAMINE	501	0.57	1822562.63	296	C19H24N2O	Wiley9

Faten-212 #141 RT: 5.48 AV: 1 RF: 6.00, 3 NL: 2.81E4
F: {0,0} + c EI Full ms [40.00-800.00]

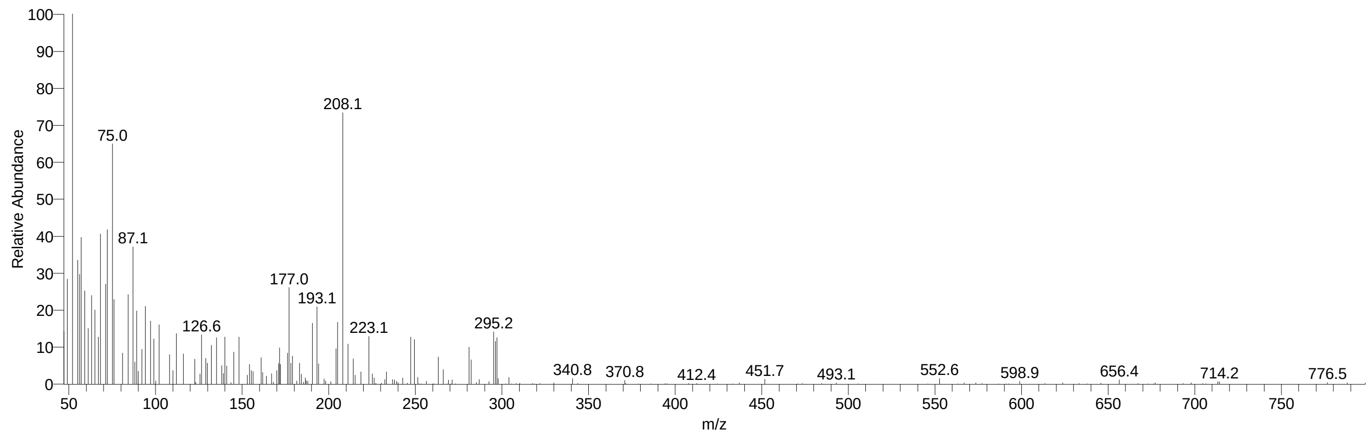

Hit Spectrum	Delta	Compound Structure

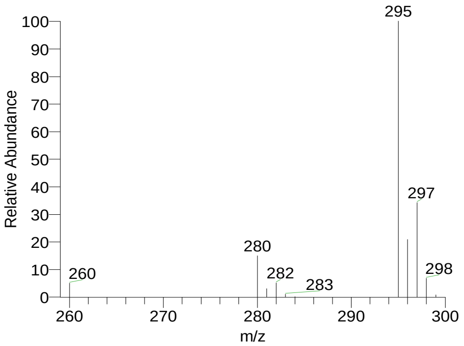

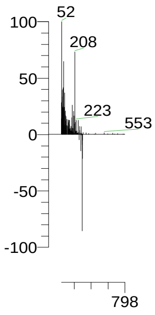

2-(p-Methoxyphenyl)-4-(p-chlorophenyl)pyridine
Formula C18H14ClNO, MW 295, CAS# NA, Entry# 363330

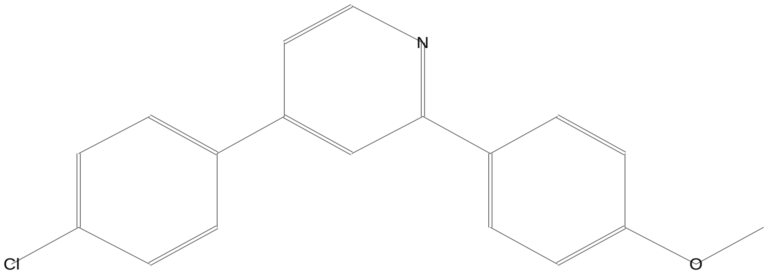

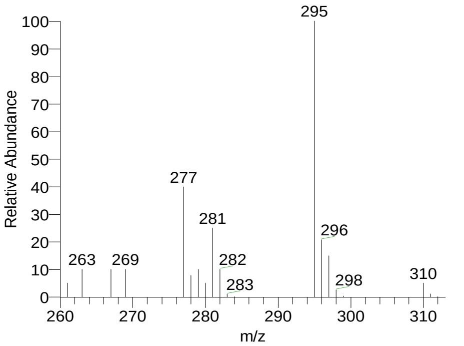

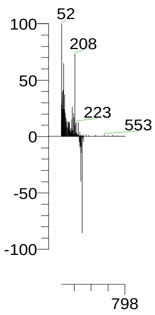

Methyl 9,10-epoxy-octadec-12-enoate
Formula C19H34O3, MW 310, CAS# NA, Entry# 396352

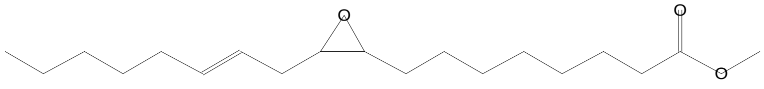

There is no signature data to report.

Library Search Report

Hit Spectrum	Delta	Compound Structure

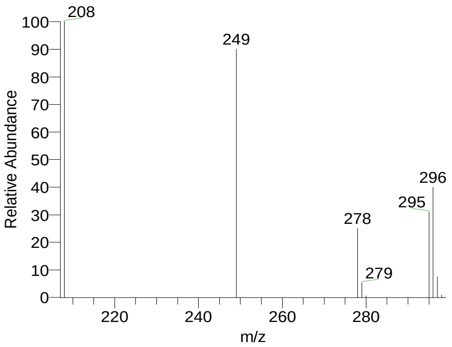

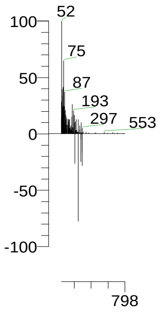

EBURNAMINE
Formula C19H24N2O, MW 296, CAS# NA, Entry# 366261
(14á)-14,15-Dihydro-eburnamenin-14-ol

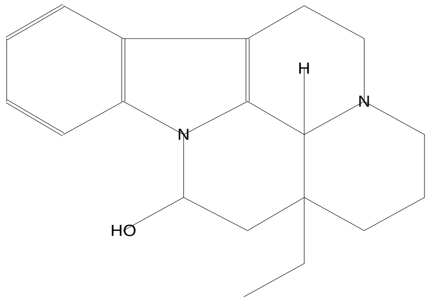

There is no signature data to report.

Library Search Report

RT	Probability	Compound Name	SI	Area %	Area	Molecular Weight	Molecular Formula	Library
6.05	31.58	5H-dibanro[b,f]azepine-5-carboxamide N-oxide-O*(18)	681	0.77	2459672.90	257	C15H15NO3	Wiley9
6.05	30.35	5H-dibanro[b,f]azepine-5-carboxamide N-oxide	680	0.77	2459672.90	257	C15H15NO3	Wiley9
6.05	13.82	6-Chloro-2-methyl-1,2-dihydroisoquinoline-3-carbaldehyde	660	0.77	2459672.90	207	C11H10ClNO	Wiley9

Faten-212 #308 RT: 6.05 AV: 1 RF: 6.00, 3 NL: 4.04E4
F: {0,0} + c EI Full ms [40.00-800.00]

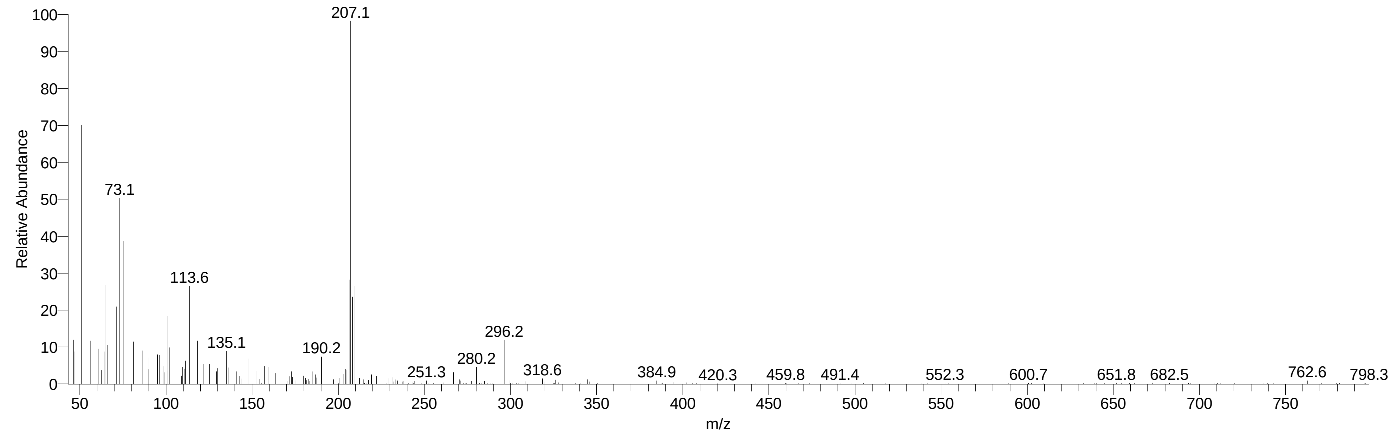

Hit Spectrum	Delta	Compound Structure

5H-dibanro[b,f]azepine-5-carboxamide N-oxide-O*(18)
Formula C15H15NO3, MW 257, CAS# NA, Entry# 276311

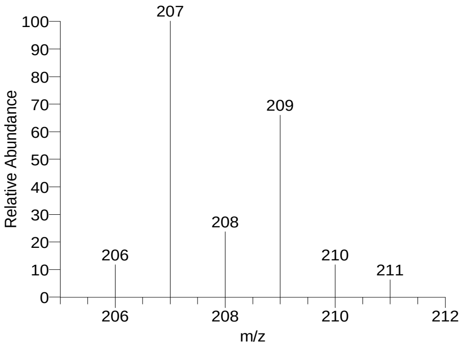

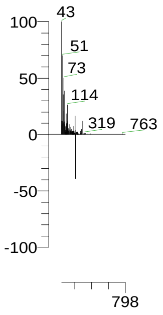

5H-dibanro[b,f]azepine-5-carboxamide N-oxide
Formula C15H15NO3, MW 257, CAS# NA, Entry# 276241

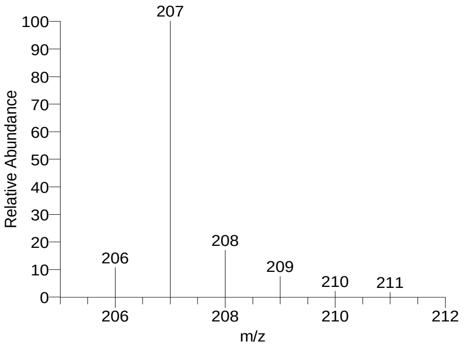

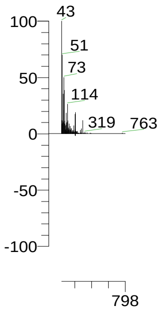

There is no signature data to report.

Library Search Report

Hit Spectrum	Delta	Compound Structure

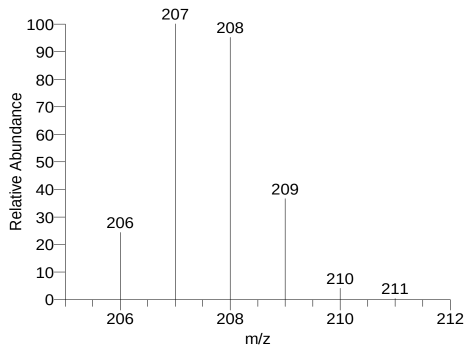

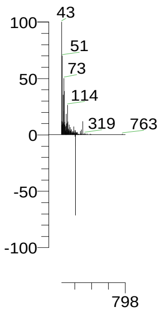

6-Chloro-2-methyl-1,2-dihydroisoquinoline-3-carbaldehyde
Formula C11H10ClNO, MW 207, CAS# NA, Entry# 158034

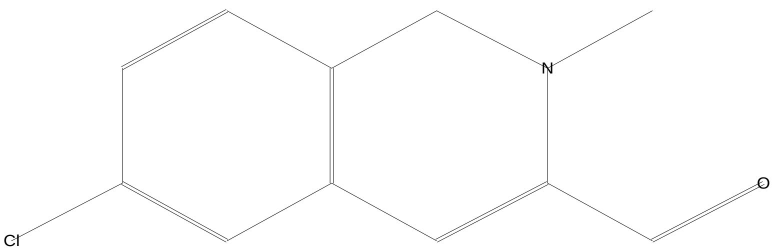

There is no signature data to report.

Library Search Report

RT	Probability	Compound Name	SI	Area %	Area	Molecular Weight	Molecular Formula	Library
6.09	23.30	7-Hydroxy-6-nitrocoumarin	541	0.32	1026114.65	207	C9H5NO5	Wiley9
6.09	22.39	3-methoxy-2,5,6-trimethylphenyl 2,4-dimethoxy-3,5,6-trimethylbenzoate	540	0.32	1026114.65	372	C22H28O5	Wiley9
6.09	16.26	3,4-Methylenedioxy-2-trimethylsilylbenzenecarbaldehyde	531	0.32	1026114.65	222	C11H14O3Si	Wiley9

Faten-212 #321 RT: 6.09 AV: 1 RF: 6.00, 3 NL: 5.63E4
F: {0,0} + c EI Full ms [40.00-800.00]

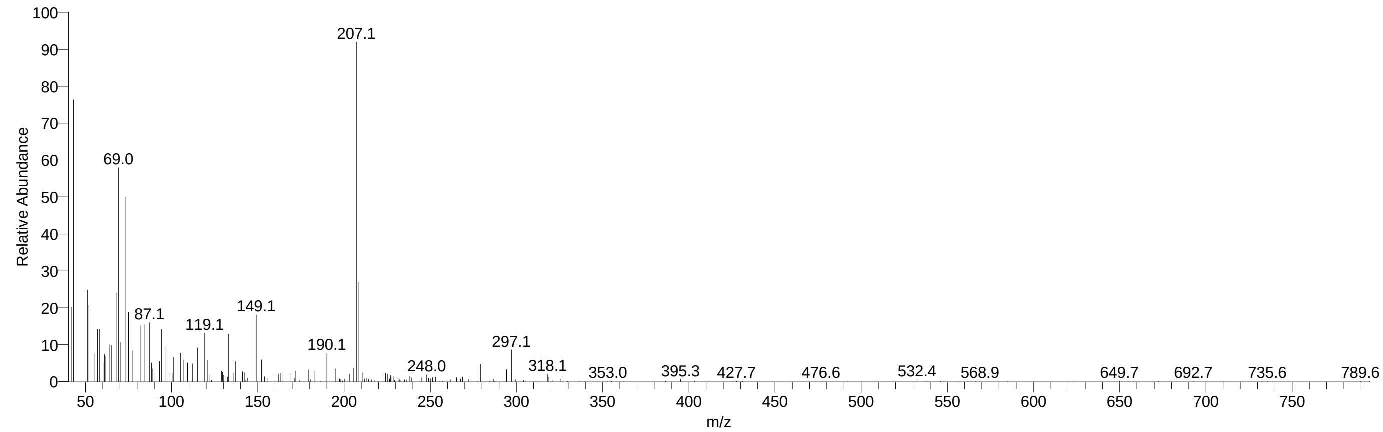

Hit Spectrum	Delta	Compound Structure

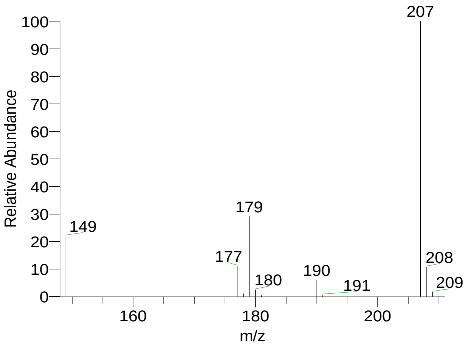

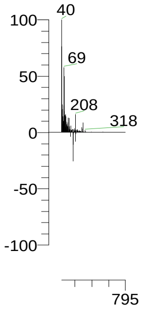

7-Hydroxy-6-nitrocoumarin
Formula C9H5NO5, MW 207, CAS# NA, Entry# 157807

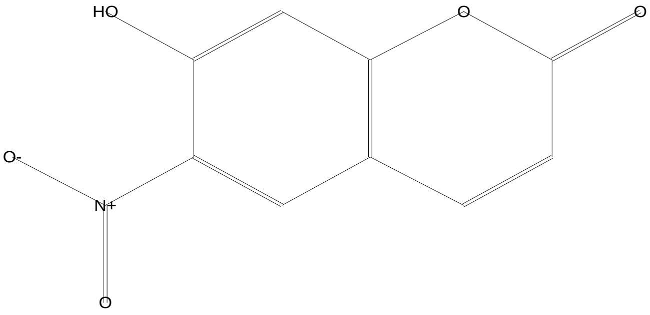

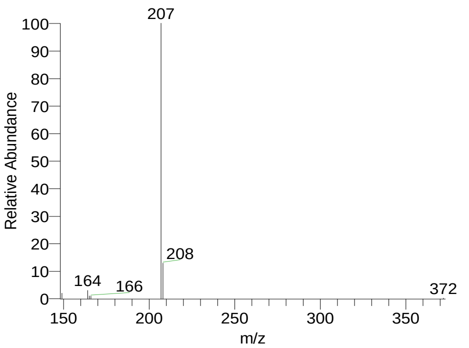

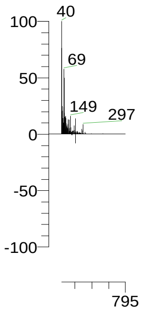

3-methoxy-2,5,6-trimethylphenyl 2,4-dimethoxy-3,5,6-trimethylbenzoate
Formula C22H28O5, MW 372, CAS# NA, Entry# 506606

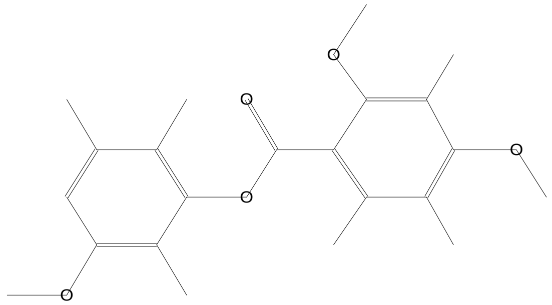

There is no signature data to report.

Library Search Report

Hit Spectrum	Delta	Compound Structure

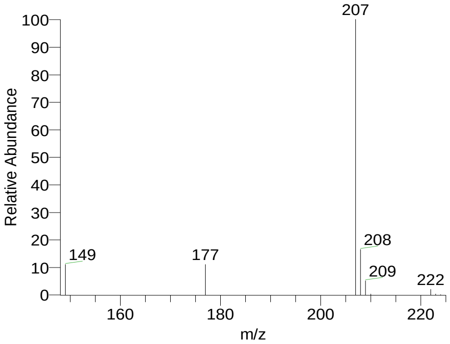

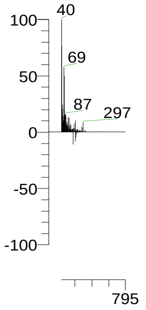

3,4-Methylenedioxy-2-trimethylsilylbenzenecarbaldehyde
Formula C11H14O3Si, MW 222, CAS# NA, Entry# 192034

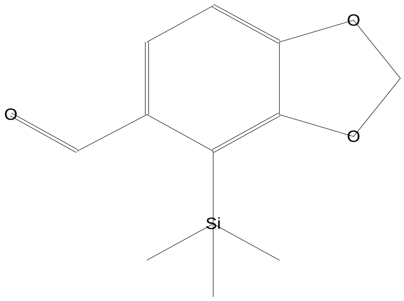

There is no signature data to report.

Library Search Report

RT	Probability	Compound Name	SI	Area %	Area	Molecular Weight	Molecular Formula	Library
6.77	11.53	1-[2-Nitro-5-chlorophenyl]-1-buten-3-ol	382	0.18	558797.44	227	C10H10ClNO3	mainlib
6.77	11.53	1-[2-Nitro-5-chlorophenyl]-1-buten-3-ol	382	0.18	558797.44	227	C10H10ClNO3	Wiley9
6.77	6.99	à-d-Glucofuranosyl benzenesulfonate	367	0.18	558797.44	320	C12H16O8S	mainlib

Faten-212 #519 RT: 6.77 AV: 1 RF: 6.00, 3 NL: 5.18E4
F: {0,0} + c EI Full ms [40.00-800.00]

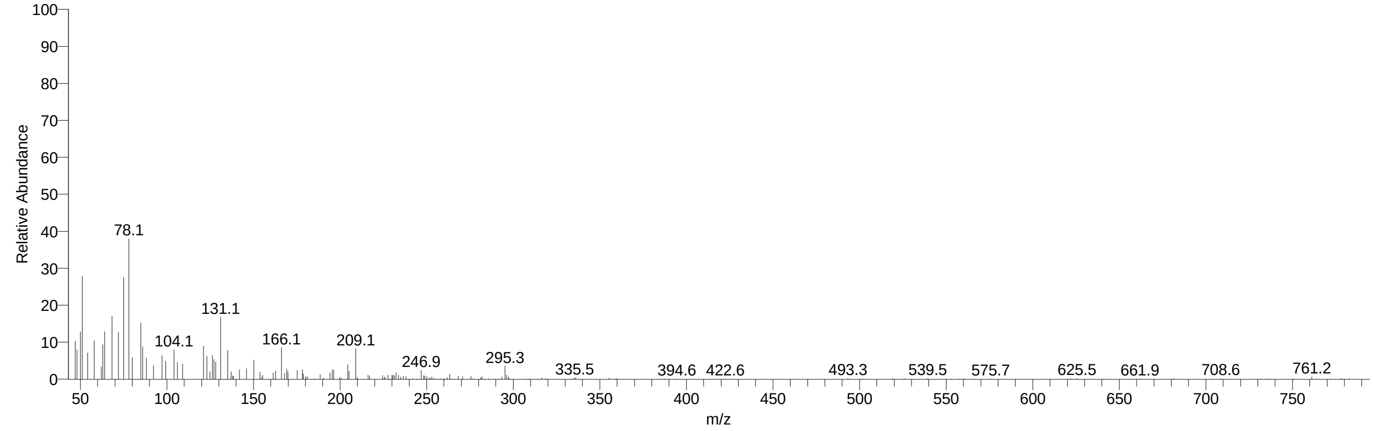

Hit Spectrum	Delta	Compound Structure

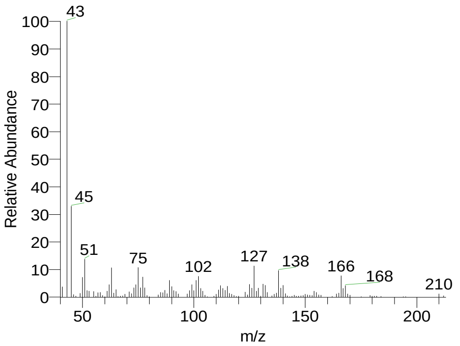

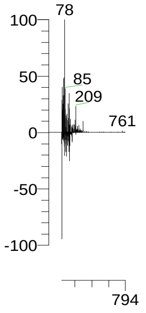

1-[2-Nitro-5-chlorophenyl]-1-buten-3-ol
Formula C10H10ClNO3, MW 227, CAS# NA, Entry# 6494
(3E)-4-(5-Chloro-2-nitrophenyl)-3-buten-2-ol #

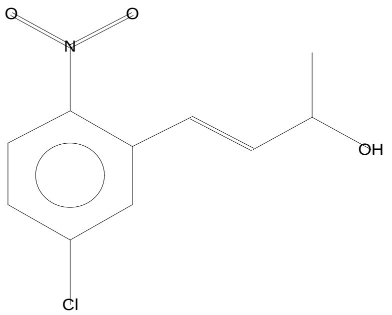

1-[2-Nitro-5-chlorophenyl]-1-buten-3-ol
Formula C10H10ClNO3, MW 227, CAS# NA, Entry# 204929
(3E)-4-(5-Chloro-2-nitrophenyl)-3-buten-2-ol

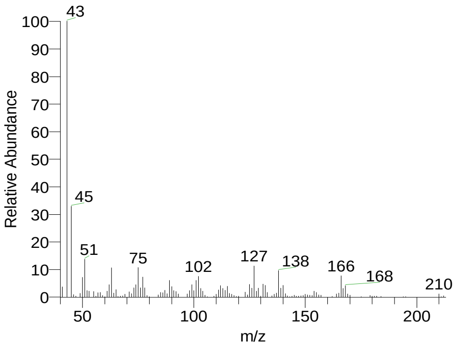

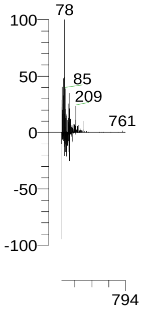

There is no signature data to report.

Library Search Report

Hit Spectrum | Delta | Compound Structure

à-d-Glucofuranosyl benzenesulfonate
Formula C12H16O8S, MW 320, CAS# NA, Entry# 50165
1-O-(Phenylsulfonyl)hexofuranose #

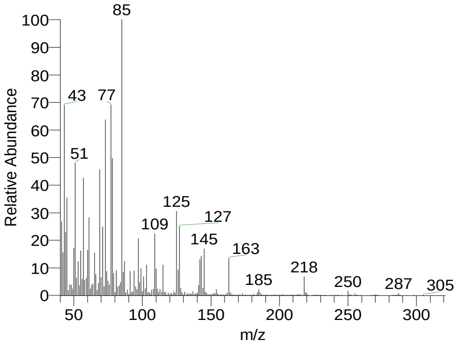

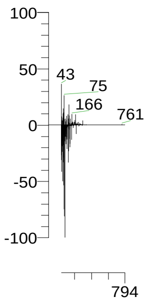

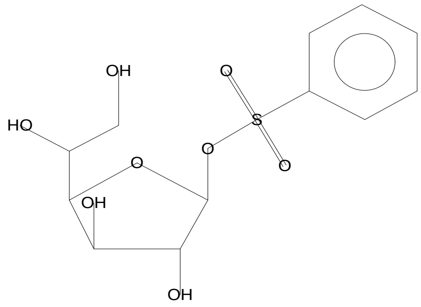

There is no signature data to report.

Library Search Report

RT	Probability	Compound Name	SI	Area %	Area	Molecular Weight	Molecular Formula	Library
6.93	11.19	1,3-Diazatricyclo[3.3.1.1(3,7)]decan-6-one, 2-(5-methoxy-1H-indol-3-yl)-5-methyl-7-propyl-	446	0.66	2108596.78	353	C21H27N3O2	mainlib
6.93	11.19	1,3-DIAZATRICYCLO[3.3.1.1(3,7)]DECAN-6-ONE, 2-(5-METHOXY-1H-INDOL-3-YL)-5-METHYL-7-PROPYL-	446	0.66	2108596.78	353	C21H27N3O2	Wiley9
6.93	9.02	1,3-Diazatricyclo[3.3.1.1(3,7)]decan-6-one, 5,7-diethyl-2-(5-methoxy-1H-indol-3-yl)-	441	0.66	2108596.78	353	C21H27N3O2	mainlib

Faten-212 #566 RT: 6.93 AV: 1 RF: 6.00, 3 NL: 1.93E4
F: {0,0} + c EI Full ms [40.00-800.00]

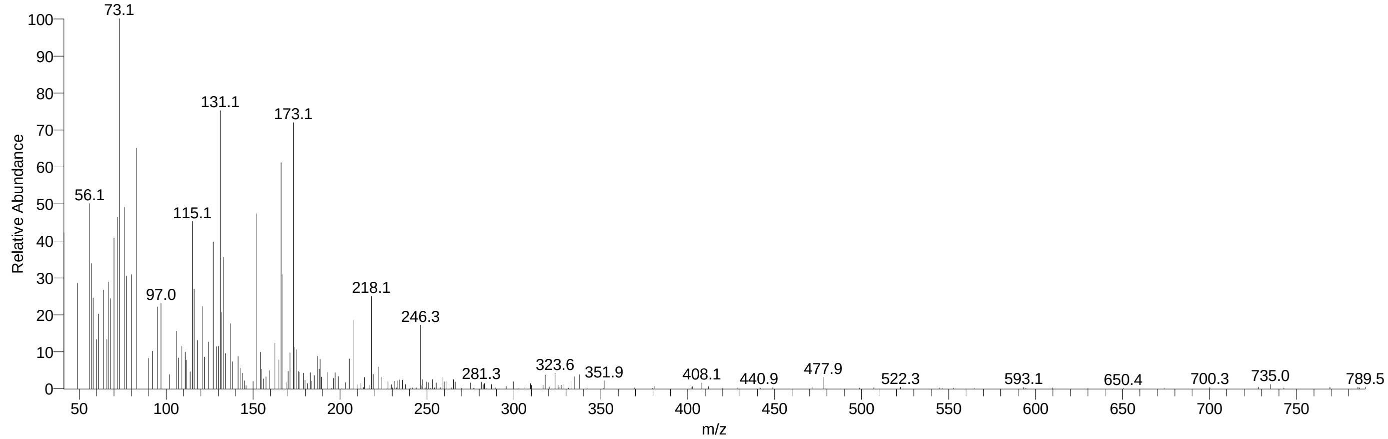

Hit Spectrum	Delta	Compound Structure

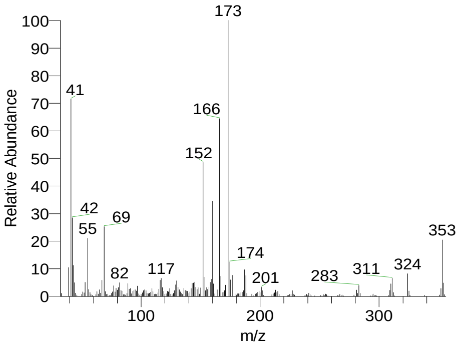

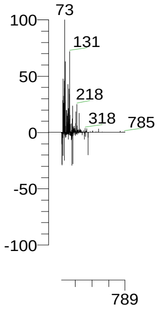

1,3-Diazatricyclo[3.3.1.1(3,7)]decan-6-one, 2-(5-methoxy-1H-indol-3-yl)-5-methyl-7-propyl-
Formula C21H27N3O2, MW 353, CAS# NA, Entry# 142477

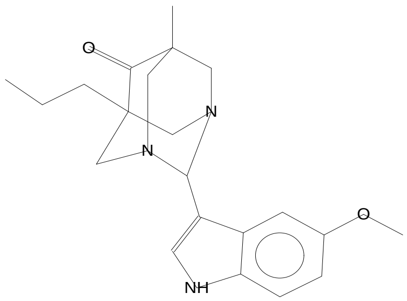

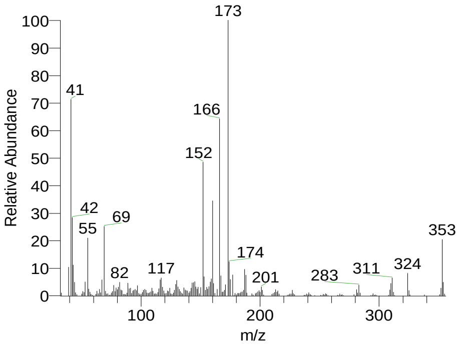

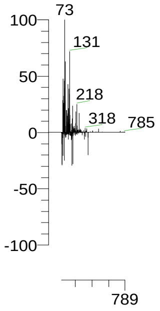

Formula C21H27N3O2, MW 353, CAS# NA, Entry# 477099

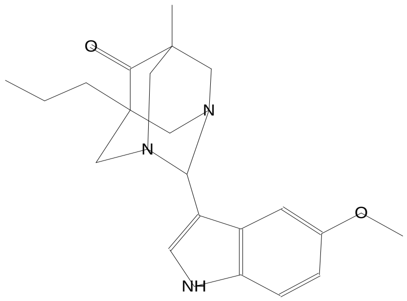

There is no signature data to report.

Library Search Report

Hit Spectrum	Delta	Compound Structure

1,3-Diazatricyclo[3.3.1.1(3,7)]decan-6-one, 5,7-diethyl-2-(5-methoxy-1H-indol-3-yl)-
Formula C21H27N3O2, MW 353, CAS# NA, Entry# 143107

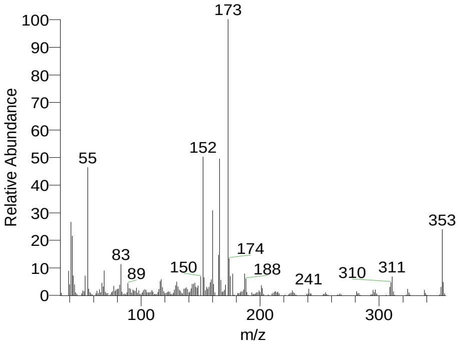

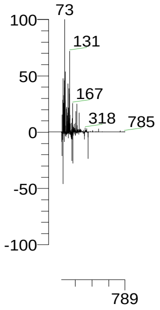

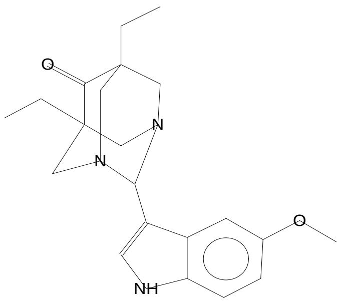

There is no signature data to report.

Library Search Report

RT	Probability	Compound Name	SI	Area %	Area	Molecular Weight	Molecular Formula	Library
7.33	12.64	Ethanedioic acid, bis(trimethylsilyl) ester (CAS)	644	0.28	895707.58	234	C8H18O4Si2	Wiley9
7.33	12.64	Ethanedioic acid, bis(trimethylsilyl) ester (CAS)	639	0.28	895707.58	234	C8H18O4Si2	Wiley9
7.33	12.64	Ethanedioic acid, bis(trimethylsilyl) ester	634	0.28	895707.58	234	C8H18O4Si2	mainlib

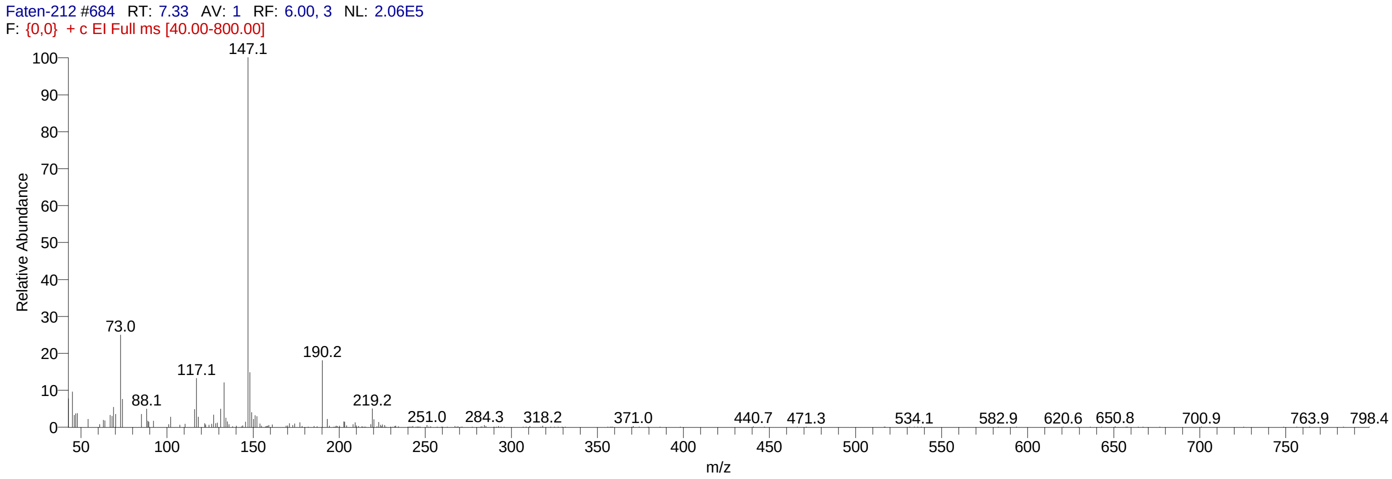

Hit Spectrum	Delta	Compound Structure

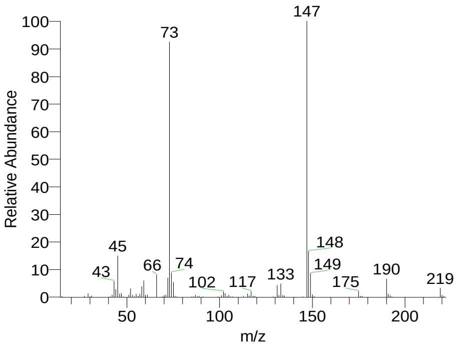

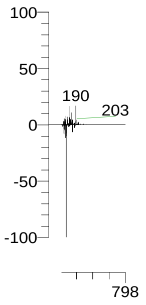

Ethanedioic acid, bis(trimethylsilyl) ester (CAS)
Formula C8H18O4Si2, MW 234, CAS# 18294-04-7, Entry# 219690
OXALIC ACID-DITMS

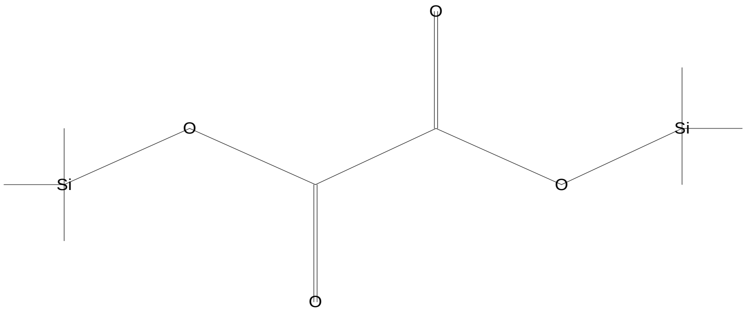

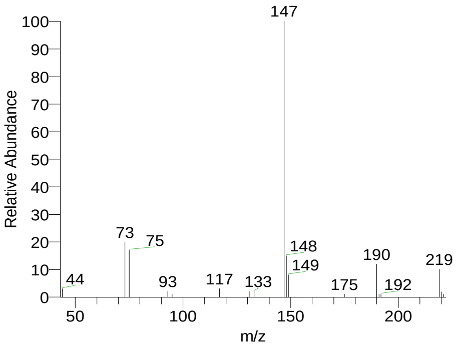

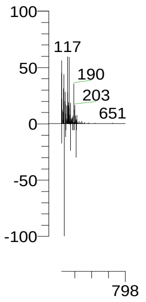

Ethanedioic acid, bis(trimethylsilyl) ester (CAS)
Formula C8H18O4Si2, MW 234, CAS# 18294-04-7, Entry# 219692
OXALIC ACID-DITMS

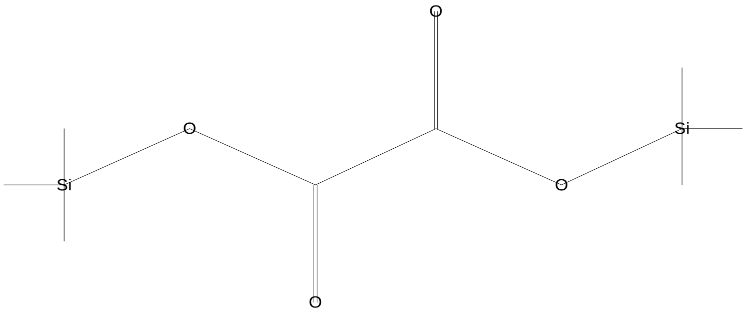

There is no signature data to report.

Library Search Report

Hit Spectrum	Delta	Compound Structure

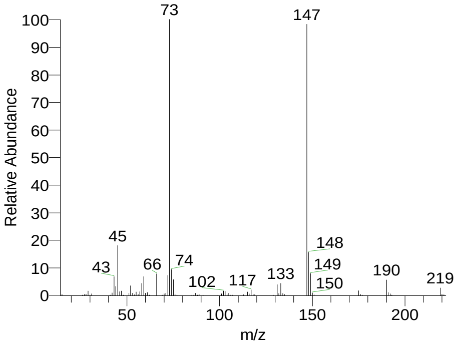

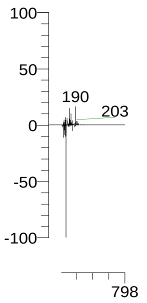

Ethanedioic acid, bis(trimethylsilyl) ester
Formula C8H18O4Si2, MW 234, CAS# 18294-04-7, Entry# 38695
Oxalic acid, bis(trimethylsilyl) ester

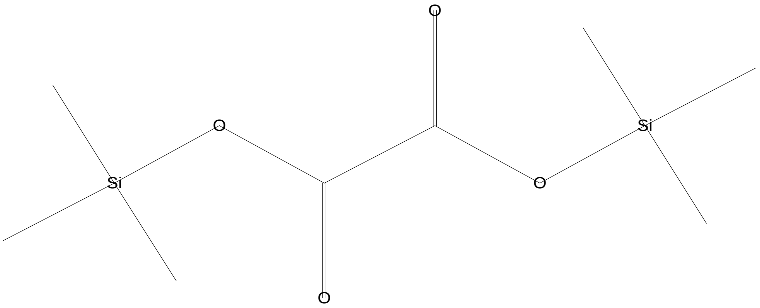

There is no signature data to report.

Library Search Report

RT	Probability	Compound Name	SI	Area %	Area	Molecular Weight	Molecular Formula	Library
7.50	22.96	Ethylene glycol butyl ether, tert-butyldimethylsilyl ether	610	7.82	24838879.42	232	C12H28O2Si	mainlib
7.50	14.83	Ethanol, 2-[(trimethylsilyl)oxy]-	597	7.82	24838879.42	134	C5H14O2Si	mainlib
7.50	8.09	Silylated Formaldehyde o-[2-(aminooxy)ethyl]oxime	580	7.82	24838879.42	176	C6H16N2O2Si	Wiley9

Faten-212 #735 RT: 7.50 AV: 1 RF: 6.00, 3 NL: 2.19E5
F: {0,0} + c EI Full ms [40.00-800.00]

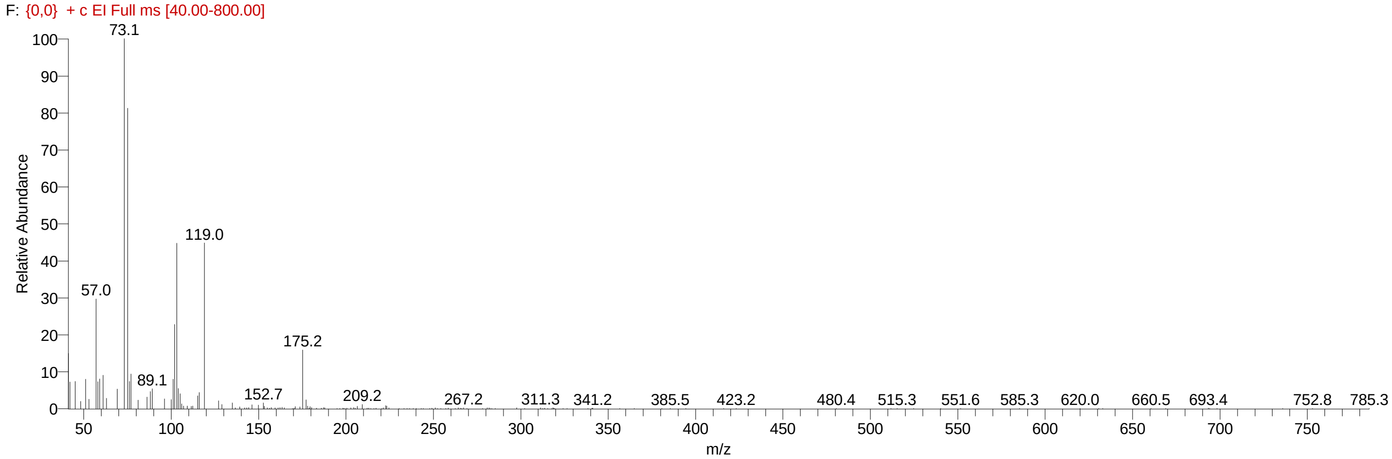

Hit Spectrum	Delta	Compound Structure

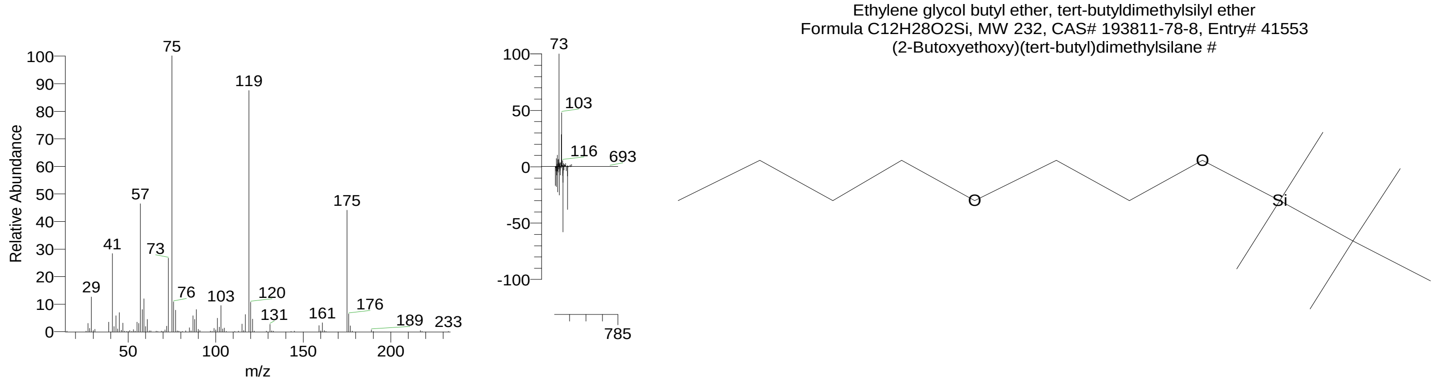

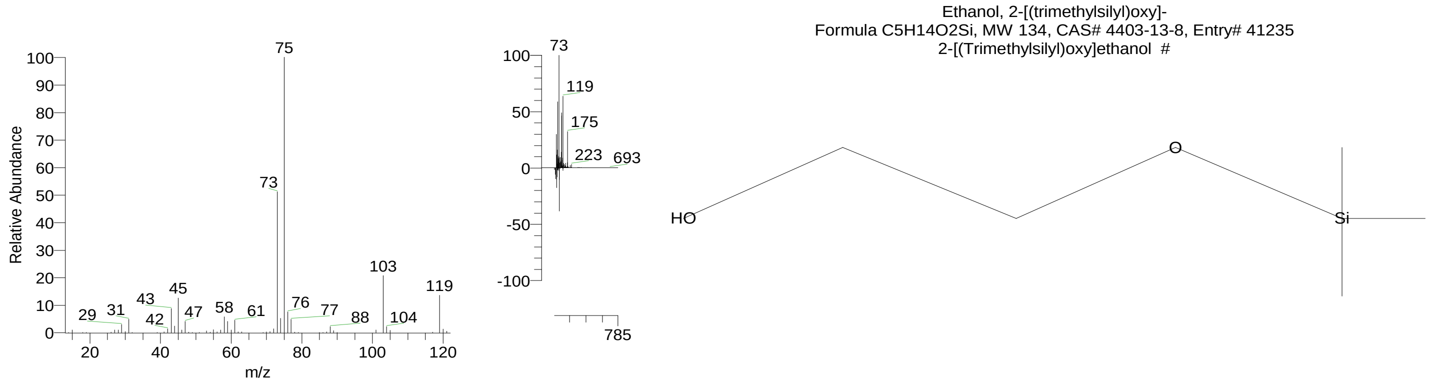

There is no signature data to report.

Library Search Report

Hit Spectrum	Delta	Compound Structure

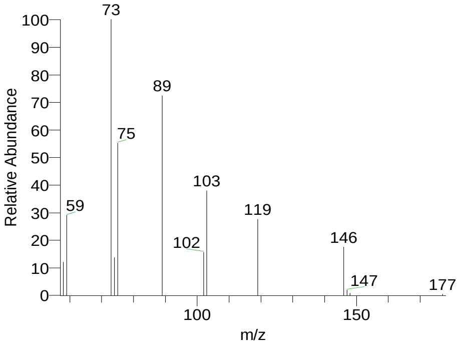

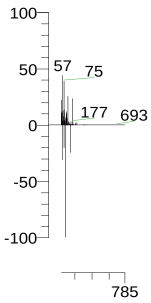

Silylated Formaldehyde o-[2-(aminooxy)ethyl]oxime
Formula C6H16N2O2Si, MW 176, CAS# 91523-96-5, Entry# 93195

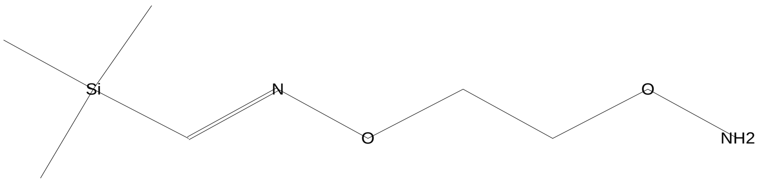

There is no signature data to report.

Library Search Report

RT	Probability	Compound Name	SI	Area %	Area	Molecular Weight	Molecular Formula	Library
7.62	36.17	Ethylene glycol butyl ether, tert-butyldimethylsilyl ether	594	10.15	32220813.98	232	C12H28O2Si	mainlib
7.62	9.07	2-Methylbutanoic acid, 3-(t-butyldimethylsilyloxy)-	559	10.15	32220813.98	232	C11H24O3Si	mainlib
7.62	9.07	2-Methylbutanoic acid, 3-(t-butyldimethylsilyloxy)-	559	10.15	32220813.98	232	C11H24O3Si	Wiley9

Faten-212 #770 RT: 7.62 AV: 1 RF: 6.00, 3 NL: 1.98E5
F: {0,0} + c EI Full ms [40.00-800.00]

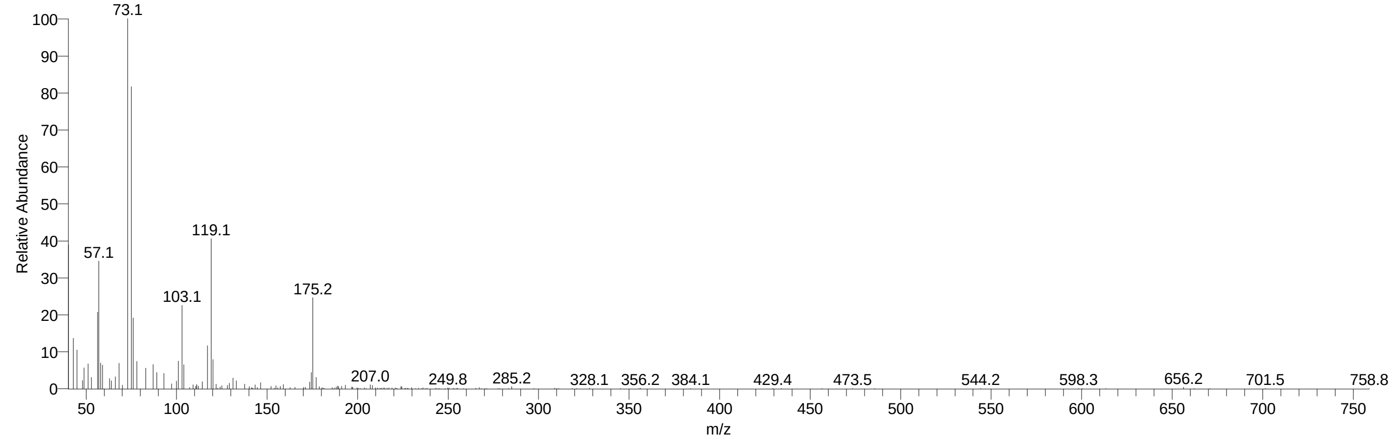

Hit Spectrum	Delta	Compound Structure

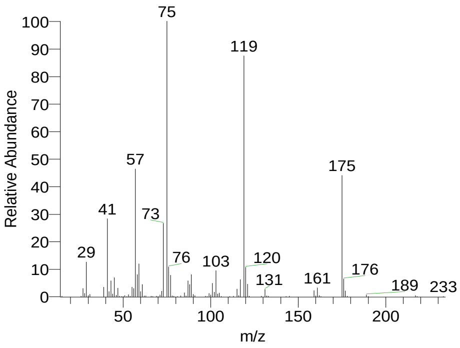

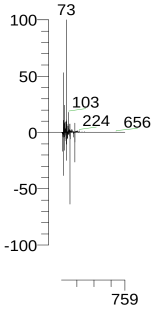

Ethylene glycol butyl ether, tert-butyldimethylsilyl ether
Formula C12H28O2Si, MW 232, CAS# 193811-78-8, Entry# 41553
(2-Butoxyethoxy)(tert-butyl)dimethylsilane #

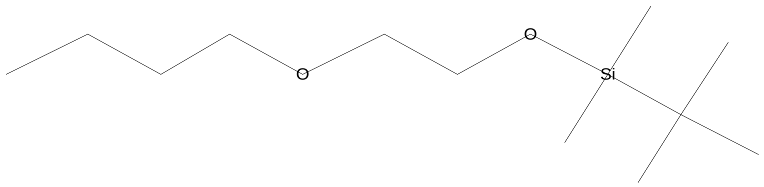

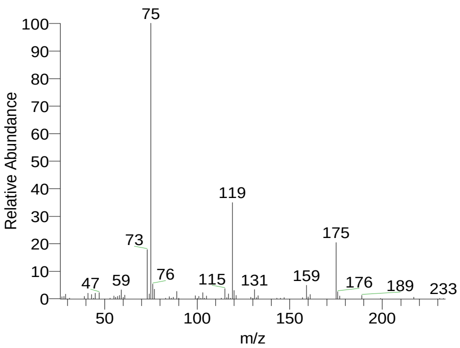

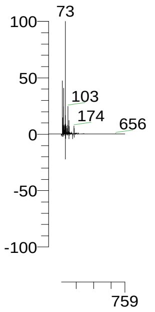

2-Methylbutanoic acid, 3-(t-butyldimethylsilyloxy)-
Formula C11H24O3Si, MW 232, CAS# 108782-00-9, Entry# 41558
3-([tert-Butyl(dimethyl)silyl]oxy)-2-methylbutanoic acid #

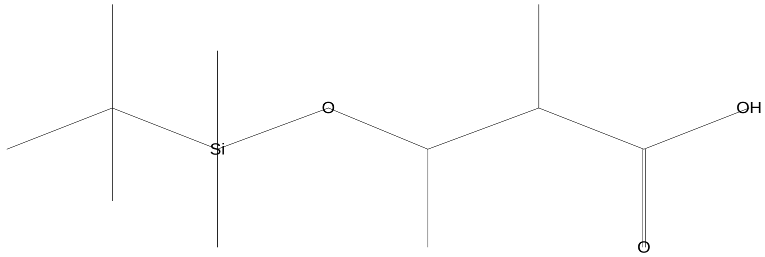

There is no signature data to report.

Library Search Report

Hit Spectrum	Delta	Compound Structure

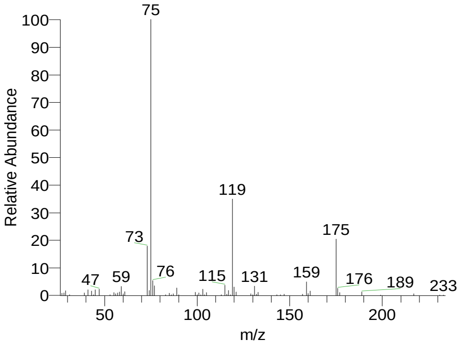

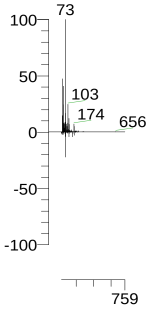

2-Methylbutanoic acid, 3-(t-butyldimethylsilyloxy)-
Formula C11H24O3Si, MW 232, CAS# 108782-00-9, Entry# 215760
3-([tert-Butyl(dimethyl)silyl]oxy)-2-methylbutanoic acid

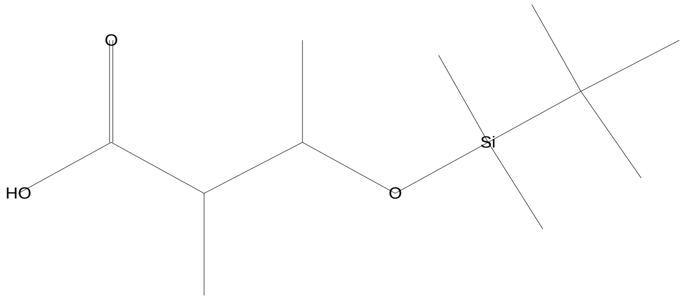

There is no signature data to report.

Library Search Report

RT	Probability	Compound Name	SI	Area %	Area	Molecular Weight	Molecular Formula	Library
7.71	17.66	Octadecylbenzene	471	7.12	22618652.74	330	C24H42	Wiley9
7.71	16.29	3-({2-[(3,5-DIMETHYL-1H-PYRAZOL-4-YL)SULFANYL]PHENYL}IMINO)-4,7-DIMETHYL-1,3-DIHYDRO-2H-INDOL-2-ONE	469	7.12	22618652.74	376	C21H20N4OS	Wiley9
7.71	4.70	N-Phenylsuccinimide	441	7.12	22618652.74	175	C10H9NO2	Wiley9

Faten-212 #796 RT: 7.71 AV: 1 RF: 6.00, 3 NL: 1.27E5
F: {0,0} + c EI Full ms [40.00-800.00]

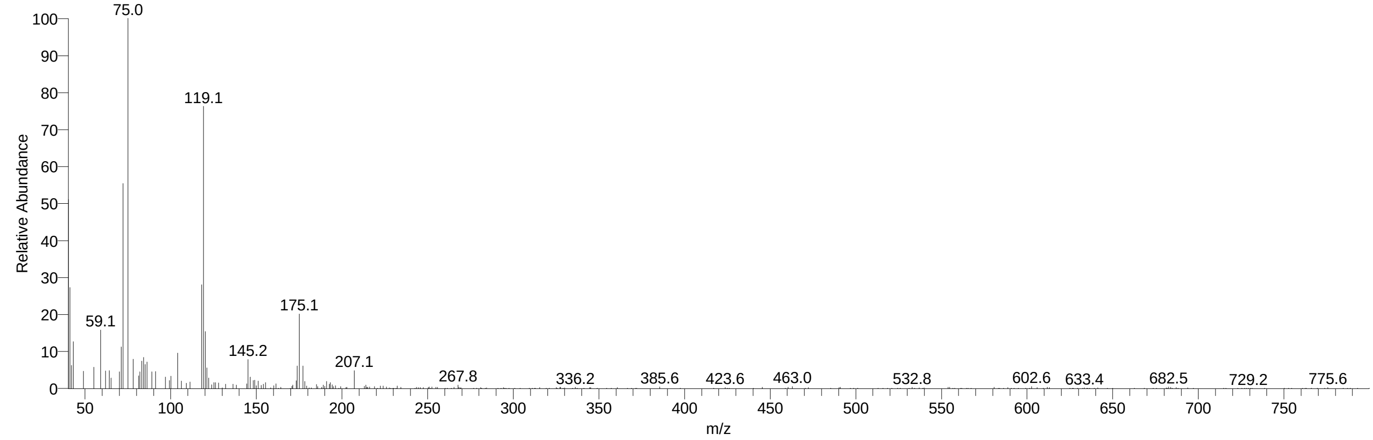

Hit Spectrum	Delta	Compound Structure

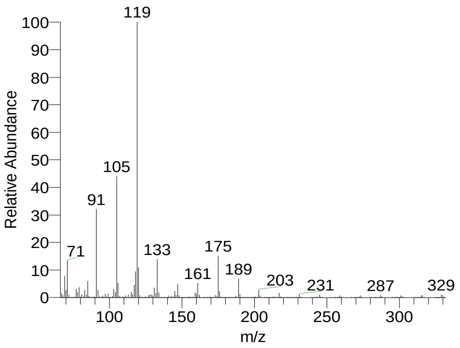

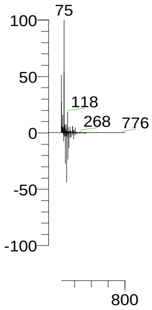

Octadecylbenzene
Formula C24H42, MW 330, CAS# 4445-07-2, Entry# 437211
Benzene, octadecyl-

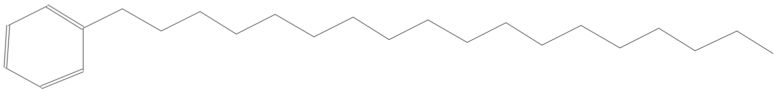

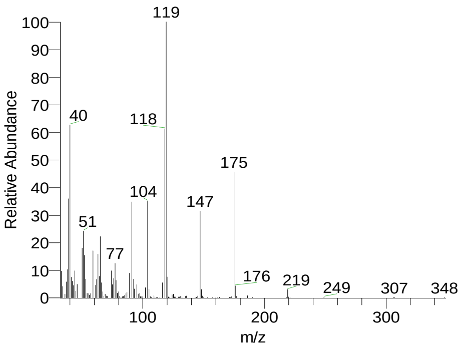

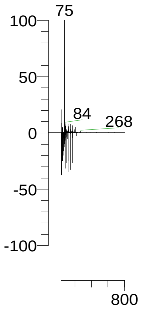

Formula C21H20N4OS, MW 376, CAS# NA, Entry# 512001

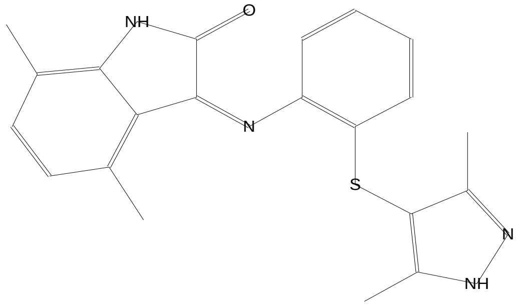

There is no signature data to report.

Library Search Report

Hit Spectrum	Delta	Compound Structure

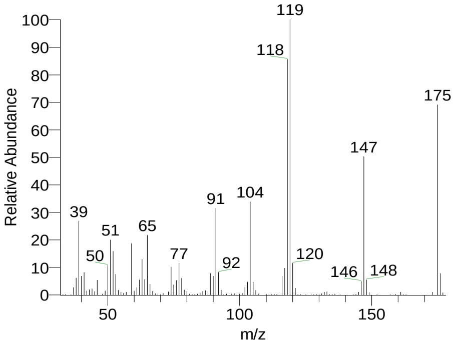

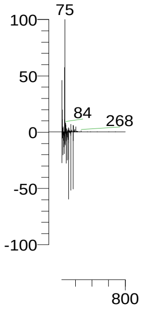

N-Phenylsuccinimide
Formula C10H9NO2, MW 175, CAS# 83-25-0, Entry# 92351
2,5-Dioxo-1-phenylpyrrolidine

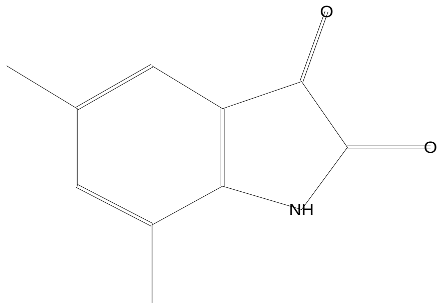

There is no signature data to report.

Library Search Report

RT	Probability	Compound Name	SI	Area %	Area	Molecular Weight	Molecular Formula	Library
8.69	10.32	1-Acetoxy-2-methylene-4-(trimethylsiloxy)decane	416	2.32	7354905.43	300	C16H32O3Si	Wiley9
8.69	7.70	methyl 11,12-bis(trimethylsilyloxy)octodecanoate	408	2.32	7354905.43	474	C25H54O4Si2	Wiley9
8.69	5.59	Diethyl (1S,2S,1'S)-2-ethyl-1-(1-phenylethylamino)cyclopropanephosphonate	399	2.32	7354905.43	325	C17H28NO3P	Wiley9

Faten-212 #1084 RT: 8.69 AV: 1 RF: 6.00, 3 NL: 9.46E4
F: {0,0} + c EI Full ms [40.00-800.00]

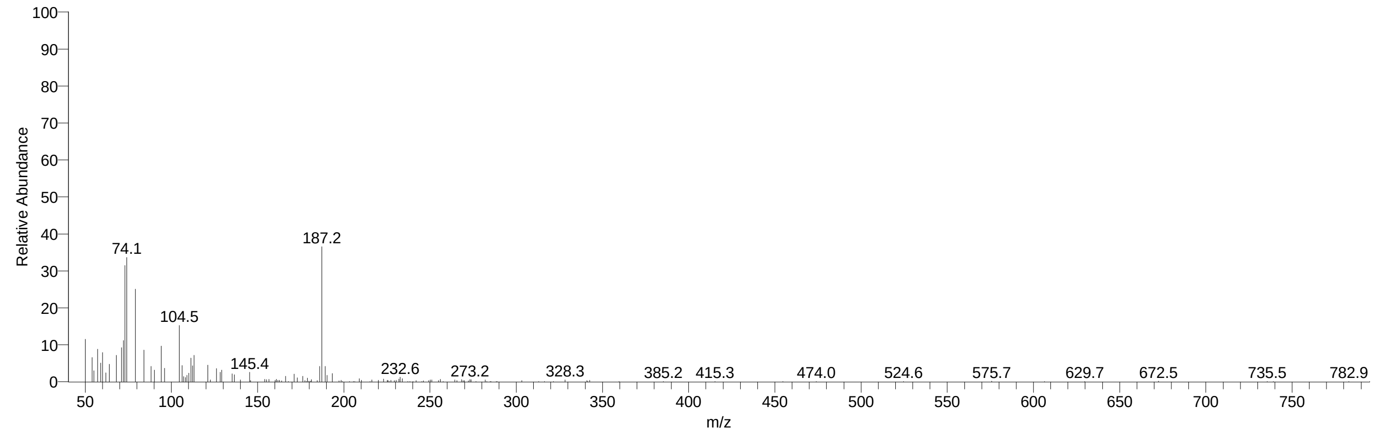

Hit Spectrum	Delta	Compound Structure

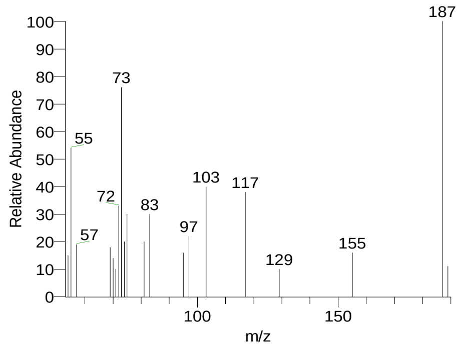

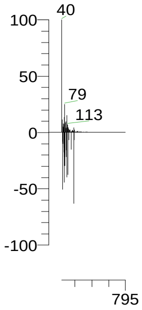

1-Acetoxy-2-methylene-4-(trimethylsiloxy)decane
Formula C16H32O3Si, MW 300, CAS# 84681-30-1, Entry# 374281
1-Decanol, 2-methylene-4-[(trimethylsilyl)oxy]-, acetate (CAS)

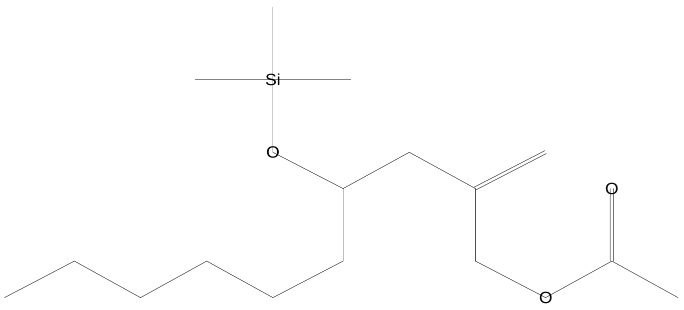

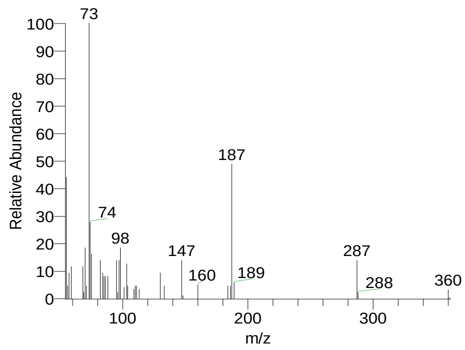

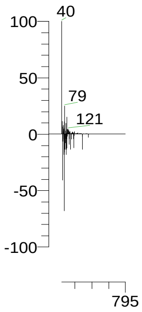

methyl 11,12-bis(trimethylsilyloxy)octodecanoate
Formula C25H54O4Si2, MW 474, CAS# NA, Entry# 604703

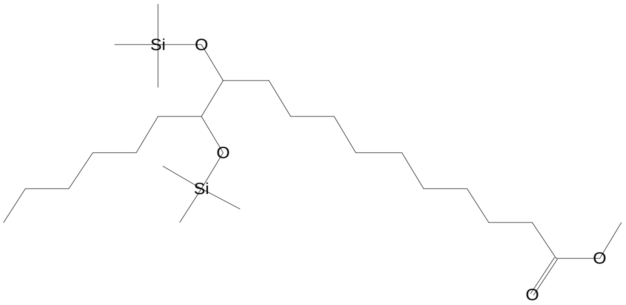

There is no signature data to report.

Library Search Report

Hit Spectrum	Delta	Compound Structure

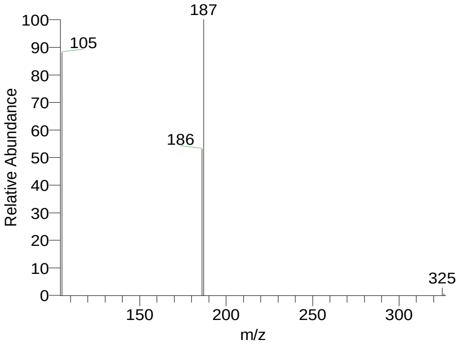

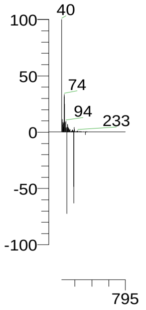

Diethyl (1S,2S,1'S)-2-ethyl-1-(1-phenylethylamino)cyclopropanephosphonate
Formula C17H28NO3P, MW 325, CAS# NA, Entry# 426131

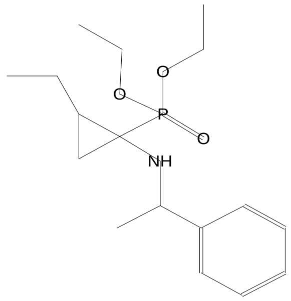

There is no signature data to report.

Library Search Report

RT	Probability	Compound Name	SI	Area %	Area	Molecular Weight	Molecular Formula	Library
9.83	5.96	[1,1'-Bicyclopropyl]-2-octanoic acid, 2'-hexyl-, methyl ester	428	0.16	517700.02	322	C21H38O2	mainlib
9.83	5.96	[1,1'-Bicyclopropyl]-2-octanoic acid, 2'-hexyl-, methyl ester (CAS)	428	0.16	517700.02	322	C21H38O2	Wiley9
9.83	5.03	1,1'-(4-Methyl-1,3-phenylene)bis[3-(5-benzyl-1,3,4-thiadiazol-2-yl)urea]	424	0.16	517700.02	556	C27H24N8O2S2	Wiley9

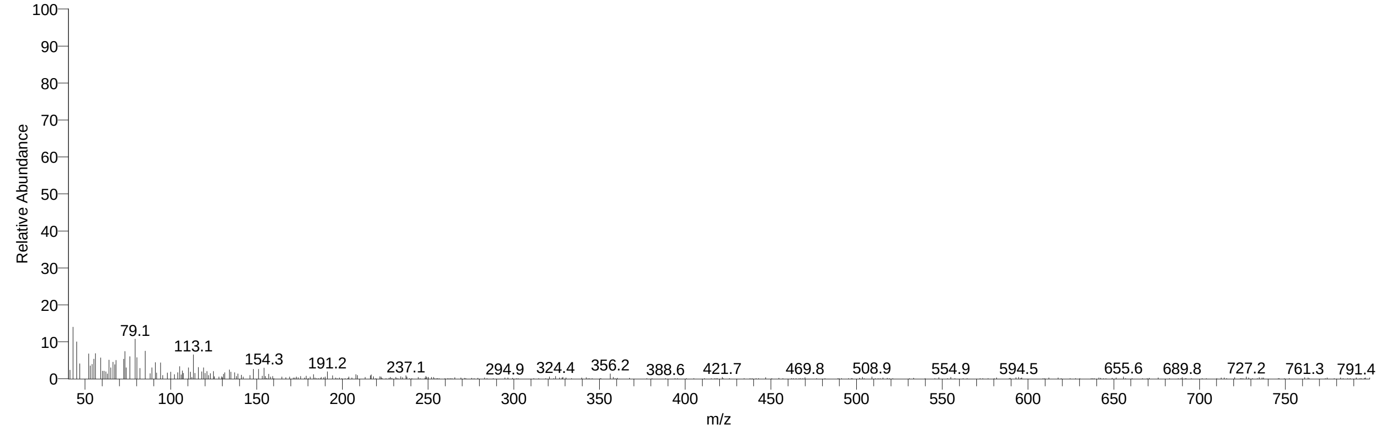

Hit Spectrum | Delta | Compound Structure

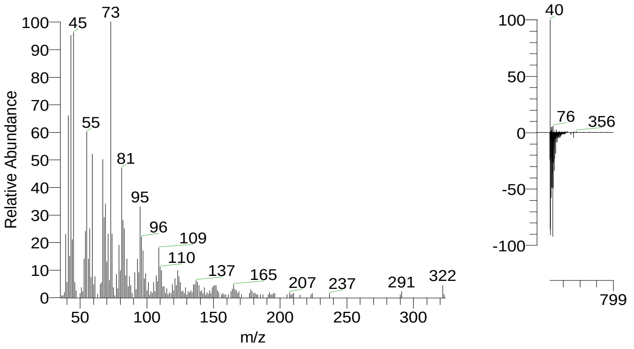

[1,1'-Bicyclopropyl]-2-octanoic acid, 2'-hexyl-, methyl ester
Formula C21H38O2, MW 322, CAS# 56687-68-4, Entry# 37105

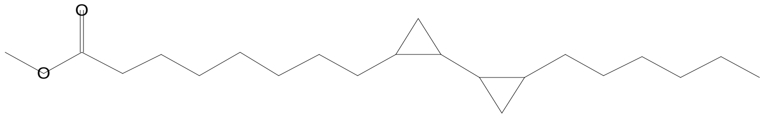

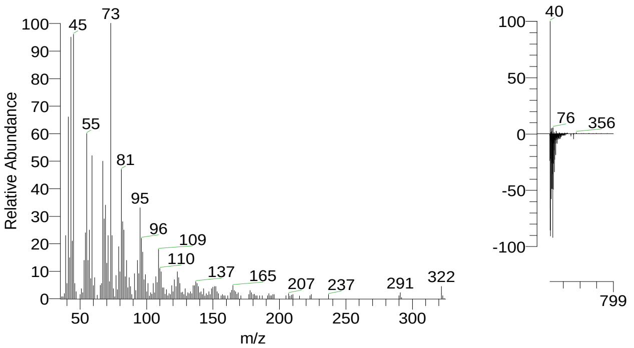

[1,1'-Bicyclopropyl]-2-octanoic acid, 2'-hexyl-, methyl ester (CAS)
Formula C21H38O2, MW 322, CAS# 56687-68-4, Entry# 421479
METHYL 9,10,11,12-DIMETHYLENE OCTADECANOATE

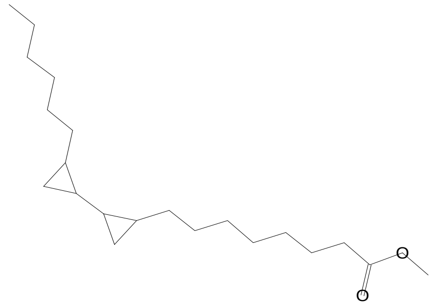

There is no signature data to report.

Library Search Report

Hit Spectrum	Delta	Compound Structure

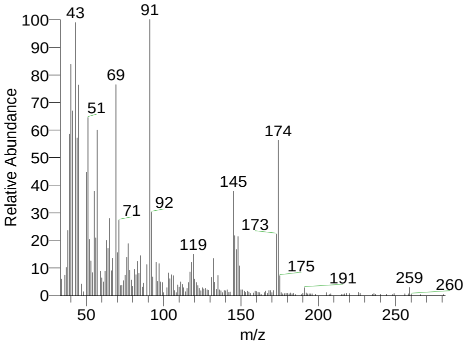

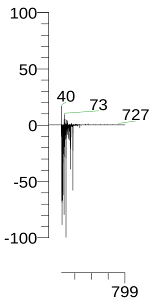

1,1'-(4-Methyl-1,3-phenylene)bis[3-(5-benzyl-1,3,4-thiadiazol-2-yl)urea]
Formula C27H24N8O2S2, MW 556, CAS# NA, Entry# 635480
1,1'-(4-METHYL-1,3-PHENYLENE)BIS[3-(5-BENZYL-1,3,4-THIADIAZOL-2-

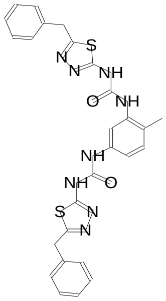

There is no signature data to report.

Library Search Report

RT	Probability	Compound Name	SI	Area %	Area	Molecular Weight	Molecular Formula	Library
10.06	39.04	1-[(2-trimethylsiloxy)vinyl]-4-trimethylsiloxy-2,6-dideuteriobenzene	477	0.14	435252.33	280	C14H22D2O2Si2	Wiley9
10.06	10.64	1-(2-trimethylsiloxy-1,1-dideuteriovinyl)-4-trimethylsiloxy-benzene	446	0.14	435252.33	280	C14H22D2O2Si2	Wiley9
10.06	7.51	1-(2-trimethylsiloxyvinyl)-4-trimethylsiloxy-3,5-dideuteriobenzene	436	0.14	435252.33	280	C14H22D2O2Si2	Wiley9

Faten-212 #1487 RT: 10.06 AV: 1 RF: 6.00, 3 NL: 1.44E5
F: {0,0} + c EI Full ms [40.00-800.00]

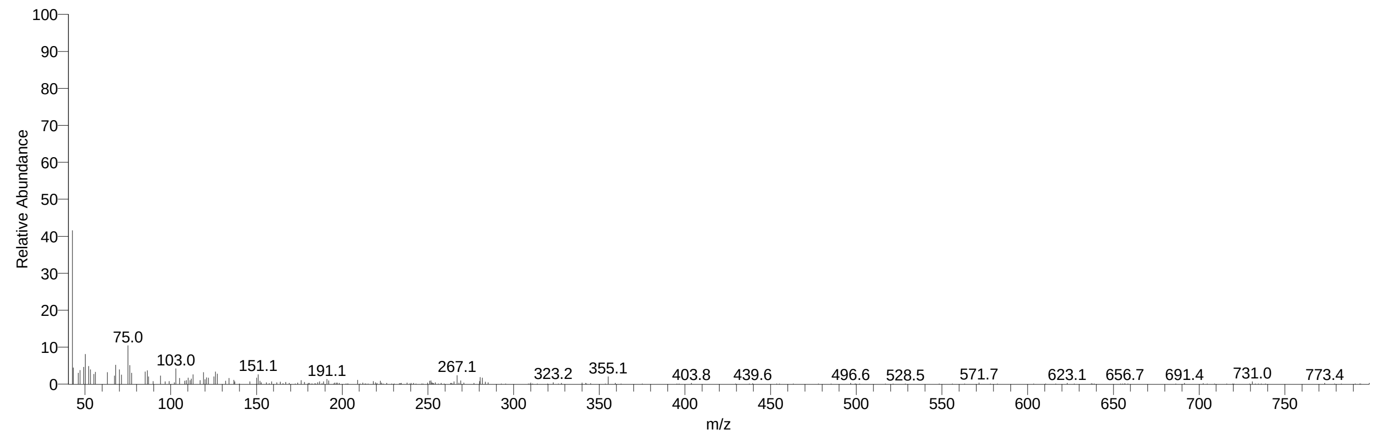

Hit Spectrum	Delta	Compound Structure

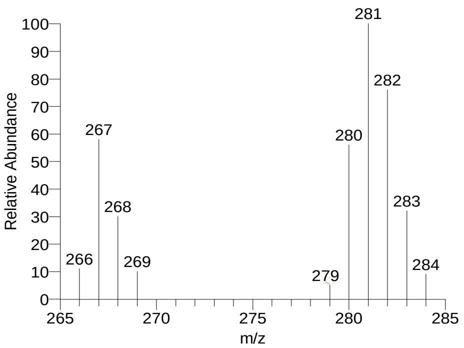

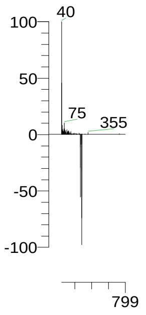

1-[(2-trimethylsiloxy)vinyl]-4-trimethylsiloxy-2,6-dideuteriobenzene
Formula C14H22D2O2Si2, MW 280, CAS# 126210-57-9, Entry# 328881
Silane, trimethyl[4-[1-[(trimethylsilyl)oxy]ethenyl]phenoxy-2,6-d2]- (CAS)

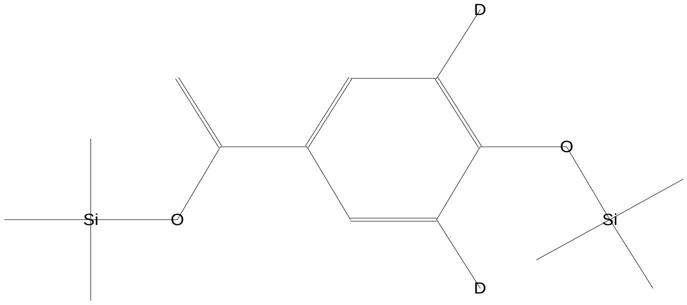

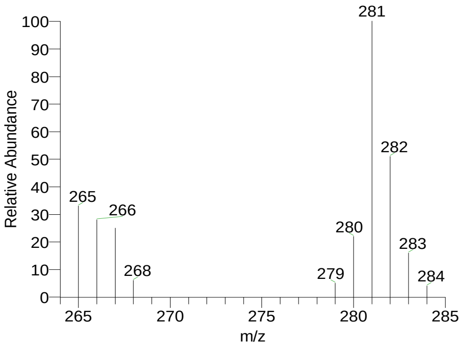

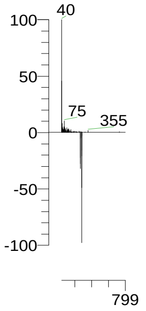

1-(2-trimethylsiloxy-1,1-dideuteriovinyl)-4-trimethylsiloxy-benzene
Formula C14H22D2O2Si2, MW 280, CAS# 126210-55-7, Entry# 328880
Silane, trimethyl[4-[1-[(trimethylsilyl)oxy]ethenyl-2,2-d2]phenoxy]- (CAS)

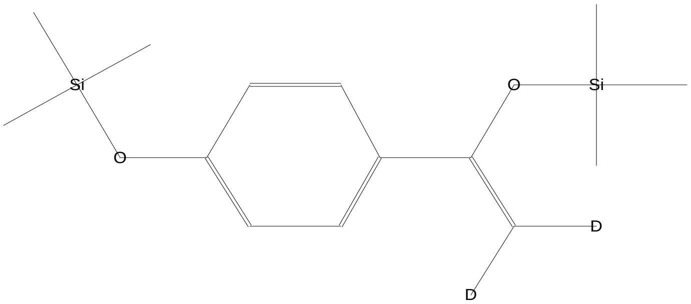

There is no signature data to report.

Library Search Report

Hit Spectrum	Delta	Compound Structure

1-(2-trimethylsiloxyvinyl)-4-trimethylsiloxy-3,5-dideuteriobenzene
Formula C14H22D2O2Si2, MW 280, CAS# 126210-56-8, Entry# 328882
Silane, trimethyl[4-[1-[(trimethylsilyl)oxy]ethenyl]phenoxy-3,5-d2]- (CAS)

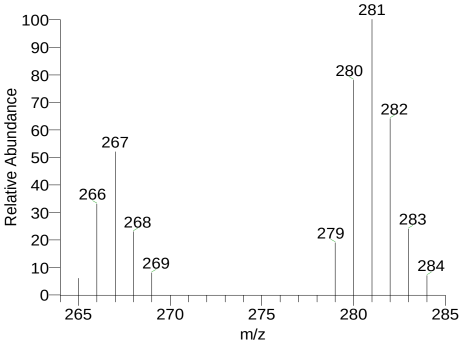

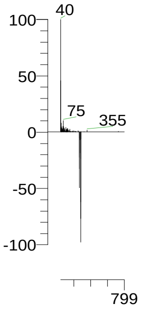

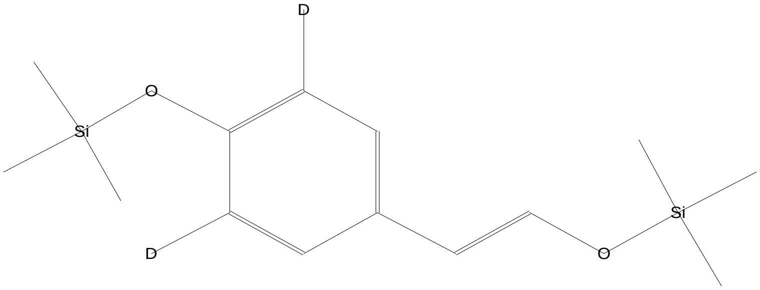

There is no signature data to report.

Library Search Report

RT	Probability	Compound Name	SI	Area %	Area	Molecular Weight	Molecular Formula	Library
11.08	86.60	1-(2-trimethylsiloxy-1,1-dideuteriovinyl)-4-trimethylsiloxy-benzene	753	1.55	4920818.77	280	C14H22D2O2Si2	Wiley9
11.08	7.32	(+)-(R)-Tanshindiol A	662	1.55	4920818.77	312	C18H16O5	Wiley9
11.08	1.91	dimethyl 6-diethylamino-2-methoxy-pyridine-3,4-dicarboxylate	629	1.55	4920818.77	296	C14H20N2O5	Wiley9

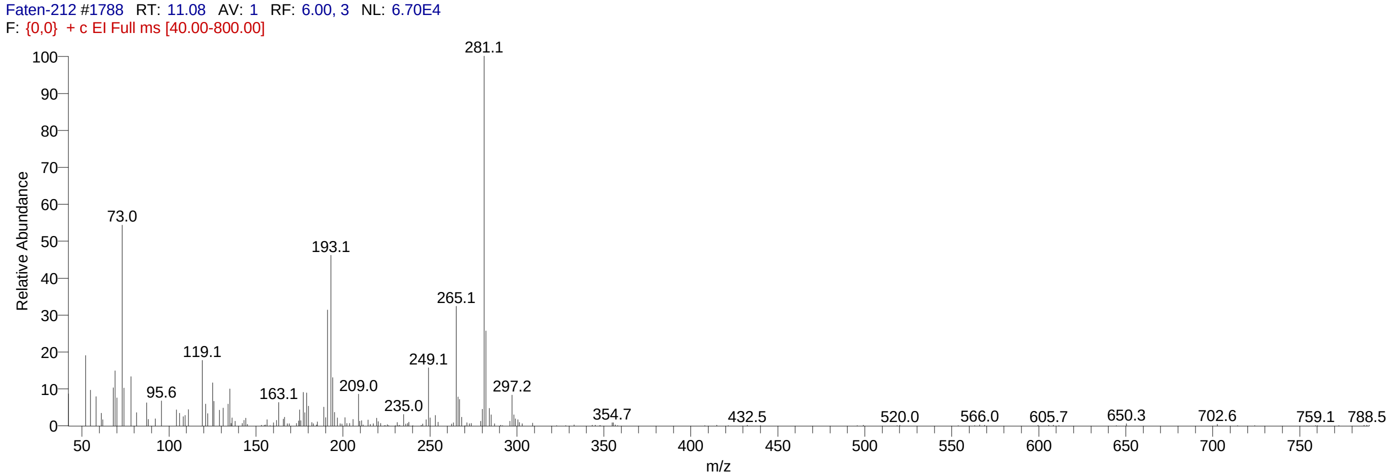

Hit Spectrum	Delta	Compound Structure

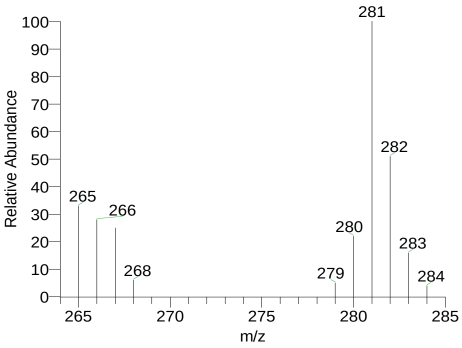

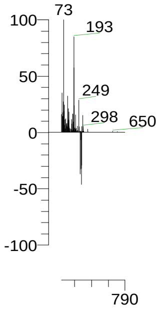

1-(2-trimethylsiloxy-1,1-dideuteriovinyl)-4-trimethylsiloxy-benzene
Formula C14H22D2O2Si2, MW 280, CAS# 126210-55-7, Entry# 328880
Silane, trimethyl[4-[1-[(trimethylsilyl)oxy]ethenyl-2,2-d2]phenoxy]- (CAS)

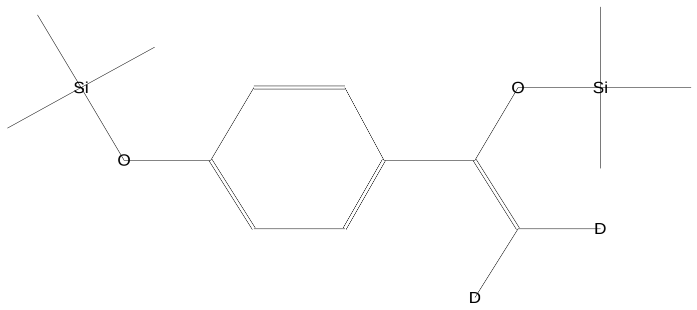

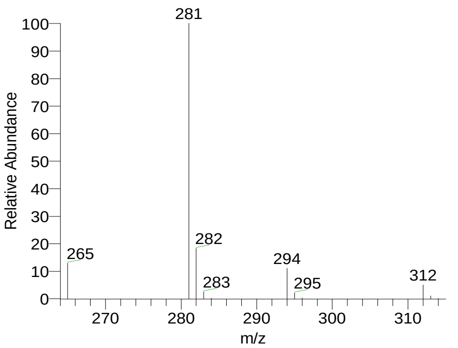

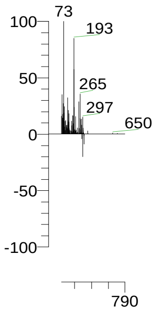

(+)-(R)-Tanshindiol A
Formula C18H16O5, MW 312, CAS# NA, Entry# 399923

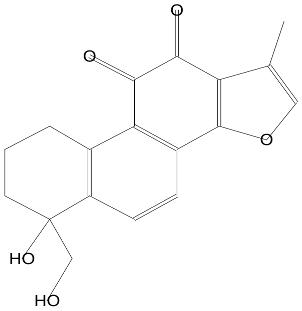

There is no signature data to report.

Library Search Report

Hit Spectrum	Delta	Compound Structure

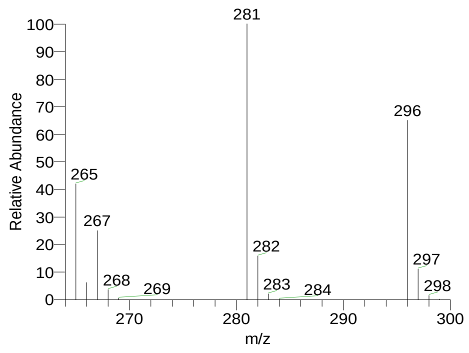

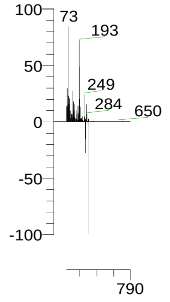

dimethyl 6-diethylamino-2-methoxy-pyridine-3,4-dicarboxylate
Formula C14H20N2O5, MW 296, CAS# 130879-61-7, Entry# 364577
3,4-Pyridinedicarboxylic acid, 6-(diethylamino)-2-methoxy-, dimethyl ester (CAS)

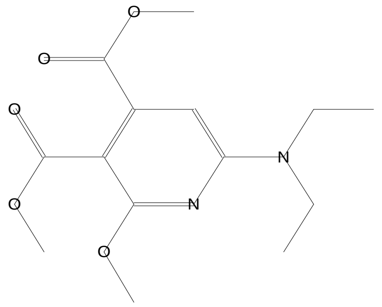

There is no signature data to report.

Library Search Report

RT	Probability	Compound Name	SI	Area %	Area	Molecular Weight	Molecular Formula	Library
11.64	42.35	1,2-Dihydro-1,4-diphenylphthalazine	477	0.50	1601053.00	284	C20H16N2	Wiley9
11.64	12.46	trans-12-Azido-1,2,11,12-tetrahydro-3-methyl-11-benz[j]acenthrylenol acetate	450	0.50	1601053.00	369	C23H19N3O2	Wiley9
11.64	11.97	2-[3'-(t-Butyldimethyldimethylsilyloxy)propyl]-1,4-bis(trimethylsilyloxy)benzene	449	0.50	1601053.00	626	C21H242O3Si3	Wiley9

Faten-212 #1951 RT: 11.64 AV: 1 RF: 6.00, 3 NL: 1.91E4
F: {0,0} + c EI Full ms [40.00-800.00]

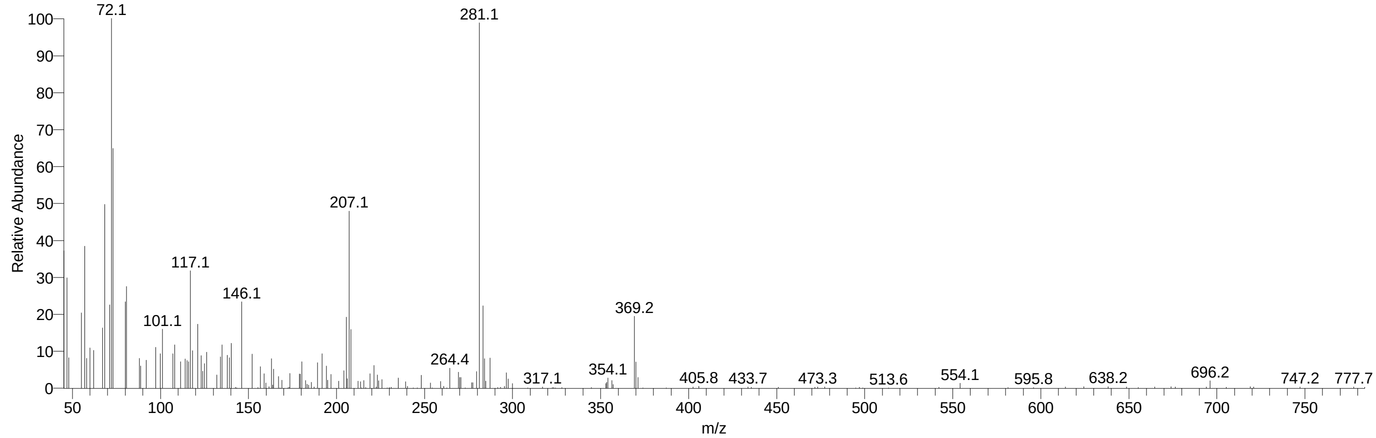

Hit Spectrum	Delta	Compound Structure

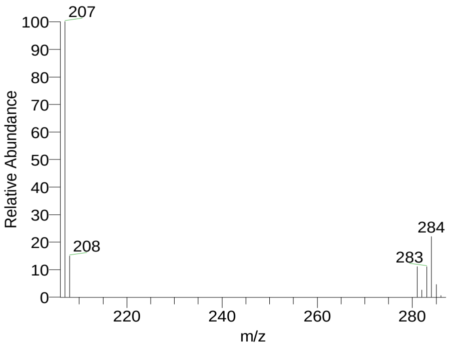

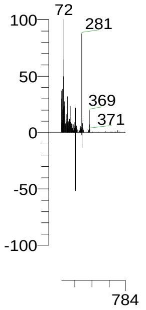

1,2-Dihydro-1,4-diphenylphthalazine
Formula C20H16N2, MW 284, CAS# 106200-50-4, Entry# 340135
Phthalazine, 1,2-dihydro-1,4-diphenyl- (CAS)

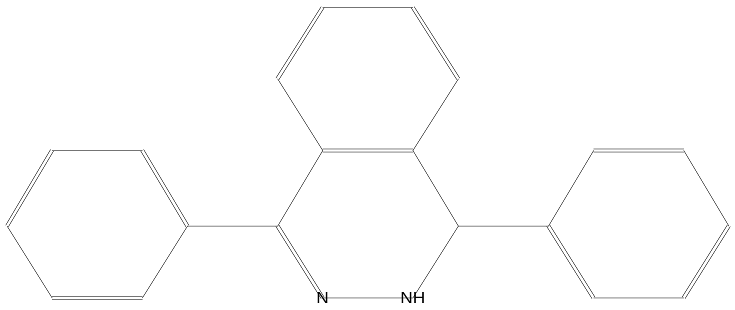

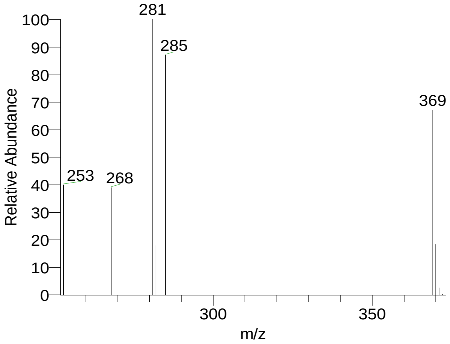

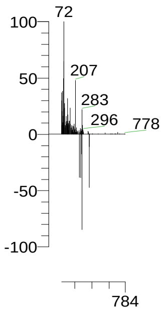

trans-12-Azido-1,2,11,12-tetrahydro-3-methyl-11-benz[j]acenthrylenol acetate
Formula C23H19N3O2, MW 369, CAS# NA, Entry# 501965

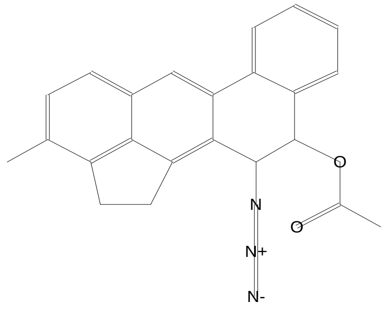

There is no signature data to report.

Library Search Report

Hit Spectrum	Delta	Compound Structure

2-[3'-(t-Butyldimethyldimethylsilyloxy)propyl]-1,4-bis(trimethylsilyloxy)benzene
Formula C21H242O3Si3, MW 626, CAS# NA, Entry# 647981

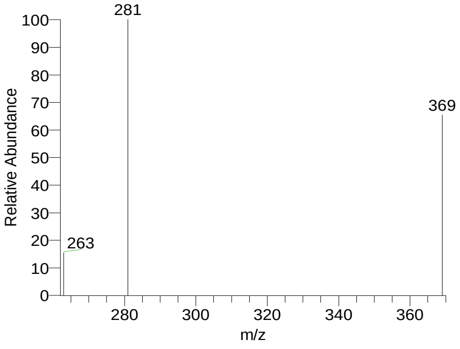

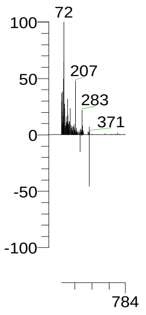

There is no signature data to report.

Library Search Report

RT	Probability	Compound Name	SI	Area %	Area	Molecular Weight	Molecular Formula	Library
11.87	10.56	1-(p-Chlorotetrafluorophenyl)-2-trimethylsilylacetylene	393	0.17	531428.00	280	C11H9ClF4Si	Wiley9
11.87	8.92	5-Amino-7-bromo-8-cyano-4-methyl-3,4-dihydro-1,6-naphthyridin-2(1H)-one	389	0.17	531428.00	280	C10H9BrN4O	Wiley9
11.87	8.57	2-chloro-8-hydroxy-7-isopropyl-6-methoxy-1,4-naphthoquinone	388	0.17	531428.00	280	C14H13ClO4	Wiley9

Faten-212 #2021 RT: 11.87 AV: 1 RF: 6.00, 3 NL: 7.13E4
F: {0,0} + c EI Full ms [40.00-800.00]

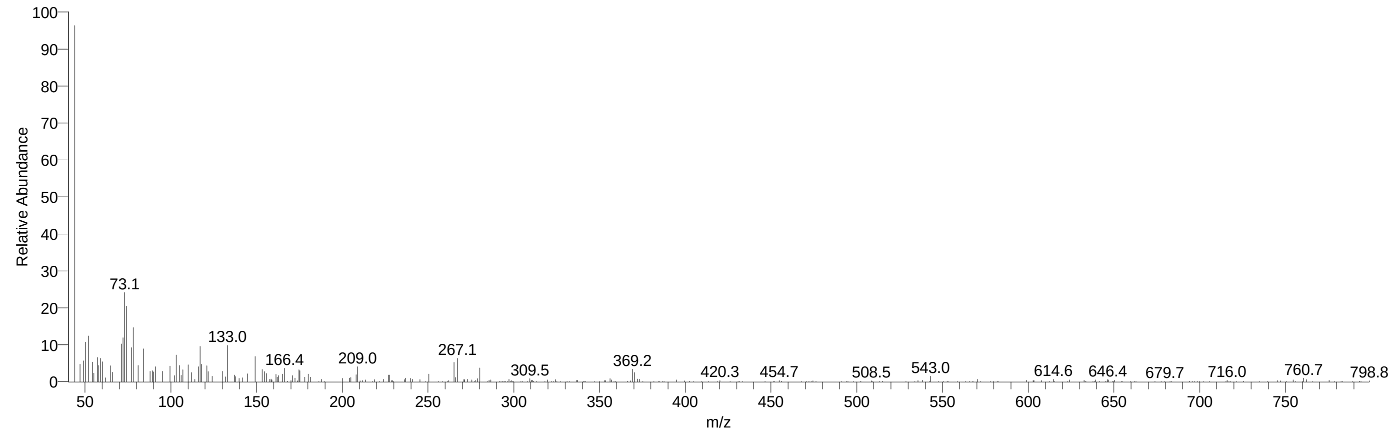

Hit Spectrum	Delta	Compound Structure

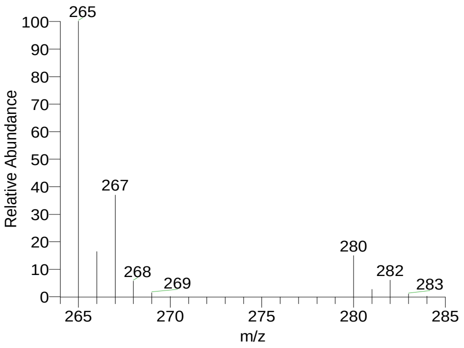

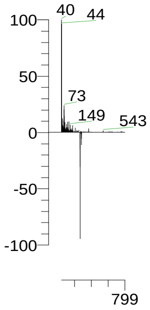

1-(p-Chlorotetrafluorophenyl)-2-trimethylsilylacetylene
Formula C11H9ClF4Si, MW 280, CAS# NA, Entry# 328090

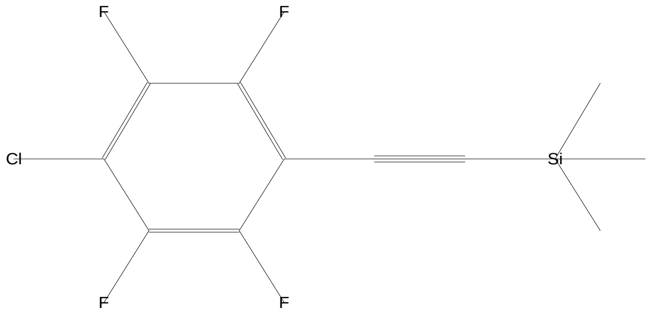

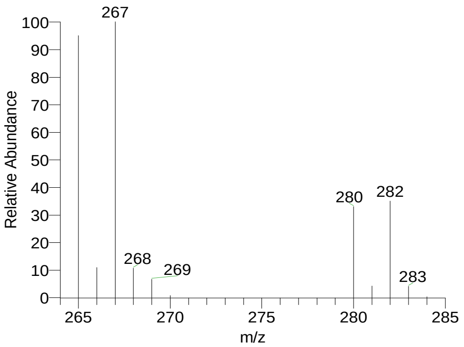

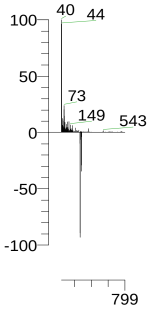

5-Amino-7-bromo-8-cyano-4-methyl-3,4-dihydro-1,6-naphthyridin-2(1H)-one
Formula C10H9BrN4O, MW 280, CAS# NA, Entry# 328014

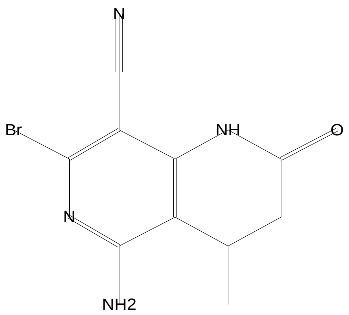

There is no signature data to report.

Library Search Report

Hit Spectrum	Delta	Compound Structure

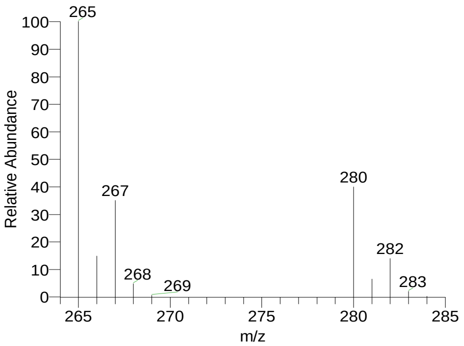

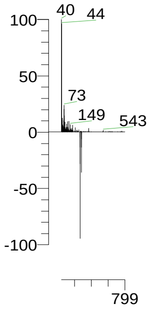

2-chloro-8-hydroxy-7-isopropyl-6-methoxy-1,4-naphthoquinone
Formula C14H13ClO4, MW 280, CAS# NA, Entry# 328607

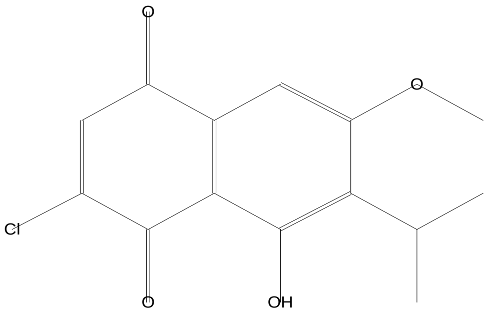

There is no signature data to report.

Library Search Report

RT	Probability	Compound Name	SI	Area %	Area	Molecular Weight	Molecular Formula	Library
12.10	6.47	Pyrazole[4,5-b]imidazole, 1-formyl-3-ethyl-6-á-d-ribofuranosyl-	407	0.18	570728.42	296	C12H16N4O5	mainlib
12.10	5.96	1-(2-trimethylsiloxy-1,1-dideuteriovinyl)-4-trimethylsiloxy-benzene	405	0.18	570728.42	280	C14H22D2O2Si2	Wiley9
12.10	5.96	Chromone, 5-hydroxy-6,7,8-trimethoxy-2,3-dimethyl-	405	0.18	570728.42	280	C14H16O6	mainlib

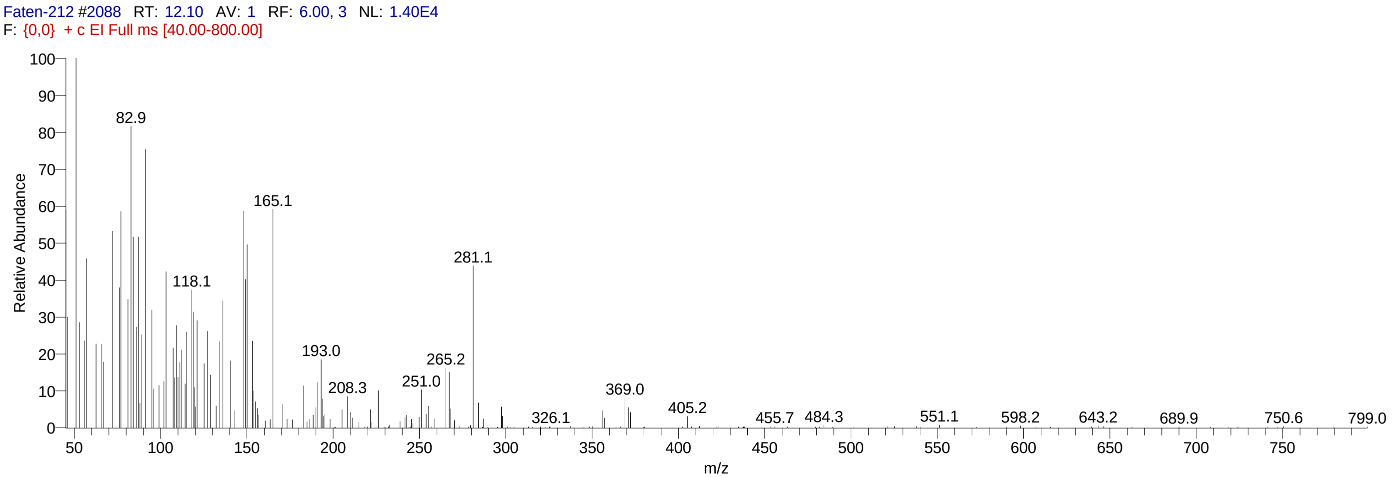

Hit Spectrum	Delta	Compound Structure

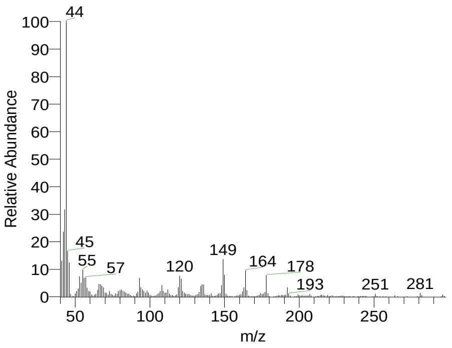

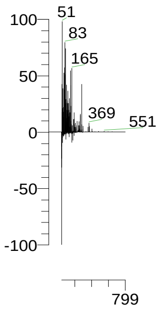

Pyrazole[4,5-b]imidazole, 1-formyl-3-ethyl-6-á-d-ribofuranosyl-
Formula C12H16N4O5, MW 296, CAS# NA, Entry# 14403

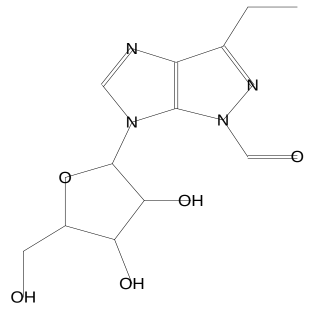

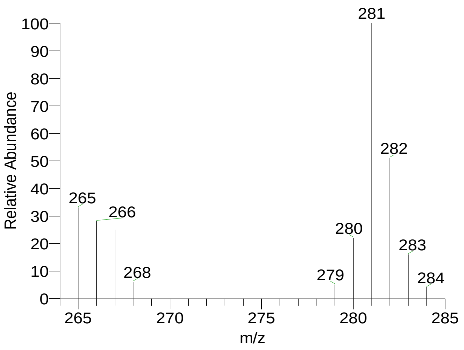

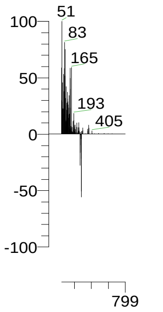

1-(2-trimethylsiloxy-1,1-dideuteriovinyl)-4-trimethylsiloxy-benzene
Formula C14H22D2O2Si2, MW 280, CAS# 126210-55-7, Entry# 328880
Silane, trimethyl[4-[1-[(trimethylsilyl)oxy]ethenyl-2,2-d2]phenoxy]- (CAS)

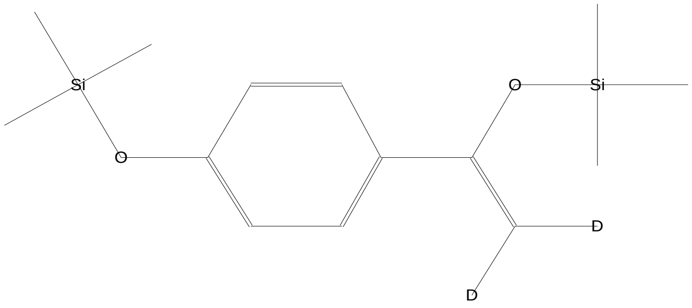

There is no signature data to report.

Library Search Report

Hit Spectrum	Delta	Compound Structure

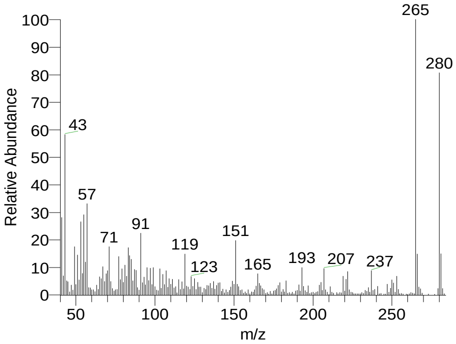

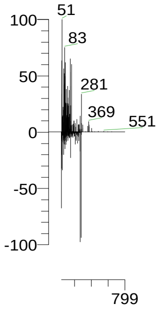

Chromone, 5-hydroxy-6,7,8-trimethoxy-2,3-dimethyl-
Formula C14H16O6, MW 280, CAS# NA, Entry# 189230
5-Hydroxy-6,7,8-trimethoxy-2,3-dimethyl-4H-chromen-4-one #

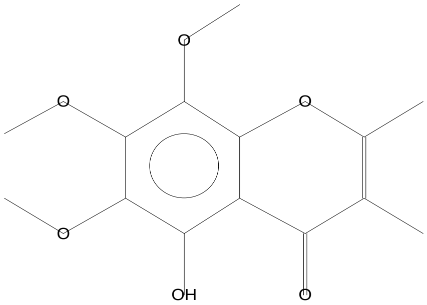

There is no signature data to report.

Library Search Report

RT	Probability	Compound Name	SI	Area %	Area	Molecular Weight	Molecular Formula	Library
12.27	19.06	2-Trifluoromethyl-3-one-buthanoic acid, ethyl ester	462	0.32	1009979.88	198	C7H9F3O3	Wiley9
12.27	19.06	2-Trifluoromethyl-3-oxobutanoic acid, ethyl ester	459	0.32	1009979.88	198	C7H9F3O3	mainlib
12.27	12.31	Propane, 2-nitro- (CAS)	449	0.32	1009979.88	89	C3H7NO2	Wiley9

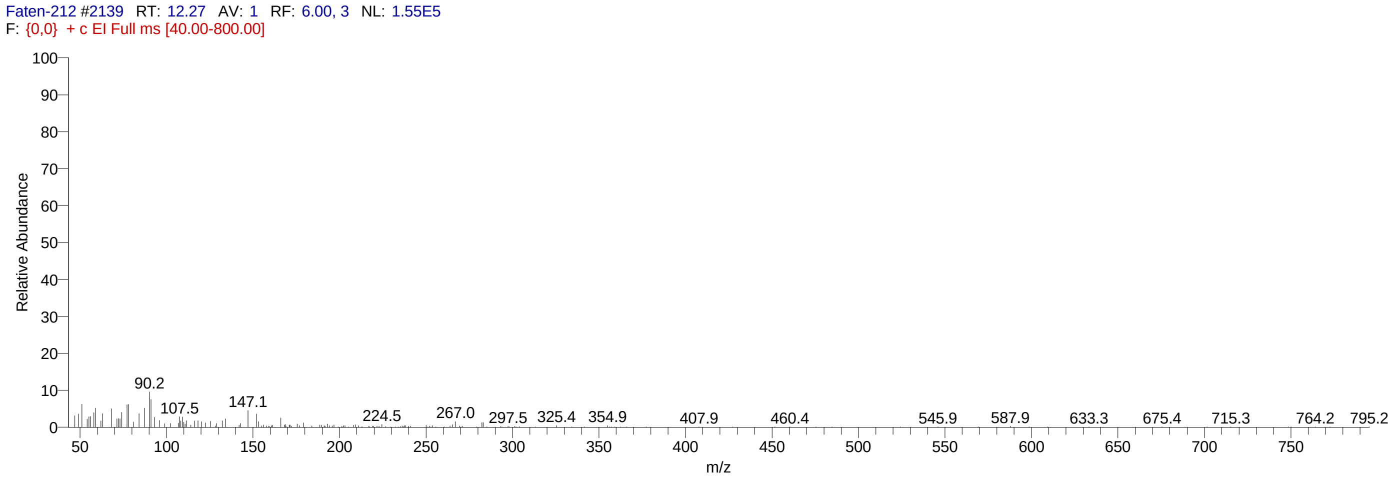

Hit Spectrum | Delta | Compound Structure

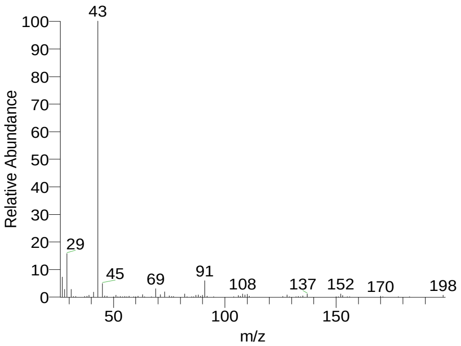

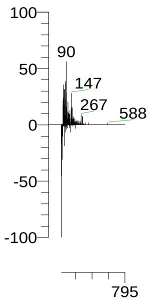

2-Trifluoromethyl-3-one-buthanoic acid, ethyl ester
Formula C7H9F3O3, MW 198, CAS# 116046-53-8, Entry# 137132
2-TRIFLUOROMETHYL-3-ONE,-BUTHANOIC ACID,ETHYL ESTER

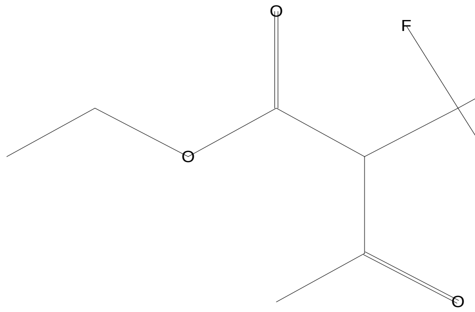

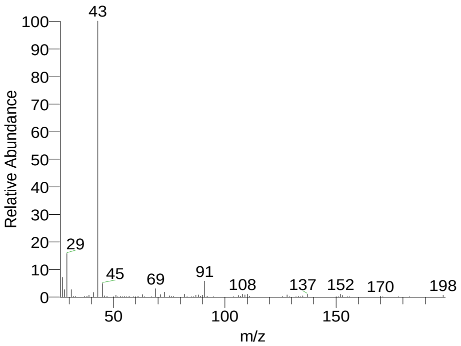

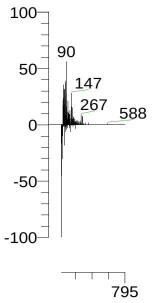

2-Trifluoromethyl-3-oxobutanoic acid, ethyl ester
Formula C7H9F3O3, MW 198, CAS# 116046-53-8, Entry# 5259
Ethyl 3-oxo-2-(trifluoromethyl)butanoate #

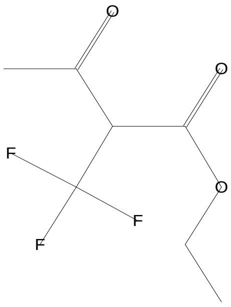

There is no signature data to report.

Library Search Report

Hit Spectrum

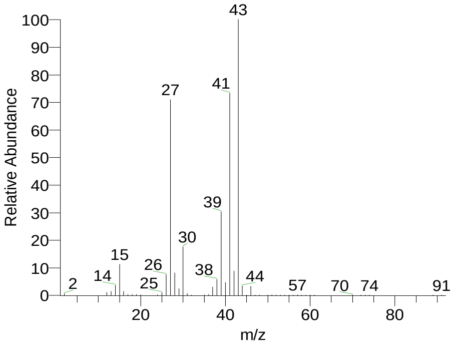

Delta

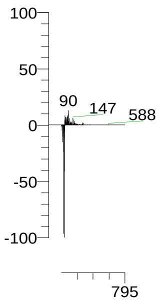

Compound Structure

Propane, 2-nitro- (CAS)
Formula C3H7NO2, MW 89, CAS# 79-46-9, Entry# 4906
2-Nitropropane

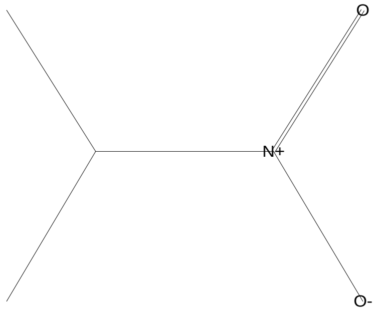

There is no signature data to report.

Library Search Report

RT	Probability	Compound Name	SI	Area %	Area	Molecular Weight	Molecular Formula	Library
12.49	8.96	à-N-Normethadol	417	0.16	504137.08	297	C20H27NO	mainlib
12.49	8.61	3-Heptanone, 6-(dimethylamino)-4,4-diphenyl- (CAS)	416	0.16	504137.08	309	C21H27NO	Wiley9
12.49	6.42	Thiourea, 1-[2-(2-benzylphenoxy)ethyl]-3-(O-tolyl)-	408	0.16	504137.08	376	C23H24N2OS	mainlib

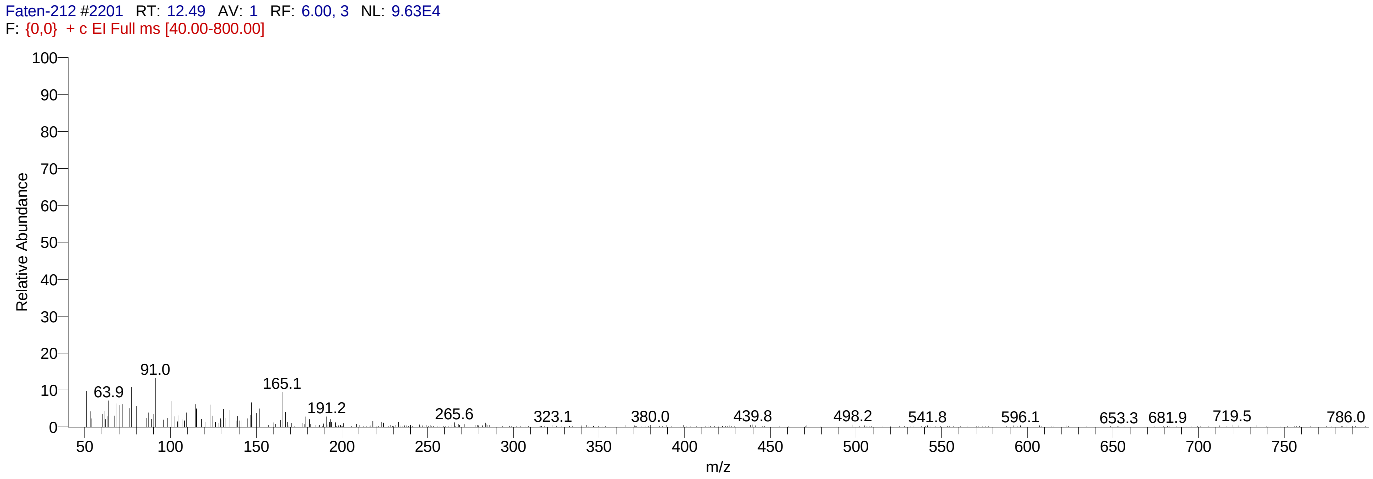

Hit Spectrum | Delta | Compound Structure

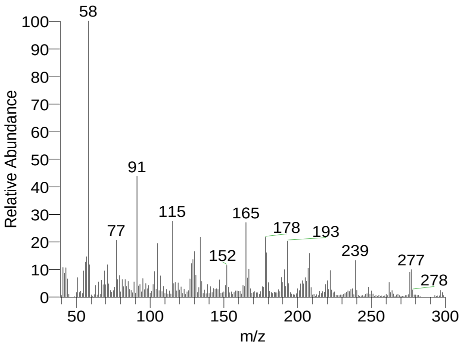

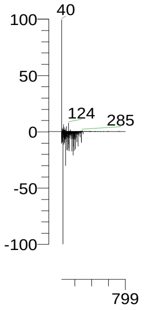

à-N-Normethadol
Formula C20H27NO, MW 297, CAS# 38455-85-5, Entry# 26410
6-(Methylamino)-4,4-diphenyl-3-heptanol #

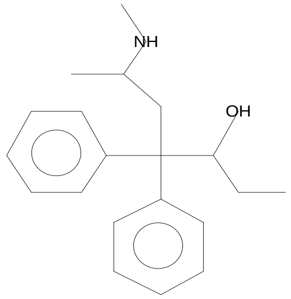

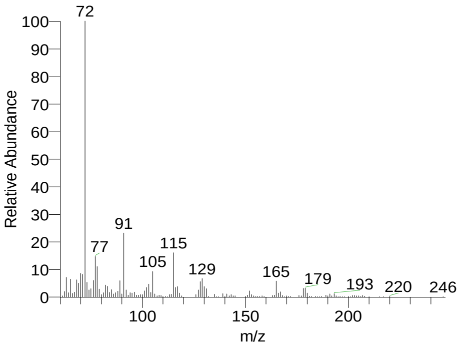

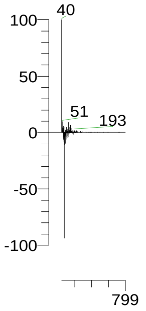

3-Heptanone, 6-(dimethylamino)-4,4-diphenyl- (CAS)
Formula C21H27NO, MW 309, CAS# 76-99-3, Entry# 394147
6-DIMETHYLAMINO-4,4-DIPHENYL-3-HEPTANONE

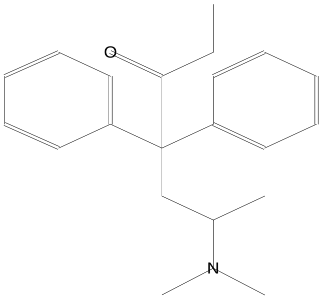

There is no signature data to report.

Library Search Report

Hit Spectrum	Delta	Compound Structure

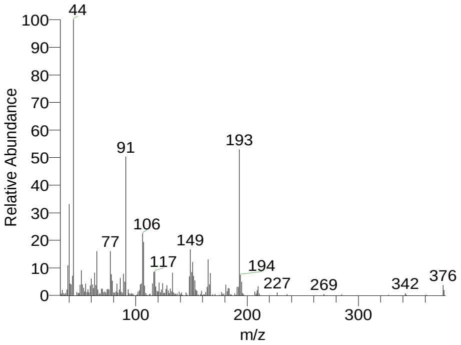

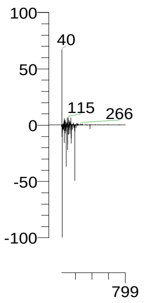

Thiourea, 1-[2-(2-benzylphenoxy)ethyl]-3-(O-tolyl)-
Formula C23H24N2OS, MW 376, CAS# NA, Entry# 15459
N-[2-(2-Benzylphenoxy)ethyl]-N'-(2-methylphenyl)thiourea #

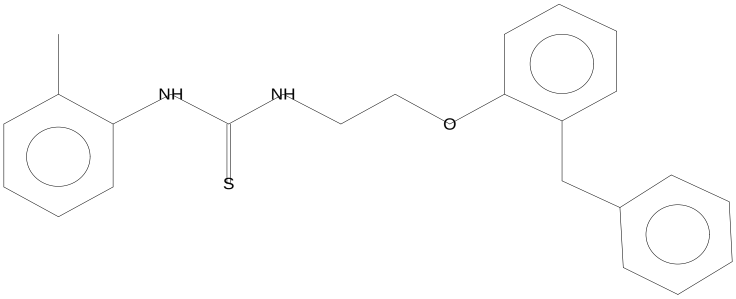

There is no signature data to report.

Library Search Report

RT	Probability	Compound Name	SI	Area %	Area	Molecular Weight	Molecular Formula	Library
12.84	18.21	GLYCOCHOLIC ACID METHYL ESTER TMS	377	0.23	719768.45	695	C36H69NO6Si3	Wiley9
12.84	18.21	Glycine, N-[(3à,5á,7à,12à)-24-oxo-3,7,12-tris[(trimethylsilyl)oxy]cholan-24-yl]-, methyl ester	376	0.23	719768.45	695	C36H69NO6Si3	mainlib
12.84	15.39	5-ETHOXYCARBONYL-5-METHYL-à,á,.DELTA.,ç-TETRAPHENYLNICKEL-5,6-HOMOPORPHYRINE	373	0.23	719768.45	770	C49H36N4NiO2	Wiley9

Faten-212 #2304 RT: 12.84 AV: 1 RF: 6.00, 3 NL: 9.81E4
F: {0,0} + c EI Full ms [40.00-800.00]

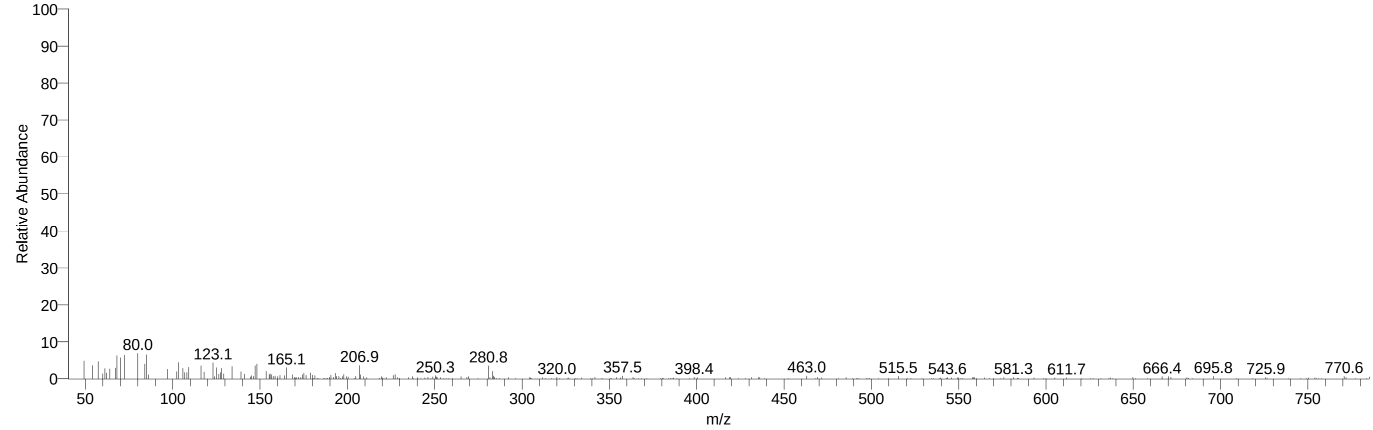

Hit Spectrum	Delta	Compound Structure

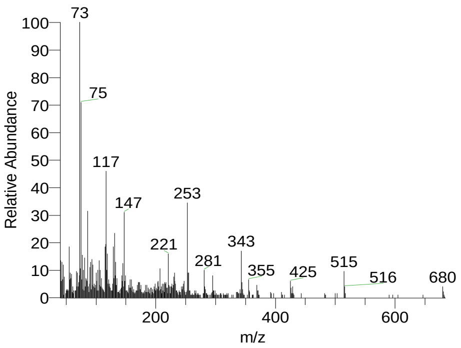

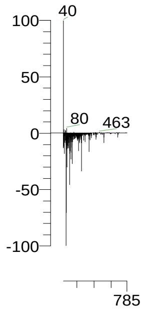

GLYCOCHOLIC ACID METHYL ESTER TMS
Formula C36H69NO6Si3, MW 695, CAS# 57326-16-6, Entry# 654329
Glycine, N-[(3à,5á,7à,12à)-24-oxo-3,7,12-tris[(trimethylsilyl)oxy]cholan-24-yl]-, methyl ester (CAS)

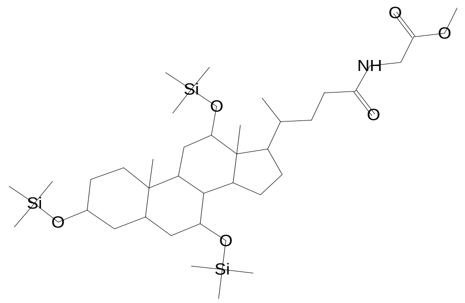

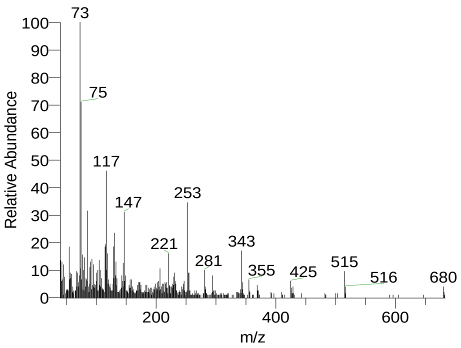

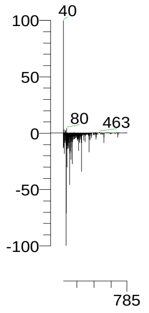

Glycine, N-[(3à,5á,7à,12à)-24-oxo-3,7,12-tris[(trimethylsilyl)oxy]cholan-24-yl]-, methyl ester
Formula C36H69NO6Si3, MW 695, CAS# 57326-16-6, Entry# 37701
Methyl ((24-oxo-3,7,12-tris[(trimethylsilyl)oxy]cholan-24-yl)amino)acetate #

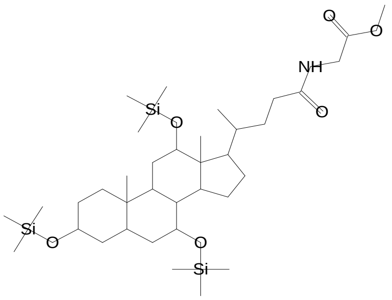

There is no signature data to report.

Library Search Report

Hit Spectrum	Delta	Compound Structure

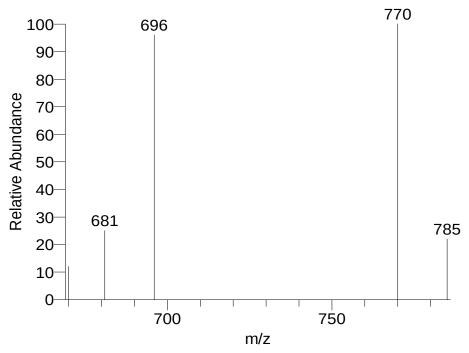

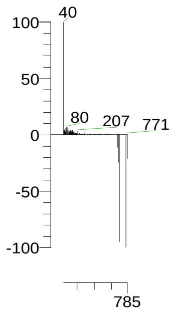

Formula C49H36N4NiO2, MW 770, CAS# 69811-91-2, Entry# 657868

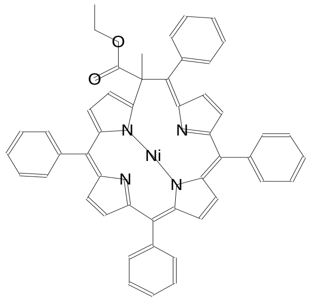

There is no signature data to report.

Library Search Report

RT	Probability	Compound Name	SI	Area %	Area	Molecular Weight	Molecular Formula	Library
13.63	29.33	Cathine (CAS)	478	0.19	594278.18	151	C9H13NO	Wiley9
13.63	14.24	(-)-Norephedrine	459	0.19	594278.18	151	C9H13NO	mainlib
13.63	14.24	Benzenemethanol, à-(1-aminoethyl)-, [R-(R*,S*)]- (CAS)	459	0.19	594278.18	151	C9H13NO	Wiley9

Faten-212 #2538 RT: 13.63 AV: 1 RF: 6.00, 3 NL: 1.44E5
F: {0,0} + c EI Full ms [40.00-800.00]

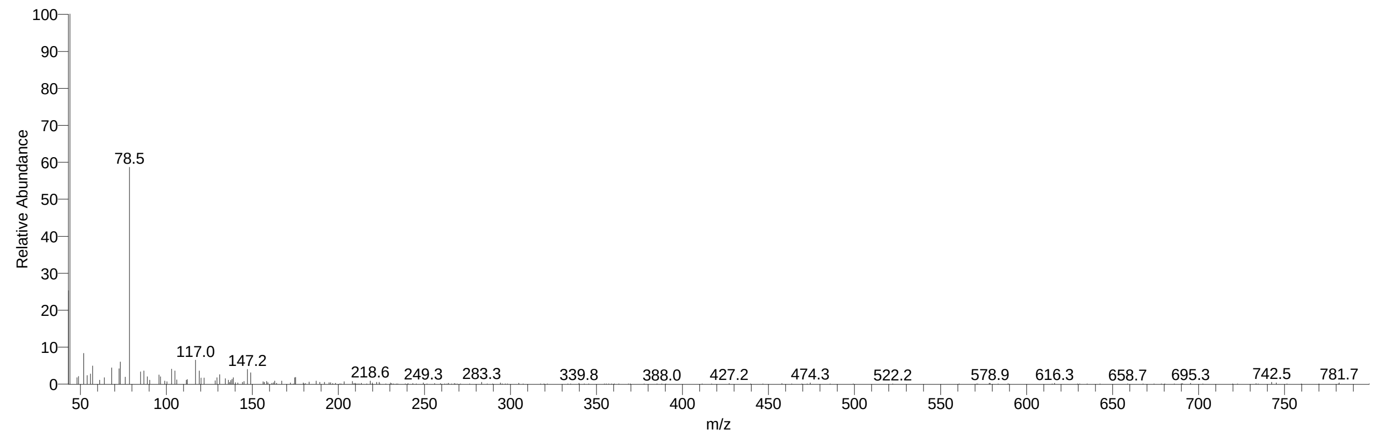

Hit Spectrum	Delta	Compound Structure

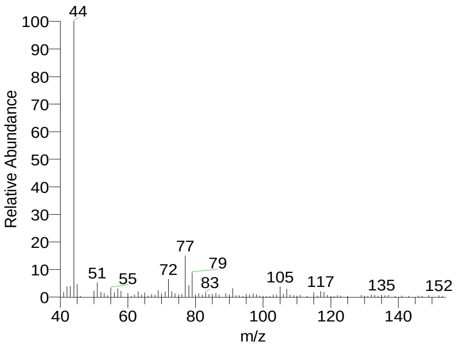

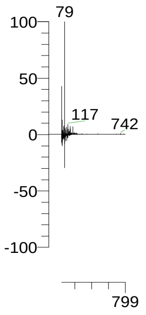

Cathine (CAS)
Formula C9H13NO, MW 151, CAS# 492-39-7, Entry# 52366
Benzenemethanol, à-(1-aminoethyl)-, [S-(R@,R@)]-

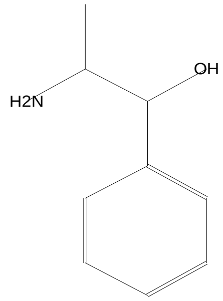

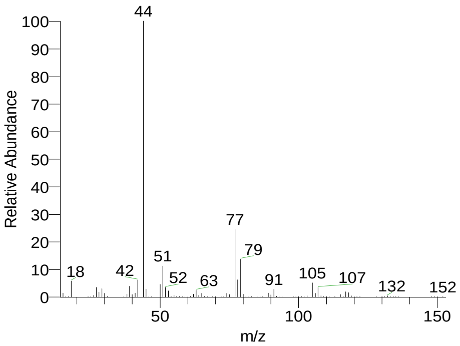

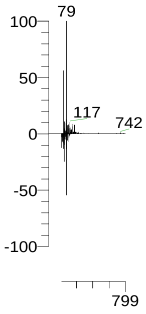

(-)-Norephedrine
Formula C9H13NO, MW 151, CAS# 492-41-1, Entry# 14851
Benzenemethanol, à-(1-aminoethyl)-, [R-(R*,S*)]-

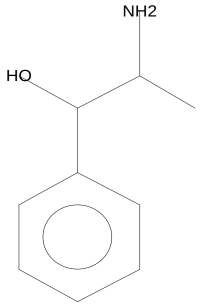

There is no signature data to report.

Library Search Report

Hit Spectrum	Delta	Compound Structure

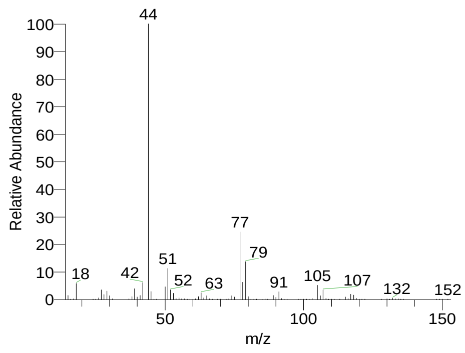

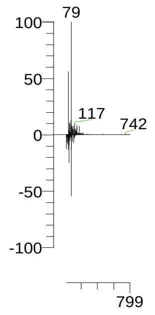

Benzenemethanol, à-(1-aminoethyl)-, [R-(R*,S*)]- (CAS)
Formula C9H13NO, MW 151, CAS# 492-41-1, Entry# 52273
NOREPHEDRINE

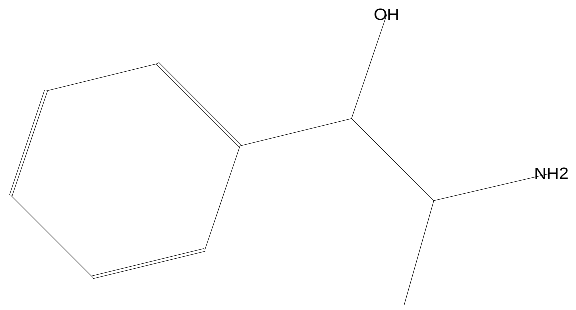

There is no signature data to report.

Library Search Report

RT	Probability	Compound Name	SI	Area %	Area	Molecular Weight	Molecular Formula	Library
15.22	16.08	10aH-2,12a-Methano-1H,4H-cyclopropa[5,6][1,3]dioxolo[2',3']cyclopenta[1',2':9,10]cyclodeca[1,2-d][1,3]dioxin-15-ol, 1a,2,7a,13,14,14a-hexahydro-1,1,6,6,9,9,11,13-octamethyl-, [1aR-(1aà,2à,7aà,7bR*,10aà,12aà,13à,14aà,15S*)]-	361	0.17	534503.25	430	C26H38O5	Wiley9
15.22	13.58	Octadecane, 3-ethyl-5-(2-ethylbutyl)-	357	0.17	534503.25	366	C26H54	mainlib
15.22	13.58	Octadecane, 3-ethyl-5-(2-ethylbutyl)- (CAS)	357	0.17	534503.25	366	C26H54	Wiley9

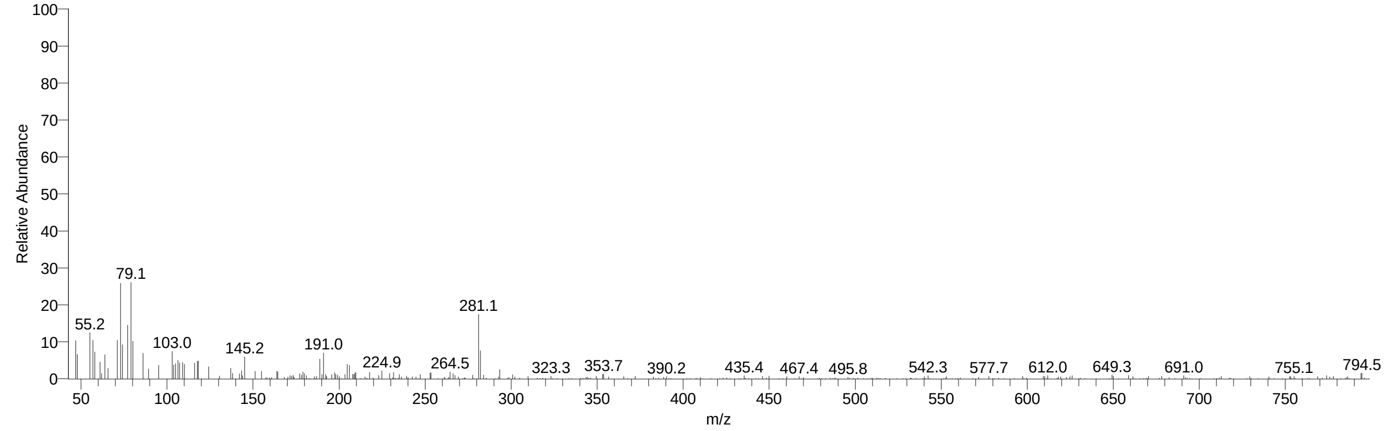

Hit Spectrum	Delta	Compound Structure

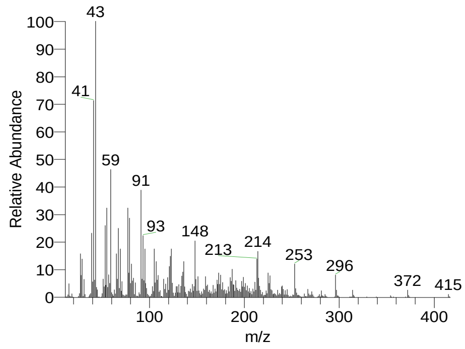

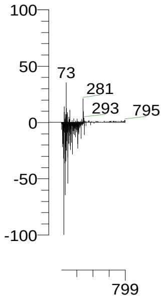

Formula C26H38O5, MW 430, CAS# 77573-45-6, Entry# 573816

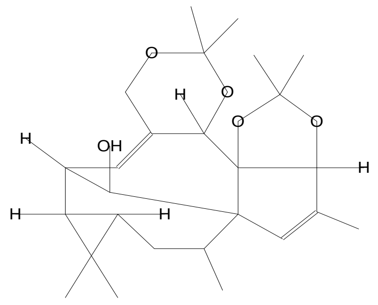

There is no signature data to report.

Library Search Report

Hit Spectrum	Delta	Compound Structure

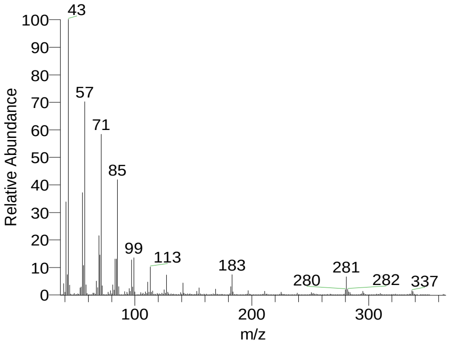

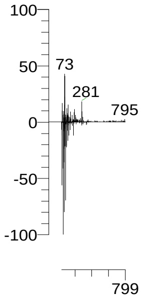

Octadecane, 3-ethyl-5-(2-ethylbutyl)-
Formula C26H54, MW 366, CAS# 55282-12-7, Entry# 7471
3-Ethyl-5-(2'-ethylbutyl)octadecane

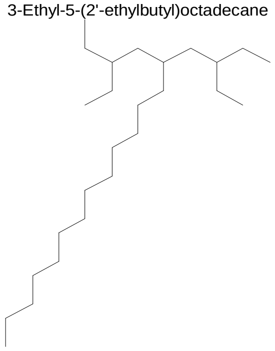

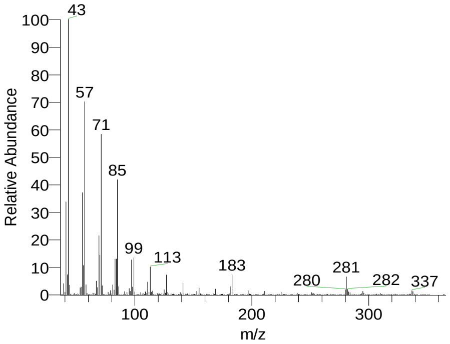

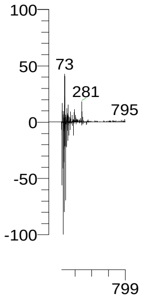

Octadecane, 3-ethyl-5-(2-ethylbutyl)- (CAS)
Formula C26H54, MW 366, CAS# 55282-12-7, Entry# 497991
3-Ethyl-5-(2'-ethylbutyl)octadecane

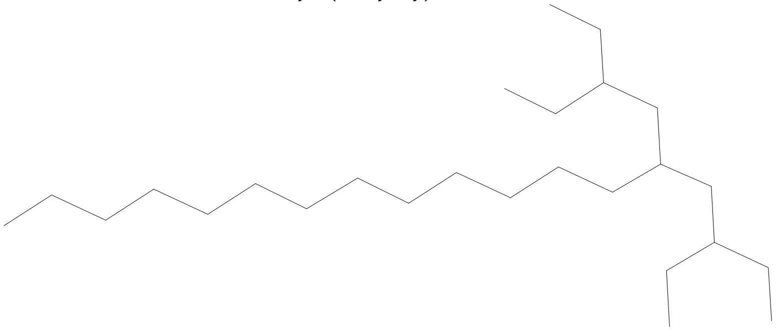

There is no signature data to report.

Library Search Report

RT	Probability	Compound Name	SI	Area %	Area	Molecular Weight	Molecular Formula	Library
15.57	90.55	1,4,7-Tris(3,5-di-tert-butyl-2-deuteriohydroxybenzyl)-1,4,7-triazacyclononane	659	0.26	824685.55	783	C51H72D9N3O3	Wiley9
15.57	0.90	Cystathionine-diTMS	440	0.26	824685.55	366	C13H30N2O4SSi2	Wiley9
15.57	0.80	4-(5-Pentyl-3a,4,5,7a-tetrahydro-4-indanyl)butanoicacid,methyl ester(stereoisomer 1)	437	0.26	824685.55	292	C19H32O2	mainlib

Faten-212 #3107 RT: 15.57 AV: 1 RF: 6.00, 3 NL: 1.00E5
F: {0,0} + c EI Full ms [40.00-800.00]

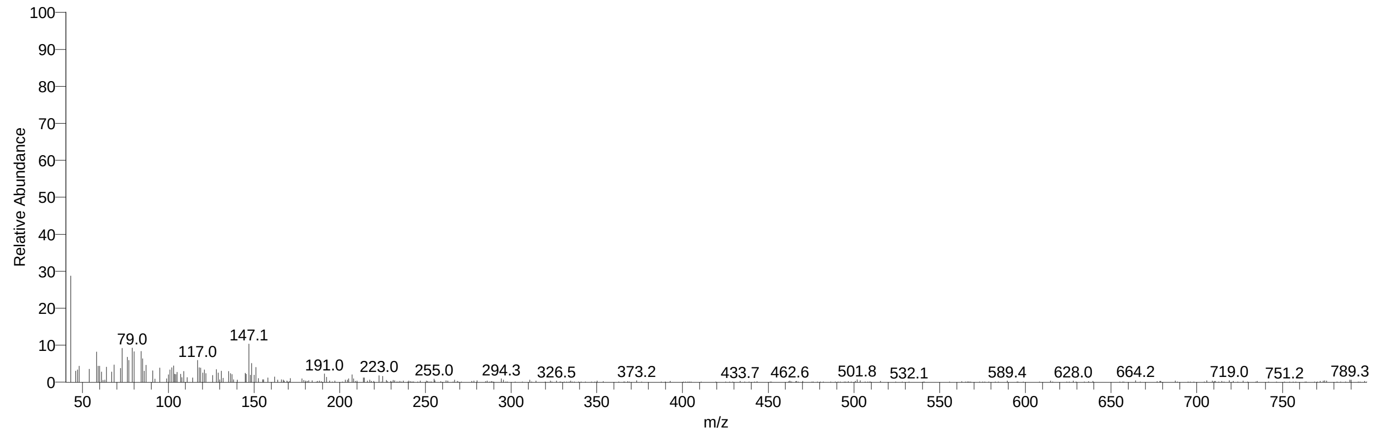

Hit Spectrum | Delta | Compound Structure

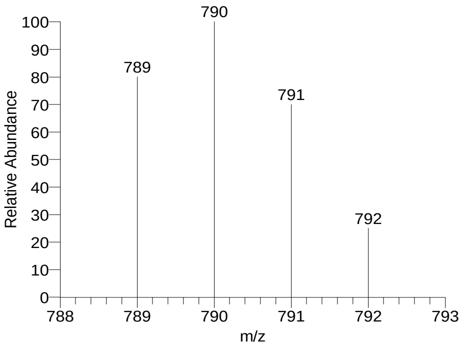

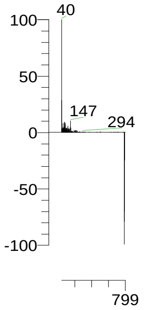

1,4,7-Tris(3,5-di-tert-butyl-2-deuteriohydroxybenzyl)-1,4,7-triazacyclononane
Formula C51H72D9N3O3, MW 783, CAS# NA, Entry# 658265

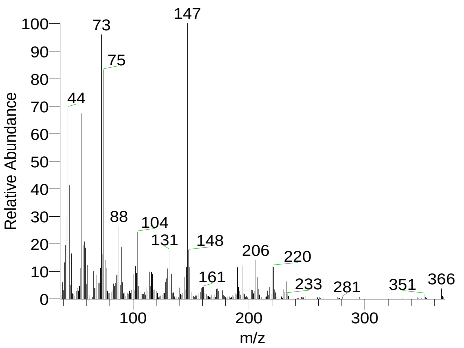

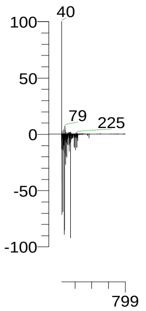

Cystathionine-diTMS
Formula C13H30N2O4SSi2, MW 366, CAS# 73090-79-6, Entry# 496338
Cystathionine, bis(triemthylsilyl) ester

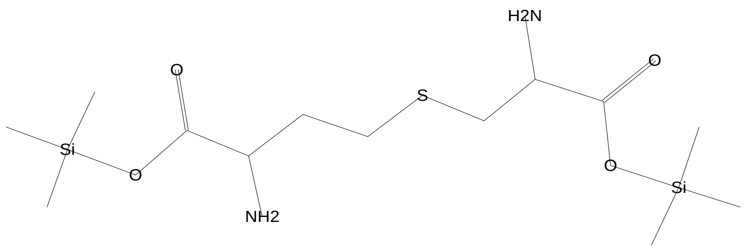

There is no signature data to report.

Library Search Report

Hit Spectrum	Delta	Compound Structure

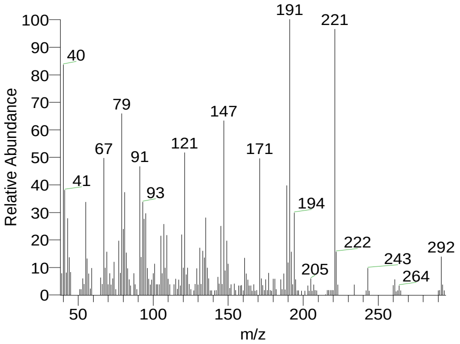

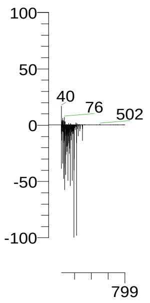

4-(5-Pentyl-3a,4,5,7a-tetrahydro-4-indanyl)butanoicacid,methyl ester(stereoisomer 1)
Formula C19H32O2, MW 292, CAS# NA, Entry# 157341
Methyl 4-(5-pentyl-2,3,3a,4,5,7a-hexahydro-1H-inden-4-yl)butanoate #

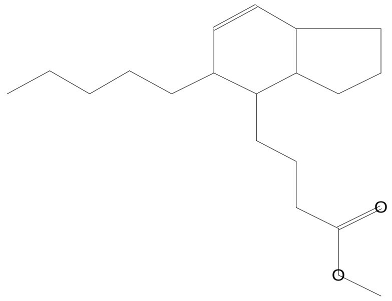

There is no signature data to report.

Library Search Report

RT	Probability	Compound Name	SI	Area %	Area	Molecular Weight	Molecular Formula	Library
16.79	15.89	1,2,3,4-Tetrahydroisoquinolin-6-ol-1-carboxylic acid	453	0.90	2844860.84	193	C10H11NO3	mainlib
16.79	6.76	3-Isopropoxyphthalide	432	0.90	2844860.84	192	C11H12O3	Wiley9
16.79	6.50	1,4-[13C]-1,2,3,4-Tetrahydro-5-naphthylamine	431	0.90	2844860.84	147	C10H13N	Wiley9

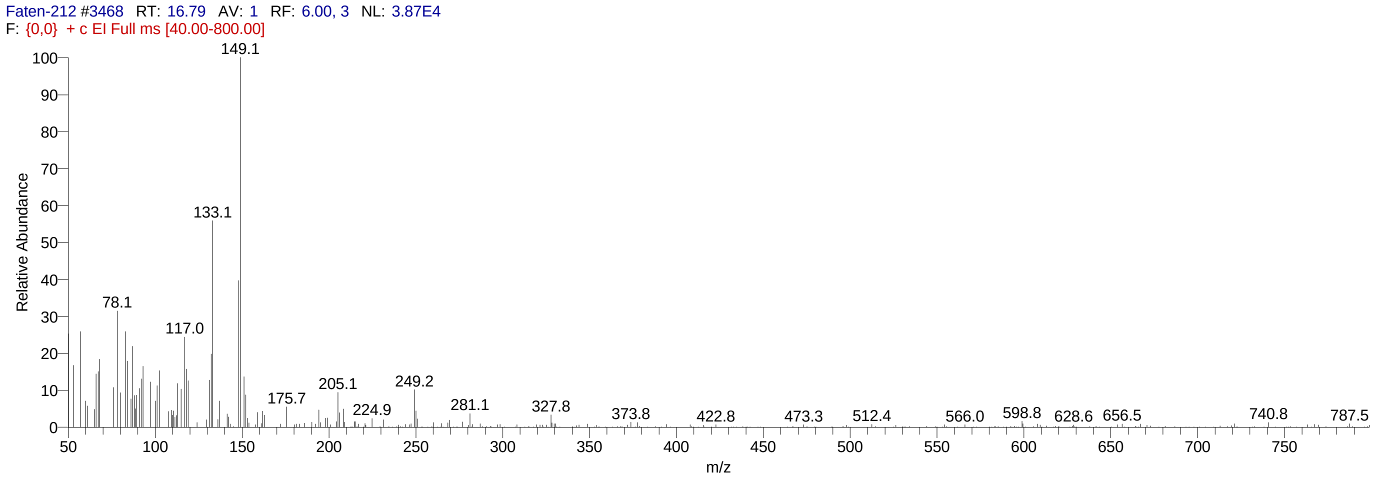

Hit Spectrum	Delta	Compound Structure

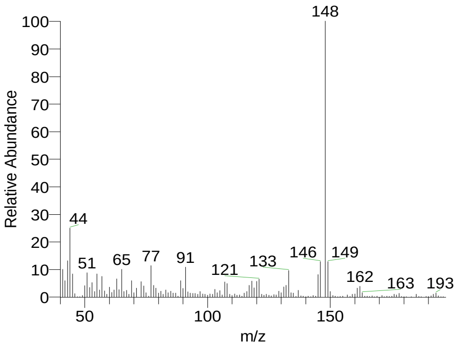

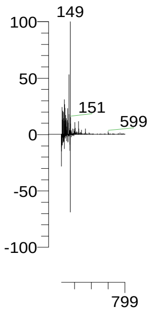

1,2,3,4-Tetrahydroisoquinolin-6-ol-1-carboxylic acid
Formula C10H11NO3, MW 193, CAS# 91523-50-1, Entry# 120283
6-Hydroxy-1,2,3,4-tetrahydro-1-isoquinolinecarboxylic acid #

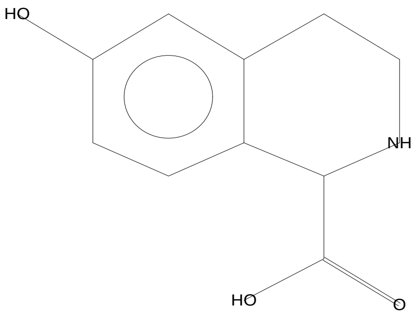

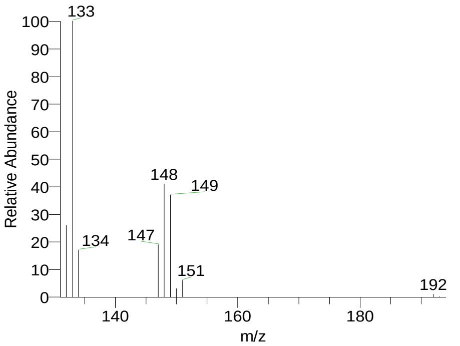

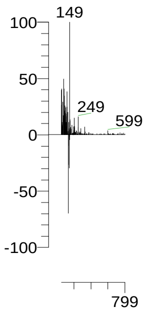

3-Isopropoxyphthalide
Formula C11H12O3, MW 192, CAS# NA, Entry# 124697

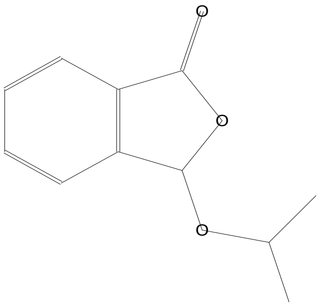

There is no signature data to report.

Library Search Report

Hit Spectrum	Delta	Compound Structure

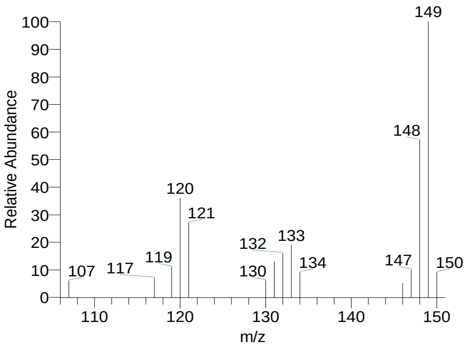

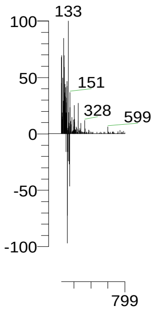

1,4-[13C]-1,2,3,4-Tetrahydro-5-naphthylamine
Formula C10H13N, MW 147, CAS# 67519-15-7, Entry# 47121
1-Naphthalenamine-5,8-13C2, 5,6,7,8-tetrahydro- (CAS)

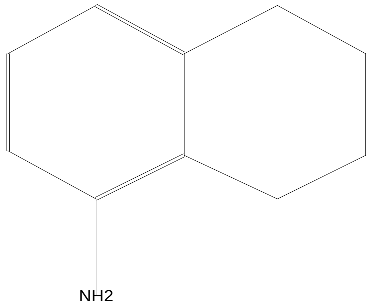

There is no signature data to report.

Library Search Report

RT	Probability	Compound Name	SI	Area %	Area	Molecular Weight	Molecular Formula	Library
17.15	13.17	Silanamine, N-[2-[3-methoxy-4-[(trimethylsilyl)oxy]phenyl]-N,1,1,1-tetramethyl-2-[(trimethylsilyl)oxy]ethyl]- (CAS)	495	0.32	1024227.87	413	C19H39NO3Si3	Wiley9
17.15	4.81	á-D-Glucopyranoside, methyl 2,3-bis-O-(trimethylsilyl)-, cyclic methylboronate (CAS)	472	0.32	1024227.87	362	C14H31BO6Si2	Wiley9
17.15	4.62	à-D-Mannopyranoside, methyl, cyclic 2,3:4,6-bis(butylboronate) (CAS)	471	0.32	1024227.87	326	C15H28B2O6	Wiley9

Faten-212 #3574 RT: 17.15 AV: 1 RF: 6.00, 3 NL: 9.37E4
F: {0,0} + c EI Full ms [40.00-800.00]

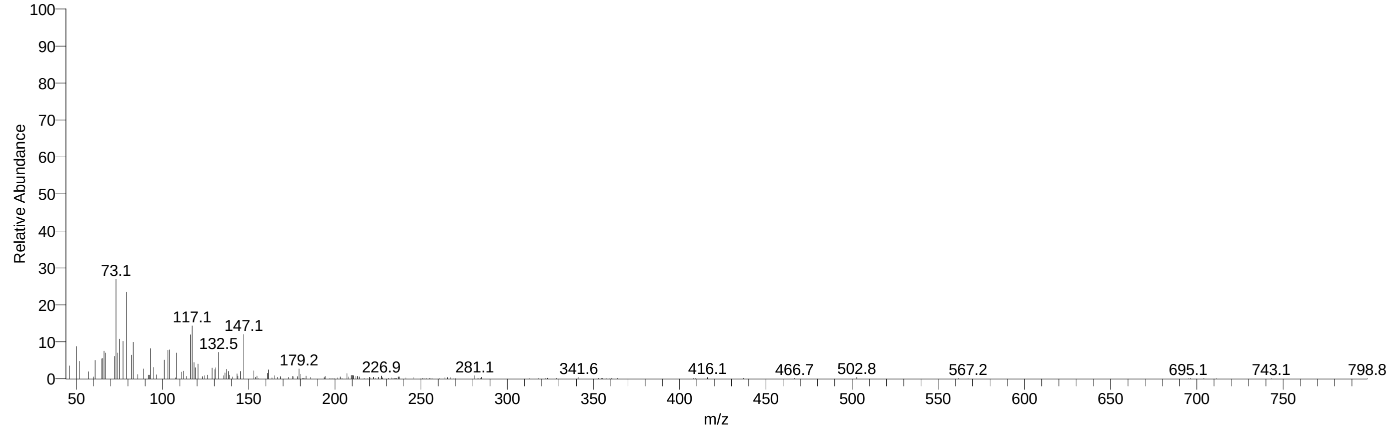

Hit Spectrum	Delta	Compound Structure

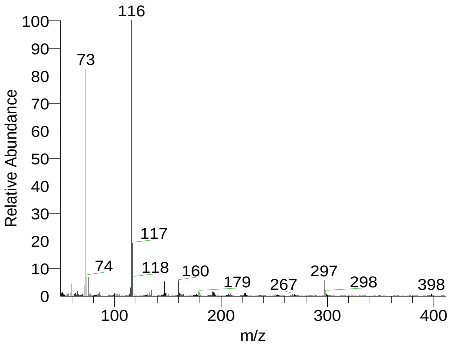

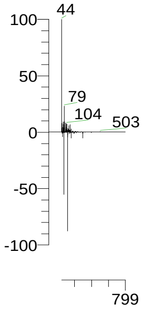

Formula C19H39NO3Si3, MW 413, CAS# 56114-63-7, Entry# 556550
METANEPHRINE-TRITMS

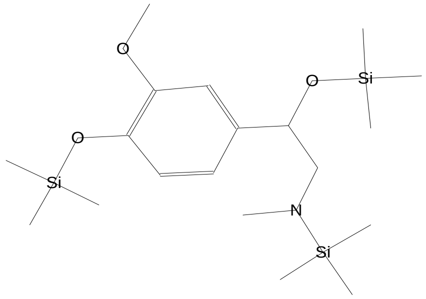

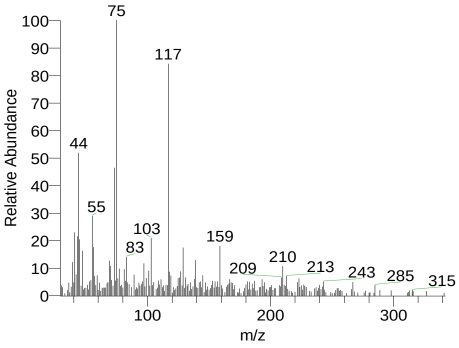

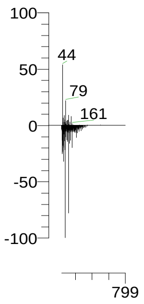

á-D-Glucopyranoside, methyl 2,3-bis-O-(trimethylsilyl)-, cyclic methylboronate (CAS)
Formula C14H31BO6Si2, MW 362, CAS# 56211-07-5, Entry# 490302
B-GLUCOPYRANOSIDE-1-METHYL-4,6-METHYLBORONATE-2,3-DITMS

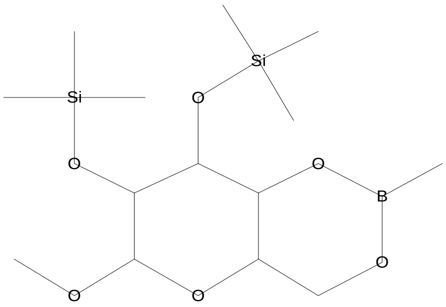

There is no signature data to report.

Library Search Report

Hit Spectrum | Delta | Compound Structure

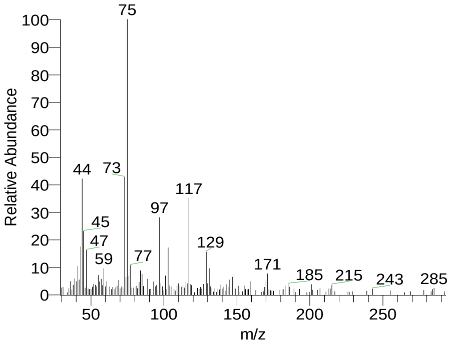

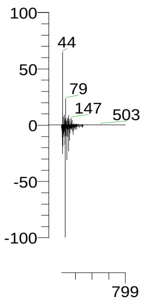

à-D-Mannopyranoside, methyl, cyclic 2,3:4,6-bis(butylboronate) (CAS)
Formula C15H28B2O6, MW 326, CAS# 54400-84-9, Entry# 427572
A-MANNOPYRANOSIDE-1-METHYL-2,3-4,6-DI-BUTYLBORONATE

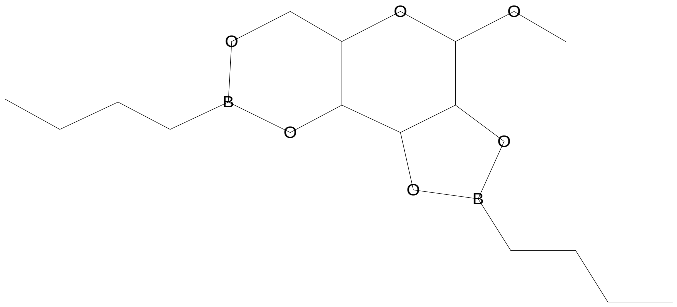

There is no signature data to report.

Library Search Report

RT	Probability	Compound Name	SI	Area %	Area	Molecular Weight	Molecular Formula	Library
17.91	6.42	Tetraacetyl-d-xylonic nitrile	425	0.19	597830.44	343	C14H17NO9	mainlib
17.91	4.92	2-Hexadecanol (CAS)	418	0.19	597830.44	242	C16H34O	Wiley9
17.91	3.86	1-[2-Nitro-5-chlorophenyl]-1-buten-3-ol	412	0.19	597830.44	227	C10H10ClNO3	mainlib

Faten-212 #3796 RT: 17.91 AV: 1 RF: 6.00, 3 NL: 7.92E4
F: {0,0} + c EI Full ms [40.00-800.00]

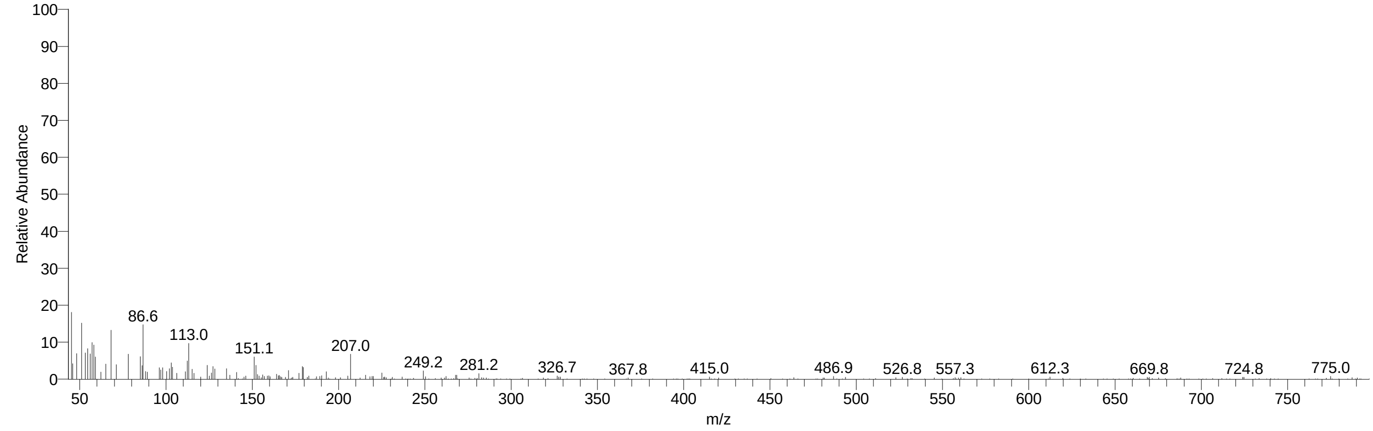

Hit Spectrum | Delta | Compound Structure

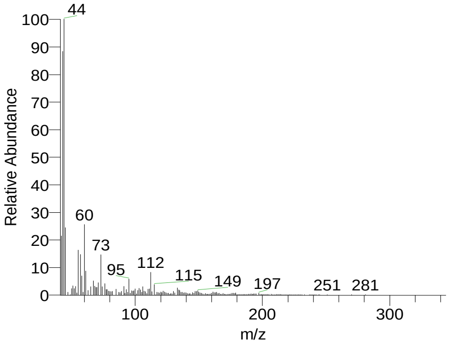

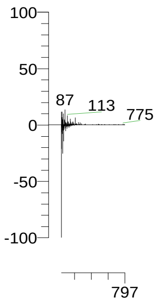

Tetraacetyl-d-xylonic nitrile
Formula C14H17NO9, MW 343, CAS# NA, Entry# 14445

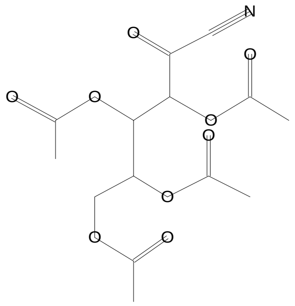

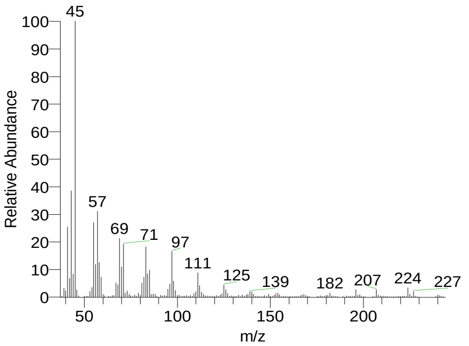

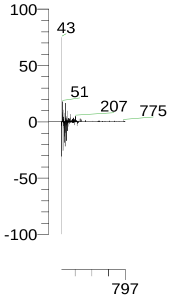

2-Hexadecanol (CAS)
Formula C16H34O, MW 242, CAS# 14852-31-4, Entry# 242086
Hexadecanol-2

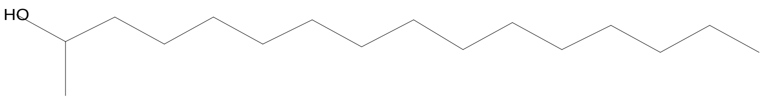

There is no signature data to report.

Library Search Report

Hit Spectrum	Delta	Compound Structure

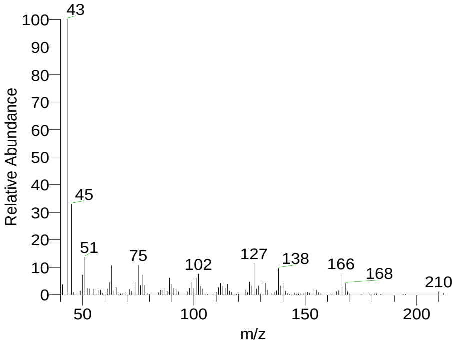

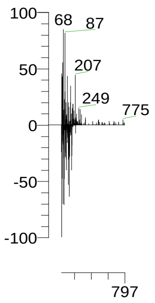

1-[2-Nitro-5-chlorophenyl]-1-buten-3-ol
Formula C10H10ClNO3, MW 227, CAS# NA, Entry# 6494
(3E)-4-(5-Chloro-2-nitrophenyl)-3-buten-2-ol #

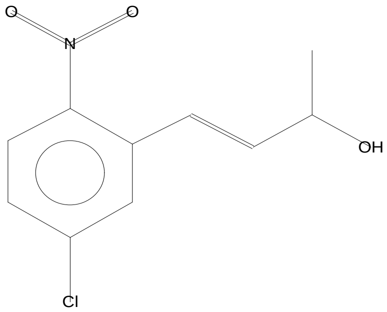

There is no signature data to report.

Library Search Report

RT	Probability	Compound Name	SI	Area %	Area	Molecular Weight	Molecular Formula	Library
18.03	3.59	2-[2-[2-(Carboxymethyloxy)ethoxy]ethoxy]acetic acid, bis(trimethylsilyl) ester	444	0.37	1168927.66	322	C12H26O6Si2	mainlib
18.03	3.31	2,2,4,7-Tetramethyl-3,6,9-trioxa-2-siladecane	442	0.37	1168927.66	220	C10H24O3Si	mainlib
18.03	2.92	2-[2-[2-[2-(Carboxymethyloxy)ethoxy]ethoxy]ethoxy]acetic acid, bis(trimethylsilyl) ester	439	0.37	1168927.66	366	C14H30O7Si2	mainlib

Faten-212 #3832 RT: 18.03 AV: 1 RF: 6.00, 3 NL: 9.48E4
F: {0,0} + c EI Full ms [40.00-800.00]

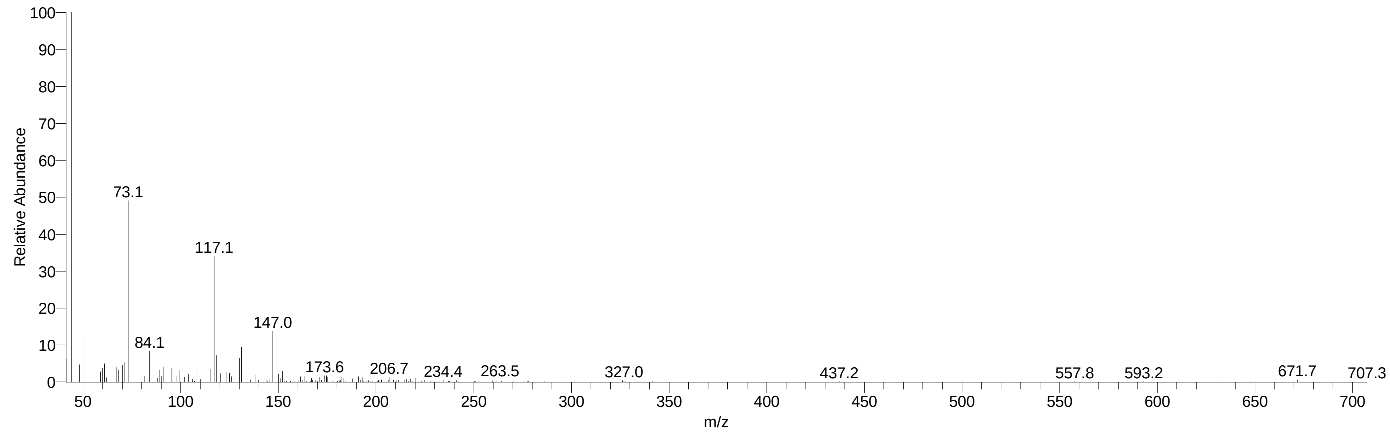

Hit Spectrum | Delta | Compound Structure

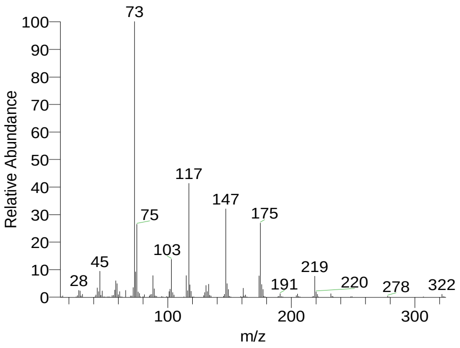

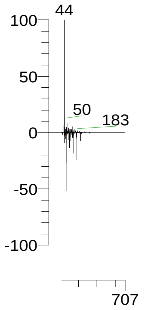

2-[2-[2-(Carboxymethyloxy)ethoxy]ethoxy]acetic acid, bis(trimethylsilyl) ester
Formula C12H26O6Si2, MW 322, CAS# NA, Entry# 38427
3,6,9-Trioxaundecanedioic acid, bis(trimethylsilyl) ester

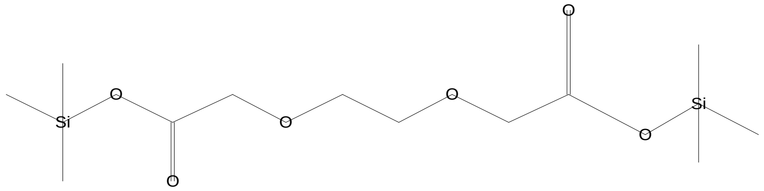

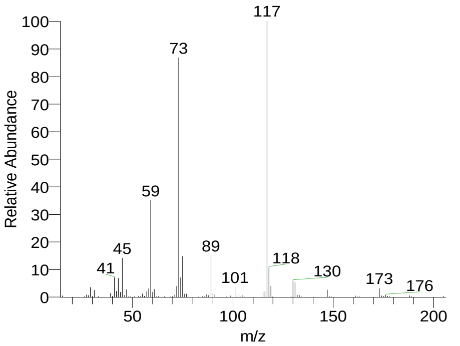

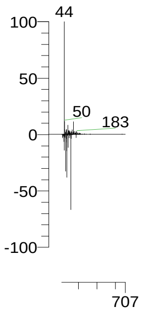

2,2,4,7-Tetramethyl-3,6,9-trioxa-2-siladecane
Formula C10H24O3Si, MW 220, CAS# NA, Entry# 86772

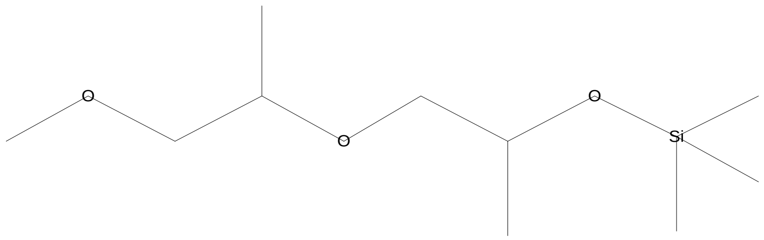

There is no signature data to report.

Library Search Report

Hit Spectrum	Delta	Compound Structure

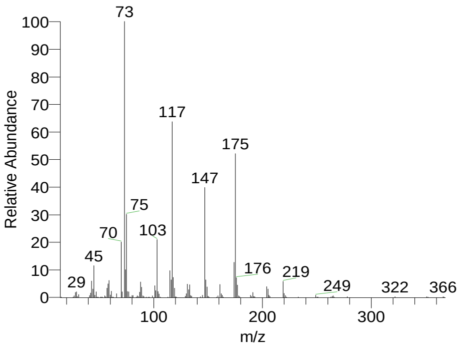

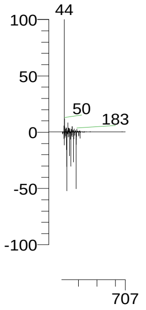

2-[2-[2-(Carboxymethyloxy)ethoxy]ethoxy]acetic acid, bis(trimethylsilyl) ester
Formula C14H30O7Si2, MW 366, CAS# NA, Entry# 38444

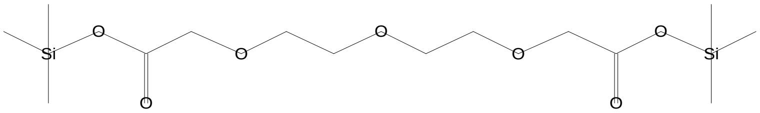

There is no signature data to report.

Library Search Report

RT	Probability	Compound Name	SI	Area %	Area	Molecular Weight	Molecular Formula	Library
18.15	4.72	á-D-Galactopyranoside, methyl 2,6-bis-O-(trimethylsilyl)-, cyclic butylboronate (CAS)	487	0.52	1640338.22	404	C17H37BO6Si2	Wiley9
18.15	4.17	á-D-Glucopyranoside, methyl 2,3-bis-O-(trimethylsilyl)-, cyclic methylboronate (CAS)	484	0.52	1640338.22	362	C14H31BO6Si2	Wiley9
18.15	3.19	10-Undecynoic acid, trimethylsilyl ester	477	0.52	1640338.22	254	C14H26O2Si	mainlib

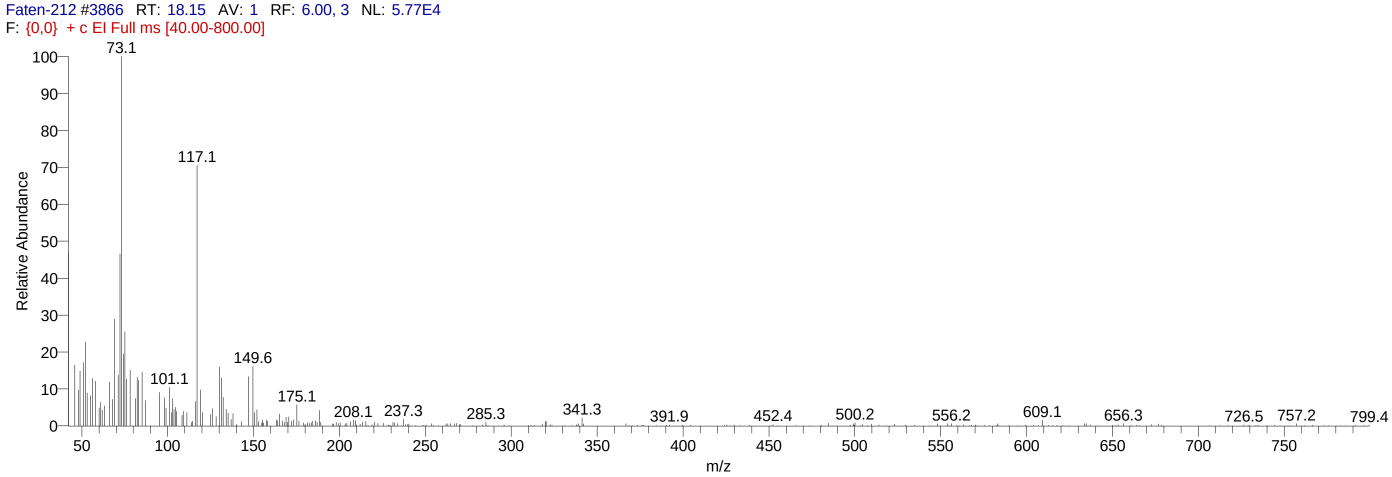

Hit Spectrum | Delta | Compound Structure

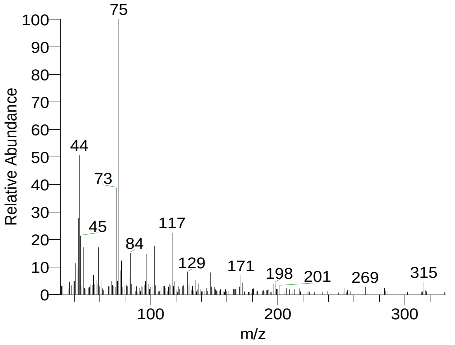

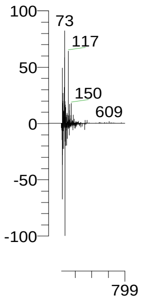

á-D-Galactopyranoside, methyl 2,6-bis-O-(trimethylsilyl)-, cyclic butylboronate (CAS)
Formula C17H37BO6Si2, MW 404, CAS# 56211-13-3, Entry# 546601
B-GALACTOPYRANOSIDE-1-METHYL-3,4-BUTYLBORONATE-2,6-DITMS

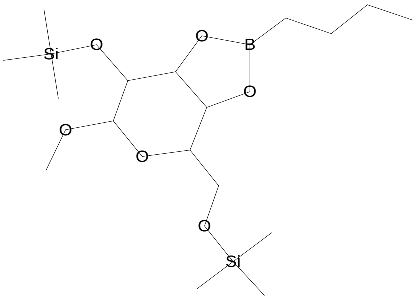

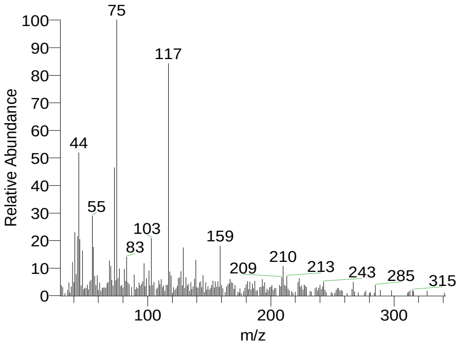

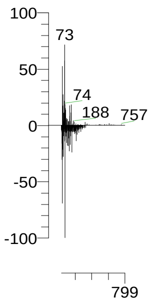

á-D-Glucopyranoside, methyl 2,3-bis-O-(trimethylsilyl)-, cyclic methylboronate (CAS)
Formula C14H31BO6Si2, MW 362, CAS# 56211-07-5, Entry# 490302
B-GLUCOPYRANOSIDE-1-METHYL-4,6-METHYLBORONATE-2,3-DITMS

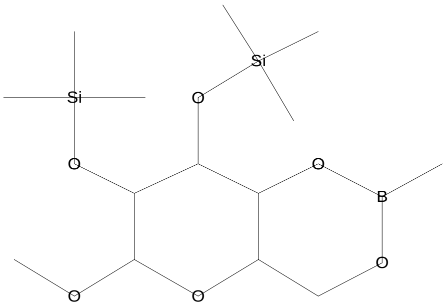

There is no signature data to report.

Library Search Report

Hit Spectrum	Delta	Compound Structure

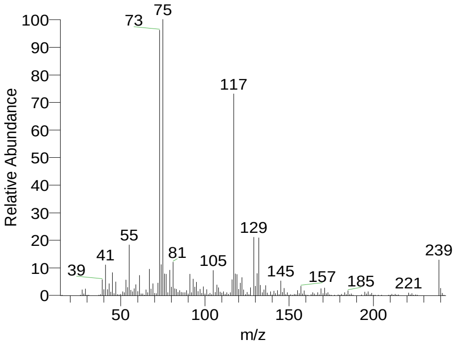

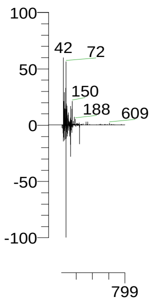

10-Undecynoic acid, trimethylsilyl ester
Formula C14H26O2Si, MW 254, CAS# NA, Entry# 41242

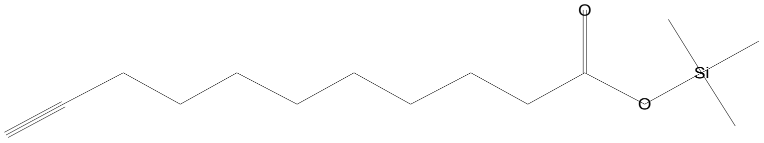

There is no signature data to report.

Library Search Report

RT	Probability	Compound Name	SI	Area %	Area	Molecular Weight	Molecular Formula	Library
18.43	15.26	8,11,14-Eicosatrienoic acid, methyl ester	463	0.86	2726093.57	320	C21H36O2	mainlib
18.43	15.26	8,11,14-Eicosatrienoic acid, methyl ester (CAS)	461	0.86	2726093.57	320	C21H36O2	Wiley9
18.43	5.57	4,6,6-Trimethyl-2-(3-methylbuta-1,3-dienyl)-3-oxatricyclo[5.1.0.0(2,4)]octane	440	0.86	2726093.57	218	C15H22O	Wiley9

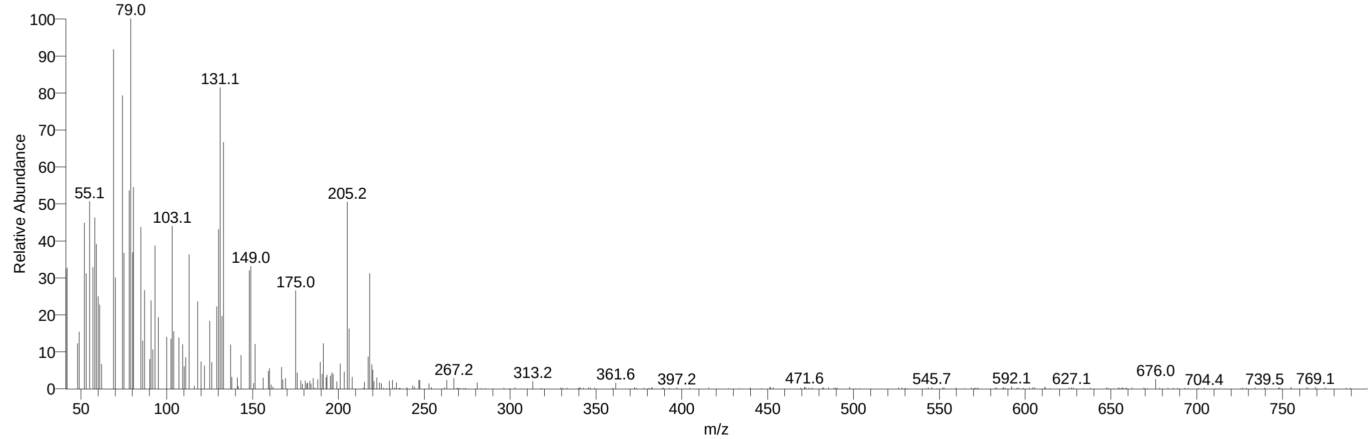

Hit Spectrum	Delta	Compound Structure

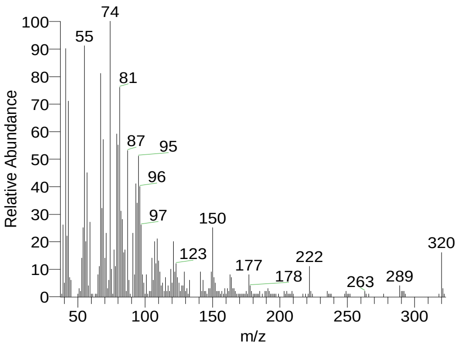

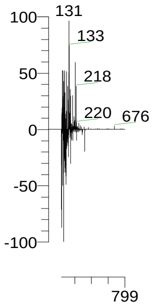

8,11,14-Eicosatrienoic acid, methyl ester
Formula C21H36O2, MW 320, CAS# 17364-32-8, Entry# 40569
Methyl (8E,11E,14E)-8,11,14-icosatrienoate #

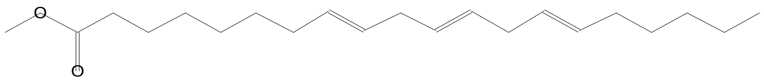

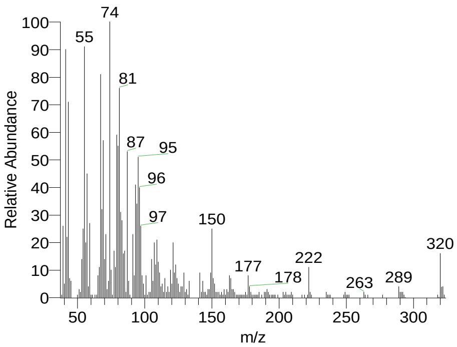

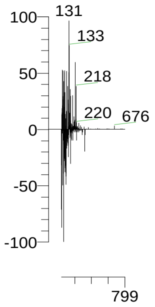

8,11,14-Eicosatrienoic acid, methyl ester (CAS)
Formula C21H36O2, MW 320, CAS# 17364-32-8, Entry# 417540
METHYL 8,11,14-EICOSATRIENOATE

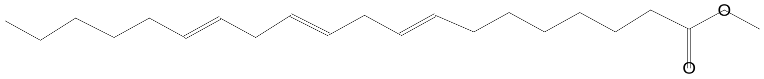

There is no signature data to report.

Library Search Report

Hit Spectrum	Delta	Compound Structure

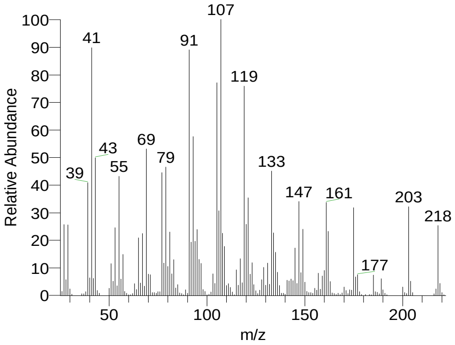

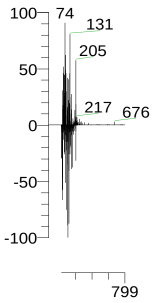

4,6,6-Trimethyl-2-(3-methylbuta-1,3-dienyl)-3-oxatricyclo[5.1.0.0(2,4)]octane
Formula C15H22O, MW 218, CAS# NA, Entry# 184075
4,6,6-TRIMETHYL-2-(3-METHYL-BUTA-1,3-DIENYL)-3-OXA-TRICYCLO[5.1.0.0 2,4]OCTANE

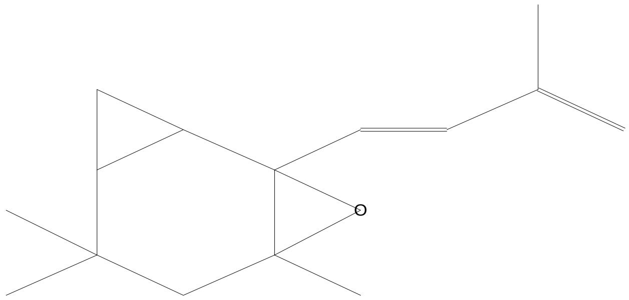

There is no signature data to report.

Library Search Report

RT	Probability	Compound Name	SI	Area %	Area	Molecular Weight	Molecular Formula	Library
18.84	5.29	Methyl à,á-D-galactoside, 2,3-O-diacetyl-4,6-dideoxy-4,6-diacetylthio-	392	0.36	1132990.22	394	C15H22O8S2	mainlib
18.84	5.29	Methyl à,á-D-galactoside, 2,3-O-diacetyl-4,6-dideoxy-4,6-diacetylthio-	392	0.36	1132990.22	394	C15H22O8S2	Wiley9
18.84	4.88	2-(2,4-Dinitrophenyl)-2-methylpropionitrile	390	0.36	1132990.22	235	C10H9N3O4	Wiley9

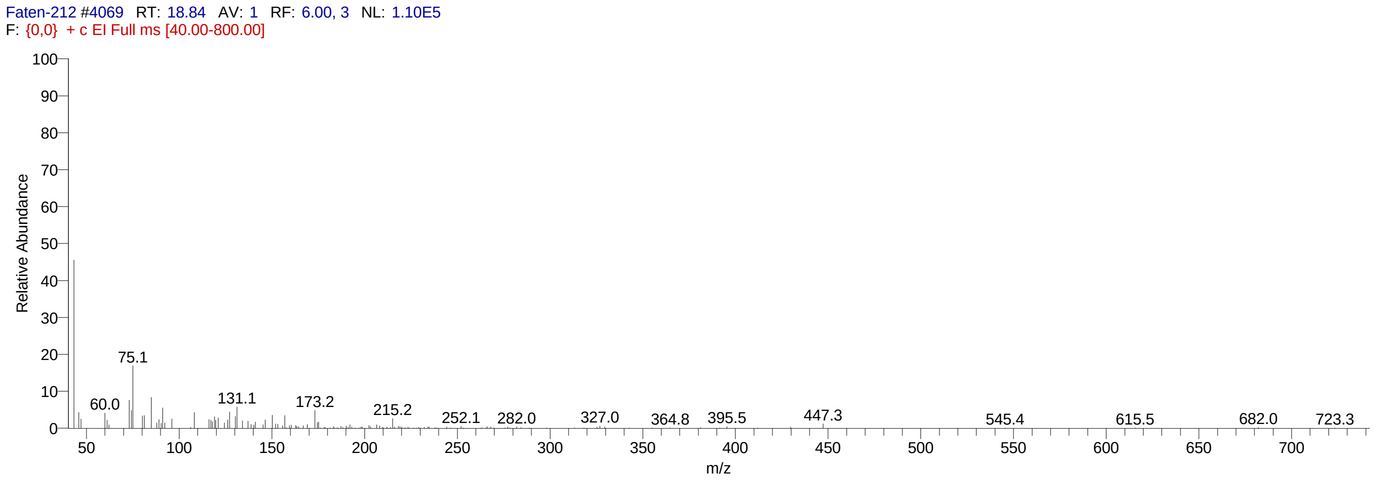

Hit Spectrum	Delta	Compound Structure

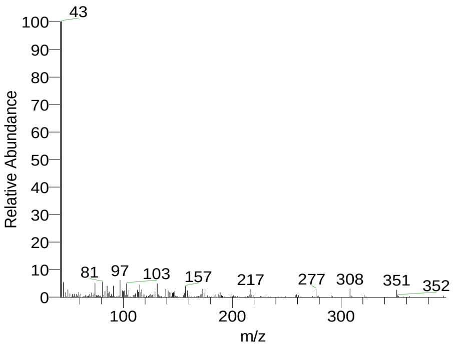

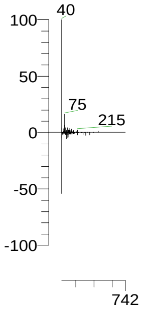

Methyl à,á-D-galactoside, 2,3-O-diacetyl-4,6-dideoxy-4,6-diacetylthio-
Formula C15H22O8S2, MW 394, CAS# 64698-04-0, Entry# 9867
Methyl à-D-galactoside, 4,6-dideoxy-4,6-dimercapto-2,3,4,6-tetraacetyl-

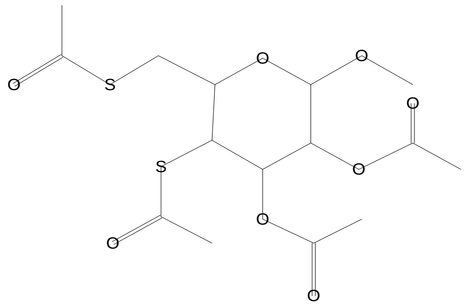

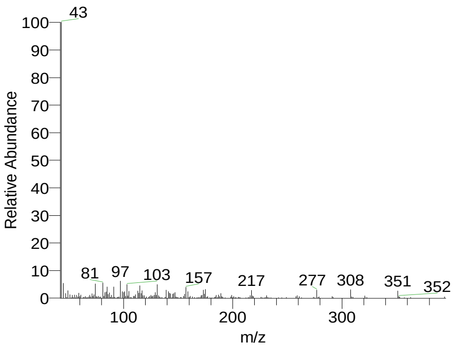

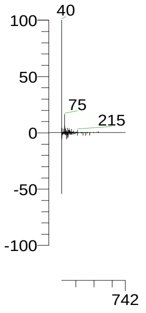

Methyl à,á-D-galactoside, 2,3-O-diacetyl-4,6-dideoxy-4,6-diacetylthio-
Formula C15H22O8S2, MW 394, CAS# 64698-04-0, Entry# 534702
Methyl à-D-galactoside, 4,6-dideoxy-4,6-dimercapto-2,3,4,6-tetraacetyl-

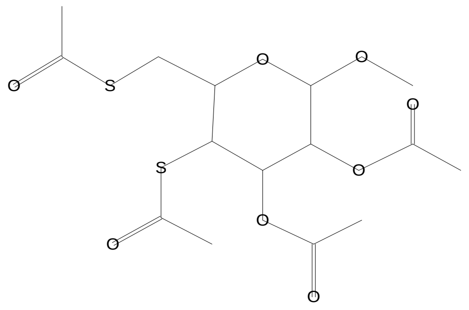

There is no signature data to report.

Library Search Report

Hit Spectrum	Delta	Compound Structure

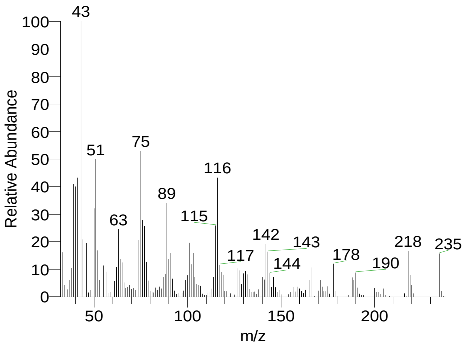

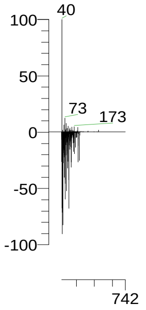

2-(2,4-Dinitrophenyl)-2-methylpropionitrile
Formula C10H9N3O4, MW 235, CAS# 313503-32-1, Entry# 222951
2-(2,4-Dinitrophenyl)-2-methylpropanenitrile

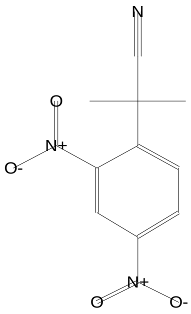

There is no signature data to report.

Library Search Report

RT	Probability	Compound Name	SI	Area %	Area	Molecular Weight	Molecular Formula	Library
18.93	11.57	2-Cyclohexene-1-carboxylic acid, 1,3-dimethyl-2-(3-methyl-7-oxo-1,3-octadienyl)-4-oxo-, methyl ester, (+)-	391	0.13	421470.06	318	C19H26O4	mainlib
18.93	11.57	Methyl trisporate B	391	0.13	421470.06	318	C19H26O4	Wiley9
18.93	9.09	Olean-12-ene-3,16,21,22,28-pentol, (3á,16à,21á,22à)-	385	0.13	421470.06	490	C30H50O5	mainlib

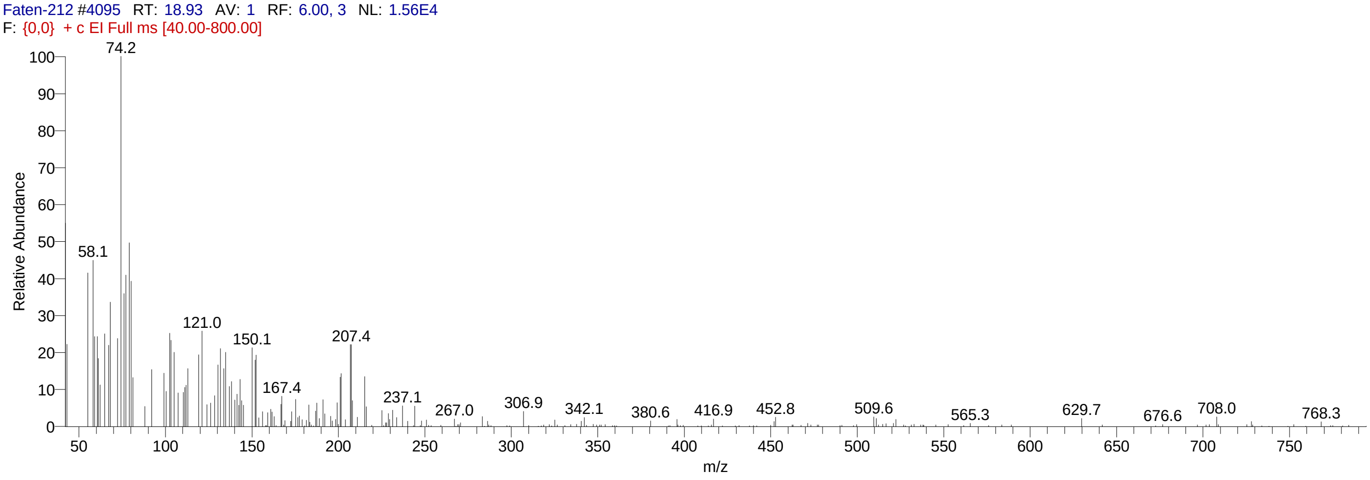

Hit Spectrum | Delta | Compound Structure

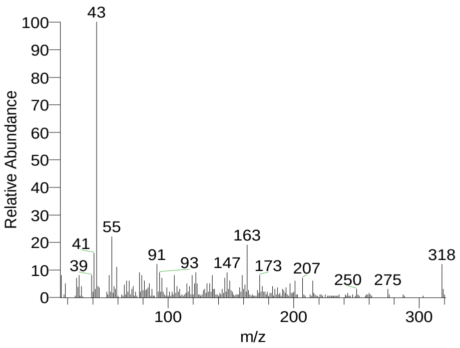

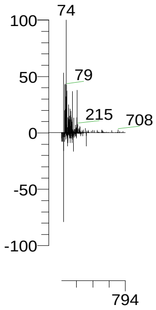

Formula C19H26O4, MW 318, CAS# 16981-58-1, Entry# 7084
Methyl trisporate B

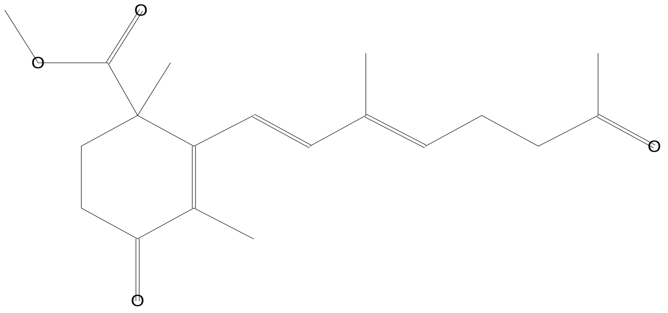

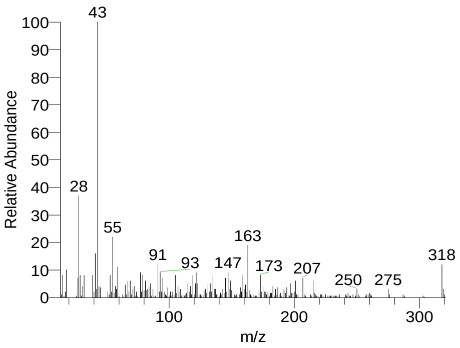

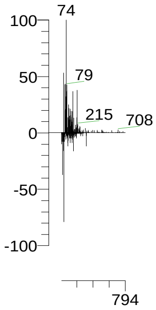

Methyl trisporate B
Formula C19H26O4, MW 318, CAS# 16981-58-1, Entry# 412848

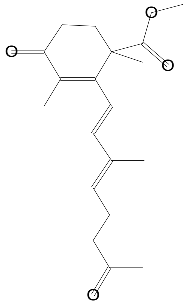

There is no signature data to report.

Library Search Report

Hit Spectrum	Delta	Compound Structure

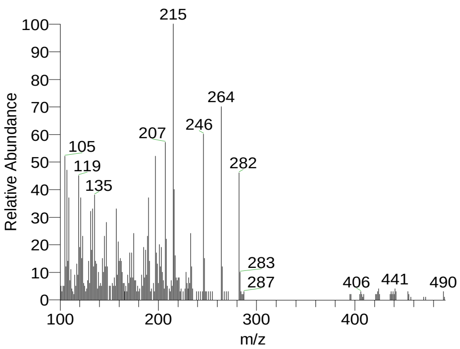

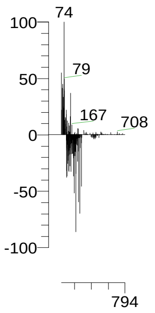

Olean-12-ene-3,16,21,22,28-pentol, (3á,16à,21á,22à)-
Formula C30H50O5, MW 490, CAS# 13844-01-4, Entry# 170277
Olean-12-ene-3á,16à,21á,22à,28-pentol

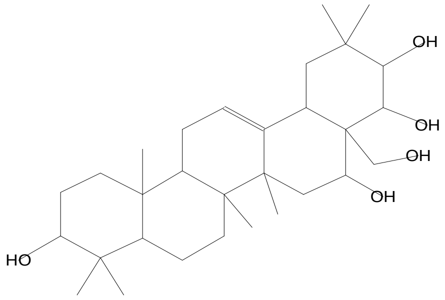

There is no signature data to report.

Library Search Report

RT	Probability	Compound Name	SI	Area %	Area	Molecular Weight	Molecular Formula	Library
19.26	21.26	5-(4-Chlorophenyl)-3-(3-phenylsydnon-4-yl)-1H-[1,2,4]triazole	425	0.17	534594.69	339	C16H10ClN5O2	Wiley9
19.26	15.44	3,20-Dioxo-11-à-hydroxyconanine-1,4-diene	416	0.17	534594.69	341	C21H27NO3	Wiley9
19.26	3.81	1-Methyl-7-azabicyclo[4.1.0]hepta-2,4-diene-7-carboxylic acid, 3,17-diacetoxy-4,4,10,13-tetramethylhexadecahydrocyclopenta[a]phenanthrene	380	0.17	534594.69	553	C33H47NO6	mainlib

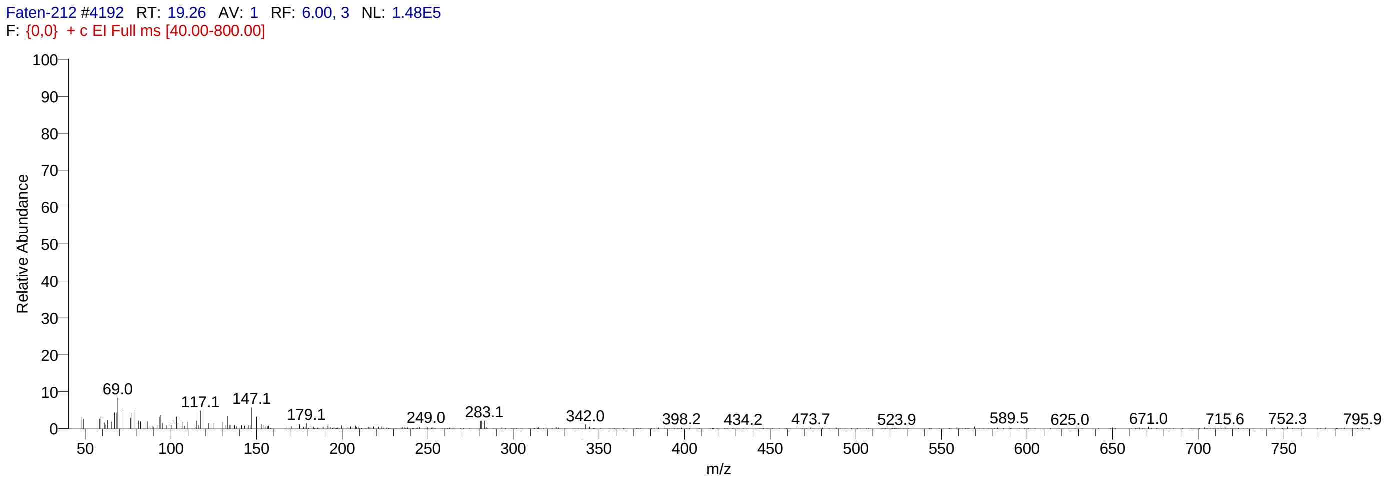

Hit Spectrum	Delta	Compound Structure

5-(4-Chlorophenyl)-3-(3-phenylsydnon-4-yl)-1H-[1,2,4]triazole
Formula C16H10ClN5O2, MW 339, CAS# NA, Entry# 452120

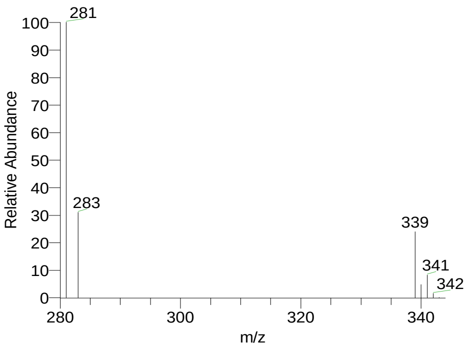

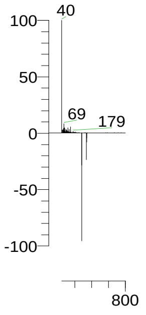

3,20-Dioxo-11-à-hydroxyconanine-1,4-diene
Formula C21H27NO3, MW 341, CAS# NA, Entry# 456515

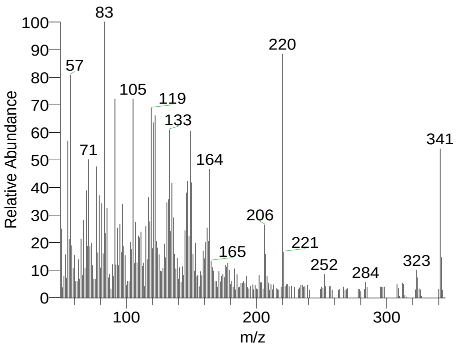

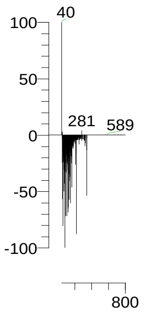

There is no signature data to report.

Library Search Report

Hit Spectrum	Delta	Compound Structure

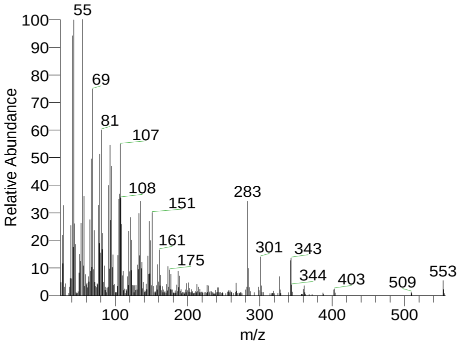

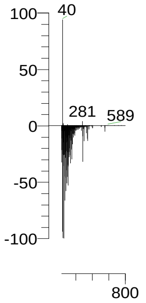

Formula C33H47NO6, MW 553, CAS# NA, Entry# 18335

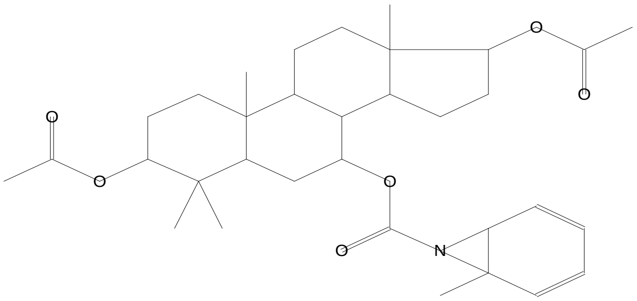

There is no signature data to report.

Library Search Report

RT	Probability	Compound Name	SI	Area %	Area	Molecular Weight	Molecular Formula	Library
19.82	19.04	Epimethendiol-diOTMS	490	0.99	3156981.79	448	C26H48O2Si2	mainlib
19.82	14.20	4-Methyl(trimethylene)silyloxyoctane	482	0.99	3156981.79	214	C12H26OSi	mainlib
19.82	13.10	Androsta-1,4-dien-3-one, 17-methyl-17-[(trimethylsilyl)oxy]-, O-methyloxime, (17á)-	480	0.99	3156981.79	401	C24H39NO2Si	mainlib

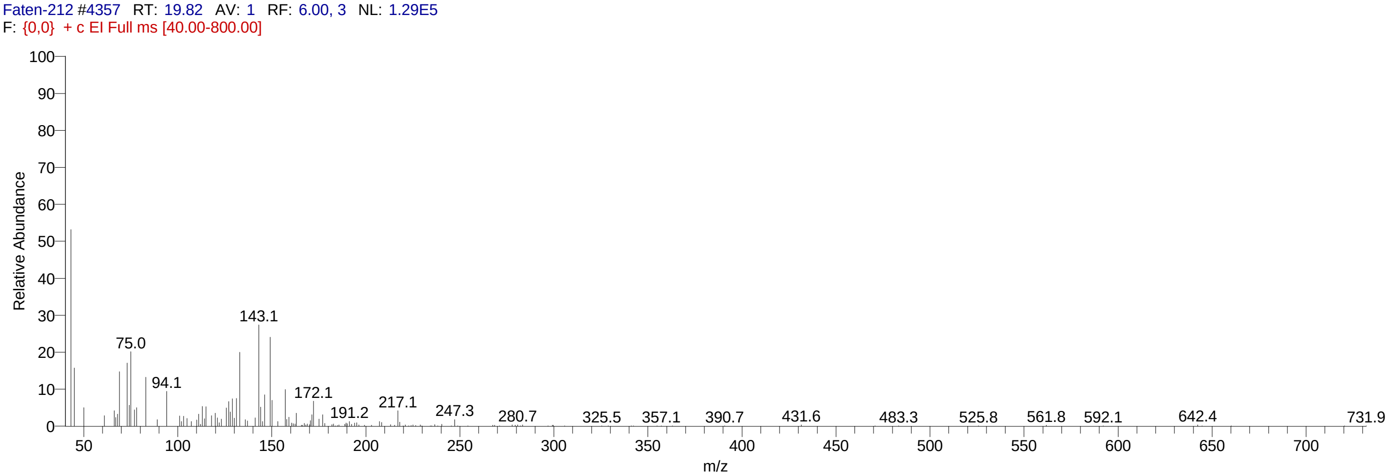

Hit Spectrum	Delta	Compound Structure

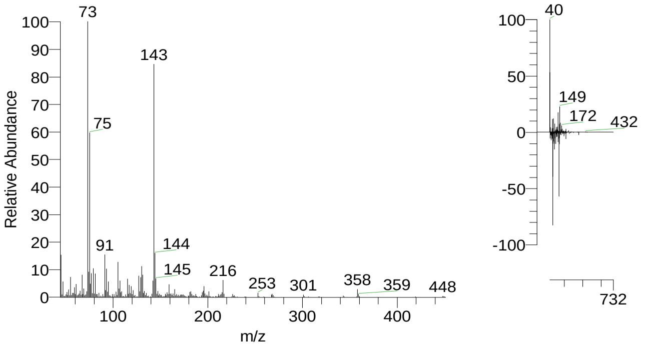

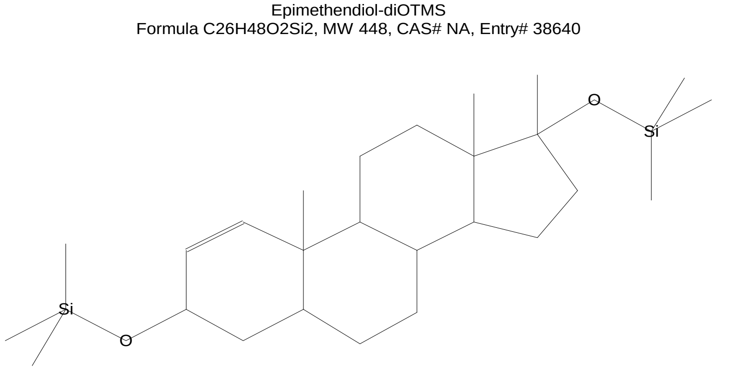

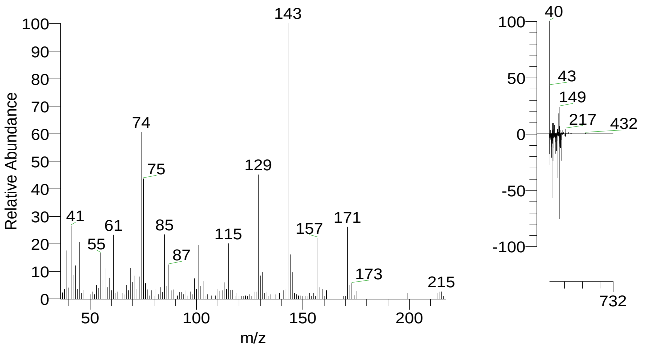

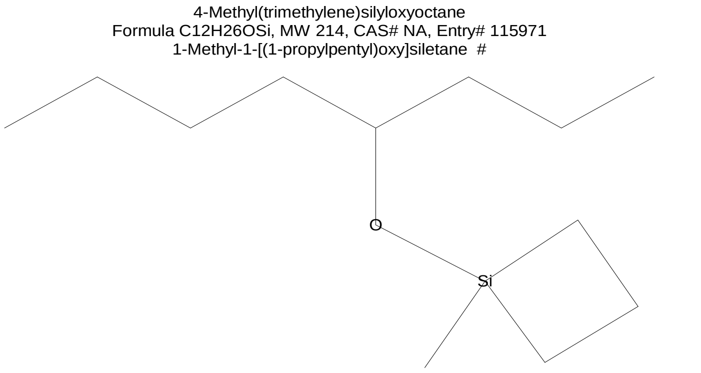

There is no signature data to report.

Library Search Report

Hit Spectrum	Delta	Compound Structure

Androsta-1,4-dien-3-one, 17-methyl-17-[(trimethylsilyl)oxy]-, O-methyloxime, (17á)-
Formula C24H39NO2Si, MW 401, CAS# 57397-03-2, Entry# 38644
17-Methyl-17-[(trimethylsilyl)oxy]androsta-1,4-dien-3-one o-methyloxime #

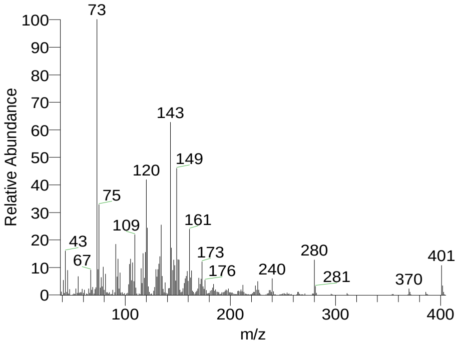

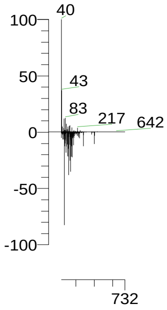

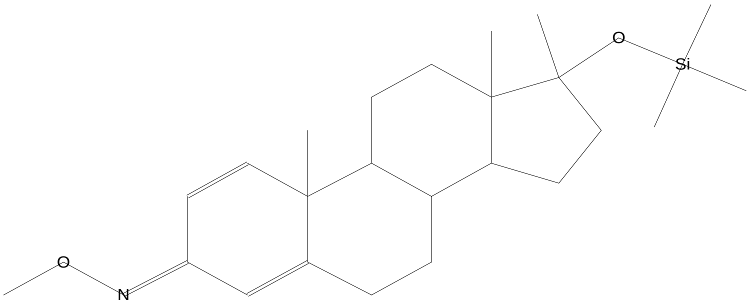

There is no signature data to report.

Library Search Report

RT	Probability	Compound Name	SI	Area %	Area	Molecular Weight	Molecular Formula	Library
20.27	21.14	[1,1'-Bicyclopropyl]-2-octanoic acid, 2'-hexyl-, methyl ester	411	0.25	780831.26	322	C21H38O2	mainlib
20.27	21.14	[1,1'-Bicyclopropyl]-2-octanoic acid, 2'-hexyl-, methyl ester (CAS)	410	0.25	780831.26	322	C21H38O2	Wiley9
20.27	16.19	9,12,15-Octadecatrienoic acid, methyl ester (CAS)	404	0.25	780831.26	292	C19H32O2	Wiley9

Faten-212 #4489 RT: 20.27 AV: 1 RF: 6.00, 3 NL: 4.03E4
F: {0,0} + c EI Full ms [40.00-800.00]

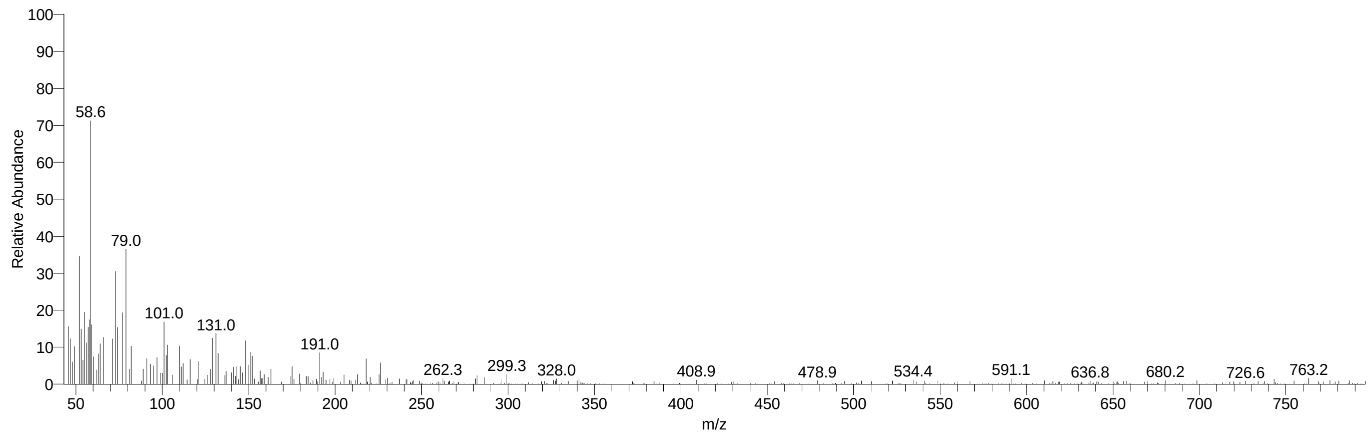

Hit Spectrum	Delta	Compound Structure

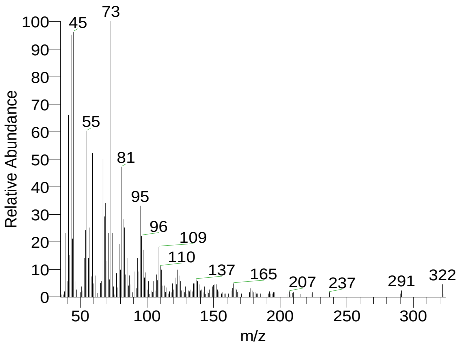

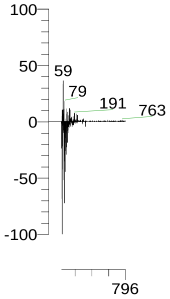

[1,1'-Bicyclopropyl]-2-octanoic acid, 2'-hexyl-, methyl ester
Formula C21H38O2, MW 322, CAS# 56687-68-4, Entry# 37105

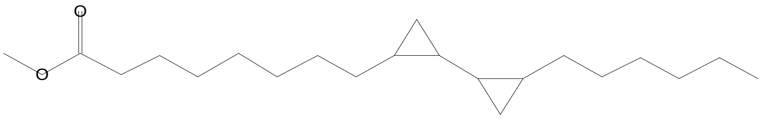

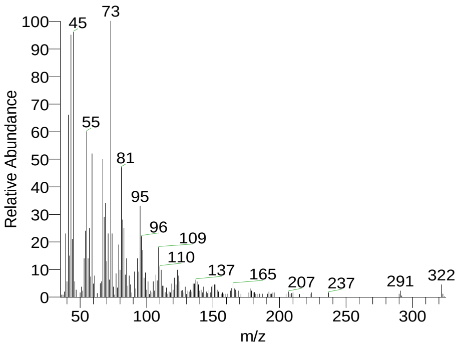

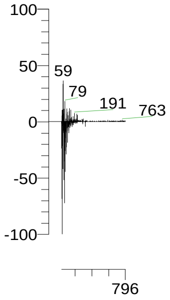

[1,1'-Bicyclopropyl]-2-octanoic acid, 2'-hexyl-, methyl ester (CAS)
Formula C21H38O2, MW 322, CAS# 56687-68-4, Entry# 421479
METHYL 9,10,11,12-DIMETHYLENE OCTADECANOATE

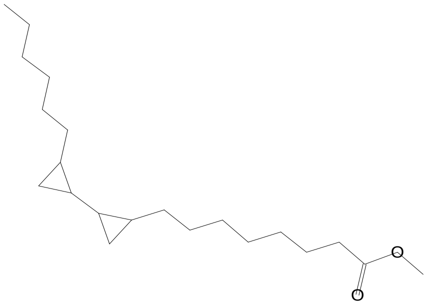

There is no signature data to report.

Library Search Report

Hit Spectrum	Delta	Compound Structure

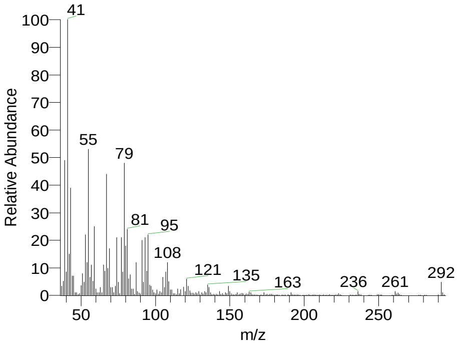

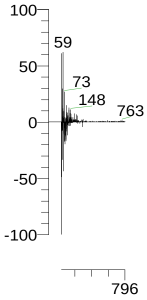

9,12,15-Octadecatrienoic acid, methyl ester (CAS)
Formula C19H32O2, MW 292, CAS# 7361-80-0, Entry# 357373
Methyl 9,12,15-octadecatrienoate

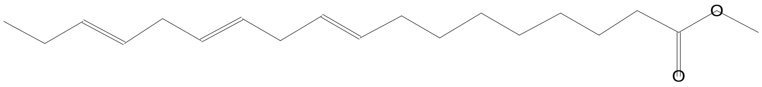

There is no signature data to report.

Library Search Report

RT	Probability	Compound Name	SI	Area %	Area	Molecular Weight	Molecular Formula	Library
20.87	10.43	á-N-Acetylneuraminic acid, methyl ester-2-methyl-8,9-methyl-boronate-3,7-di(trimethylsilyl)-	331	0.37	1178136.59	505	C20H40BNO9Si2	mainlib
20.87	10.43	B-N-ACETYLNEURAMINIC ME ESTER-2-ME-8,9-ME-BORONATE-3,7-DITMS	331	0.37	1178136.59	505	C20H40BNO9Si2	Wiley9
20.87	7.99	Digitoxin	324	0.37	1178136.59	764	C41H64O13	Wiley9

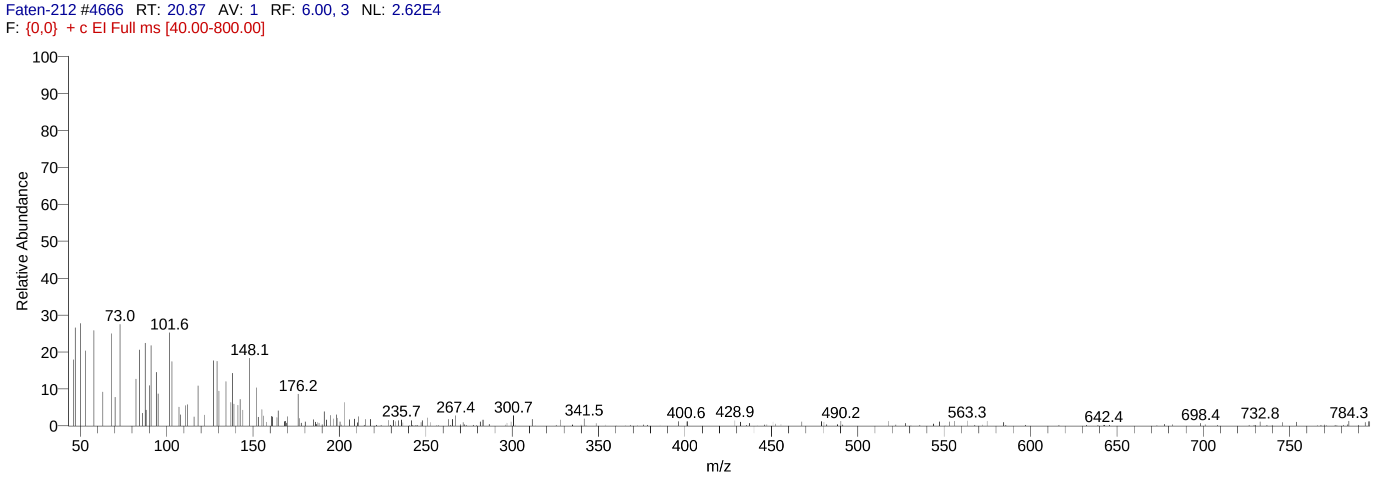

Hit Spectrum	Delta	Compound Structure

á-N-Acetylneuraminic acid, methyl ester-2-methyl-8,9-methyl-boronate-3,7-di(trimethylsilyl)-
Formula C20H40BNO9Si2, MW 505, CAS# NA, Entry# 41310

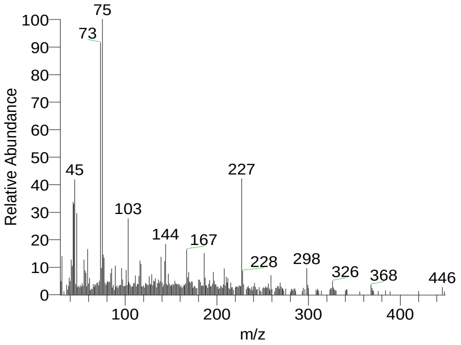

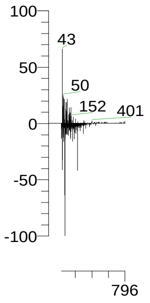

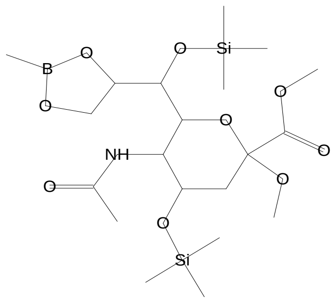

B-N-ACETYLNEURAMINIC ME ESTER-2-ME-8,9-ME-BORONATE-3,7-DITMS
Formula C20H40BNO9Si2, MW 505, CAS# NA, Entry# 619809

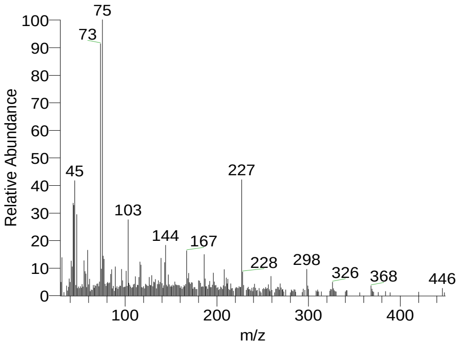

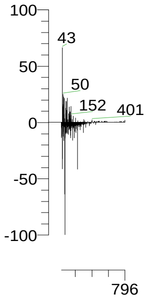

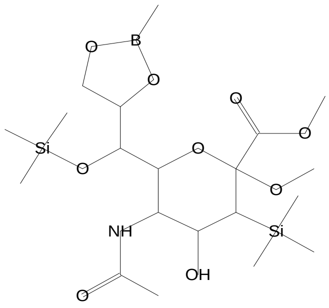

There is no signature data to report.

Library Search Report

Hit Spectrum	Delta	Compound Structure

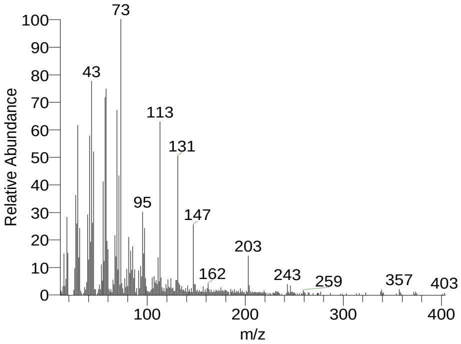

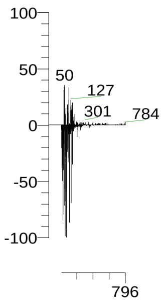

Digitoxin
Formula C41H64O13, MW 764, CAS# 71-63-6, Entry# 657631

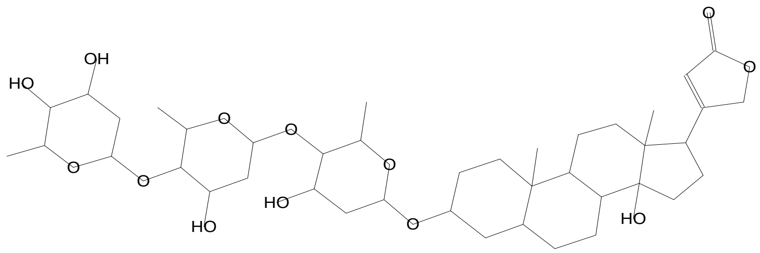

There is no signature data to report.

Library Search Report

RT	Probability	Compound Name	SI	Area %	Area	Molecular Weight	Molecular Formula	Library
20.96	43.38	Formic acid, ethenyl ester (CAS)	505	0.22	706019.11	72	C3H4O2	Wiley9
20.96	12.52	ethenol	477	0.22	706019.11	44	C2H4O	Wiley9
20.96	10.09	Propane (CAS)	472	0.22	706019.11	44	C3H8	Wiley9

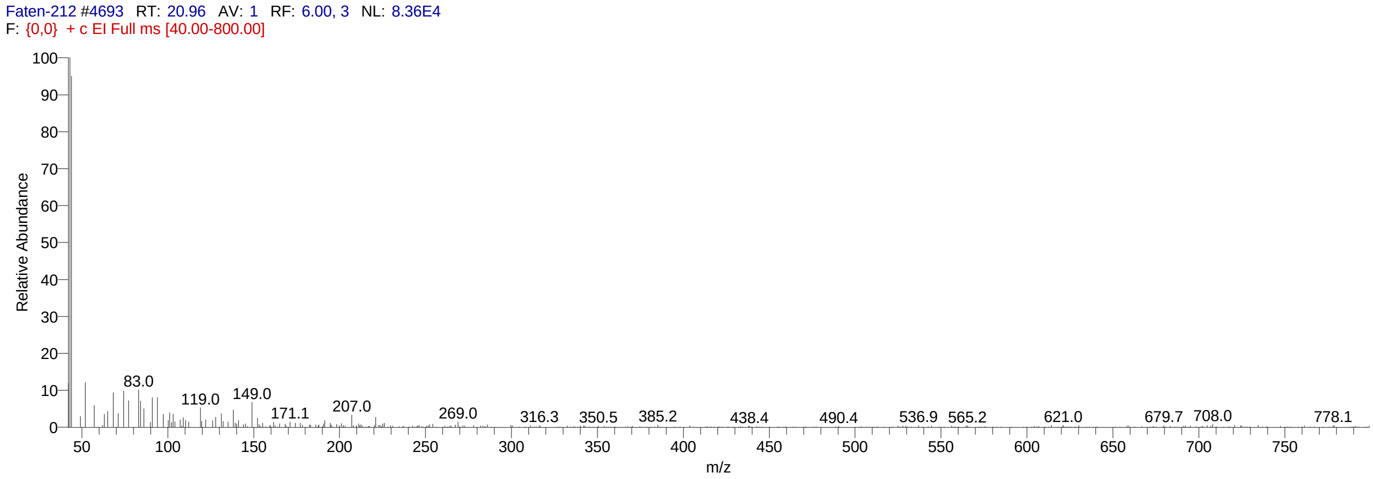

Hit Spectrum | Delta | Compound Structure

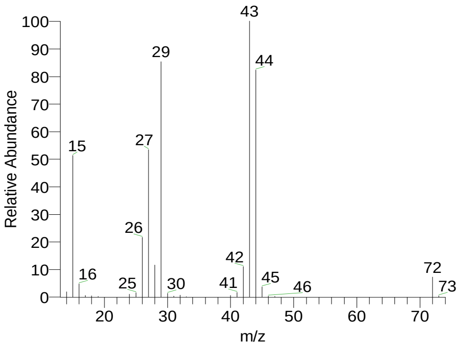

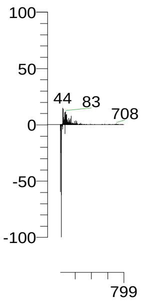

Formic acid, ethenyl ester (CAS)
Formula C3H4O2, MW 72, CAS# 692-45-5, Entry# 1494
Vinyl formate

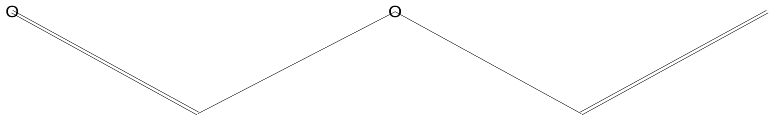

SI 477, RSI 930, Wiley9, Entry# 221, CAS# 57239-63-1, ethenol

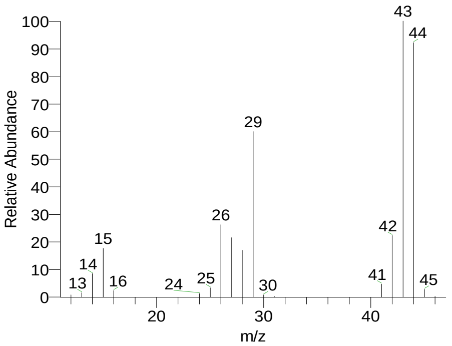

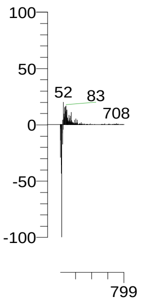

ethenol
Formula C2H4O, MW 44, CAS# 57239-63-1, Entry# 221
Ethenol, radical ion(1+) (CAS)

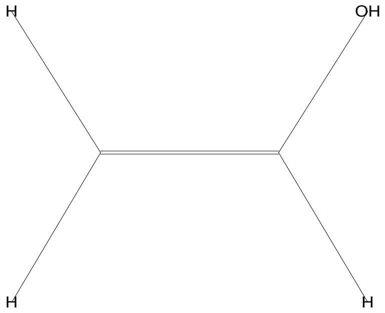

There is no signature data to report.

Library Search Report

Hit Spectrum | Delta | Compound Structure

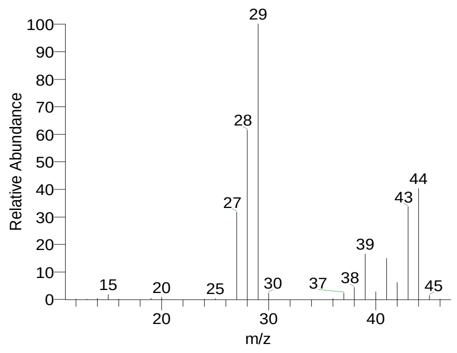

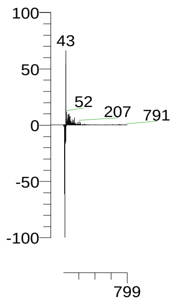

Propane (CAS)
Formula C3H8, MW 44, CAS# 74-98-6, Entry# 225
R 290

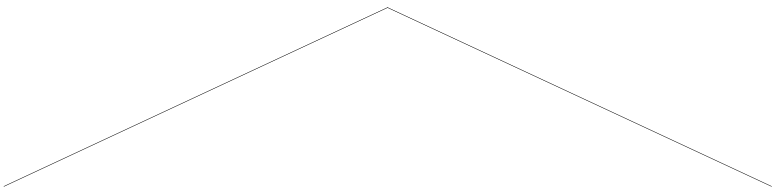

There is no signature data to report.

Library Search Report

RT	Probability	Compound Name	SI	Area %	Area	Molecular Weight	Molecular Formula	Library
21.50	5.86	7-Isopropylidene-5-methyl-2,3-diazabicyclo[2.2.1]hept-5-ene-2,3-dicarboxylic acid, diethyl ester	425	0.13	428552.38	294	C15H22N2O4	mainlib
21.50	5.86	7-Isopropylidene-5-methyl-2,3-diazabicyclo[2.2.1]hept-5-ene-2,3-dicarboxylic acid, diethyl ester	425	0.13	428552.38	294	C15H22N2O4	Wiley9
21.50	5.40	1-Deoxy-1-[3-(o-tolyl)-2-thioureido]-á-d-glucopyranose 2,3,4,6-tetraacetate	423	0.13	428552.38	496	C22H28N2O9S	mainlib

Faten-212 #4853 RT: 21.50 AV: 1 RF: 6.00, 3 NL: 6.74E4
F: {0,0} + c EI Full ms [40.00-800.00]

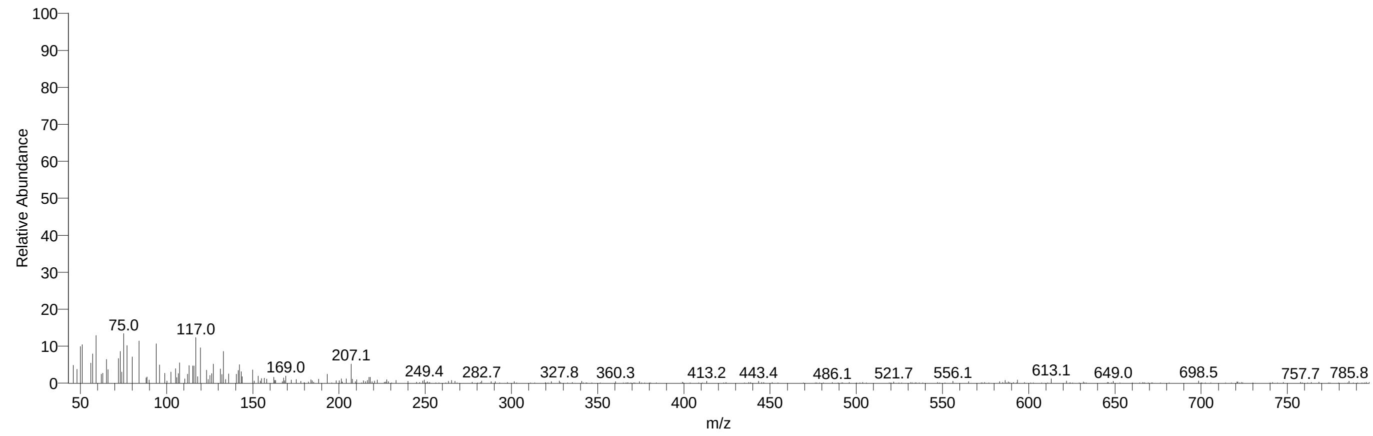

Hit Spectrum	Delta	Compound Structure

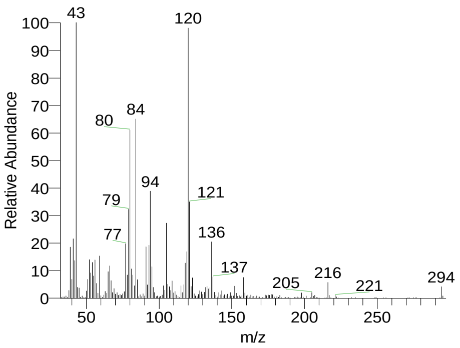

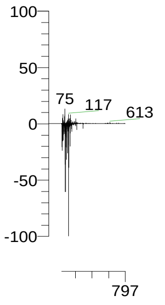

7-Isopropylidene-5-methyl-2,3-diazabicyclo[2.2.1]hept-5-ene-2,3-dicarboxylic acid, diethyl ester
Formula C15H22N2O4, MW 294, CAS# NA, Entry# 10886
Diethyl 5-methyl-7-(1-methylethylidene)-2,3-diazabicyclo[2.2.1]hept-5-ene-2,3-dicarboxylate #

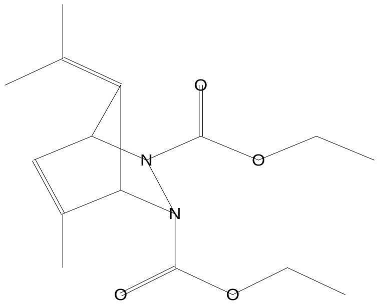

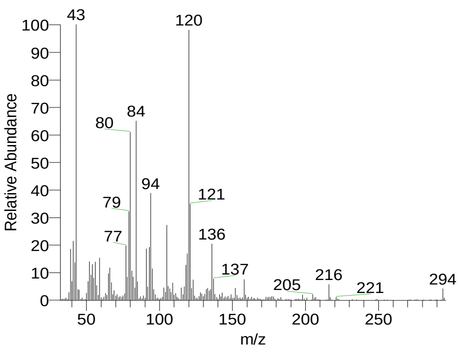

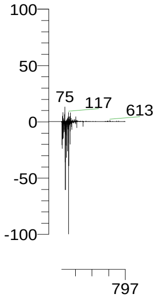

7-Isopropylidene-5-methyl-2,3-diazabicyclo[2.2.1]hept-5-ene-2,3-dicarboxylic acid, diethyl ester
Formula C15H22N2O4, MW 294, CAS# NA, Entry# 360410
7-ISOPROPYLIDENE-5-METHYL-2,3-DIAZA-BICYCLO[2.2.1]HEPT-5-ENE-2,3-DICARBOXYLIC AC

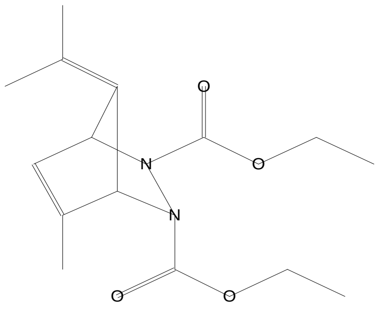

There is no signature data to report.

Library Search Report

Hit Spectrum	Delta	Compound Structure

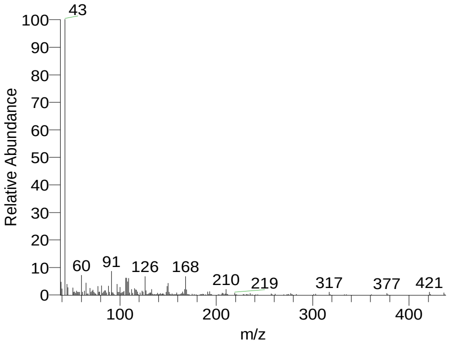

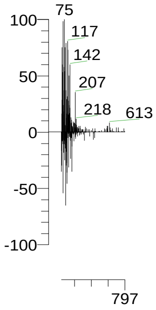

1-Deoxy-1-[3-(o-tolyl)-2-thioureido]-á-d-glucopyranose 2,3,4,6-tetraacetate
Formula C22H28N2O9S, MW 496, CAS# NA, Entry# 9624
2,3,4,6-Tetra-O-acetyl-N-(2-toluidinocarbothioyl)hexopyranosylamine #

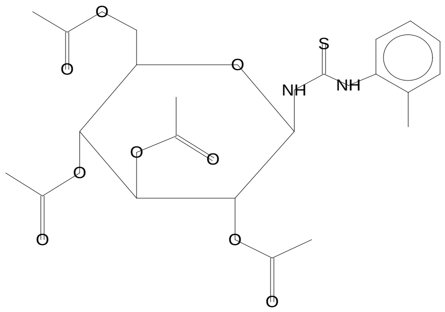

There is no signature data to report.

Library Search Report

RT	Probability	Compound Name	SI	Area %	Area	Molecular Weight	Molecular Formula	Library
22.38	45.05	1-Nitro-á-d-arabinofuranose, tetraacetate	554	0.35	1126679.55	363	C13H17NO11	mainlib
22.38	3.97	N-(2,3,4,6-Tetra-O-acetyl-á-d-glucopyranosyl)-glycine, ethyl ester	467	0.35	1126679.55	433	C18H27NO11	mainlib
22.38	3.12	METHYL 2,3,5,6-TETRA-O-ACETYLHEXOFURANOSIDE #	461	0.35	1126679.55	362	C15H22O10	Wiley9

Faten-212 #5110 RT: 22.38 AV: 1 RF: 6.00, 3 NL: 1.98E5
F: {0,0} + c EI Full ms [40.00-800.00]

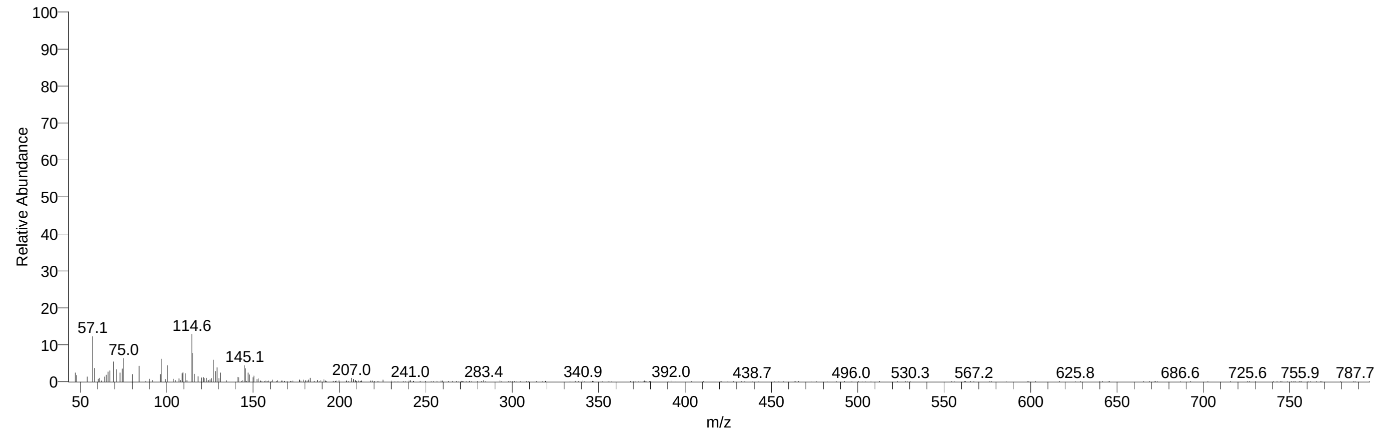

Hit Spectrum	Delta	Compound Structure

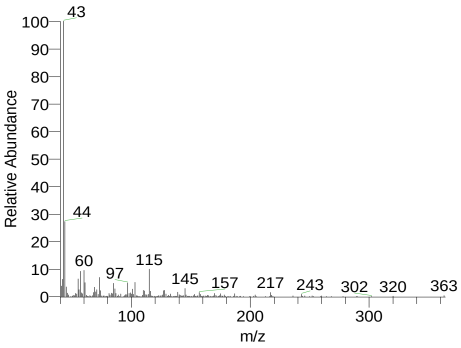

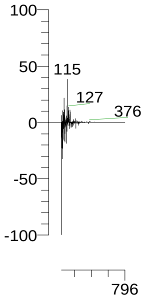

1-Nitro-á-d-arabinofuranose, tetraacetate
Formula C13H17NO11, MW 363, CAS# NA, Entry# 6423

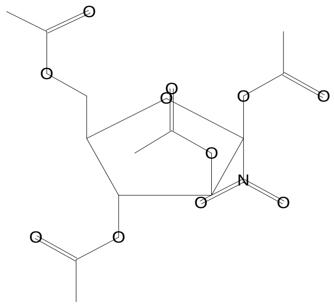

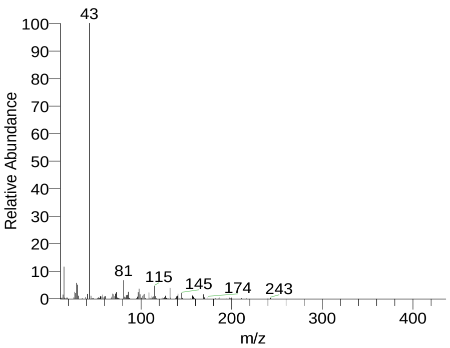

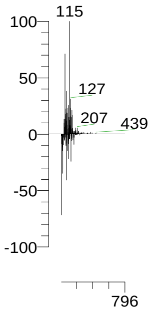

N-(2,3,4,6-Tetra-O-acetyl-á-d-glucopyranosyl)-glycine, ethyl ester
Formula C18H27NO11, MW 433, CAS# NA, Entry# 5196

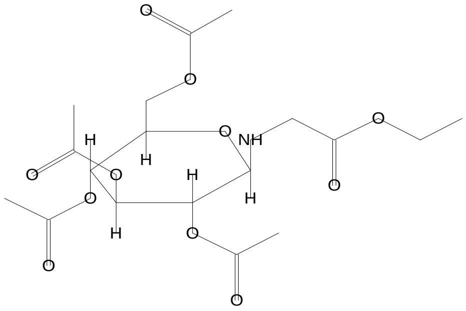

There is no signature data to report.

Library Search Report

Hit Spectrum	Delta	Compound Structure

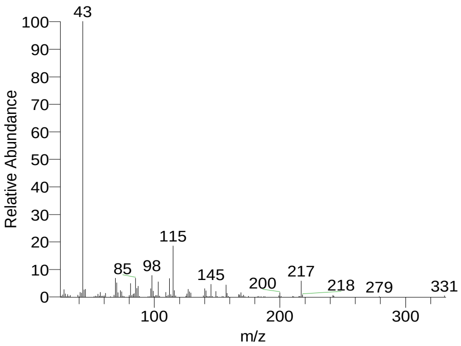

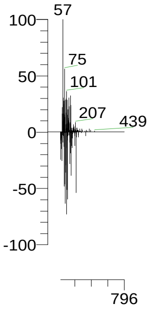

METHYL 2,3,5,6-TETRA-O-ACETYLHEXOFURANOSIDE #
Formula C15H22O10, MW 362, CAS# 24916-40-3, Entry# 490363
á-D-MANNOFURANOSIDE, METHYL, TETRAACETATE

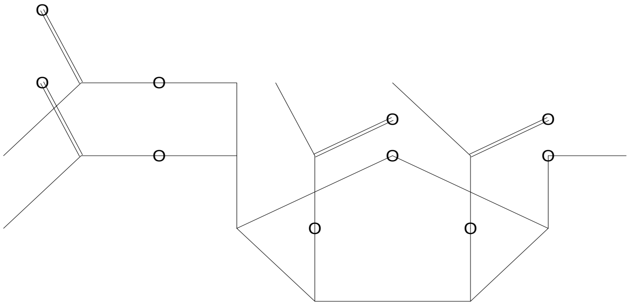

There is no signature data to report.

Library Search Report

RT	Probability	Compound Name	SI	Area %	Area	Molecular Weight	Molecular Formula	Library
23.16	54.34	TRANS-á-IONON-5,6-EPOXIDE	478	0.19	608833.83	208	C13H20O2	Wiley9
23.16	11.16	L(-)-CYSTINE	429	0.19	608833.83	240	C6H12N2O4S2	Wiley9
23.16	6.09	Cystine	412	0.19	608833.83	240	C6H12N2O4S2	mainlib

Faten-212 #5339 RT: 23.16 AV: 1 RF: 6.00, 3 NL: 8.16E4
F: {0,0} + c EI Full ms [40.00-800.00]

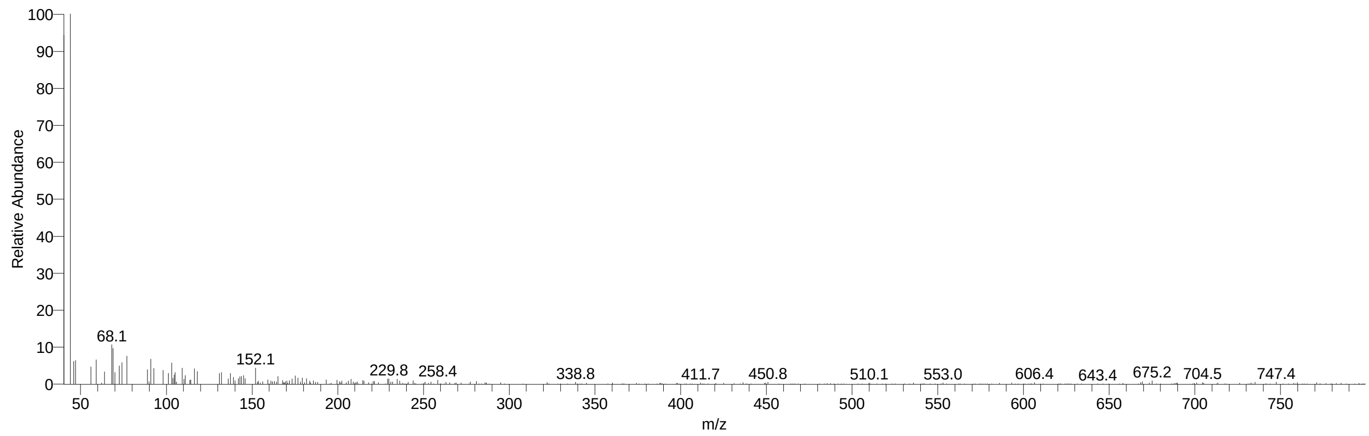

Hit Spectrum	Delta	Compound Structure

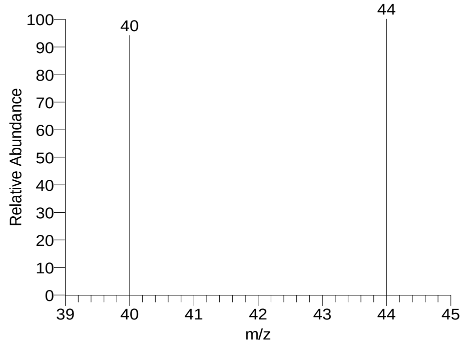

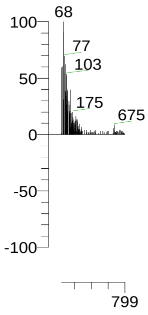

TRANS-á-IONON-5,6-EPOXIDE
Formula C13H20O2, MW 208, CAS# 23267-57-4, Entry# 161086
3-Buten-2-one, 4-(2,2,6-trimethyl-7-oxabicyclo[4.1.0]hept-1-yl)- (CAS)

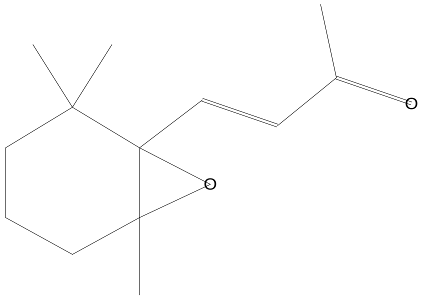

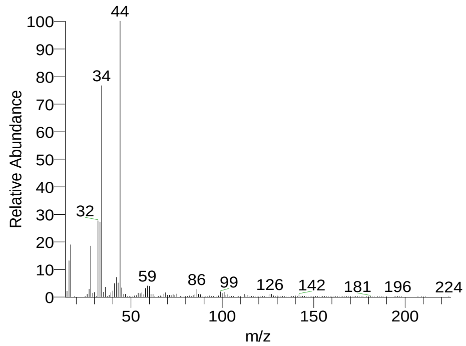

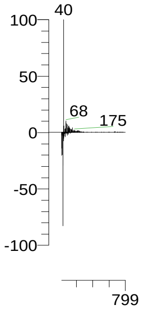

L(-)-CYSTINE
Formula C6H12N2O4S2, MW 240, CAS# 923-32-0, Entry# 234660
DL-Cystine

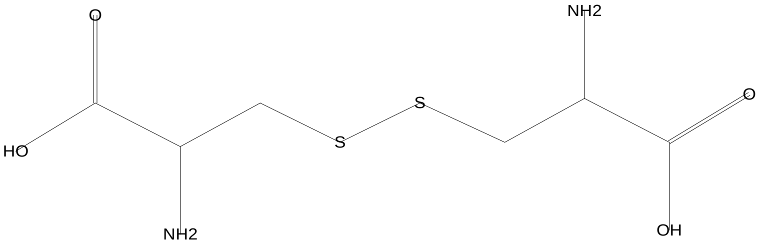

There is no signature data to report.

Library Search Report

Hit Spectrum | Delta | Compound Structure

SI 412, RSI 486, mainlib, Entry# 14242, CAS# 56-89-3, Cystine

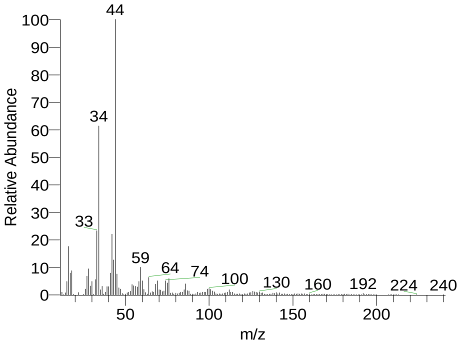

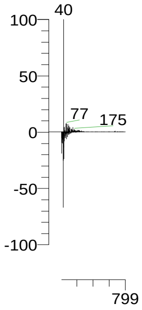

Cystine
Formula C6H12N2O4S2, MW 240, CAS# 56-89-3, Entry# 14242
l-Cystine

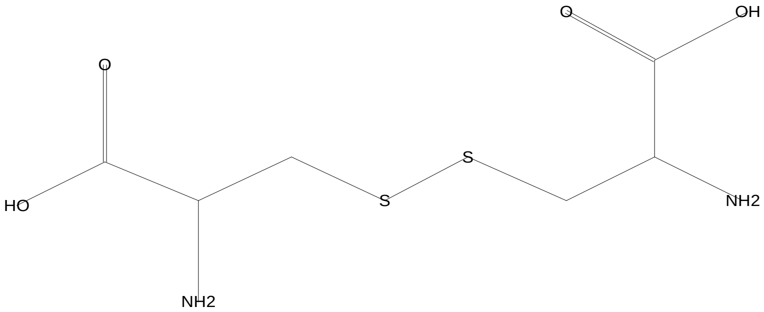

There is no signature data to report.

Library Search Report

RT	Probability	Compound Name	SI	Area %	Area	Molecular Weight	Molecular Formula	Library
23.57	9.87	2,2-Dimethyl-5-[2-(2-trimethylsilylethoxymethoxy)-propyl]-[1,3]dioxolane-4-carboxaldehyde	581	2.31	7334832.83	318	C15H30O5Si	mainlib
23.57	9.87	2,2-Dimethyl-5-[2-(2-trimethylsilylethoxymethoxy)-propyl]-[1,3]dioxolane-4-carboxaldehyde	581	2.31	7334832.83	318	C15H30O5Si	Wiley9
23.57	7.36	9,12,15-Octadecatrienoic acid, 2-[(trimethylsilyl)oxy]-1-[[(trimethylsilyl)oxy]methyl]ethyl ester, (Z,Z,Z)-	573	2.31	7334832.83	496	C27H52O4Si2	mainlib

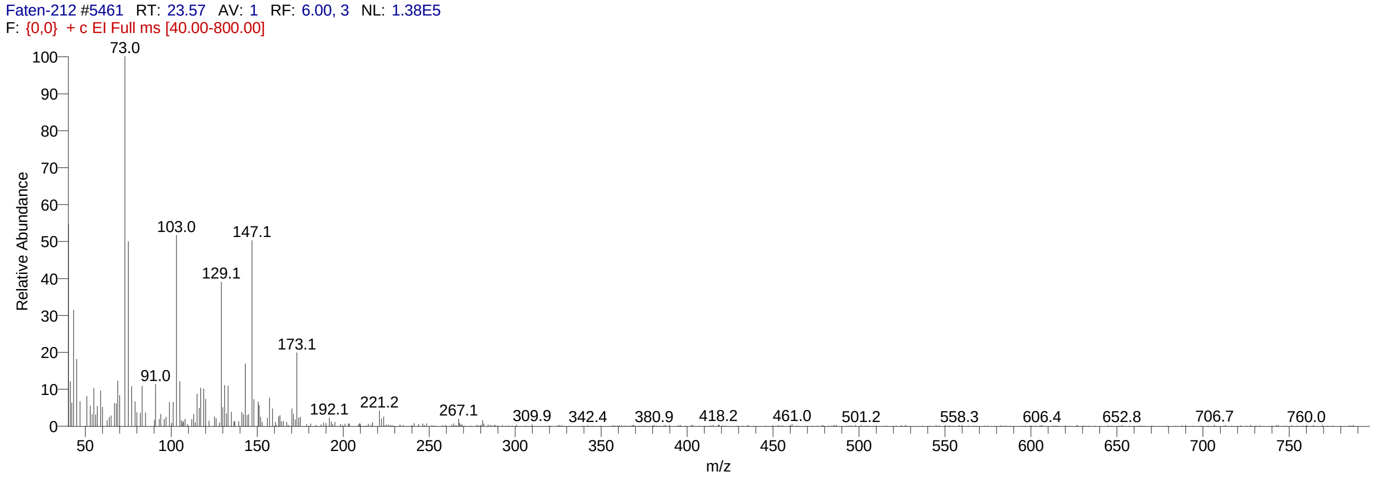

Hit Spectrum | Delta | Compound Structure

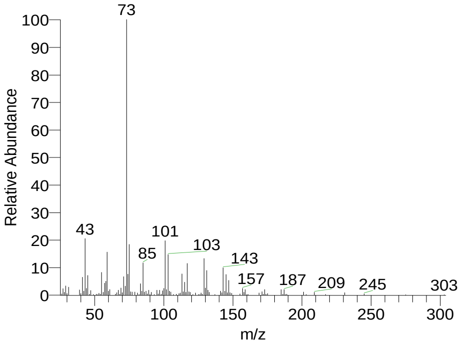

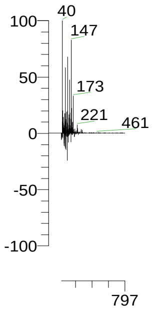

2,2-Dimethyl-5-[2-(2-trimethylsilylethoxymethoxy)-propyl]-[1,3]dioxolane-4-carboxaldehyde
Formula C15H30O5Si, MW 318, CAS# NA, Entry# 37019
4,6-Dideoxy-2,3-O-(1-methylethylidene)-5-O-([2-(trimethylsilyl)ethoxy]methyl)hexose #

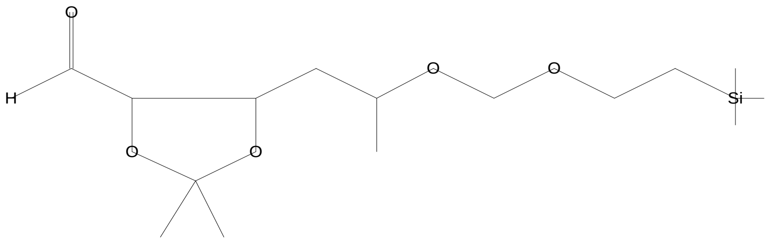

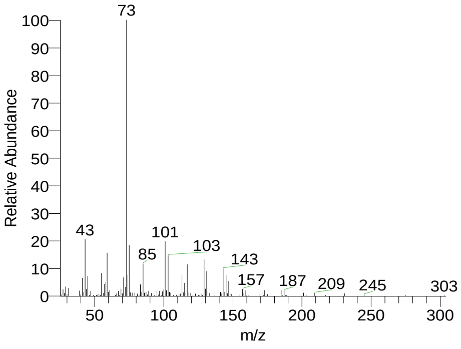

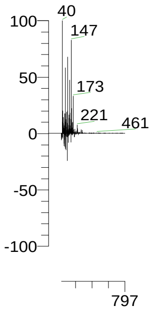

2,2-Dimethyl-5-[2-(2-trimethylsilylethoxymethoxy)-propyl]-[1,3]dioxolane-4-carboxaldehyde
Formula C15H30O5Si, MW 318, CAS# NA, Entry# 411942

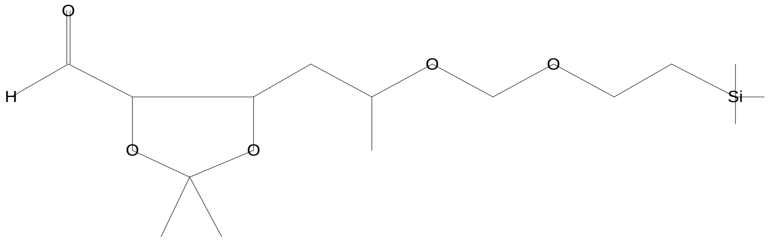

There is no signature data to report.

Library Search Report

Hit Spectrum	Delta	Compound Structure

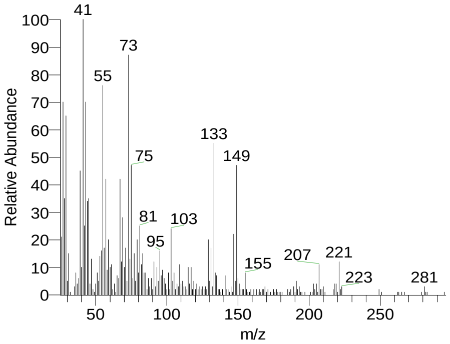

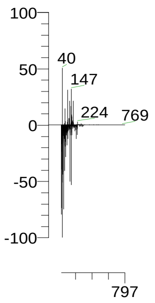

9,12,15-Octadecatrienoic acid, 2-[(trimethylsilyl)oxy]-1-[[(trimethylsilyl)oxy]methyl]ethyl ester, (Z,Z,Z)-
Formula C27H52O4Si2, MW 496, CAS# 55521-23-8, Entry# 3361
2-[(Trimethylsilyl)oxy]-1-([(trimethylsilyl)oxy]methyl)ethyl (9E,12E,15E)-9,12,15-octadecatrienoate #

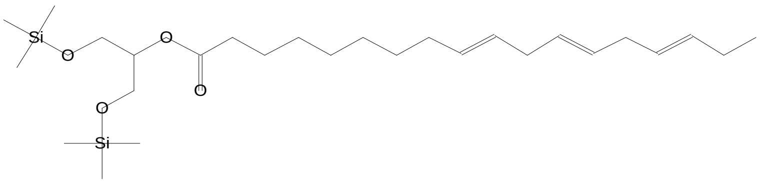

There is no signature data to report.

Library Search Report

RT	Probability	Compound Name	SI	Area %	Area	Molecular Weight	Molecular Formula	Library
23.76	10.17	á-D-Galactopyranoside, methyl 2,3-bis-O-(trimethylsilyl)-, cyclic butylboronate	458	0.26	811517.49	404	C17H37BO6Si2	mainlib
23.76	10.17	á-D-Galactopyranoside, methyl 2,3-bis-O-(trimethylsilyl)-, cyclic butylboronate (CAS)	458	0.26	811517.49	404	C17H37BO6Si2	Wiley9
23.76	6.16	à-D-Galactopyranoside, methyl 2,3-bis-O-(trimethylsilyl)-, cyclic methylboronate	443	0.26	811517.49	362	C14H31BO6Si2	mainlib

Faten-212 #5516 RT: 23.76 AV: 1 RF: 6.00, 3 NL: 2.10E4
F: {0,0} + c EI Full ms [40.00-800.00]

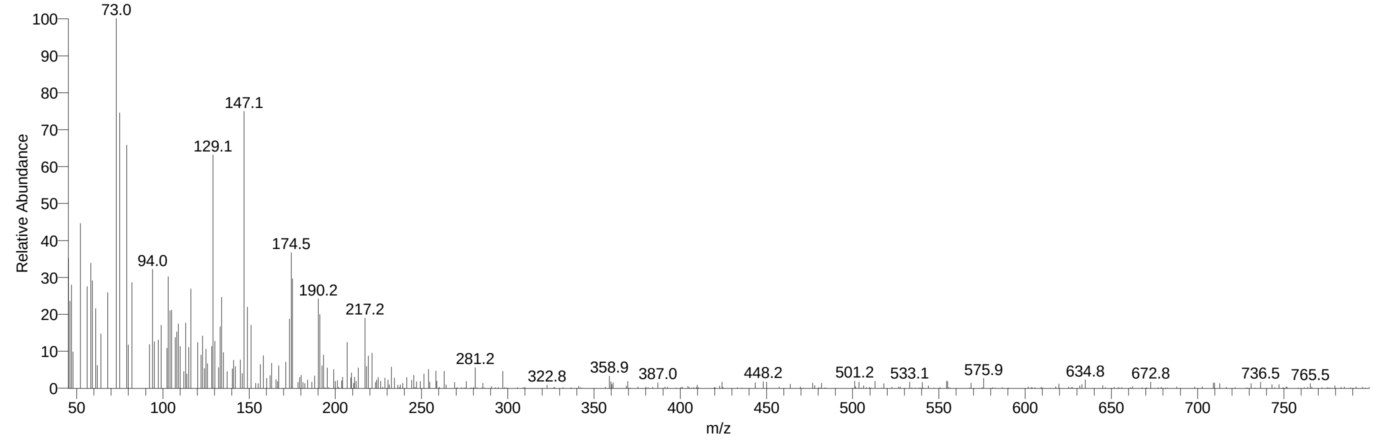

Hit Spectrum | Delta | Compound Structure

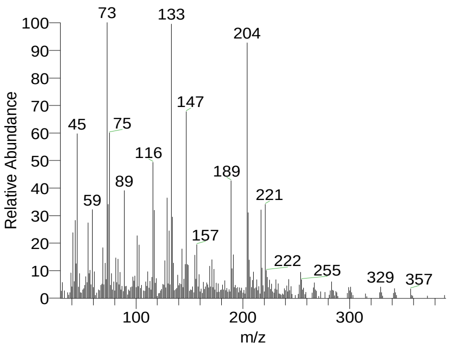

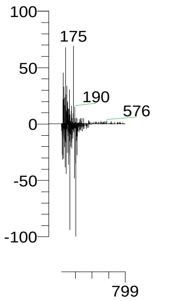

á-D-Galactopyranoside, methyl 2,3-bis-O-(trimethylsilyl)-, cyclic butylboronate
Formula C17H37BO6Si2, MW 404, CAS# 56211-10-0, Entry# 38594

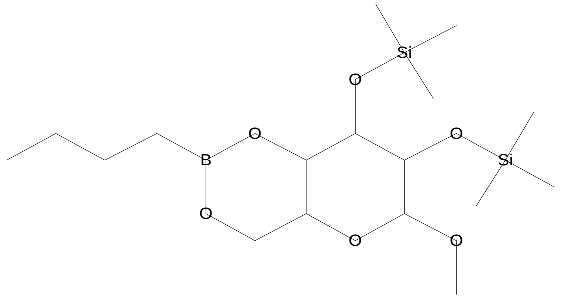

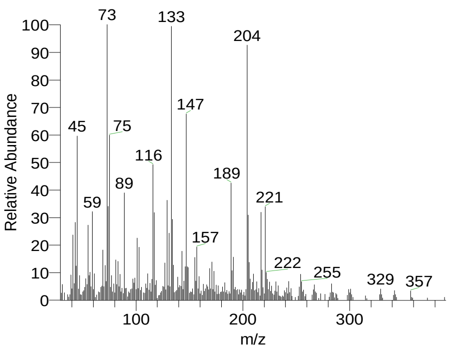

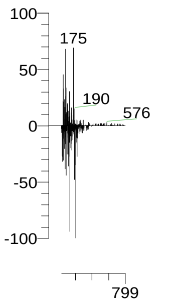

á-D-Galactopyranoside, methyl 2,3-bis-O-(trimethylsilyl)-, cyclic butylboronate (CAS)
Formula C17H37BO6Si2, MW 404, CAS# 56211-10-0, Entry# 546606
B-GALACTOPYRANOSIDE-1-METHYL-4,6-BUTYLBORONATE-2,3-DITMS

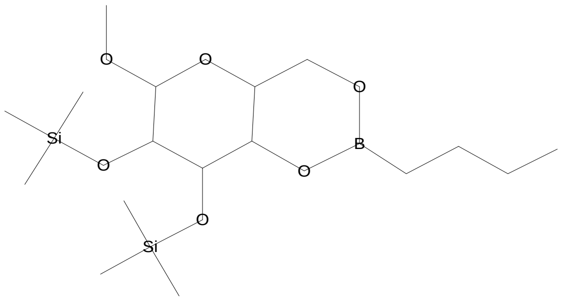

There is no signature data to report.

Library Search Report

Hit Spectrum	Delta	Compound Structure

à-D-Galactopyranoside, methyl 2,3-bis-O-(trimethylsilyl)-, cyclic methylboronate
Formula C14H31BO6Si2, MW 362, CAS# 54400-88-3, Entry# 38593

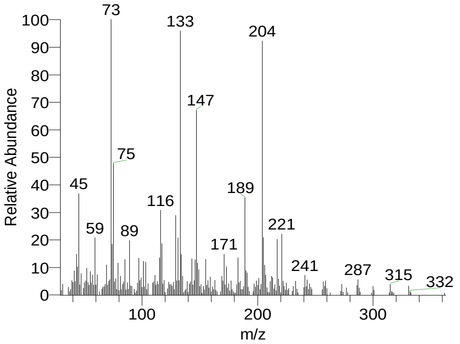

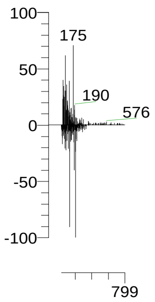

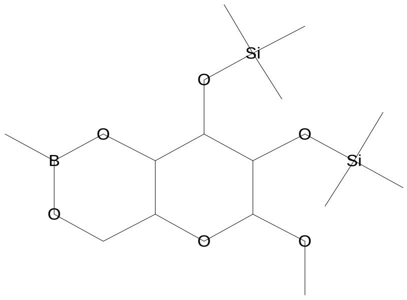

There is no signature data to report.

Library Search Report

RT	Probability	Compound Name	SI	Area %	Area	Molecular Weight	Molecular Formula	Library
24.11	60.83	1,1,1-Tris(hydroxymethyl)propane, tris(trimethylsilyl) ether	683	1.80	5721645.07	350	C15H38O3Si3	mainlib
24.11	4.92	3,6-Dioxa-2,7-disilaoctane, 2,2,7,7-tetramethyl- (CAS)	588	1.80	5721645.07	206	C8H22O2Si2	Wiley9
24.11	4.92	3,6-Dioxa-2,7-disilaoctane, 2,2,7,7-tetramethyl- (CAS)	582	1.80	5721645.07	206	C8H22O2Si2	Wiley9

Faten-212 #5620 RT: 24.11 AV: 1 RF: 6.00, 3 NL: 9.91E4
F: {0,0} + c EI Full ms [40.00-800.00]

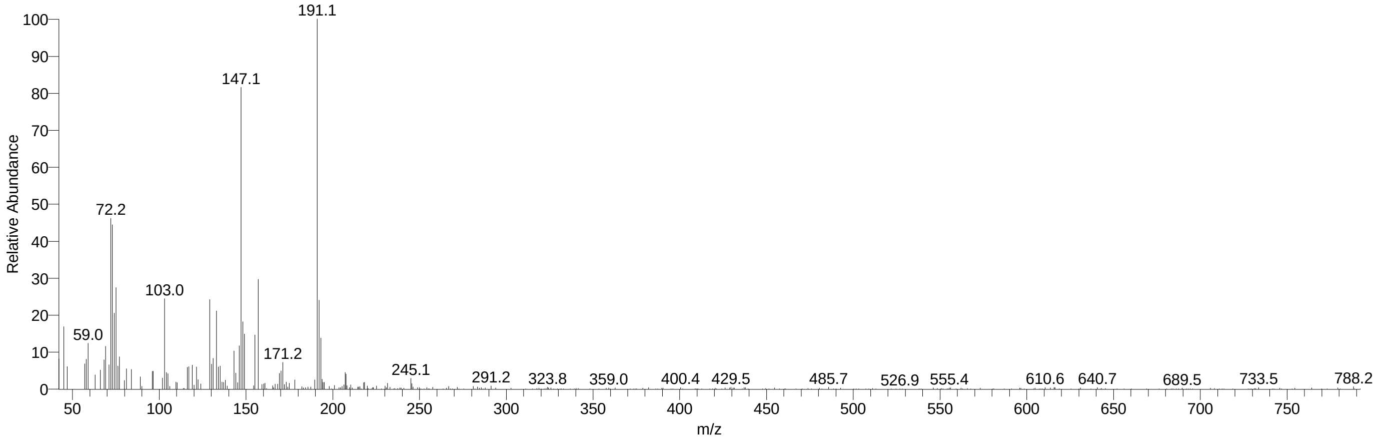

Hit Spectrum | Delta | Compound Structure

1,1,1-Tris(hydroxymethyl)propane, tris(trimethylsilyl) ether
Formula C15H38O3Si3, MW 350, CAS# NA, Entry# 156424

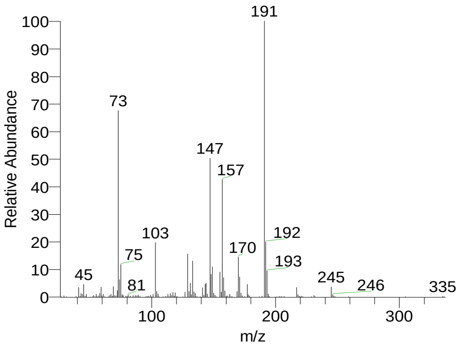

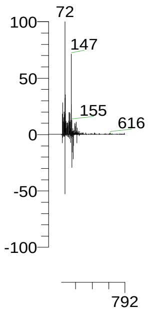

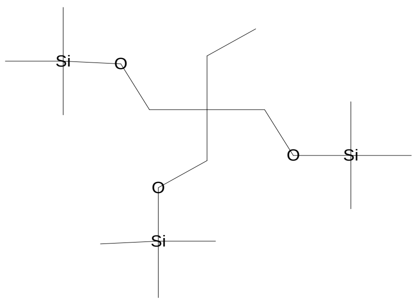

3,6-Dioxa-2,7-disilaoctane, 2,2,7,7-tetramethyl- (CAS)
Formula C8H22O2Si2, MW 206, CAS# 7381-30-8, Entry# 154602
ETHYLENE GLYCOL BISTRIMETHYLSILYL ETHER

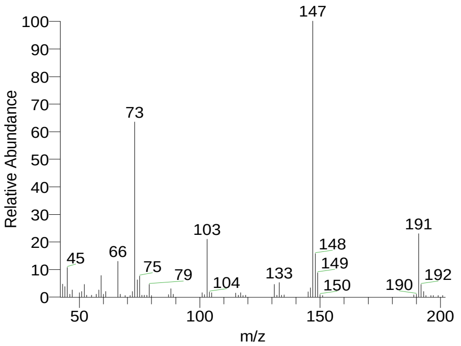

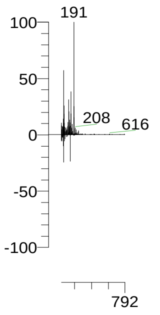

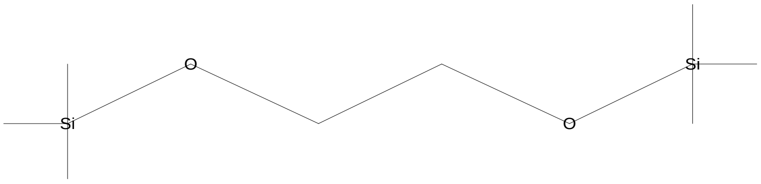

There is no signature data to report.

Library Search Report

Hit Spectrum | Delta | Compound Structure

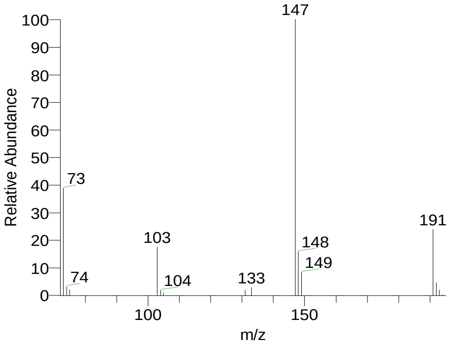

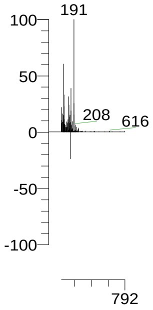

3,6-Dioxa-2,7-disilaoctane, 2,2,7,7-tetramethyl- (CAS)
Formula C8H22O2Si2, MW 206, CAS# 7381-30-8, Entry# 154607
ETHYLENE GLYCOL BISTRIMETHYLSILYL ETHER

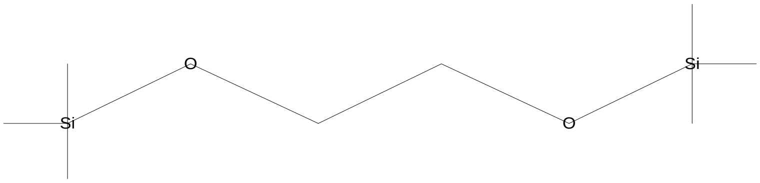

There is no signature data to report.

Library Search Report

RT	Probability	Compound Name	SI	Area %	Area	Molecular Weight	Molecular Formula	Library
24.37	7.37	7a-Isopropenyl-4,5-dimethyloctahydroindene-4-carboxylic acid	425	0.22	703282.39	236	C15H24O2	mainlib
24.37	7.37	7a-Isopropenyl-4,5-dimethyloctahydroindene-4-carboxylic acid	425	0.22	703282.39	236	C15H24O2	Wiley9
24.37	5.20	2-AMINO-3-CYANO-6-METHYL-4,5,6,7-TETRAHYDROPYRIDO(3,4-B)THIOPHENE	415	0.22	703282.39	193	C9H11N3S	Wiley9

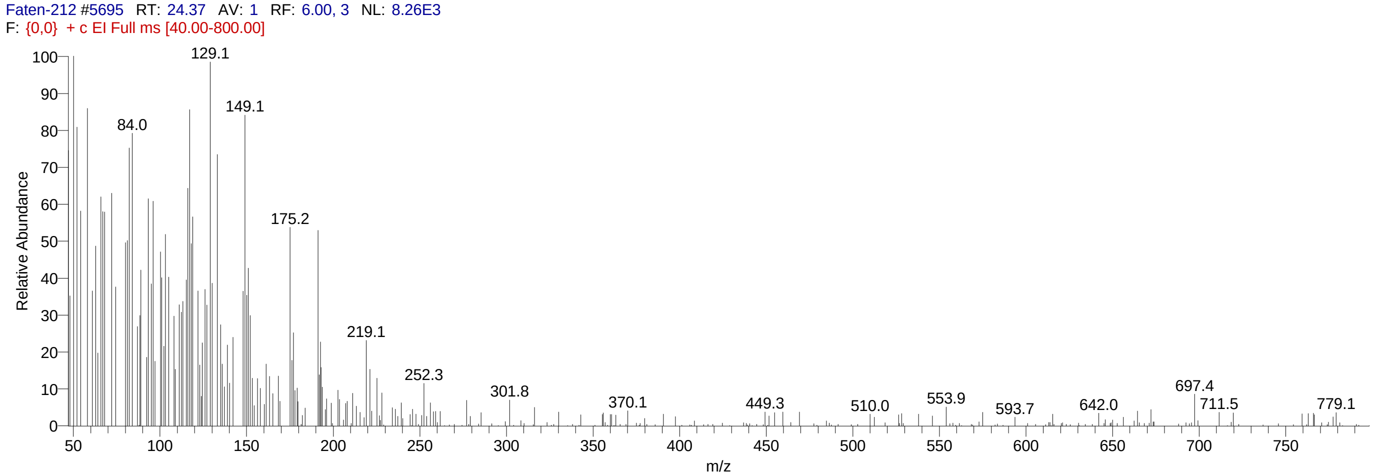

Hit Spectrum | Delta | Compound Structure

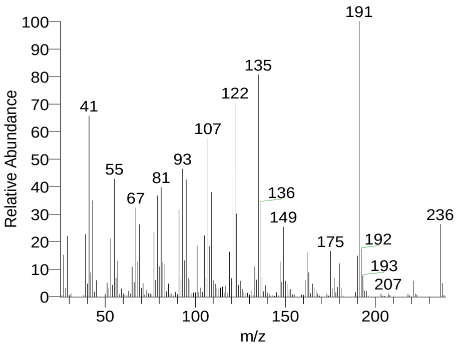

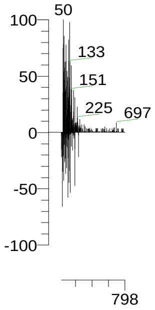

7a-Isopropenyl-4,5-dimethyloctahydroindene-4-carboxylic acid
Formula C15H24O2, MW 236, CAS# NA, Entry# 156606
7a-Isopropenyl-4,5-dimethyloctahydro-1H-indene-4-carboxylic acid #

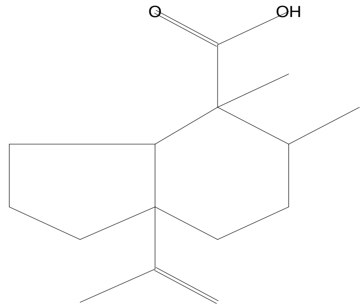

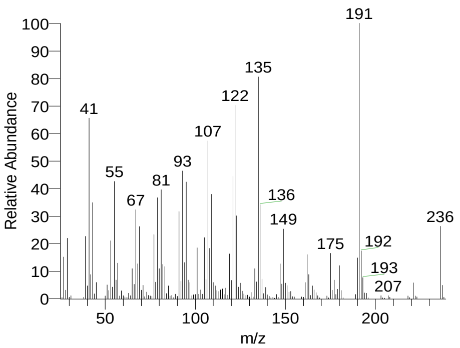

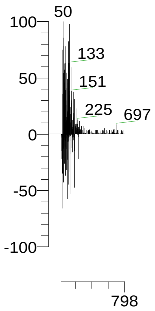

7a-Isopropenyl-4,5-dimethyloctahydroindene-4-carboxylic acid
Formula C15H24O2, MW 236, CAS# NA, Entry# 226886
7A-ISOPROPENYL-4,5-DIMETHYL-OCTAHYDRO-INDENE-4-CARBOXYLIC ACID

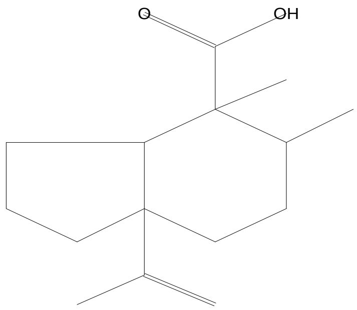

There is no signature data to report.

Library Search Report

Hit Spectrum	Delta	Compound Structure

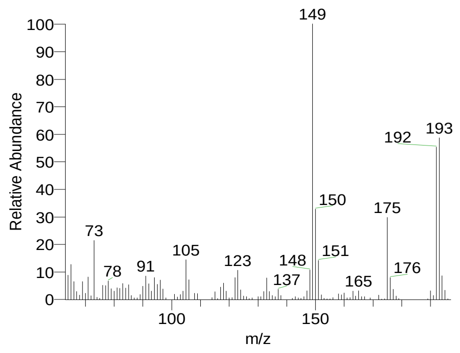

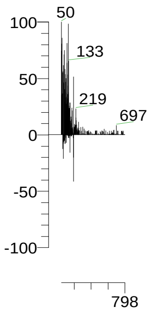

2-AMINO-3-CYANO-6-METHYL-4,5,6,7-TETRAHYDROPYRIDO(3,4-B)THIOPHENE
Formula C9H11N3S, MW 193, CAS# 37578-06-6, Entry# 126780
2-AMINO-6-METHYL-4,5,6,7-TETRAHYDROTHIENO[2,3-C]PYRIDINE-3-CARBONITRILE

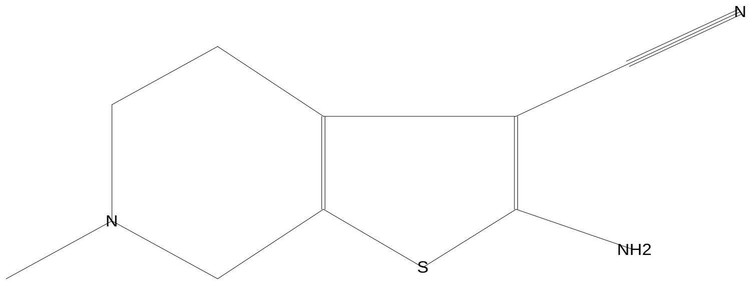

There is no signature data to report.

Library Search Report

RT	Probability	Compound Name	SI	Area %	Area	Molecular Weight	Molecular Formula	Library
24.60	10.96	3,20-Dioxo-11-à-hydroxyconanine-1,4-diene	412	0.13	415483.86	341	C21H27NO3	Wiley9
24.60	7.95	2-Acetyl-3-(2-benzenesulphonamido)ethyl-7-methoxyindole	403	0.13	415483.86	372	C19H20N2O4S	Wiley9
24.60	7.65	5à,17à-Dihydroxy-1-oxo-6à, 7 alpha.-Epoxy(22R)-witha-2,24-dienolide	402	0.13	415483.86	470	C28H38O6	Wiley9

Faten-212 #5764 RT: 24.60 AV: 1 RF: 6.00, 3 NL: 1.35E4
F: {0,0} + c EI Full ms [40.00-800.00]

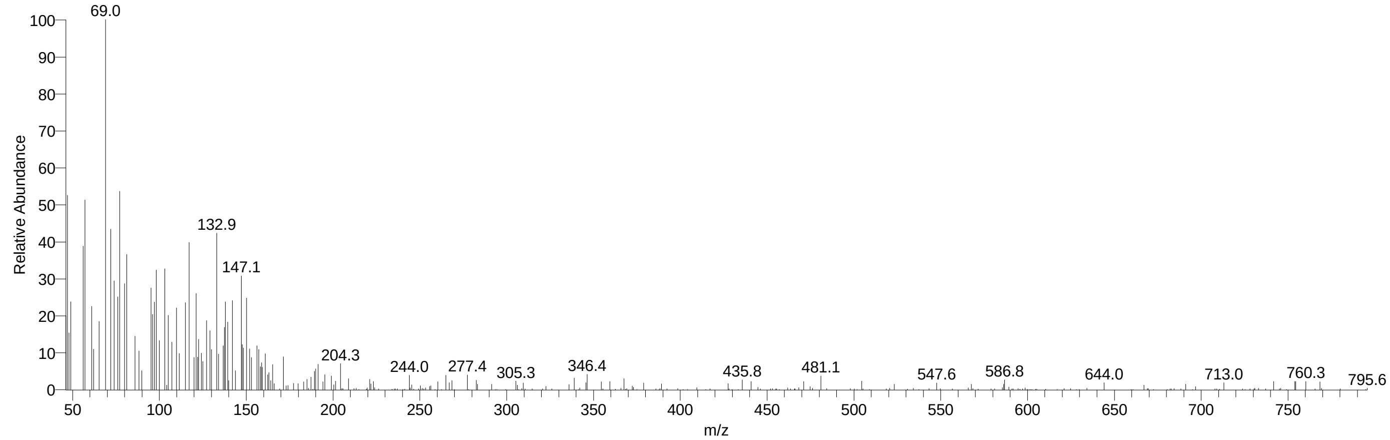

Hit Spectrum	Delta	Compound Structure

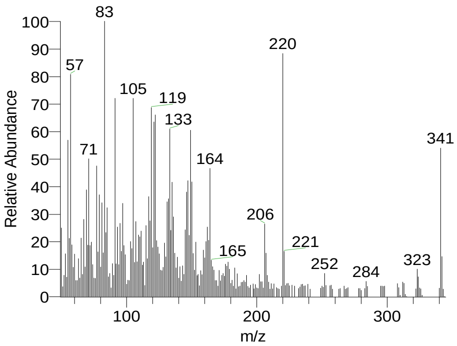

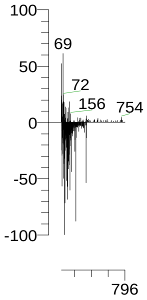

3,20-Dioxo-11-à-hydroxyconanine-1,4-diene
Formula C21H27NO3, MW 341, CAS# NA, Entry# 456515

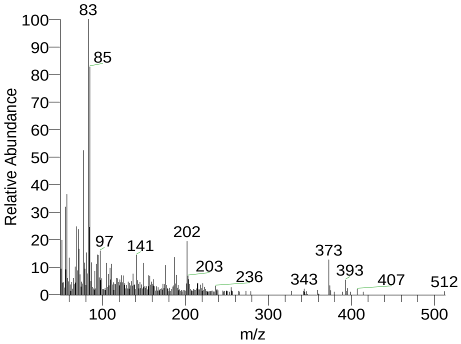

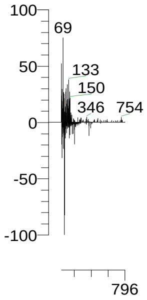

2-Acetyl-3-(2-benzenesulphonamido)ethyl-7-methoxyindole
Formula C19H20N2O4S, MW 372, CAS# NA, Entry# 505973

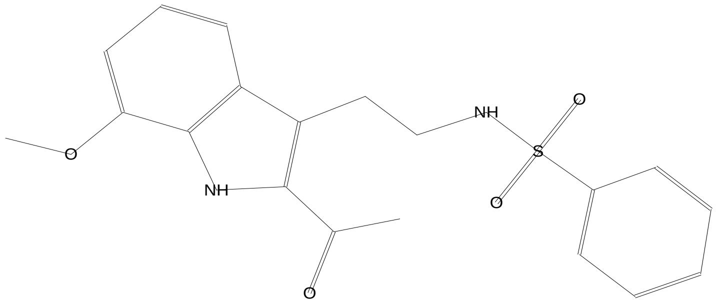

There is no signature data to report.

Library Search Report

Hit Spectrum	Delta	Compound Structure

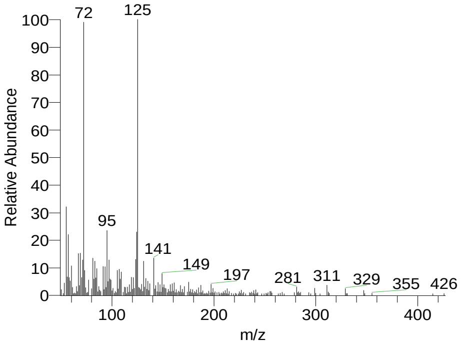

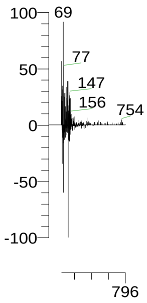

5à,17à-Dihydroxy-1-oxo-6à, 7 alpha.-Epoxy (22R)-witha-2,24-dienolide
Formula C28H38O6, MW 470, CAS# NA, Entry# 602407
5à,17à-DIHYDROXY-1-OXO-6à, 7 ALPHA.-EPOXY (22R)-WITHA-2,24-DIENOLIDE

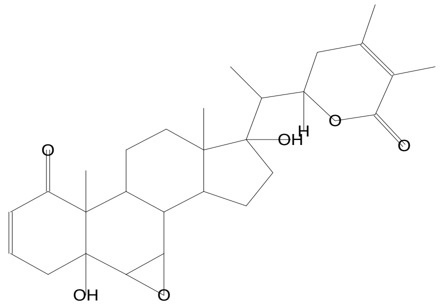

There is no signature data to report.

Library Search Report

RT	Probability	Compound Name	SI	Area %	Area	Molecular Weight	Molecular Formula	Library
24.73	8.15	Phenol, 4-(1,1-dimethylethyl)-2,6-dinitro- (CAS)	417	0.20	634161.36	240	C10H12N2O5	Wiley9
24.73	5.26	9,12,15-Octadecatrienoic acid, 2-[(trimethylsilyl)oxy]-1-[[(trimethylsilyl)oxy]methyl]ethyl ester, (Z,Z,Z)-	404	0.20	634161.36	496	C27H52O4Si2	mainlib
24.73	5.26	9,12,15-Octadecatrienoic acid, 2-[(trimethylsilyl)oxy]-1-[[(trimethylsilyl)oxy]methyl]ethyl ester, (Z,Z,Z)- (CAS)	404	0.20	634161.36	496	C27H52O4Si2	Wiley9

Faten-212 #5803 RT: 24.73 AV: 1 RF: 6.00, 3 NL: 5.02E4
F: {0,0} + c EI Full ms [40.00-800.00]

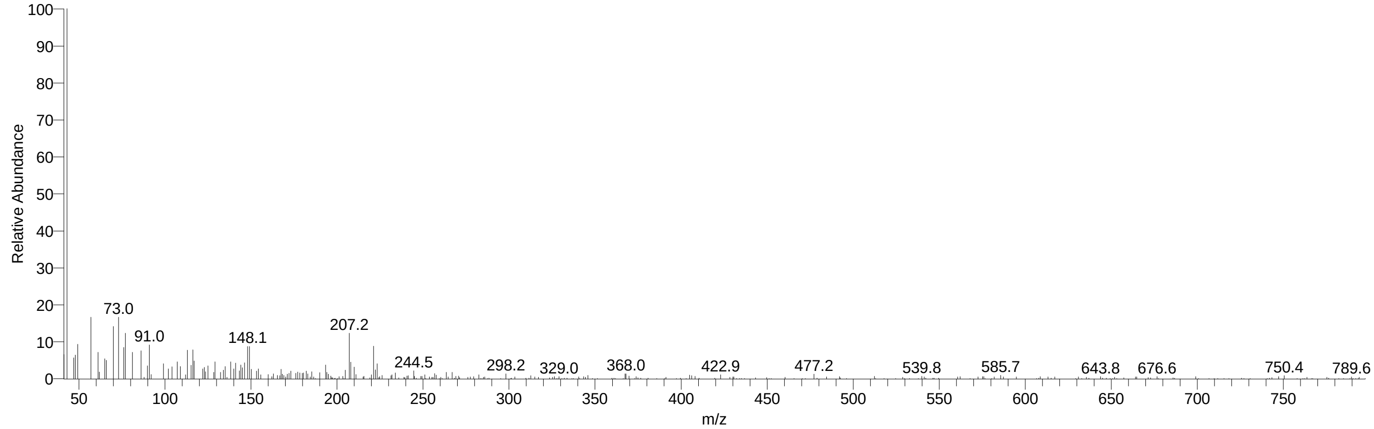

Hit Spectrum | Delta | Compound Structure

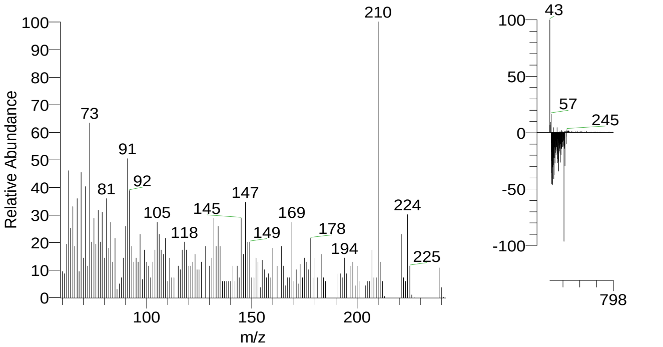

Phenol, 4-(1,1-dimethylethyl)-2,6-dinitro- (CAS)
Formula C10H12N2O5, MW 240, CAS# 4097-49-8, Entry# 234995
2,6-DINITRO-4-T-BUTYLPHENOL

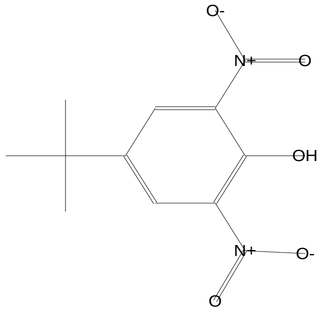

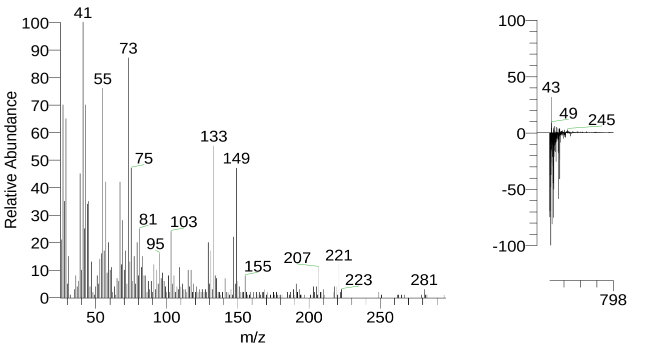

9,12,15-Octadecatrienoic acid, 2-[(trimethylsilyl)oxy]-1-[[(trimethylsilyl)oxy]methyl]ethyl ester, (Z,Z,Z)-
Formula C27H52O4Si2, MW 496, CAS# 55521-23-8, Entry# 3361
2-[(Trimethylsilyl)oxy]-1-([(trimethylsilyl)oxy]methyl)ethyl (9E,12E,15E)-9,12,15-octadecatrienoate #

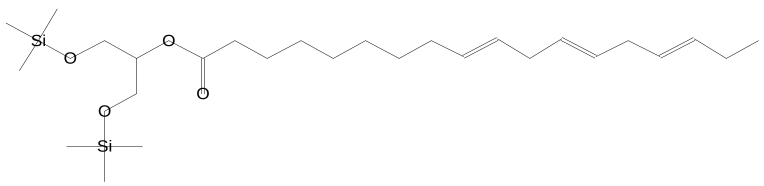

There is no signature data to report.

Library Search Report

Hit Spectrum	Delta	Compound Structure

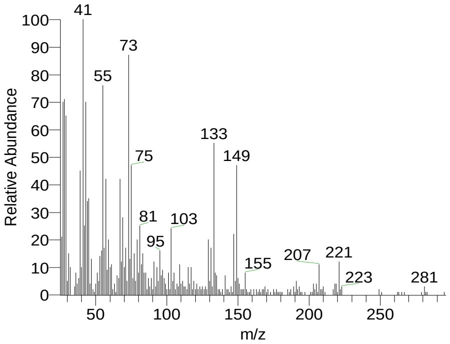

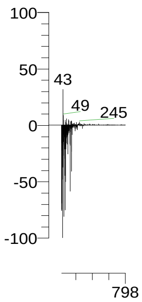

Formula C27H52O4Si2, MW 496, CAS# 55521-23-8, Entry# 616116
TRIMETHYLSILYLETHER DERIVATIVE OF 2-MONOLINOLENIN

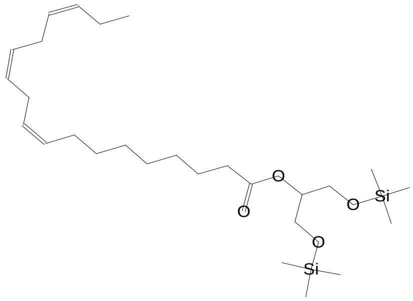

There is no signature data to report.

Library Search Report

RT	Probability	Compound Name	SI	Area %	Area	Molecular Weight	Molecular Formula	Library
25.36	22.74	10-(9-Anthrylmethyl)anthrone	453	0.17	547913.23	384	C29H20O	Wiley9
25.36	16.51	3-Fluoroquinoline-2-carboxylic acid	444	0.17	547913.23	191	C10H6FNO2	Wiley9
25.36	4.32	2-(4'-Chlorophenyl)-1-methylimidazole dev	411	0.17	547913.23	192	C10H9ClN2	Wiley9

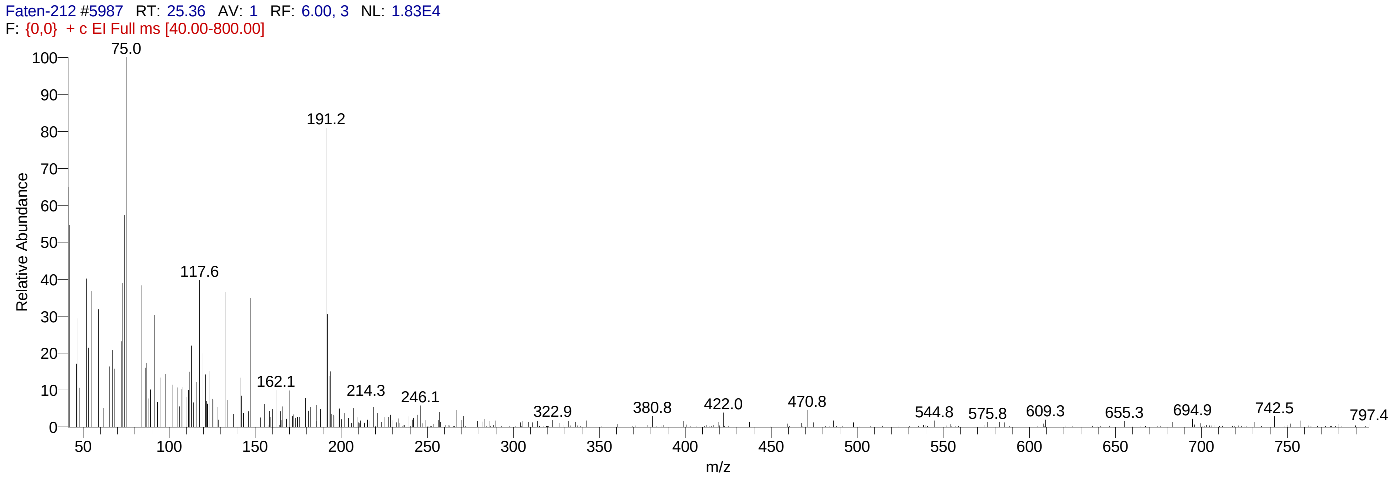

Hit Spectrum | Delta | Compound Structure

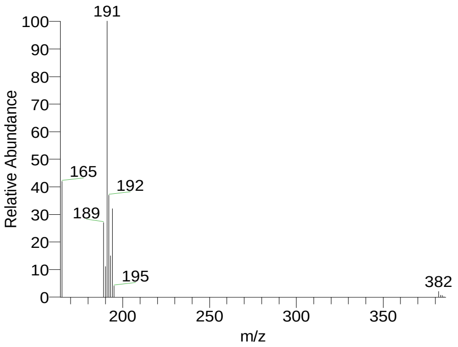

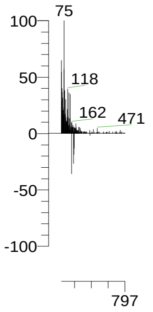

10-(9-Anthrylmethyl)anthrone
Formula C29H20O, MW 384, CAS# 68975-23-5, Entry# 523544

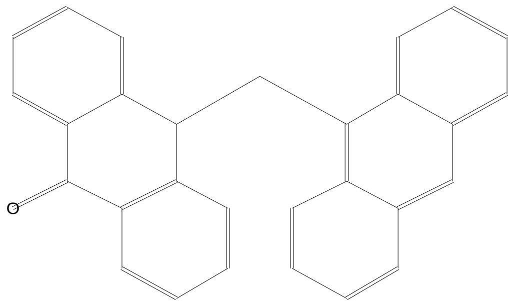

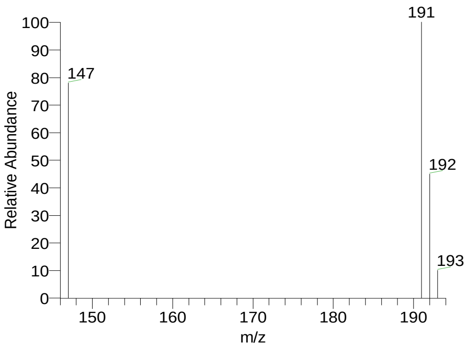

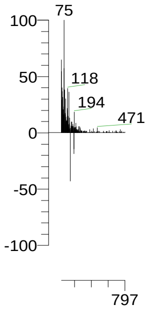

3-Fluoroquinoline-2-carboxylic acid
Formula C10H6FNO2, MW 191, CAS# NA, Entry# 122645

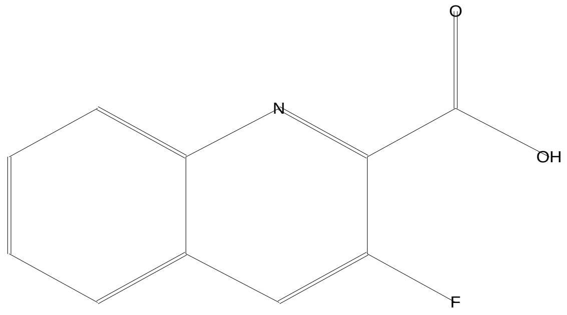

There is no signature data to report.

Library Search Report

Hit Spectrum | Delta | Compound Structure

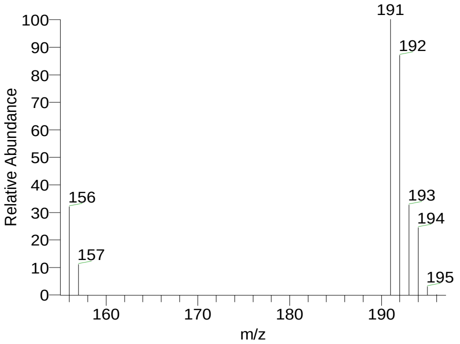

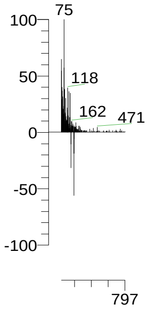

2-(4'-Chlorophenyl)-1-methylimidazole dev
Formula C10H9ClN2, MW 192, CAS# NA, Entry# 124293

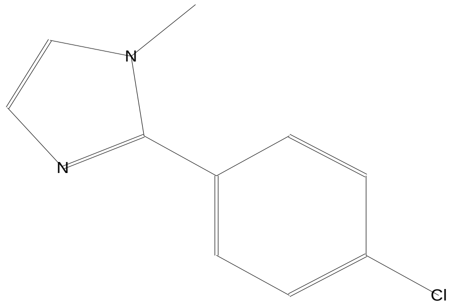

There is no signature data to report.

Library Search Report

RT	Probability	Compound Name	SI	Area %	Area	Molecular Weight	Molecular Formula	Library
25.39	13.96	5-Allyl-6-methyl-2-phenyl-5,6-dihydro-4H-oxazolo[4,5-c]pyridin-7-one	400	0.17	551581.58	268	C16H16N2O2	mainlib
25.39	10.98	1,2-Nonadecanediol (CAS)	394	0.17	551581.58	300	C19H40O2	Wiley9
25.39	6.65	13-Oxabicyclo[10.1.0]tridecane (CAS)	379	0.17	551581.58	182	C12H22O	Wiley9

Faten-212 #5997 RT: 25.39 AV: 1 RF: 6.00, 3 NL: 1.05E4
F: {0,0} + c EI Full ms [40.00-800.00]

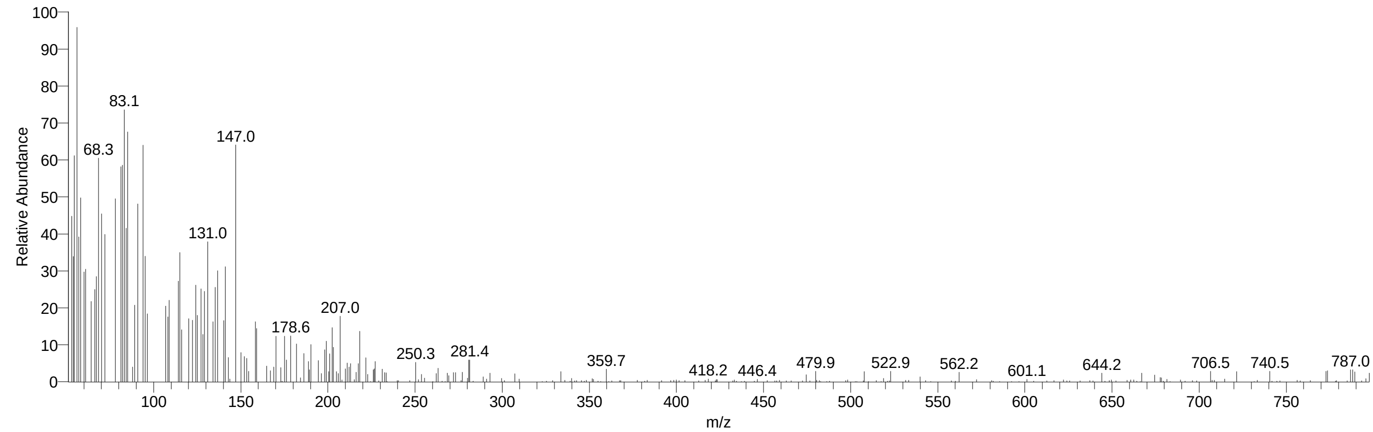

Hit Spectrum | Delta | Compound Structure

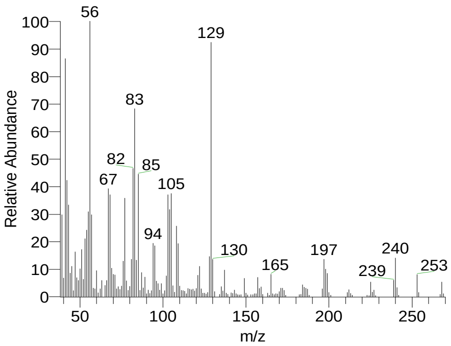

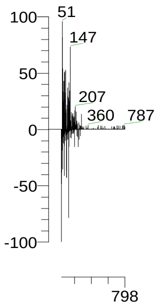

5-Allyl-6-methyl-2-phenyl-5,6-dihydro-4H-oxazolo[4,5-c]pyridin-7-one
Formula C16H16N2O2, MW 268, CAS# NA, Entry# 21231
5-Allyl-6-methyl-2-phenyl-5,6-dihydro[1,3]oxazolo[4,5-c]pyridin-7(4H)-one #

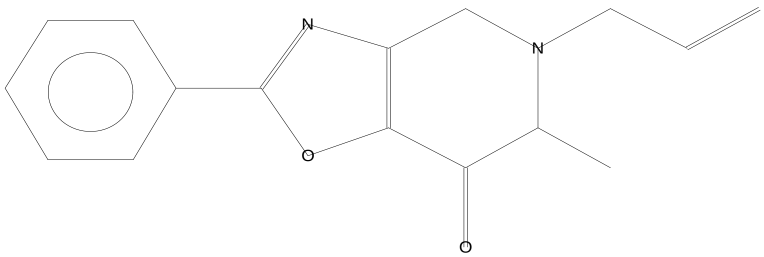

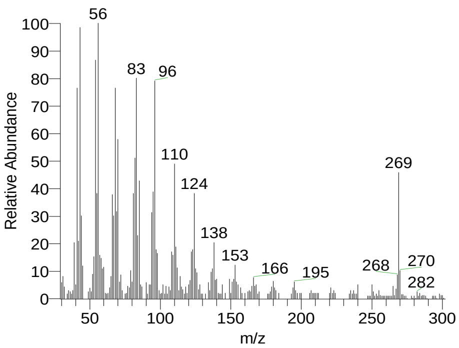

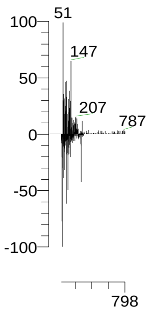

1,2-Nonadecanediol (CAS)
Formula C19H40O2, MW 300, CAS# 39516-65-9, Entry# 375295
Nonadecanediol-1,2

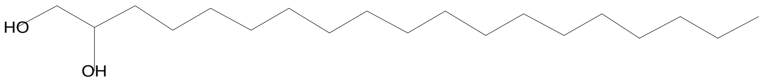

There is no signature data to report.

Library Search Report

Hit Spectrum	Delta	Compound Structure

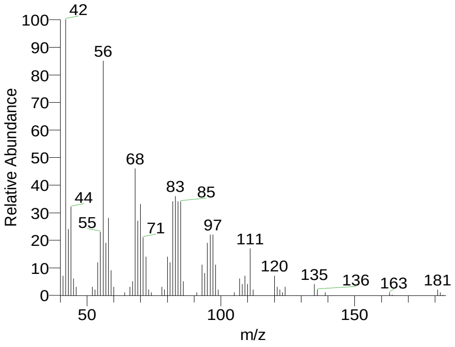

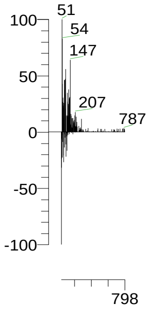

13-Oxabicyclo[10.1.0]tridecane (CAS)
Formula C12H22O, MW 182, CAS# 286-99-7, Entry# 106775
Epoxycyclododecane

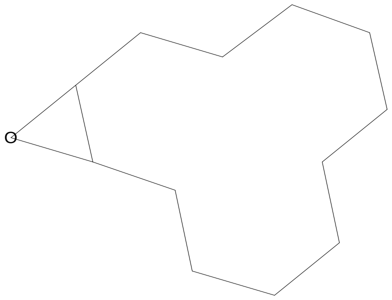

There is no signature data to report.

Library Search Report

RT	Probability	Compound Name	SI	Area %	Area	Molecular Weight	Molecular Formula	Library
26.93	83.98	1-(2'-Quinolyl)-3-methylazulene	789	0.58	1843015.42	269	C20H15N	Wiley9
26.93	11.17	12-(Methylthio)-1,2,3,4-tetrahydrobenzimidazo[1,2-b]isoquinoline	715	0.58	1843015.42	268	C16H16N2S	Wiley9
26.93	2.16	{[(2''-Phenylethynyl)cyclopent-1'-en-1'-yl]ethynyl}benzene	660	0.58	1843015.42	268	C21H16	Wiley9

Faten-212 #6449 RT: 26.93 AV: 1 RF: 6.00, 3 NL: 4.42E4
F: {0,0} + c EI Full ms [40.00-800.00]

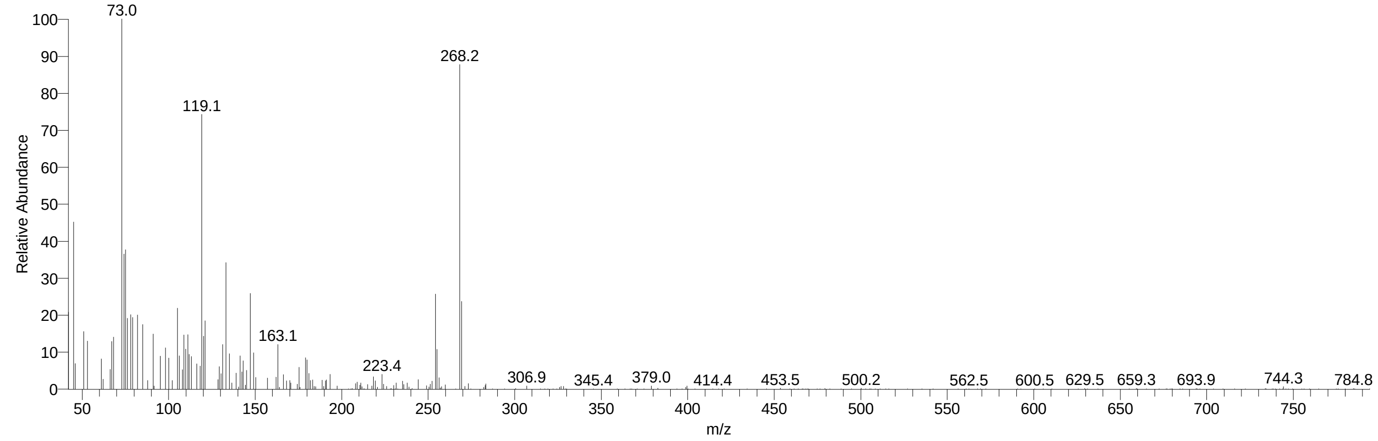

Hit Spectrum	Delta	Compound Structure

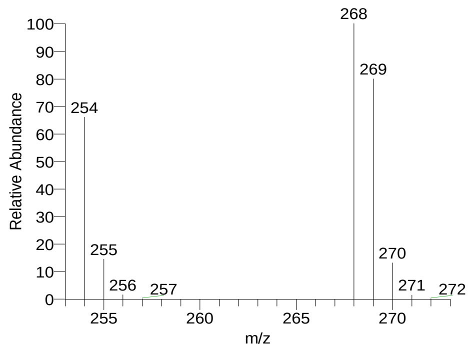

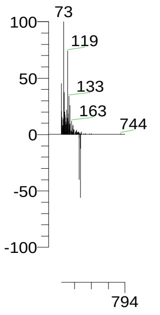

1-(2'-Quinolyl)-3-methylazulene
Formula C20H15N, MW 269, CAS# NA, Entry# 304988

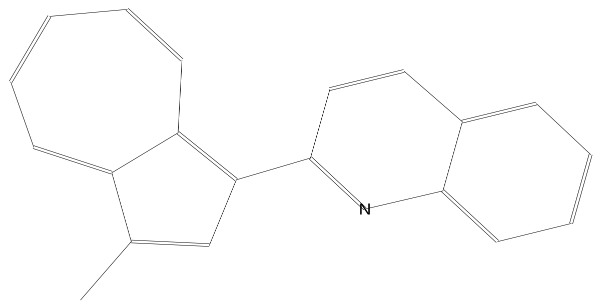

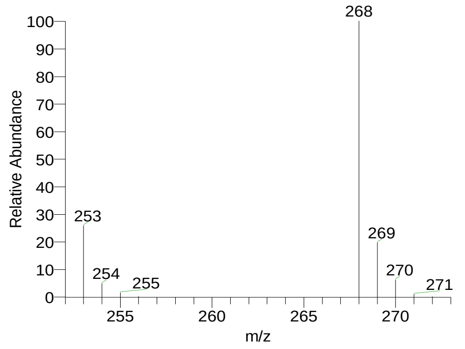

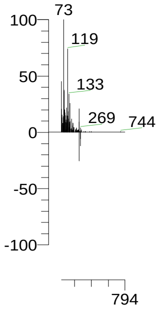

12-(Methylthio)-1,2,3,4-tetrahydrobenzimidazo[1,2-b]isoquinoline
Formula C16H16N2S, MW 268, CAS# NA, Entry# 302363
11-(Methylthio)-1,2,3,4-tetrahydrobenzimidazo[1,2-b]isoquinoline (name from MOL file)

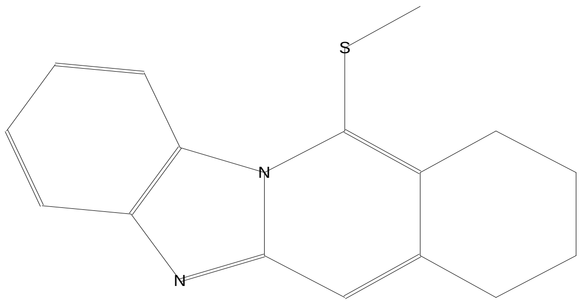

There is no signature data to report.

Library Search Report

Hit Spectrum	Delta	Compound Structure

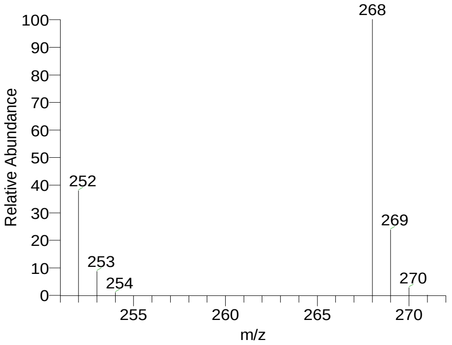

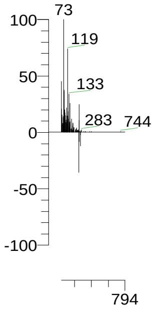

{[(2''-Phenylethynyl)cyclopent-1'-en-1'-yl]ethynyl}benzene
Formula C21H16, MW 268, CAS# NA, Entry# 303462

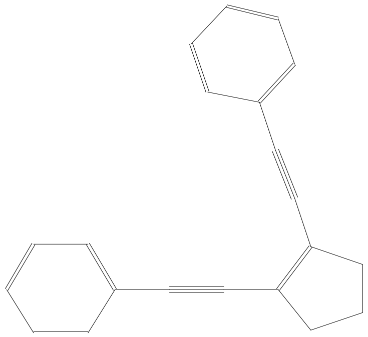

There is no signature data to report.

Library Search Report

RT	Probability	Compound Name	SI	Area %	Area	Molecular Weight	Molecular Formula	Library
26.98	6.08	Methyl 6.á-(acetyloxy)-Labda-8(17),13-dien-15-oate	329	0.14	443747.78	376	C23H36O4	Wiley9
26.98	5.13	2-(4-CHLOROPHENYL)-5-METHYL-N-[2-NITRO-4-(TRIFLUOROMETHYL)PHENYL]-1,3-DIOXAN-5-AMINE	325	0.14	443747.78	416	C18H16ClF3N2O4	Wiley9
26.98	4.54	2-(1H-BENZIMIDAZOL-2-YLSULFANYL)-N'-[(3-FLUOROPHENYL)METHYLIDENE]ACETOHYDRAZIDE	322	0.14	443747.78	328	C16H13FN4OS	Wiley9

Faten-212 #6464 RT: 26.98 AV: 1 RF: 6.00, 3 NL: 3.89E4
F: {0,0} + c EI Full ms [40.00-800.00]

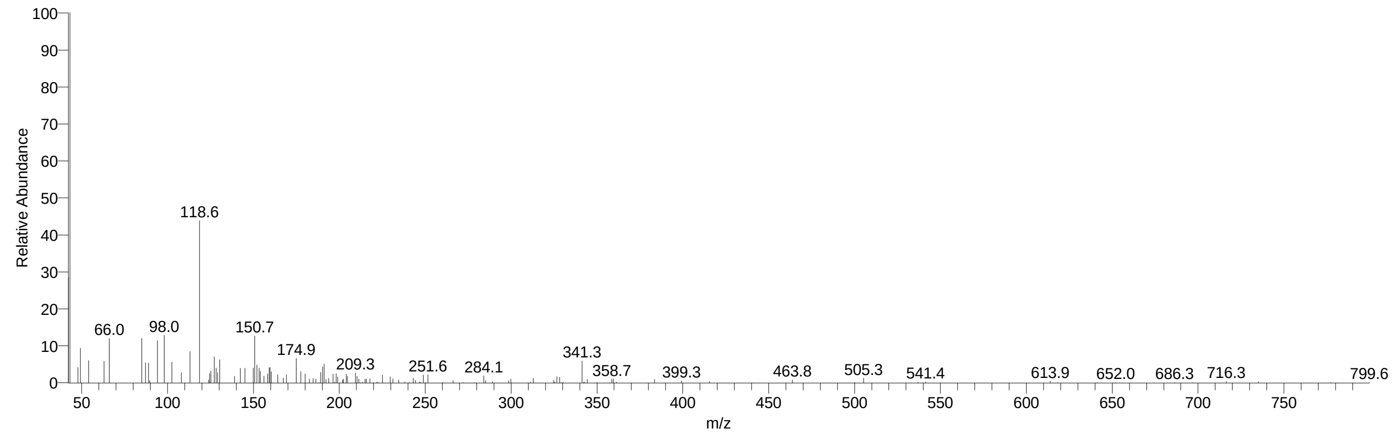

Hit Spectrum | Delta | Compound Structure

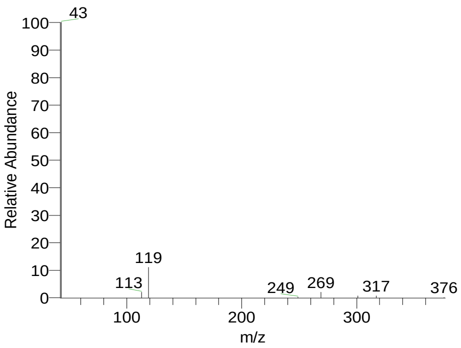

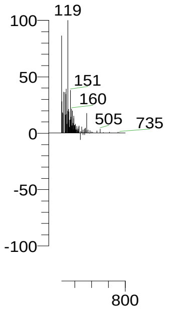

Methyl 6.á-(acetyloxy)-Labda-8(17),13-dien-15-oate
Formula C23H36O4, MW 376, CAS# NA, Entry# 512584

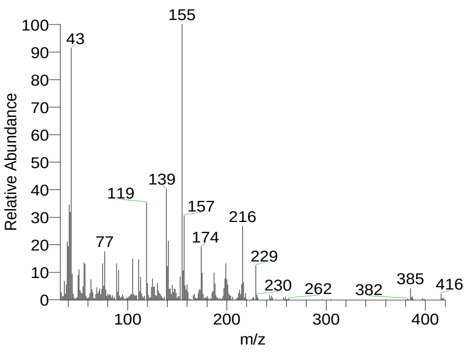

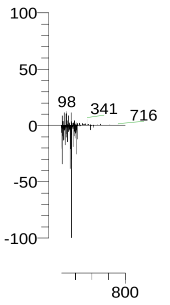

Formula C18H16ClF3N2O4, MW 416, CAS# NA, Entry# 559546

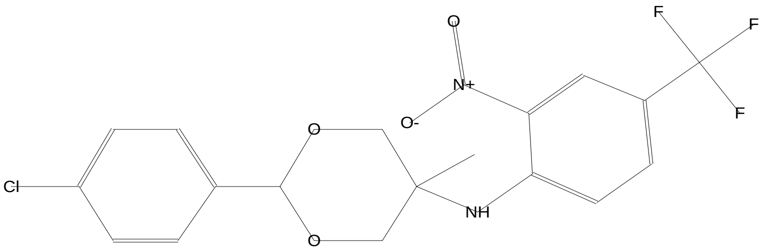

There is no signature data to report.

Library Search Report

Hit Spectrum	Delta	Compound Structure

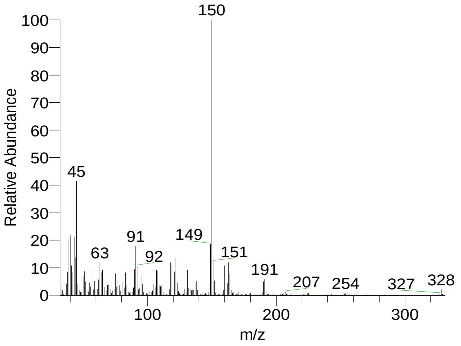

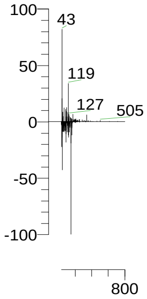

Formula C16H13FN4OS, MW 328, CAS# NA, Entry# 431447
ACETHYDRAZIDE, 2-(2-BENZIMIDAZOLYLTHIO)-, N2-(3-FLUOROBENZYLIDENO)-

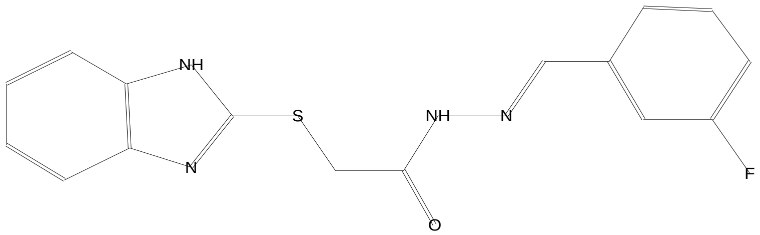

There is no signature data to report.

Library Search Report

RT	Probability	Compound Name	SI	Area %	Area	Molecular Weight	Molecular Formula	Library
27.40	91.93	N,N'-Dicyclohexyl-1,6,7-tribromoperylene-3,4:9,10-tetracarboxylic acid bisimide	631	0.14	456331.08	788	C36H27Br3N2O4	Wiley9
27.40	0.91	1,4,7-Tris(3,5-di-tert-butyl-2-deuteriohydroxybenzyl)-1,4,7-triazacyclononane	406	0.14	456331.08	783	C51H72D9N3O3	Wiley9
27.40	0.72	t-Butyl-{2-[3-(2,2-dimethyl-6-methylene-cyclohexyl)-propyl]-[1,3]dithian-2-yl}-dimethyl-silane	400	0.14	456331.08	398	C22H42S2Si	mainlib

Faten-212 #6586 RT: 27.40 AV: 1 RF: 6.00, 3 NL: 4.65E4
F: {0,0} + c EI Full ms [40.00-800.00]

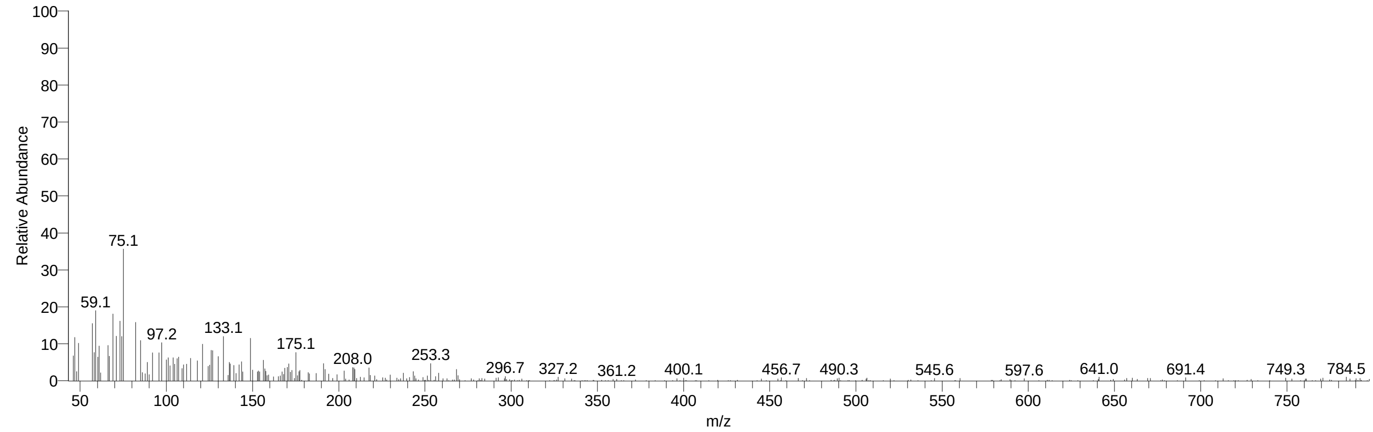

Hit Spectrum | Delta | Compound Structure

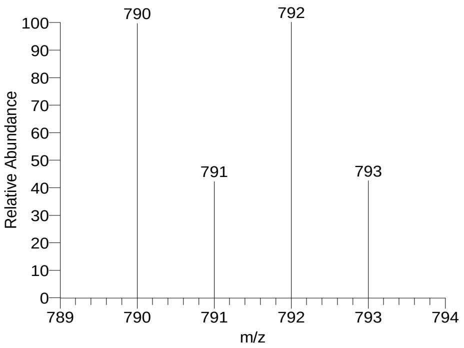

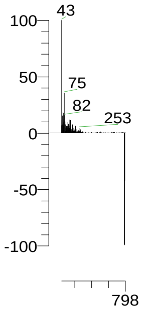

N,N'-Dicyclohexyl-1,6,7-tribromoperylene-3,4:9,10-tetracarboxylic acid bisimide
Formula C36H27Br3N2O4, MW 788, CAS# NA, Entry# 658382

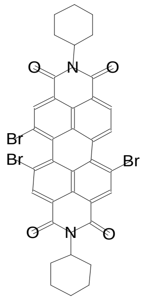

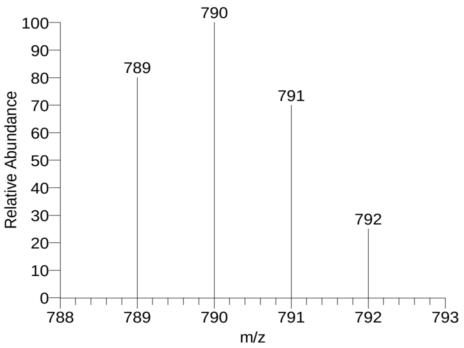

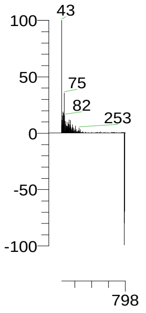

1,4,7-Tris(3,5-di-tert-butyl-2-deuteriohydroxybenzyl)-1,4,7-triazacyclononane
Formula C51H72D9N3O3, MW 783, CAS# NA, Entry# 658265

There is no signature data to report.

Library Search Report

Hit Spectrum

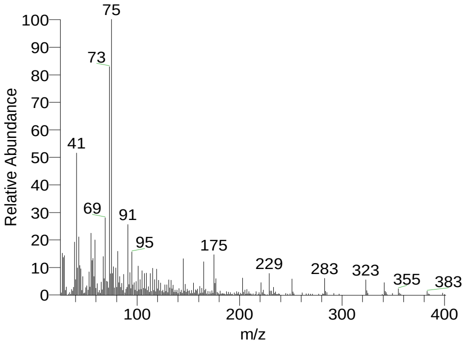

Delta

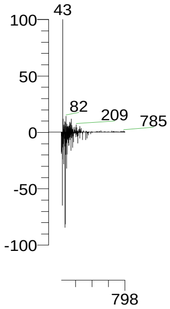

Compound Structure

t-Butyl-{2-[3-(2,2-dimethyl-6-methylene-cyclohexyl)-propyl]-[1,3]dithian-2-yl}-dimethyl-silane
Formula C22H42S2Si, MW 398, CAS# 95472-42-7, Entry# 41195
tert-Butyl(2-[3-(2,2-dimethyl-6-methylenecyclohexyl)propyl]-1,3-dithian-2-yl)dimethylsilane #

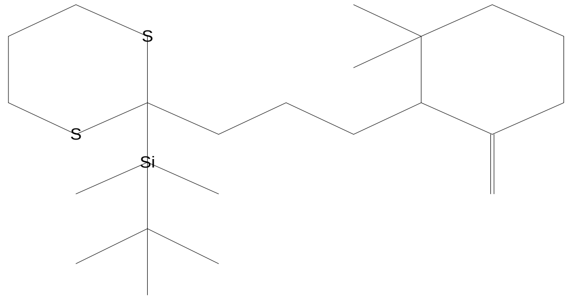

There is no signature data to report.

Library Search Report

RT	Probability	Compound Name	SI	Area %	Area	Molecular Weight	Molecular Formula	Library
27.61	12.07	TETRANEURIN - C	353	0.21	675951.28	366	C19H26O7	Wiley9
27.61	6.22	erythro-Pentitol, 2-deoxy-1,3,4,5-tetrakis-O-(trimethylsilyl)- (CAS)	335	0.21	675951.28	424	C17H44O4Si4	Wiley9
27.61	4.64	2-Deoxycytidine, tri(trimethylsilyl)-	327	0.21	675951.28	443	C18H37N3O4Si3	mainlib

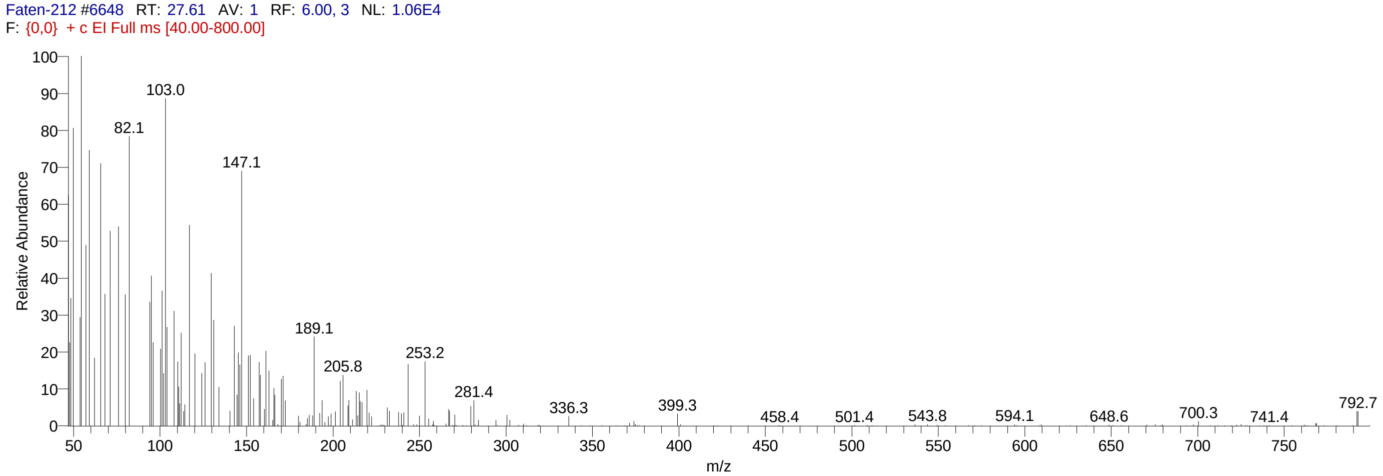

Hit Spectrum	Delta	Compound Structure

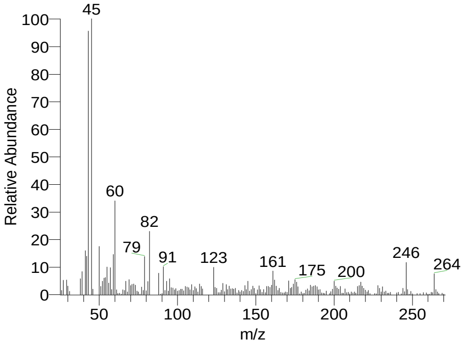

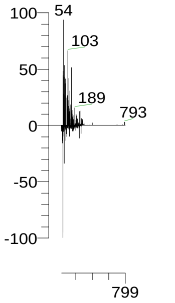

TETRANEURIN - C
Formula C19H26O7, MW 366, CAS# 28587-46-4, Entry# 496919

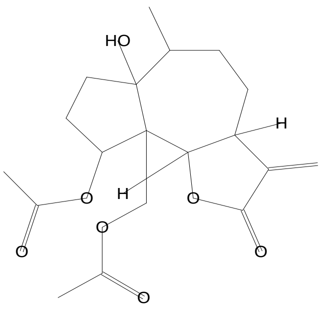

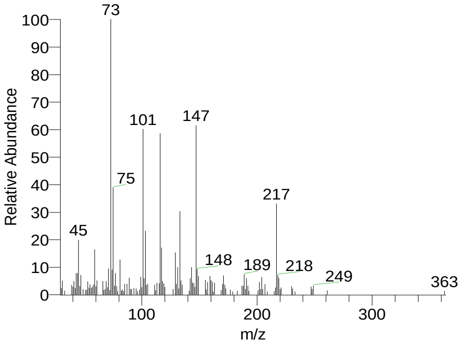

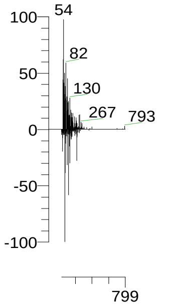

erythro-Pentitol, 2-deoxy-1,3,4,5-tetrakis-O-(trimethylsilyl)- (CAS)
Formula C17H44O4Si4, MW 424, CAS# 56271-71-7, Entry# 567197
2-DEOXYRIBITOL-1,3,4,5-TETRATMS

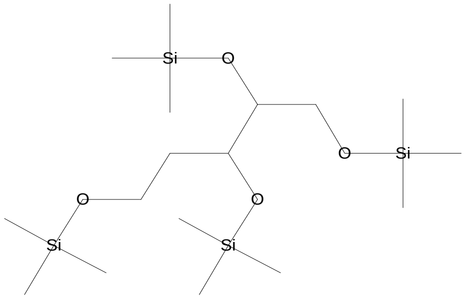

There is no signature data to report.

Library Search Report

Hit Spectrum	Delta	Compound Structure

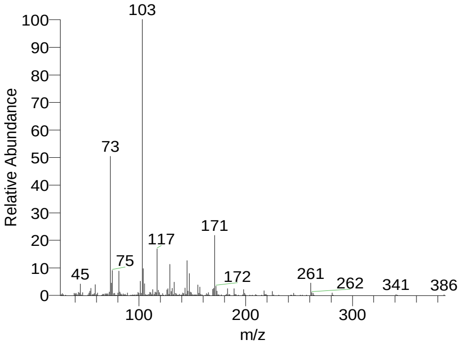

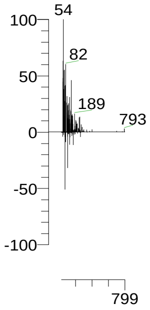

2-Deoxycytidine, tri(trimethylsilyl)-
Formula C18H37N3O4Si3, MW 443, CAS# NA, Entry# 68929

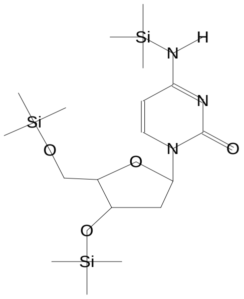

There is no signature data to report.

Library Search Report

RT	Probability	Compound Name	SI	Area %	Area	Molecular Weight	Molecular Formula	Library
27.80	81.75	1-.alpha-Methoxy-di(cholestano[2,3-b : 2',3'-e]pyrazine	614	0.16	519636.18	794	C55H90N2O	Wiley9
27.80	14.51	1-à.-Methoxy-di(cholestano[2,3-b : 3',2'-e]pyrazine	554	0.16	519636.18	794	C55H90N2O	Wiley9
27.80	0.37	Acetic acid, 10-dimethoxymethyl-13-methyl-3-oxo-4,5,6,7,8,9,10,11,12,13,14,15,16,17-tetradecahydro-3H-cyclopenta[a]phenanthren-17-yl (ester)	398	0.16	519636.18	390	C23H34O5	mainlib

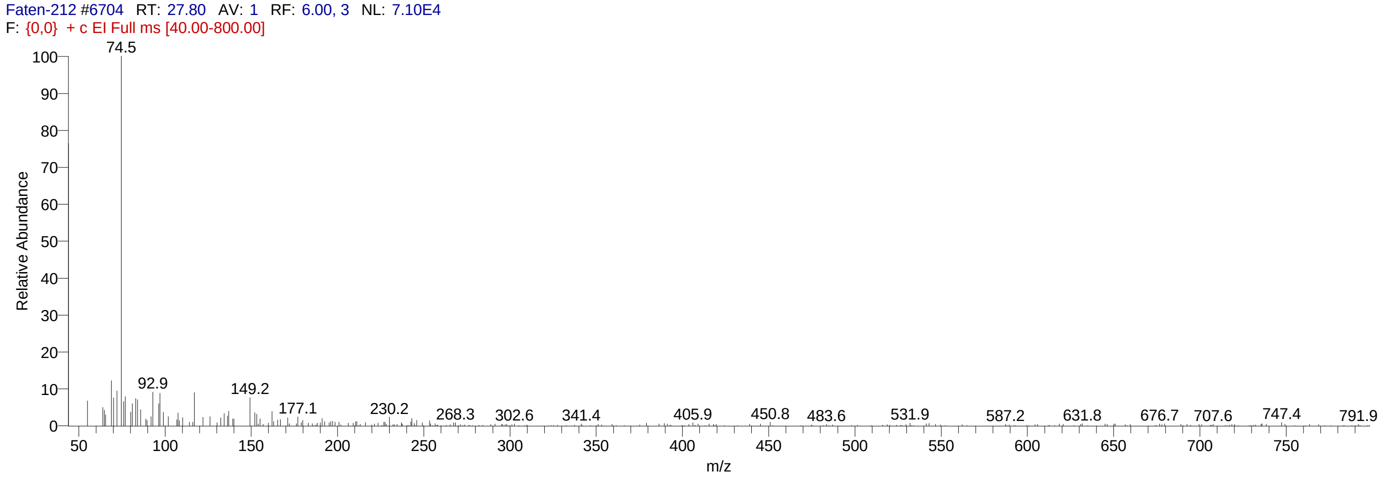

Hit Spectrum	Delta	Compound Structure

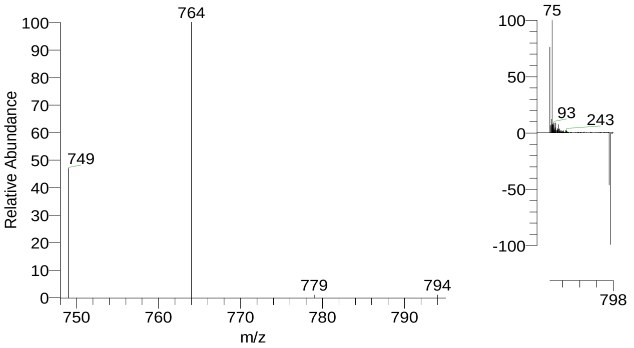

1-.alpha-Methoxy-di(cholestano[2,3-b : 2',3'-e]pyrazine
Formula C55H90N2O, MW 794, CAS# NA, Entry# 658597

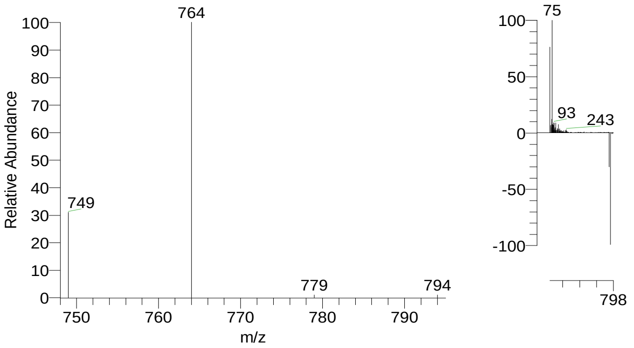

1-à.-Methoxy-di(cholestano[2,3-b : 3',2'-e]pyrazine
Formula C55H90N2O, MW 794, CAS# NA, Entry# 658595

There is no signature data to report.

Library Search Report

Hit Spectrum | Delta | Compound Structure

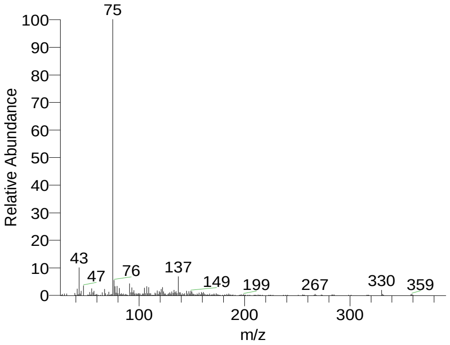

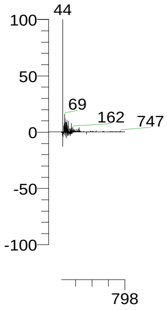

Formula C23H34O5, MW 390, CAS# NA, Entry# 41049
19,19-Dimethoxy-3-oxoandrost-1-en-17-yl acetate #

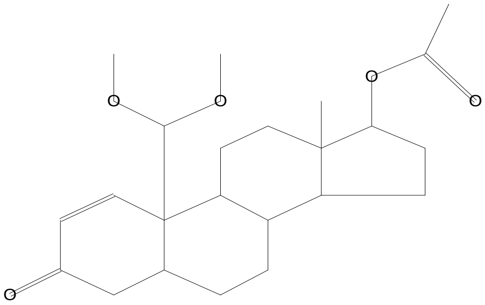

There is no signature data to report.

Library Search Report

RT	Probability	Compound Name	SI	Area %	Area	Molecular Weight	Molecular Formula	Library
28.39	15.05	2,2'-Dibromo-5,5'-bis(4-biphenylyl)-4,4'-di-tert-butylbiphenyl	349	0.23	733172.42	726	C44H40Br2	Wiley9
28.39	13.88	Methoxytrimethylgermanium	347	0.23	733172.42	150	C4H12GeO	mainlib
28.39	8.96	medrol acetate	334	0.23	733172.42	416	C24H32O6	Wiley9

Faten-212 #6879 RT: 28.39 AV: 1 RF: 6.00, 3 NL: 7.82E3
F: {0,0} + c EI Full ms [40.00-800.00]

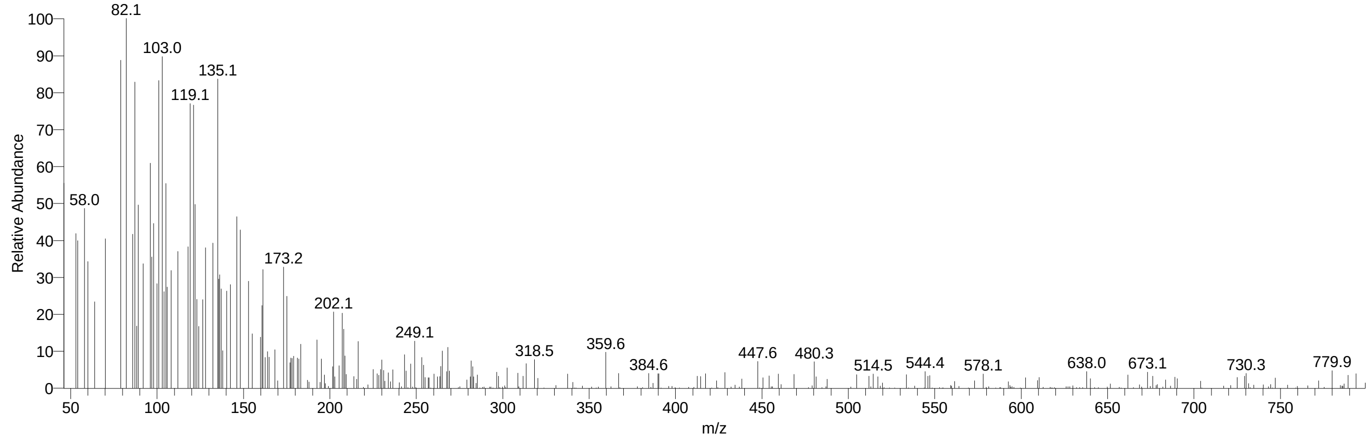

Hit Spectrum | Delta | Compound Structure

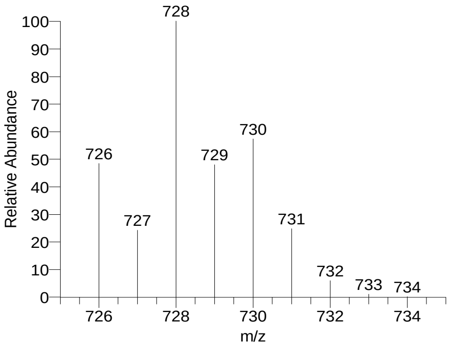

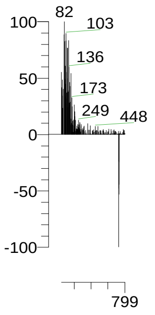

2,2'-Dibromo-5,5'-bis(4-biphenylyl)-4,4'-di-tert-butylbiphenyl
Formula C44H40Br2, MW 726, CAS# NA, Entry# 656129

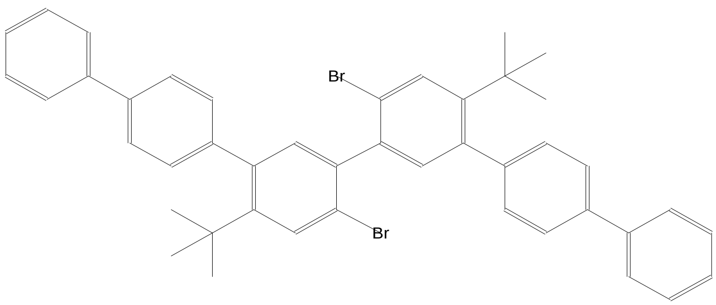

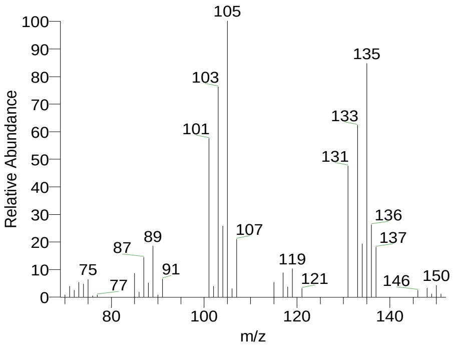

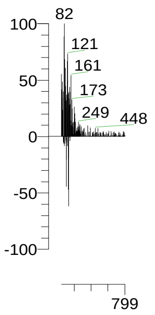

Methoxytrimethylgermanium
Formula C4H12GeO, MW 150, CAS# 6163-67-3, Entry# 73145
Methoxy(trimethyl)germane #

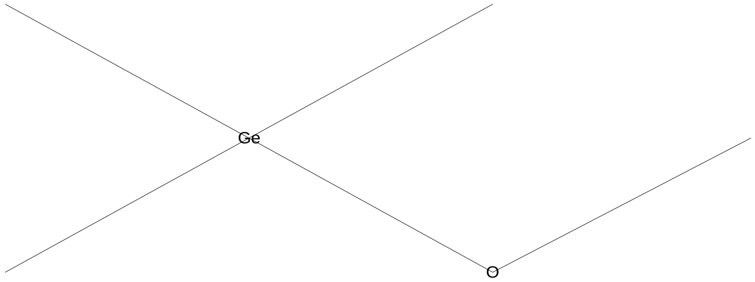

There is no signature data to report.

Library Search Report

Hit Spectrum	Delta	Compound Structure

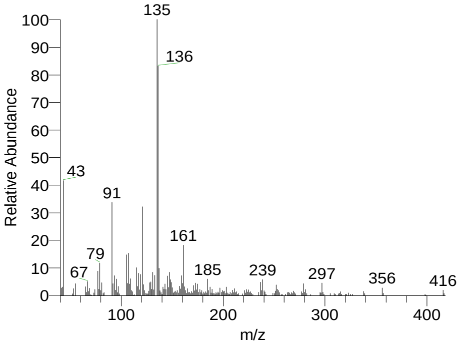

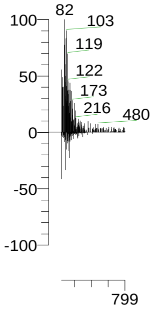

medrol acetate
Formula C24H32O6, MW 416, CAS# 53-36-1, Entry# 560229
Methylprednisolone Acetate

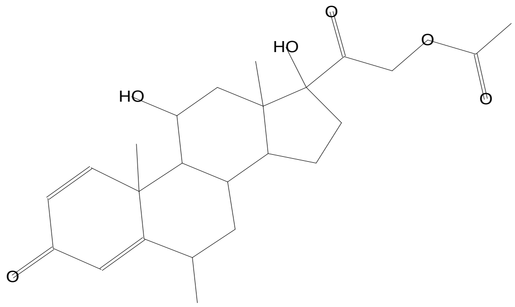

There is no signature data to report.

Library Search Report

RT	Probability	Compound Name	SI	Area %	Area	Molecular Weight	Molecular Formula	Library
28.49	13.71	3-Oxo-20-methyl-11-à-hydroxyconanine-1,4-diene	450	0.48	1514889.24	341	C22H31NO2	Wiley9
28.49	7.48	2-Acetyl-3-(2-cinnamido)ethyl-7-methoxyindole	433	0.48	1514889.24	362	C22H22N2O3	Wiley9
28.49	7.48	QUERCETIN 7,3',4'-TRIMETHOXY	433	0.48	1514889.24	344	C18H16O7	Wiley9

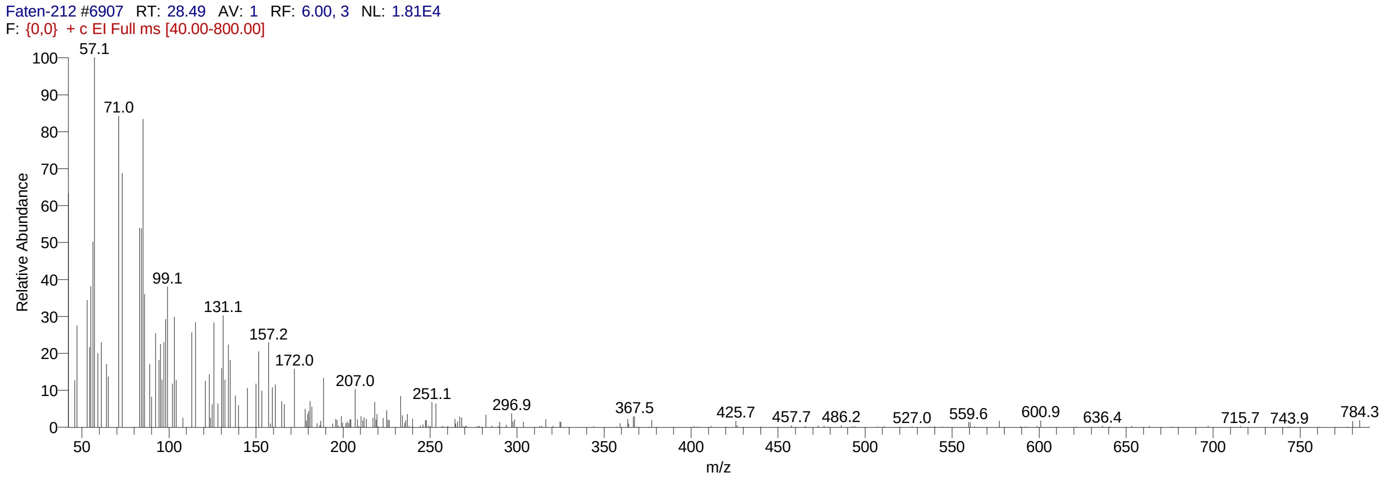

Hit Spectrum	Delta	Compound Structure

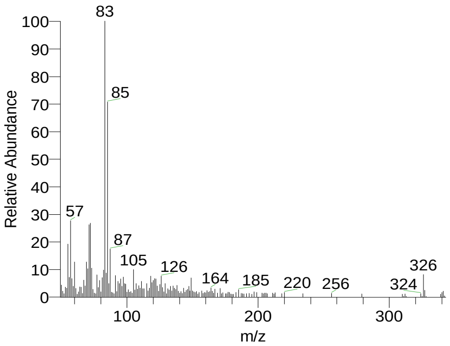

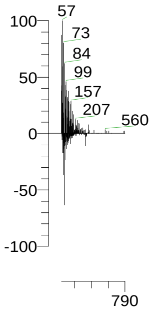

3-Oxo-20-methyl-11-à-hydroxyconanine-1,4-diene
Formula C22H31NO2, MW 341, CAS# NA, Entry# 456618

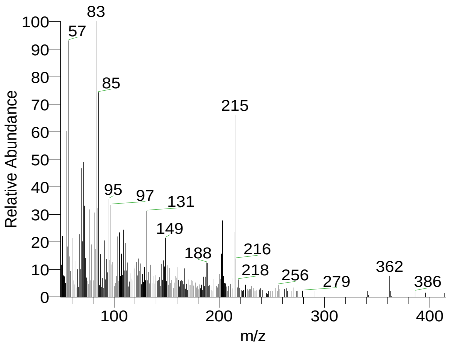

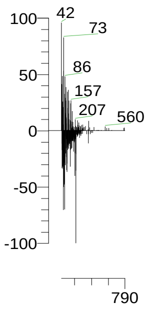

2-Acetyl-3-(2-cinnamido)ethyl-7-methoxyindole
Formula C22H22N2O3, MW 362, CAS# NA, Entry# 491579

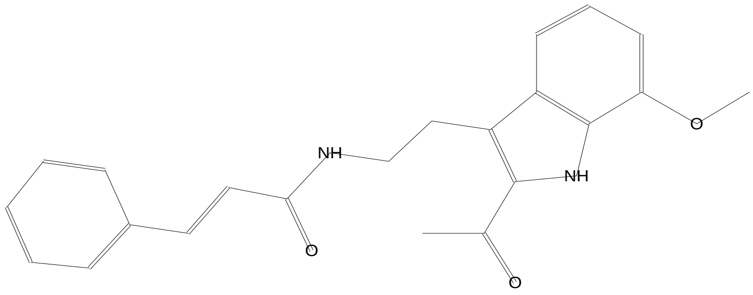

There is no signature data to report.

Library Search Report

Hit Spectrum	Delta	Compound Structure

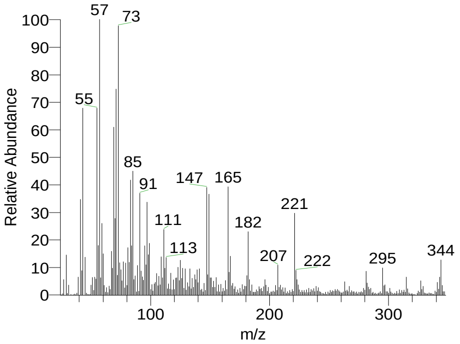

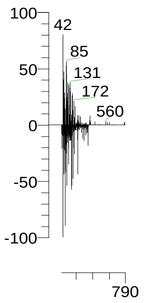

QUERCETIN 7,3',4'-TRIMETHOXY
Formula C18H16O7, MW 344, CAS# 6068-80-0, Entry# 461110
4H-1-Benzopyran-4-one, 2-(3,4-dimethoxyphenyl)-3,5-dihydroxy-7-methoxy- (CAS)

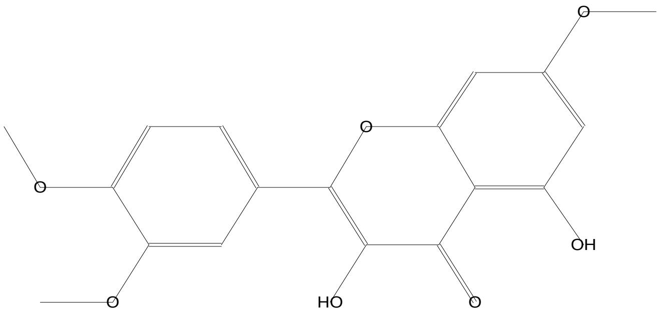

There is no signature data to report.

Library Search Report

RT	Probability	Compound Name	SI	Area %	Area	Molecular Weight	Molecular Formula	Library
28.75	29.07	9,12,15-Octadecatrienoic acid, 2-[(trimethylsilyl)oxy]-1-[[(trimethylsilyl)oxy]methyl]ethyl ester, (Z,Z,Z)-	456	0.15	486563.25	496	C27H52O4Si2	mainlib
28.75	29.07	9,12,15-Octadecatrienoic acid, 2-[(trimethylsilyl)oxy]-1-[[(trimethylsilyl)oxy]methyl]ethyl ester, (Z,Z,Z)- (CAS)	456	0.15	486563.25	496	C27H52O4Si2	Wiley9
28.75	6.85	2-Butanone, (2,4-dinitrophenyl)hydrazone (CAS)	417	0.15	486563.25	252	C10H12N4O4	Wiley9

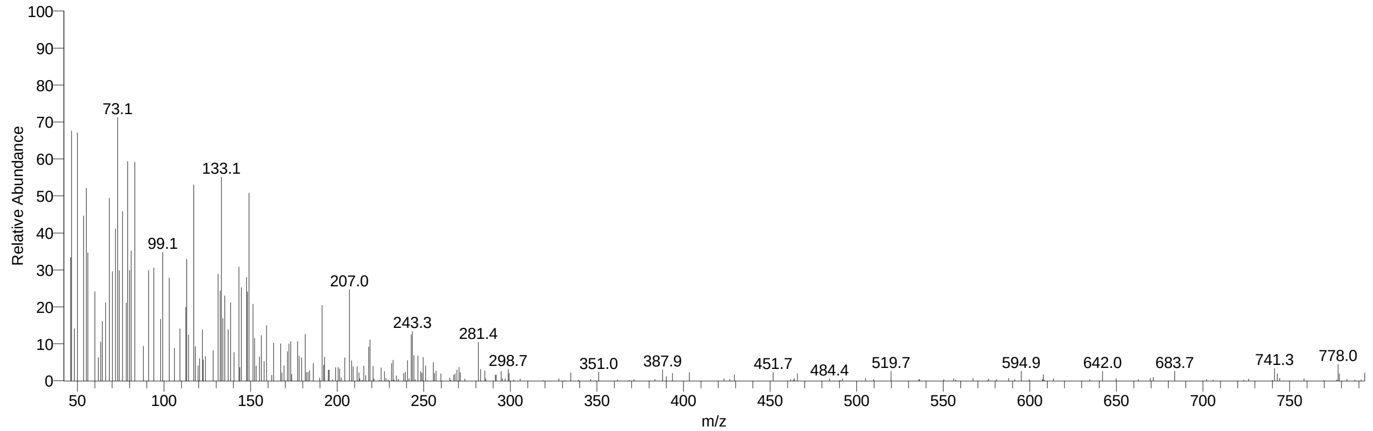

Hit Spectrum	Delta	Compound Structure

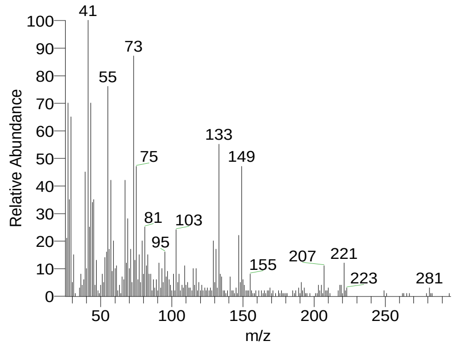

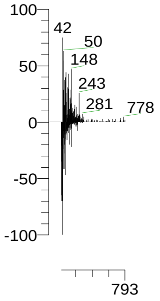

9,12,15-Octadecatrienoic acid, 2-[(trimethylsilyl)oxy]-1-[[(trimethylsilyl)oxy]methyl]ethyl ester, (Z,Z,Z)-
Formula C27H52O4Si2, MW 496, CAS# 55521-23-8, Entry# 3361
2-[(Trimethylsilyl)oxy]-1-([(trimethylsilyl)oxy]methyl)ethyl (9E,12E,15E)-9,12,15-octadecatrienoate #

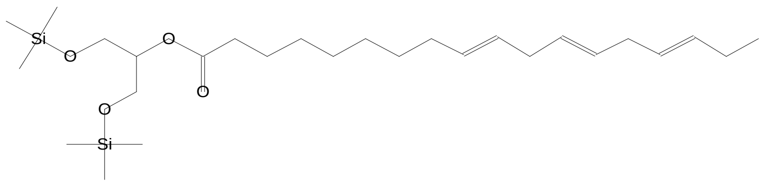

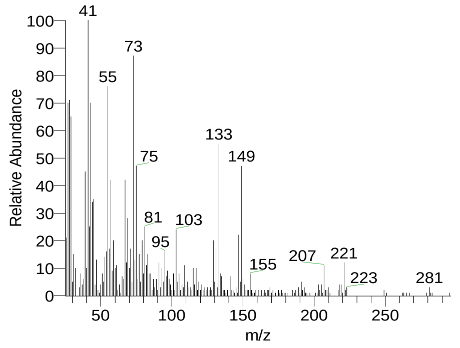

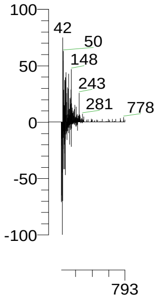

Formula C27H52O4Si2, MW 496, CAS# 55521-23-8, Entry# 616116
TRIMETHYLSILYLETHER DERIVATIVE OF 2-MONOLINOLENIN

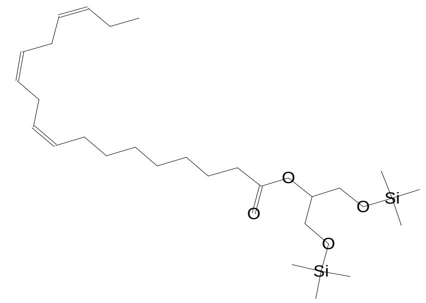

There is no signature data to report.

Library Search Report

Hit Spectrum	Delta	Compound Structure

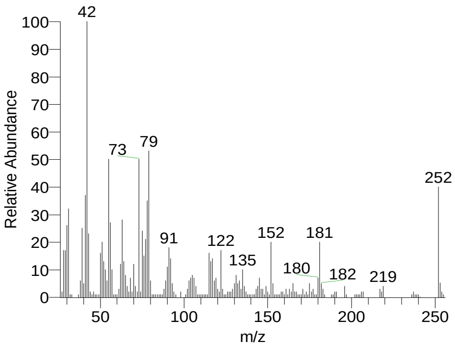

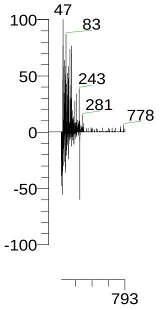

2-Butanone, (2,4-dinitrophenyl)hydrazone (CAS)
Formula C10H12N4O4, MW 252, CAS# 958-60-1, Entry# 262880
METHYLETHYLKETONE-2,4-DNP

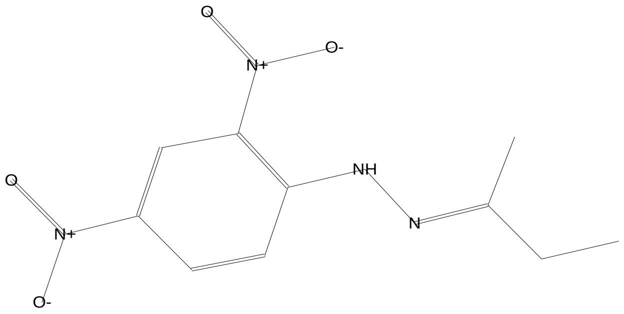

There is no signature data to report.

Library Search Report

RT	Probability	Compound Name	SI	Area %	Area	Molecular Weight	Molecular Formula	Library
28.88	13.30	Docosane (CAS)	451	0.18	585438.82	310	C22H46	Wiley9
28.88	8.06	Nonacosane (CAS)	436	0.18	585438.82	408	C29H60	Wiley9
28.88	5.36	Octadecanoic acid, 9,10-dichloro-, methyl ester	424	0.18	585438.82	366	C19H36Cl2O2	mainlib

Faten-212 #7023 RT: 28.88 AV: 1 RF: 6.00, 3 NL: 6.24E4
F: {0,0} + c EI Full ms [40.00-800.00]

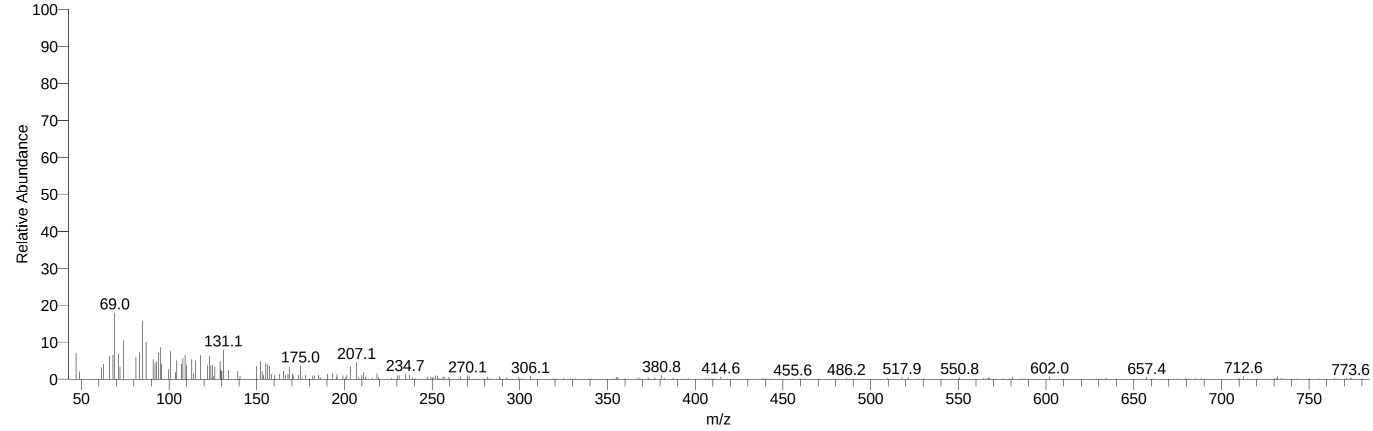

Hit Spectrum	Delta	Compound Structure

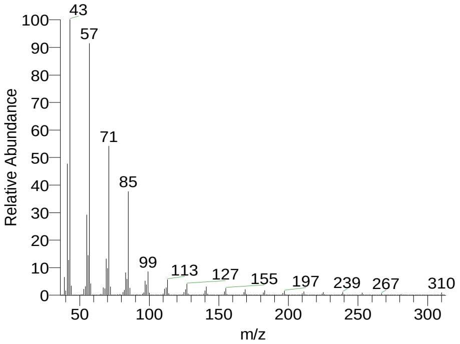

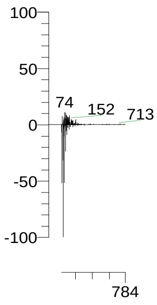

Docosane (CAS)
Formula C22H46, MW 310, CAS# 629-97-0, Entry# 396959
n-Docosane

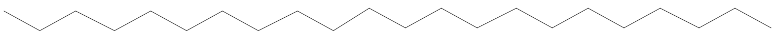

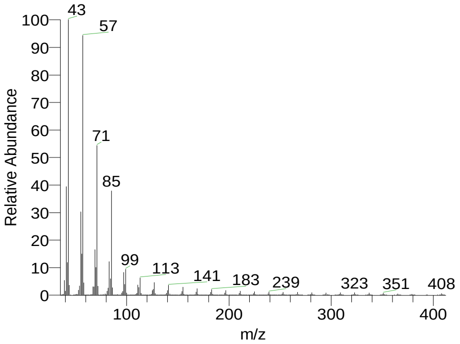

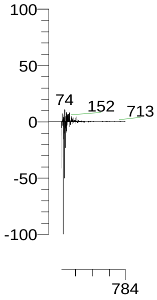

Nonacosane (CAS)
Formula C29H60, MW 408, CAS# 630-03-5, Entry# 552005
n-Nonacosane

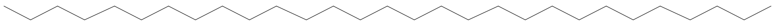

There is no signature data to report.

Library Search Report

Hit Spectrum | Delta | Compound Structure

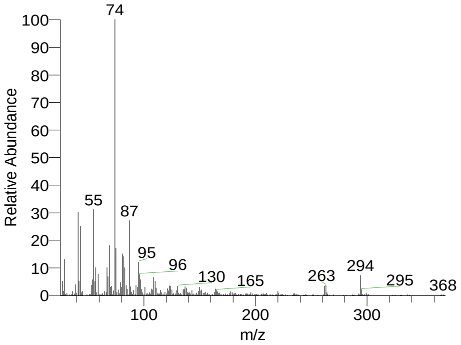

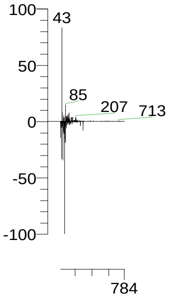

Octadecanoic acid, 9,10-dichloro-, methyl ester
Formula C19H36Cl2O2, MW 366, CAS# 33094-27-8, Entry# 40571
Methyl 9,10-dichlorooctadecanoate

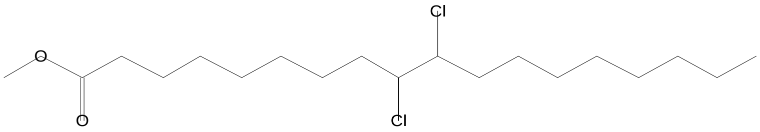

There is no signature data to report.

Library Search Report

RT	Probability	Compound Name	SI	Area %	Area	Molecular Weight	Molecular Formula	Library
28.98	30.49	Silanamine, N-[2-[3-methoxy-4-[(trimethylsilyl)oxy]phenyl]-N,1,1,1-tetramethyl-2-[(trimethylsilyl)oxy]ethyl]- (CAS)	422	0.16	513527.98	413	C19H39NO3Si3	Wiley9
28.98	10.21	cis-5,8,11-Eicosatrienoic acid, trimethylsilyl ester	398	0.16	513527.98	378	C23H42O2Si	mainlib
28.98	5.88	cis-4,7,10,13,16,19-Docosahexaenoic acid, trimethylsilyl ester	382	0.16	513527.98	400	C25H40O2Si	mainlib

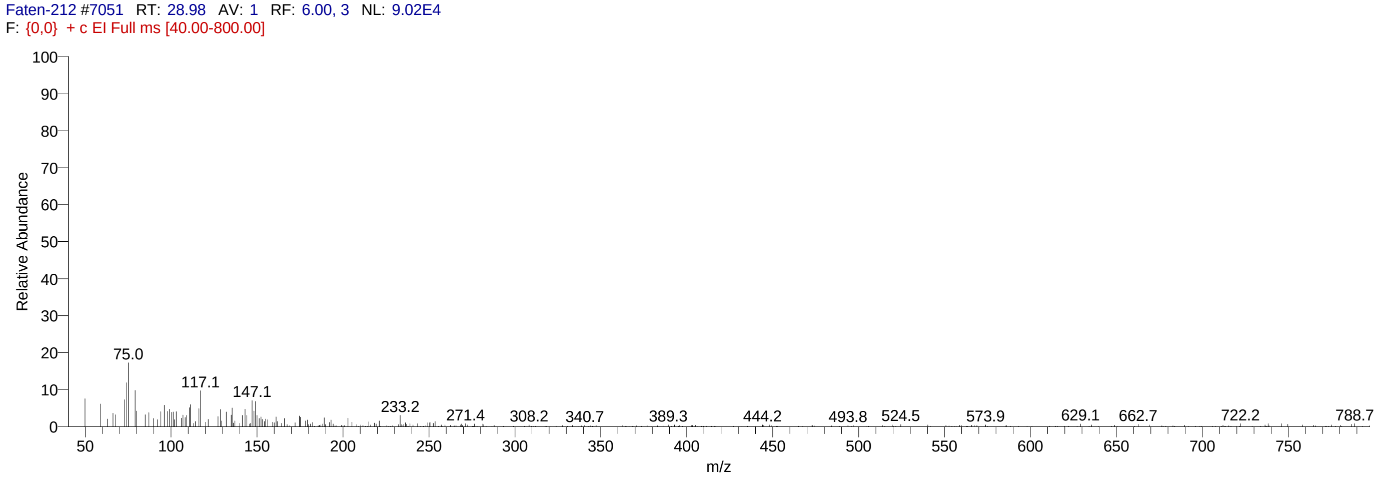

Hit Spectrum	Delta	Compound Structure

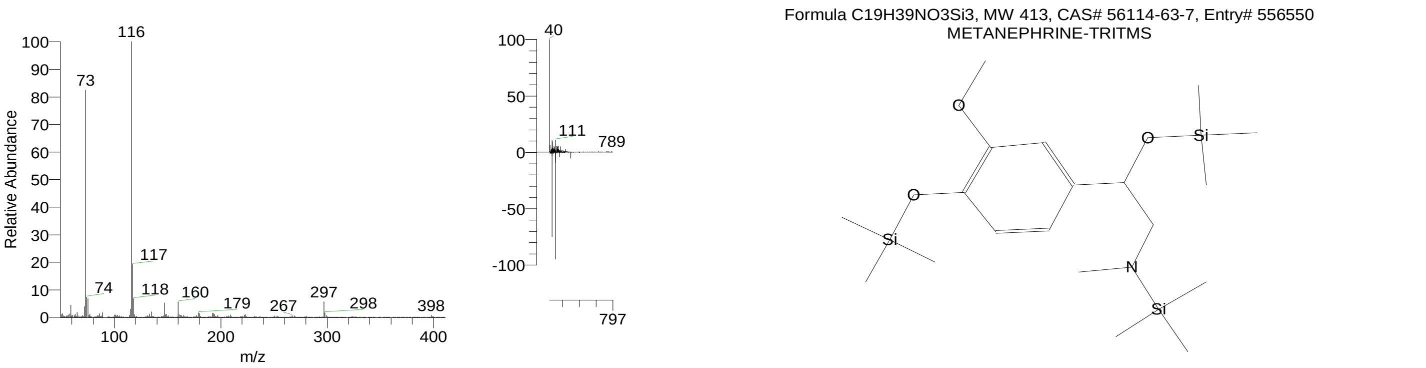

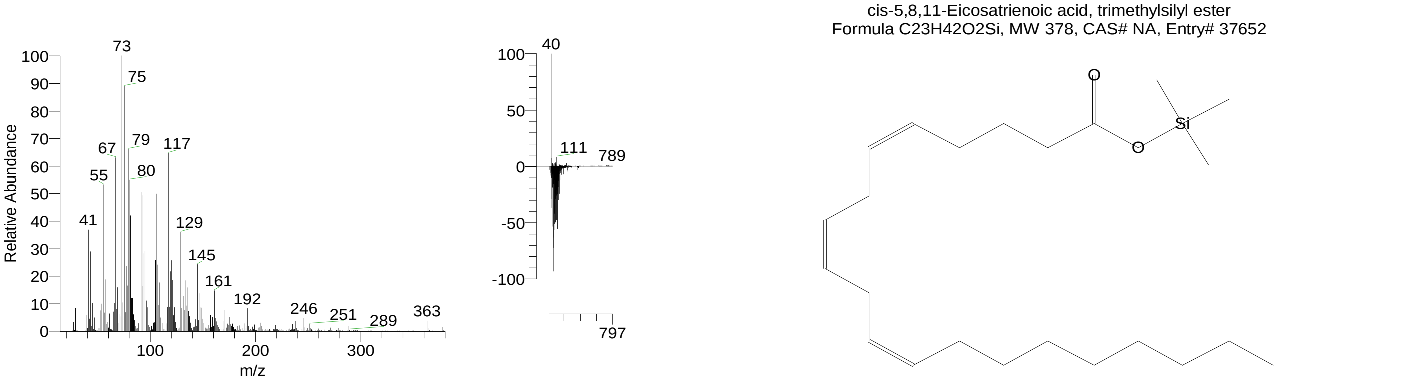

There is no signature data to report.

Library Search Report

Hit Spectrum	Delta	Compound Structure

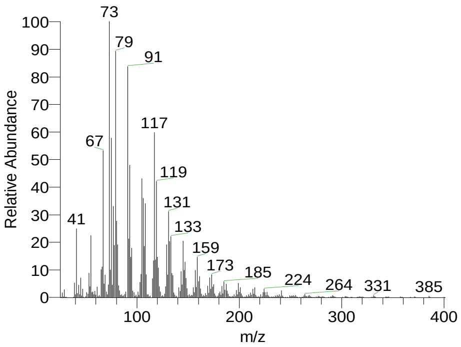

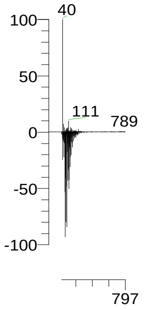

cis-4,7,10,13,16,19-Docosahexaenoic acid, trimethylsilyl ester
Formula C25H40O2Si, MW 400, CAS# NA, Entry# 37920

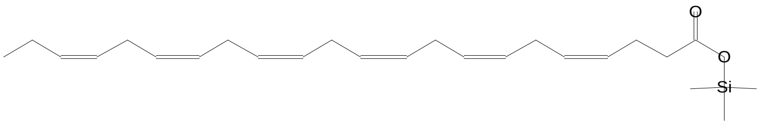

There is no signature data to report.

Library Search Report

RT	Probability	Compound Name	SI	Area %	Area	Molecular Weight	Molecular Formula	Library
29.08	11.43	Dihydroxanthin	428	0.20	638965.57	308	C17H24O5	mainlib
29.08	11.43	Dihydroxanthin	428	0.20	638965.57	308	C17H24O5	Wiley9
29.08	4.17	2-(5-Acetyl-3-cyano-6-methyl-pyridin-2-ylsulfanyl)-N-(3-trifluoromethyl-phenyl)-acetamide	405	0.20	638965.57	393	C18H14F3N3O2S	mainlib

Faten-212 #7081 RT: 29.08 AV: 1 RF: 6.00, 3 NL: 6.76E4
F: {0,0} + c EI Full ms [40.00-800.00]

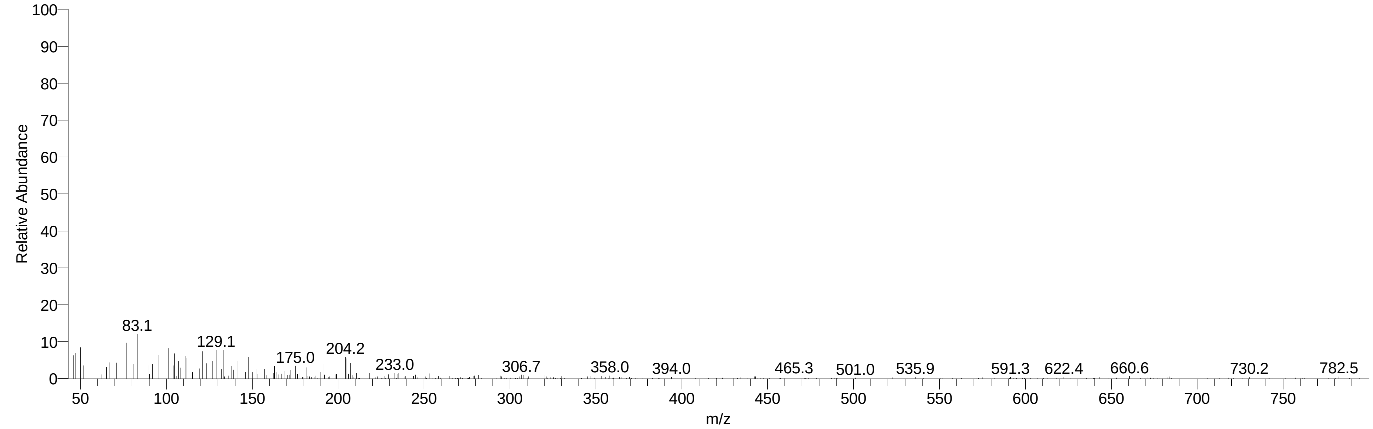

Hit Spectrum	Delta	Compound Structure

SI 428, RSI 481, mainlib, Entry# 6656, CAS# NA, Dihydroxanthin

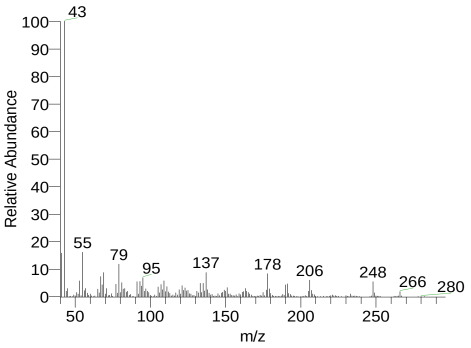

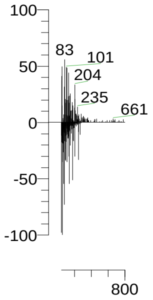

Dihydroxanthin
Formula C17H24O5, MW 308, CAS# NA, Entry# 6656
1-(3,7-Dimethyl-2-oxo-3,3a,4,5,8,8a-hexahydro-2H-cyclohepta[b]furan-6-yl)-3-oxobutyl acetate #

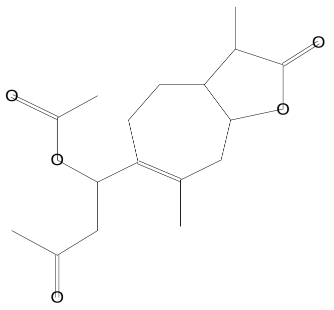

Dihydroxanthin
Formula C17H24O5, MW 308, CAS# NA, Entry# 391300
1-(3,7-Dimethyl-2-oxo-3,3a,4,5,8,8a-hexahydro-2H-cyclohepta[b]furan-6-yl)-3-oxobutyl acetate

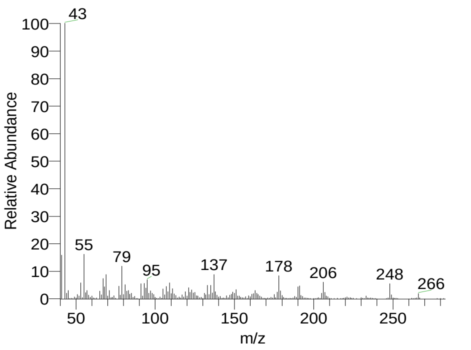

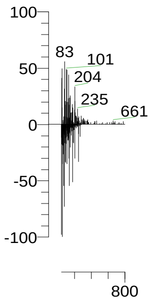

There is no signature data to report.

Library Search Report

Hit Spectrum	Delta	Compound Structure

2-(5-Acetyl-3-cyano-6-methyl-pyridin-2-ylsulfanyl)-N-(3-trifluoromethyl-phenyl)-acetamide
Formula C18H14F3N3O2S, MW 393, CAS# NA, Entry# 6569

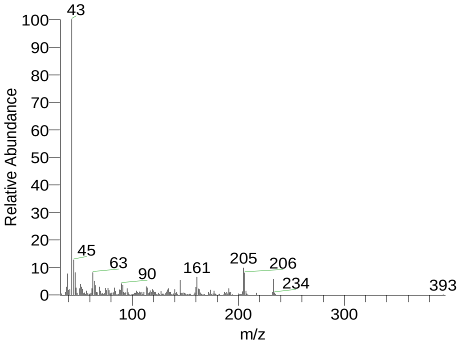

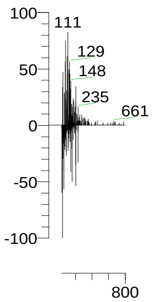

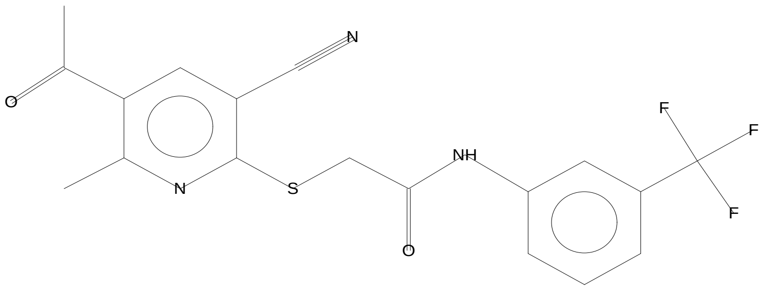

There is no signature data to report.

Library Search Report

RT	Probability	Compound Name	SI	Area %	Area	Molecular Weight	Molecular Formula	Library
29.35	8.72	[5,9-Dimethyl-1-(3-phenyl-oxiran-2-yl)-deca-4,8-dienylidene]-(2-phenyl-aziridin-1-yl)-amine	436	0.15	478532.45	414	C28H34N2O	mainlib
29.35	8.72	[5,9-Dimethyl-1-(3-phenyl-oxiran-2-yl)-deca-4,8-dienylidene]-(2-phenyl-aziridin-1-yl)-amine	436	0.15	478532.45	414	C28H34N2O	Wiley9
29.35	6.85	Aspidospermidine (CAS)	430	0.15	478532.45	282	C19H26N2	Wiley9

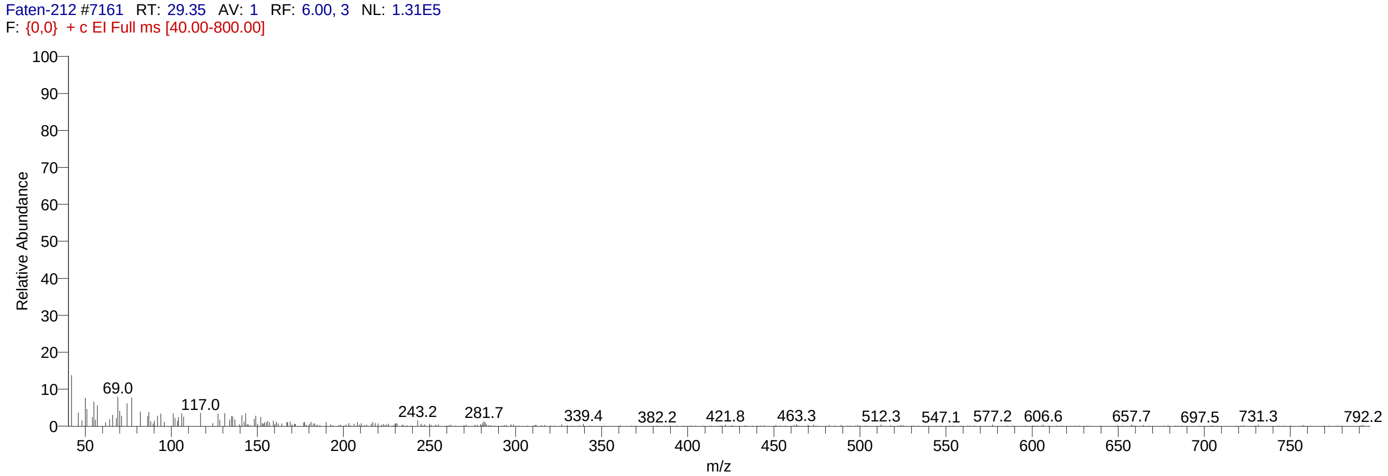

Hit Spectrum	Delta	Compound Structure

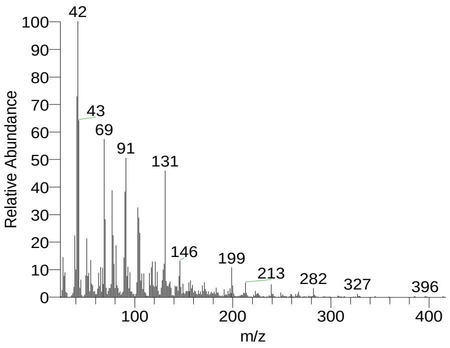

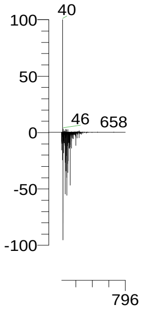

[5,9-Dimethyl-1-(3-phenyl-oxiran-2-yl)-deca-4,8-dienylidene]-(2-phenyl-aziridin-1-yl)-amine
Formula C28H34N2O, MW 414, CAS# NA, Entry# 4208
N-[(4E)-5,9-Dimethyl-1-(3-phenyl-2-oxiranyl)-4,8-decadienylidene]-2-phenyl-1-aziridinamine #

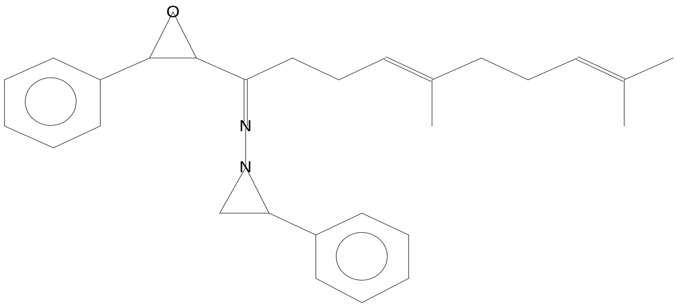

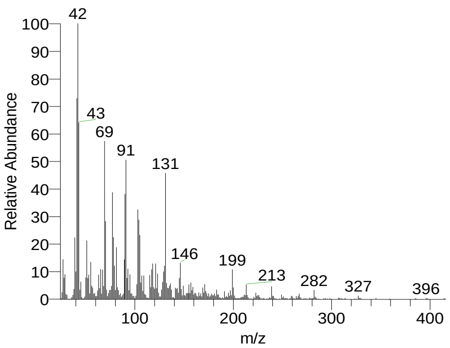

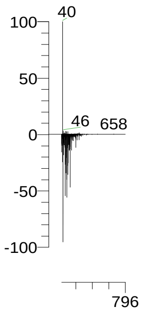

[5,9-Dimethyl-1-(3-phenyl-oxiran-2-yl)-deca-4,8-dienylidene]-(2-phenyl-aziridin-1-yl)-amine
Formula C28H34N2O, MW 414, CAS# NA, Entry# 558433
[5,9-DIMETHYL-1-(3-PHENYL-OXIRAN-2-YL)-DECA-4,8-DIENYLIDENE]-(2-PHENYL-AZIRIDIN-

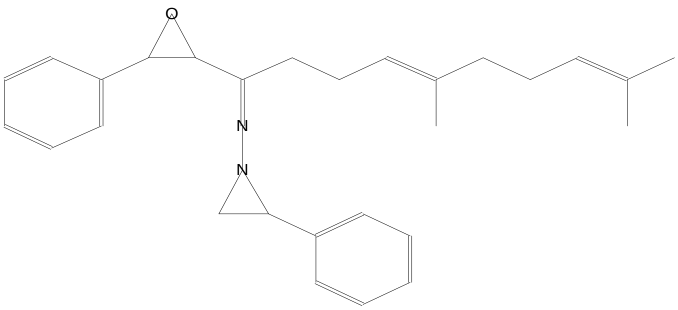

There is no signature data to report.

Library Search Report

Hit Spectrum	Delta	Compound Structure

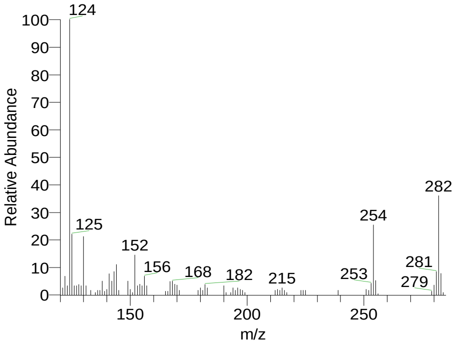

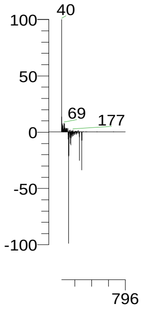

Aspidospermidine (CAS)
Formula C19H26N2, MW 282, CAS# 2912-09-6, Entry# 335384
(+)-Aspidospermidine

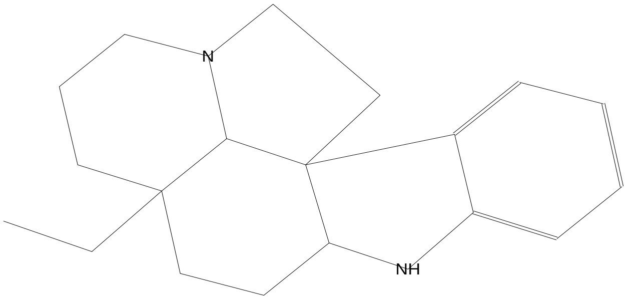

There is no signature data to report.

Library Search Report

RT	Probability	Compound Name	SI	Area %	Area	Molecular Weight	Molecular Formula	Library
29.76	5.30	Bicyclo[4.4.0]dec-2-ene-4-ol, 2-methyl-9-(prop-1-en-3-ol-2-yl)-	415	0.42	1336240.15	236	C15H24O2	mainlib
29.76	5.30	Bicyclo[4.4.0]dec-2-ene-4-ol, 2-methyl-9-(prop-1-en-3-ol-2-yl)-	415	0.42	1336240.15	236	C15H24O2	Wiley9
29.76	4.89	9,12,15-Octadecatrienoic acid, 2-[(trimethylsilyl)oxy]-1-[[(trimethylsilyl)oxy]methyl]ethyl ester, (Z,Z,Z)-	413	0.42	1336240.15	496	C27H52O4Si2	mainlib

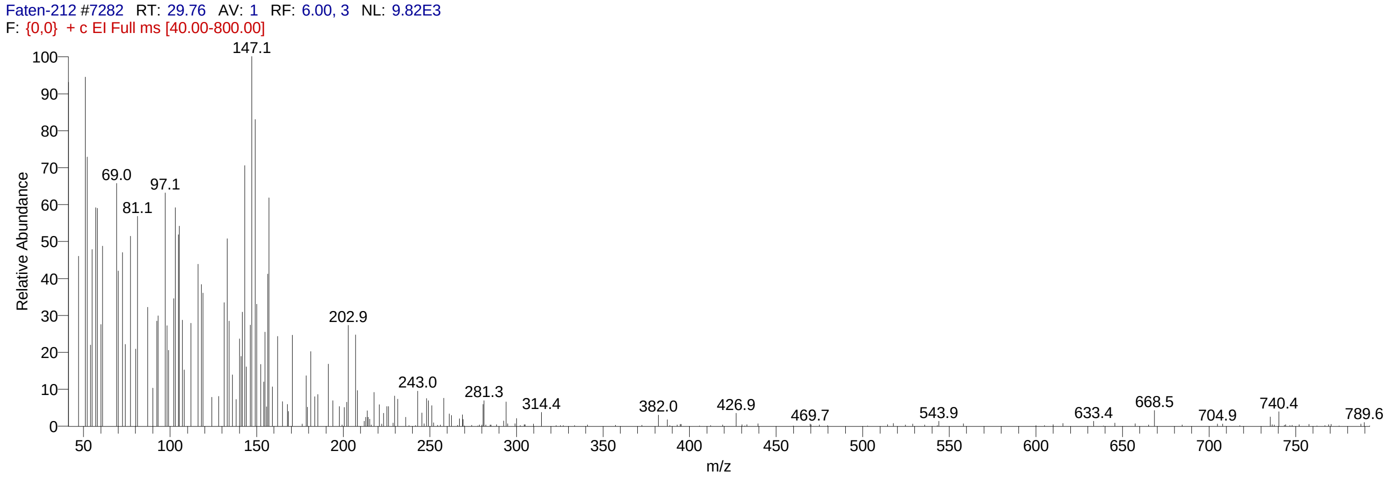

Hit Spectrum	Delta	Compound Structure

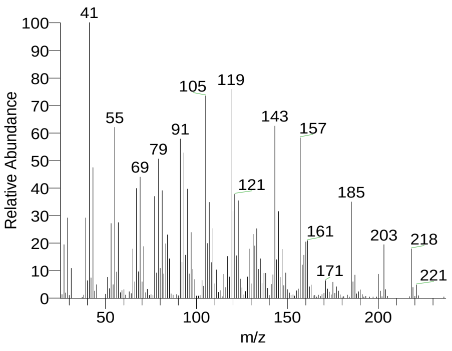

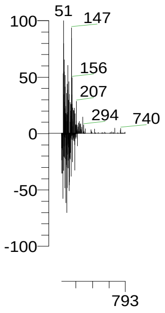

Bicyclo[4.4.0]dec-2-ene-4-ol, 2-methyl-9-(prop-1-en-3-ol-2-yl)-
Formula C15H24O2, MW 236, CAS# NA, Entry# 3766
6-[1-(Hydroxymethyl)vinyl]-4,8a-dimethyl-1,2,4a,5,6,7,8,8a-octahydro-2-naphthalenol #

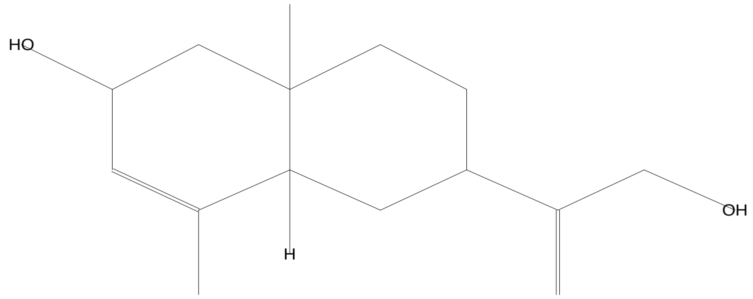

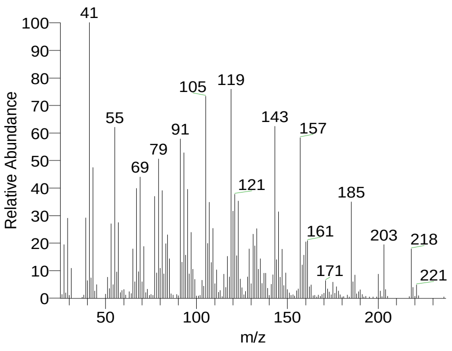

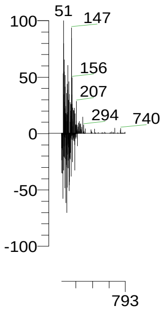

Bicyclo[4.4.0]dec-2-ene-4-ol, 2-methyl-9-(prop-1-en-3-ol-2-yl)-
Formula C15H24O2, MW 236, CAS# NA, Entry# 226889
6-[1-(Hydroxymethyl)vinyl]-4,8a-dimethyl-1,2,4a,5,6,7,8,8a-octahydro-2-naphthalenol

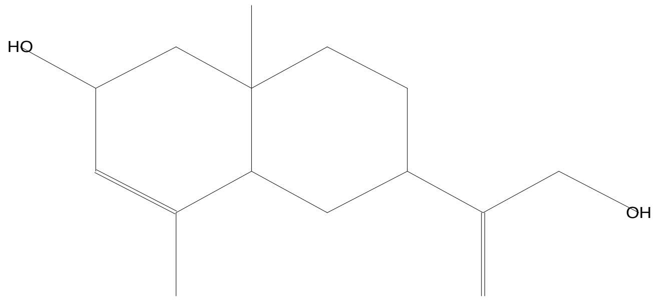

There is no signature data to report.

Library Search Report

Hit Spectrum

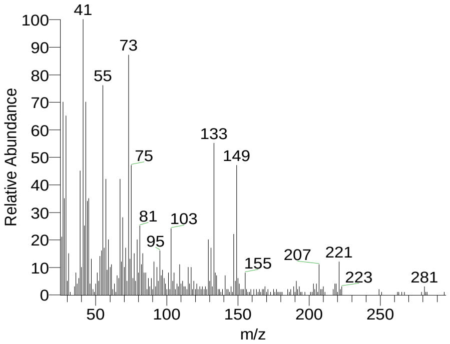

Delta

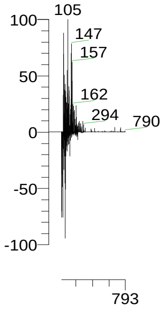

Compound Structure

9,12,15-Octadecatrienoic acid, 2-[(trimethylsilyl)oxy]-1-[[(trimethylsilyl)oxy]methyl]ethyl ester, (Z,Z,Z)-
Formula C27H52O4Si2, MW 496, CAS# 55521-23-8, Entry# 3361
2-[(Trimethylsilyl)oxy]-1-([(trimethylsilyl)oxy]methyl)ethyl (9E,12E,15E)-9,12,15-octadecatrienoate #

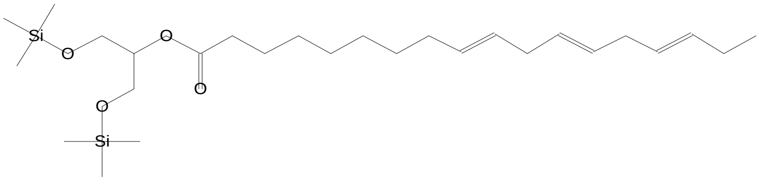

There is no signature data to report.

Library Search Report

RT	Probability	Compound Name	SI	Area %	Area	Molecular Weight	Molecular Formula	Library
30.24	57.91	2,4-diphenyl-glutaronitrile	663	1.33	4214125.25	246	C17H14N2	Wiley9
30.24	9.51	3,6,10,13-Tetraoxa-2,14-disilapentadecane, 2,2,14,14-tetramethyl-	599	1.33	4214125.25	308	C13H32O4Si2	mainlib
30.24	9.51	2,2,14,14-TETRAMETHYL-3,6,10,13-TETRAOXA-2,14-DISILAPENTADECANE	599	1.33	4214125.25	308	C13H32O4Si2	Wiley9

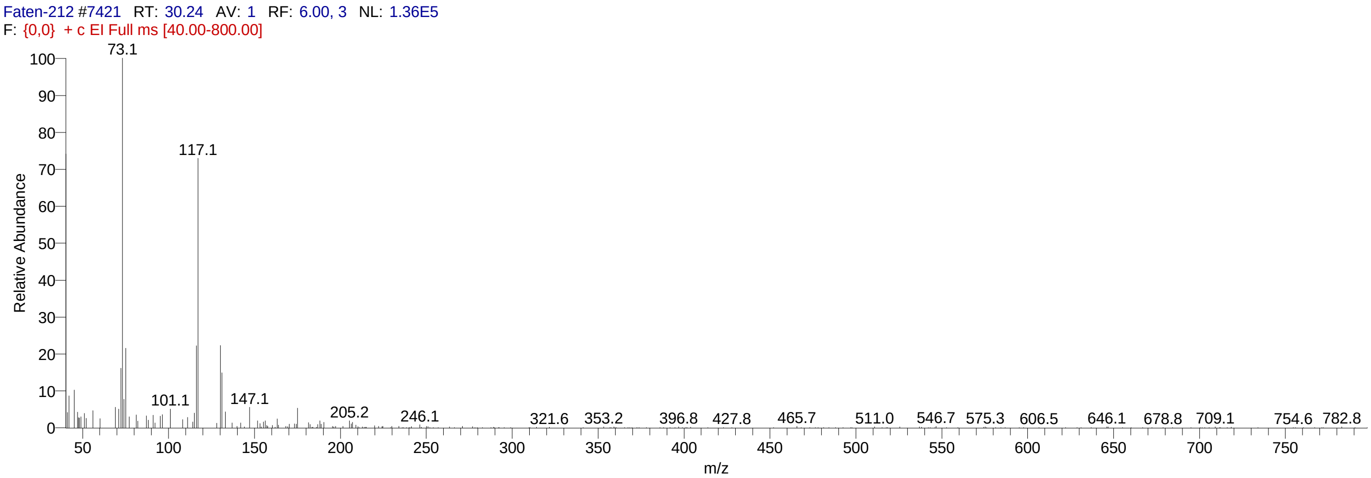

Hit Spectrum | Delta | Compound Structure

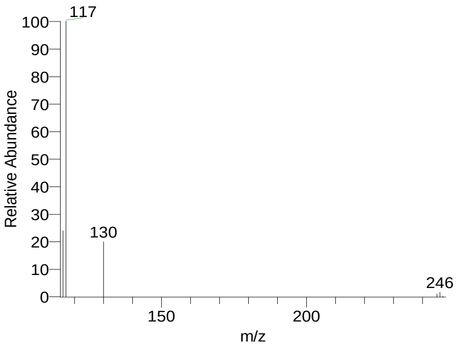

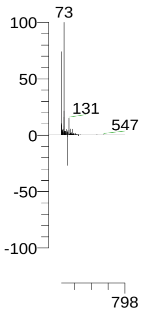

2,4-diphenyl-glutaronitrile
Formula C17H14N2, MW 246, CAS# 19657-49-9, Entry# 250780

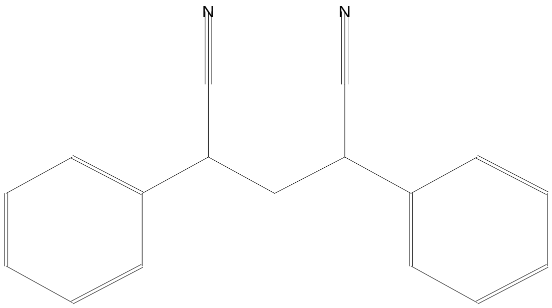

3,6,10,13-Tetraoxa-2,14-disilapentadecane, 2,2,14,14-tetramethyl-
Formula C13H32O4Si2, MW 308, CAS# NA, Entry# 38404
2,2,14,14-Tetramethyl-3,6,10,13-tetraoxa-2,14-disilapentadecane #

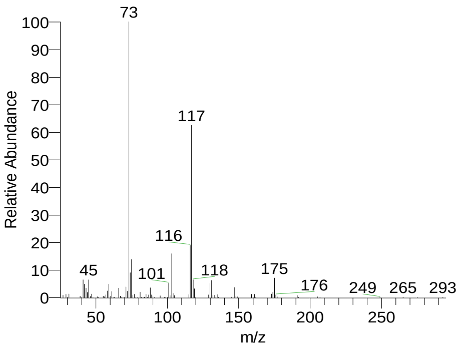

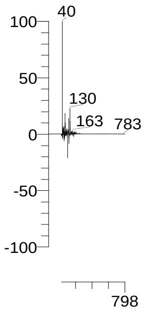

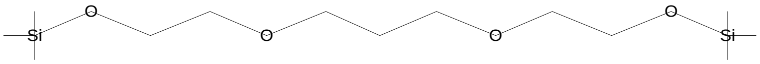

There is no signature data to report.

Library Search Report

Hit Spectrum	Delta	Compound Structure

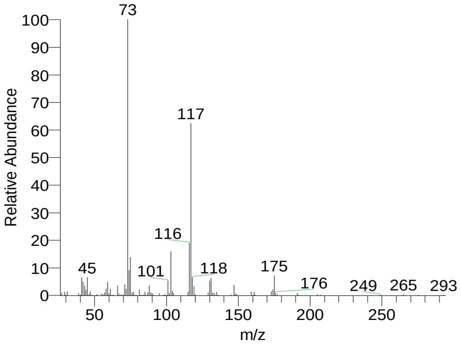

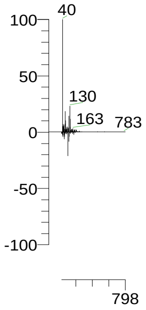

2,2,14,14-TETRAMETHYL-3,6,10,13-TETRAOXA-2,14-DISILAPENTADECANE
Formula C13H32O4Si2, MW 308, CAS# NA, Entry# 390458

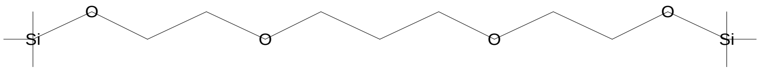

There is no signature data to report.

Library Search Report

RT	Probability	Compound Name	SI	Area %	Area	Molecular Weight	Molecular Formula	Library
30.51	9.27	2,2,18,18-TETRAMETHYL-3,6,10,13,17-PENTAOXA-2,18-DISILANEONADECANE	534	1.19	3792960.04	366	C16H38O5Si2	Wiley9
30.51	7.83	3,7,11,14,18-Pentaoxa-2,19-disilaeicosane, 2,2,19,19-tetramethyl-	530	1.19	3792960.04	380	C17H40O5Si2	mainlib
30.51	7.83	2,2,19,19-TETRAMETHYL-3,7,11,14,18-PENTAOXA-2,19-DISILAEICOSANE	530	1.19	3792960.04	380	C17H40O5Si2	Wiley9

Faten-212 #7500 RT: 30.51 AV: 1 RF: 6.00, 3 NL: 1.18E5
F: {0,0} + c EI Full ms [40.00-800.00]

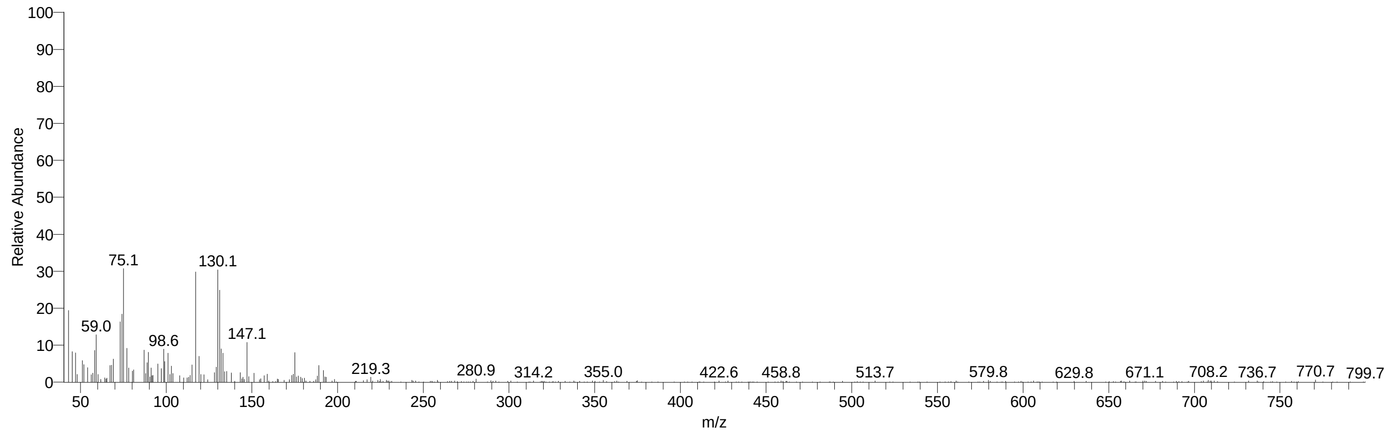

Hit Spectrum | Delta | Compound Structure

2,2,18,18-TETRAMETHYL-3,6,10,13,17-PENTAOXA-2,18-DISILANEONADECANE
Formula C16H38O5Si2, MW 366, CAS# NA, Entry# 496578

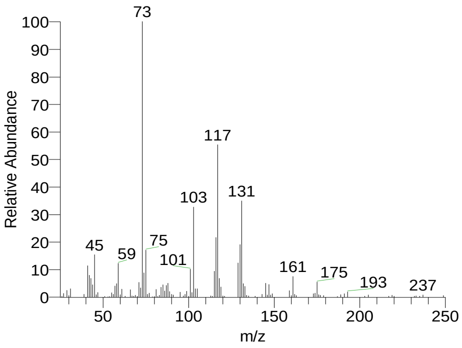

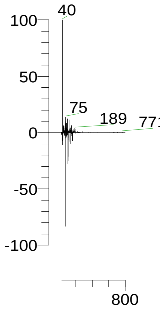

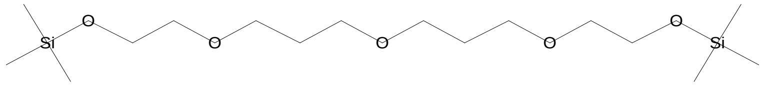

3,7,11,14,18-Pentaoxa-2,19-disilaeicosane, 2,2,19,19-tetramethyl-
Formula C17H40O5Si2, MW 380, CAS# NA, Entry# 38558
2,2,19,19-Tetramethyl-3,7,10,14,18-pentaoxa-2,19-disilaicosane #

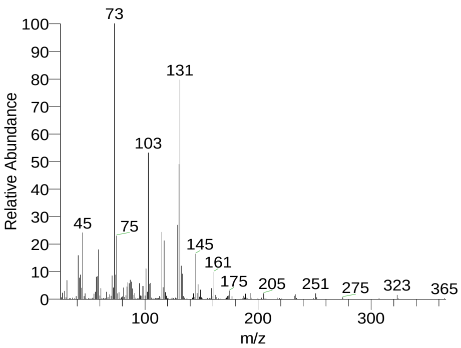

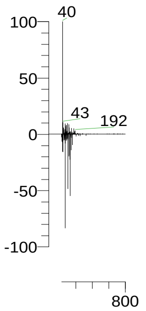

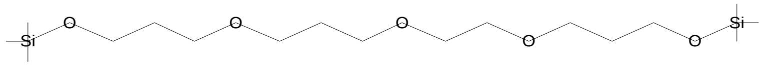

There is no signature data to report.

Library Search Report

Hit Spectrum	Delta	Compound Structure

2,2,19,19-TETRAMETHYL-3,7,11,14,18-PENTAOXA-2,19-DISILAEICOSANE
Formula C17H40O5Si2, MW 380, CAS# NA, Entry# 516845

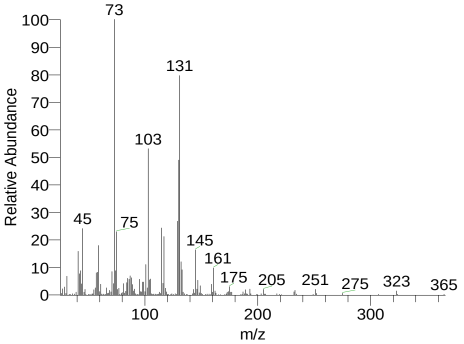

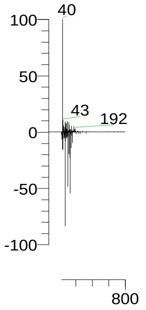

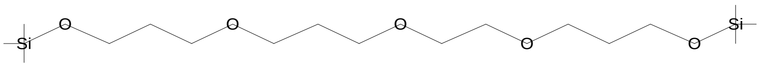

There is no signature data to report.

Library Search Report

RT	Probability	Compound Name	SI	Area %	Area	Molecular Weight	Molecular Formula	Library
31.39	11.25	1,1,1-Tris(hydroxymethyl)propane, tris(trimethylsilyl) ether	579	4.24	13450336.03	350	C15H38O3Si3	mainlib
31.39	10.38	7,9-Diethyl-2,4-bis(dimethylamino)-10-imino-8-thio-1,7,9-triazaspiro[4.5]-1,3-decadiene-6,8-dione	577	4.24	13450336.03	336	C15H24N6OS	mainlib
31.39	8.36	erythro-Pentitol, 2-deoxy-1,3,4,5-tetrakis-O-(trimethylsilyl)-(CAS)	572	4.24	13450336.03	424	C17H44O4Si4	Wiley9

Faten-212 #7760 RT: 31.39 AV: 1 RF: 6.00, 3 NL: 1.09E5
F: {0,0} + c EI Full ms [40.00-800.00]

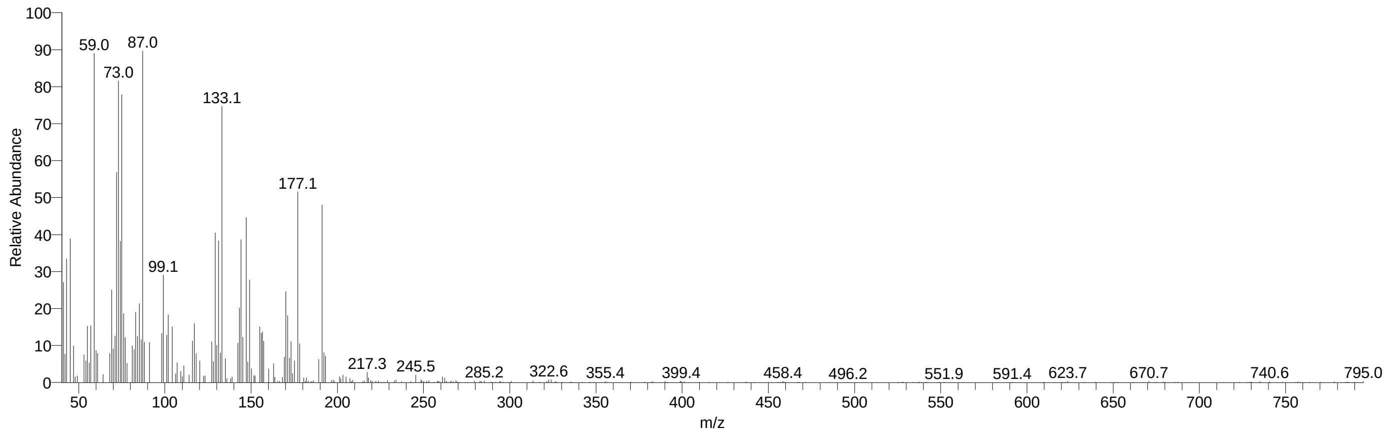

Hit Spectrum	Delta	Compound Structure

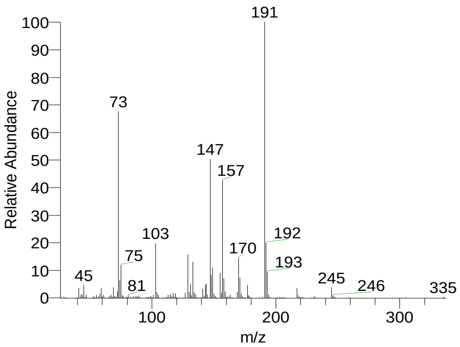

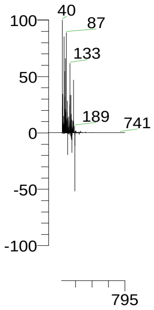

1,1,1-Tris(hydroxymethyl)propane, tris(trimethylsilyl) ether
Formula C15H38O3Si3, MW 350, CAS# NA, Entry# 156424

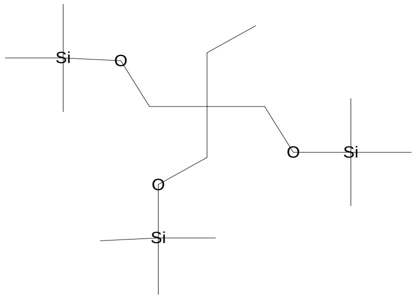

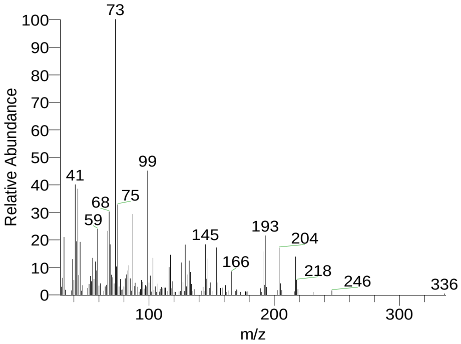

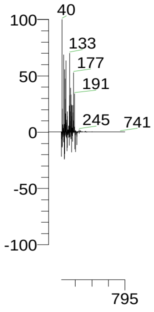

7,9-Diethyl-2,4-bis(dimethylamino)-10-imino-8-thio-1,7,9-triazaspiro[4.5]-1,3-decadiene-6,8-dione
Formula C15H24N6OS, MW 336, CAS# NA, Entry# 38103
2,4-Bis(dimethylamino)-7,9-diethyl-10-imino-8-thioxo-1,7,9-triazaspiro[4.5]deca-1,3-dien-6-one #

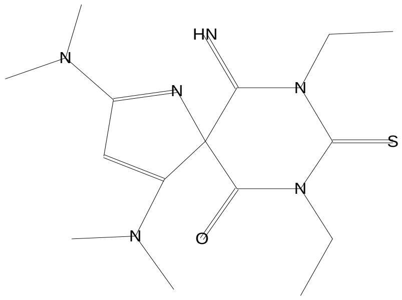

There is no signature data to report.

Library Search Report

Hit Spectrum	Delta	Compound Structure

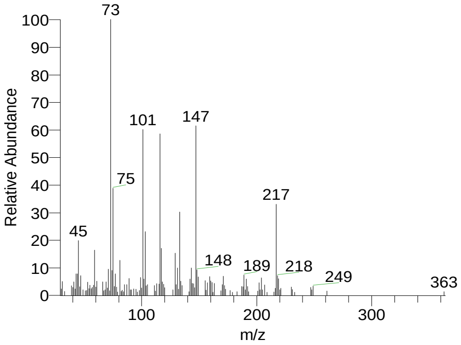

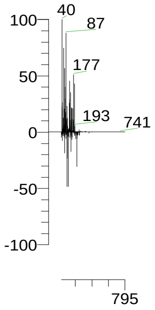

erythro-Pentitol, 2-deoxy-1,3,4,5-tetrakis-O-(trimethylsilyl)- (CAS)
Formula C17H44O4Si4, MW 424, CAS# 56271-71-7, Entry# 567197
2-DEOXYRIBITOL-1,3,4,5-TETRATMS

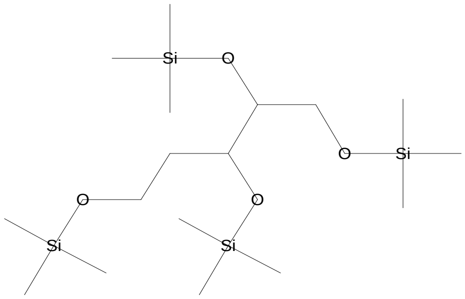

There is no signature data to report.

Library Search Report

RT	Probability	Compound Name	SI	Area %	Area	Molecular Weight	Molecular Formula	Library
31.78	95.97	1,4,7-Tris(3,5-di-tert-butyl-2-deuteriohydroxybenzyl)-1,4,7-triazacyclononane	690	0.15	473178.36	783	C51H72D9N3O3	Wiley9
31.78	0.95	GWTLNSAGYLLGPHAVGNHRSFSDKNGLTS/4	447	0.15	473178.36	3159	N/A	nist_msms2
31.78	0.43	Prost-13-en-1-oic acid, 9-(methoxyimino)-11,15-bis[(trimethylsilyl)oxy]-, trimethylsilyl ester, (8.xi.,12.xi.)-	427	0.15	473178.36	599	C30H61NO5Si3	mainlib

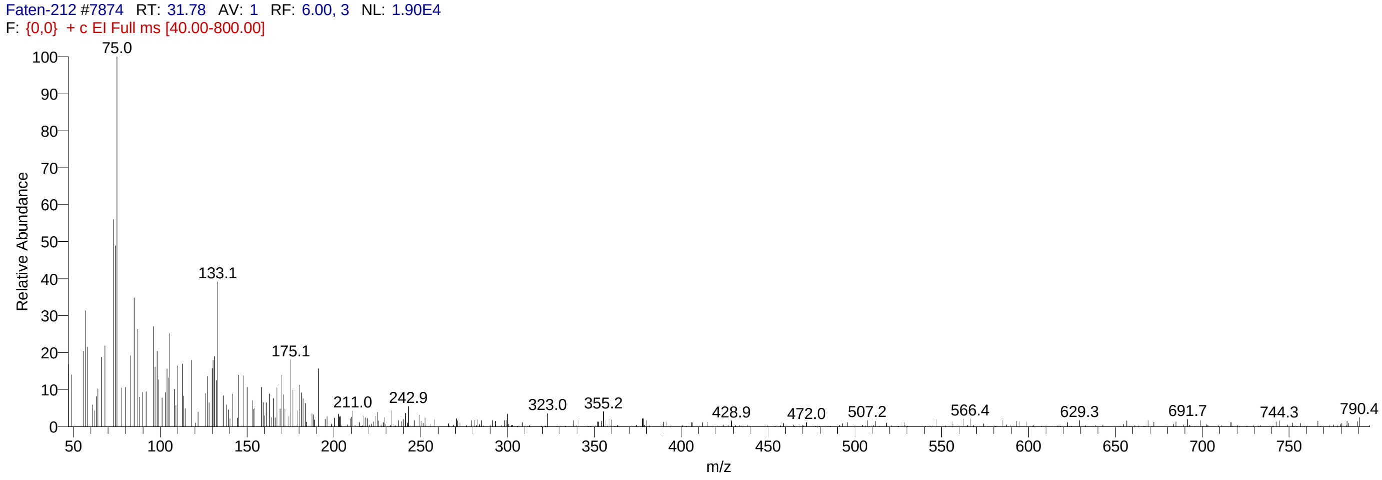

Hit Spectrum	Delta	Compound Structure

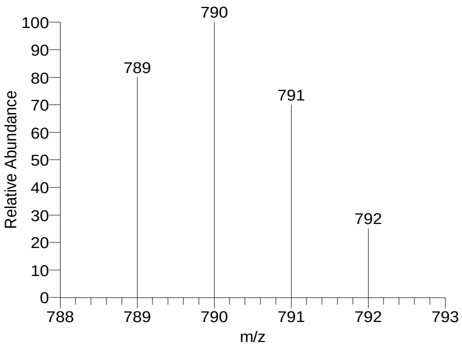

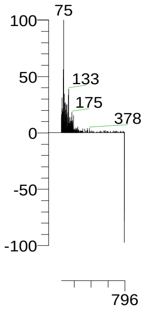

1,4,7-Tris(3,5-di-tert-butyl-2-deuteriohydroxybenzyl)-1,4,7-triazacyclononane
Formula C51H72D9N3O3, MW 783, CAS# NA, Entry# 658265

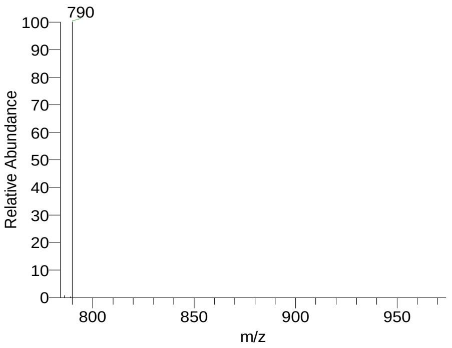

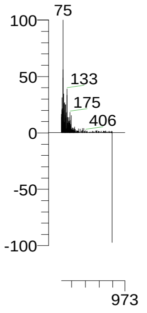

GWTLNSAGYLLGPHAVGNHRSFSDKNGLTS/4
Formula , MW 3159, CAS# NA, Entry# 9262
$:00ms2

There is no signature data to report.

Library Search Report

Hit Spectrum	Delta	Compound Structure

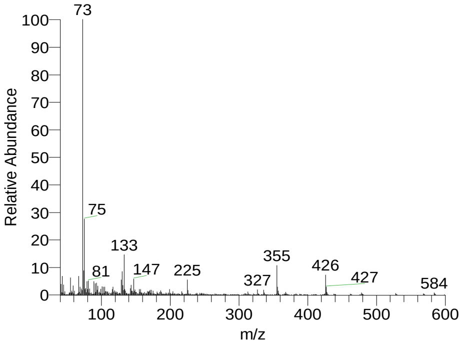

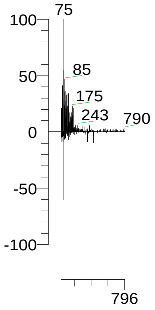

Formula C30H61NO5Si3, MW 599, CAS# 72150-30-2, Entry# 37735
Trimethylsilyl 9-(methoxyimino)-11,15-bis[(trimethylsilyl)oxy]prost-13-en-1-oate

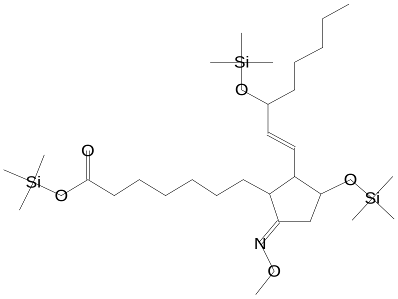

There is no signature data to report.

Library Search Report

RT	Probability	Compound Name	SI	Area %	Area	Molecular Weight	Molecular Formula	Library
31.86	27.34	à-D-Glucopyranoside, methyl 2-(acetylamino)-2-deoxy-3-O-(trimethylsilyl)-, cyclic methylboronate (CAS)	395	0.17	528511.93	331	C13H26BNO6Si	Wiley9
31.86	27.34	à-D-Glucopyranoside, methyl 2-(acetylamino)-2-deoxy-3-O-(trimethylsilyl)-, cyclic methylboronate	393	0.17	528511.93	331	C13H26BNO6Si	mainlib
31.86	18.21	1-(4'-TRIMETHYSILYLOXYPHENYL)-1-TRIMETHYLSILYLOXY-2-METHYLTRIMETHYLSILYL-AMINOETHANE	383	0.17	528511.93	383	C18H37NO2Si3	Wiley9

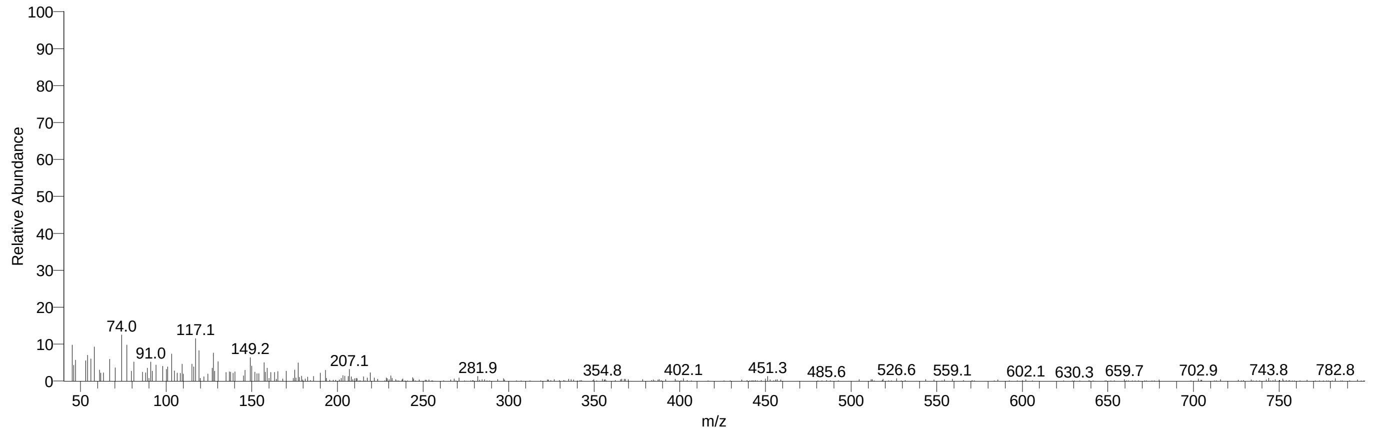

Hit Spectrum | Delta | Compound Structure

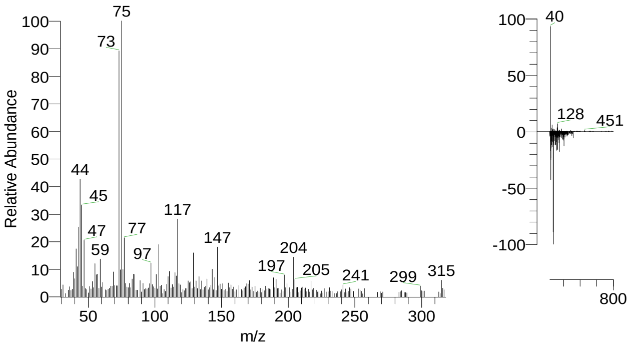

Formula C13H26BNO6Si, MW 331, CAS# 54477-01-9, Entry# 437410
2-ACETAMIDO-A-GLUCOPYRANOSIDE-1-METHYL-4,6-METHYLBORONATE-3-TMS

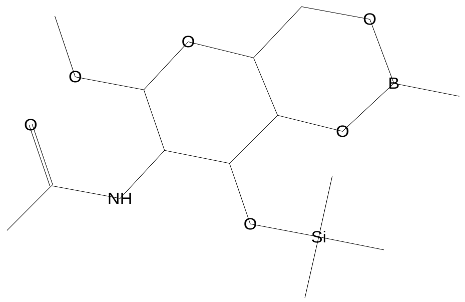

There is no signature data to report.

Library Search Report

Hit Spectrum	Delta	Compound Structure

à-D-Glucopyranoside, methyl 2-(acetylamino)-2-deoxy-3-O-(trimethylsilyl)-, cyclic methylboronate
Formula C13H26BNO6Si, MW 331, CAS# 54477-01-9, Entry# 41200

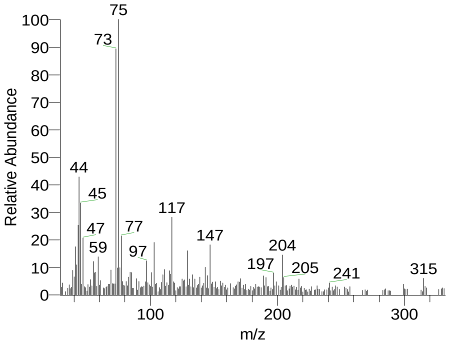

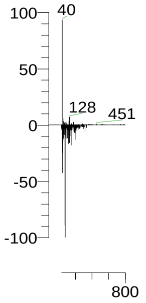

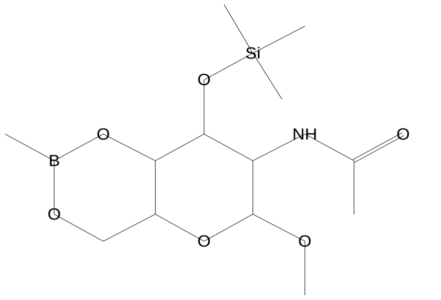

Formula C18H37NO2Si3, MW 383, CAS# NA, Entry# 520945

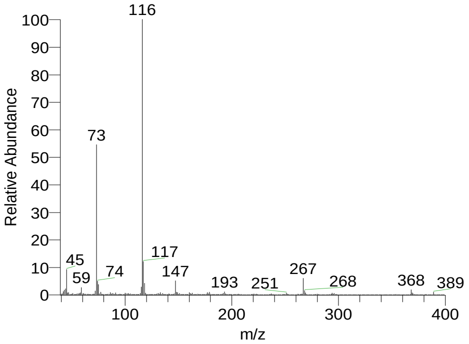

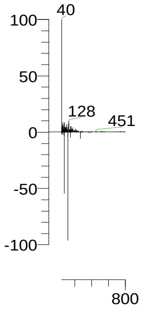

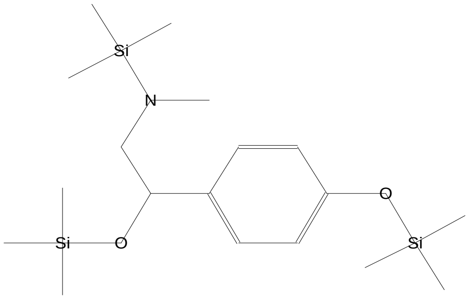

There is no signature data to report.

Library Search Report

RT	Probability	Compound Name	SI	Area %	Area	Molecular Weight	Molecular Formula	Library
32.12	23.15	Lucenin 2	444	0.14	457604.61	610	C27H30O16	Wiley9
32.12	15.88	QUERCETIN 7,3',4'-TRIMETHOXY	433	0.14	457604.61	344	C18H16O7	Wiley9
32.12	12.16	Cyclopropa[5,6]-A-nor-5à-androstane-3,7-dione, 3',6á-dihydro-17á-hydroxy-3',3'-dimethyl-, acetate	426	0.14	457604.61	372	C23H32O4	mainlib

Faten-212 #7975 RT: 32.12 AV: 1 RF: 6.00, 3 NL: 4.67E4
F: {0,0} + c EI Full ms [40.00-800.00]

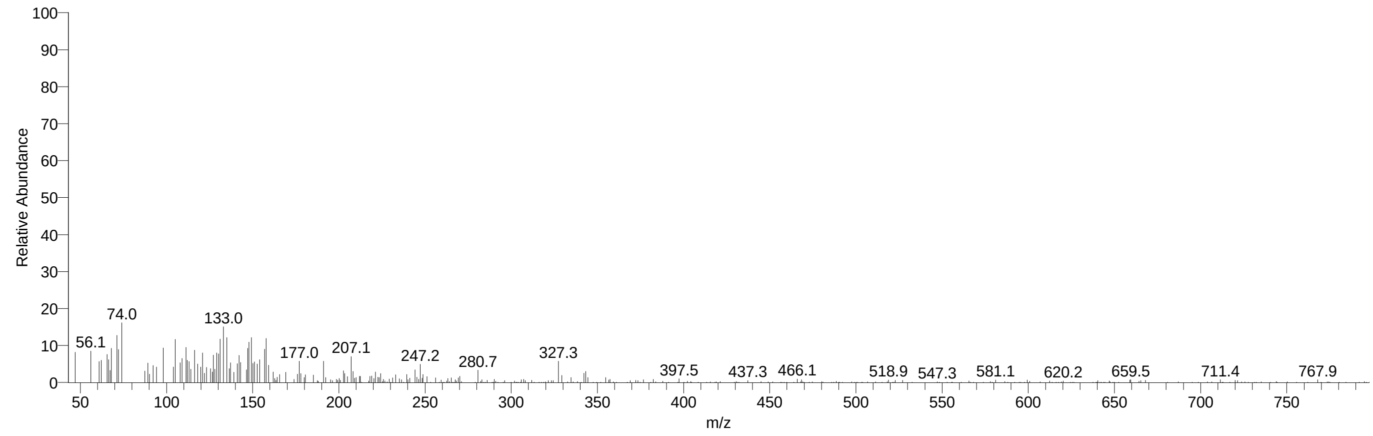

Hit Spectrum	Delta	Compound Structure

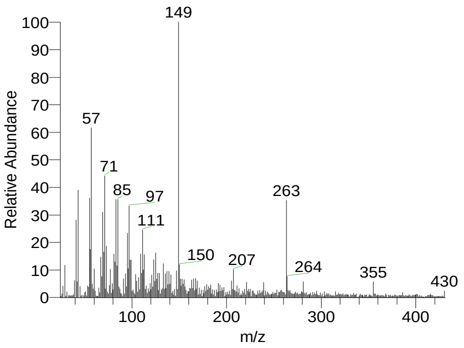

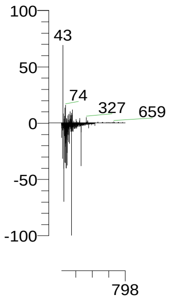

Lucenin 2
Formula C27H30O16, MW 610, CAS# 29428-58-8, Entry# 645795
4H-1-Benzopyran-4-one, 2-(3,4-dihydroxyphenyl)-6,8-di-á-D-glucopyranosyl-5,7-dihydroxy- (CAS)

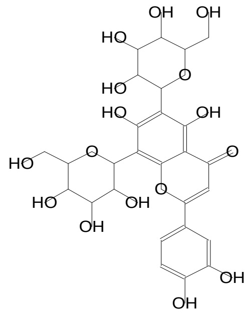

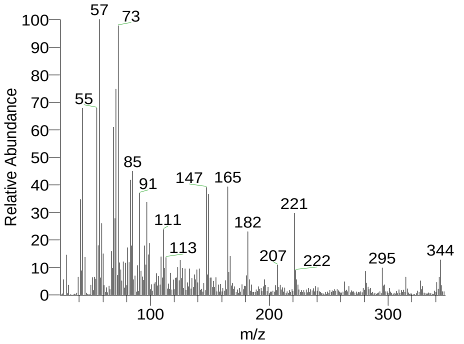

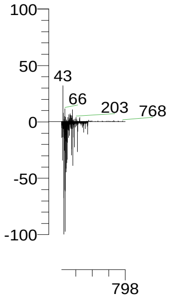

QUERCETIN 7,3',4'-TRIMETHOXY
Formula C18H16O7, MW 344, CAS# 6068-80-0, Entry# 461110
4H-1-Benzopyran-4-one, 2-(3,4-dimethoxyphenyl)-3,5-dihydroxy-7-methoxy- (CAS)

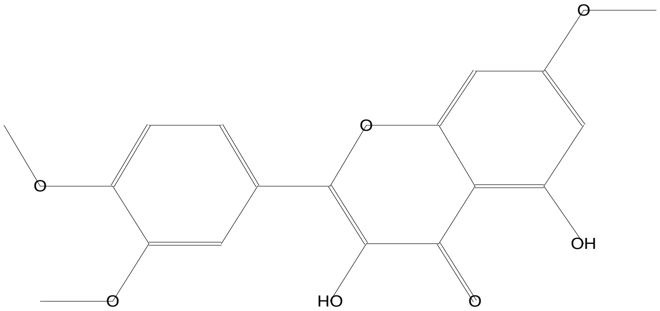

There is no signature data to report.

Library Search Report

Hit Spectrum	Delta	Compound Structure

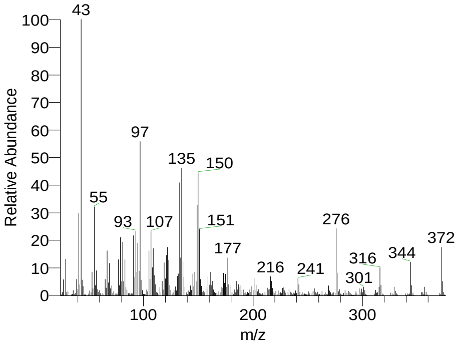

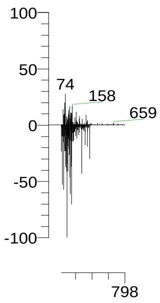

Cyclopropa[5,6]-A-nor-5à-androstane-3,7-dione, 3',6á-dihydro-17á-hydroxy-3',3'-dimethyl-, acetate
Formula C23H32O4, MW 372, CAS# 24634-23-9, Entry# 9890

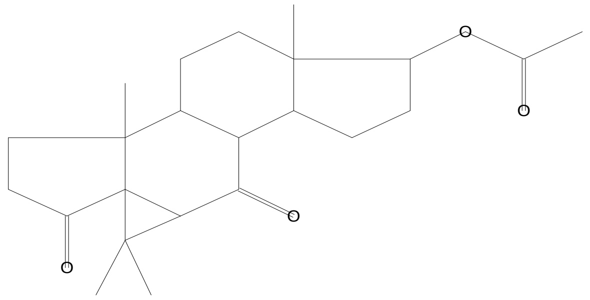

There is no signature data to report.

Library Search Report

RT	Probability	Compound Name	SI	Area %	Area	Molecular Weight	Molecular Formula	Library
32.27	11.67	Cystathionine-diTMS	476	0.32	1004912.30	366	C13H30N2O4SSi2	Wiley9
32.27	8.94	(1rs,3rs,4rs,8sr)-[3-(2-nitrophenyl)sulfenyl-11-oxatricyclo[6.2.1.0(2,7)]undec-2(7)-en-4-yl methyl ketone	469	0.32	1004912.30	345	C18H19NO4S	Wiley9
32.27	6.85	CIS-CYCLOHEXANE-1,3-DIOL TMS (CALLED TRANS)	462	0.32	1004912.30	260	C12H28O2Si2	Wiley9

Faten-212 #8018 RT: 32.27 AV: 1 RF: 6.00, 3 NL: 2.04E4
F: {0,0} + c EI Full ms [40.00-800.00]

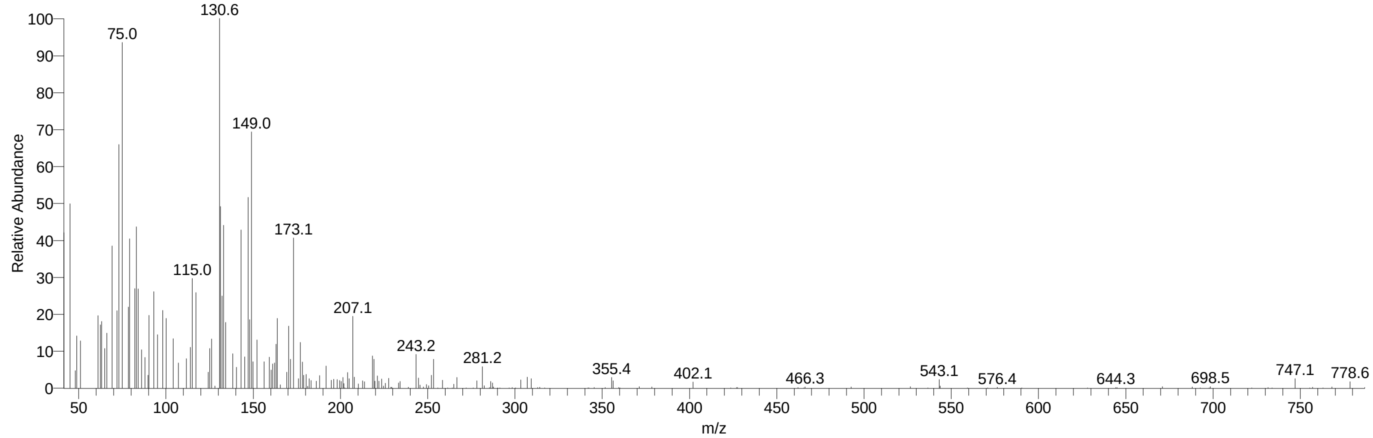

Hit Spectrum	Delta	Compound Structure

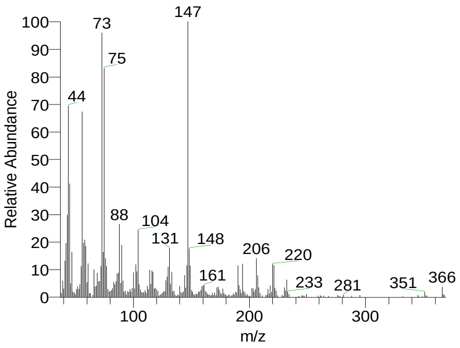

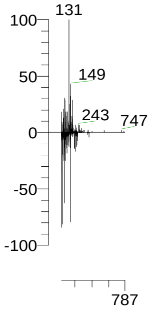

Cystathionine-diTMS
Formula C13H30N2O4SSi2, MW 366, CAS# 73090-79-6, Entry# 496338
Cystathionine, bis(triemthylsilyl) ester

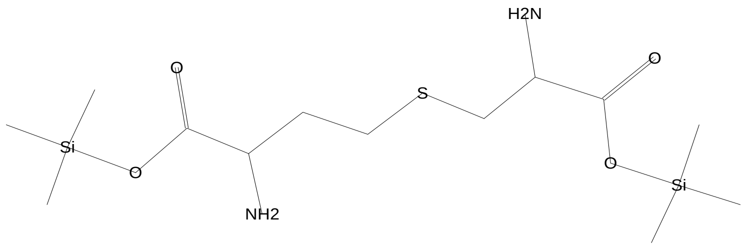

(1rs,3rs,4rs,8sr)-[3-(2-nitrophenyl)sulfenyl-11-oxatricyclo[6.2.1.0(2,7)]undec-2(7)-en-4-yl methyl ketone
Formula C18H19NO4S, MW 345, CAS# 105308-79-0, Entry# 463100

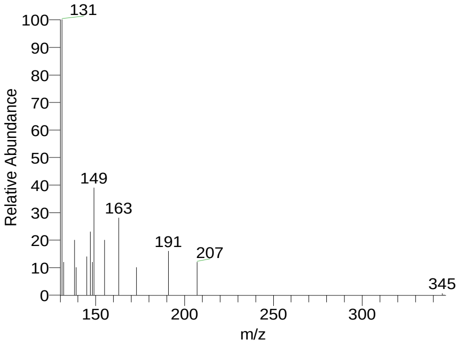

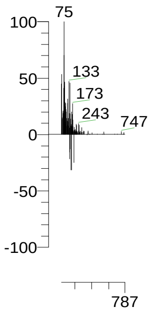

There is no signature data to report.

Library Search Report

Hit Spectrum	Delta	Compound Structure

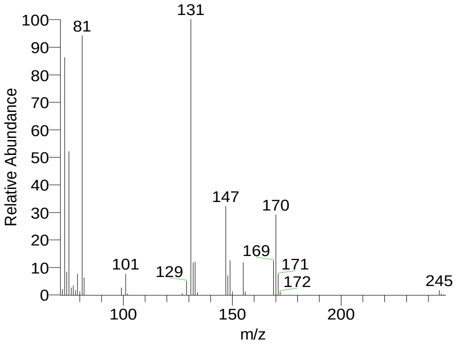

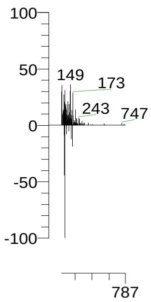

CIS-CYCLOHEXANE-1,3-DIOL TMS (CALLED TRANS)
Formula C12H28O2Si2, MW 260, CAS# 29753-63-7, Entry# 281961
Silane, [1,4-cyclohexanediylbis(oxy)]bis[trimethyl-, cis- (CAS)

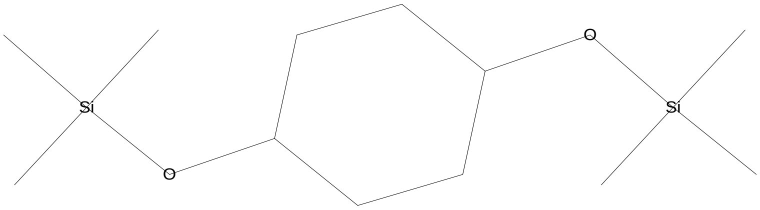

There is no signature data to report.

Library Search Report

RT	Probability	Compound Name	SI	Area %	Area	Molecular Weight	Molecular Formula	Library
32.58	6.86	3-Acetoxybutyric acid, trimethylsilyl ester	607	1.51	4786416.64	218	C9H18O4Si	mainlib
32.58	6.86	3-Acetoxybutyric acid, trimethylsilyl ester	607	1.51	4786416.64	218	C9H18O4Si	Wiley9
32.58	3.53	4-Penten-2-ol, trimethylsilyl ether	589	1.51	4786416.64	158	C8H18OSi	mainlib

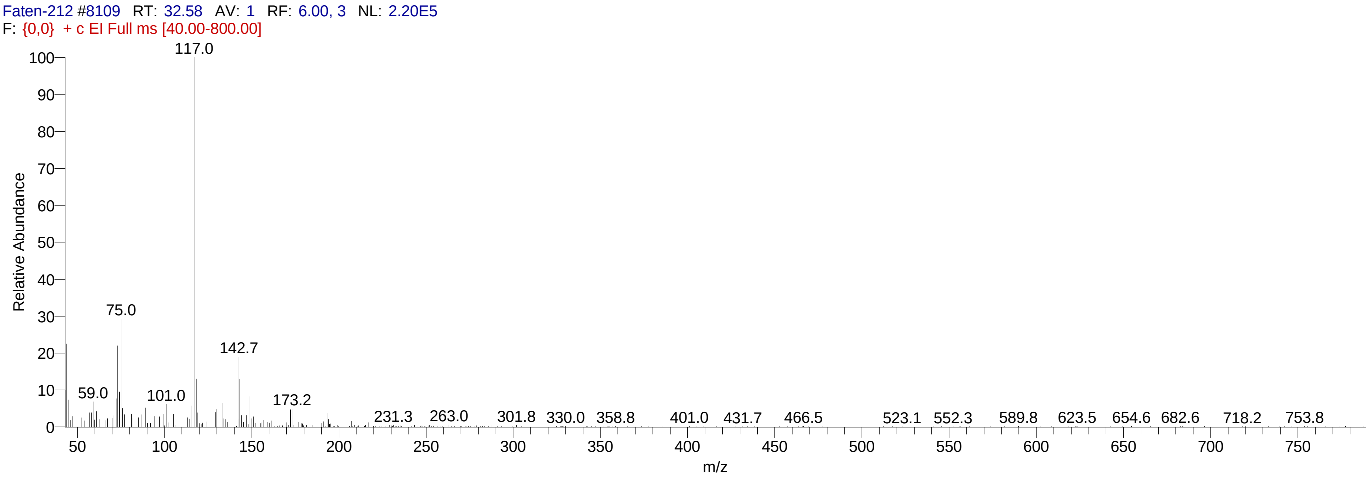

Hit Spectrum | Delta | Compound Structure

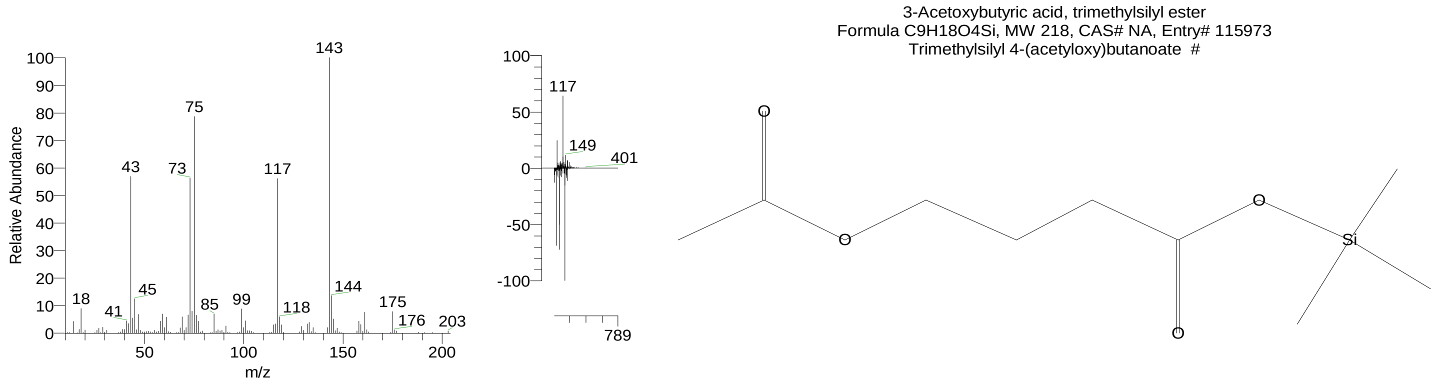

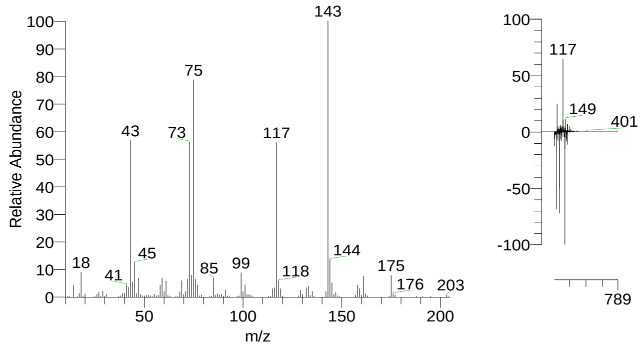

3-Acetoxybutyric acid, trimethylsilyl ester
Formula C9H18O4Si, MW 218, CAS# NA, Entry# 181615
Trimethylsilyl 4-(acetyloxy)butanoate

There is no signature data to report.

Library Search Report

Hit Spectrum	Delta	Compound Structure

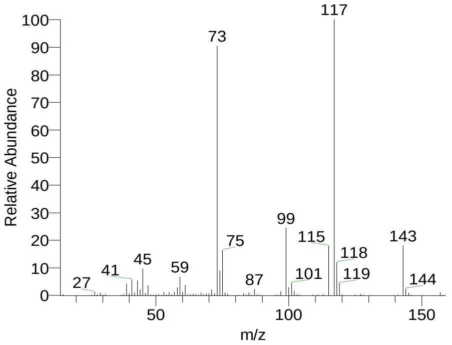

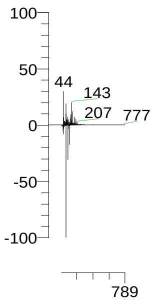

4-Penten-2-ol, trimethylsilyl ether
Formula C8H18OSi, MW 158, CAS# NA, Entry# 86826
Trimethyl[(1-methylbut-3-en-1-yl)oxy]silane #

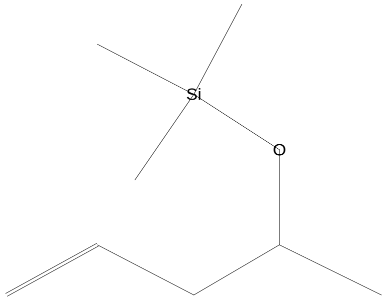

There is no signature data to report.

Library Search Report

RT	Probability	Compound Name	SI	Area %	Area	Molecular Weight	Molecular Formula	Library
32.86	9.41	7-ISOPROPENYL-1,4A-DIMETHYL-4,4A,5,6,7,8-HEXAHYDRO-2(3H)-NAPHTHALENONE	406	0.14	441735.06	218	C15H21DO	Wiley9
32.86	7.39	l-Galactopyranose, 6-deoxy-1,2-bis-O-(trimethylsilyl)-, cyclic methylboronate	400	0.14	441735.06	332	C13H29BO5Si2	mainlib
32.86	7.39	l-Galactopyranose, 6-deoxy-1,2-bis-O-(trimethylsilyl)-, cyclic methylboronate (CAS)	400	0.14	441735.06	332	C13H29BO5Si2	Wiley9

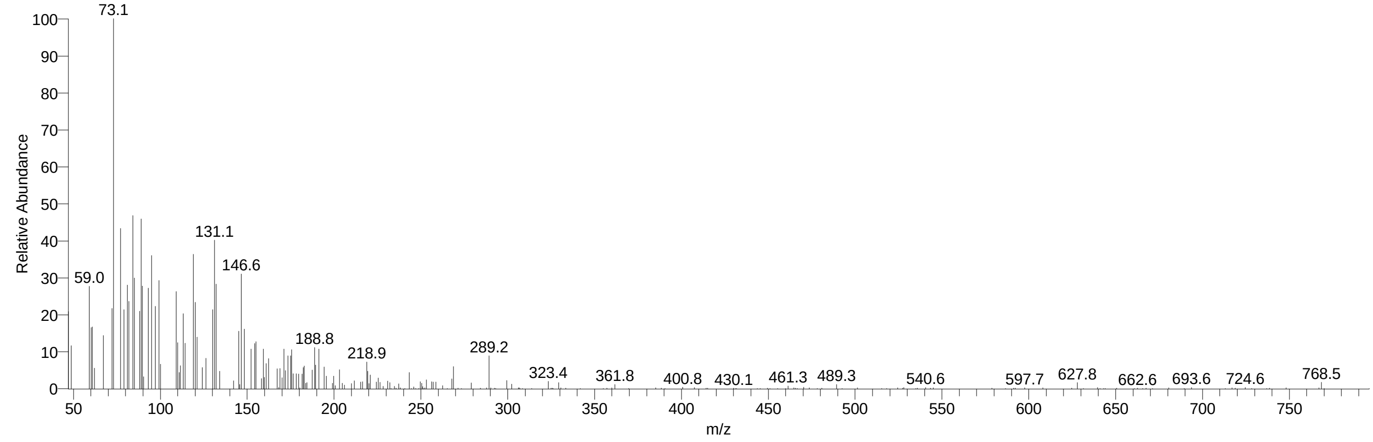

Hit Spectrum	Delta	Compound Structure

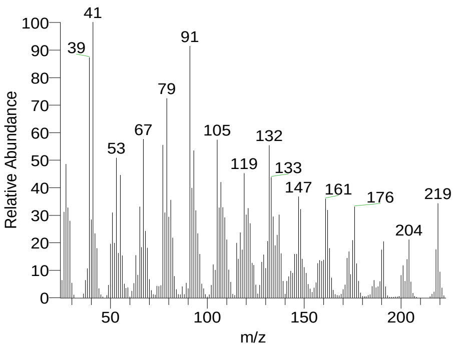

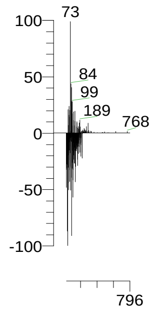

7-ISOPROPENYL-1,4A-DIMETHYL-4,4A,5,6,7,8-HEXAHYDRO-2(3H)-NAPHTHALENONE
Formula C15H21DO, MW 218, CAS# NA, Entry# 184309

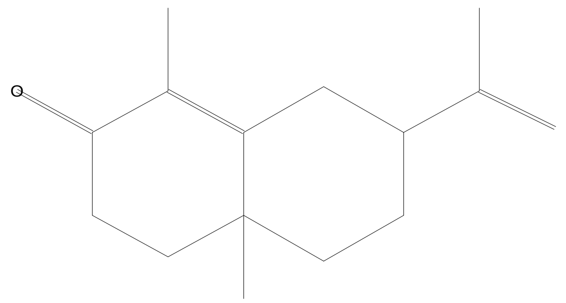

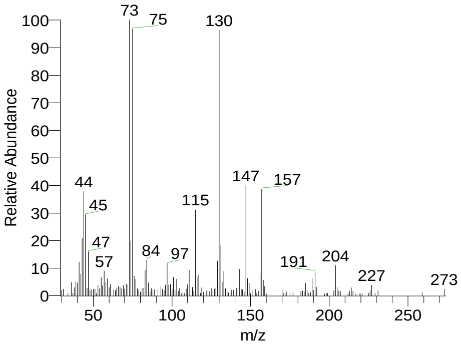

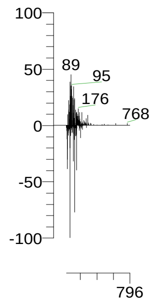

l-Galactopyranose, 6-deoxy-1,2-bis-O-(trimethylsilyl)-, cyclic methylboronate
Formula C13H29BO5Si2, MW 332, CAS# 56196-83-9, Entry# 37729

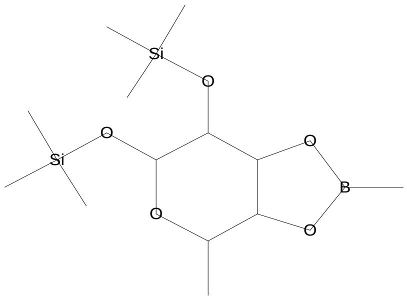

There is no signature data to report.

Library Search Report

Hit Spectrum	Delta	Compound Structure

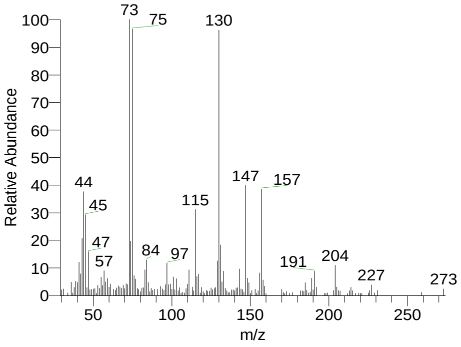

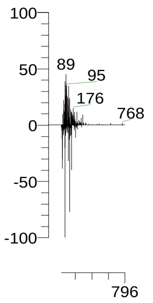

l-Galactopyranose, 6-deoxy-1,2-bis-O-(trimethylsilyl)-, cyclic methylboronate (CAS)
Formula C13H29BO5Si2, MW 332, CAS# 56196-83-9, Entry# 438915
FUCOPYRANOSE-3,4-METHYLBORONATE-1,2-DITMS

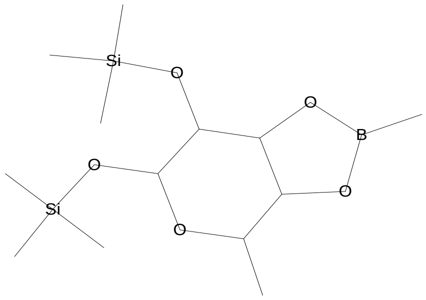

There is no signature data to report.

Library Search Report

RT	Probability	Compound Name	SI	Area %	Area	Molecular Weight	Molecular Formula	Library
33.51	11.14	á-D-Galactopyranoside, methyl 2,3-bis-O-(trimethylsilyl)-, cyclic butylboronate	476	0.29	916758.17	404	C17H37BO6Si2	mainlib
33.51	11.14	á-D-Galactopyranoside, methyl 2,3-bis-O-(trimethylsilyl)-, cyclic butylboronate (CAS)	476	0.29	916758.17	404	C17H37BO6Si2	Wiley9
33.51	10.71	à-D-Galactopyranose, 1,2,3-tris-O-(trimethylsilyl)-, cyclic methylboronate	475	0.29	916758.17	420	C16H37BO6Si3	mainlib

Faten-212 #8384 RT: 33.51 AV: 1 RF: 6.00, 3 NL: 6.28E4
F: {0,0} + c EI Full ms [40.00-800.00]

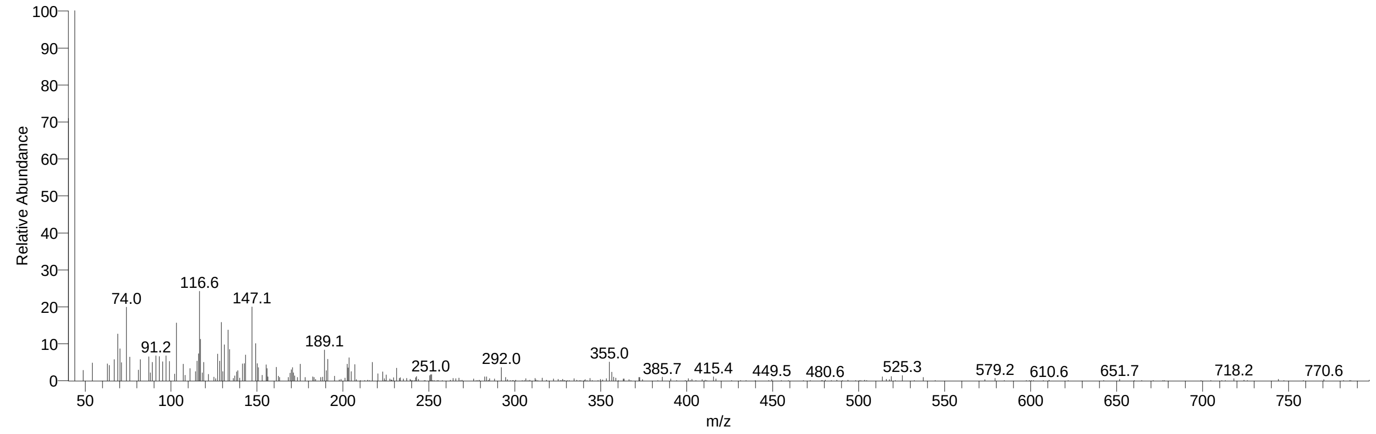

Hit Spectrum | Delta | Compound Structure

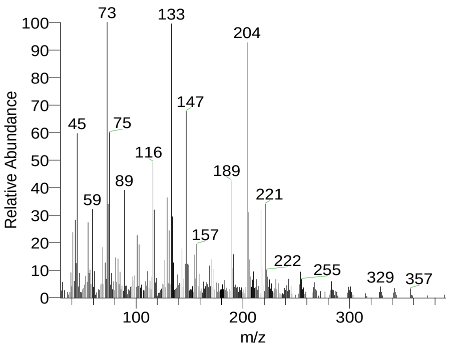

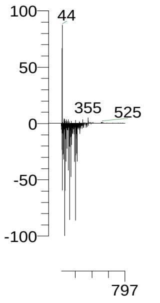

á-D-Galactopyranoside, methyl 2,3-bis-O-(trimethylsilyl)-, cyclic butylboronate
Formula C17H37BO6Si2, MW 404, CAS# 56211-10-0, Entry# 38594

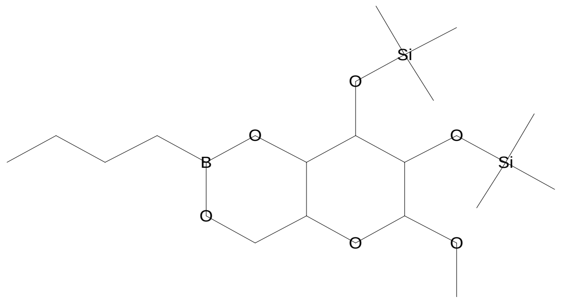

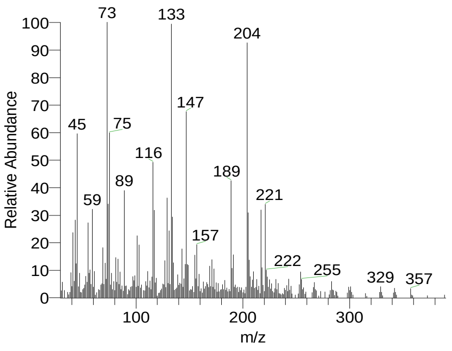

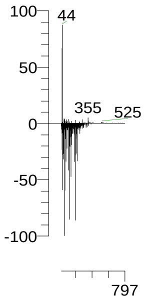

á-D-Galactopyranoside, methyl 2,3-bis-O-(trimethylsilyl)-, cyclic butylboronate (CAS)
Formula C17H37BO6Si2, MW 404, CAS# 56211-10-0, Entry# 546606
B-GALACTOPYRANOSIDE-1-METHYL-4,6-BUTYLBORONATE-2,3-DITMS

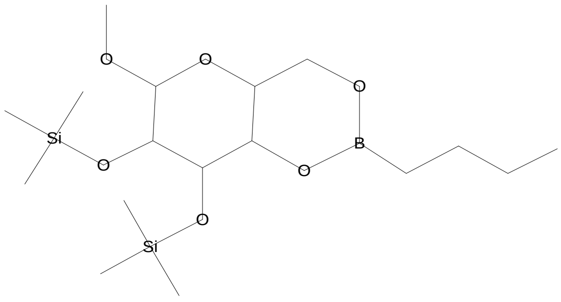

There is no signature data to report.

Library Search Report

Hit Spectrum	Delta	Compound Structure

à-D-Galactopyranose, 1,2,3-tris-O-(trimethylsilyl)-, cyclic methylboronate
Formula C16H37BO6Si3, MW 420, CAS# 56196-95-3, Entry# 37818

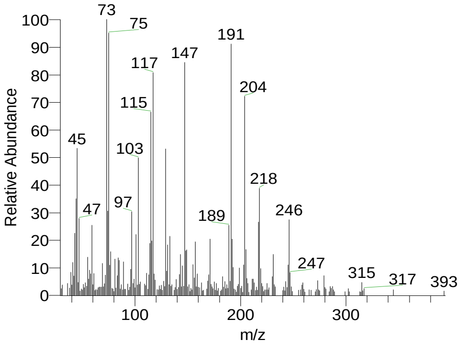

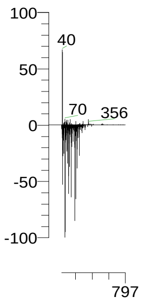

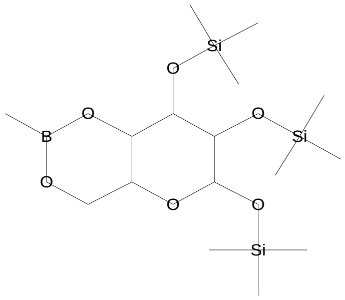

There is no signature data to report.

Library Search Report

RT	Probability	Compound Name	SI	Area %	Area	Molecular Weight	Molecular Formula	Library
34.35	10.17	Benzenesulfonamide, 4-amino-N-2-thiazolyl- (CAS)	397	0.15	483966.48	255	C9H9N3O2S2	Wiley9
34.35	7.79	TATP	390	0.15	483966.48	222	C9H18O6	mainlib
34.35	7.79	TATP	390	0.15	483966.48	222	C9H18O6	Wiley9

Faten-212 #8629 RT: 34.35 AV: 1 RF: 6.00, 3 NL: 4.81E4
F: {0,0} + c EI Full ms [40.00-800.00]

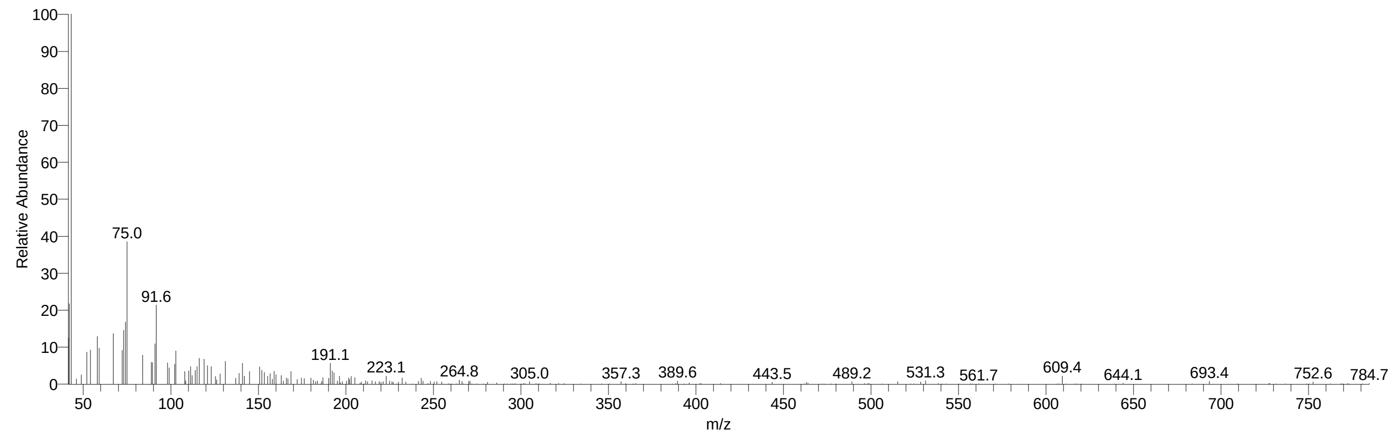

Hit Spectrum	Delta	Compound Structure

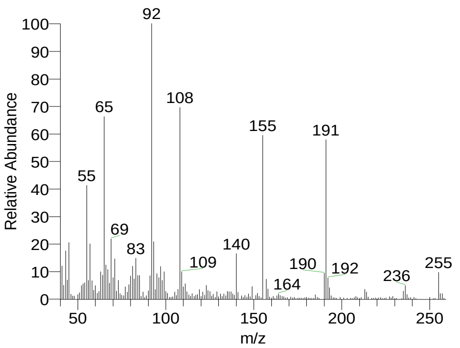

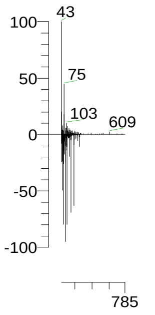

Benzenesulfonamide, 4-amino-N-2-thiazolyl- (CAS)
Formula C9H9N3O2S2, MW 255, CAS# 72-14-0, Entry# 270702
Duatok

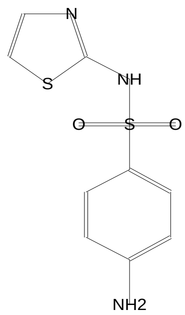

SI 390, RSI 755, mainlib, Entry# 7664, CAS# 17088-37-8, TATP

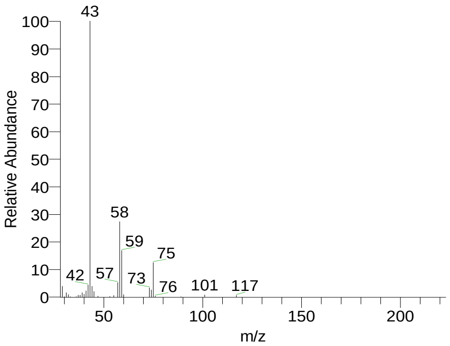

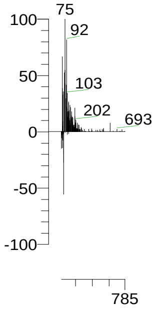

TATP
Formula C9H18O6, MW 222, CAS# 17088-37-8, Entry# 7664
Triacetone triperoxide

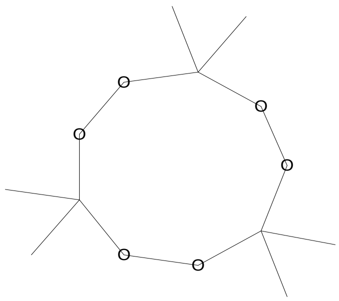

There is no signature data to report.

Library Search Report

Hit Spectrum | Delta | Compound Structure

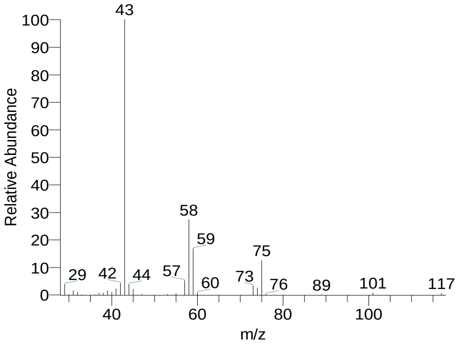

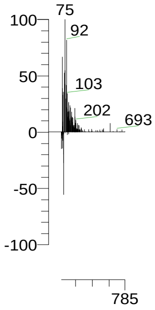

TATP
Formula C9H18O6, MW 222, CAS# 17088-37-8, Entry# 191468
Triacetone triperoxide

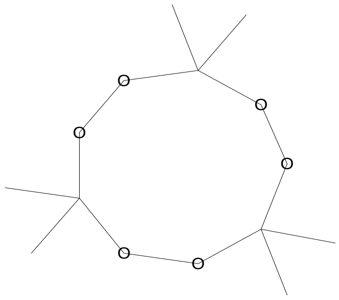

There is no signature data to report.

Library Search Report

RT	Probability	Compound Name	SI	Area %	Area	Molecular Weight	Molecular Formula	Library
34.54	7.32	Thiofanox	409	0.20	641881.22	218	C9H18N2O2S	mainlib
34.54	6.19	Benzoic acid, 4-tert-butyl-, 3,5-dichloro-4-pyridyl ester	405	0.20	641881.22	323	C16H15Cl2NO2	mainlib
34.54	6.19	Ethyl 9,9-diformylnona-2,4,6,8-tetraenoate	405	0.20	641881.22	234	C13H14O4	mainlib

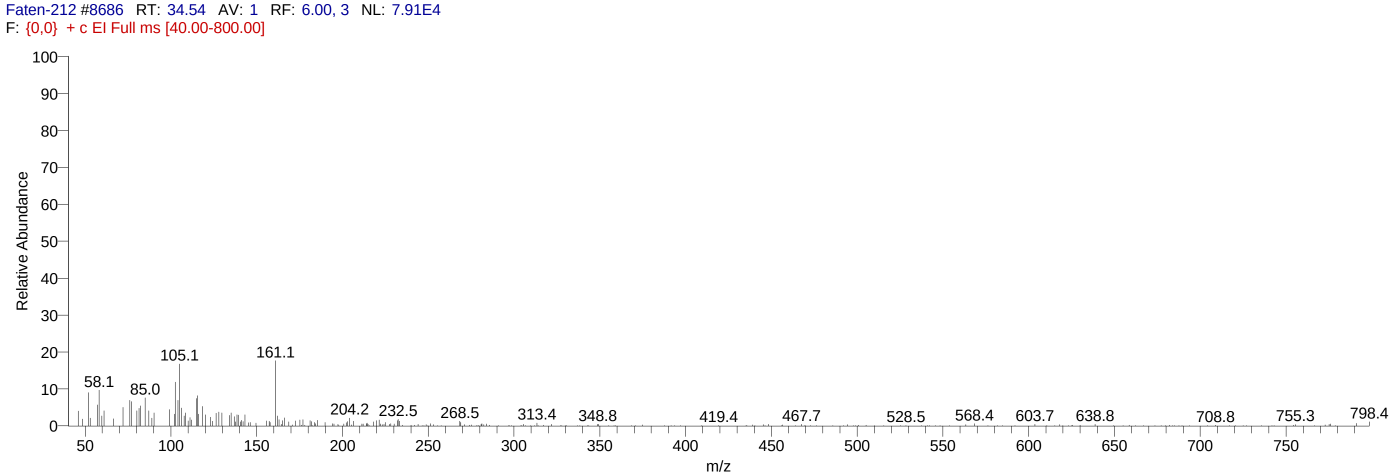

Hit Spectrum	Delta	Compound Structure

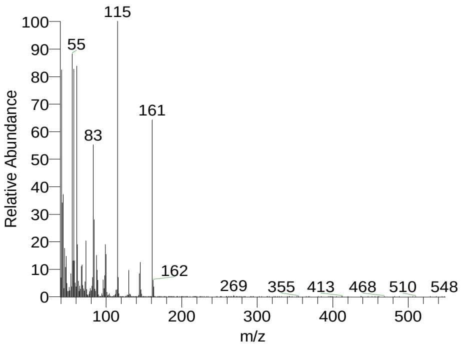

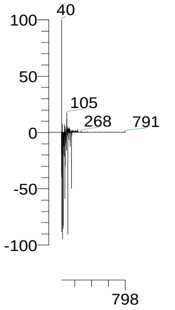

Thiofanox
Formula C9H18N2O2S, MW 218, CAS# 39196-18-4, Entry# 82603
2-Butanone, 3,3-dimethyl-1-(methylthio)-, O-[(methylamino)carbonyl]oxime

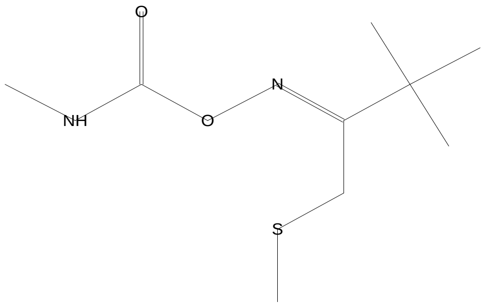

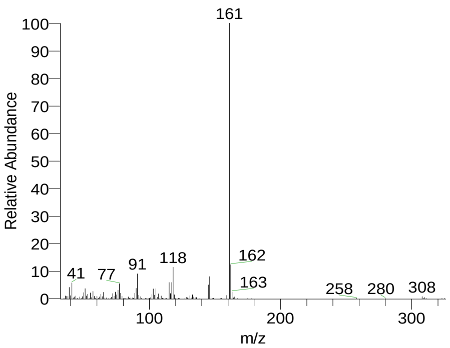

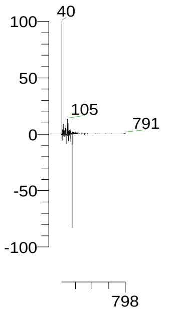

Benzoic acid, 4-tert-butyl-, 3,5-dichloro-4-pyridyl ester
Formula C16H15Cl2NO2, MW 323, CAS# NA, Entry# 133436
3,5-Dichloro-4-pyridinyl 4-tert-butylbenzoate #

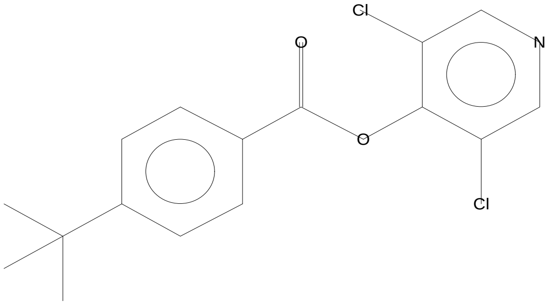

There is no signature data to report.

Library Search Report

Hit Spectrum	Delta	Compound Structure

Ethyl 9,9-diformylnona-2,4,6,8-tetraenoate
Formula C13H14O4, MW 234, CAS# 98834-95-8, Entry# 43060
Ethyl (2E,4E,6E)-9-formyl-10-oxo-2,4,6,8-decatetraenoate #

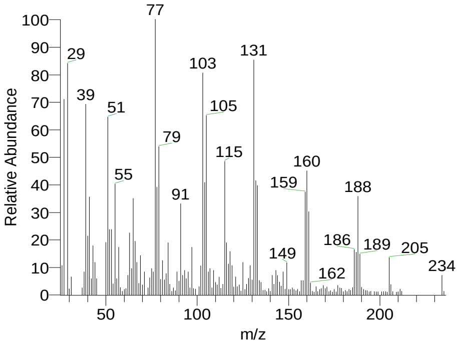

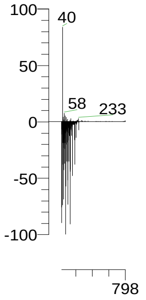

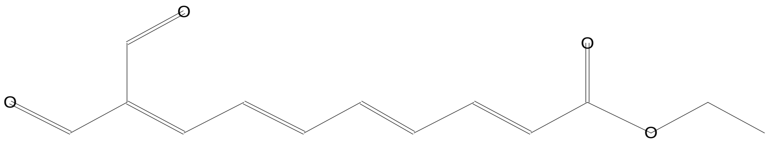

There is no signature data to report.

Library Search Report

RT	Probability	Compound Name	SI	Area %	Area	Molecular Weight	Molecular Formula	Library
34.67	15.17	2-Benzyl-3-hydroxy-2-methylsuccinic acid, diethyl ester	530	0.95	3009196.19	294	C16H22O5	mainlib
34.67	15.17	2-Benzyl-3-hydroxy-2-methylsuccinic acid, diethyl ester	530	0.95	3009196.19	294	C16H22O5	Wiley9
34.67	12.23	1,1,1-Tris(hydroxymethyl)propane, tris(trimethylsilyl) ether	525	0.95	3009196.19	350	C15H38O3Si3	mainlib

Faten-212 #8724 RT: 34.67 AV: 1 RF: 6.00, 3 NL: 9.24E4
F: {0,0} + c EI Full ms [40.00-800.00]

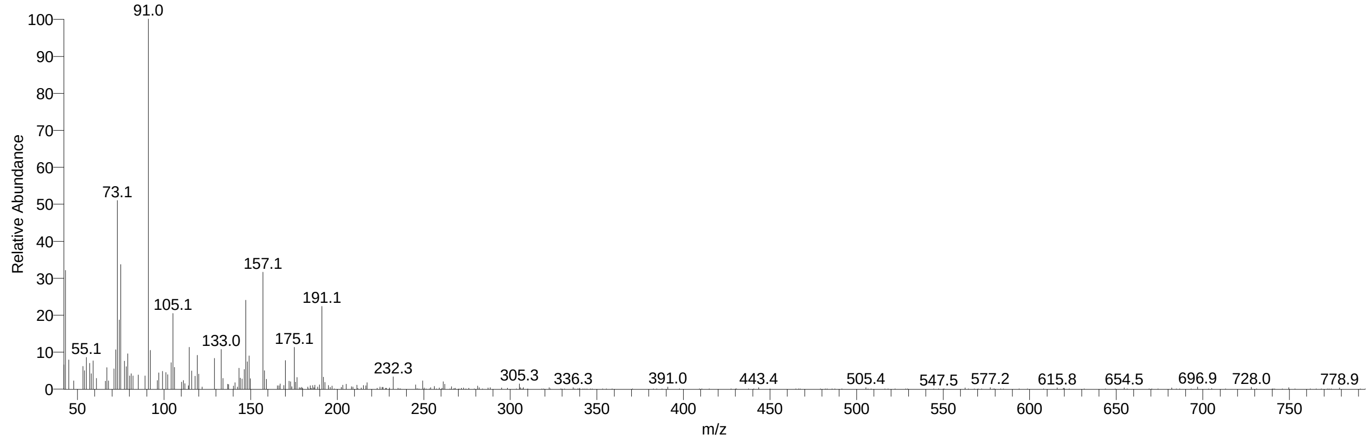

Hit Spectrum | Delta | Compound Structure

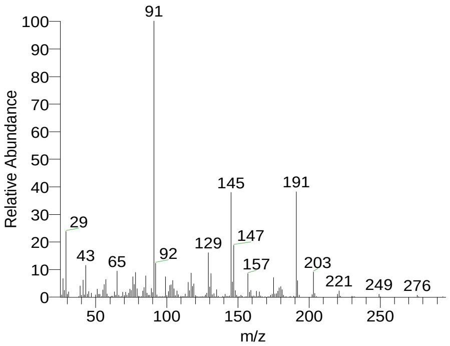

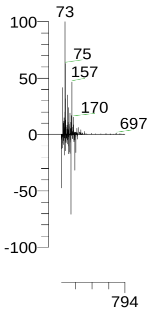

2-Benzyl-3-hydroxy-2-methylsuccinic acid, diethyl ester
Formula C16H22O5, MW 294, CAS# 111833-01-3, Entry# 57612
Diethyl 2-benzyl-3-hydroxy-2-methylsuccinate #

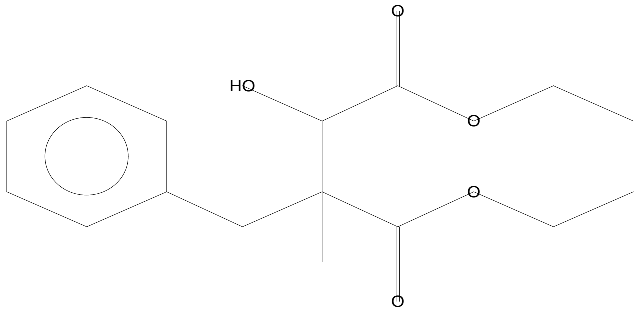

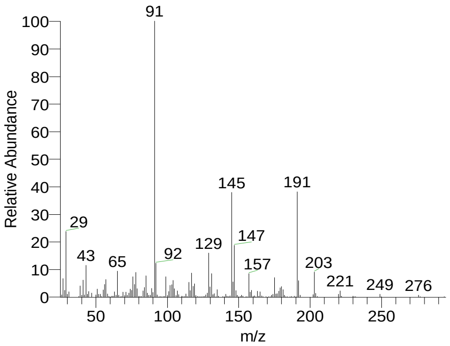

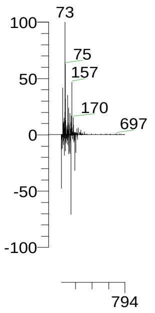

2-Benzyl-3-hydroxy-2-methylsuccinic acid, diethyl ester
Formula C16H22O5, MW 294, CAS# 111833-01-3, Entry# 360713
2-BENZYL-3-HYDROXY-2-METHYL-SUCCINIC ACID DIETHYL ESTER

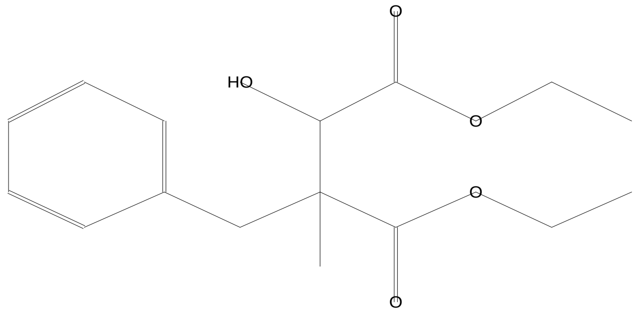

There is no signature data to report.

Library Search Report

Hit Spectrum	Delta	Compound Structure

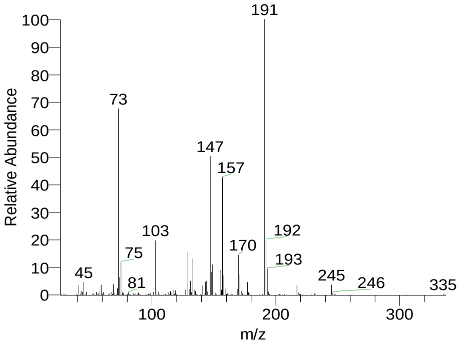

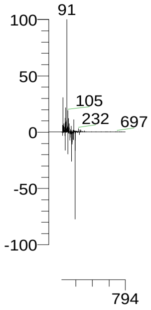

1,1,1-Tris(hydroxymethyl)propane, tris(trimethylsilyl) ether
Formula C15H38O3Si3, MW 350, CAS# NA, Entry# 156424

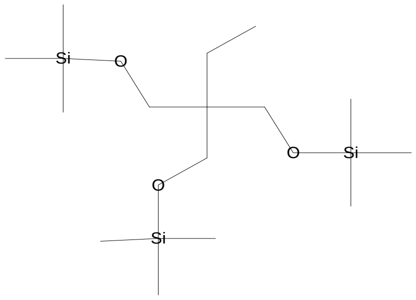

There is no signature data to report.

Library Search Report

RT	Probability	Compound Name	SI	Area %	Area	Molecular Weight	Molecular Formula	Library
34.92	6.91	Decane, 1,9-bis[(trimethylsilyl)oxy]-	658	6.99	22207131.85	318	C16H38O2Si2	mainlib
34.92	6.91	Decane, 1,9-bis[(trimethylsilyl)oxy]-	658	6.99	22207131.85	318	C16H38O2Si2	Wiley9
34.92	5.84	Hexane, 2,5-bis[(trimethylsilyl)oxy]-	654	6.99	22207131.85	262	C12H30O2Si2	mainlib

Faten-212 #8798 RT: 34.92 AV: 1 RF: 6.00, 3 NL: 7.20E5
F: {0,0} + c EI Full ms [40.00-800.00]

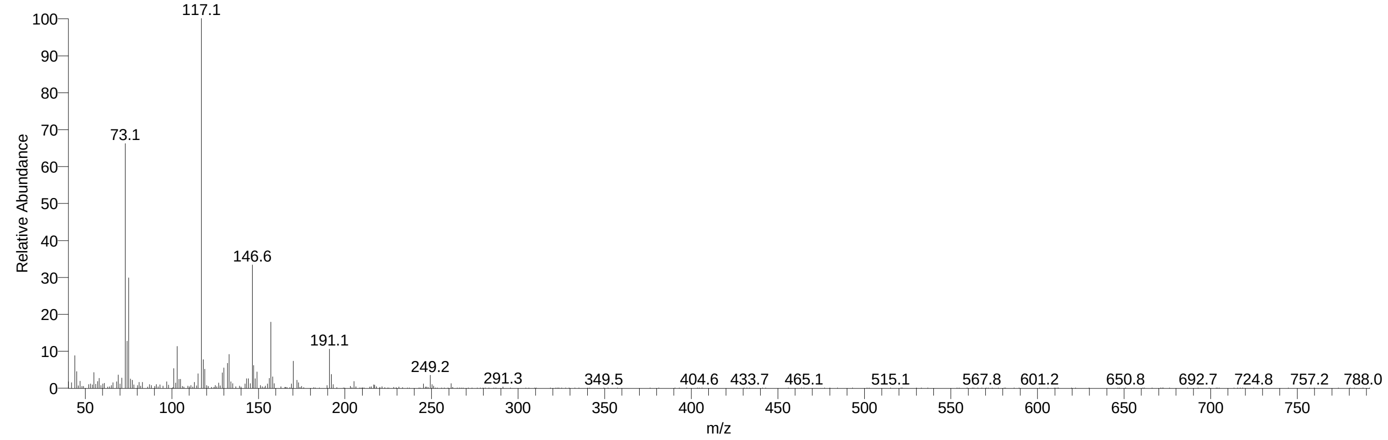

Hit Spectrum	Delta	Compound Structure

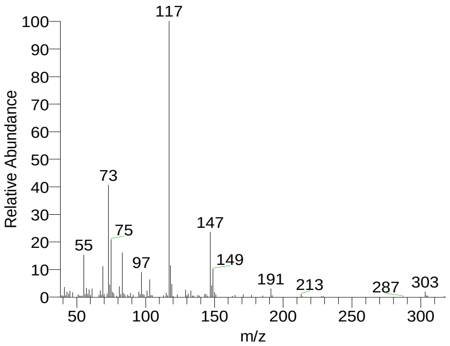

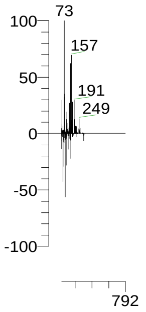

Decane, 1,9-bis[(trimethylsilyl)oxy]-
Formula C16H38O2Si2, MW 318, CAS# NA, Entry# 86841
2,2,4,14,14-Pentamethyl-3,13-dioxa-2,14-disilapentadecane #

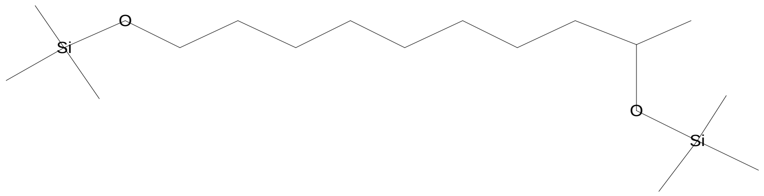

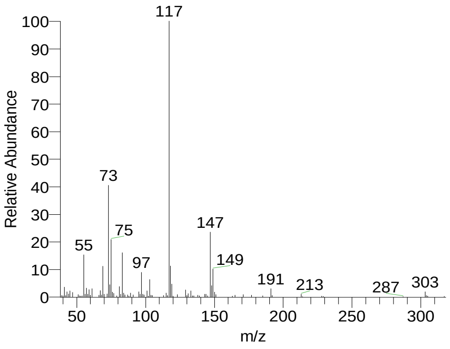

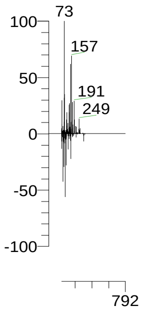

Decane, 1,9-bis[(trimethylsilyl)oxy]-
Formula C16H38O2Si2, MW 318, CAS# NA, Entry# 412213
2,2,4,14,14-Pentamethyl-3,13-dioxa-2,14-disilapentadecane

There is no signature data to report.

Library Search Report

Hit Spectrum	Delta	Compound Structure

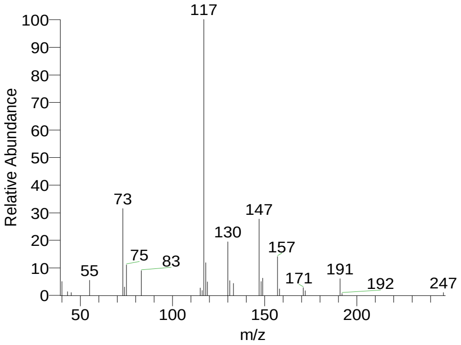

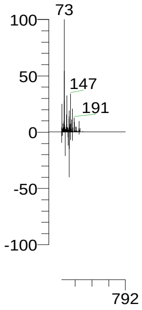

Hexane, 2,5-bis[(trimethylsilyl)oxy]-
Formula C12H30O2Si2, MW 262, CAS# 66956-94-3, Entry# 86851
2,2,4,7,9,9-Hexamethyl-3,8-dioxa-2,9-disiladecane #

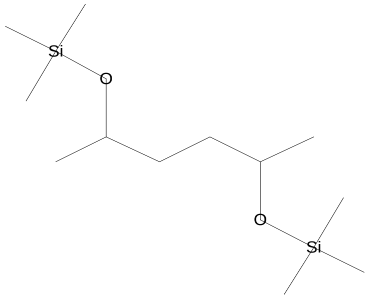

There is no signature data to report.

Library Search Report

RT	Probability	Compound Name	SI	Area %	Area	Molecular Weight	Molecular Formula	Library
35.12	6.43	3-Hydroxymethyl-2-trimethylsilyloxypentane	559	2.63	8352797.36	190	C9H22O2Si	mainlib
35.12	4.67	3-Hydroxymethyl-2-trimethylsilyloxypentane	550	2.63	8352797.36	190	C9H22O2Si	Wiley9
35.12	3.67	à-D-Mannopyranoside, methyl, cyclic 2,3:4,6-bis(butylboronate) (CAS)	544	2.63	8352797.36	326	C15H28B2O6	Wiley9

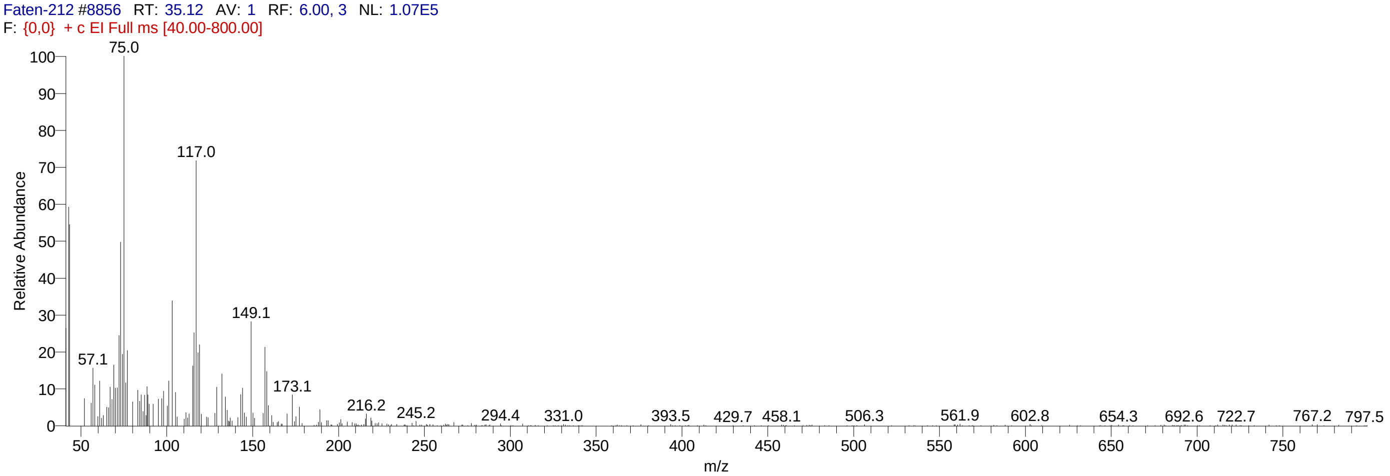

Hit Spectrum | Delta | Compound Structure

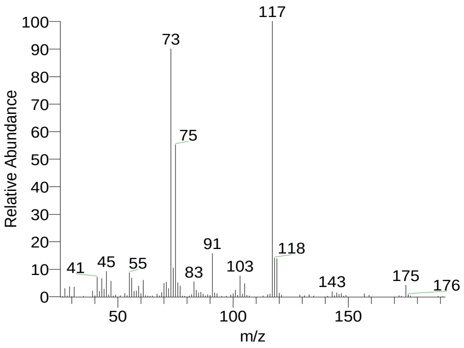

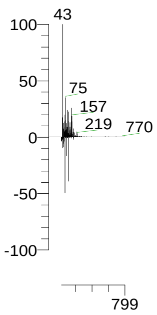

3-Hydroxymethyl-2-trimethylsilyloxypentane
Formula C9H22O2Si, MW 190, CAS# NA, Entry# 86781
2-Ethyl-3-[(trimethylsilyl)oxy]-1-butanol #

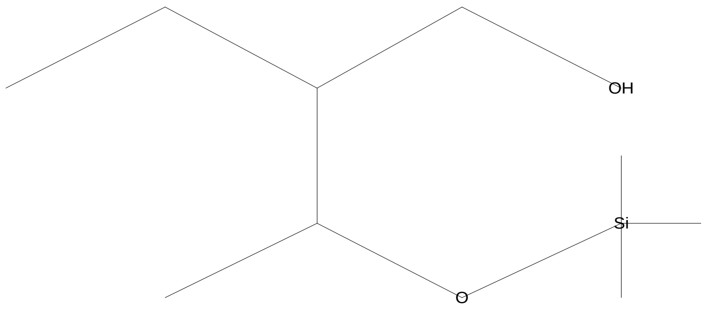

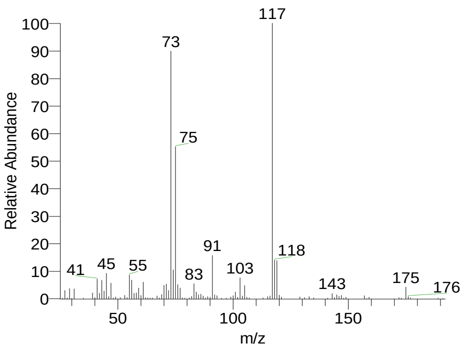

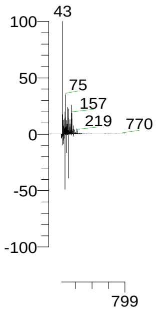

3-Hydroxymethyl-2-trimethylsilyloxypentane
Formula C9H22O2Si, MW 190, CAS# NA, Entry# 120448
2-Ethyl-3-[(trimethylsilyl)oxy]-1-butanol

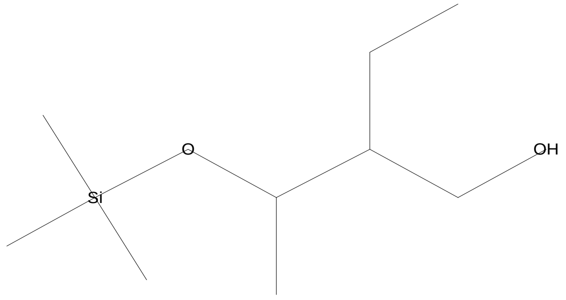

There is no signature data to report.

Library Search Report

Hit Spectrum	Delta	Compound Structure

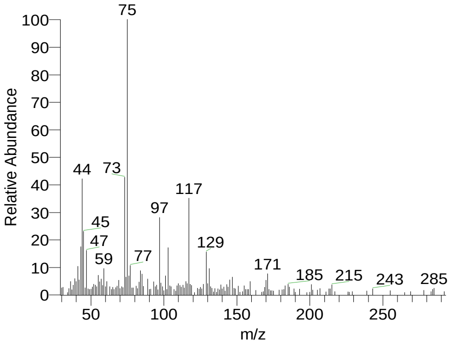

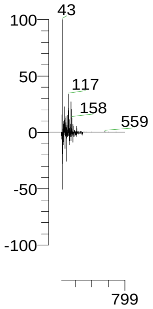

à-D-Mannopyranoside, methyl, cyclic 2,3:4,6-bis(butylboronate) (CAS)
Formula C15H28B2O6, MW 326, CAS# 54400-84-9, Entry# 427572
A-MANNOPYRANOSIDE-1-METHYL-2,3-4,6-DI-BUTYLBORONATE

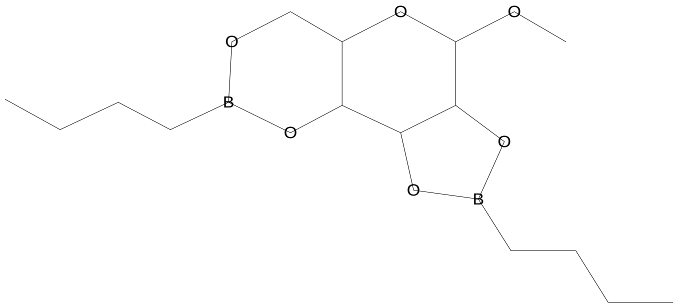

There is no signature data to report.

Library Search Report

RT	Probability	Compound Name	SI	Area %	Area	Molecular Weight	Molecular Formula	Library
35.96	12.81	Benzene, (3-octylundecyl)-	482	0.54	1713825.63	344	C25H44	mainlib
35.96	12.81	Benzene, (3-octylundecyl)- (CAS)	482	0.54	1713825.63	344	C25H44	Wiley9
35.96	8.79	2,5-Octadecadiynoic acid, methyl ester	471	0.54	1713825.63	290	C19H30O2	mainlib

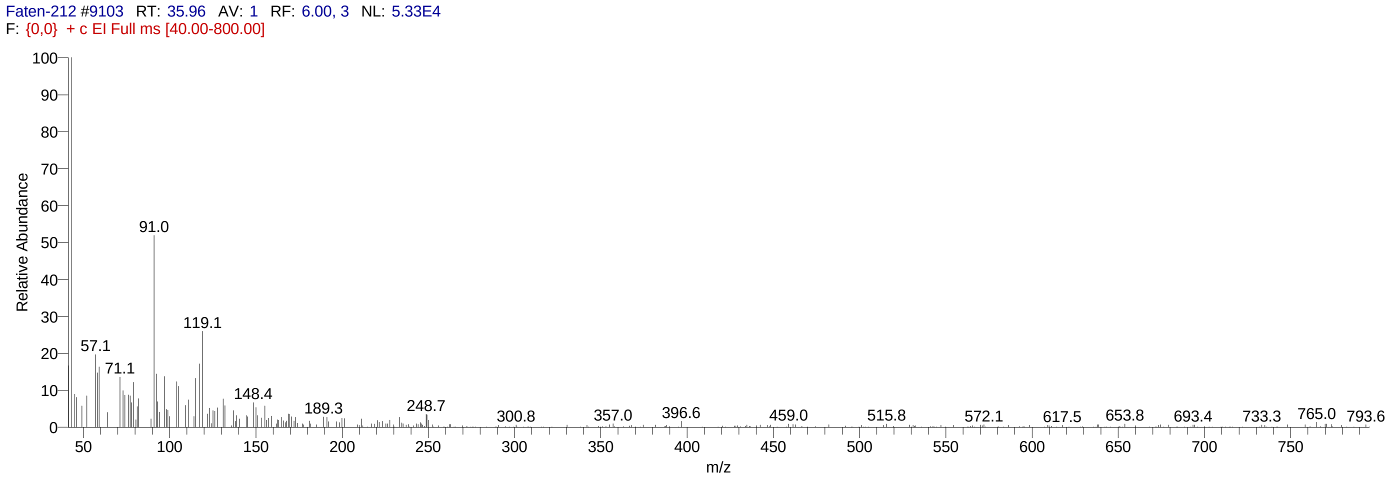

Hit Spectrum	Delta	Compound Structure

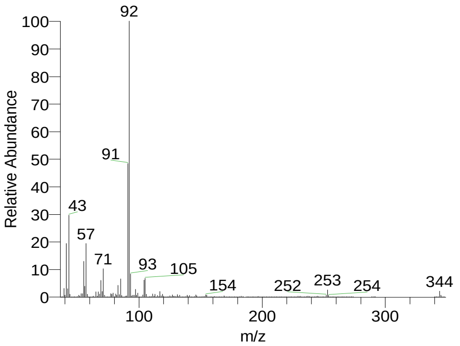

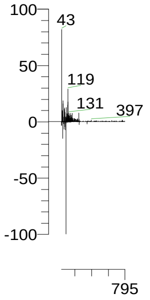

Benzene, (3-octylundecyl)-
Formula C25H44, MW 344, CAS# 5637-96-7, Entry# 59236
Heptadecane, 9-phenethyl-

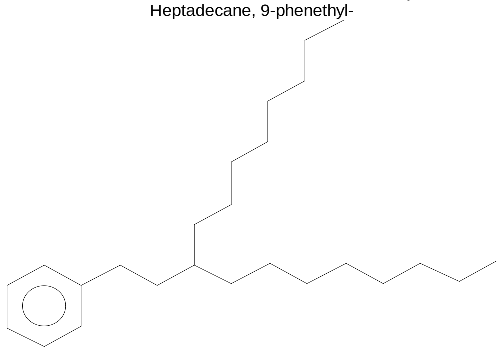

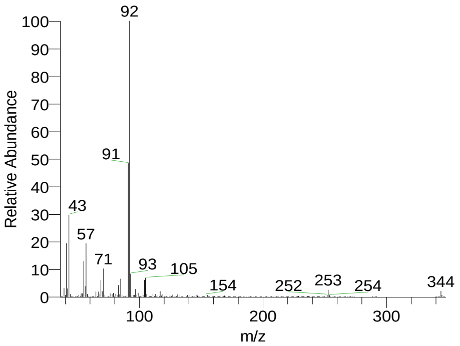

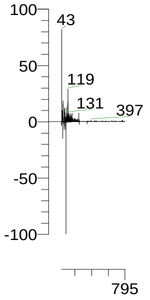

Benzene, (3-octylundecyl)- (CAS)
Formula C25H44, MW 344, CAS# 5637-96-7, Entry# 462591
Heptadecane, 9-phenethyl-

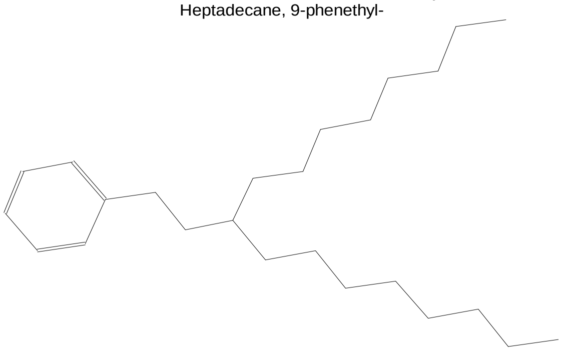

There is no signature data to report.

Library Search Report

Hit Spectrum	Delta	Compound Structure

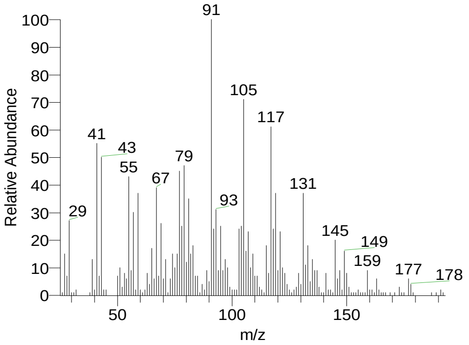

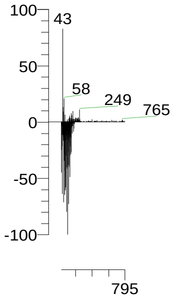

2,5-Octadecadiynoic acid, methyl ester
Formula C19H30O2, MW 290, CAS# 57156-91-9, Entry# 55436
Methyl 2,5-octadecadiynoate #

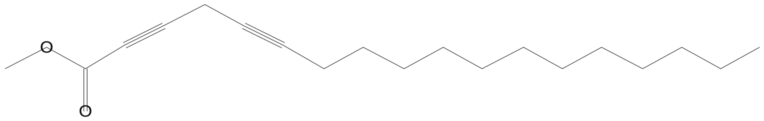

There is no signature data to report.

Library Search Report

RT	Probability	Compound Name	SI	Area %	Area	Molecular Weight	Molecular Formula	Library
36.22	17.00	SILICATE ANION TETRAMER	399	0.13	425012.15	888	C24H72O12Si12	Wiley9
36.22	10.64	QUERCETIN 7,3',4'-TRIMETHOXY	385	0.13	425012.15	344	C18H16O7	Wiley9
36.22	5.48	1-Monolinoleoylglycerol trimethylsilyl ether	367	0.13	425012.15	498	C27H54O4Si2	mainlib

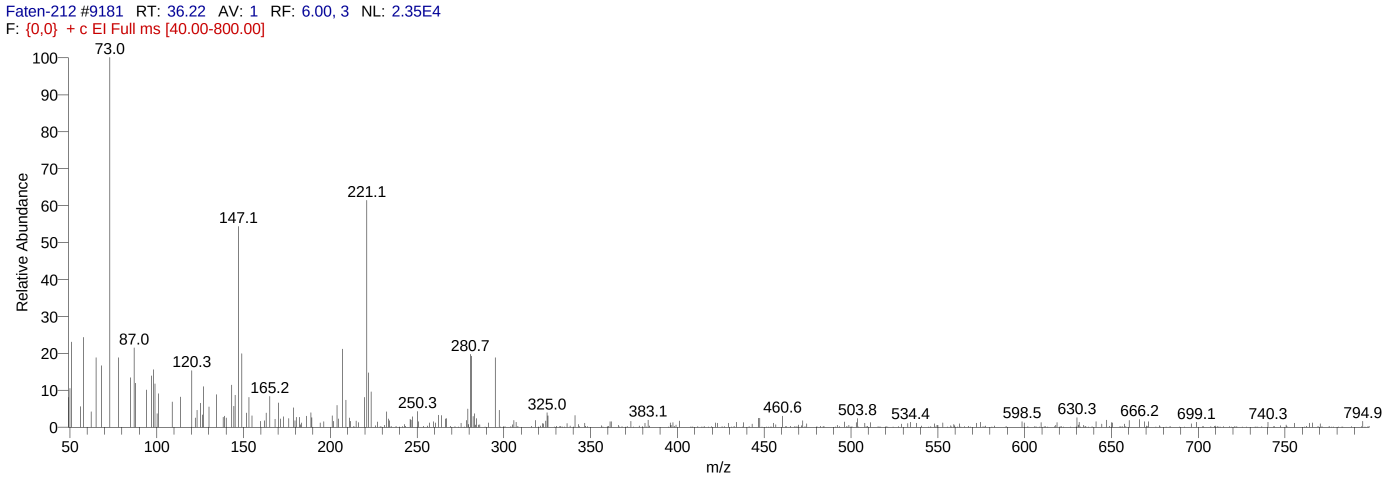

Hit Spectrum	Delta	Compound Structure

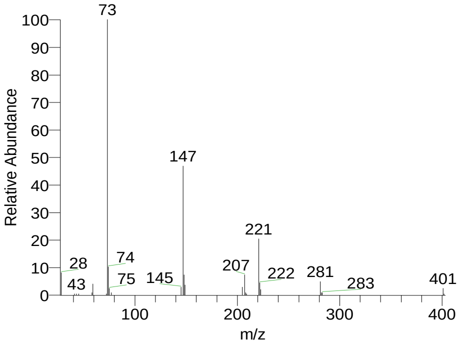

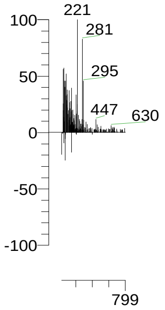

SILICATE ANION TETRAMER
Formula C24H72O12Si12, MW 888, CAS# NA, Entry# 660325

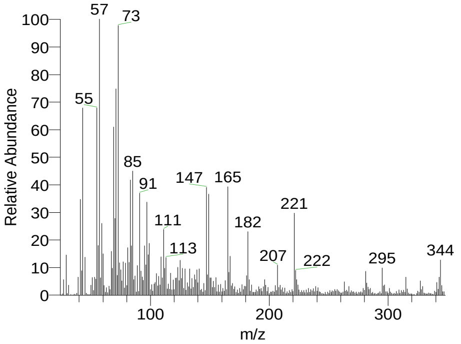

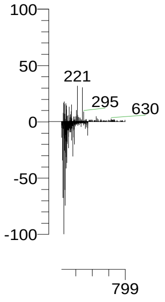

QUERCETIN 7,3',4'-TRIMETHOXY
Formula C18H16O7, MW 344, CAS# 6068-80-0, Entry# 461110
4H-1-Benzopyran-4-one, 2-(3,4-dimethoxyphenyl)-3,5-dihydroxy-7-methoxy- (CAS)

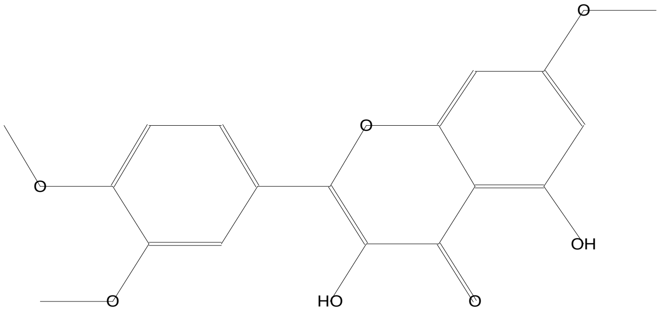

There is no signature data to report.

Library Search Report

Hit Spectrum	Delta	Compound Structure

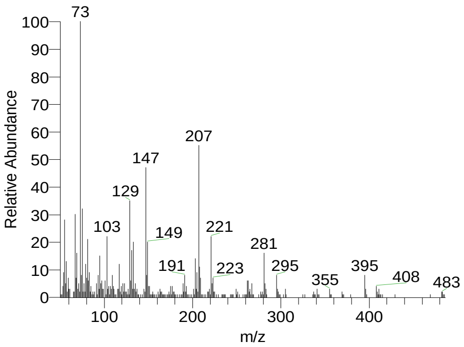

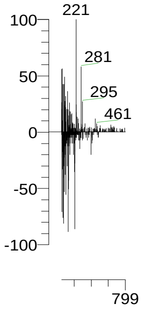

1-Monolinoleoylglycerol trimethylsilyl ether
Formula C27H54O4Si2, MW 498, CAS# 54284-45-6, Entry# 39425
9,12-Octadecadienoic acid (Z,Z)-, 2,3-bis[(trimethylsilyl)oxy]propyl ester

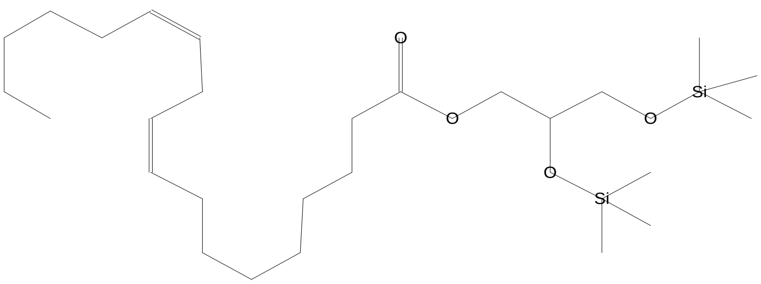

There is no signature data to report.

Library Search Report

RT	Probability	Compound Name	SI	Area %	Area	Molecular Weight	Molecular Formula	Library
36.30	6.52	Musk ketone	348	0.21	674855.80	294	C14H18N2O5	mainlib
36.30	6.02	Acetic acid, 17-(1-acetoxy-ethyl)-10,13-dimethyl-3-oxo-2,3,8,9,10,11,12,13,14,15,16,17-dodecahydro-1H-cyclopenta[a]phenanthren-11-yl (ester)	346	0.21	674855.80	414	C25H34O5	mainlib
36.30	6.02	Acetic acid, 17-(1-acetoxy-ethyl)-10,13-dimethyl-3-oxo-2,3,8,9,10,11,12,13,14,15,16,17-dodecahydro-1H-cyclopenta[a]phenanthren-11-yl	346	0.21	674855.80	414	C25H34O5	Wiley9

Faten-212 #9203 RT: 36.30 AV: 1 RF: 6.00, 3 NL: 8.64E4
F: {0,0} + c EI Full ms [40.00-800.00]

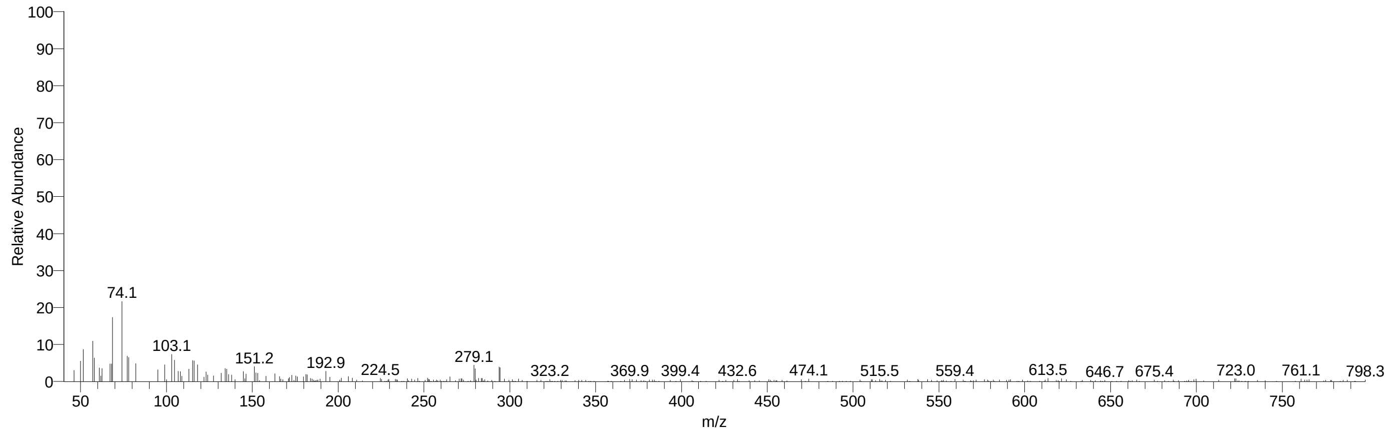

Hit Spectrum | Delta | Compound Structure

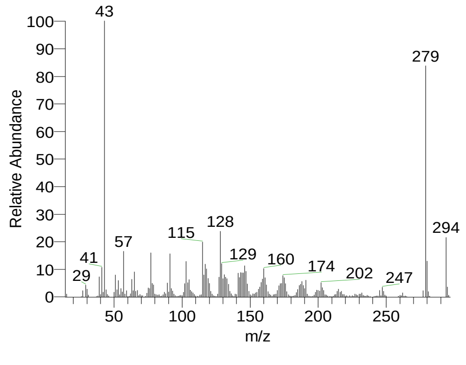

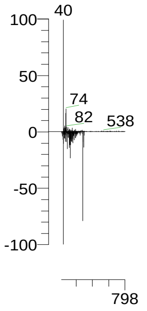

Musk ketone
Formula C14H18N2O5, MW 294, CAS# 81-14-1, Entry# 13458
Acetophenone, 4'-tert-butyl-2',6'-dimethyl-3',5'-dinitro-

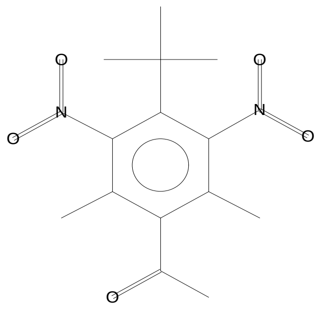

There is no signature data to report.

Library Search Report

Hit Spectrum	Delta	Compound Structure

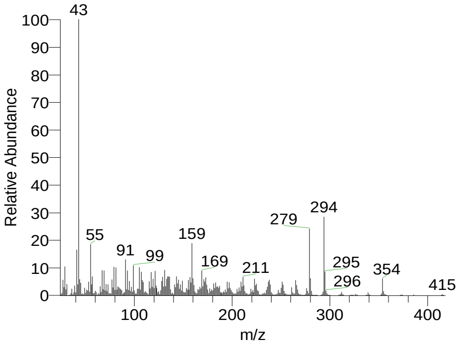

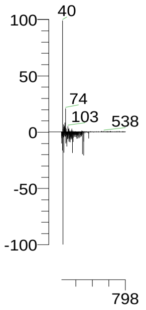

Formula C25H34O5, MW 414, CAS# NA, Entry# 13576
11-(Acetyloxy)-3-oxopregna-4,6-dien-20-yl acetate #

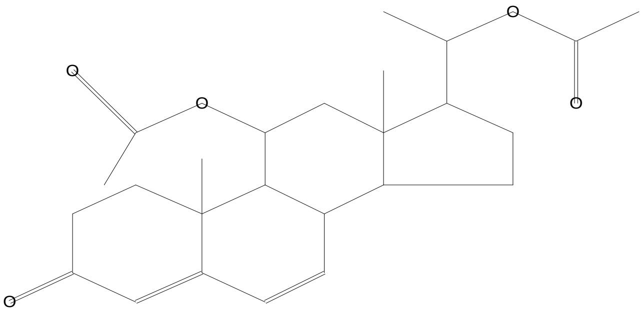

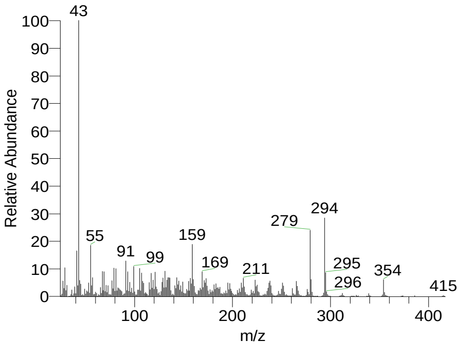

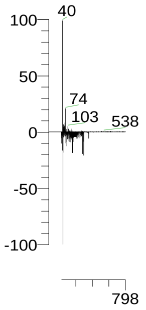

Formula C25H34O5, MW 414, CAS# NA, Entry# 558115
ACETIC ACID 17-(1-ACETOXY-ETHYL)-10,13-DIMETHYL-3-OXO-2,3,8,9,10,11,12,13,14,15,

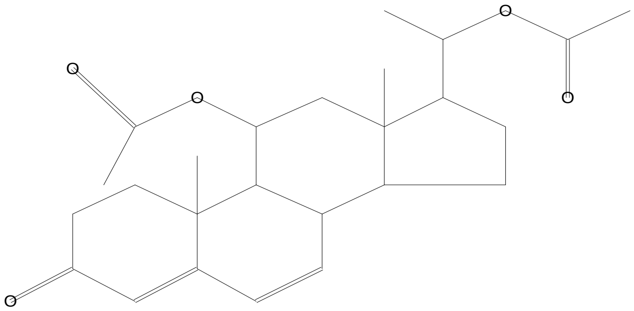

There is no signature data to report.

Library Search Report

RT	Probability	Compound Name	SI	Area %	Area	Molecular Weight	Molecular Formula	Library
36.48	55.96	1,4,7-Tris(3,5-di-tert-butyl-2-deuteriohydroxybenzyl)-1,4,7-triazacyclononane	428	0.14	451042.43	783	C51H72D9N3O3	Wiley9
36.48	7.44	Octaphenylfluorenone	354	0.14	451042.43	788	C61H40O	Wiley9
36.48	2.94	Naphthalene-2-carboxylic acid, 2,3,4,4a,5,6,7,8-octahydro-1,2-dicyano-3-oxo-4-phenyl-, ethyl ester	332	0.14	451042.43	348	C21H20N2O3	mainlib

Faten-212 #9258 RT: 36.48 AV: 1 RF: 6.00, 3 NL: 1.23E4
F: {0,0} + c EI Full ms [40.00-800.00]

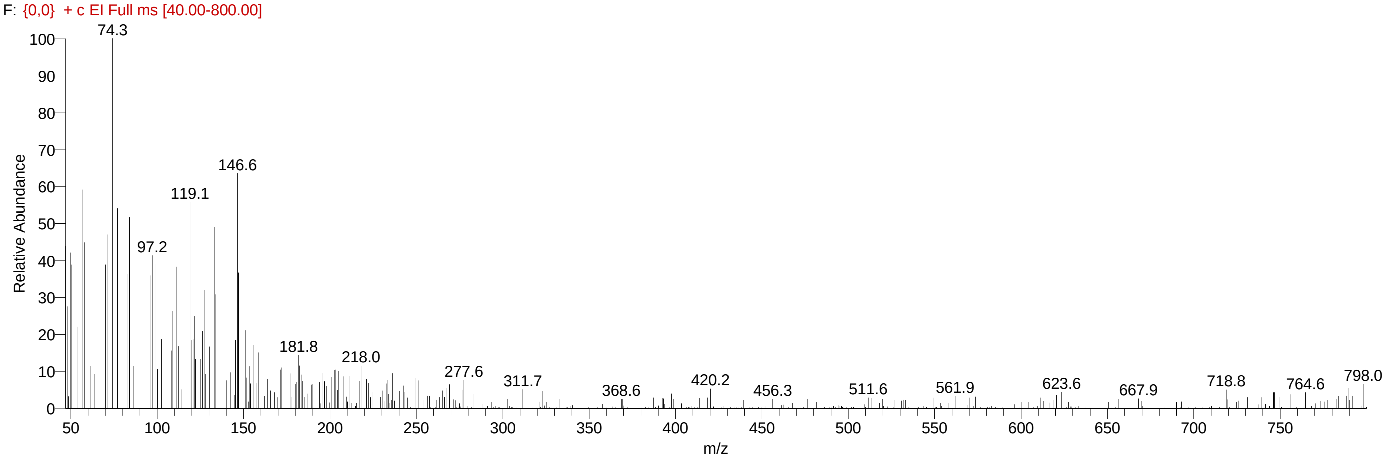

Hit Spectrum | Delta | Compound Structure

1,4,7-Tris(3,5-di-tert-butyl-2-deuteriohydroxybenzyl)-1,4,7-triazacyclononane
Formula C51H72D9N3O3, MW 783, CAS# NA, Entry# 658265

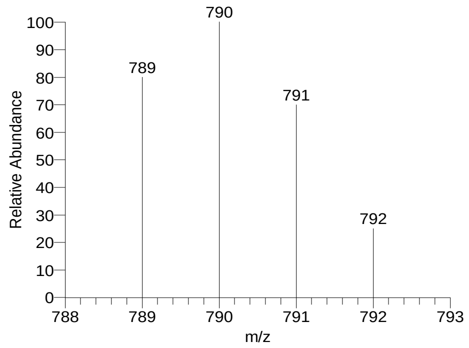

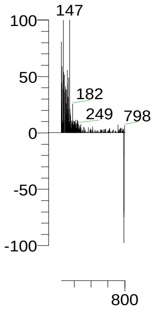

Octaphenylfluorenone
Formula C61H40O, MW 788, CAS# NA, Entry# 658423

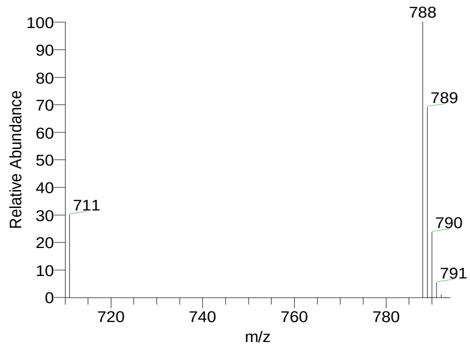

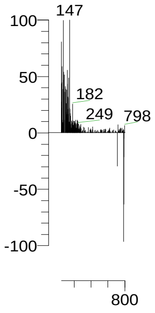

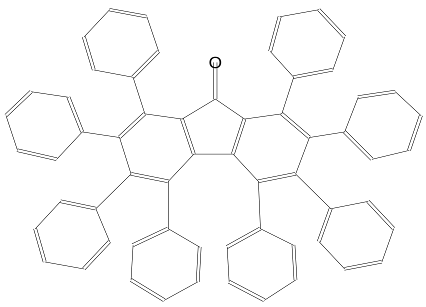

There is no signature data to report.

Library Search Report

Hit Spectrum | Delta | Compound Structure

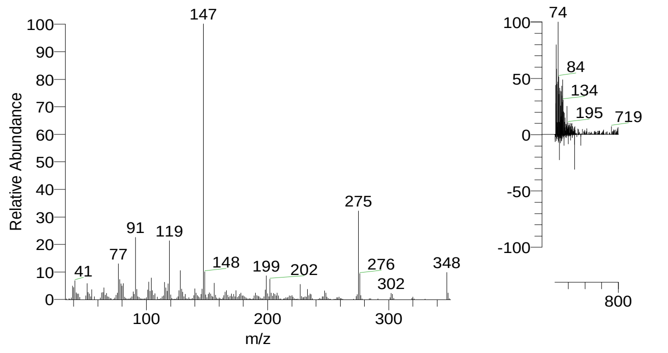

Naphthalene-2-carboxylic acid, 2,3,4,4a,5,6,7,8-octahydro-1,2-dicyano-3-oxo-4-phenyl-, ethyl ester
Formula C21H20N2O3, MW 348, CAS# NA, Entry# 120197
Ethyl 1,2-dicyano-3-oxo-4-phenyl-2,3,4,4a,5,6,7,8-octahydro-2-naphthalenecarboxylate #

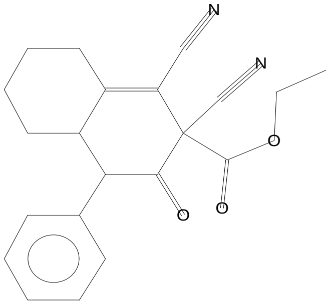

There is no signature data to report.

Library Search Report

RT	Probability	Compound Name	SI	Area %	Area	Molecular Weight	Molecular Formula	Library
36.67	10.92	à-D-Glucopyranoside, methyl 2-(acetylamino)-2-deoxy-3-O-(trimethylsilyl)-, cyclic methylboronate (CAS)	453	0.20	644617.83	331	C13H26BNO6Si	Wiley9
36.67	10.92	à-D-Glucopyranoside, methyl 2-(acetylamino)-2-deoxy-3-O-(trimethylsilyl)-, cyclic methylboronate	450	0.20	644617.83	331	C13H26BNO6Si	mainlib
36.67	6.29	9,12,15-Octadecatrienoic acid, 2-[(trimethylsilyl)oxy]-1-[[(trimethylsilyl)oxy]methyl]ethyl ester, (Z,Z,Z)- (CAS)	437	0.20	644617.83	496	C27H52O4Si2	Wiley9

Faten-212 #9314 RT: 36.67 AV: 1 RF: 6.00, 3 NL: 2.91E4
F: {0,0} + c EI Full ms [40.00-800.00]

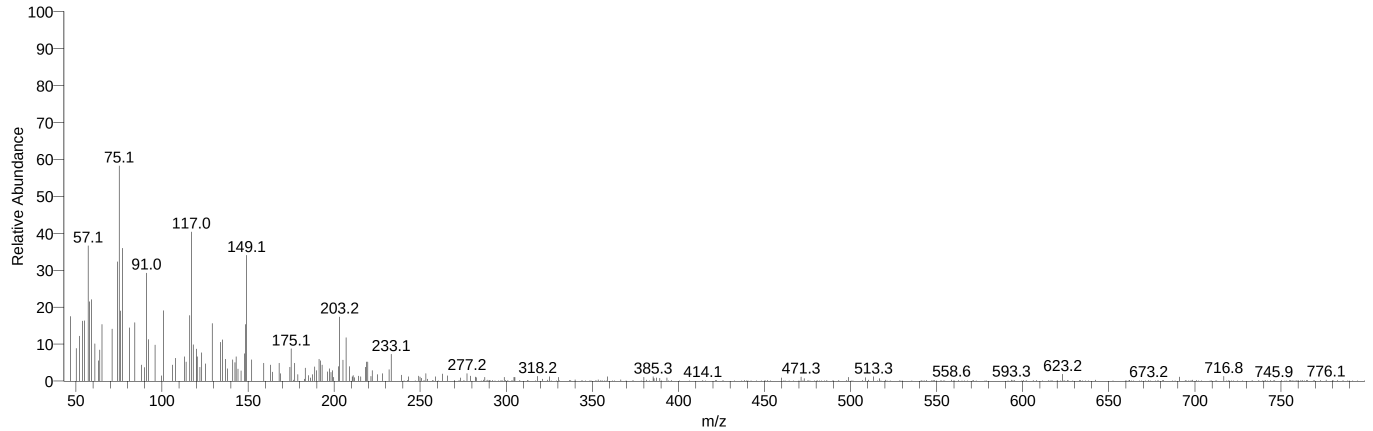

Hit Spectrum | Delta | Compound Structure

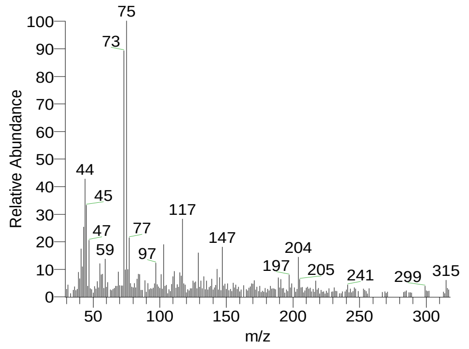

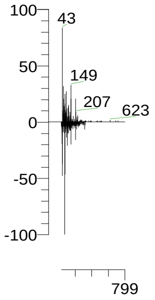

Formula C13H26BNO6Si, MW 331, CAS# 54477-01-9, Entry# 437410
2-ACETAMIDO-A-GLUCOPYRANOSIDE-1-METHYL-4,6-METHYLBORONATE-3-TMS

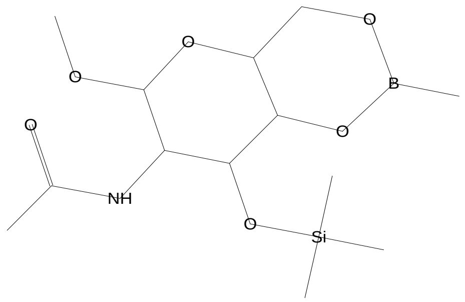

There is no signature data to report.

Library Search Report

Hit Spectrum	Delta	Compound Structure

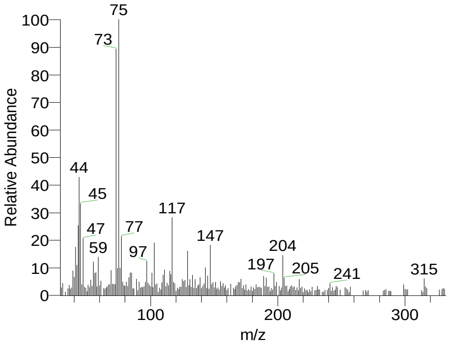

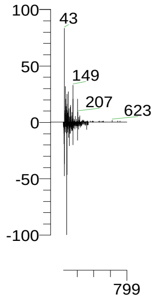

à-D-Glucopyranoside, methyl 2-(acetylamino)-2-deoxy-3-O-(trimethylsilyl)-, cyclic methylboronate
Formula C13H26BNO6Si, MW 331, CAS# 54477-01-9, Entry# 41200

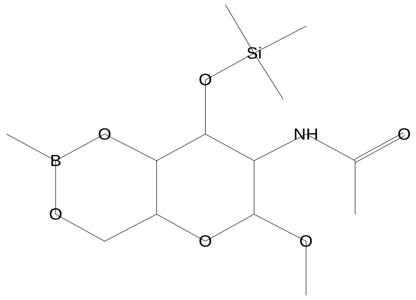

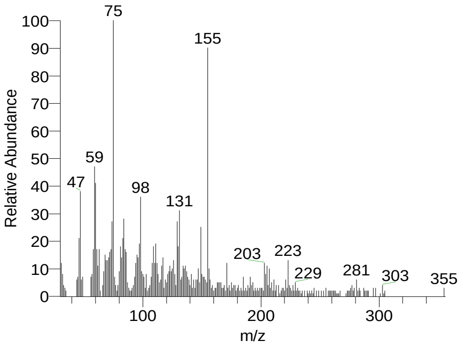

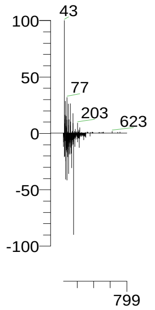

Formula C27H52O4Si2, MW 496, CAS# 55521-23-8, Entry# 616117
TRIMETHYLSILYLETHER DERIVATIVE OF 2-MONOLINOLENIN

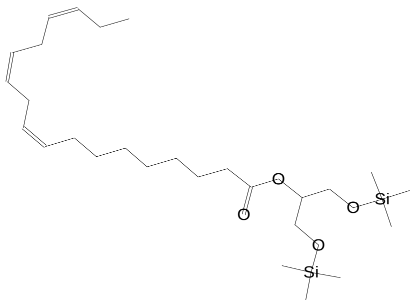

There is no signature data to report.

Library Search Report

RT	Probability	Compound Name	SI	Area %	Area	Molecular Weight	Molecular Formula	Library
36.77	16.23	{ 5-[2',3'-Dimethoxyphenyl]-10,15,20-triphenylporphyrinato}-zinc (II)	402	0.16	522606.05	736	C46H32N4O2Zn	Wiley9
36.77	14.34	{ 5-[3',4'-Dimethoxyphenyl]-10,15,20-triphenylporphyrinato}-zinc (II)	399	0.16	522606.05	736	C46H32N4O2Zn	Wiley9
36.77	9.26	Tetrakis(4-bromo-2,6-dimethylphenyl)ethylene	386	0.16	522606.05	756	C34H32Br4	Wiley9

Faten-212 #9343 RT: 36.77 AV: 1 RF: 6.00, 3 NL: 4.98E4
F: {0,0} + c EI Full ms [40.00-800.00]

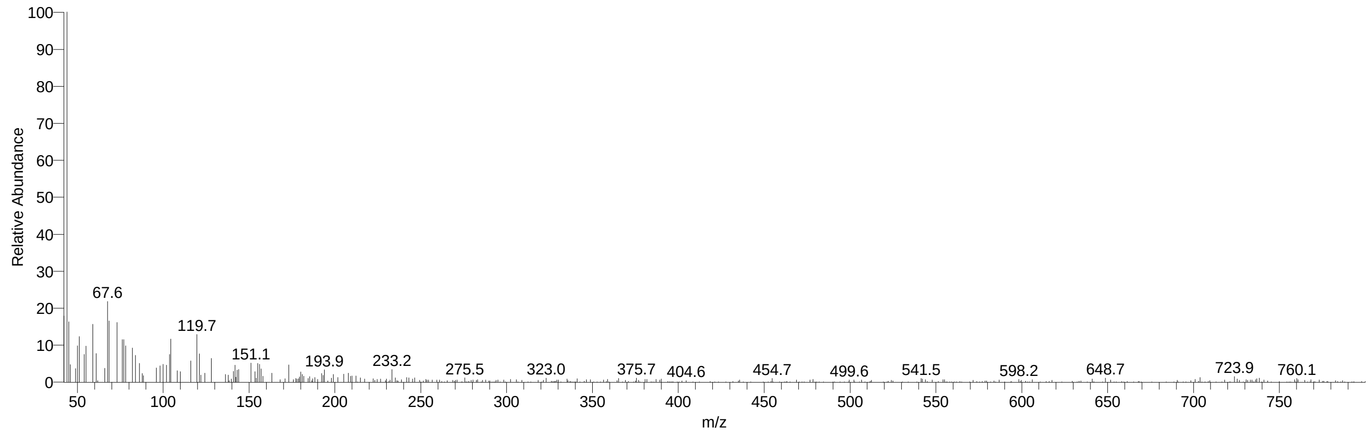

Hit Spectrum	Delta	Compound Structure

{ 5-[2',3'-Dimethoxyphenyl]-10,15,20-triphenylporphyrinato}-zinc (II)
Formula C46H32N4O2Zn, MW 736, CAS# NA, Entry# 656593

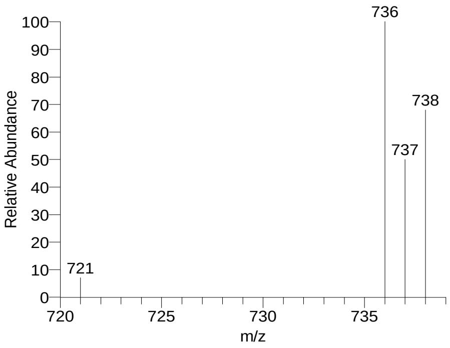

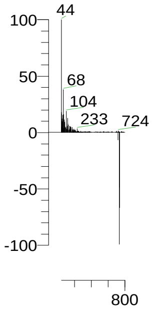

{ 5-[3',4'-Dimethoxyphenyl]-10,15,20-triphenylporphyrinato}-zinc (II)
Formula C46H32N4O2Zn, MW 736, CAS# NA, Entry# 656594

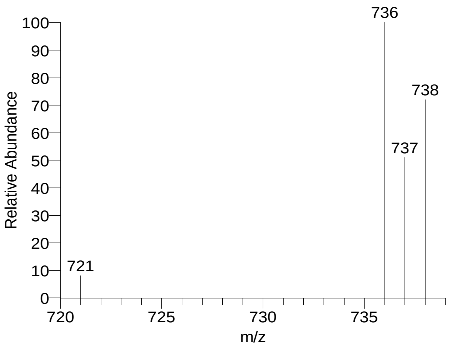

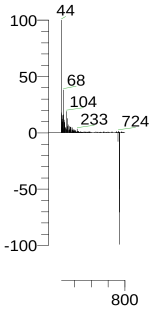

There is no signature data to report.

Library Search Report

Hit Spectrum	Delta	Compound Structure

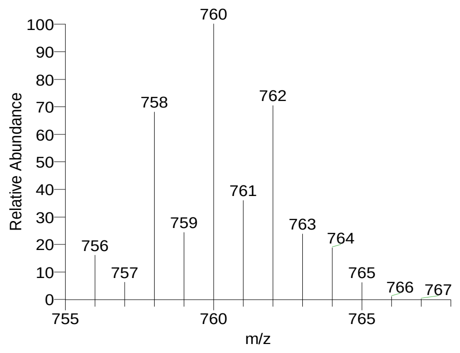

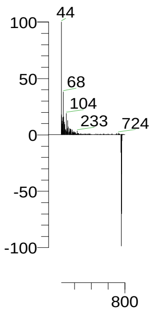

Tetrakis(4-bromo-2,6-dimethylphenyl)ethylene
Formula C34H32Br4, MW 756, CAS# NA, Entry# 657330

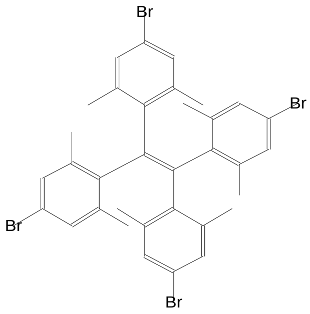

There is no signature data to report.

Library Search Report

RT	Probability	Compound Name	SI	Area %	Area	Molecular Weight	Molecular Formula	Library
37.15	16.19	Tertbutyloxyformamide, N-methyl-N-[4-(1-pyrrolidinyl)-2-butynyl]-	442	0.13	405047.80	252	C14H24N2O2	mainlib
37.15	11.75	2-Acetyltetrazole	433	0.13	405047.80	112	C3H4N4O	Wiley9
37.15	8.29	Cyclopropanetetradecanoic acid, 2-octyl-, methyl ester	423	0.13	405047.80	394	C26H50O2	mainlib

Faten-212 #9455 RT: 37.15 AV: 1 RF: 6.00, 3 NL: 8.87E4
F: {0,0} + c EI Full ms [40.00-800.00]

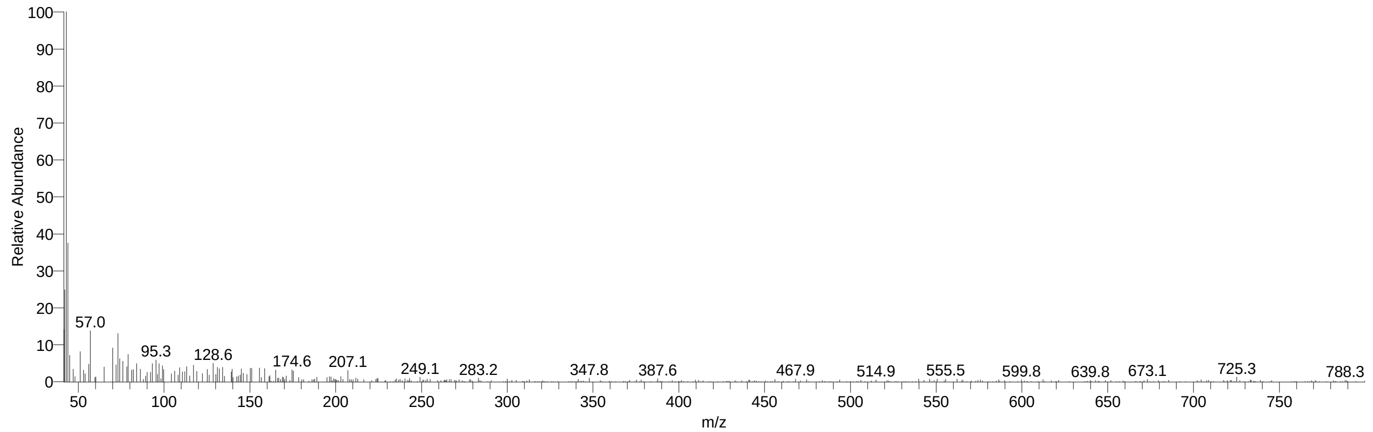

Hit Spectrum	Delta	Compound Structure

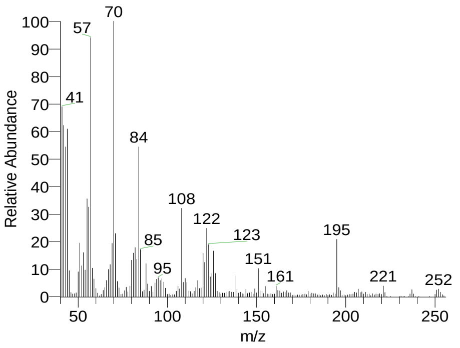

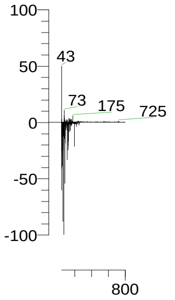

Tertbutyloxyformamide, N-methyl-N-[4-(1-pyrrolidinyl)-2-butynyl]-
Formula C14H24N2O2, MW 252, CAS# 124045-68-7, Entry# 34145
t-Butoxyformamid, N-methyl-N-[4-(1-pyrrolidinyl)-2-butynyl]-

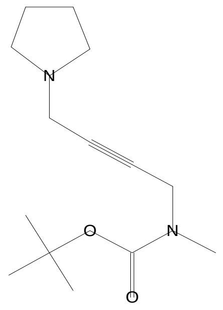

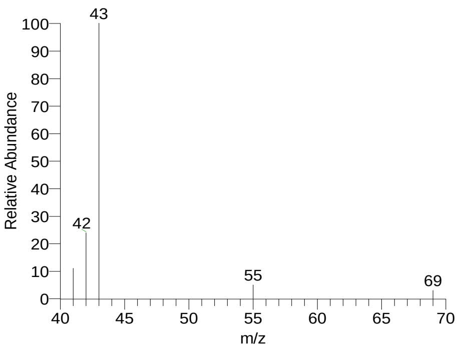

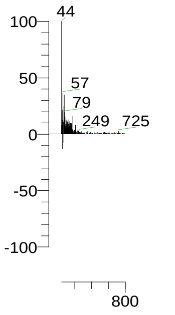

2-Acetyltetrazole
Formula C3H4N4O, MW 112, CAS# 51410-11-8, Entry# 12962
2H-Tetrazole, 2-acetyl- (CAS)

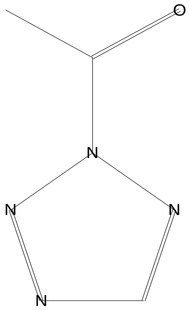

There is no signature data to report.

Library Search Report

Hit Spectrum	Delta	Compound Structure

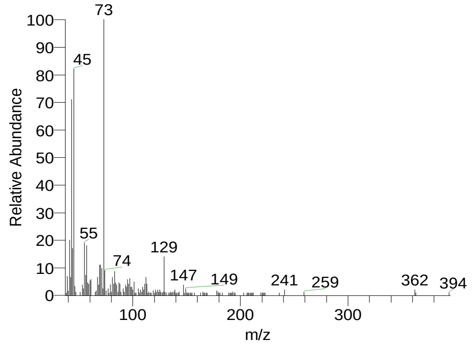

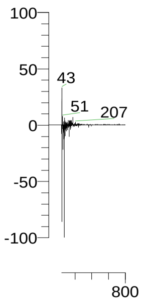

Cyclopropanetetradecanoic acid, 2-octyl-, methyl ester
Formula C26H50O2, MW 394, CAS# 52355-42-7, Entry# 37108
Methyl 14-(2-octylcyclopropyl)tetradecanoate #

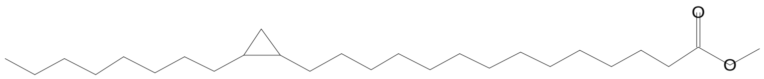

There is no signature data to report.

Library Search Report

RT	Probability	Compound Name	SI	Area %	Area	Molecular Weight	Molecular Formula	Library
37.34	8.36	1H-Cyclopropa[3,4]benz[1,2-e]azulene-5,7b,9,9a-tetrol, 1a,1b,4,4a,5,7a,8,9-octahydro-3-(hydroxymethyl)-1,1,6,8-tetramethyl-, 5,9,9a-triacetate, [1aR-(1aà,1bá,4aá,5á,7aà,7bà,8à,9á,9aà)]-	378	0.20	634692.82	476	C26H36O8	mainlib
37.34	4.31	3-Oxatricyclo[20.8.0.0(7,16)]triaconta-1(22),7(16),9,13,23,29-hexaene	360	0.20	634692.82	406	C29H42O	mainlib
37.34	4.31	3-Oxatricyclo[20.8.0.0(7,16)]triaconta-1(22),7(16),9,13,23,29-hexaene	360	0.20	634692.82	406	C29H42O	Wiley9

Faten-212 #9509 RT: 37.34 AV: 1 RF: 6.00, 3 NL: 9.57E3
F: {0,0} + c EI Full ms [40.00-800.00]

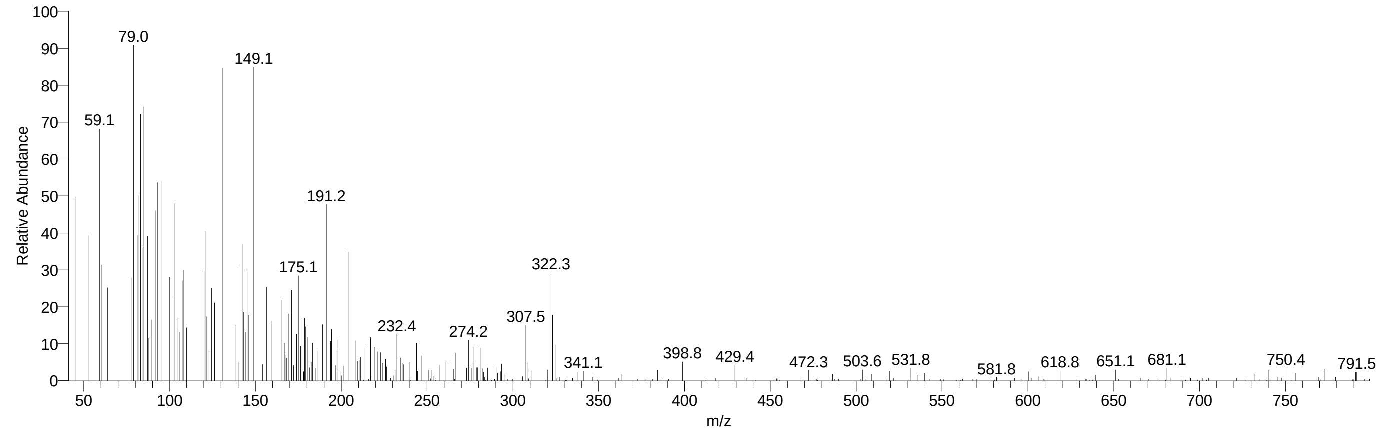

Hit Spectrum	Delta	Compound Structure

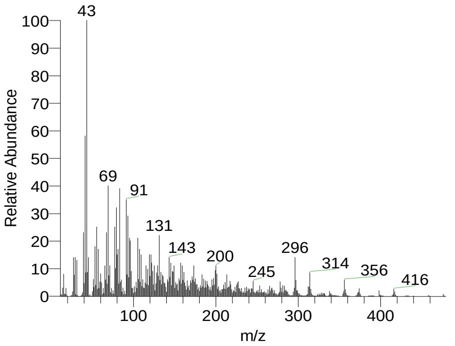

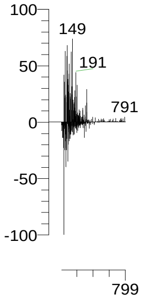

Formula C26H36O8, MW 476, CAS# 77508-64-6, Entry# 5847

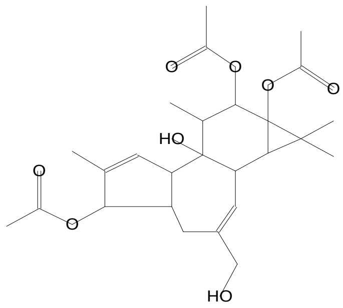

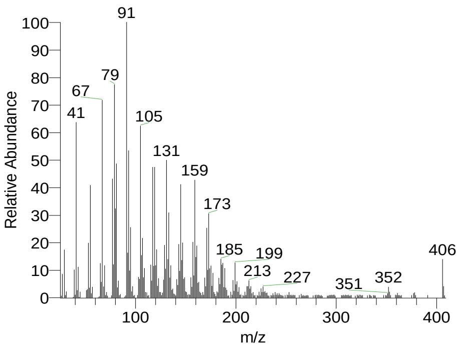

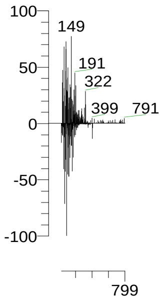

3-Oxatricyclo[20.8.0.0(7,16)]triaconta-1(22),7(16),9,13,23,29-hexaene
Formula C29H42O, MW 406, CAS# NA, Entry# 54773

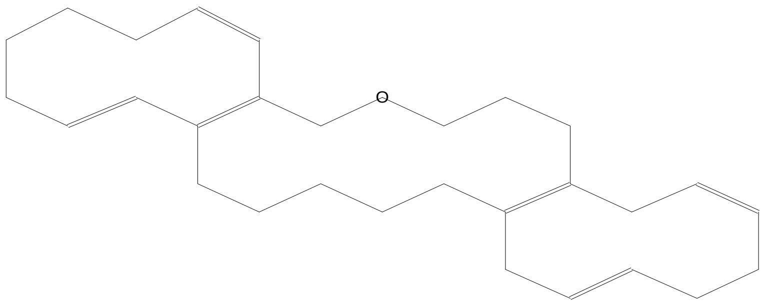

There is no signature data to report.

Library Search Report

Hit Spectrum	Delta	Compound Structure

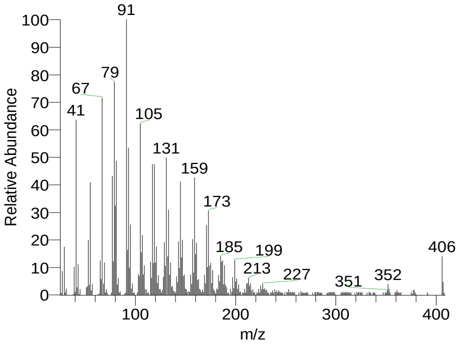

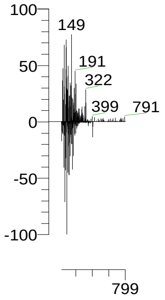

3-Oxatricyclo[20.8.0.0(7,16)]triaconta-1(22),7(16),9,13,23,29-hexaene
Formula C29H42O, MW 406, CAS# NA, Entry# 549989
3-OXATRICYCLO[20.8.0.0E7,16]TRICONTA-1(22),7(16),9,13,23,29-HEXAEN

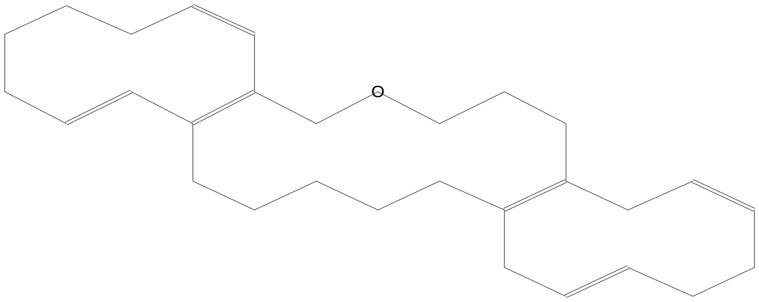

There is no signature data to report.

Library Search Report

RT	Probability	Compound Name	SI	Area %	Area	Molecular Weight	Molecular Formula	Library
37.51	7.99	L(-)-CYSTINE	379	0.21	652926.52	240	C6H12N2O4S2	Wiley9
37.51	4.84	Acetamide, N-methyl-N-[4-(3-hydroxypyrrolidinyl)-2-butynyl]-	364	0.21	652926.52	210	C11H18N2O2	mainlib
37.51	4.65	Pregan-20-one, 2-hydroxy-5,6-epoxy-15-methyl-	363	0.21	652926.52	346	C22H34O3	mainlib

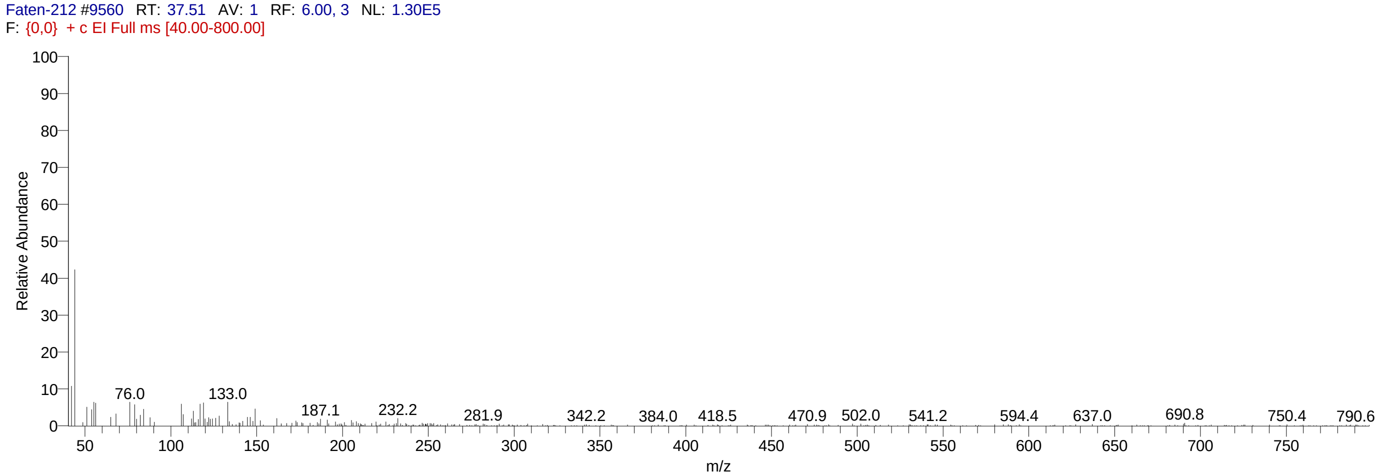

Hit Spectrum	Delta	Compound Structure

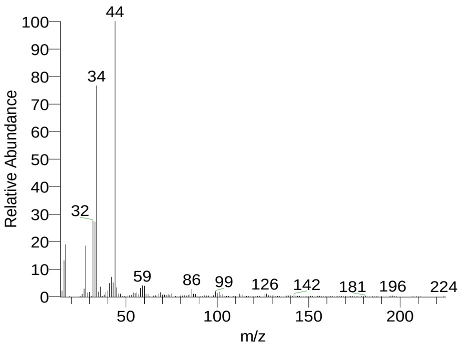

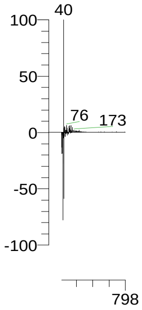

L(-)-CYSTINE
Formula C6H12N2O4S2, MW 240, CAS# 923-32-0, Entry# 234660
DL-Cystine

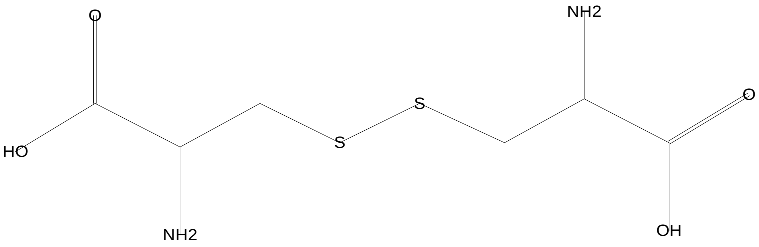

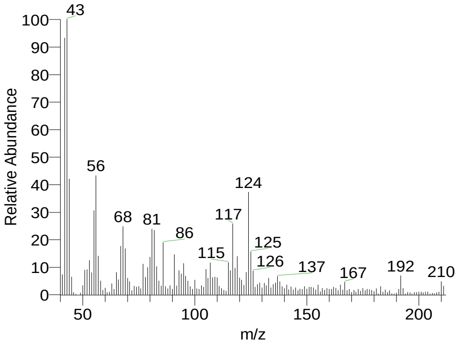

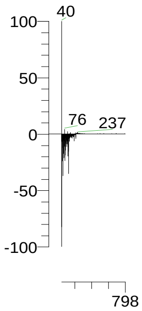

Acetamide, N-methyl-N-[4-(3-hydroxypyrrolidinyl)-2-butynyl]-
Formula C11H18N2O2, MW 210, CAS# 130403-61-1, Entry# 6216
N-[4-(3-Hydroxy-1-pyrrolidinyl)-2-butynyl]-N-methylacetamide #

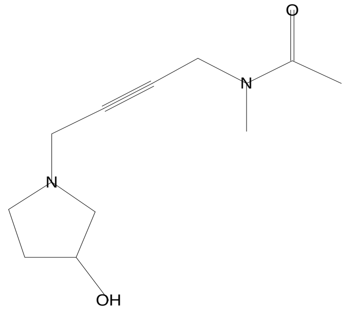

There is no signature data to report.

Library Search Report

Hit Spectrum | Delta | Compound Structure

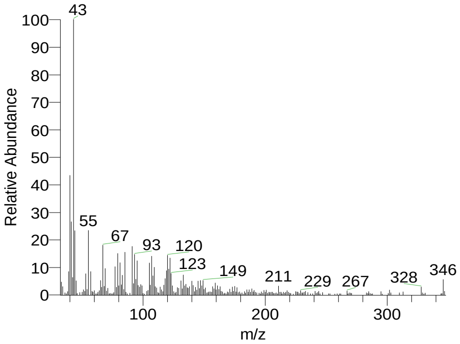

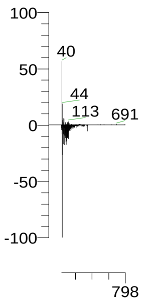

Pregan-20-one, 2-hydroxy-5,6-epoxy-15-methyl-
Formula C22H34O3, MW 346, CAS# NA, Entry# 5378

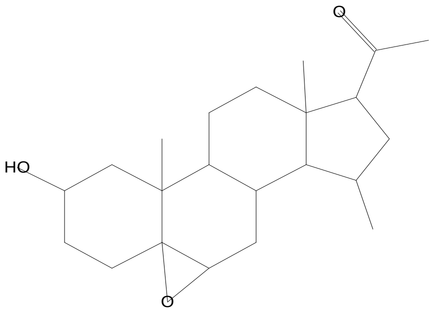

There is no signature data to report.

Library Search Report

RT	Probability	Compound Name	SI	Area %	Area	Molecular Weight	Molecular Formula	Library
37.75	17.63	Pentadecylbenzene	464	0.20	639754.56	288	C21H36	Wiley9
37.75	5.28	Ethyl iso-allocholate	438	0.20	639754.56	436	C26H44O5	mainlib
37.75	5.28	Ethyl iso-allocholate	438	0.20	639754.56	436	C26H44O5	Wiley9

Faten-212 #9630 RT: 37.75 AV: 1 RF: 6.00, 3 NL: 6.52E4
F: {0,0} + c EI Full ms [40.00-800.00]

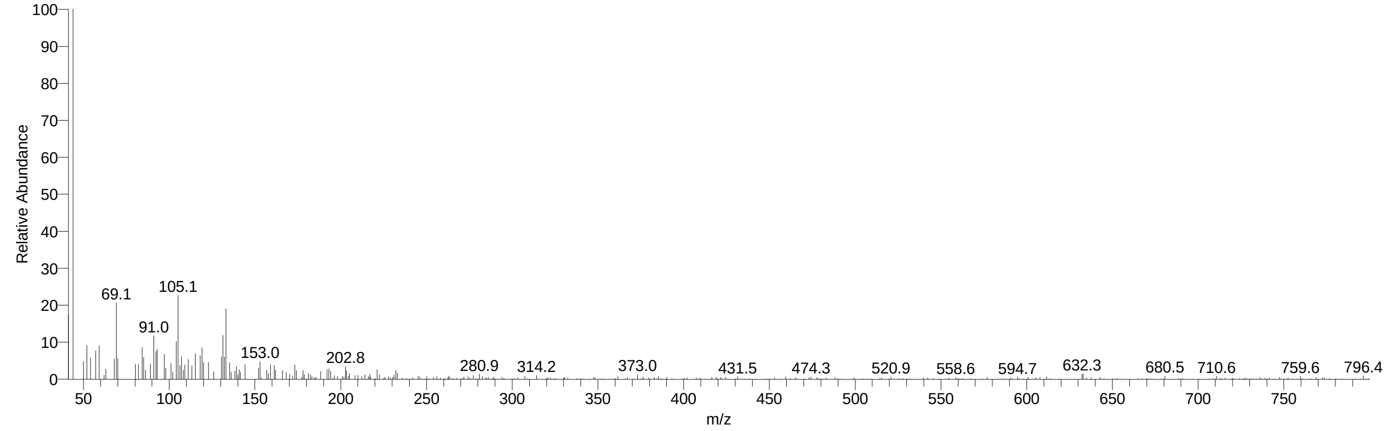

Hit Spectrum	Delta	Compound Structure

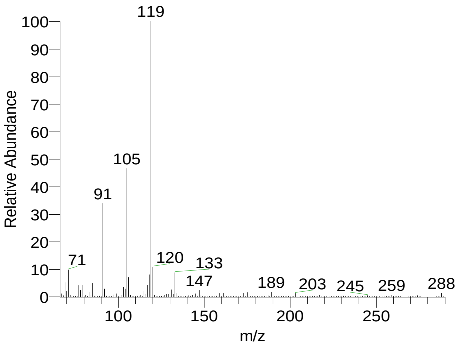

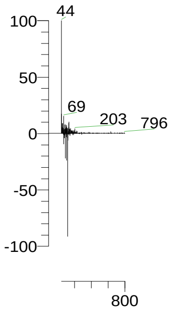

Pentadecylbenzene
Formula C21H36, MW 288, CAS# 2131-18-2, Entry# 349051
Benzene, pentadecyl- (CAS)

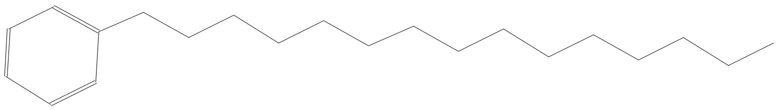

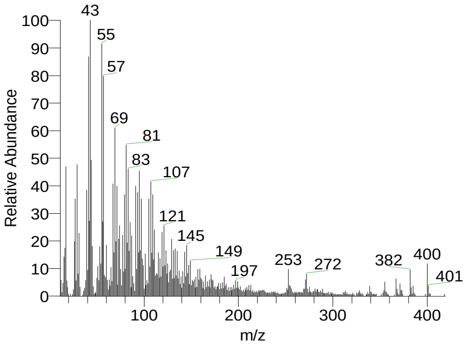

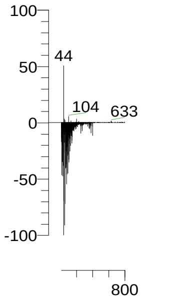

Ethyl iso-allocholate
Formula C26H44O5, MW 436, CAS# NA, Entry# 6654
Ethyl 3,7,12-trihydroxycholan-24-oate #

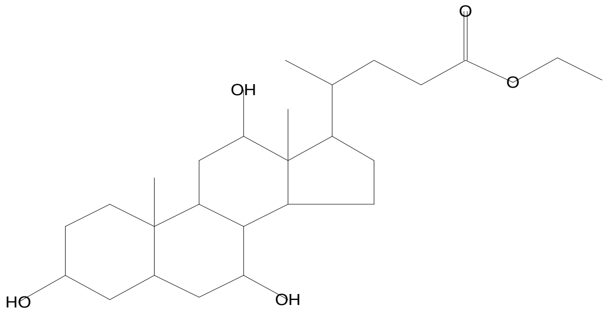

There is no signature data to report.

Library Search Report

Hit Spectrum	Delta	Compound Structure

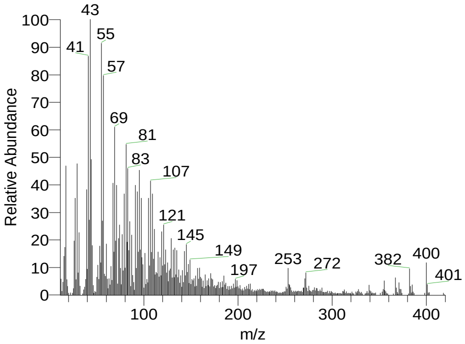

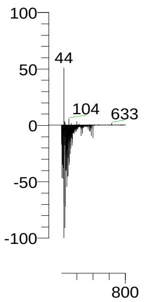

Ethyl iso-allocholate
Formula C26H44O5, MW 436, CAS# NA, Entry# 578772
Ethyl 3,7,12-trihydroxycholan-24-oate

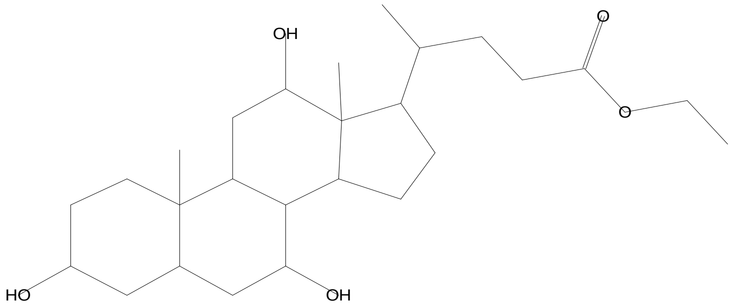

There is no signature data to report.

Library Search Report

RT	Probability	Compound Name	SI	Area %	Area	Molecular Weight	Molecular Formula	Library
38.23	26.59	Benzene, (1-pentylheptyl)- (CAS)	569	0.53	1686674.58	246	C18H30	Wiley9
38.23	16.11	Benzene, (1-hexyltetradecyl)- (CAS)	554	0.53	1686674.58	358	C26H46	Wiley9
38.23	3.80	Pentacosane, 13-phenyl-	515	0.53	1686674.58	428	C31H56	mainlib

Faten-212 #9770 RT: 38.23 AV: 1 RF: 6.00, 3 NL: 1.52E5
F: {0,0} + c EI Full ms [40.00-800.00]

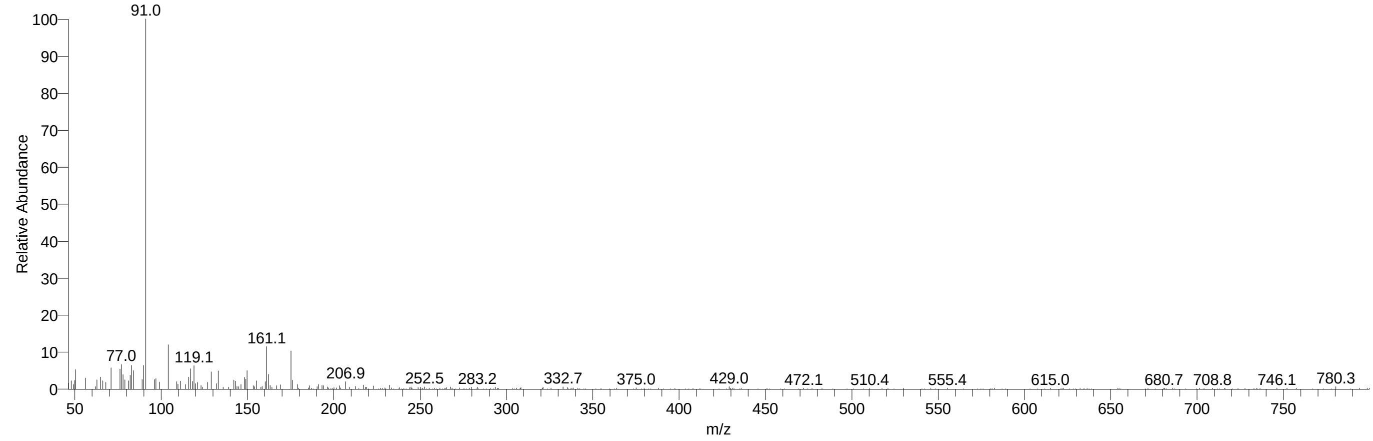

Hit Spectrum	Delta	Compound Structure

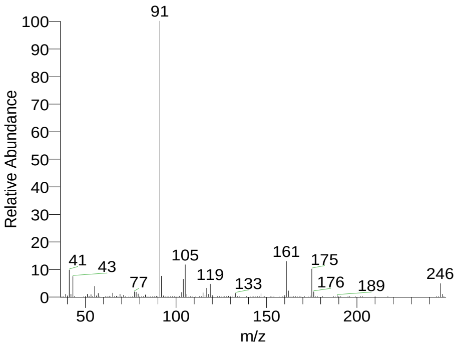

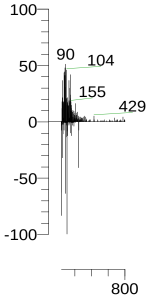

Benzene, (1-pentylheptyl)- (CAS)
Formula C18H30, MW 246, CAS# 2719-62-2, Entry# 251019
6-Phenyldodecane

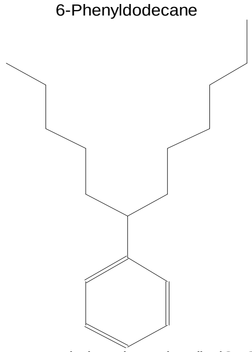

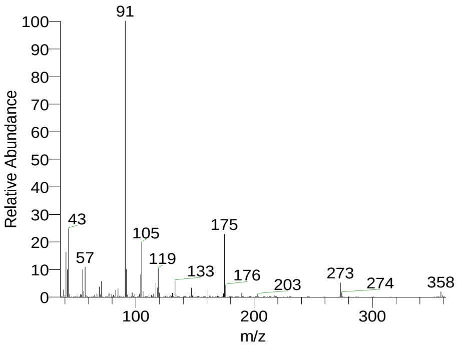

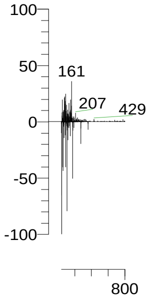

Benzene, (1-hexyltetradecyl)- (CAS)
Formula C26H46, MW 358, CAS# 2398-64-3, Entry# 485824
Eicosane, 7-phenyl-

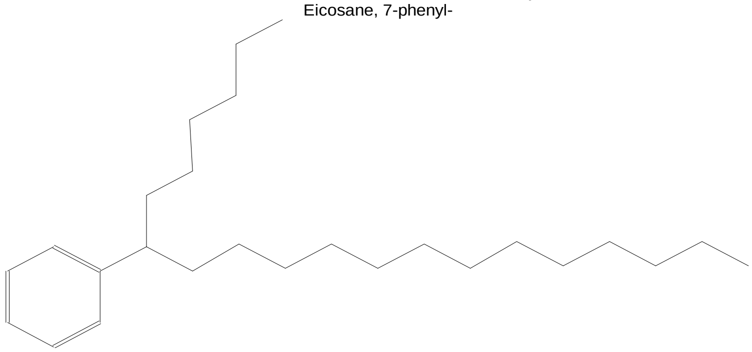

There is no signature data to report.

Library Search Report

Hit Spectrum	Delta	Compound Structure

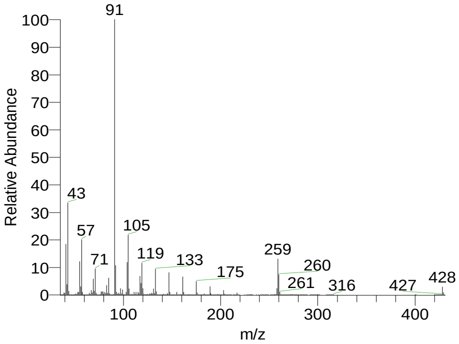

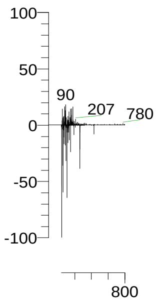

Pentacosane, 13-phenyl-
Formula C31H56, MW 428, CAS# 6006-90-2, Entry# 53730
Benzene, (1-dodecyltridecyl)-

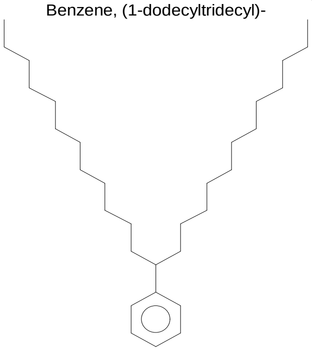

There is no signature data to report.

Library Search Report

RT	Probability	Compound Name	SI	Area %	Area	Molecular Weight	Molecular Formula	Library
38.39	8.61	3-(2-benzo[b]thienyl)propane-1,1,2,2-tetracarbonitrile	440	0.75	2388254.24	276	C15H8N4S	Wiley9
38.39	8.61	2-(2,4,6-Trimethyl-phenyl)-[1,3]dioxolane-4,5-dicarboxylic acid, dimethyl ester	440	0.75	2388254.24	308	C16H20O6	mainlib
38.39	8.61	(4R,5R)-2-Mesityl-1,3-dioxolane-4,5-dicarbonsaure-dimethylester	440	0.75	2388254.24	308	C16H20O6	Wiley9

Faten-212 #9817 RT: 38.39 AV: 1 RF: 6.00, 3 NL: 9.72E4
F: {0,0} + c EI Full ms [40.00-800.00]

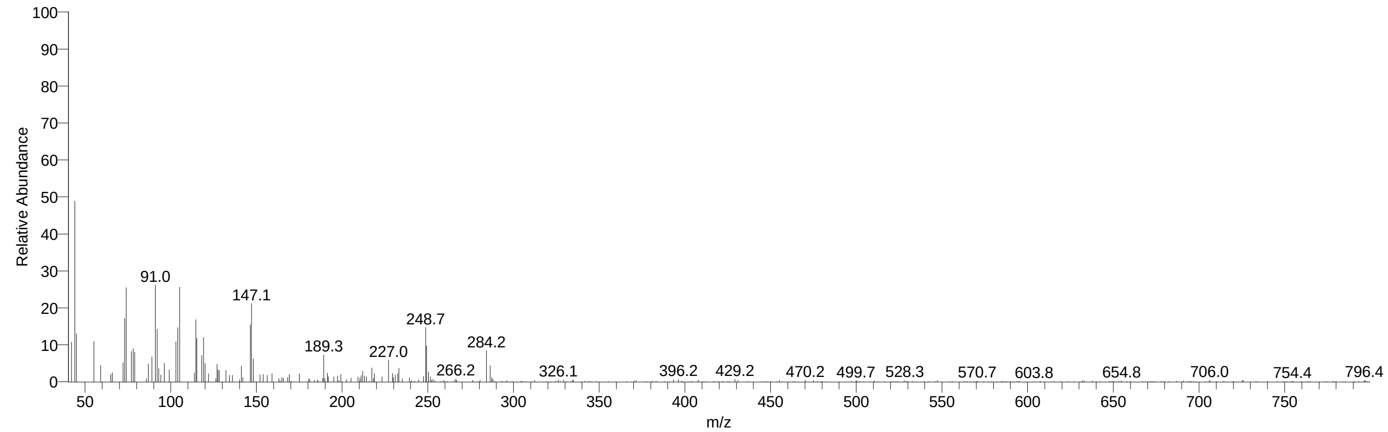

Hit Spectrum | Delta | Compound Structure

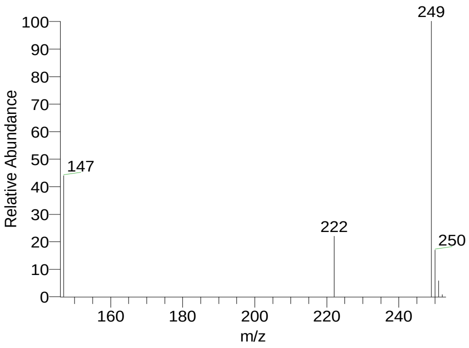

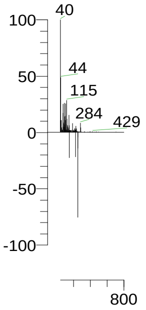

3-(2-benzo[b]thienyl)propane-1,1,2,2-tetracarbonitrile
Formula C15H8N4S, MW 276, CAS# 119522-00-8, Entry# 319873
1,1,2,2-Propanetetracarbonitrile, 3-benzo[b]thien-2-yl- (CAS)

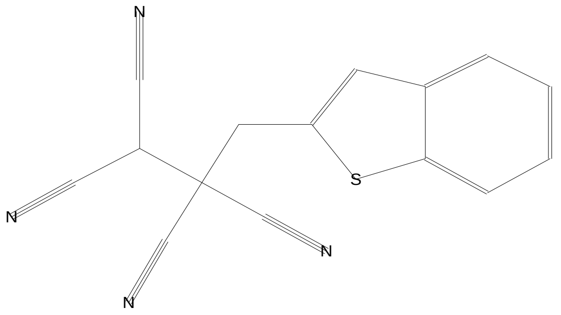

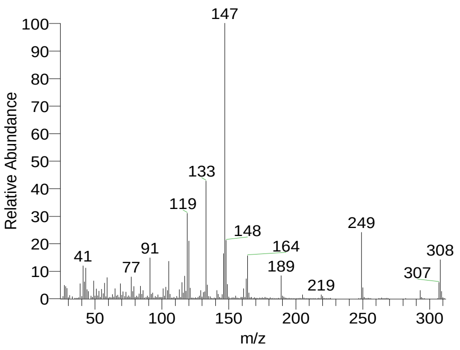

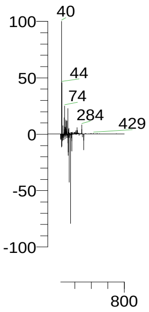

2-(2,4,6-Trimethyl-phenyl)-[1,3]dioxolane-4,5-dicarboxylic acid, dimethyl ester
Formula C16H20O6, MW 308, CAS# 114026-67-4, Entry# 119835
Dimethyl 2-mesityl-1,3-dioxolane-4,5-dicarboxylate #

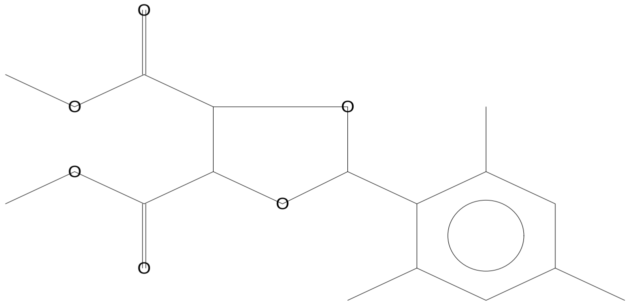

There is no signature data to report.

Library Search Report

Hit Spectrum	Delta	Compound Structure

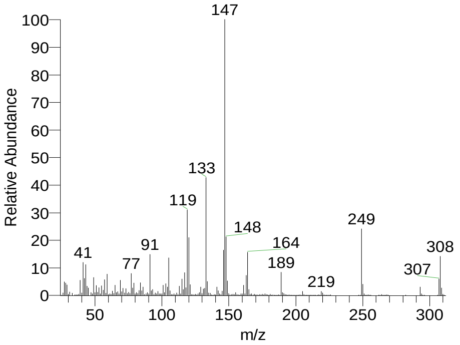

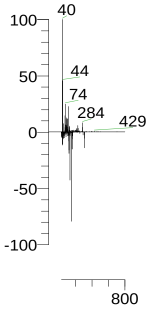

(4R,5R)-2-Mesityl-1,3-dioxolane-4,5-dicarbonsaure-dimethylester
Formula C16H20O6, MW 308, CAS# 114026-67-4, Entry# 390948
2-(2,4,6-Trimethyl-phenyl)-[1,3]dioxolane-4,5-dicarboxylic acid, dimethyl ester

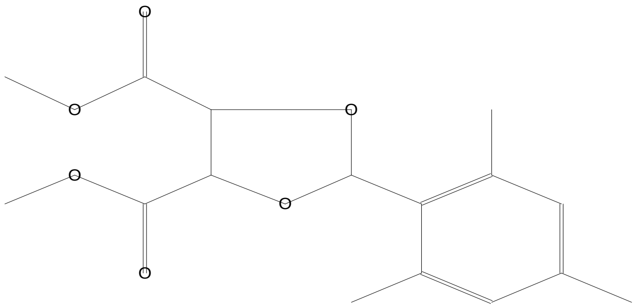

There is no signature data to report.

Library Search Report

RT	Probability	Compound Name	SI	Area %	Area	Molecular Weight	Molecular Formula	Library
38.88	11.43	Benzene, (1-propylnonyl)- (CAS)	459	0.72	2278081.58	246	C18H30	Wiley9
38.88	8.53	Pentadecylbenzene	451	0.72	2278081.58	288	C21H36	Wiley9
38.88	6.87	Benzene, (1-propylheptadecyl)- (CAS)	446	0.72	2278081.58	358	C26H46	Wiley9

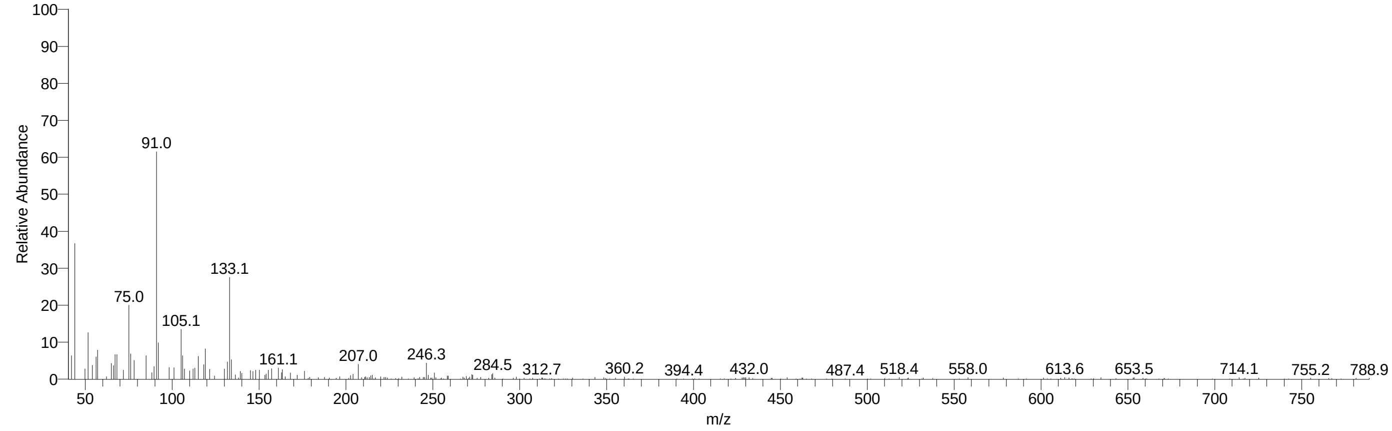

Hit Spectrum	Delta	Compound Structure

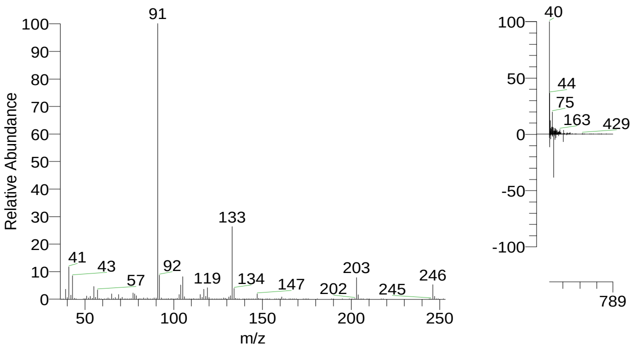

Benzene, (1-propylnonyl)- (CAS)
Formula C18H30, MW 246, CAS# 2719-64-4, Entry# 251013
4-Phenyldodecane

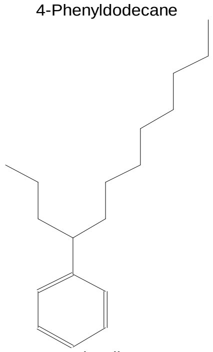

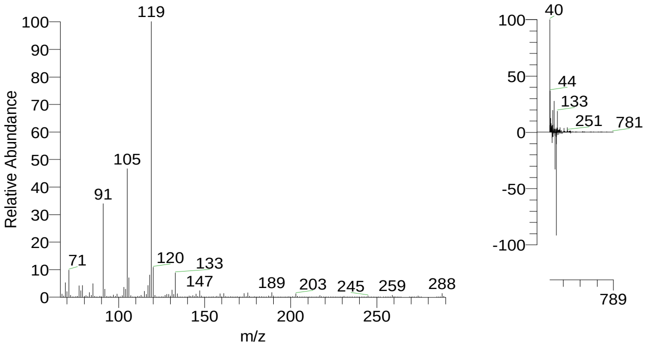

Pentadecylbenzene
Formula C21H36, MW 288, CAS# 2131-18-2, Entry# 349051
Benzene, pentadecyl- (CAS)

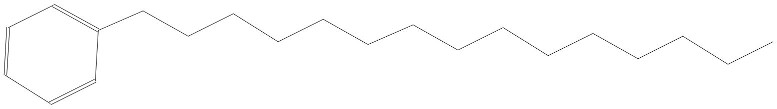

There is no signature data to report.

Library Search Report

Hit Spectrum	Delta	Compound Structure

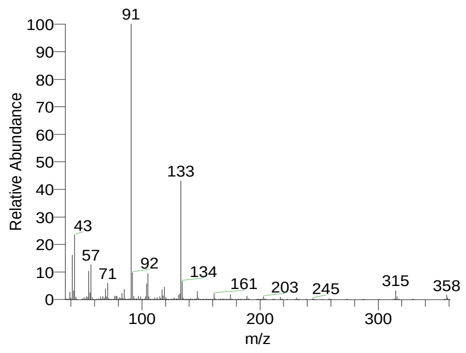

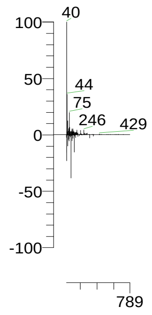

Benzene, (1-propylheptadecyl)- (CAS)
Formula C26H46, MW 358, CAS# 2400-03-5, Entry# 485834
Eicosane, 4-phenyl-

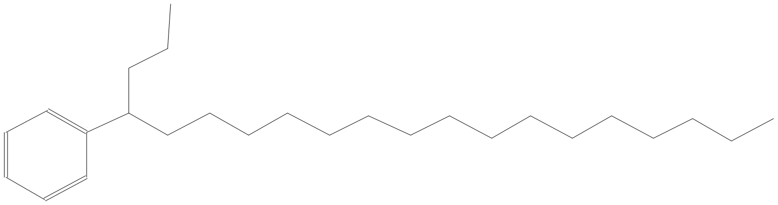

There is no signature data to report.

Library Search Report

RT	Probability	Compound Name	SI	Area %	Area	Molecular Weight	Molecular Formula	Library
39.12	9.21	à-D-Galactopyranoside, methyl 2,3-bis-O-(trimethylsilyl)-, cyclic methylboronate	490	0.31	984420.76	362	C14H31BO6Si2	mainlib
39.12	9.21	à-D-Galactopyranoside, methyl 2,3-bis-O-(trimethylsilyl)-, cyclic methylboronate (CAS)	490	0.31	984420.76	362	C14H31BO6Si2	Wiley9
39.12	8.50	á-D-Galactopyranoside, methyl 2,3-bis-O-(trimethylsilyl)-, cyclic butylboronate	488	0.31	984420.76	404	C17H37BO6Si2	mainlib

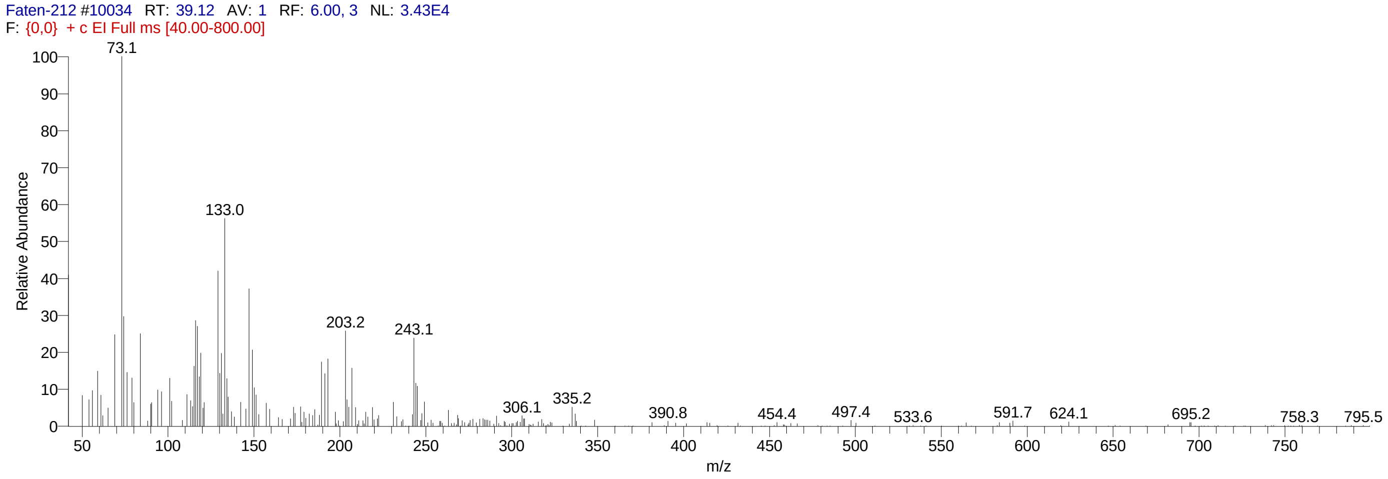

Hit Spectrum	Delta	Compound Structure

à-D-Galactopyranoside, methyl 2,3-bis-O-(trimethylsilyl)-, cyclic methylboronate
Formula C14H31BO6Si2, MW 362, CAS# 54400-88-3, Entry# 38593

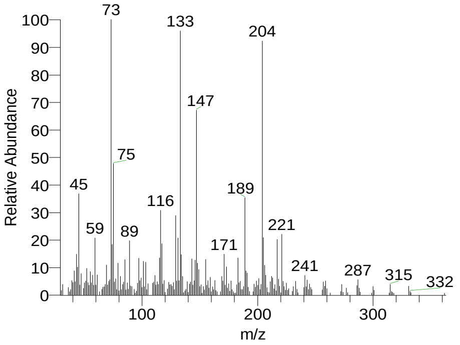

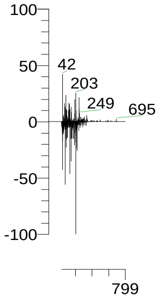

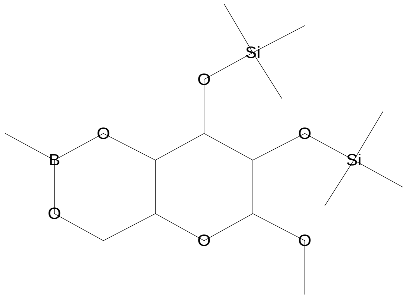

à-D-Galactopyranoside, methyl 2,3-bis-O-(trimethylsilyl)-, cyclic methylboronate (CAS)
Formula C14H31BO6Si2, MW 362, CAS# 54400-88-3, Entry# 490297
A-GALACTOPYRANOSIDE-1-METHYL-4,6-METHYLBORONATE-2,3-DITMS

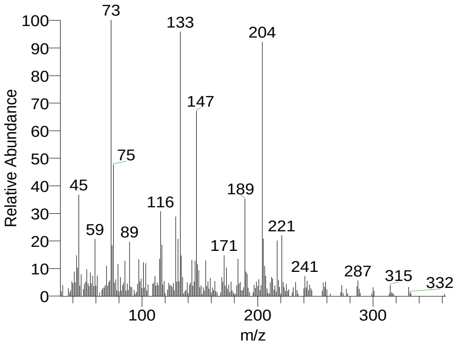

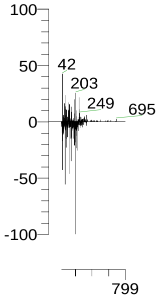

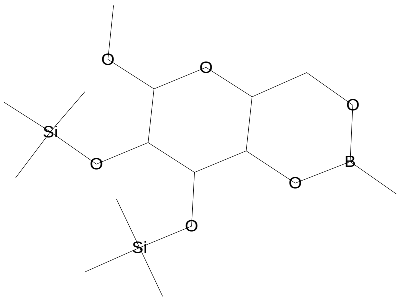

There is no signature data to report.

Library Search Report

Hit Spectrum	Delta	Compound Structure

á-D-Galactopyranoside, methyl 2,3-bis-O-(trimethylsilyl)-, cyclic butylboronate
Formula C17H37BO6Si2, MW 404, CAS# 56211-10-0, Entry# 38594

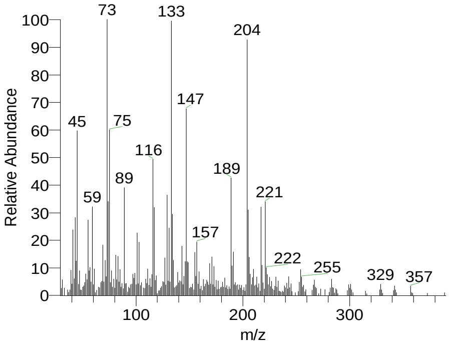

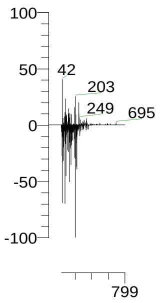

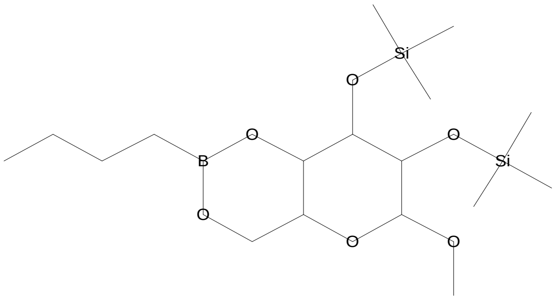

There is no signature data to report.

Library Search Report

RT	Probability	Compound Name	SI	Area %	Area	Molecular Weight	Molecular Formula	Library
39.53	15.01	Silanamine, N-[2-[3-methoxy-4-[(trimethylsilyl)oxy]phenyl]-N,1,1,1-tetramethyl-2-[(trimethylsilyl)oxy]ethyl]- (CAS)	443	0.15	469862.96	413	C19H39NO3Si3	Wiley9
39.53	9.99	1-(4'-TRIMETHYSILYLOXYPHENYL)-1-TRIMETHYLSILYLOXY-2-METHYLTRIMETHYLSILYL-AMINOETHANE	431	0.15	469862.96	383	C18H37NO2Si3	Wiley9
39.53	5.45	1-methyl-4-(1-methylpropenyl)benzene	414	0.15	469862.96	146	C11H14	Wiley9

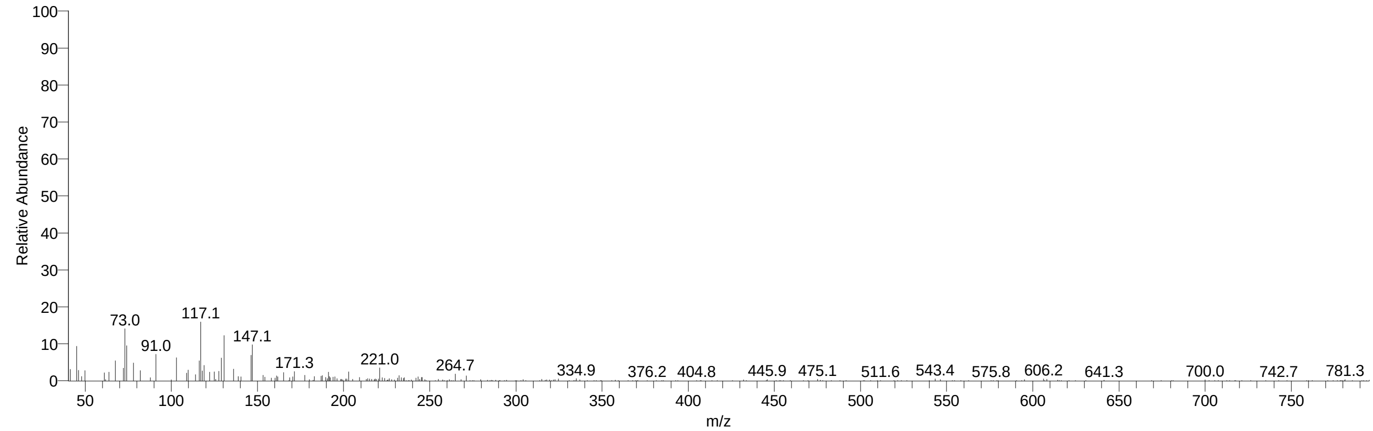

Hit Spectrum	Delta	Compound Structure

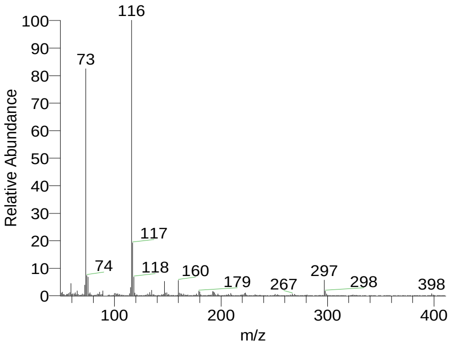

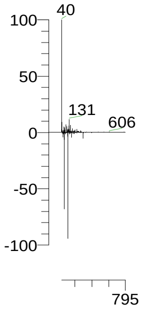

Formula C19H39NO3Si3, MW 413, CAS# 56114-63-7, Entry# 556550
METANEPHRINE-TRITMS

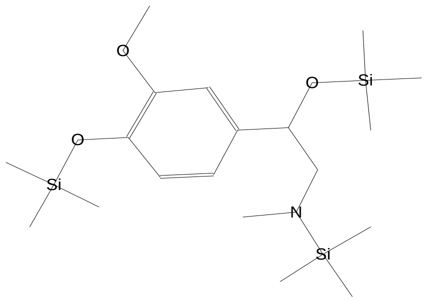

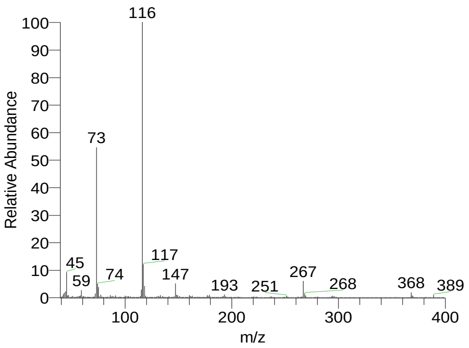

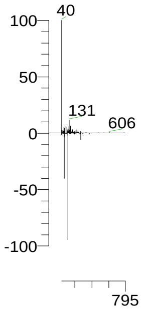

Formula C18H37NO2Si3, MW 383, CAS# NA, Entry# 520945

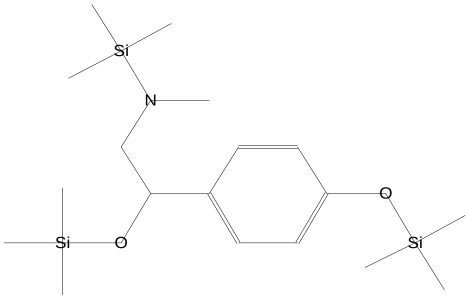

There is no signature data to report.

Library Search Report

Hit Spectrum | Delta | Compound Structure

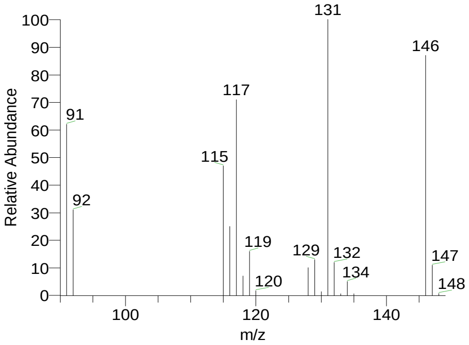

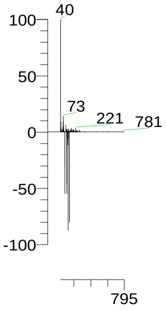

1-methyl-4-(1-methylpropenyl)benzene
Formula C11H14, MW 146, CAS# NA, Entry# 46417

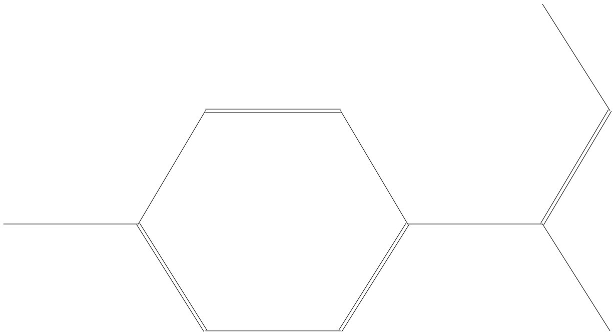

There is no signature data to report.

Library Search Report

RT	Probability	Compound Name	SI	Area %	Area	Molecular Weight	Molecular Formula	Library
39.74	65.15	2,13-Dithia(3)metacyclo(3)naphthalenophane	940	7.74	24573453.27	322	C20H18S2	Wiley9
39.74	9.07	5-Chloro-3-geranylorcylaldehyde	868	7.74	24573453.27	322	C18H23ClO3	Wiley9
39.74	6.77	4-N-(4'-Acetylphenyl)amino-6,7-dimethoxyquinazoline	860	7.74	24573453.27	323	C18H17N3O3	Wiley9

Faten-212 #10215 RT: 39.74 AV: 1 RF: 6.00, 3 NL: 4.86E5
F: {0,0} + c EI Full ms [40.00-800.00]

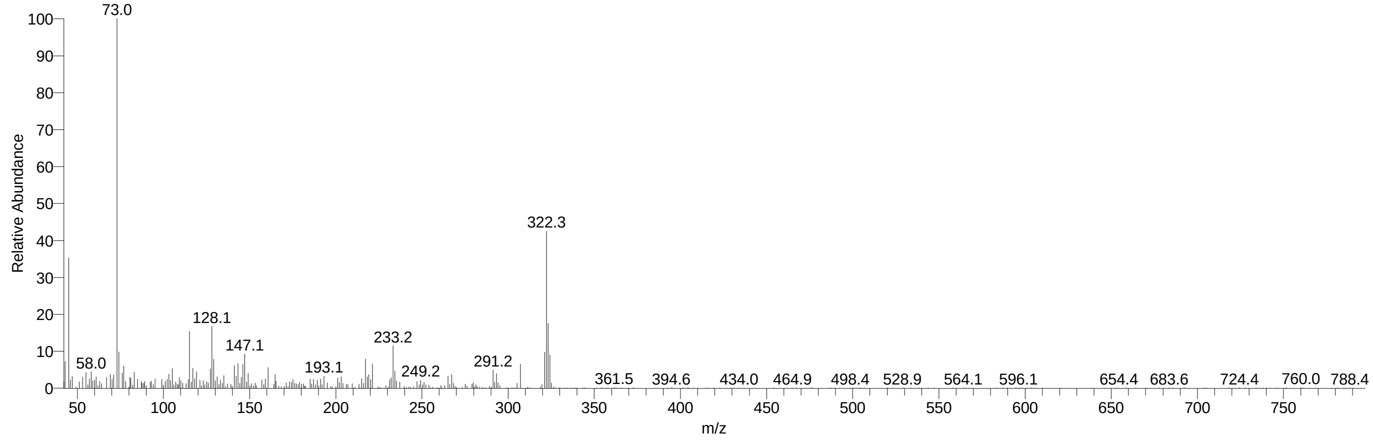

Hit Spectrum	Delta	Compound Structure

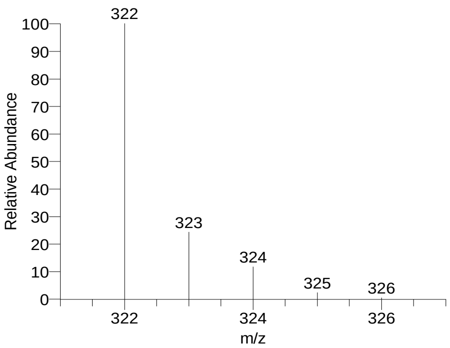

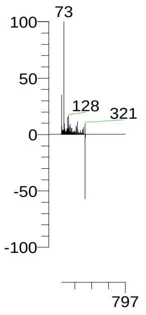

2,13-Dithia(3)metacyclo(3)naphthalenophane
Formula C20H18S2, MW 322, CAS# 72150-49-3, Entry# 421048
10H,12H-1,13:5,9-Dimetheno-2H,4H-3,11-benzodithiacyclohexadecin, stereoisomer (CAS)

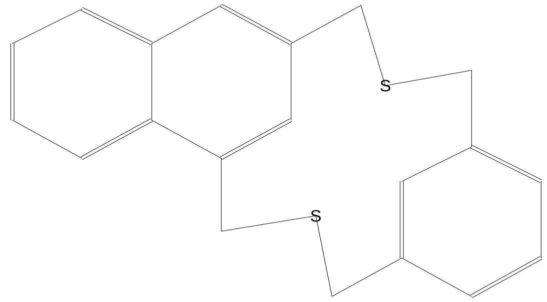

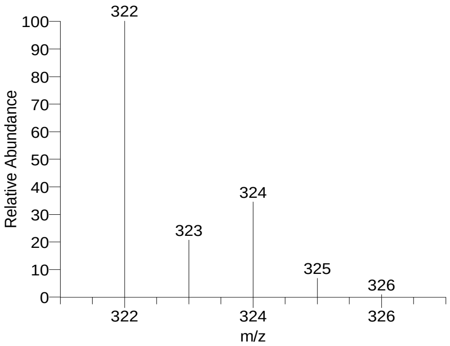

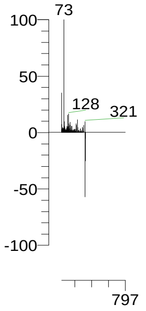

5-Chloro-3-geranylorcylaldehyde
Formula C18H23ClO3, MW 322, CAS# 83324-48-5, Entry# 420439
Colletochlorin B

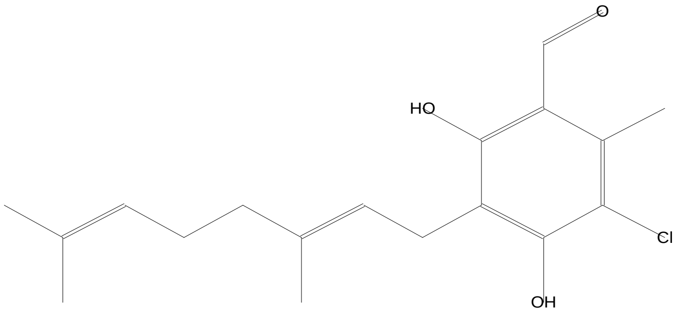

There is no signature data to report.

Library Search Report

Hit Spectrum	Delta	Compound Structure

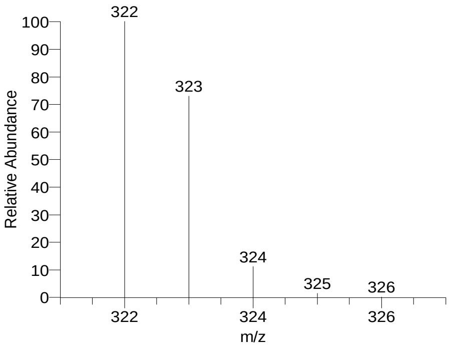

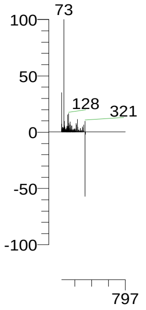

4-N-(4'-Acetylphenyl)amino-6,7-dimethoxyquinazoline
Formula C18H17N3O3, MW 323, CAS# NA, Entry# 422436

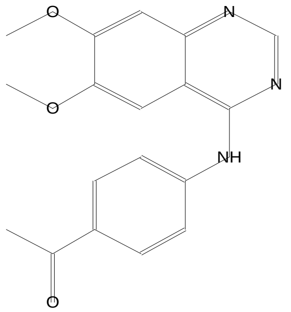

There is no signature data to report.

Library Search Report

RT	Probability	Compound Name	SI	Area %	Area	Molecular Weight	Molecular Formula	Library
40.24	11.03	1H-Indene, 1-hexadecyl-2,3-dihydro- (CAS)	433	0.28	896748.08	342	C25H42	Wiley9
40.24	5.69	à-D-Glucofuranose, 6-O-(trimethylsilyl)-, cyclic 1,2:3,5-bis(butylboronate)	415	0.28	896748.08	384	C17H34B2O6Si	mainlib
40.24	5.69	à-D-Glucofuranose, 6-O-(trimethylsilyl)-, cyclic 1,2:3,5-bis(butylboronate) (CAS)	415	0.28	896748.08	384	C17H34B2O6Si	Wiley9

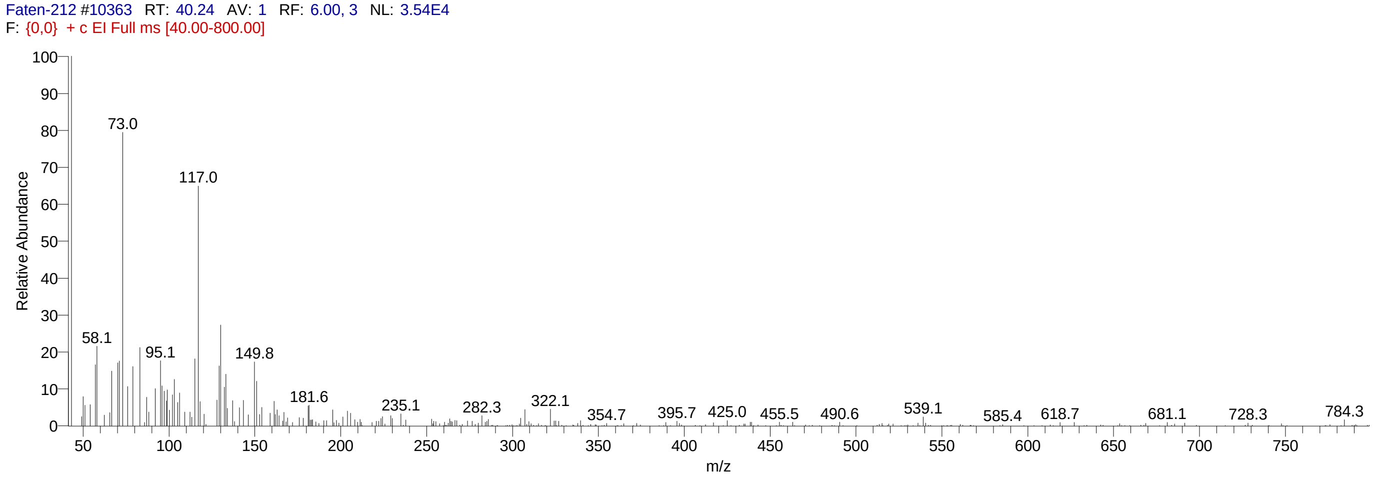

Hit Spectrum	Delta	Compound Structure

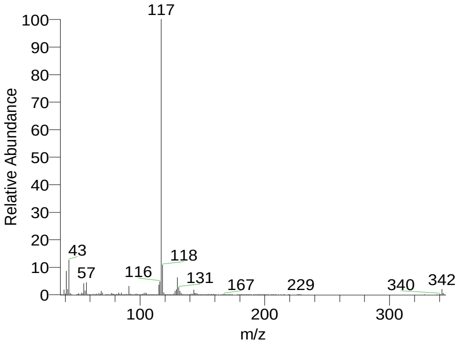

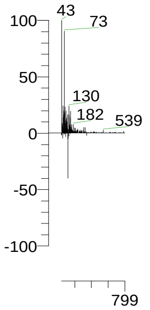

1H-Indene, 1-hexadecyl-2,3-dihydro- (CAS)
Formula C25H42, MW 342, CAS# 55334-29-7, Entry# 459014
1-N-HEXADECYLINDANE

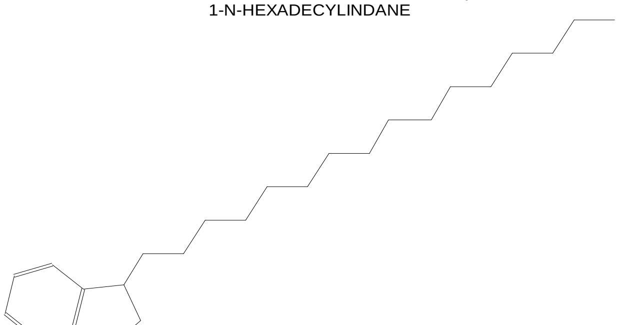

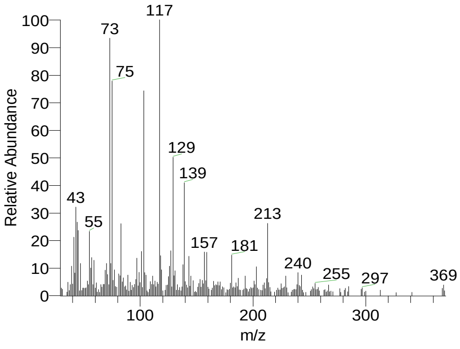

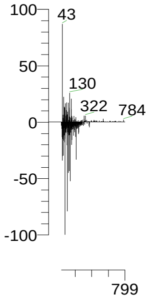

à-D-Glucofuranose, 6-O-(trimethylsilyl)-, cyclic 1,2:3,5-bis(butylboronate)
Formula C17H34B2O6Si, MW 384, CAS# 72347-48-9, Entry# 86804

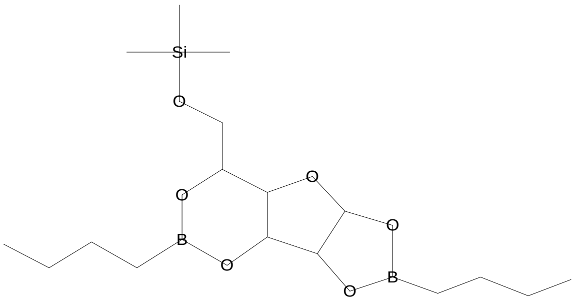

There is no signature data to report.

Library Search Report

Hit Spectrum | Delta | Compound Structure

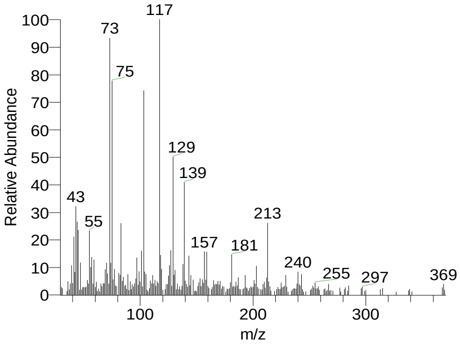

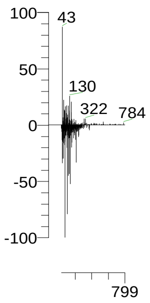

à-D-Glucofuranose, 6-O-(trimethylsilyl)-, cyclic 1,2:3,5-bis(butylboronate) (CAS)
Formula C17H34B2O6Si, MW 384, CAS# 72347-48-9, Entry# 521970
A-GLUCOFURANOSE-1,2-3,5-DI-BUTYLBORONATE-3-TMS

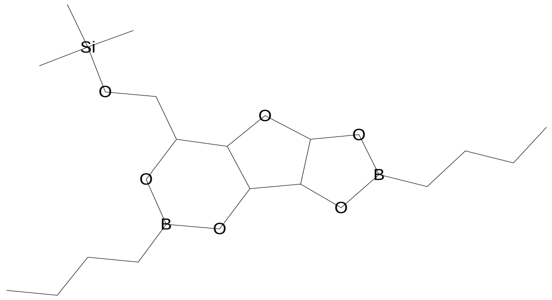

There is no signature data to report.

Library Search Report

RT	Probability	Compound Name	SI	Area %	Area	Molecular Weight	Molecular Formula	Library
40.49	95.87	34,38-Dioxo-33-(2-propenyl-1-oxy)-35,37-dimethoxy-36-(phenylmethoxy)-10,15,20,31-tetramethyl-3,7,23,27-tetraazaheptacyclo(27.3.1.1.1.1.1.1)ocaconta-1(33),8(35),9,11,13-(36),14,16,18(37),19,21,29,31-dodecaene	604	0.16	497551.67	806	C50H54N4O6	Wiley9
40.49	0.95	[5,9-Dimethyl-1-(3-phenyl-oxiran-2-yl)-deca-4,8-dienylidene]-(2-phenyl-aziridin-1-yl)-amine	373	0.16	497551.67	414	C28H34N2O	mainlib
40.49	0.95	[5,9-Dimethyl-1-(3-phenyl-oxiran-2-yl)-deca-4,8-dienylidene]-(2-phenyl-aziridin-1-yl)-amine	373	0.16	497551.67	414	C28H34N2O	Wiley9

Faten-212 #10437 RT: 40.49 AV: 1 RF: 6.00, 3 NL: 1.61E4
F: {0,0} + c EI Full ms [40.00-800.00]

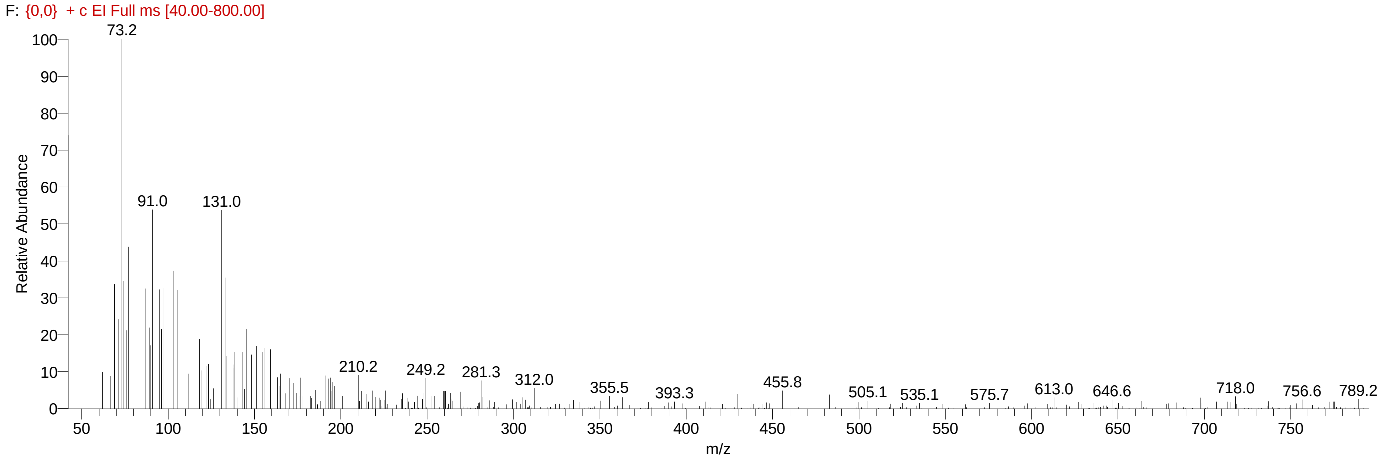

Hit Spectrum	Delta	Compound Structure

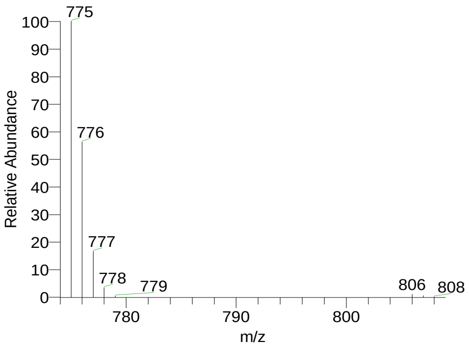

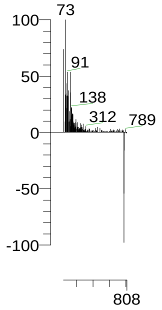

Formula C50H54N4O6, MW 806, CAS# 84379-21-5, Entry# 658875

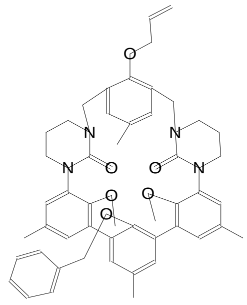

There is no signature data to report.

Library Search Report

Hit Spectrum | Delta | Compound Structure

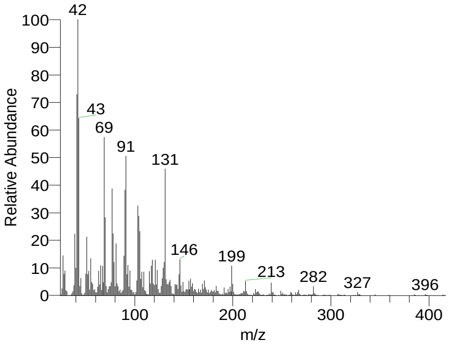

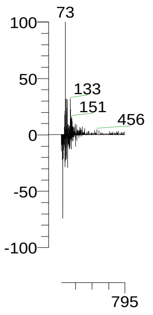

[5,9-Dimethyl-1-(3-phenyl-oxiran-2-yl)-deca-4,8-dienylidene]-(2-phenyl-aziridin-1-yl)-amine
Formula C28H34N2O, MW 414, CAS# NA, Entry# 4208
N-[(4E)-5,9-Dimethyl-1-(3-phenyl-2-oxiranyl)-4,8-decadienylidene]-2-phenyl-1-aziridinamine #

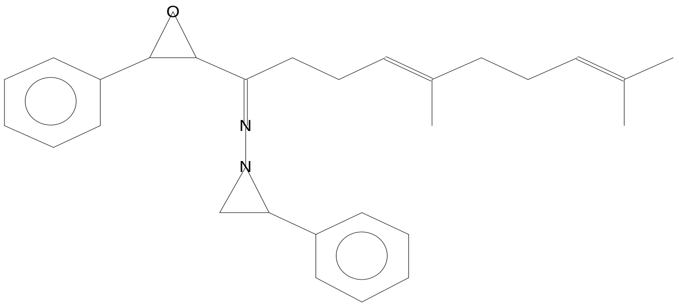

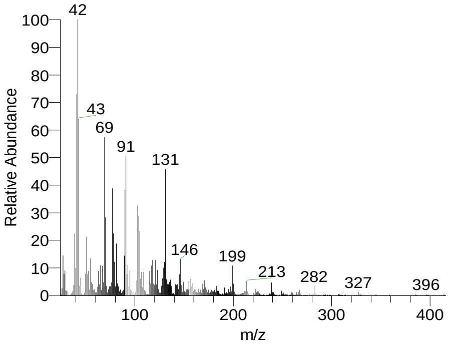

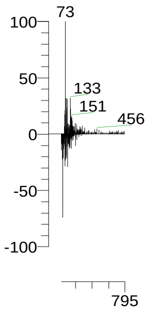

[5,9-Dimethyl-1-(3-phenyl-oxiran-2-yl)-deca-4,8-dienylidene]-(2-phenyl-aziridin-1-yl)-amine
Formula C28H34N2O, MW 414, CAS# NA, Entry# 558433
[5,9-DIMETHYL-1-(3-PHENYL-OXIRAN-2-YL)-DECA-4,8-DIENYLIDENE]-(2-PHENYL-AZIRIDIN-

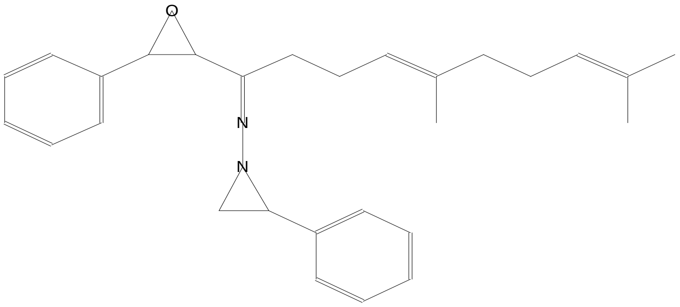

There is no signature data to report.

Library Search Report

RT	Probability	Compound Name	SI	Area %	Area	Molecular Weight	Molecular Formula	Library
40.60	41.22	1,2-Diaminopropane-N,N,N',N'-tetraacetic acid	437	0.15	469134.89	306	C11H18N2O8	mainlib
40.60	8.23	Androst-9(11)-en-17-one, 3-[(trimethylsilyl)oxy]-, O-methyloxime, (3à,5à)- (CAS)	385	0.15	469134.89	389	C23H39NO2Si	Wiley9
40.60	8.23	Androst-9(11)-en-17-one, 3-[(trimethylsilyl)oxy]-, O-methyloxime, (3à,5à)-	384	0.15	469134.89	389	C23H39NO2Si	mainlib

Faten-212 #10469 RT: 40.60 AV: 1 RF: 6.00, 3 NL: 5.86E4
F: {0,0} + c EI Full ms [40.00-800.00]

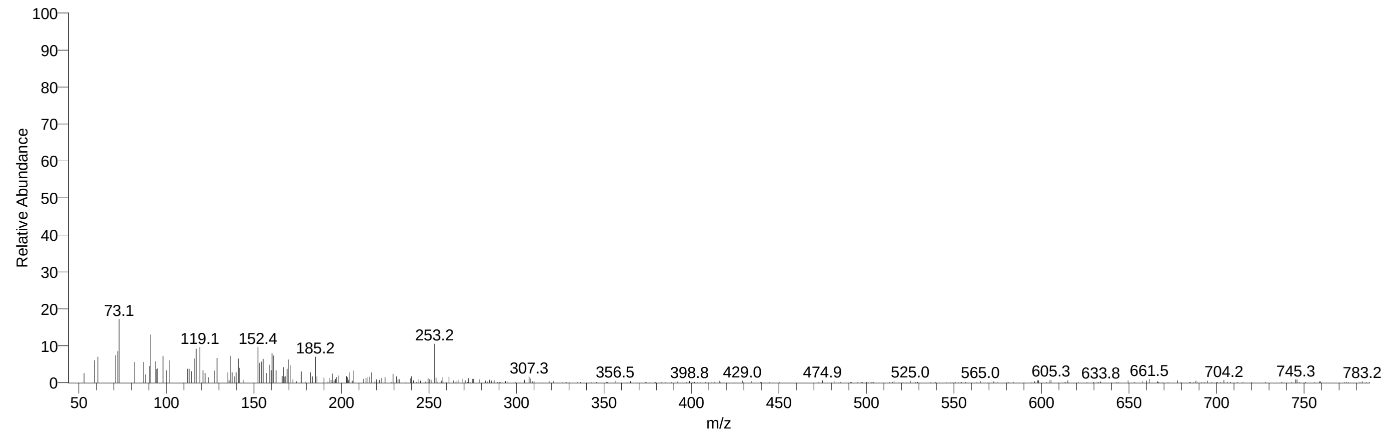

Hit Spectrum	Delta	Compound Structure

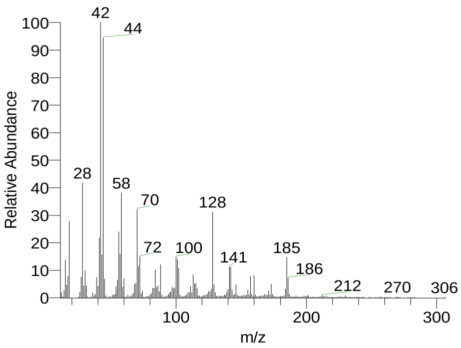

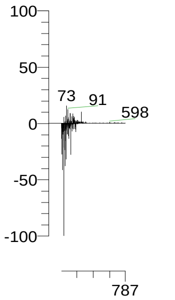

1,2-Diaminopropane-N,N,N',N'-tetraacetic acid
Formula C11H18N2O8, MW 306, CAS# 4408-81-5, Entry# 4374
Glycine, N,N'-(1-methyl-1,2-ethanediyl)bis[N-(carboxymethyl)-

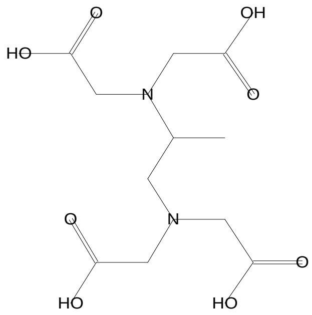

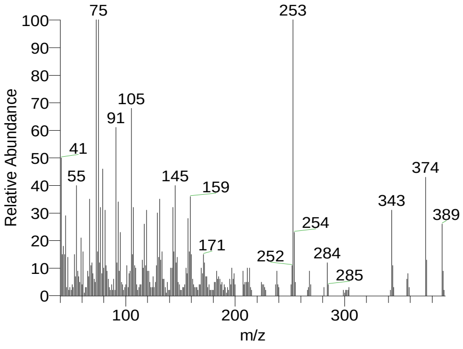

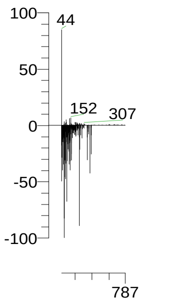

Androst-9(11)-en-17-one, 3-[(trimethylsilyl)oxy]-, O-methyloxime, (3à,5à)- (CAS)
Formula C23H39NO2Si, MW 389, CAS# 57305-05-2, Entry# 529495
5à-ANDROST-9(11)-EN-3à-OL-17-ONE MO TMS

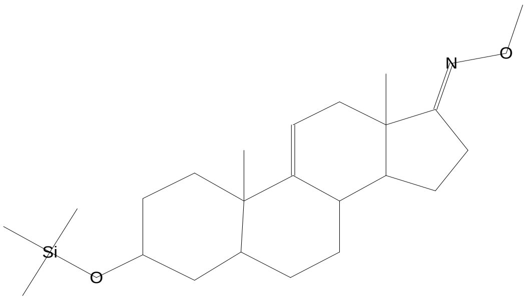

There is no signature data to report.

Library Search Report

Hit Spectrum | Delta | Compound Structure

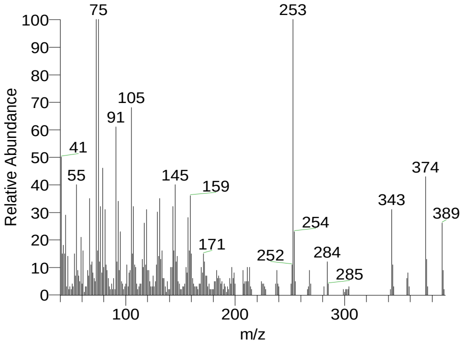

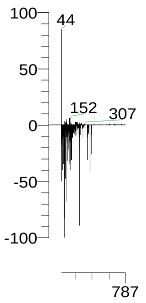

Androst-9(11)-en-17-one, 3-[(trimethylsilyl)oxy]-, O-methyloxime, (3à,5à)-
Formula C23H39NO2Si, MW 389, CAS# 57305-05-2, Entry# 37846
3-[(Trimethylsilyl)oxy]androst-9(11)-en-17-one o-methyloxime #

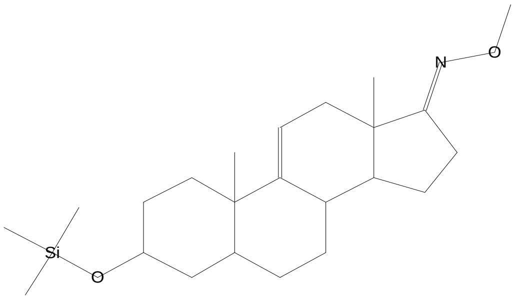

There is no signature data to report.

Library Search Report

RT	Probability	Compound Name	SI	Area %	Area	Molecular Weight	Molecular Formula	Library
40.94	7.45	1-Hydroxy-8-chloroacetylaminonaphthalene	354	0.18	574795.82	235	C12H10ClNO2	Wiley9
40.94	7.45	à-(2'-Hydroxy-5'-chlorophenyl)-2-pyridinemethanol	354	0.18	574795.82	235	C12H10ClNO2	Wiley9
40.94	4.96	à-(2'-Hydroxy-5'-chlorophenyl)-4-pyridinemethanol	342	0.18	574795.82	235	C12H10ClNO2	Wiley9

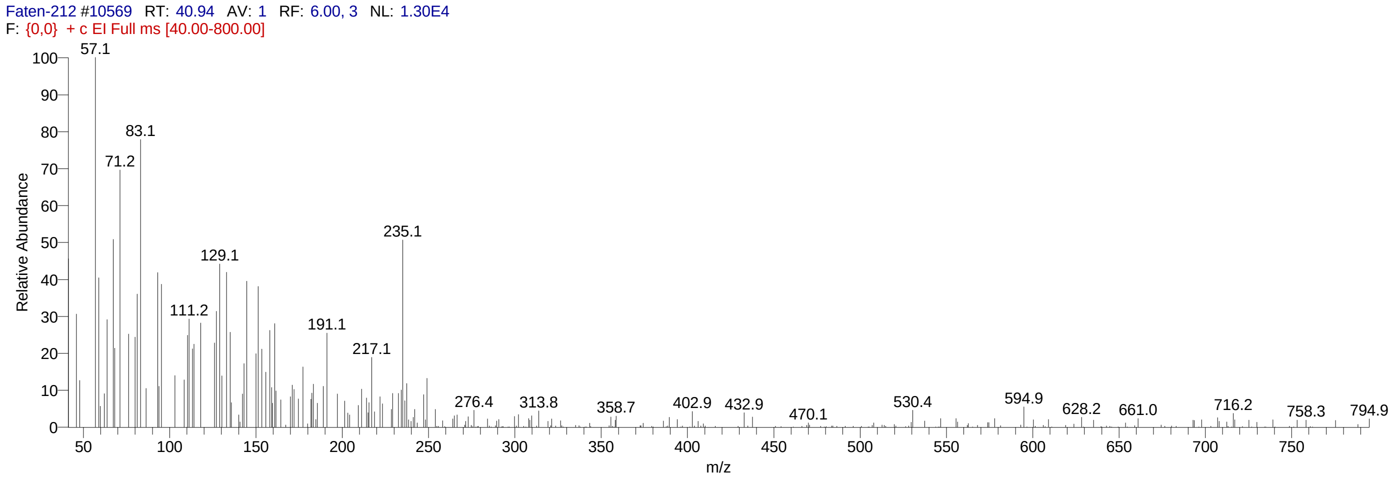

Hit Spectrum | Delta | Compound Structure

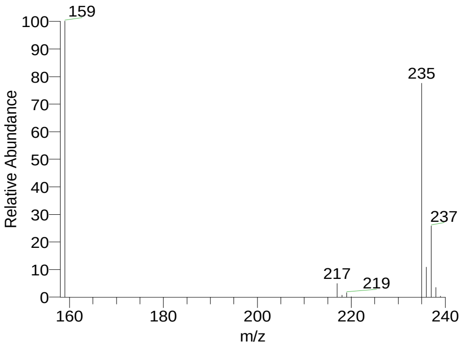

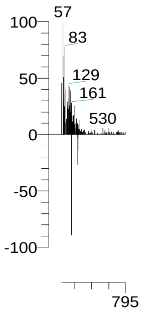

1-Hydroxy-8-chloroacetylaminonaphthalene
Formula C12H10ClNO2, MW 235, CAS# NA, Entry# 223164

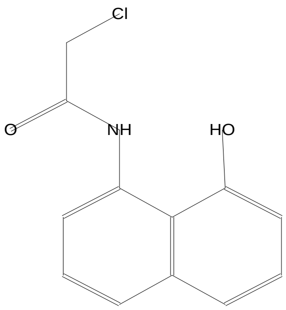

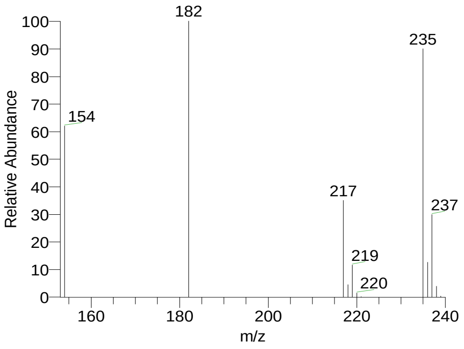

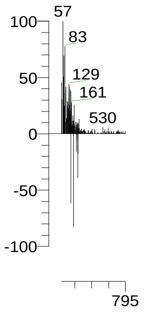

à-(2'-Hydroxy-5'-chlorophenyl)-2-pyridinemethanol
Formula C12H10ClNO2, MW 235, CAS# NA, Entry# 223161

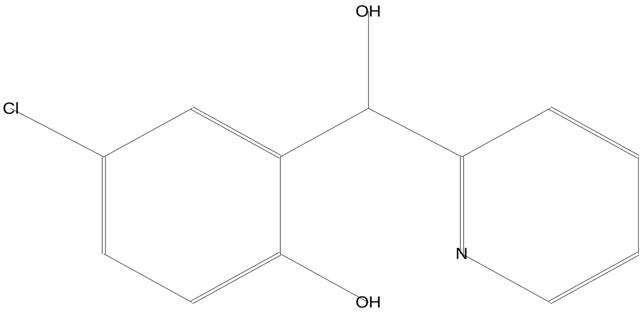

There is no signature data to report.

Library Search Report

Hit Spectrum	Delta	Compound Structure

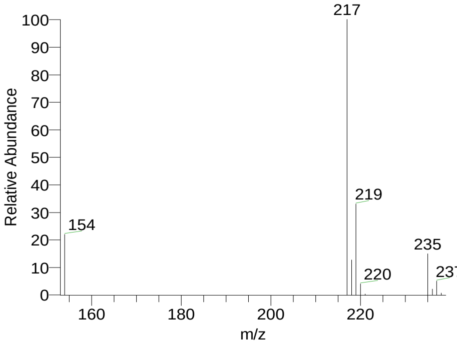

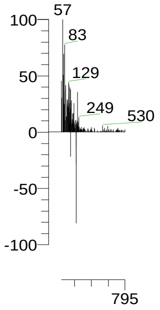

à-(2'-Hydroxy-5'-chlorophenyl)-4-pyridinemethanol
Formula C12H10ClNO2, MW 235, CAS# NA, Entry# 223159

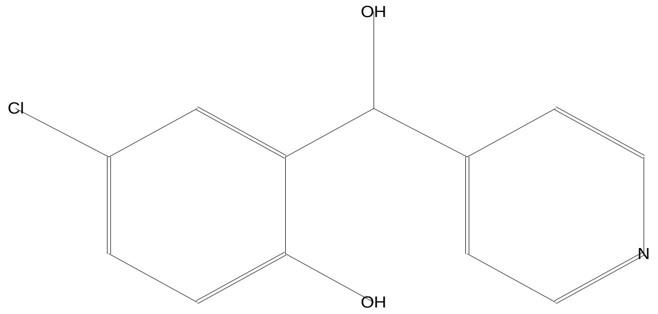

There is no signature data to report.

Library Search Report

RT	Probability	Compound Name	SI	Area %	Area	Molecular Weight	Molecular Formula	Library
41.03	8.11	2-(1-Methyl-1-silacyclobutyl)benzoic acid trimethyl-silyl ester	379	0.28	878542.98	278	C14H22O2Si2	mainlib
41.03	7.17	4,8-Bis(methylamino)-2-chloro-1,5-naphoquinone	376	0.28	878542.98	250	C12H11ClN2O2	Wiley9
41.03	6.06	1-(P-FLUOROPHENYL)-2,3-DIMETHYLNAPHTHALENE	372	0.28	878542.98	250	C18H15F	Wiley9

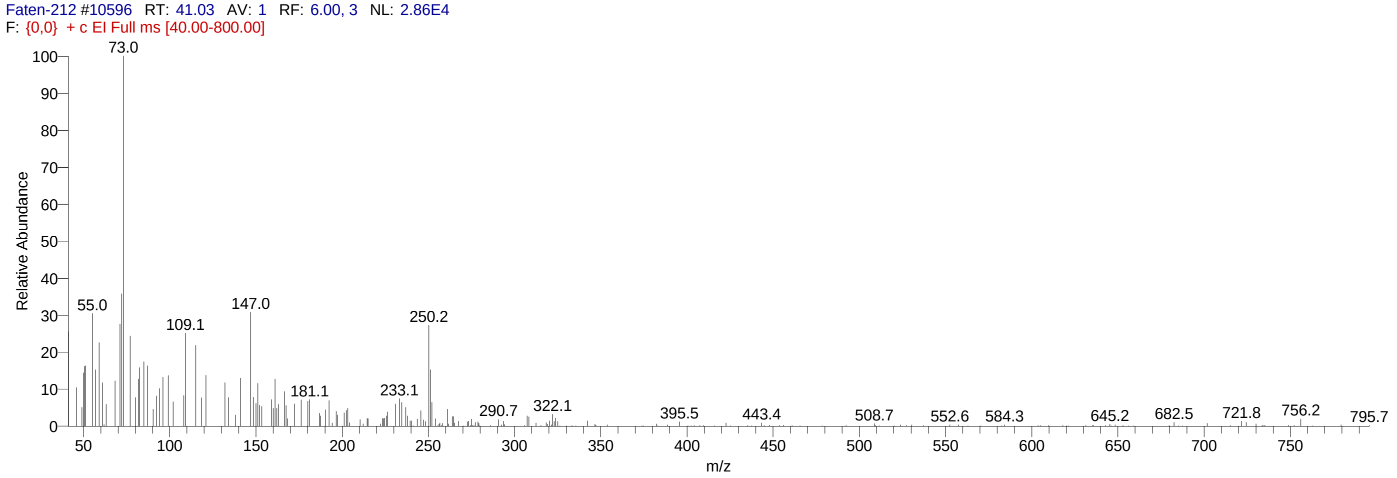

Hit Spectrum | Delta | Compound Structure

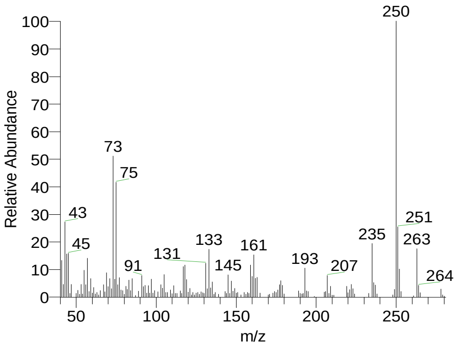

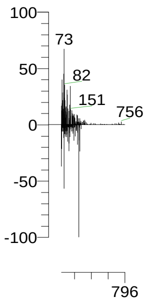

2-(1-Methyl-1-silacyclobutyl)benzoic acid trimethyl-silyl ester
Formula C14H22O2Si2, MW 278, CAS# NA, Entry# 184232
Trimethylsilyl 2-(1-methyl-1-siletanyl)benzoate #

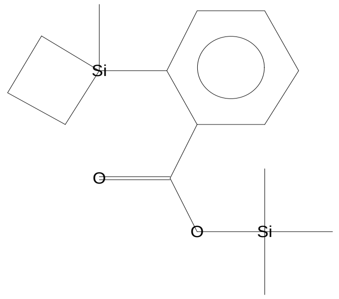

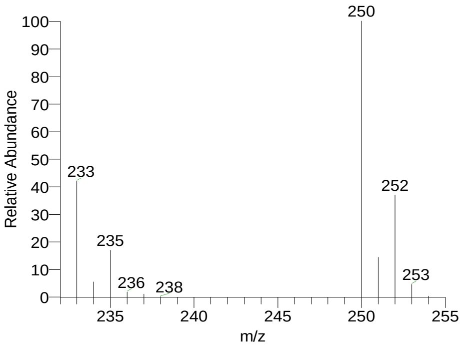

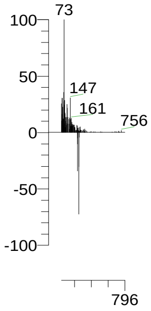

4,8-Bis(methylamino)-2-chloro-1,5-naphoquinone
Formula C12H11ClN2O2, MW 250, CAS# NA, Entry# 258253

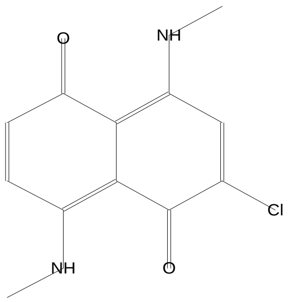

There is no signature data to report.

Library Search Report

Hit Spectrum	Delta	Compound Structure

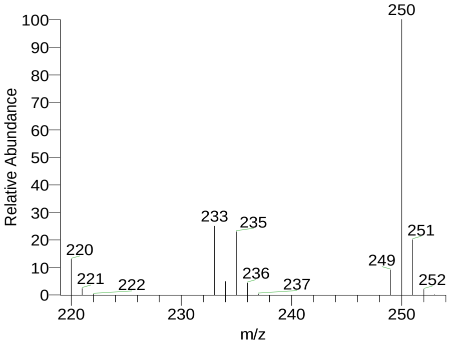

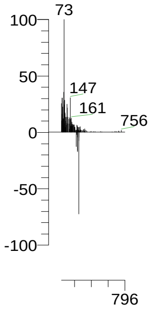

1-(P-FLUOROPHENYL)-2,3-DIMETHYLNAPHTHALENE
Formula C18H15F, MW 250, CAS# 72968-93-5, Entry# 260773
Naphthalene, 1-(4-fluorophenyl)-2,3-dimethyl- (CAS)

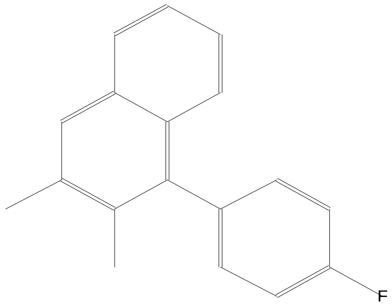

There is no signature data to report.

Library Search Report

RT	Probability	Compound Name	SI	Area %	Area	Molecular Weight	Molecular Formula	Library
41.34	6.98	Trimethylsilyl 23-acetoxy-3,6,9,12,15,18,21-heptaoxatricosan-1-oate	517	2.00	6340349.45	498	C21H42O11Si	mainlib
41.34	5.21	1-(4'-TRIMETHYSILYLOXYPHENYL)-1-TRIMETHYLSILYLOXY-2-METHYLTRIMETHYLSILYL-AMINOETHANE	509	2.00	6340349.45	383	C18H37NO2Si3	Wiley9
41.34	3.99	2-[2-[2-[2-[2-[2-[2-(2-Methoxyethoxy)ethoxy]ethoxy]ethoxy]ethoxy]ethoxy]ethoxy-trimethylsilane	502	2.00	6340349.45	456	C20H44O9Si	mainlib

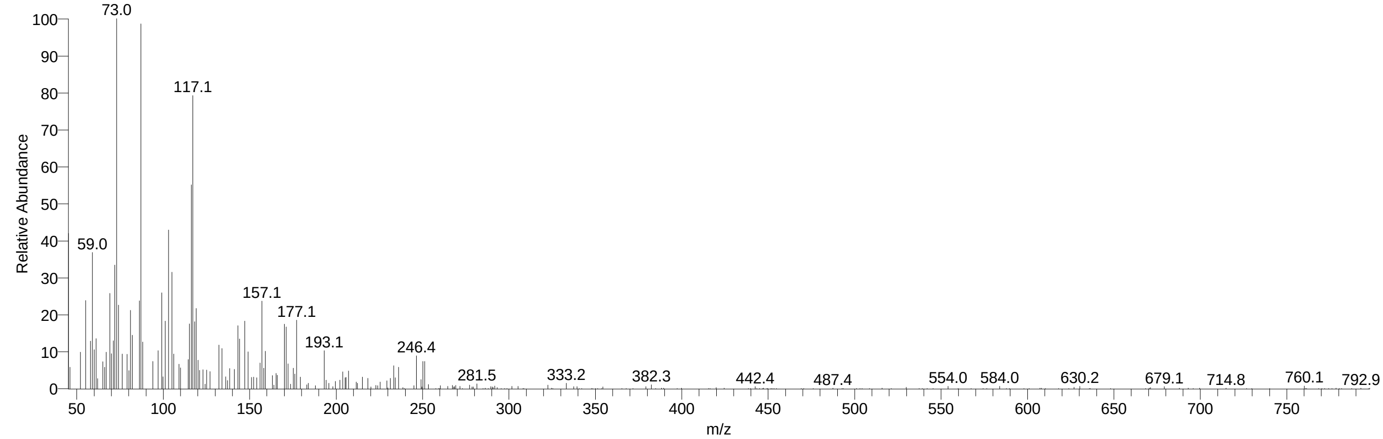

Hit Spectrum	Delta	Compound Structure

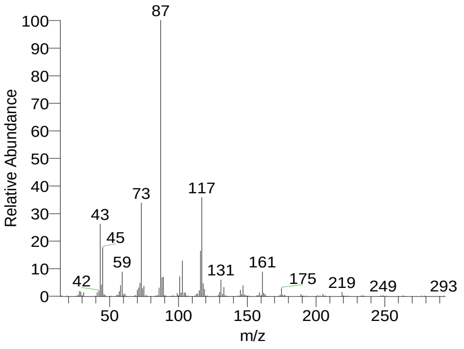

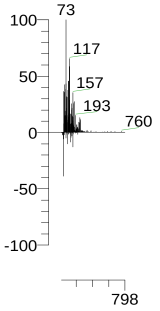

Trimethylsilyl 23-acetoxy-3,6,9,12,15,18,21-heptaoxatricosan-1-oate
Formula C21H42O11Si, MW 498, CAS# NA, Entry# 52269

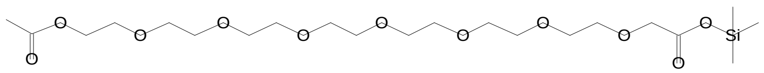

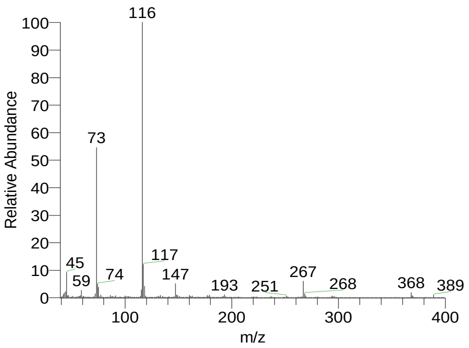

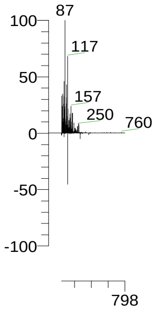

Formula C18H37NO2Si3, MW 383, CAS# NA, Entry# 520945

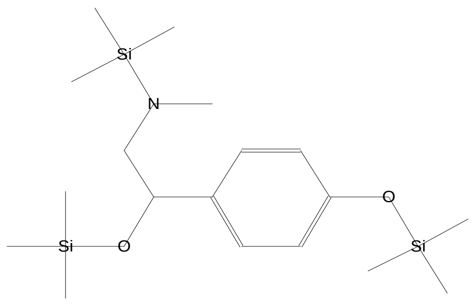

There is no signature data to report.

Library Search Report

Hit Spectrum | Delta | Compound Structure

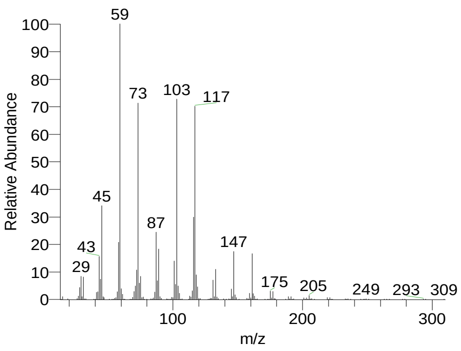

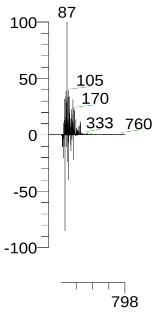

2-[2-[2-[2-[2-[2-[2-(2-Methoxyethoxy)ethoxy]ethoxy]ethoxy]ethoxy]ethoxy]ethoxy]ethoxy-trimethylsilane
Formula C20H44O9Si, MW 456, CAS# NA, Entry# 27898
2,2-Dimethyl-3,6,9,12,15,18,21,24,27-nonaoxa-2-silaoctacosane

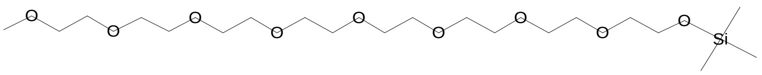

There is no signature data to report.

Library Search Report

RT	Probability	Compound Name	SI	Area %	Area	Molecular Weight	Molecular Formula	Library
41.75	9.58	(3E)-5-Isopropyliden-6-methyl-3,6,9-decatrien-2-one	532	1.16	3689071.10	204	C14H20O	Wiley9
41.75	6.76	Methyl 2,4-tridecadiynoate	522	1.16	3689071.10	220	C14H20O2	mainlib
41.75	6.50	5-Isopropylidene-6-methylhyldeca-3,6,9-trien-2-one	521	1.16	3689071.10	204	C14H20O	mainlib

Faten-212 #10805 RT: 41.75 AV: 1 RF: 6.00, 3 NL: 9.11E4
F: {0,0} + c EI Full ms [40.00-800.00]

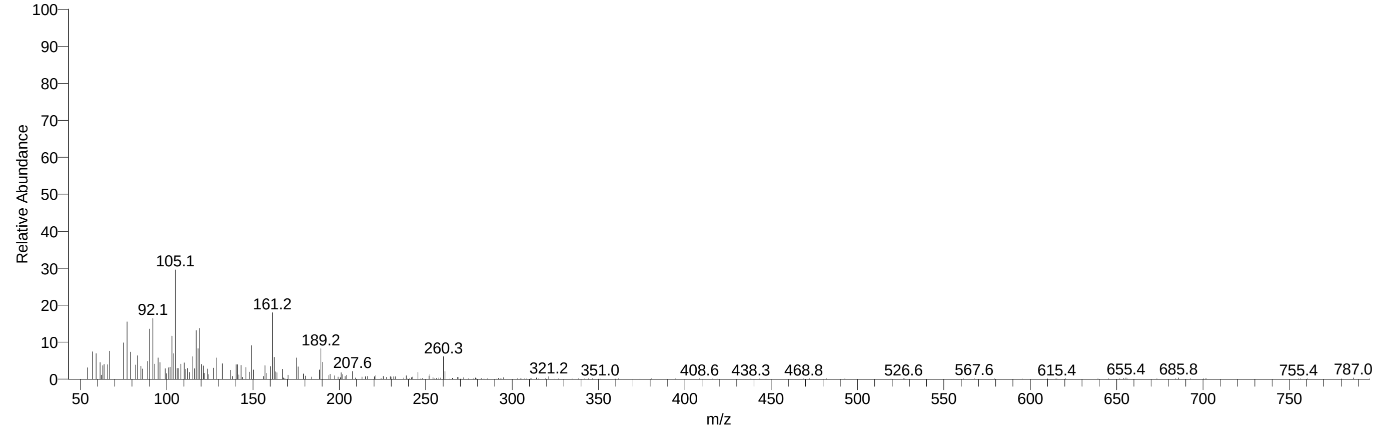

Hit Spectrum | Delta | Compound Structure

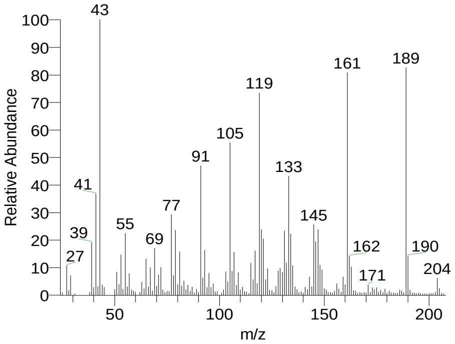

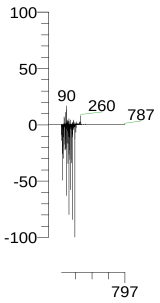

(3E)-5-Isopropyliden-6-methyl-3,6,9-decatrien-2-one
Formula C14H20O, MW 204, CAS# NA, Entry# 151496
5-Isopropylidene-6-methyldeca-3,6,9-trien-2-one

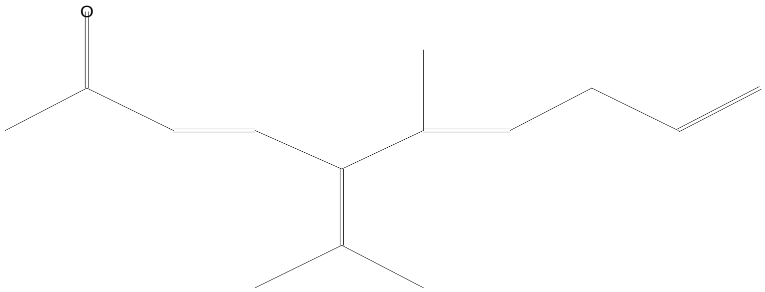

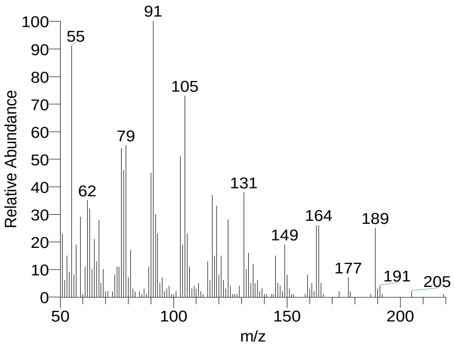

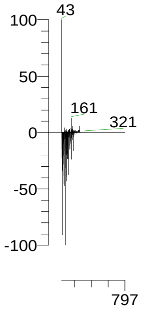

Methyl 2,4-tridecadiynoate
Formula C14H20O2, MW 220, CAS# NA, Entry# 53887

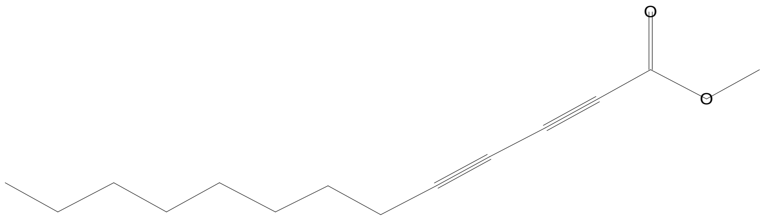

There is no signature data to report.

Library Search Report

Hit Spectrum	Delta	Compound Structure

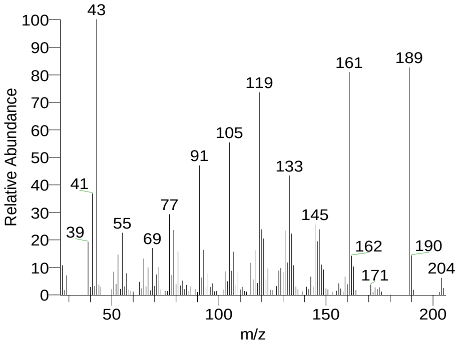

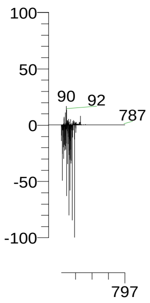

5-Isopropylidene-6-methyldeca-3,6,9-trien-2-one
Formula C14H20O, MW 204, CAS# NA, Entry# 12568
(3E,6E)-6-Methyl-5-(1-methylethylidene)-3,6,9-decatrien-2-one #

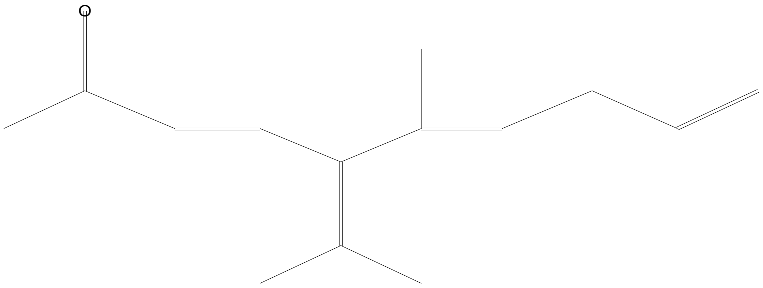

There is no signature data to report.

Library Search Report

RT	Probability	Compound Name	SI	Area %	Area	Molecular Weight	Molecular Formula	Library
41.98	22.90	Benzene, (1-butyloctyl)- (CAS)	576	0.64	2023222.66	246	C18H30	Wiley9
41.98	11.12	Benzene, (1-butylhexadecyl)- (CAS)	557	0.64	2023222.66	358	C26H46	Wiley9
41.98	6.96	Pentadecylbenzene	543	0.64	2023222.66	288	C21H36	Wiley9

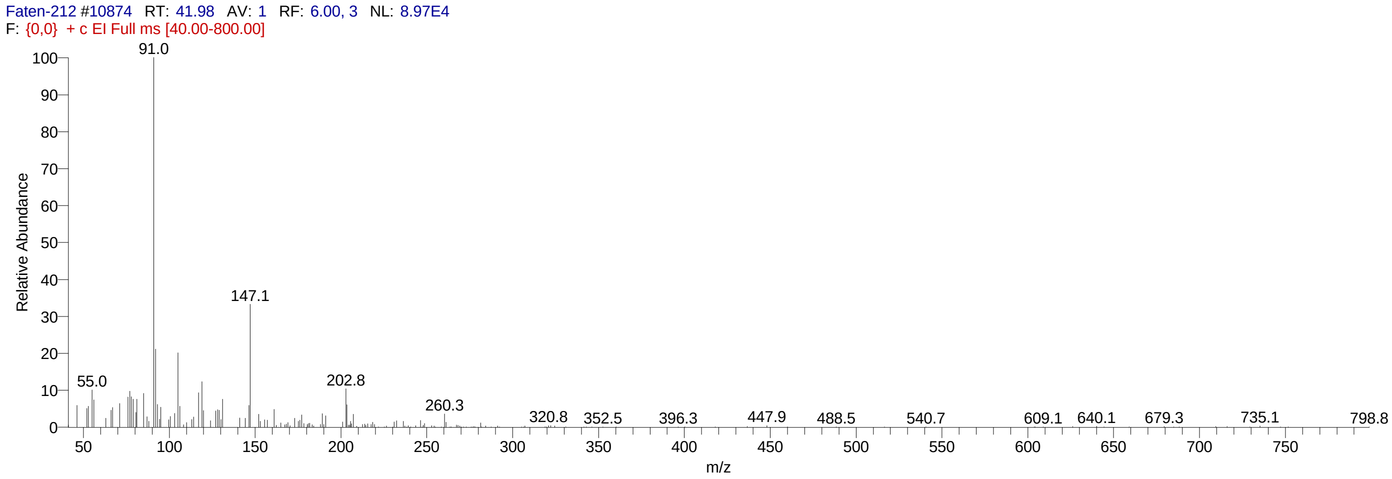

Hit Spectrum	Delta	Compound Structure

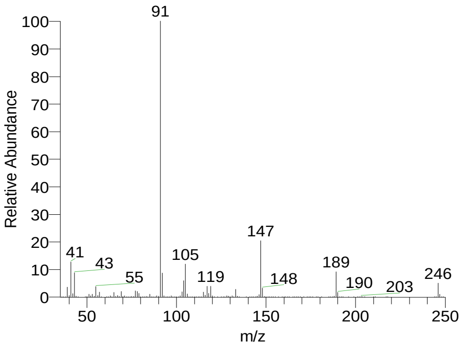

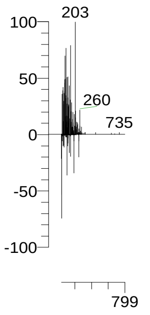

Benzene, (1-butyloctyl)- (CAS)
Formula C18H30, MW 246, CAS# 2719-63-3, Entry# 251015
5-Phenyldodecane

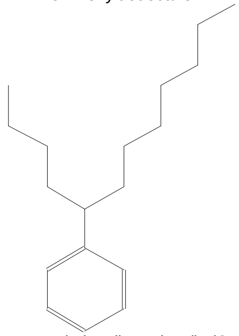

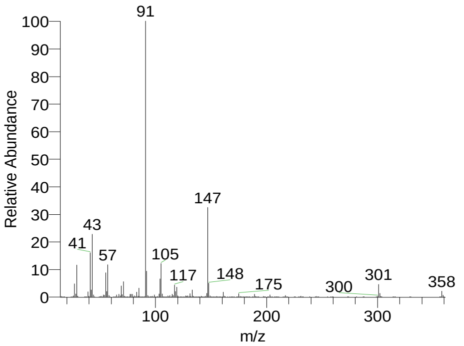

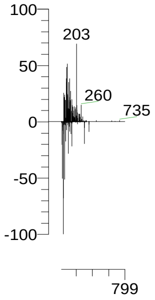

Benzene, (1-butylhexadecyl)- (CAS)
Formula C26H46, MW 358, CAS# 2400-04-6, Entry# 485833
Eicosane, 5-phenyl-

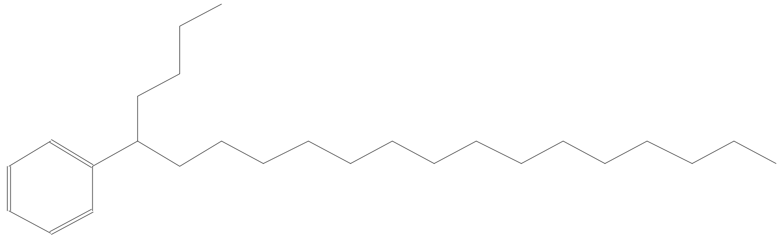

There is no signature data to report.

Library Search Report

Hit Spectrum	Delta	Compound Structure

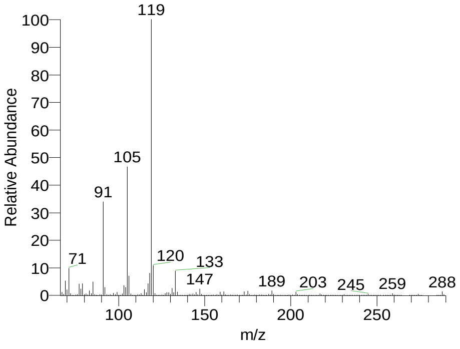

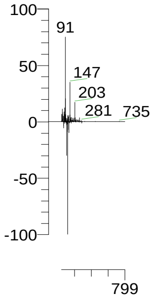

Pentadecylbenzene
Formula C21H36, MW 288, CAS# 2131-18-2, Entry# 349051
Benzene, pentadecyl- (CAS)

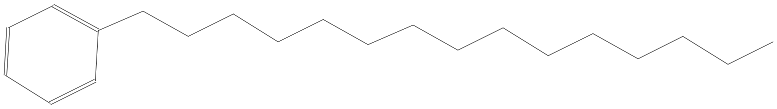

There is no signature data to report.

Library Search Report

RT	Probability	Compound Name	SI	Area %	Area	Molecular Weight	Molecular Formula	Library
42.45	39.04	(E)-4-methylene-1-phenyl-1,5-hexadien-3-ol	537	0.61	1949300.89	186	C13H14O	Wiley9
42.45	8.61	2,6-Dibromophenyl á-phenylpropionate	494	0.61	1949300.89	382	C15H12Br2O2	mainlib
42.45	8.61	3-Pentanone, 1,5-diphenyl- (CAS)	494	0.61	1949300.89	238	C17H18O	Wiley9

Faten-212 #11011 RT: 42.45 AV: 1 RF: 6.00, 3 NL: 5.04E4
F: {0,0} + c EI Full ms [40.00-800.00]

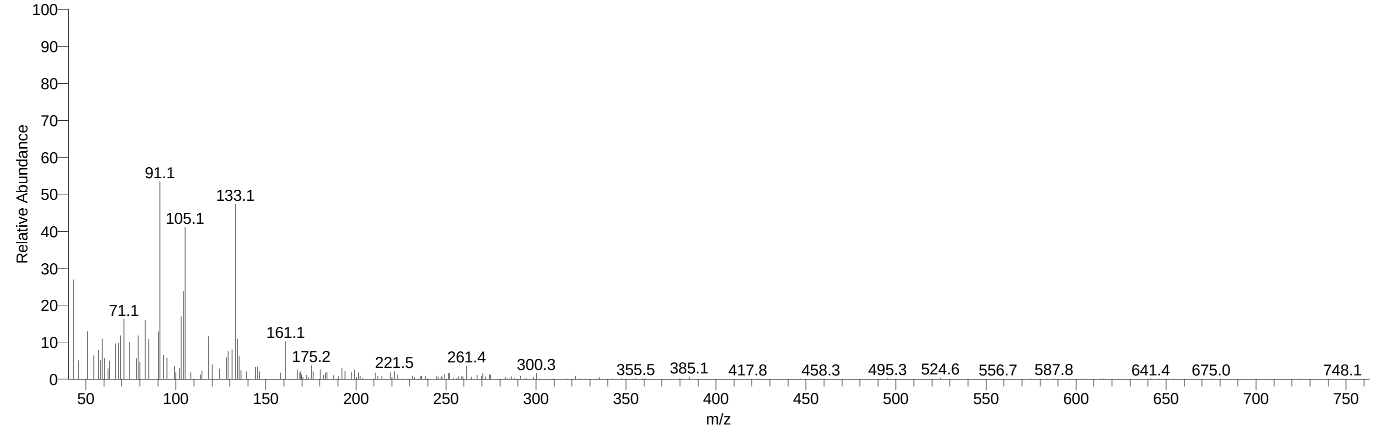

Hit Spectrum	Delta	Compound Structure

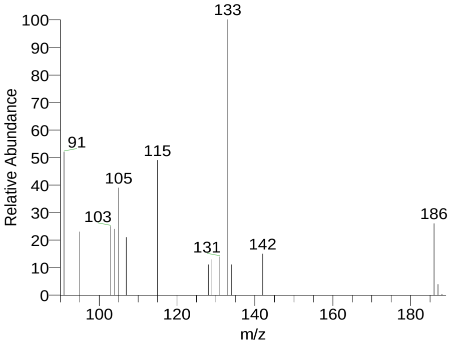

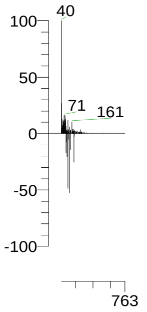

(E)-4-methylene-1-phenyl-1,5-hexadien-3-ol
Formula C13H14O, MW 186, CAS# 100281-14-9, Entry# 114691
1,5-Hexadien-3-ol, 4-methylene-1-phenyl-, (E)- (CAS)

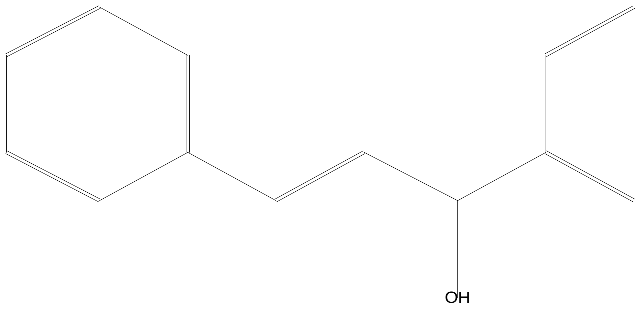

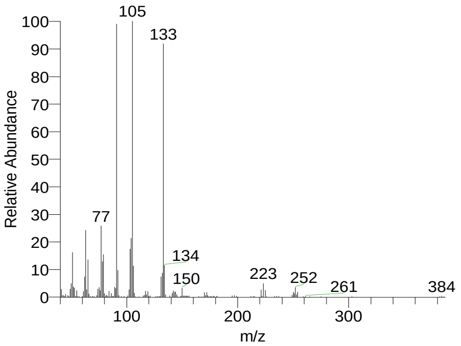

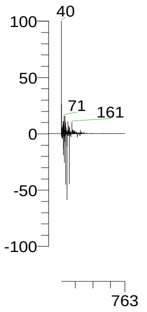

2,6-Dibromophenyl á-phenylpropionate
Formula C15H12Br2O2, MW 382, CAS# 40123-52-2, Entry# 72523
2,6-Dibromophenyl 3-phenylpropanoate #

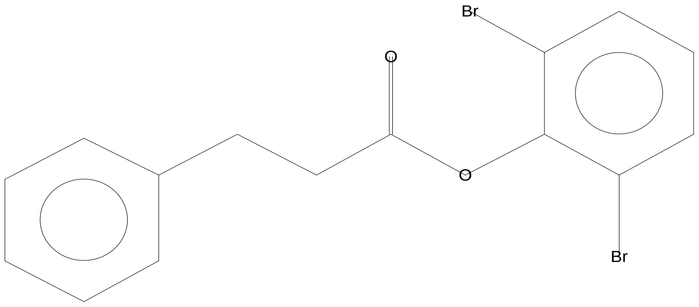

There is no signature data to report.

Library Search Report

Hit Spectrum	Delta	Compound Structure

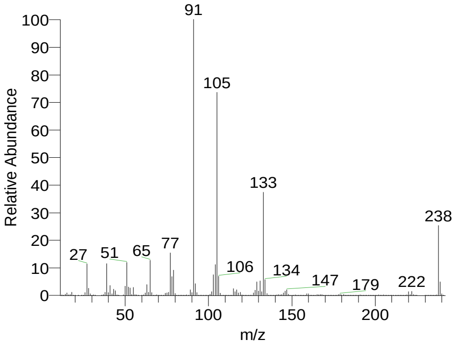

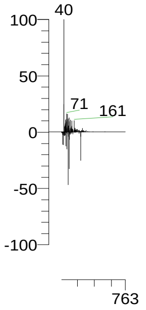

3-Pentanone, 1,5-diphenyl- (CAS)
Formula C17H18O, MW 238, CAS# 5396-91-8, Entry# 232843
1,5-Diphenyl-3-pentanone

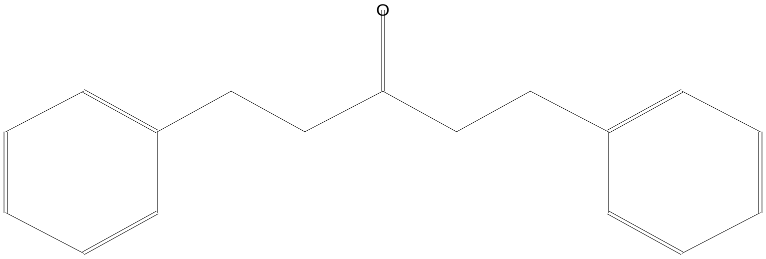

There is no signature data to report.

Library Search Report

RT	Probability	Compound Name	SI	Area %	Area	Molecular Weight	Molecular Formula	Library
43.13	85.59	Ethyl-(Z)-2-chloromethylene-2-[2',4',6'-tri(t-butyl)phenyl]-phosphanylidene-acetate	818	0.43	1357074.90	396	C22H34ClO2P	Wiley9
43.13	4.62	(22S,23R,25R)-3.beta-.Acetoxy-16á,23:23,26-diepoxycholest-5-ene	699	0.43	1357074.90	456	C29H44O4	Wiley9
43.13	2.89	(+-)-10,11-cis-Dihydro-10-isopropyl-4-propyl-6,6,11-trimethyl-2H,6H,12H-benzo[1,2-b:3,4:b',5,6-b'']tripyran-2,12-dione	685	0.43	1357074.90	396	C24H28O5	Wiley9

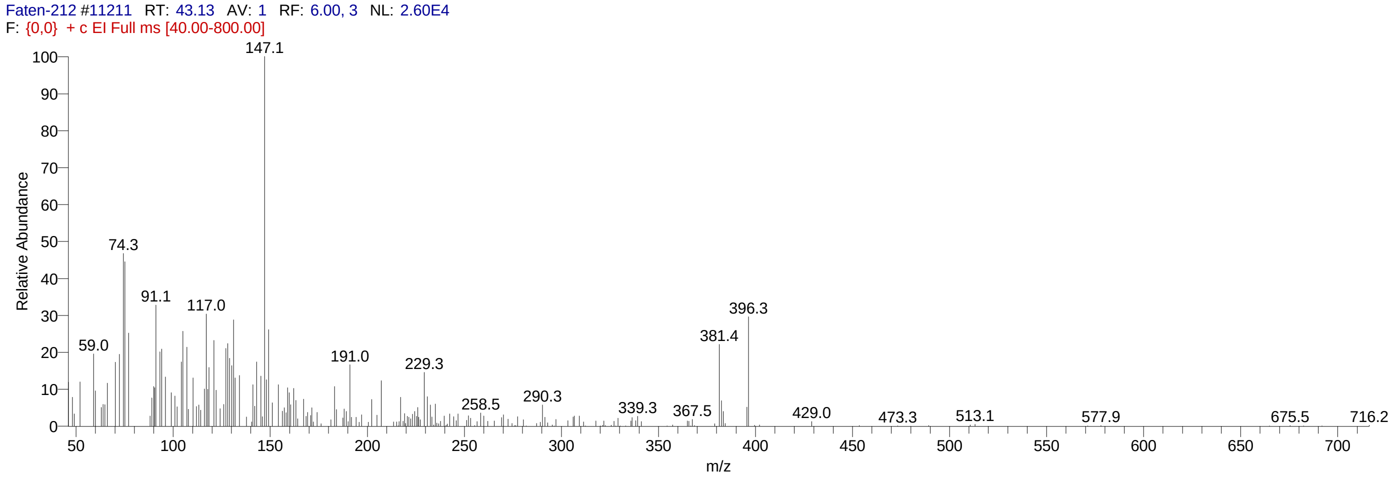

Hit Spectrum | Delta | Compound Structure

Ethyl-(Z)-2-chloromethylene-2-[2',4',6'-tri(t-butyl)phenyl]-phosphanylidene-acetate
Formula C22H34ClO2P, MW 396, CAS# NA, Entry# 537623

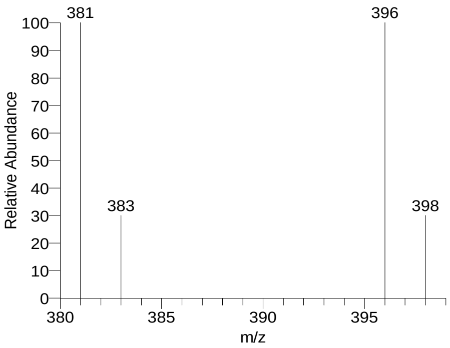

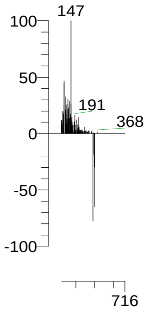

(22S,23R,25R)-3.beta-.Acetoxy-16á,23:23,26-diepoxycholest-5-ene
Formula C29H44O4, MW 456, CAS# NA, Entry# 593492

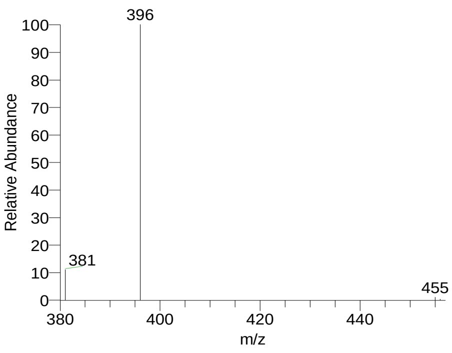

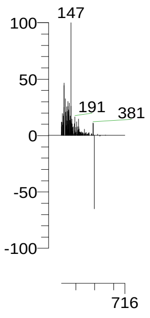

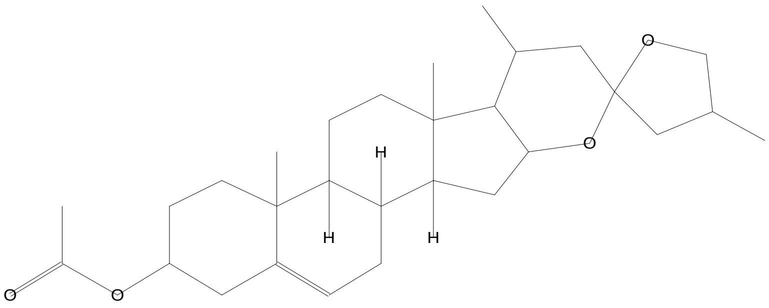

There is no signature data to report.

Library Search Report

Hit Spectrum	Delta	Compound Structure

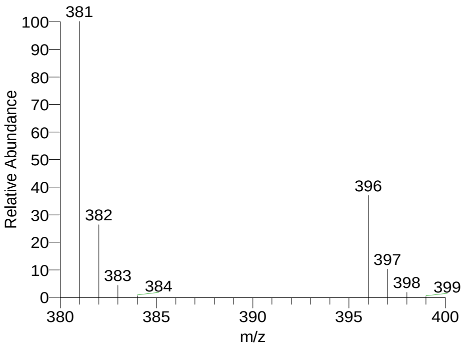

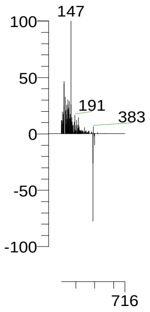

Formula C24H28O5, MW 396, CAS# NA, Entry# 537909

There is no signature data to report.

Library Search Report

RT	Probability	Compound Name	SI	Area %	Area	Molecular Weight	Molecular Formula	Library
43.32	9.27	cis-4,7,10,13,16,19-Docosahexaenoic acid, trimethylsilyl ester	456	0.50	1596754.63	400	C25H40O2Si	mainlib
43.32	7.83	cis-4,7,10,13,16,19-Docosahexaenoic acid, tert-butyldimethylsilyl ester	452	0.50	1596754.63	442	C28H46O2Si	mainlib
43.32	4.04	Pentacosane, 13-phenyl-	434	0.50	1596754.63	428	C31H56	mainlib

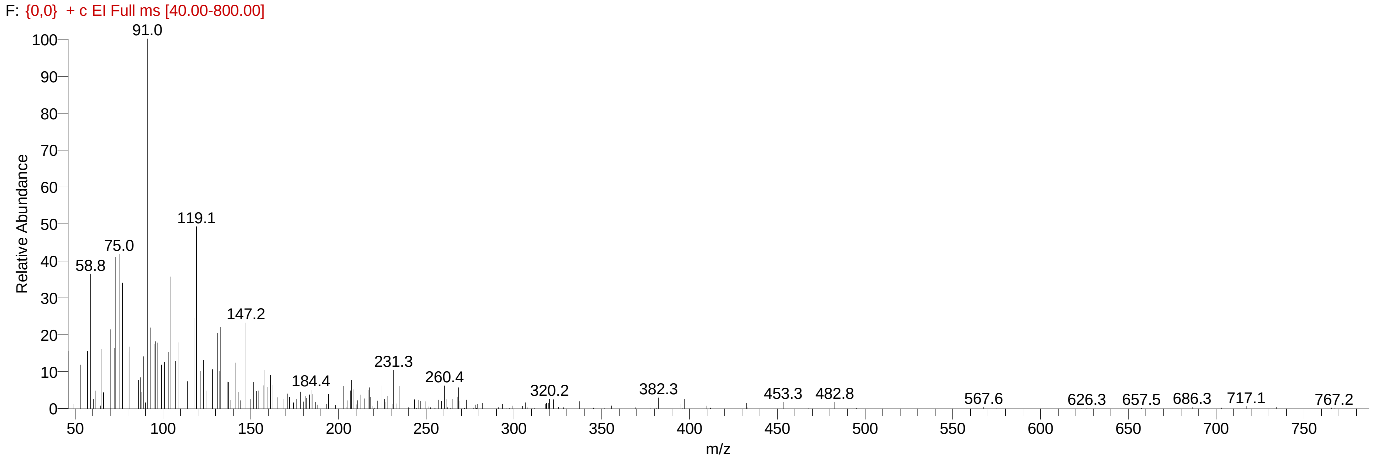

Hit Spectrum	Delta	Compound Structure

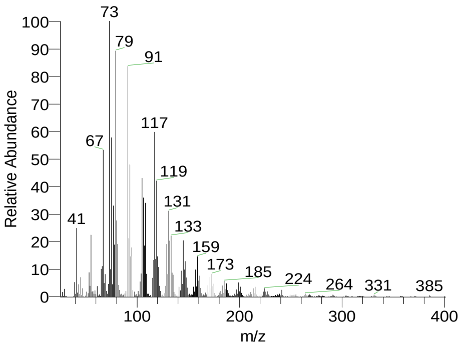

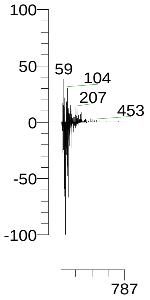

cis-4,7,10,13,16,19-Docosahexaenoic acid, trimethylsilyl ester
Formula C25H40O2Si, MW 400, CAS# NA, Entry# 37920

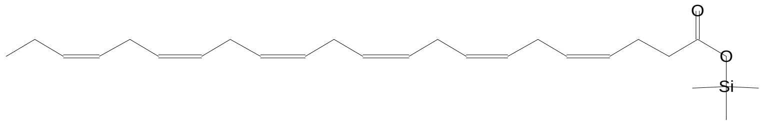

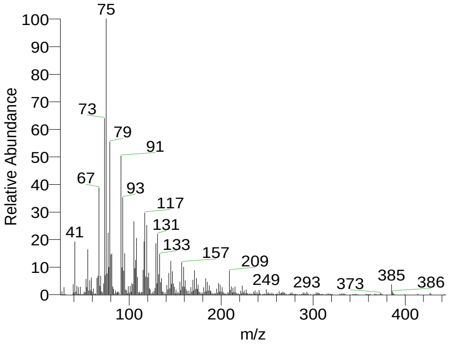

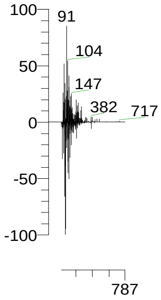

cis-4,7,10,13,16,19-Docosahexaenoic acid, tert-butyldimethylsilyl ester
Formula C28H46O2Si, MW 442, CAS# NA, Entry# 41215

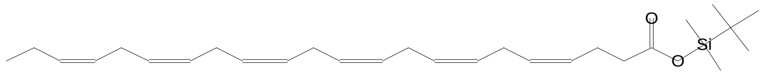

There is no signature data to report.

Library Search Report

Hit Spectrum	Delta	Compound Structure

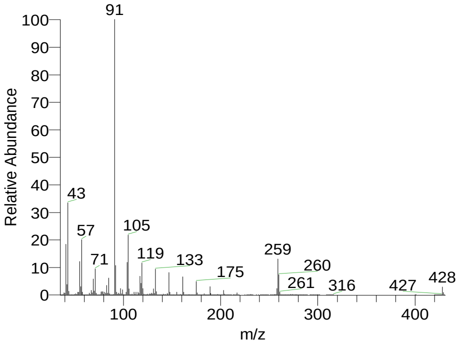

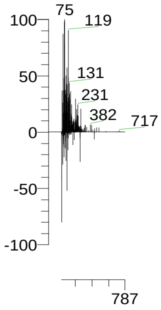

Pentacosane, 13-phenyl-
Formula C31H56, MW 428, CAS# 6006-90-2, Entry# 53730
Benzene, (1-dodecyltridecyl)-

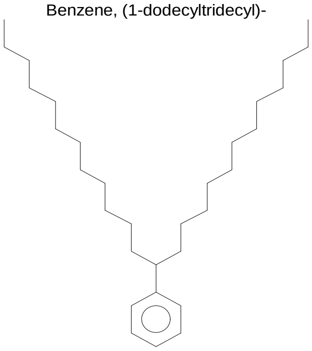

There is no signature data to report.

Library Search Report

RT	Probability	Compound Name	SI	Area %	Area	Molecular Weight	Molecular Formula	Library
43.64	26.77	Butanoic acid, heptafluoro-, methyl ester (CAS)	417	0.40	1259776.60	228	C5H3F7O2	Wiley9
43.64	26.77	Butanoic acid, heptafluoro-, methyl ester (CAS)	414	0.40	1259776.60	228	C5H3F7O2	Wiley9
43.64	6.51	6,6'-Dimethyl-5,5',8,8'-tetrahydroxy-2,2'-binaphthalene-1,1',4,4'-tetrone	380	0.40	1259776.60	406	C22H14O8	Wiley9

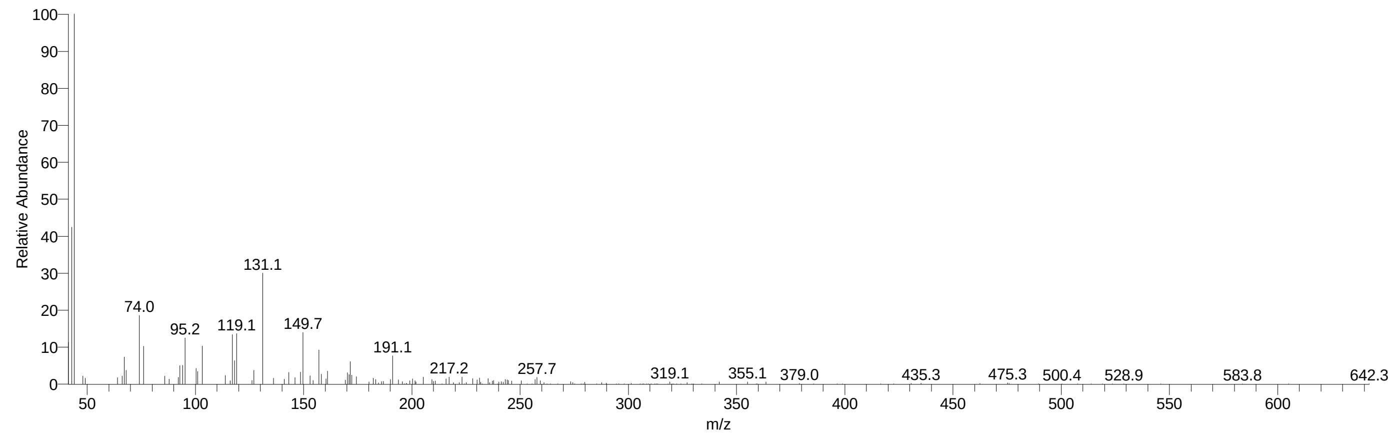

Hit Spectrum	Delta	Compound Structure

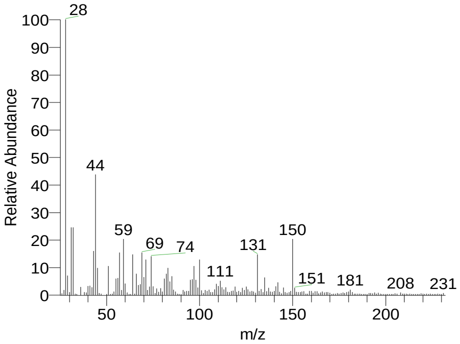

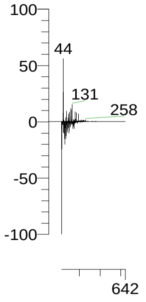

Butanoic acid, heptafluoro-, methyl ester (CAS)
Formula C5H3F7O2, MW 228, CAS# 356-24-1, Entry# 206198
Methyl perfluorobutyrate

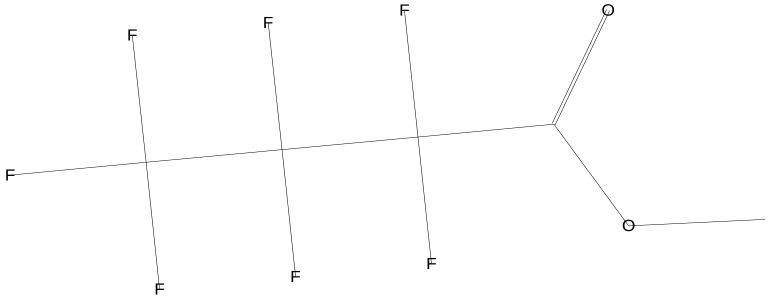

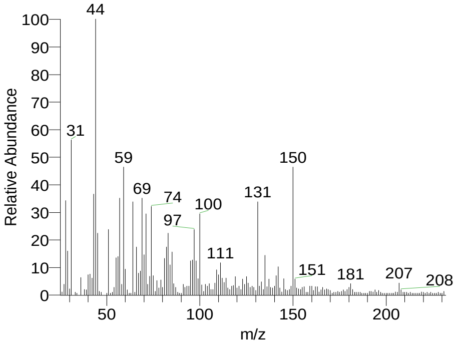

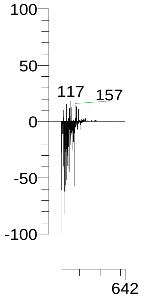

Butanoic acid, heptafluoro-, methyl ester (CAS)
Formula C5H3F7O2, MW 228, CAS# 356-24-1, Entry# 206200
Methyl perfluorobutyrate

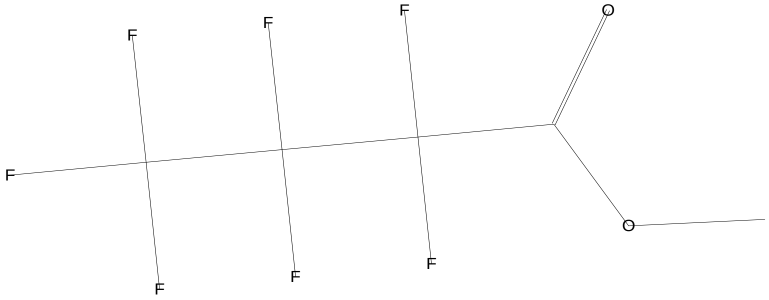

There is no signature data to report.

Library Search Report

Hit Spectrum	Delta	Compound Structure

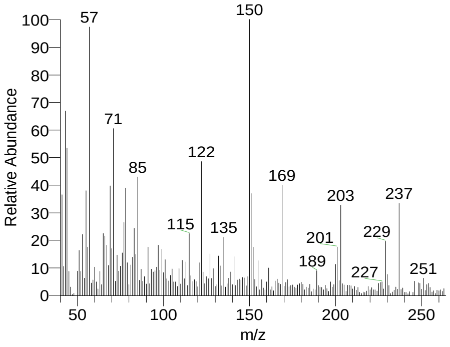

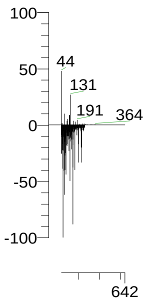

6,6'-Dimethyl-5,5',8,8'-tetrahydroxy-2,2'-binaphthalene-1,1',4,4'-tetrone
Formula C22H14O8, MW 406, CAS# 104505-76-2, Entry# 549166

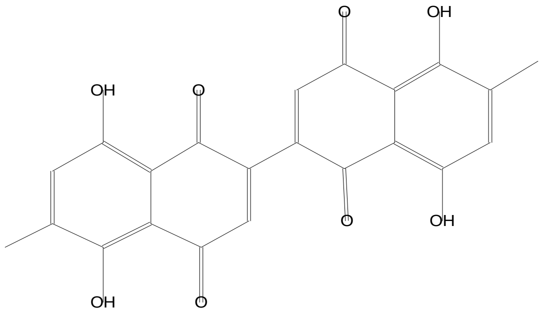

There is no signature data to report.

Library Search Report

RT	Probability	Compound Name	SI	Area %	Area	Molecular Weight	Molecular Formula	Library
43.90	11.87	2,2,18,18-TETRAMETHYL-3,6,10,13,17-PENTAOXA-2,18-DISILANEONADECANE	602	2.22	7060952.45	366	C16H38O5Si2	Wiley9
43.90	5.41	PENTITOL-1,1-D2, 2-DESOXY-TETRAKIS-O-(TRIMETHYLSILYL)-	582	2.22	7060952.45	424	C17H42D2O4Si4	Wiley9
43.90	4.57	3,6,10,14,17-Pentaoxa-2,18-disilanonadecane, 2,2,18,18-tetramethyl-	578	2.22	7060952.45	366	C16H38O5Si2	mainlib

Faten-212 #11439 RT: 43.90 AV: 1 RF: 6.00, 3 NL: 2.33E5
F: {0,0} + c EI Full ms [40.00-800.00]

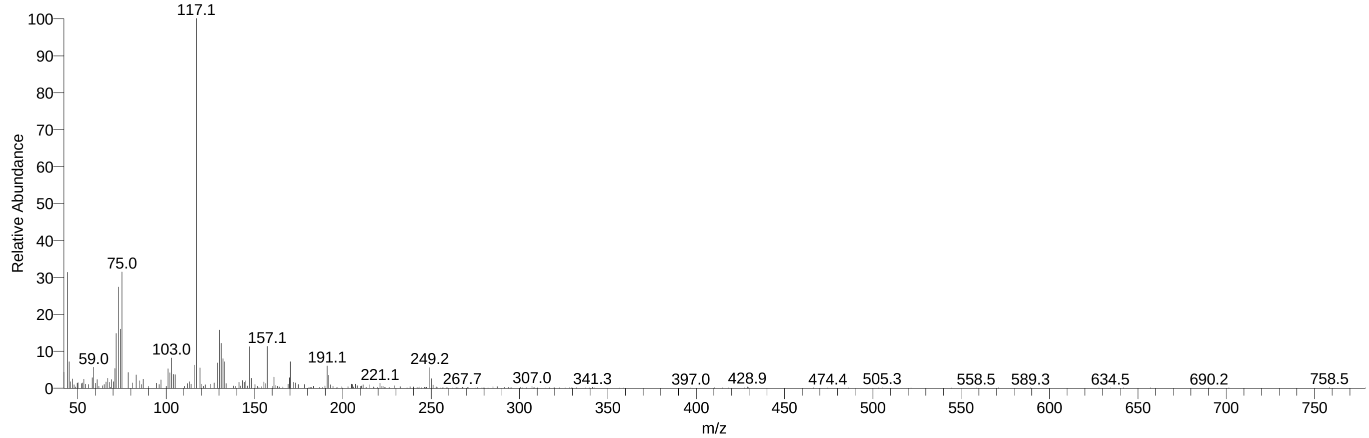

Hit Spectrum	Delta	Compound Structure

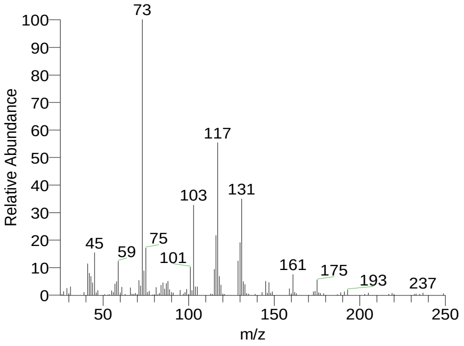

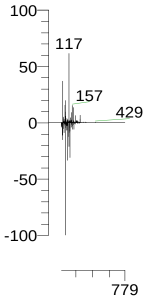

2,2,18,18-TETRAMETHYL-3,6,10,13,17-PENTAOXA-2,18-DISILANEONADECANE
Formula C16H38O5Si2, MW 366, CAS# NA, Entry# 496578

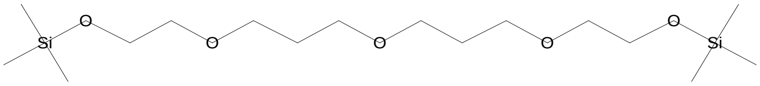

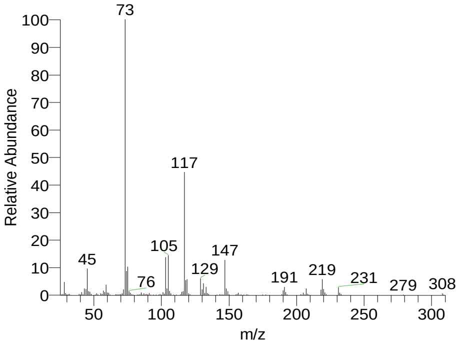

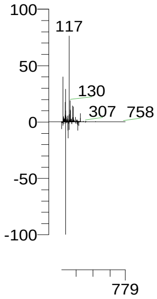

PENTITOL-1,1-D2, 2-DESOXY-TETRAKIS-O-(TRIMETHYLSILYL)-
Formula C17H42D2O4Si4, MW 424, CAS# 61141-90-0, Entry# 567212

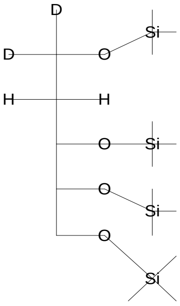

There is no signature data to report.

Library Search Report

Hit Spectrum	Delta	Compound Structure

3,6,10,14,17-Pentaoxa-2,18-disilanonadecane, 2,2,18,18-tetramethyl-
Formula C16H38O5Si2, MW 366, CAS# NA, Entry# 38406
2,2,18,18-Tetramethyl-3,6,10,14,17-pentaoxa-2,18-disilanonadecane #

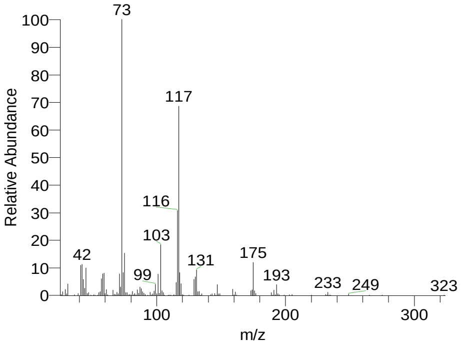

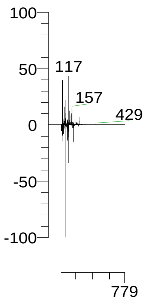

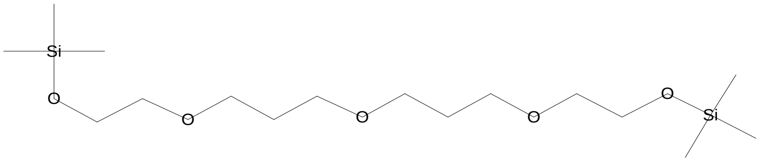

There is no signature data to report.

Library Search Report

RT	Probability	Compound Name	SI	Area %	Area	Molecular Weight	Molecular Formula	Library
44.41	6.67	2-tert-Butyl-4,6-dinitrophenyl acetate	409	0.23	732876.13	282	C12H14N2O6	mainlib
44.41	6.15	1-(2-ALLYL-PHENOXY)-3-METHYLAMINO-PROPAN-2-OL	407	0.23	732876.13	221	C13H19NO2	Wiley9
44.41	5.68	Acetamide, N-[2-chloro-4-(2,4-dimethoxybenzylidenamino phenyl]-	405	0.23	732876.13	332	C17H17ClN2O3	mainlib

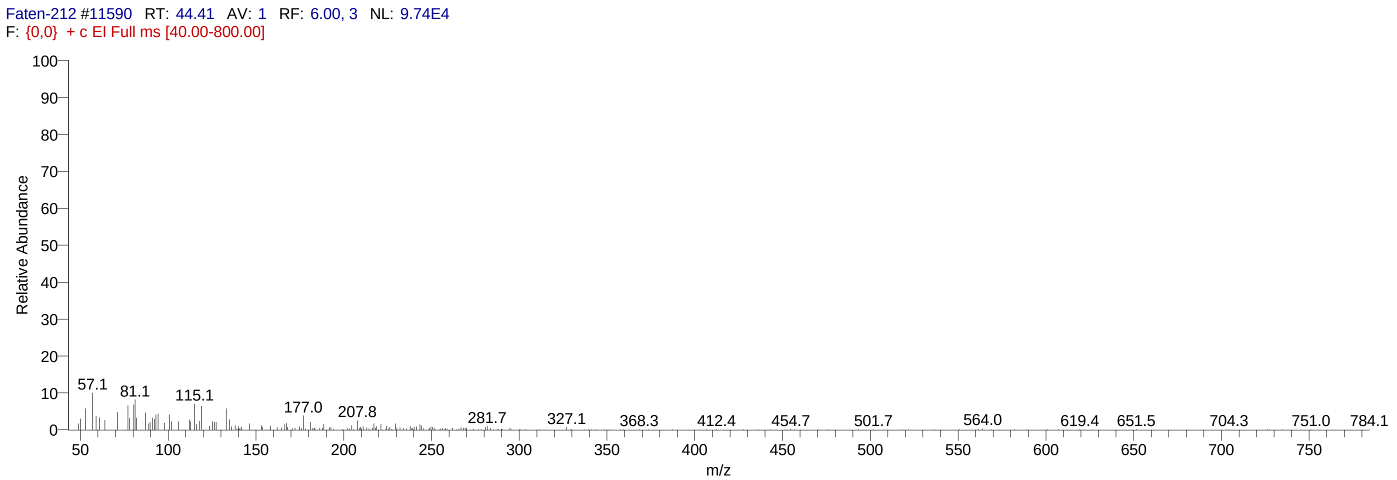

Hit Spectrum | Delta | Compound Structure

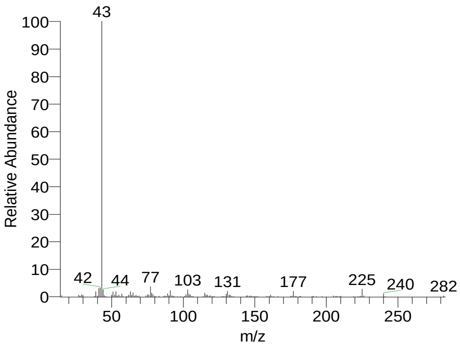

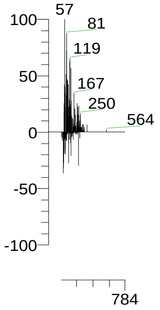

2-tert-Butyl-4,6-dinitrophenyl acetate
Formula C12H14N2O6, MW 282, CAS# NA, Entry# 8822
2-tert-Butyl-4,6-dinitro-phenol acetate

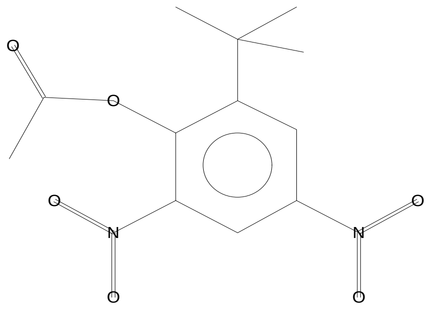

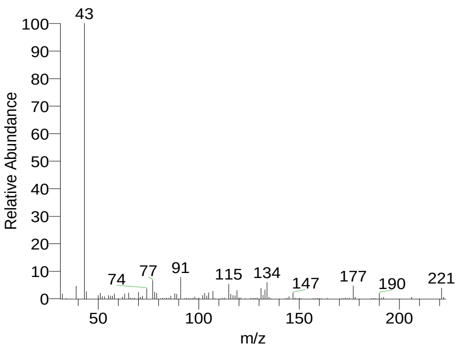

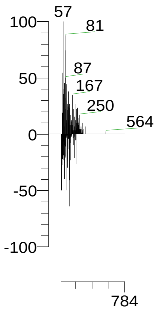

1-(2-ALLYL-PHENOXY)-3-METHYLAMINO-PROPAN-2-OL
Formula C13H19NO2, MW 221, CAS# NA, Entry# 190653
1-(2-ALLYLPHENOXY)-3-(METHYLAMINO)-2-PROPANOL

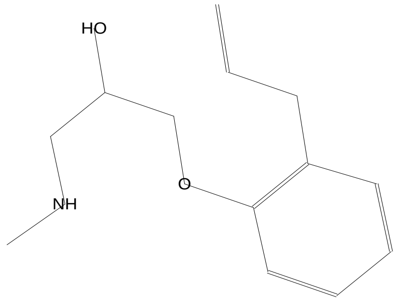

There is no signature data to report.

Library Search Report

Hit Spectrum	Delta	Compound Structure

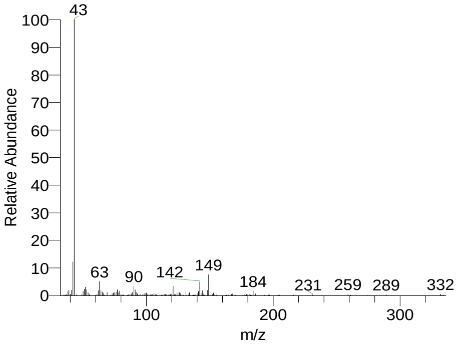

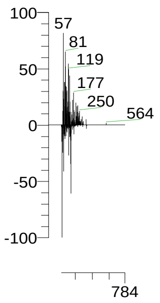

Acetamide, N-[2-chloro-4-(2,4-dimethoxybenzylidenaminophenyl]-
Formula C17H17ClN2O3, MW 332, CAS# NA, Entry# 6291
N-(2-Chloro-4-([(E)-(2,4-dimethoxyphenyl)methylidene]amino)phenyl)acetamide #

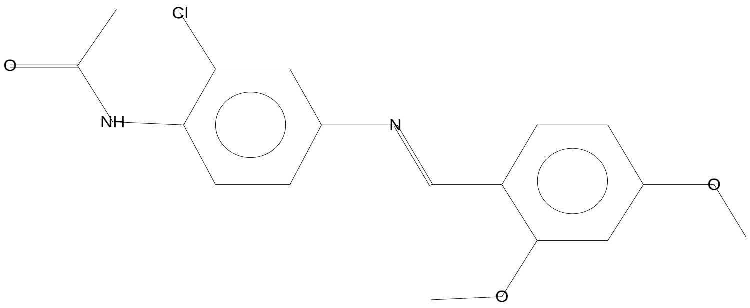

There is no signature data to report.

Library Search Report

RT	Probability	Compound Name	SI	Area %	Area	Molecular Weight	Molecular Formula	Library
44.79	11.93	à-N-Normethadol	400	0.24	763196.84	297	C20H27NO	mainlib
44.79	9.61	Ethane, 1,2-bis(2-methyl-5-nitrophenyl)-	395	0.24	763196.84	300	C16H16N2O4	mainlib
44.79	9.61	Ethane, 1,2-bis(1-methyl-4-nitrobenzen-2-yl)-	395	0.24	763196.84	300	C16H16N2O4	Wiley9

Faten-212 #11700 RT: 44.79 AV: 1 RF: 6.00, 3 NL: 9.53E3
F: {0,0} + c EI Full ms [40.00-800.00]

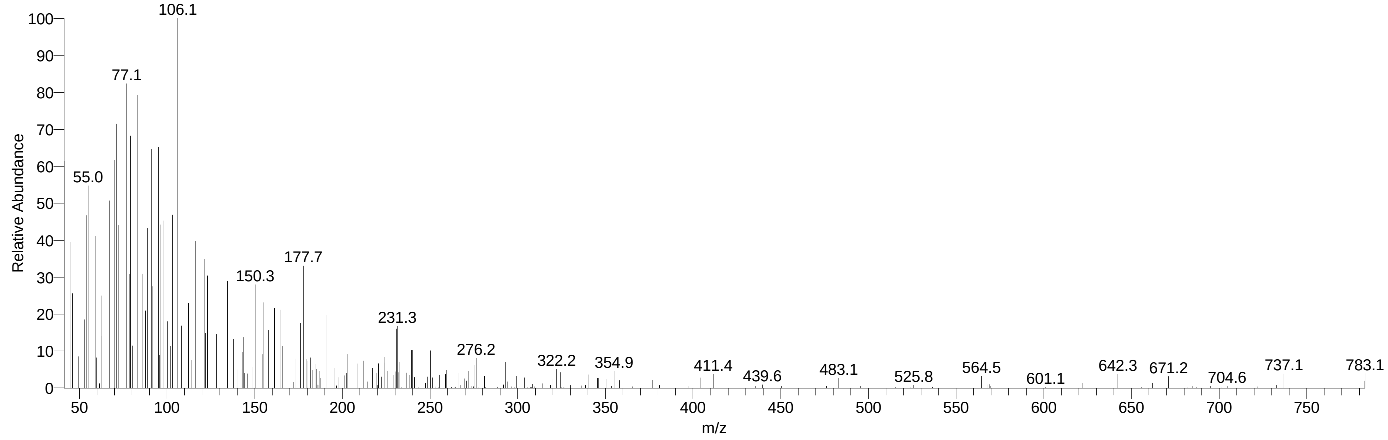

Hit Spectrum	Delta	Compound Structure

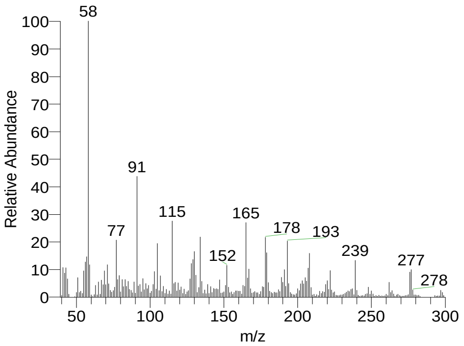

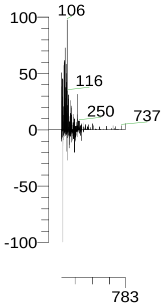

à-N-Normethadol
Formula C20H27NO, MW 297, CAS# 38455-85-5, Entry# 26410
6-(Methylamino)-4,4-diphenyl-3-heptanol #

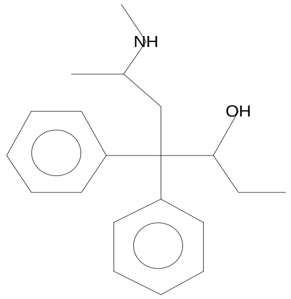

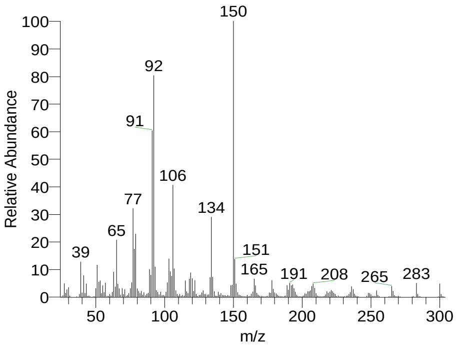

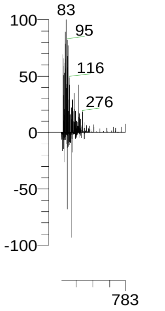

Ethane, 1,2-bis(2-methyl-5-nitrophenyl)-
Formula C16H16N2O4, MW 300, CAS# NA, Entry# 123991
1-Methyl-2-[2-(2-methyl-5-nitrophenyl)ethyl]-3-nitrobenzene #

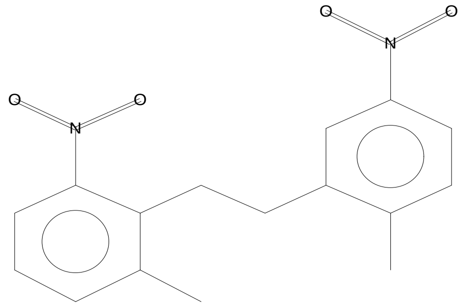

There is no signature data to report.

Library Search Report

Hit Spectrum	Delta	Compound Structure

Ethane, 1,2-bis(1-methyl-4-nitrobenzen-2-yl)-
Formula C16H16N2O4, MW 300, CAS# NA, Entry# 374127

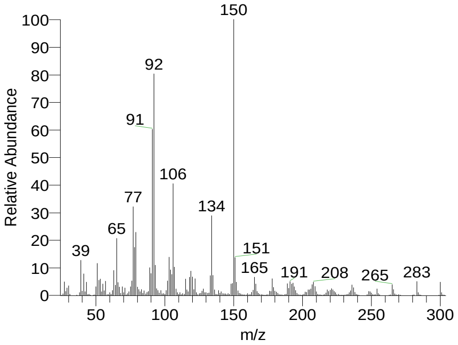

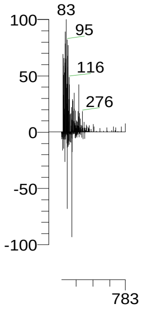

There is no signature data to report.

Library Search Report

RT	Probability	Compound Name	SI	Area %	Area	Molecular Weight	Molecular Formula	Library
44.85	22.82	10,13-Octadecadiynoic acid, methyl ester	504	0.20	634853.92	290	C19H30O2	mainlib
44.85	22.82	10,13-Octadecadiynoic acid, methyl ester (CAS)	504	0.20	634853.92	290	C19H30O2	Wiley9
44.85	6.46	Benzene, (1-methylhexadecyl)- (CAS)	475	0.20	634853.92	316	C23H40	Wiley9

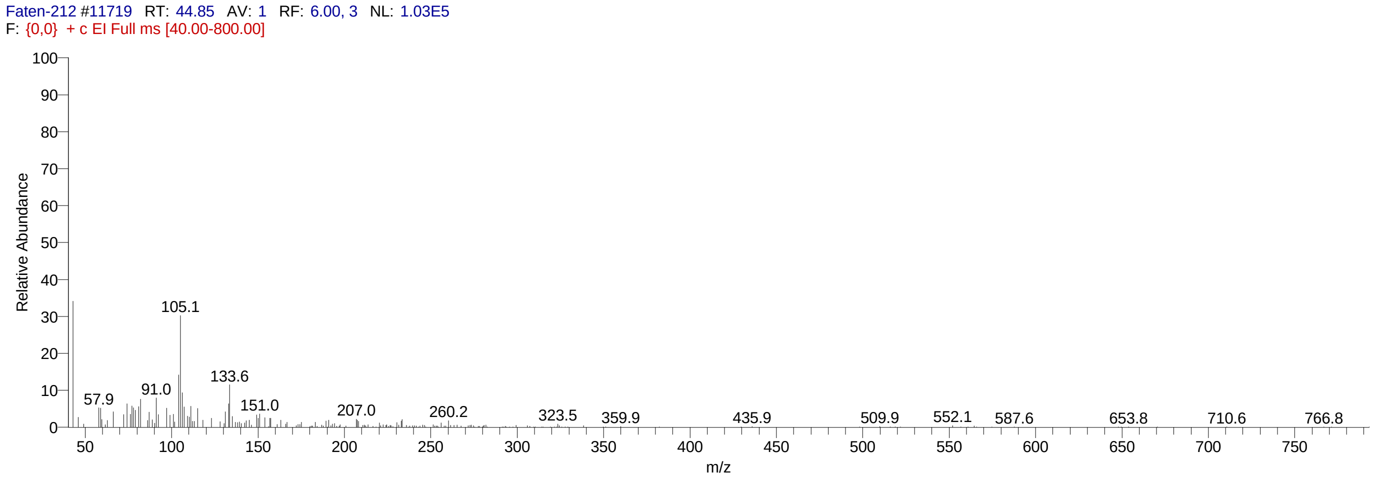

Hit Spectrum | Delta | Compound Structure

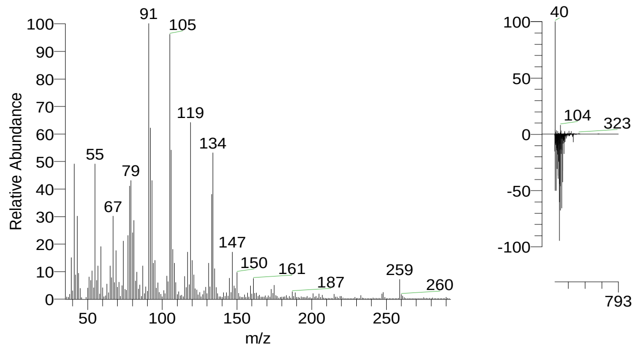

10,13-Octadecadiynoic acid, methyl ester
Formula C19H30O2, MW 290, CAS# 18202-24-9, Entry# 55439
Methyl 10,13-octadecadiynoate #

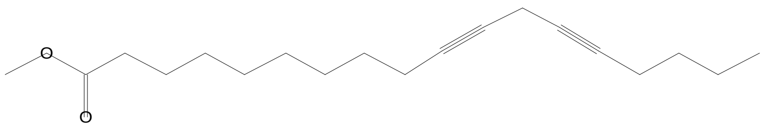

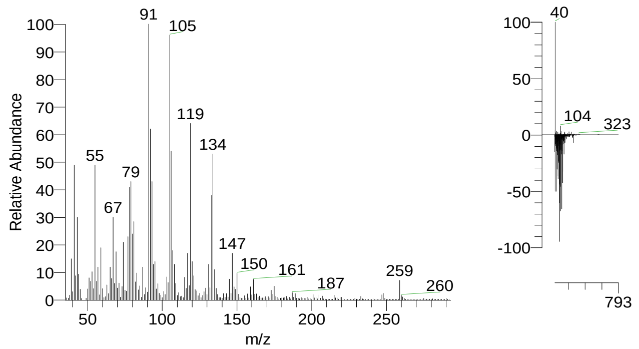

10,13-Octadecadiynoic acid, methyl ester (CAS)
Formula C19H30O2, MW 290, CAS# 18202-24-9, Entry# 352881
METHYL 10,13 OCTADECADIYNOATE

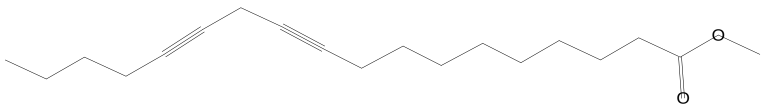

There is no signature data to report.

Library Search Report

Hit Spectrum	Delta	Compound Structure

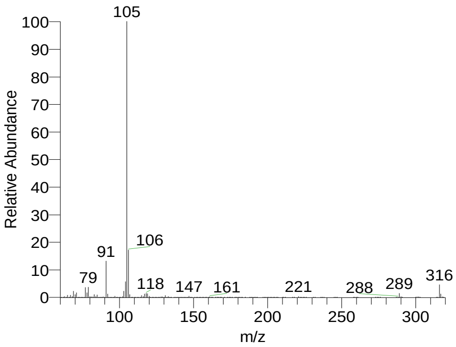

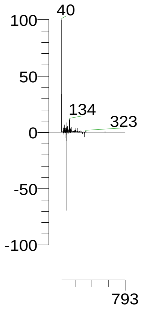

Benzene, (1-methylhexadecyl)- (CAS)
Formula C23H40, MW 316, CAS# 55125-25-2, Entry# 409814
2-PHENYLHEPTADECANE

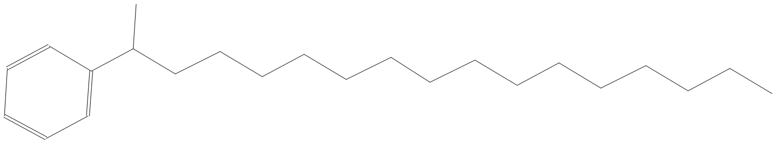

There is no signature data to report.

Library Search Report

RT	Probability	Compound Name	SI	Area %	Area	Molecular Weight	Molecular Formula	Library
45.06	20.90	TRANS-2-PHENYL-1,3-DIOXOLANE-4-METHYL OCTADEC-9,12,15-TRIENOATE	470	0.13	408709.49	440	C28H40O4	Wiley9
45.06	16.01	5-BENZOYL-4-(SELENAUREIDOMETHYL)THIADIAZOLE	463	0.13	408709.49	406	C11H11BrN4OSSe	Wiley9
45.06	4.88	PROPOXYPHENE METABOLITE 3	438	0.13	408709.49	325	C21H27NO2	Wiley9

Faten-212 #11781 RT: 45.06 AV: 1 RF: 6.00, 3 NL: 7.18E4
F: {0,0} + c EI Full ms [40.00-800.00]

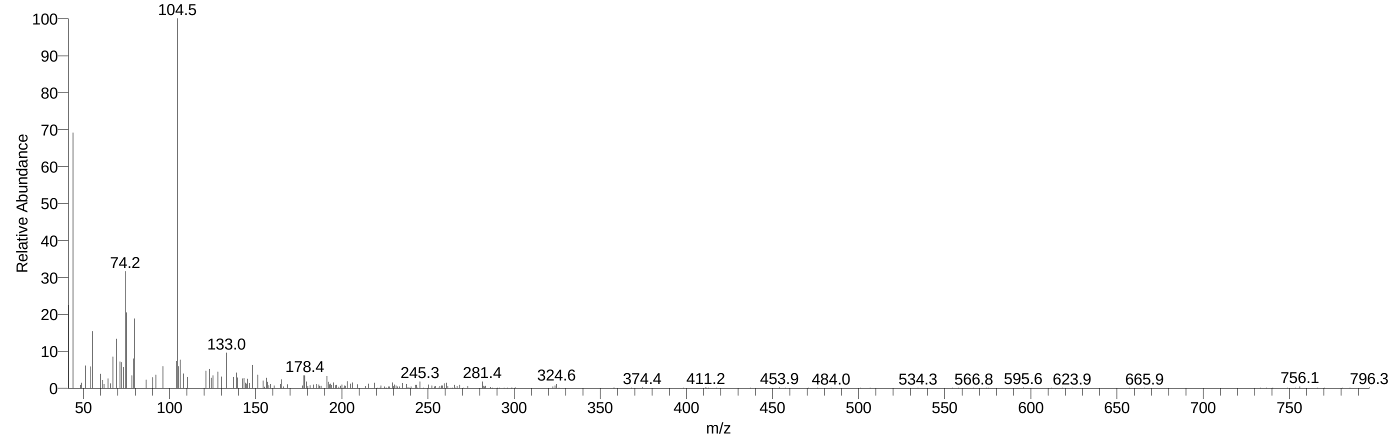

Hit Spectrum	Delta	Compound Structure

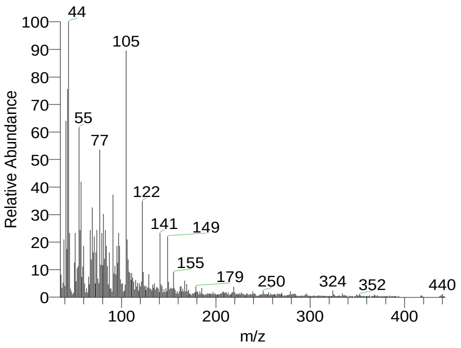

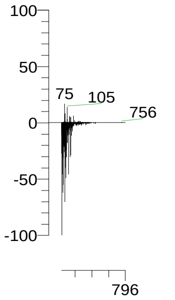

TRANS-2-PHENYL-1,3-DIOXOLANE-4-METHYL OCTADEC-9,12,15-TRIENOATE
Formula C28H40O4, MW 440, CAS# 56847-06-4, Entry# 581966
9,12,15-Octadecatrienoic acid, (2-phenyl-1,3-dioxolan-4-yl)methyl ester (CAS)

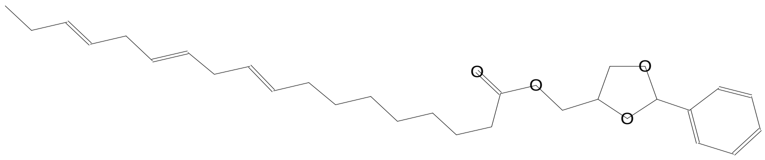

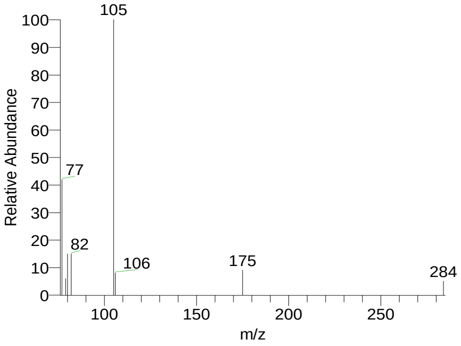

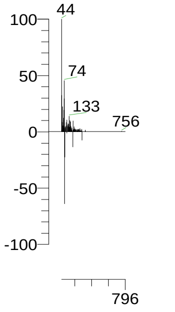

5-BENZOYL-4-(SELENAUREIDOMETHYL)THIADIAZOLE
Formula C11H11BrN4OSSe, MW 406, CAS# NA, Entry# 548642

There is no signature data to report.

Library Search Report

Hit Spectrum	Delta	Compound Structure

PROPOXYPHENE METABOLITE 3
Formula C21H27NO2, MW 325, CAS# NA, Entry# 426766

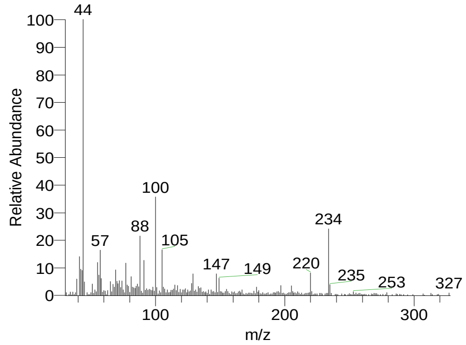

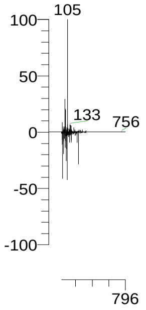

There is no signature data to report.

Library Search Report

RT	Probability	Compound Name	SI	Area %	Area	Molecular Weight	Molecular Formula	Library
45.58	9.50	1-[3-(4-Bromophenyl)-2-thioureido]-1-deoxy-b-d-glucopyranose 2,3,4,6-tetraacetate	432	0.16	506885.27	560	C21H25BrN2O9S	Wiley9
45.58	9.50	1-[3-(4-Bromophenyl)-2-thioureido]-1-deoxy-b-d-glucopyranose 2,3,4,6-tetraacetate	432	0.16	506885.27	560	C21H25BrN2O9S	mainlib
45.58	8.76	DI-2-BENZOTHIAZOLE DISULFANE	430	0.16	506885.27	332	C14H8N2S4	Wiley9

Faten-212 #11933 RT: 45.58 AV: 1 RF: 6.00, 3 NL: 1.00E5
F: {0,0} + c EI Full ms [40.00-800.00]

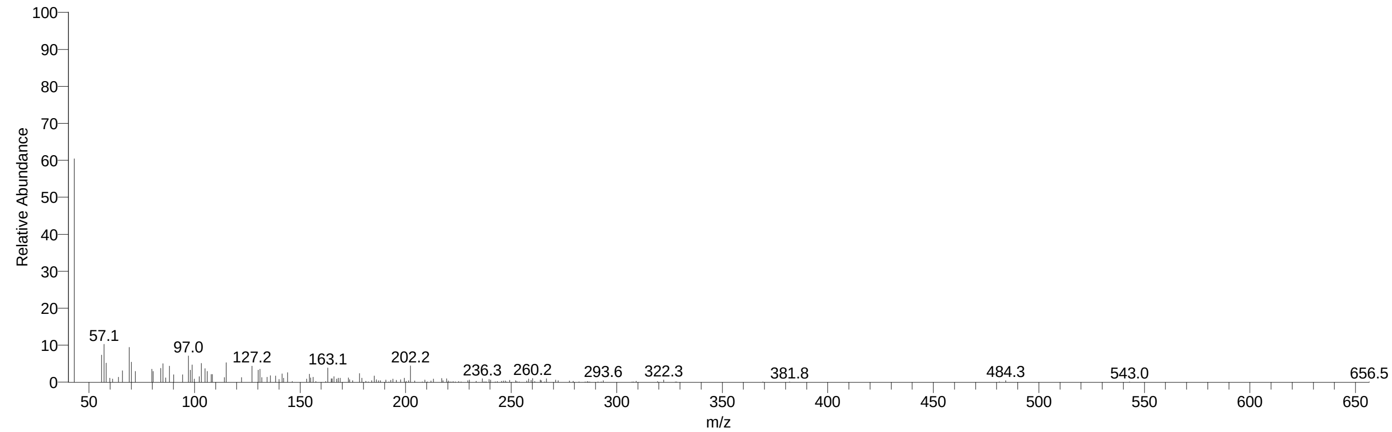

Hit Spectrum | Delta | Compound Structure

1-[3-(4-Bromophenyl)-2-thioureido]-1-deoxy-b-d-glucopyranose 2,3,4,6-tetraacetate
Formula C21H25BrN2O9S, MW 560, CAS# 94273-12-8, Entry# 636354
1-[3-(4-BROMOPHENYL)-2-THIOUREIDO]-1-DEOXY-B-D-GLUCOPYRANOSE 2,3,4,6-

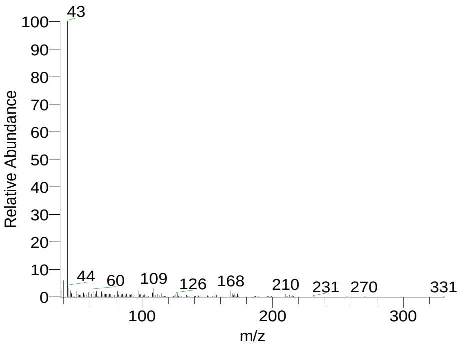

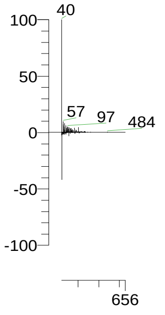

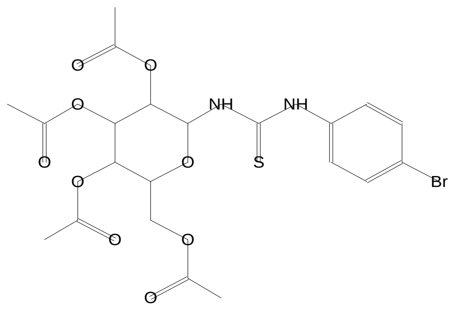

1-[3-(4-Bromophenyl)-2-thioureido]-1-deoxy-b-d-glucopyranose 2,3,4,6-tetraacetate
Formula C21H25BrN2O9S, MW 560, CAS# 94273-12-8, Entry# 5382
2,3,4,6-Tetra-O-acetyl-N-[(4-bromoanilino)carbothioyl]hexopyranosylamine #

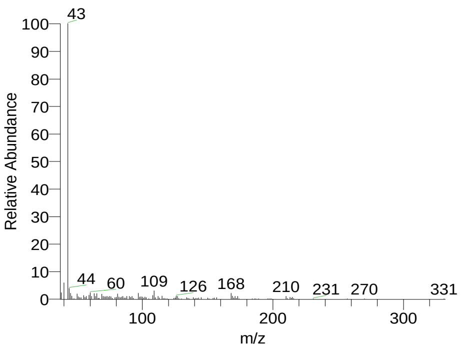

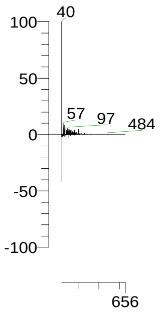

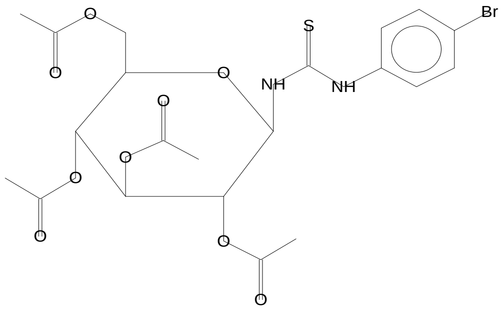

There is no signature data to report.

Library Search Report

Hit Spectrum	Delta	Compound Structure

DI-2-BENZOTHIAZOLE DISULFANE
Formula C14H8N2S4, MW 332, CAS# NA, Entry# 438926

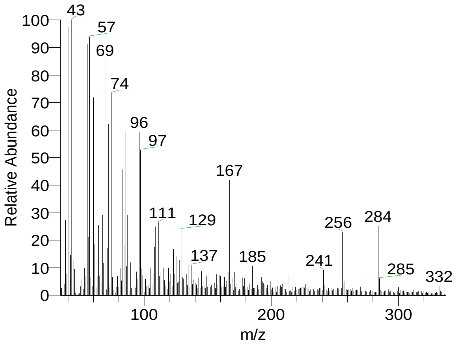

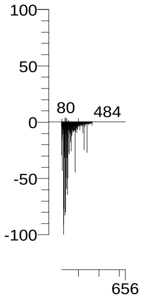

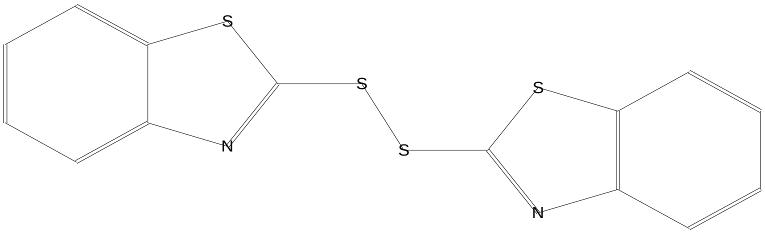

There is no signature data to report.

Library Search Report

RT	Probability	Compound Name	SI	Area %	Area	Molecular Weight	Molecular Formula	Library
45.73	18.34	Pentadecanoic acid, 14-methyl-, methyl ester (CAS)	407	0.19	608028.96	270	C17H34O2	Wiley9
45.73	17.63	DI-2-BENZOTHIAZOLE DISULFANE	406	0.19	608028.96	332	C14H8N2S4	Wiley9
45.73	5.90	Hexadecanoic acid, methyl ester (CAS)	382	0.19	608028.96	270	C17H34O2	Wiley9

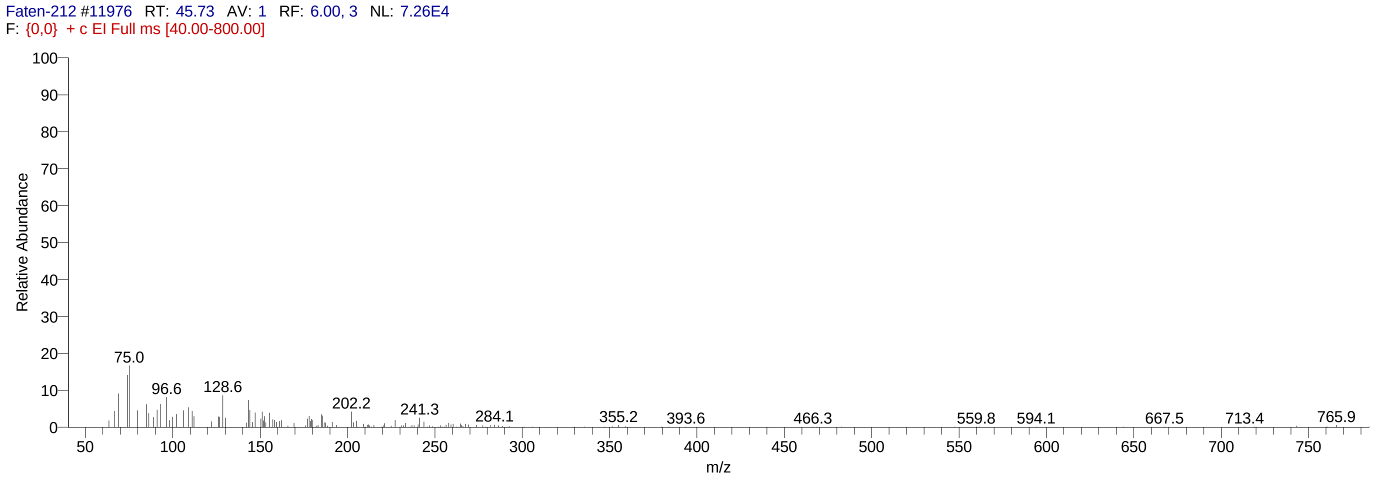

Hit Spectrum	Delta	Compound Structure

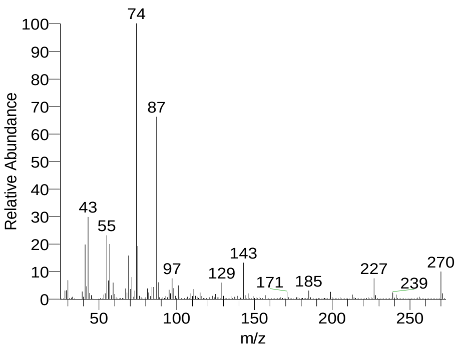

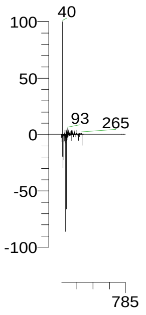

Pentadecanoic acid, 14-methyl-, methyl ester (CAS)
Formula C17H34O2, MW 270, CAS# 5129-60-2, Entry# 307599
METHYL 14-METHYL-PENTADECANOATE

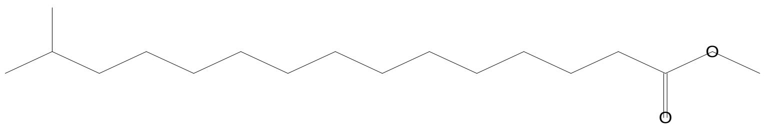

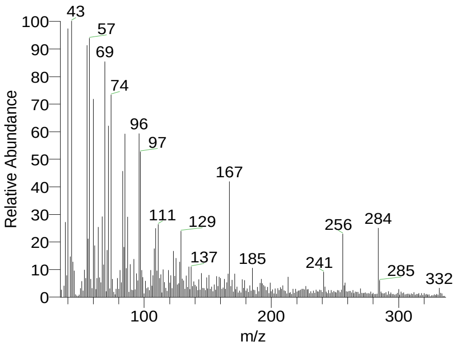

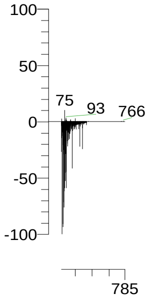

DI-2-BENZOTHIAZOLE DISULFANE
Formula C14H8N2S4, MW 332, CAS# NA, Entry# 438926

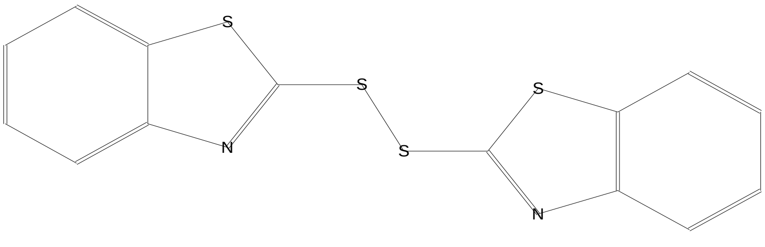

There is no signature data to report.

Library Search Report

Hit Spectrum | Delta | Compound Structure

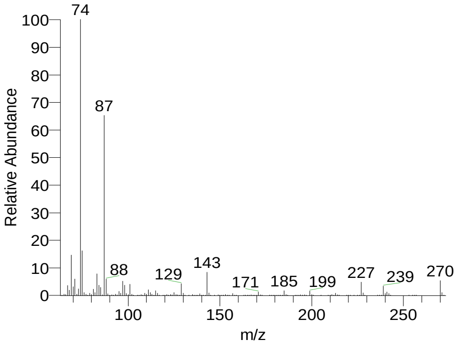

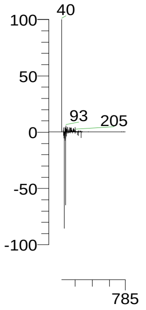

Hexadecanoic acid, methyl ester (CAS)
Formula C17H34O2, MW 270, CAS# 112-39-0, Entry# 307544
Methyl palmitate

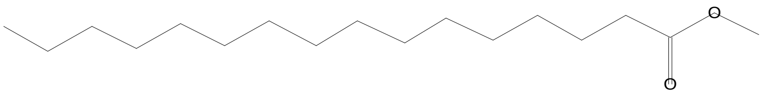

There is no signature data to report.

Library Search Report

RT	Probability	Compound Name	SI	Area %	Area	Molecular Weight	Molecular Formula	Library
45.99	24.45	Tetraacetyl-d-xylonic nitrile	476	0.28	898232.01	343	C14H17NO9	mainlib
45.99	5.48	Imidazole, 2-amino-5-[(2-carboxy)vinyl]-	434	0.28	898232.01	153	C6H7N3O2	mainlib
45.99	3.43	Actinobolin	420	0.28	898232.01	300	C13H20N2O6	mainlib

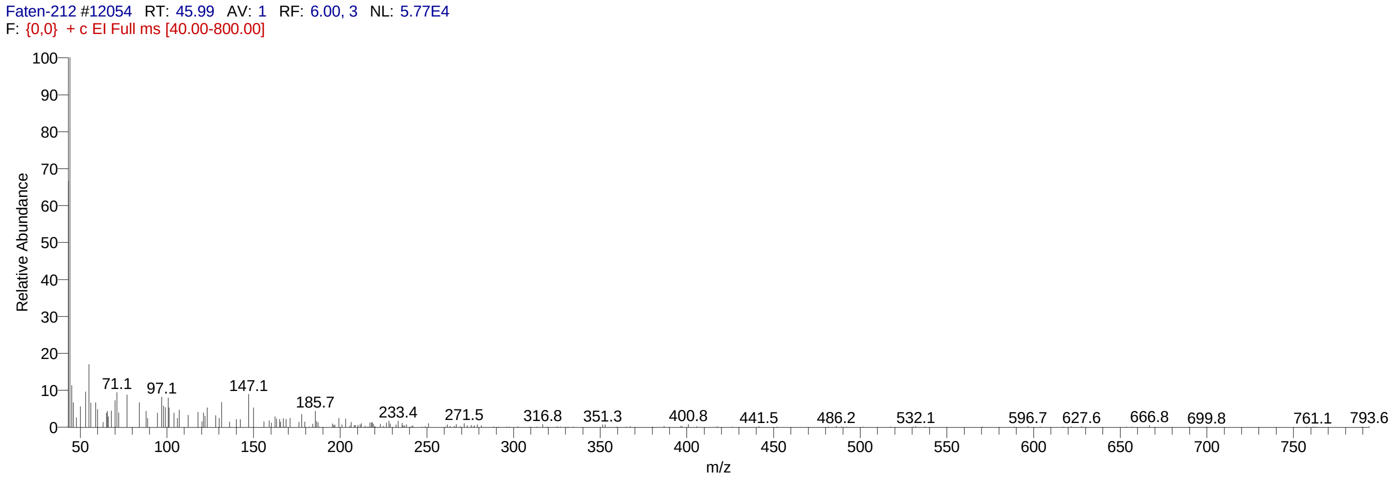

Hit Spectrum	Delta	Compound Structure

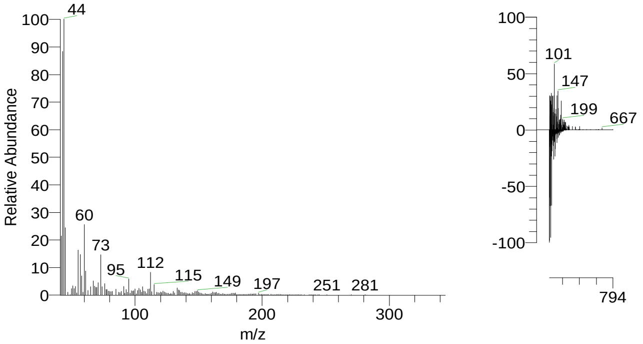

Tetraacetyl-d-xylonic nitrile
Formula C14H17NO9, MW 343, CAS# NA, Entry# 14445

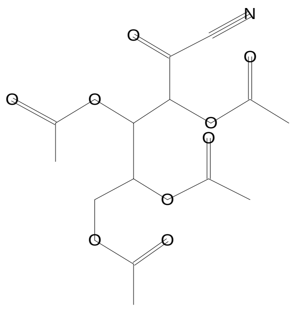

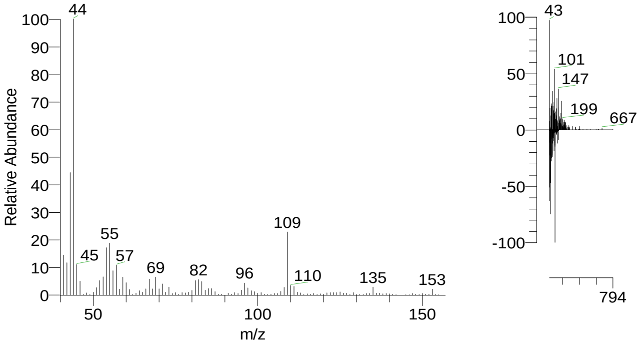

Imidazole, 2-amino-5-[(2-carboxy)vinyl]-
Formula C6H7N3O2, MW 153, CAS# NA, Entry# 14473
(2E)-3-(2-Amino-1H-imidazol-5-yl)-2-propenoic acid #

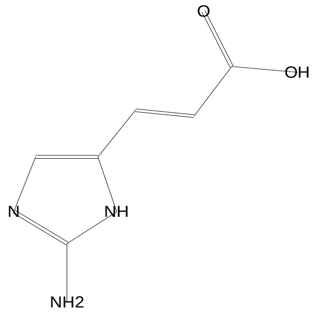

There is no signature data to report.

Library Search Report

Hit Spectrum	Delta	Compound Structure

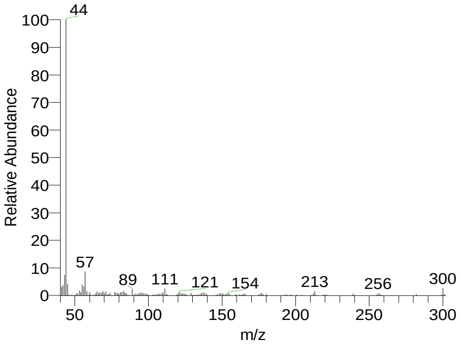

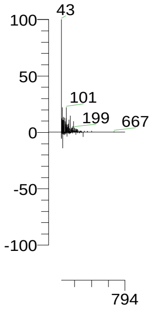

Actinobolin
Formula C13H20N2O6, MW 300, CAS# 24397-89-5, Entry# 14620
Isocoumarin, 4-(2-aminopropionamido)-3,4,4a,5,6,7-hexahydro-5,6,8-trihydroxy-3-methyl-

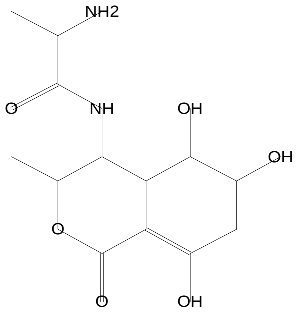

There is no signature data to report.

Library Search Report

RT	Probability	Compound Name	SI	Area %	Area	Molecular Weight	Molecular Formula	Library
46.05	7.92	á-N-Acetylneuraminic acid, methyl ester-2-methyl-7,9-methyl-boronate-3,8-di(trimethylsilyl)-	422	0.27	868960.82	505	C20H40BNO9Si2	mainlib
46.05	7.92	B-N-ACETYLNEURAMINIC ME ESTER-2-ME-7,9-ME-BORONATE-3,8-DITMS	422	0.27	868960.82	505	C20H40BNO9Si2	Wiley9
46.05	5.91	Pyrazole[4,5-b]imidazole, 1-formyl-3-ethyl-6-á-d-ribofuranosyl-	414	0.27	868960.82	296	C12H16N4O5	mainlib

Faten-212 #12070 RT: 46.05 AV: 1 RF: 6.00, 3 NL: 8.62E4
F: {0,0} + c EI Full ms [40.00-800.00]

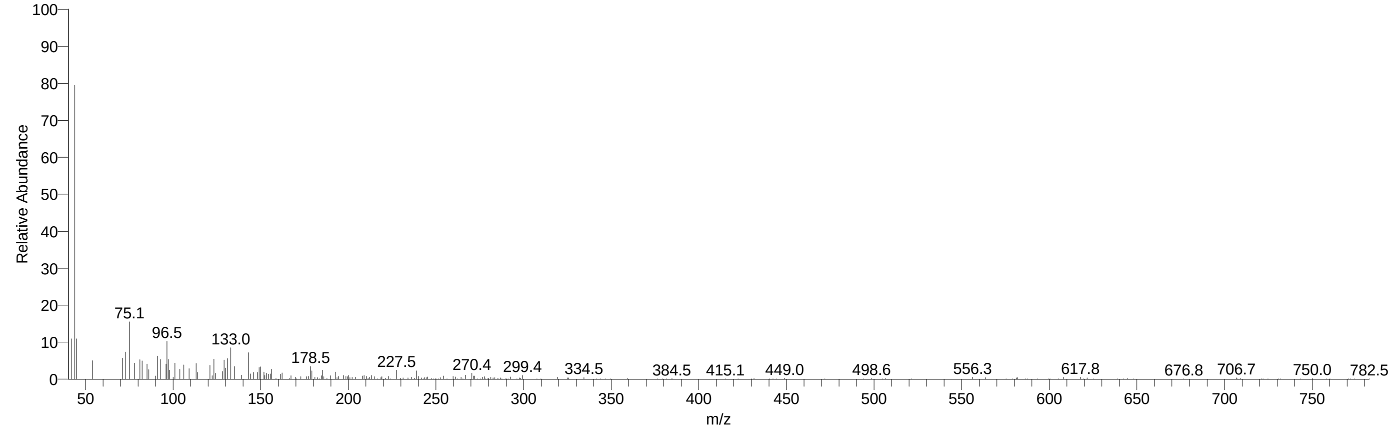

Hit Spectrum	Delta	Compound Structure

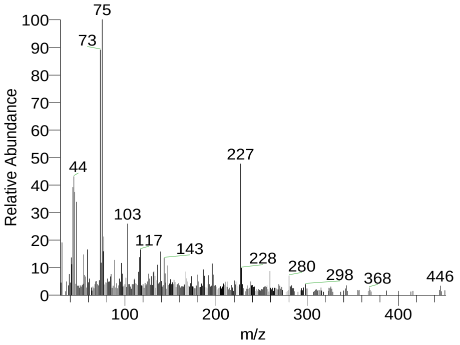

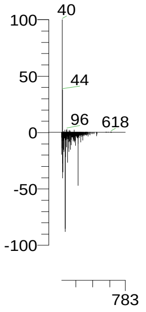

á-N-Acetylneuraminic acid, methyl ester-2-methyl-7,9-methyl-boronate-3,8-di(trimethylsilyl)-
Formula C20H40BNO9Si2, MW 505, CAS# NA, Entry# 41311

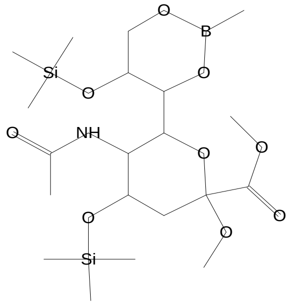

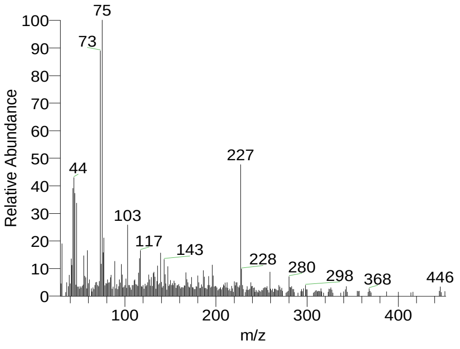

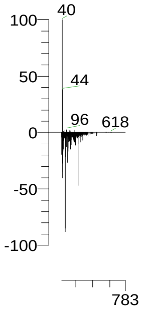

B-N-ACETYLNEURAMINIC ME ESTER-2-ME-7,9-ME-BORONATE-3,8-DITMS
Formula C20H40BNO9Si2, MW 505, CAS# NA, Entry# 619810

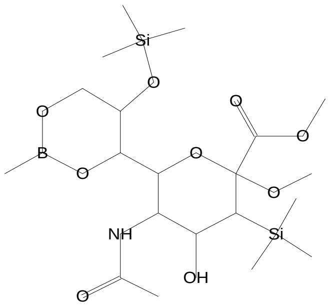

There is no signature data to report.

Library Search Report

Hit Spectrum	Delta	Compound Structure

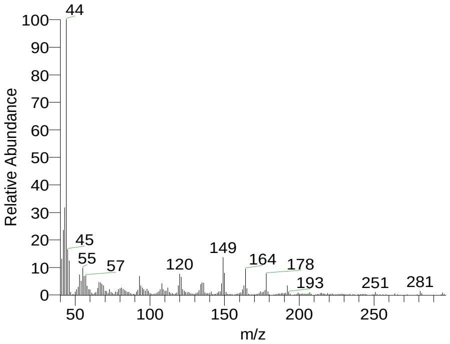

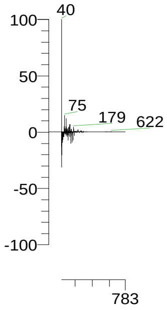

Pyrazole[4,5-b]imidazole, 1-formyl-3-ethyl-6-á-d-ribofuranosyl-
Formula C12H16N4O5, MW 296, CAS# NA, Entry# 14403

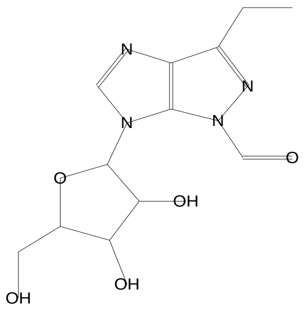

There is no signature data to report.

Library Search Report

RT	Probability	Compound Name	SI	Area %	Area	Molecular Weight	Molecular Formula	Library
46.22	8.26	1-Monolinoleoylglycerol trimethylsilyl ether	448	0.21	676866.36	498	C27H54O4Si2	mainlib
46.22	8.26	9,12-Octadecadienoic acid (Z,Z)-, 2,3-bis[(trimethylsilyl)oxy]propyl ester (CAS)	446	0.21	676866.36	498	C27H54O4Si2	Wiley9
46.22	5.50	5,8-Dimethoxy-2(1H)-quinolinethione	436	0.21	676866.36	221	C11H11NO2S	Wiley9

Faten-212 #12122 RT: 46.22 AV: 1 RF: 6.00, 3 NL: 1.45E4
F: {0,0} + c EI Full ms [40.00-800.00]

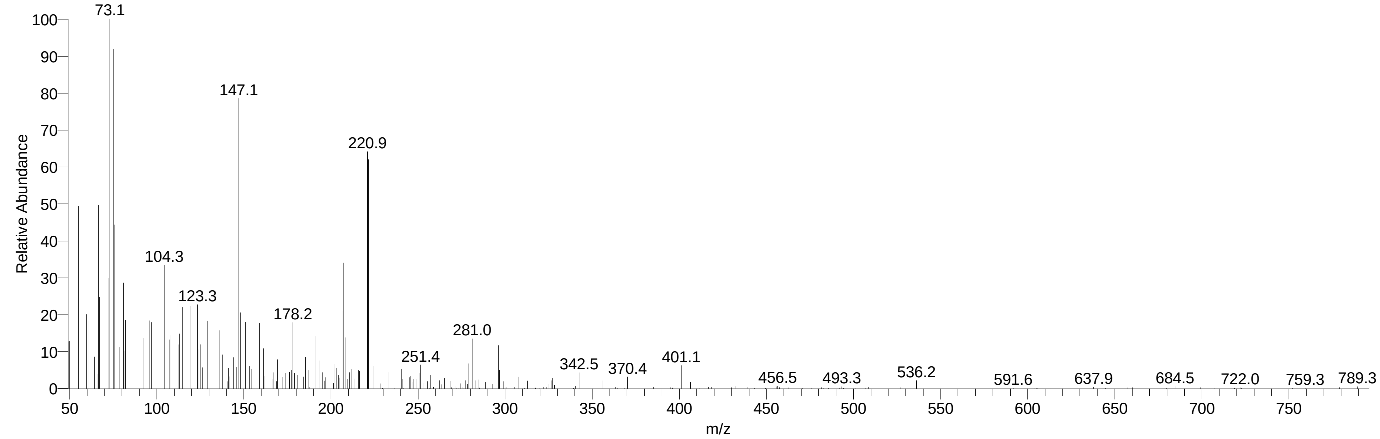

Hit Spectrum | Delta | Compound Structure

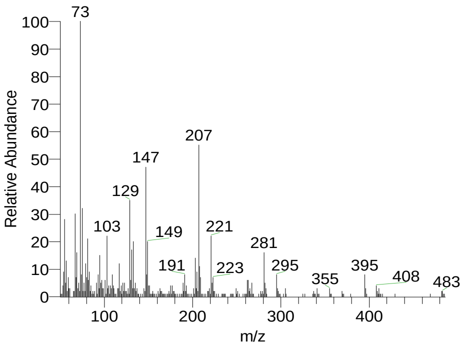

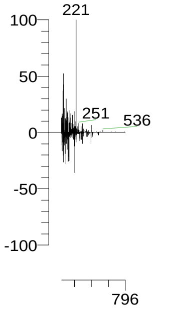

1-Monolinoleoylglycerol trimethylsilyl ether
Formula C27H54O4Si2, MW 498, CAS# 54284-45-6, Entry# 39425
9,12-Octadecadienoic acid (Z,Z)-, 2,3-bis[(trimethylsilyl)oxy]propyl ester

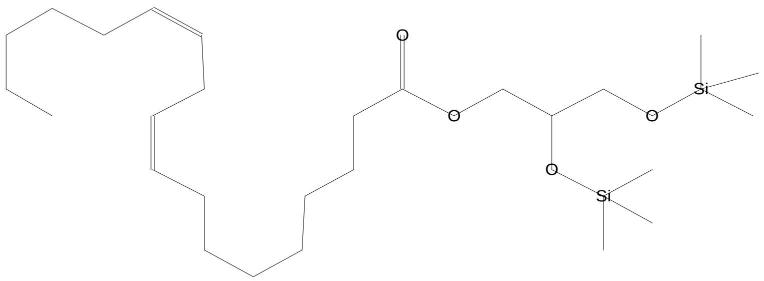

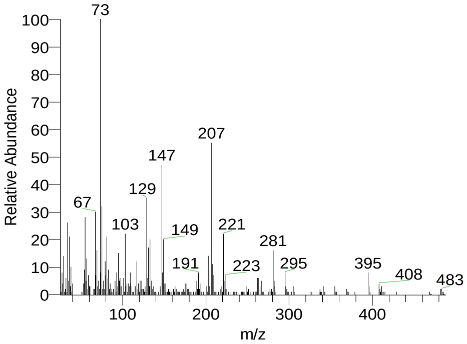

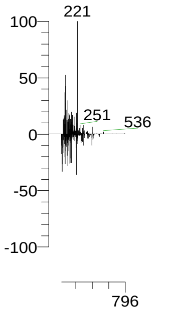

9,12-Octadecadienoic acid (Z,Z)-, 2,3-bis[(trimethylsilyl)oxy]propyl ester (CAS)
Formula C27H54O4Si2, MW 498, CAS# 54284-45-6, Entry# 616958
TRIMETHYLSILYLETHER DERIVATIVE OF 1-MONOLINOLEIN

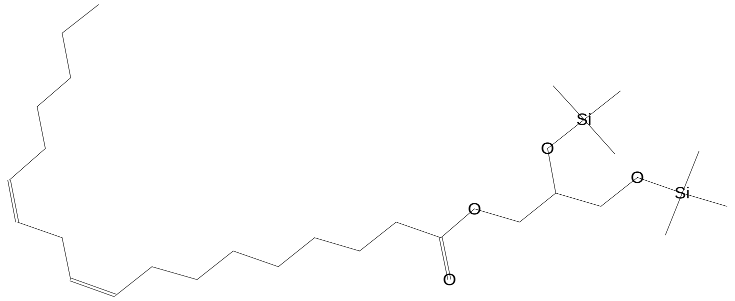

There is no signature data to report.

Library Search Report

Hit Spectrum	Delta	Compound Structure

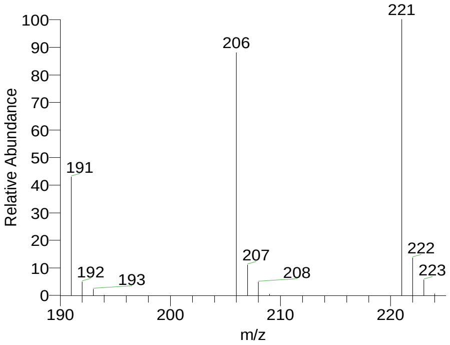

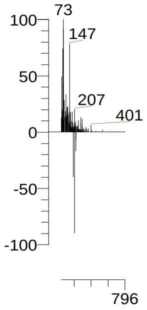

5,8-Dimethoxy-2(1H)-quinolinethione
Formula C11H11NO2S, MW 221, CAS# NA, Entry# 189968

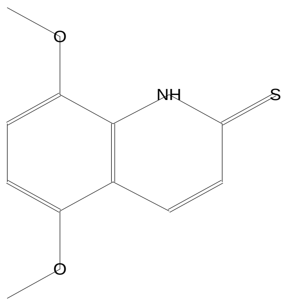

There is no signature data to report.

Library Search Report

RT	Probability	Compound Name	SI	Area %	Area	Molecular Weight	Molecular Formula	Library
46.53	21.11	á-D-Galactopyranoside, methyl 2,6-bis-O-(trimethylsilyl)-, cyclic butylboronate (CAS)	501	0.13	417450.47	404	C17H37BO6Si2	Wiley9
46.53	10.88	à-D-Glucopyranoside, methyl 2-(acetylamino)-2-deoxy-3-O-(trimethylsilyl)-, cyclic methylboronate (CAS)	483	0.13	417450.47	331	C13H26BNO6Si	Wiley9
46.53	10.88	à-D-Glucopyranoside, methyl 2-(acetylamino)-2-deoxy-3-O-(trimethylsilyl)-, cyclic methylboronate	480	0.13	417450.47	331	C13H26BNO6Si	mainlib

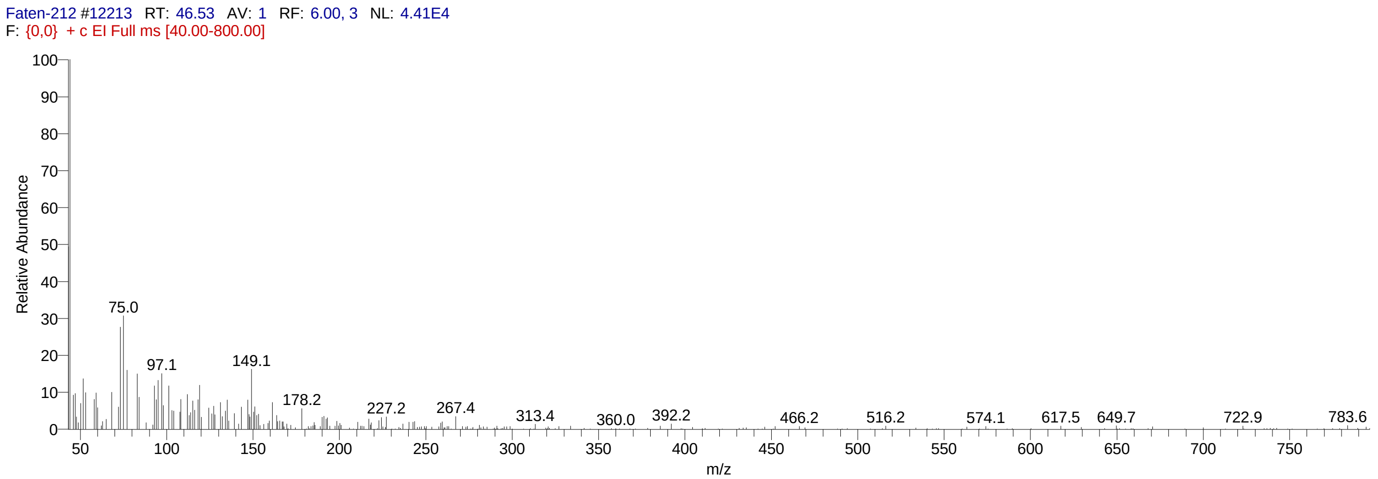

Hit Spectrum	Delta	Compound Structure

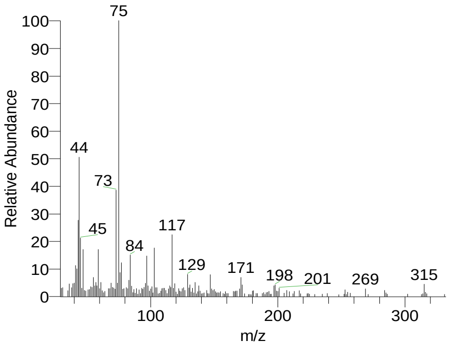

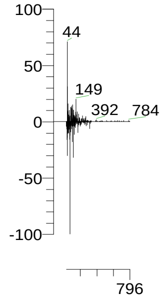

á-D-Galactopyranoside, methyl 2,6-bis-O-(trimethylsilyl)-, cyclic butylboronate (CAS)
Formula C17H37BO6Si2, MW 404, CAS# 56211-13-3, Entry# 546601
B-GALACTOPYRANOSIDE-1-METHYL-3,4-BUTYLBORONATE-2,6-DITMS

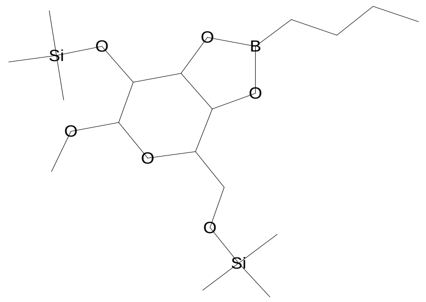

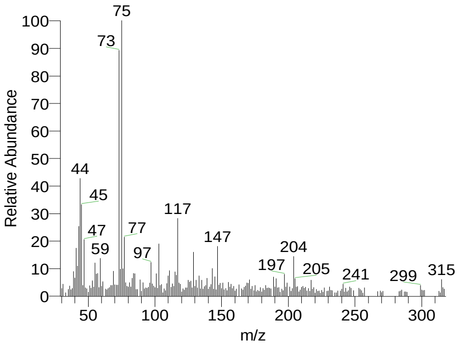

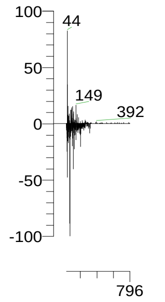

Formula C13H26BNO6Si, MW 331, CAS# 54477-01-9, Entry# 437410
2-ACETAMIDO-A-GLUCOPYRANOSIDE-1-METHYL-4,6-METHYLBORONATE-3-TMS

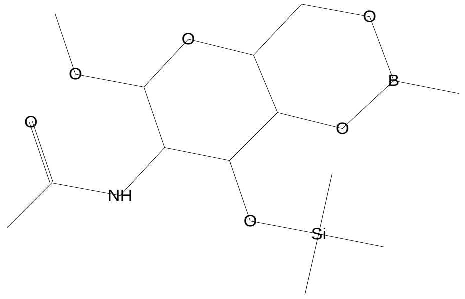

There is no signature data to report.

Library Search Report

Hit Spectrum

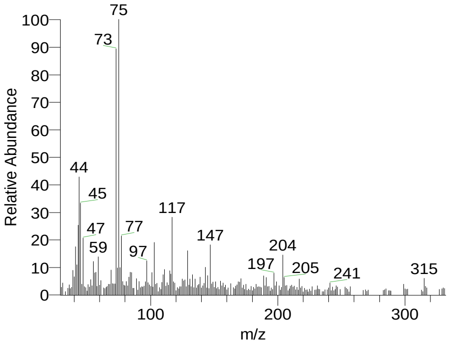

Delta

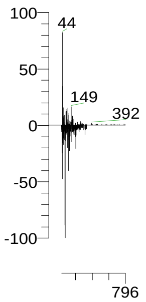

Compound Structure

à-D-Glucopyranoside, methyl 2-(acetylamino)-2-deoxy-3-O-(trimethylsilyl)-, cyclic methylboronate
Formula C13H26BNO6Si, MW 331, CAS# 54477-01-9, Entry# 41200

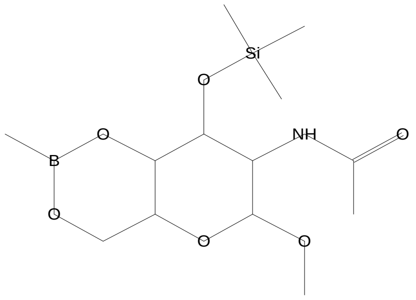

There is no signature data to report.

Library Search Report

RT	Probability	Compound Name	SI	Area %	Area	Molecular Weight	Molecular Formula	Library
47.59	12.28	TRIDEUTERIOMETHYL 10-EPOXY-7-ETHYL-3,11-DIMETHYLTRIDECA-2,6-DIENOATE	434	0.13	411307.01	294	C18H27D3O3	Wiley9
47.59	5.96	Cyclopenta[a,d]cyclooctten-5-one, 1,2,3,3a,4,5,6,8,9,9a,10,10a-dodecahydro-7-(1-methylethyl)-1,9a-dimethyl-4-methylene	415	0.13	411307.01	286	C20H30O	mainlib
47.59	5.73	Cyclopenta[a,d]cyclooctten-5-one, 1,2,3,3a,4,5,6,8,9,9a,10,10a-dodecahydro-7-(1-methylethyl)-1,9a-dimethyl-4-methylene	414	0.13	411307.01	286	C20H30O	Wiley9

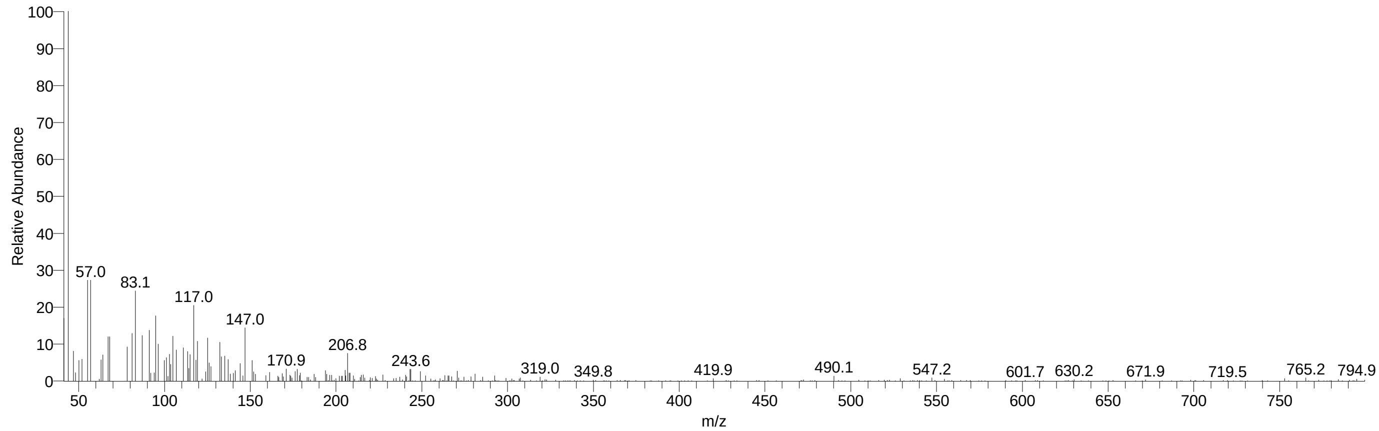

Hit Spectrum | Delta | Compound Structure

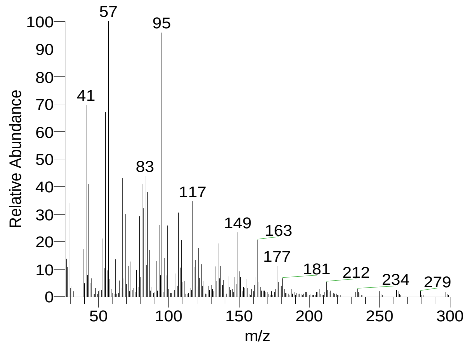

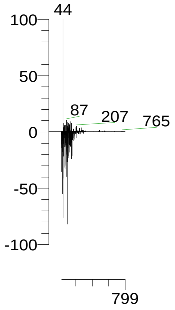

TRIDEUTERIOMETHYL 10-EPOXY-7-ETHYL-3,11-DIMETHYLTRIDECA-2,6-DIENOATE
Formula C18H27D3O3, MW 294, CAS# 56805-11-9, Entry# 361576

There is no signature data to report.

Library Search Report

Hit Spectrum | Delta | Compound Structure

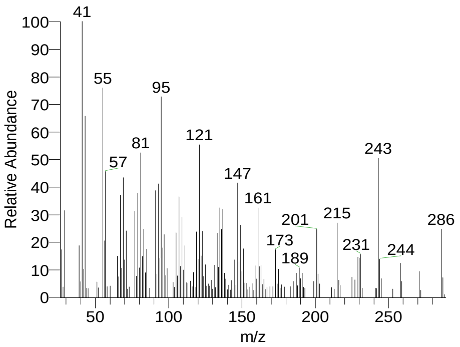

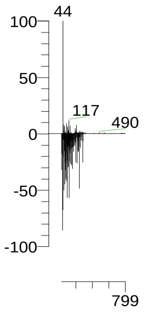

Formula C20H30O, MW 286, CAS# NA, Entry# 2716

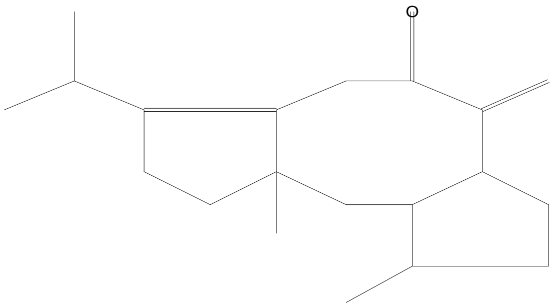

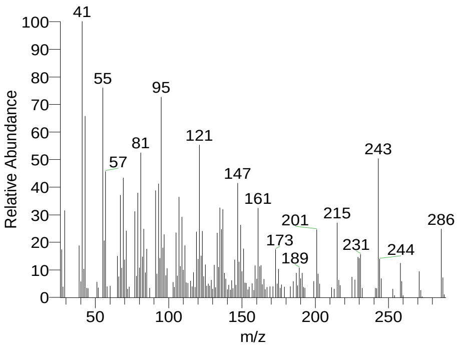

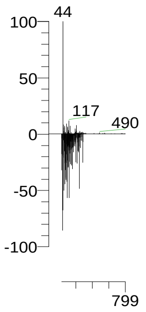

Formula C20H30O, MW 286, CAS# NA, Entry# 344639

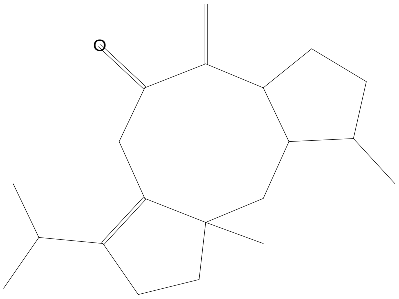

There is no signature data to report.

Library Search Report

RT	Probability	Compound Name	SI	Area %	Area	Molecular Weight	Molecular Formula	Library
48.27	12.62	Glycine, N-[(3à,5á,7à,12à)-24-oxo-3,7,12-tris[(trimethylsilyl)oxy]cholan-24-yl]-, methyl ester	414	0.13	403920.72	695	C36H69NO6Si3	mainlib
48.27	12.62	GLYCOCHOLIC ACID METHYL ESTER TMS	413	0.13	403920.72	695	C36H69NO6Si3	Wiley9
48.27	9.92	QUERCETIN 7,3',4'-TRIMETHOXY	408	0.13	403920.72	344	C18H16O7	Wiley9

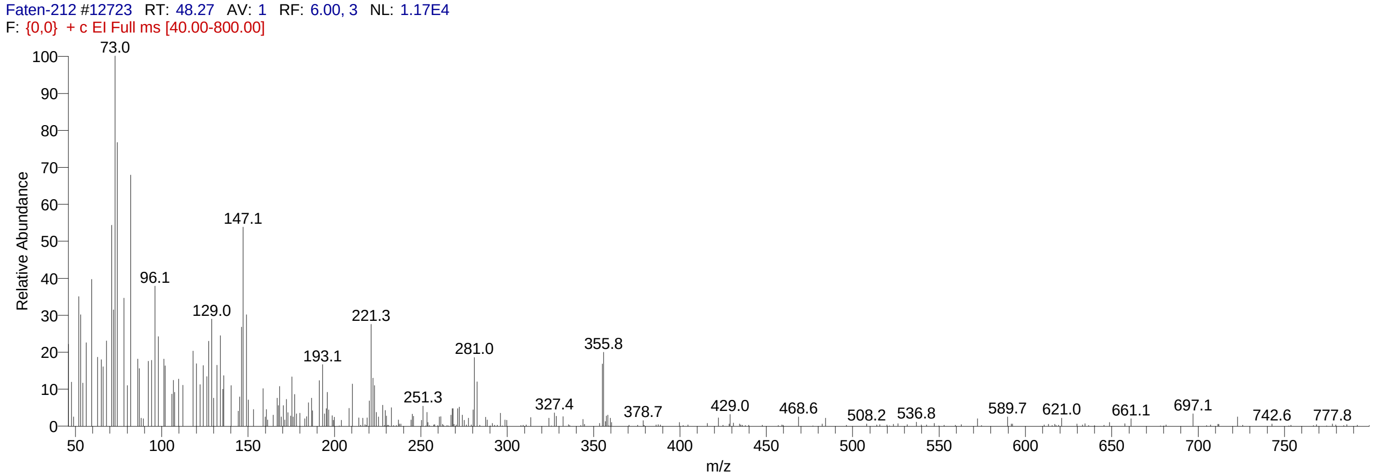

Hit Spectrum	Delta	Compound Structure

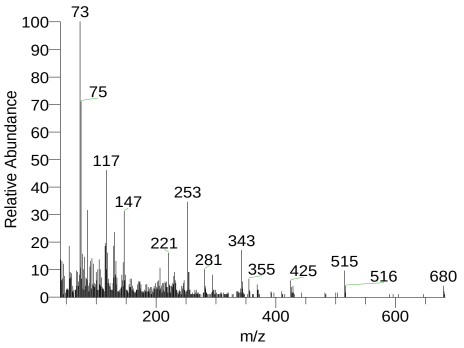

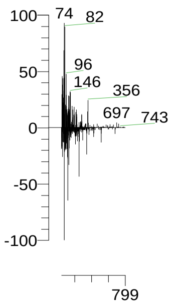

Glycine, N-[(3à,5á,7à,12à)-24-oxo-3,7,12-tris[(trimethylsilyl)oxy]cholan-24-yl]-, methyl ester
Formula C36H69NO6Si3, MW 695, CAS# 57326-16-6, Entry# 37701
Methyl ((24-oxo-3,7,12-tris[(trimethylsilyl)oxy]cholan-24-yl)amino)acetate #

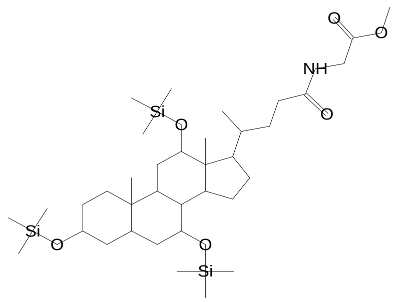

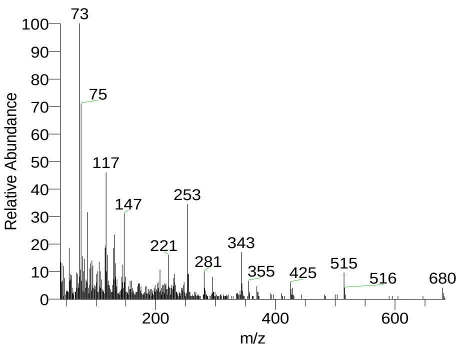

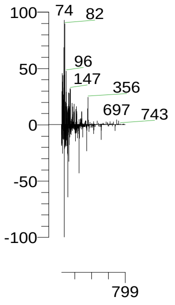

GLYCOCHOLIC ACID METHYL ESTER TMS
Formula C36H69NO6Si3, MW 695, CAS# 57326-16-6, Entry# 654329
Glycine, N-[(3à,5á,7à,12à)-24-oxo-3,7,12-tris[(trimethylsilyl)oxy]cholan-24-yl]-, methyl ester (CAS)

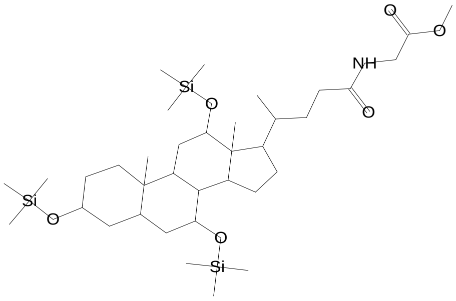

There is no signature data to report.

Library Search Report

Hit Spectrum	Delta	Compound Structure

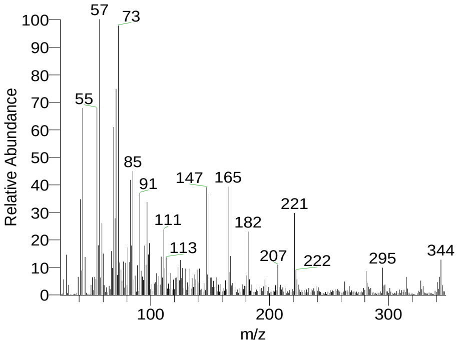

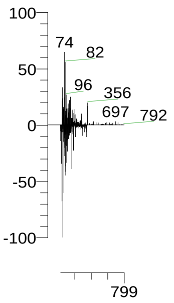

QUERCETIN 7,3',4'-TRIMETHOXY
Formula C18H16O7, MW 344, CAS# 6068-80-0, Entry# 461110
4H-1-Benzopyran-4-one, 2-(3,4-dimethoxyphenyl)-3,5-dihydroxy-7-methoxy- (CAS)

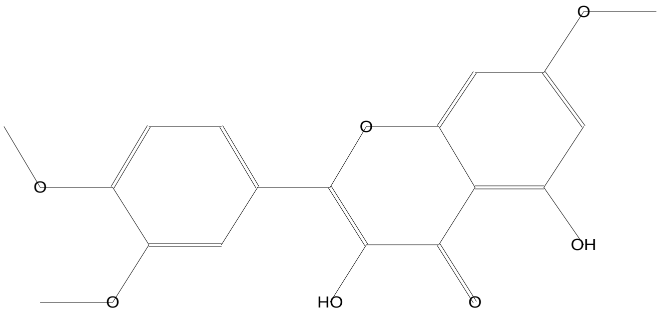

There is no signature data to report.

Library Search Report

RT	Probability	Compound Name	SI	Area %	Area	Molecular Weight	Molecular Formula	Library
50.15	7.89	Heptanoic acid, docosyl ester	380	0.16	495232.94	438	C29H58O2	mainlib
50.15	7.89	Heptanoic acid, docosyl ester (CAS)	380	0.16	495232.94	438	C29H58O2	Wiley9
50.15	7.89	á-N-Acetylneuraminic acid, methyl ester-2-methyl-7,9-methyl-boronate-3,8-di(trimethylsilyl)-	380	0.16	495232.94	505	C20H40BNO9Si2	mainlib

Faten-212 #13275 RT: 50.15 AV: 1 RF: 6.00, 3 NL: 4.66E4
F: {0,0} + c EI Full ms [40.00-800.00]

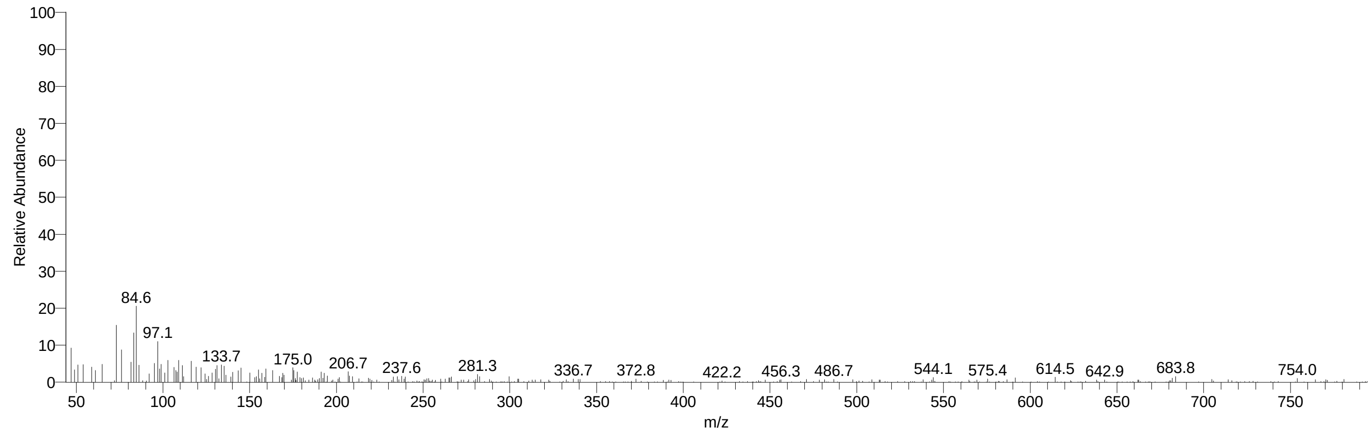

Hit Spectrum	Delta	Compound Structure

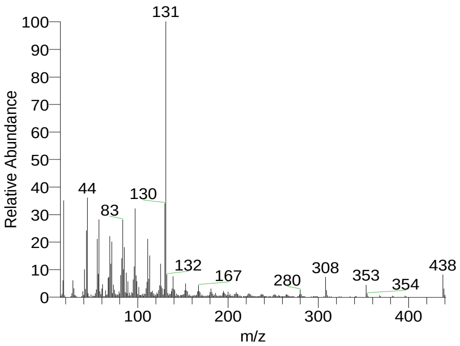

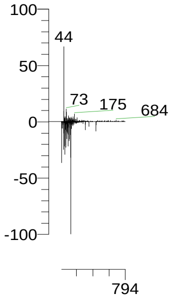

Heptanoic acid, docosyl ester
Formula C29H58O2, MW 438, CAS# 55320-07-5, Entry# 102870
Docosyl heptanoate #

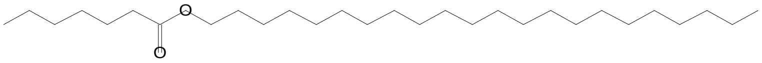

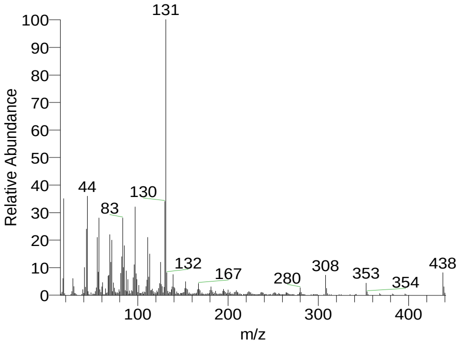

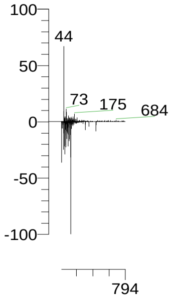

Heptanoic acid, docosyl ester (CAS)
Formula C29H58O2, MW 438, CAS# 55320-07-5, Entry# 580507
N-DOCOSYL N-HEPTANOATE

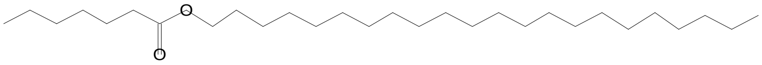

There is no signature data to report.

Library Search Report

Hit Spectrum	Delta	Compound Structure

á-N-Acetylneuraminic acid, methyl ester-2-methyl-7,9-methyl-boronate-3,8-di(trimethylsilyl)-
Formula C20H40BNO9Si2, MW 505, CAS# NA, Entry# 41311

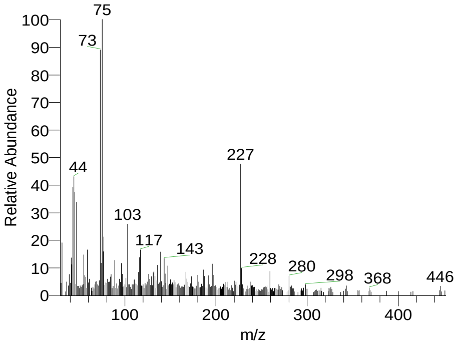

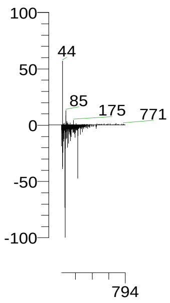

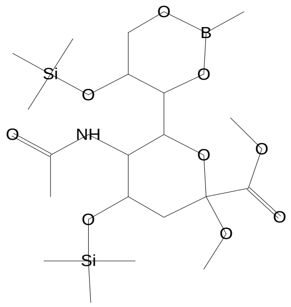

There is no signature data to report.

Library Search Report

RT	Probability	Compound Name	SI	Area %	Area	Molecular Weight	Molecular Formula	Library
50.24	13.52	à-D-Mannopyranoside, methyl, cyclic 2,3:4,6-bis(methylboronate) (CAS)	378	0.16	505098.79	242	C9H16B2O6	Wiley9
50.24	12.99	à-D-Galactopyranoside, methyl 2,3-bis-O-(trimethylsilyl)-, cyclic methylboronate (CAS)	377	0.16	505098.79	362	C14H31BO6Si2	Wiley9
50.24	8.91	DEMYCAROSYLTURIMYCIN H	366	0.16	505098.79	842	C43H74N2O14	Wiley9

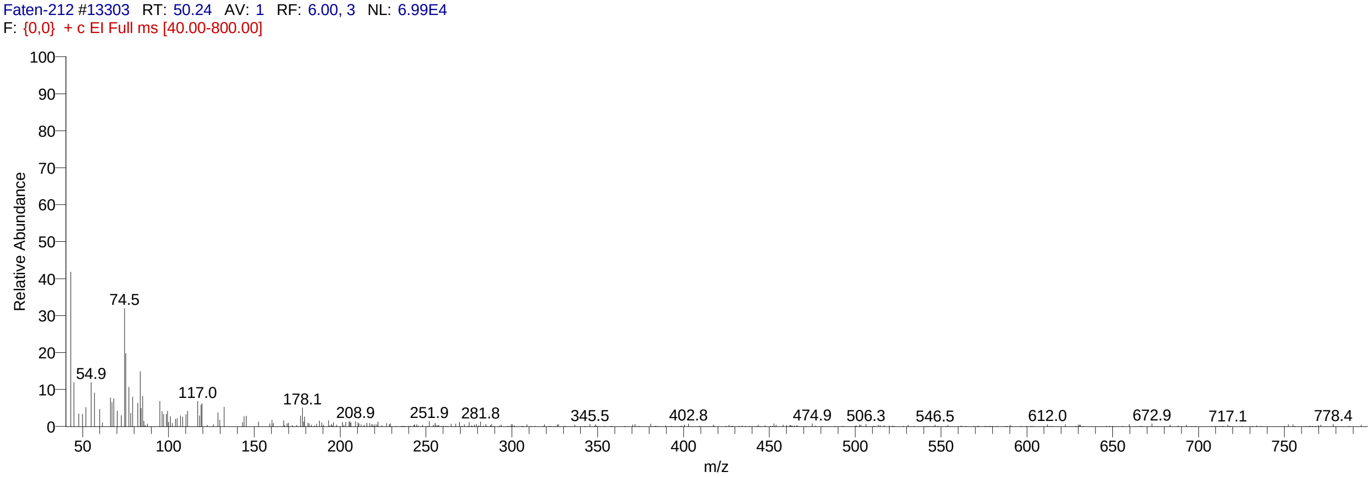

Hit Spectrum | Delta | Compound Structure

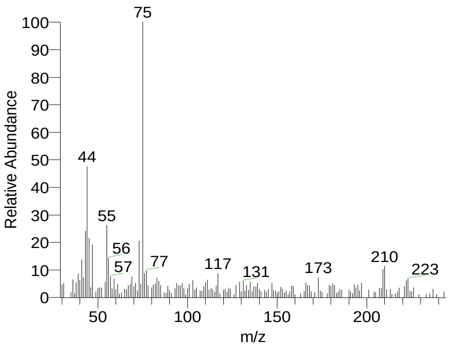

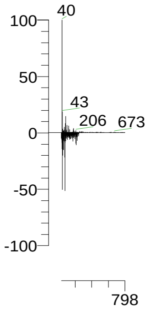

à-D-Mannopyranoside, methyl, cyclic 2,3:4,6-bis(methylboronate) (CAS)
Formula C9H16B2O6, MW 242, CAS# 54400-85-0, Entry# 239606
A-MANNOPYRANOSIDE-1-METHYL-2,3-4,6-DI-METHYLBORONATE

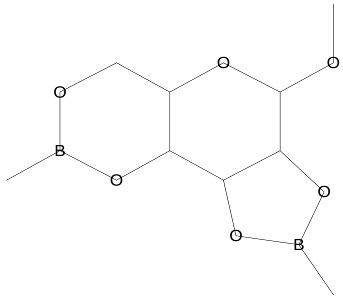

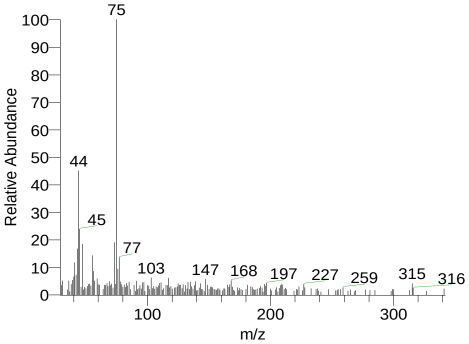

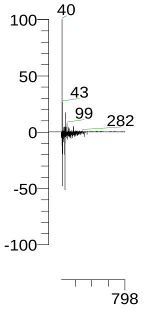

à-D-Galactopyranoside, methyl 2,3-bis-O-(trimethylsilyl)-, cyclic methylboronate (CAS)
Formula C14H31BO6Si2, MW 362, CAS# 54400-88-3, Entry# 490296
A-GALACTOPYRANOSIDE-1-METHYL-4,6-METHYLBORONATE-2,3-DITMS

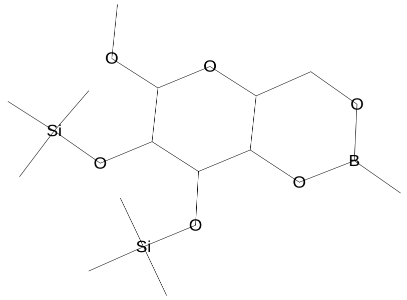

There is no signature data to report.

Library Search Report

Hit Spectrum	Delta	Compound Structure

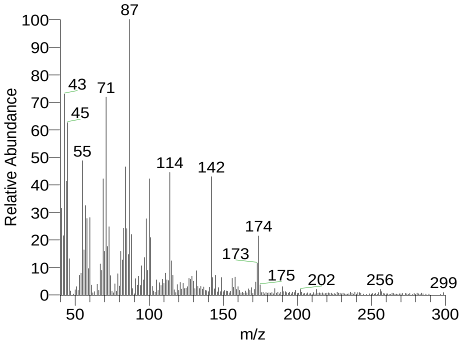

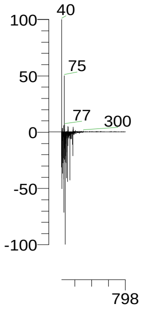

DEMYCAROSYLTURIMYCIN H
Formula C43H74N2O14, MW 842, CAS# 24916-50-5, Entry# 659647
FOROMACIDINE A

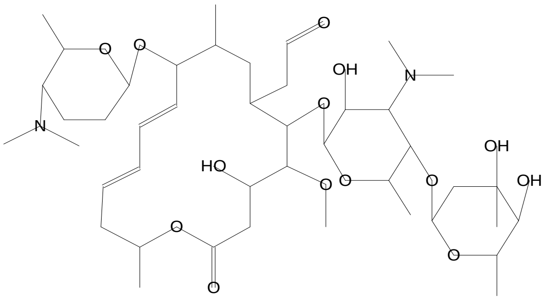

There is no signature data to report.

Library Search Report

RT	Probability	Compound Name	SI	Area %	Area	Molecular Weight	Molecular Formula	Library
51.08	19.22	Phenanthrene, 9-dodecyltetradecahydro- (CAS)	413	0.17	547049.20	360	C26H48	Wiley9
51.08	5.65	Anthracene, 9-dodecyltetradecahydro-	386	0.17	547049.20	360	C26H48	mainlib
51.08	5.65	Anthracene, 9-dodecyltetradecahydro- (CAS)	385	0.17	547049.20	360	C26H48	Wiley9

Faten-212 #13551 RT: 51.08 AV: 1 RF: 6.00, 3 NL: 4.94E4
F: {0,0} + c EI Full ms [40.00-800.00]

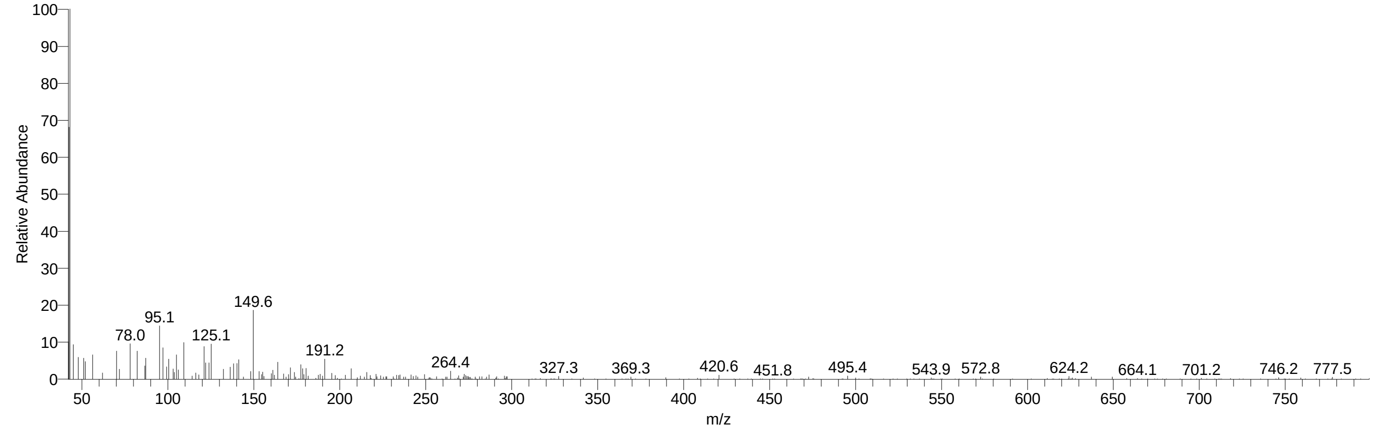

Hit Spectrum | Delta | Compound Structure

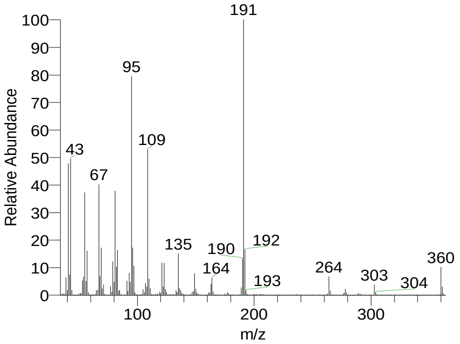

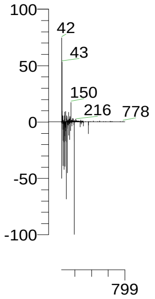

Phenanthrene, 9-dodecyltetradecahydro- (CAS)
Formula C26H48, MW 360, CAS# 55334-01-5, Entry# 488985
9-N-DODECYL-(TETRADECAHYDROPHENANTHRENE)

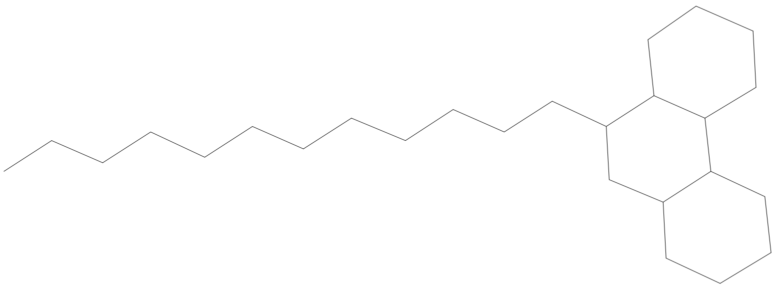

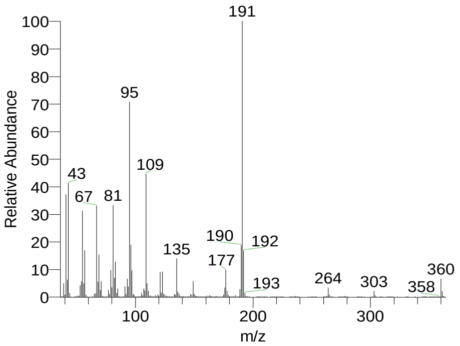

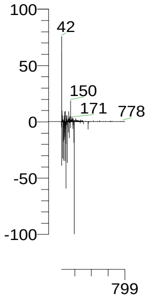

Anthracene, 9-dodecyltetradecahydro-
Formula C26H48, MW 360, CAS# 55401-75-7, Entry# 156504
9-n-Dodecyl(tetradecahydroanthracene)

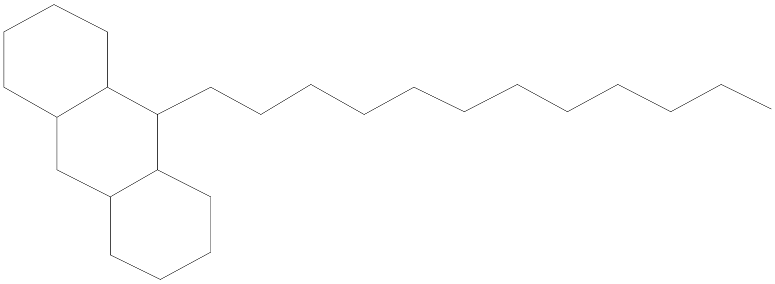

There is no signature data to report.

Library Search Report

Hit Spectrum	Delta	Compound Structure

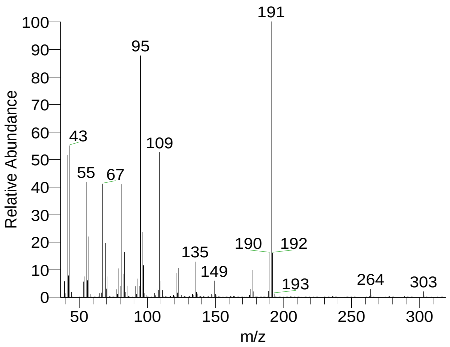

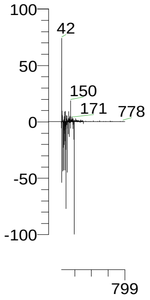

Anthracene, 9-dodecyltetradecahydro- (CAS)
Formula C26H48, MW 360, CAS# 55401-75-7, Entry# 488990
9-n-Dodecyl(tetradecahydroanthracene)

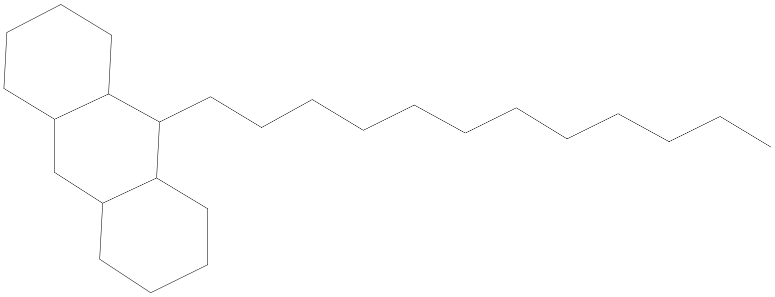

There is no signature data to report.

Library Search Report

RT	Probability	Compound Name	SI	Area %	Area	Molecular Weight	Molecular Formula	Library
51.19	9.60	6,9,12,15-Docosatetraenoic acid, methyl ester	431	0.29	914952.37	346	C23H38O2	mainlib
51.19	9.60	6,9,12,15-Docosatetraenoic acid, methyl ester (CAS)	431	0.29	914952.37	346	C23H38O2	Wiley9
51.19	8.11	Cyclopropanenonanoic acid, 2-[(2-butylcyclopropyl)methyl]-, methyl ester	427	0.29	914952.37	322	C21H38O2	mainlib

Faten-212 #13582 RT: 51.19 AV: 1 RF: 6.00, 3 NL: 1.34E4
F: {0,0} + c EI Full ms [40.00-800.00]

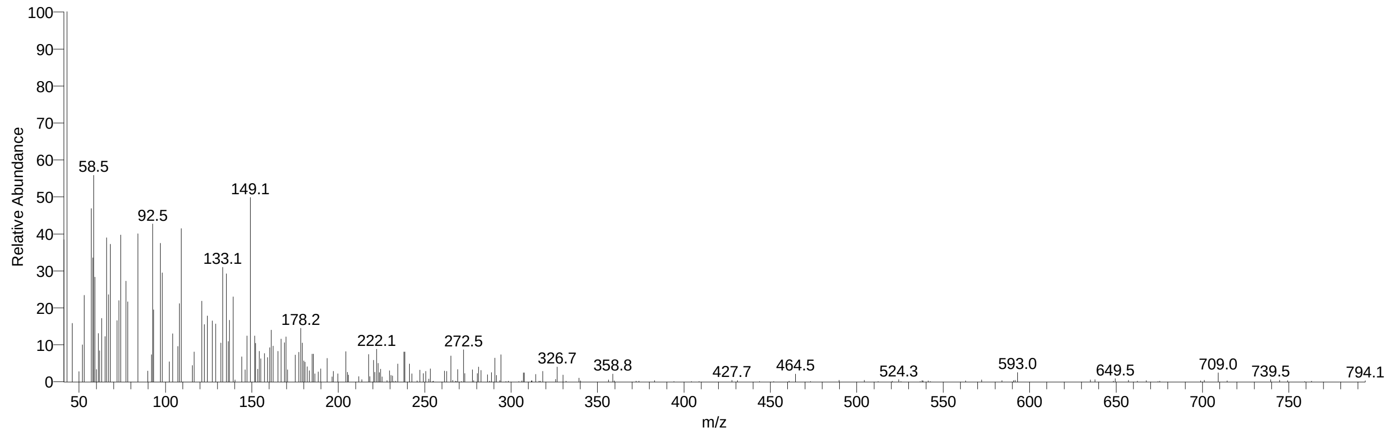

Hit Spectrum	Delta	Compound Structure

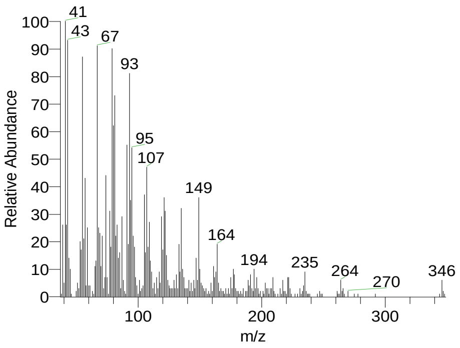

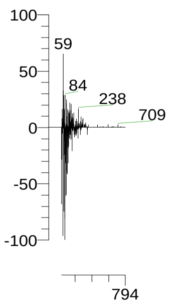

6,9,12,15-Docosatetraenoic acid, methyl ester
Formula C23H38O2, MW 346, CAS# 17364-34-0, Entry# 2311
Methyl (6E,9E,12E,15E)-6,9,12,15-docosatetraenoate #

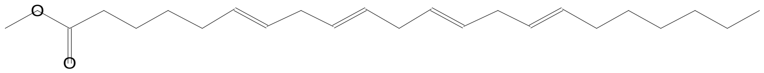

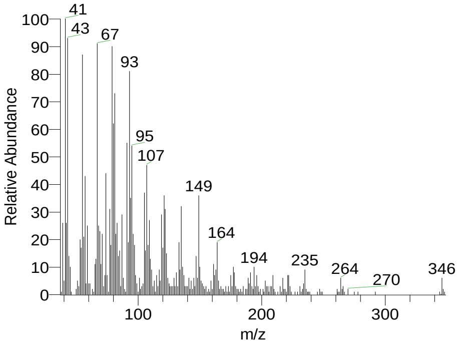

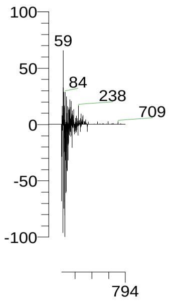

6,9,12,15-Docosatetraenoic acid, methyl ester (CAS)
Formula C23H38O2, MW 346, CAS# 17364-34-0, Entry# 465998
METHYL 6,9,12,15-DOCOSATETRAENOATE

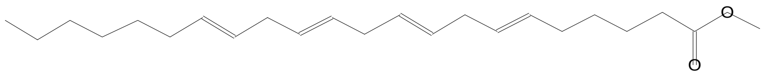

There is no signature data to report.

Library Search Report

Hit Spectrum	Delta	Compound Structure

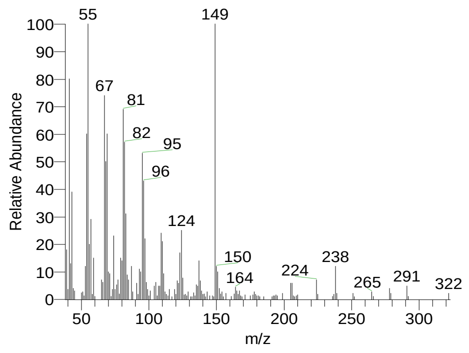

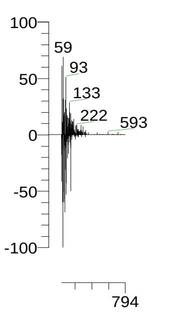

Cyclopropanenonanoic acid, 2-[(2-butylcyclopropyl)methyl]-, methyl ester
Formula C21H38O2, MW 322, CAS# 10152-69-9, Entry# 20140
Methyl 9-(2-[(2-butylcyclopropyl)methyl]cyclopropyl)nonanoate #

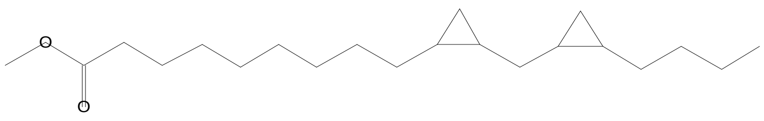

There is no signature data to report.

Library Search Report

RT	Probability	Compound Name	SI	Area %	Area	Molecular Weight	Molecular Formula	Library
51.26	24.51	(E,Z)-4,5-Dihydro-1,4,4-trimethyl-5-methylimino-1H-1,2,4-triazloe	341	0.30	956020.71	140	C6H12N4	Wiley9
51.26	5.87	1-TRIDEUTEROMETHYL-2R-METHYL-4R-ETHYNYL-TRANS-DECAHYDROQUINOLIN-4-OL AXIAL N-OXIDE	303	0.30	956020.71	223	C13H18D3NO2	Wiley9
51.26	4.26	Butane, 2-chloro-2-methyl- (CAS)	294	0.30	956020.71	106	C5H11Cl	Wiley9

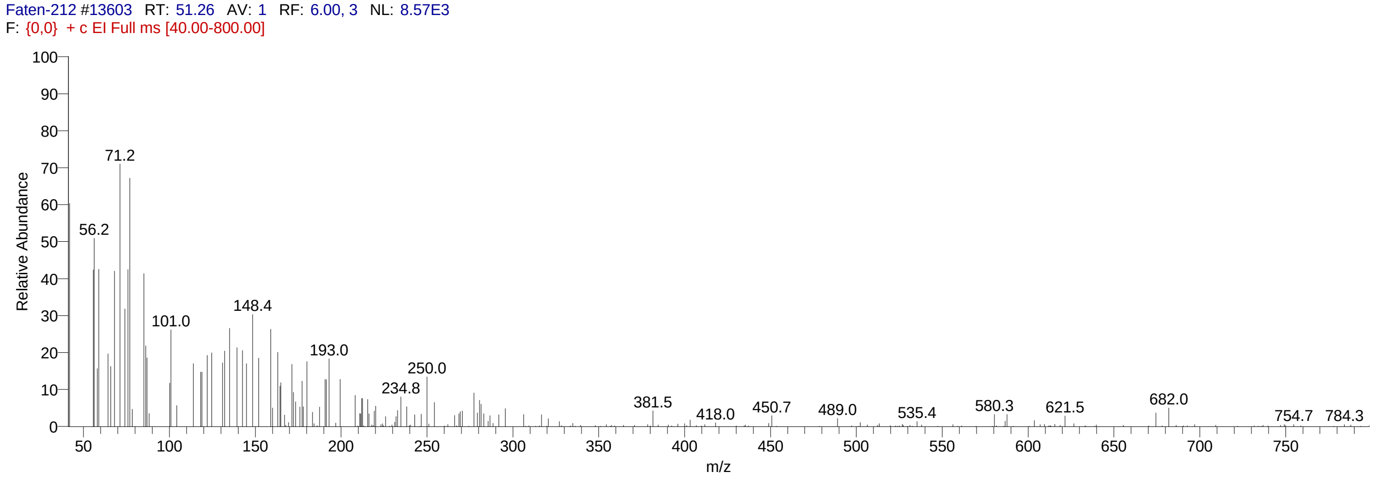

Hit Spectrum	Delta	Compound Structure

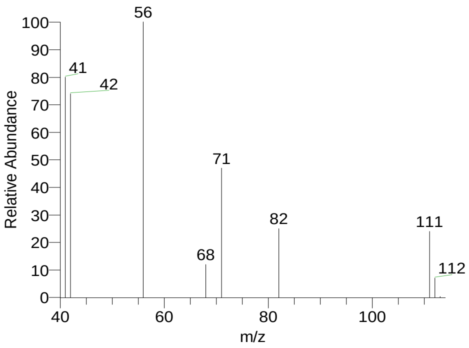

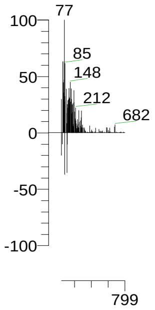

(E,Z)-4,5-Dihydro-1,4,4-trimethyl-5-methylimino-1H-1,2,4-triazloe
Formula C6H12N4, MW 140, CAS# 127645-61-8, Entry# 37530

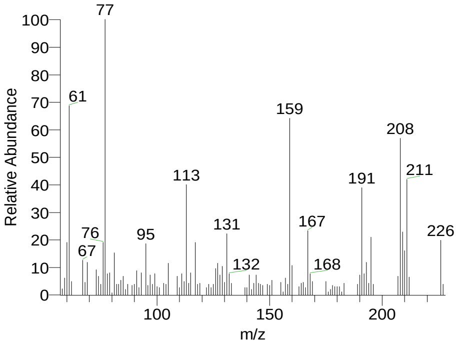

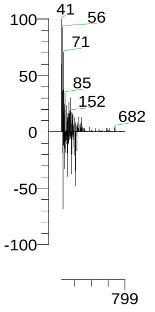

Formula C13H18D3NO2, MW 223, CAS# NA, Entry# 196077

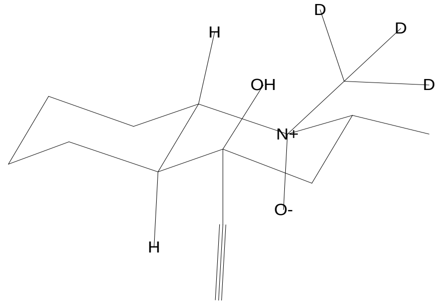

There is no signature data to report.

Library Search Report

Hit Spectrum

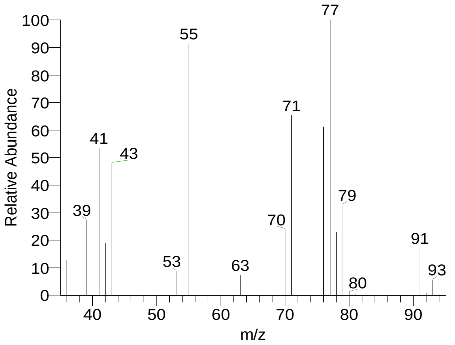

Delta

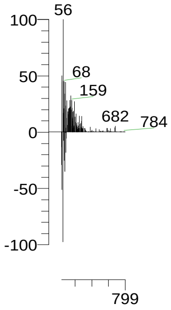

Compound Structure

Butane, 2-chloro-2-methyl- (CAS)
Formula C5H11Cl, MW 106, CAS# 594-36-5, Entry# 10609
tert-Amyl chloride

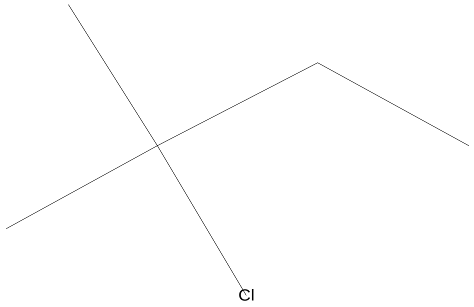

There is no signature data to report.

Library Search Report

RT	Probability	Compound Name	SI	Area %	Area	Molecular Weight	Molecular Formula	Library
53.10	8.17	8,11,14-Eicosatrienoic acid, methyl ester, (Z,Z,Z)-	423	0.13	418264.90	320	C21H36O2	mainlib
53.10	8.17	8,11,14-Eicosatrienoic acid, methyl ester (CAS)	423	0.13	418264.90	320	C21H36O2	Wiley9
53.10	8.17	8,11,14-Eicosatrienoic acid, methyl ester, (Z,Z,Z)- (CAS)	423	0.13	418264.90	320	C21H36O2	Wiley9

Faten-212 #14145 RT: 53.10 AV: 1 RF: 6.00, 3 NL: 3.95E4
F: {0,0} + c EI Full ms [40.00-800.00]

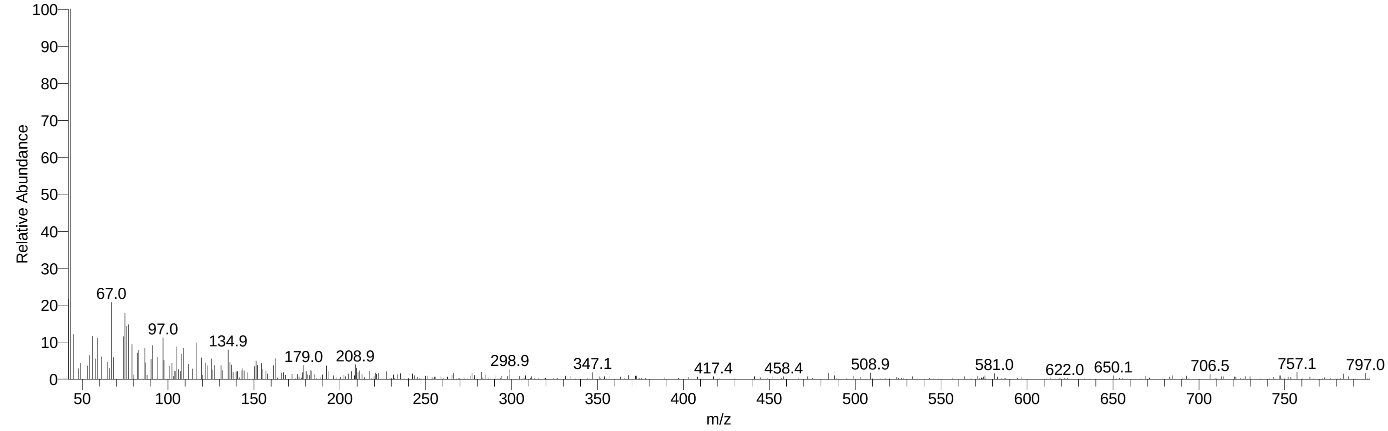

Hit Spectrum	Delta	Compound Structure

8,11,14-Eicosatrienoic acid, methyl ester, (Z,Z,Z)-
Formula C21H36O2, MW 320, CAS# 21061-10-9, Entry# 5561
Methyl dihomo-ç-linolenate

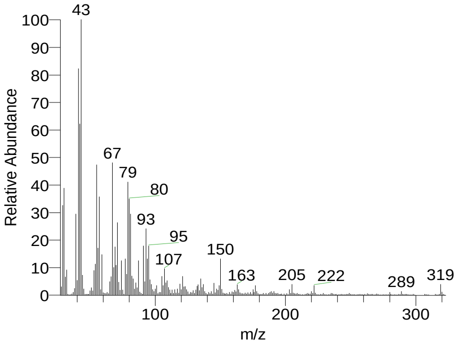

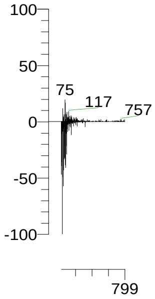

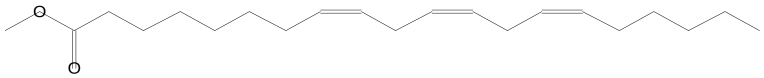

8,11,14-Eicosatrienoic acid, methyl ester (CAS)
Formula C21H36O2, MW 320, CAS# 17364-32-8, Entry# 417539
METHYL 8,11,14-EICOSATRIENOATE

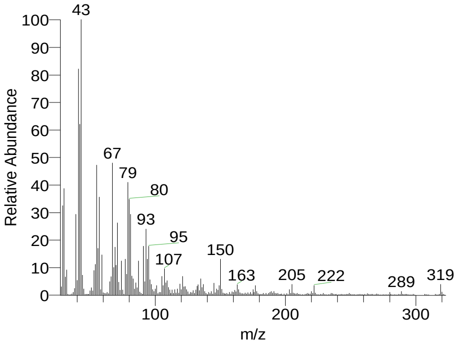

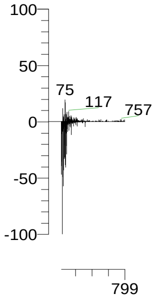

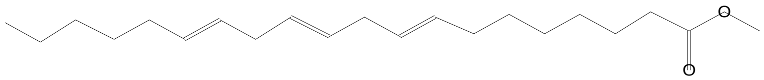

There is no signature data to report.

Library Search Report

Hit Spectrum	Delta	Compound Structure

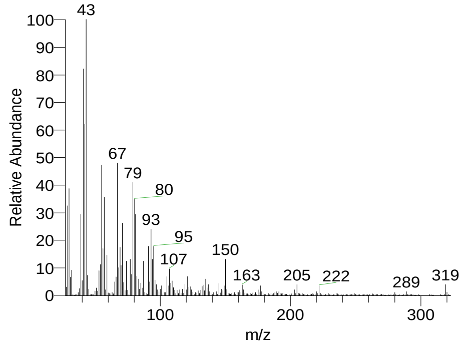

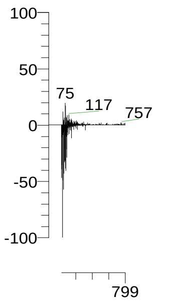

8,11,14-Eicosatrienoic acid, methyl ester, (Z,Z,Z)- (CAS)
Formula C21H36O2, MW 320, CAS# 21061-10-9, Entry# 417564
Methyl dihomo-ç-linolenate

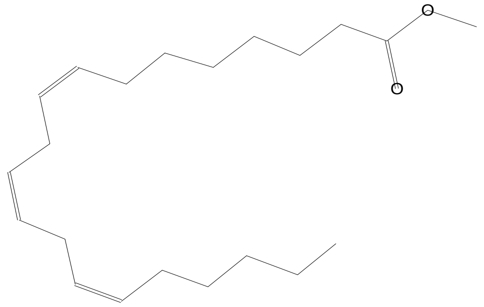

There is no signature data to report.

Library Search Report

RT	Probability	Compound Name	SI	Area %	Area	Molecular Weight	Molecular Formula	Library
53.59	10.84	Octadecatrienoic acid, methyl ester, labeled with carbon-13 (CAS)	425	0.16	495720.29	292	C19H32O2	Wiley9
53.59	10.84	METHYL 13C OCTADECATRIENOATE	425	0.16	495720.29	292	C19H32O2	Wiley9
53.59	9.15	2-Acetyl-3-(2-benzenesulphonamido)ethyl-7-methoxyindole	421	0.16	495720.29	372	C19H20N2O4S	Wiley9

Faten-212 #14288 RT: 53.59 AV: 1 RF: 6.00, 3 NL: 1.14E4
F: {0,0} + c EI Full ms [40.00-800.00]

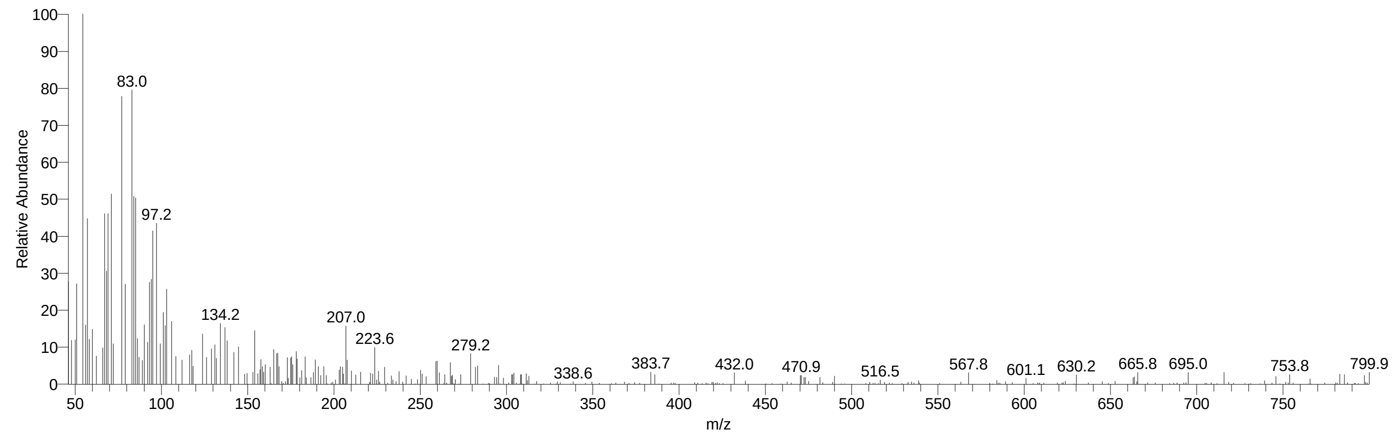

Hit Spectrum	Delta	Compound Structure

Octadecatrienoic acid, methyl ester, labeled with carbon-13 (CAS)
Formula C19H32O2, MW 292, CAS# 72088-15-4, Entry# 357377

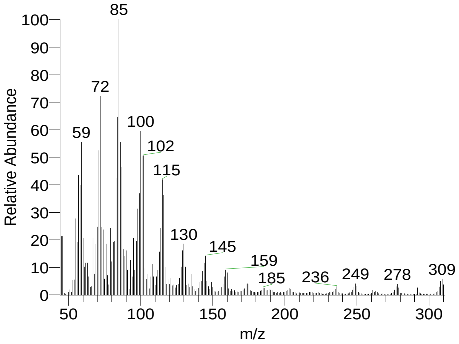

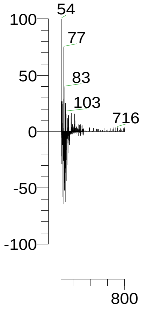

METHYL 13C OCTADECATRIENOATE
Formula C19H32O2, MW 292, CAS# NA, Entry# 357460

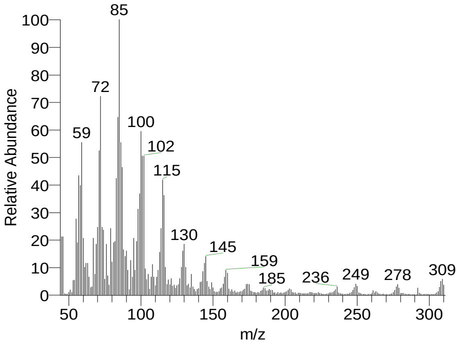

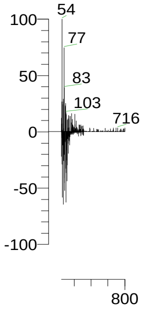

There is no signature data to report.

Library Search Report

Hit Spectrum	Delta	Compound Structure

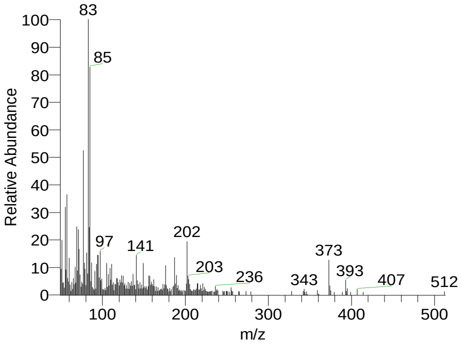

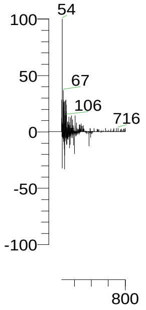

2-Acetyl-3-(2-benzenesulphonamido)ethyl-7-methoxyindole
Formula C19H20N2O4S, MW 372, CAS# NA, Entry# 505973

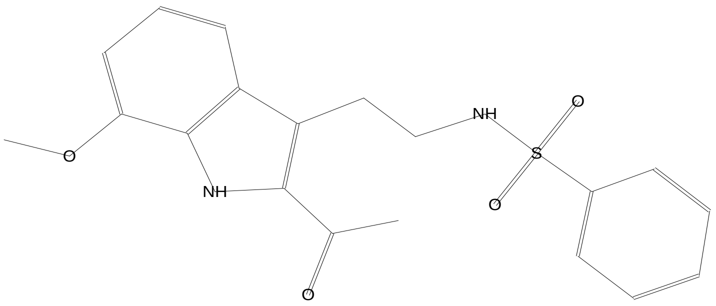

There is no signature data to report.

Library Search Report

RT	Probability	Compound Name	SI	Area %	Area	Molecular Weight	Molecular Formula	Library
55.16	17.19	2,16,19,20-TETRAHYDROMOSSAMBINE	387	0.14	437538.93	342	C20H26N2O3	Wiley9
55.16	7.83	Olean-12-ene-3,16,21,22,28-pentol, 21-(2-methyl-2-butenoate), [3á,16à,21á(Z),22à]-	367	0.14	437538.93	572	C35H56O6	mainlib
55.16	7.83	Olean-12-ene-3,16,21,22,28-pentol, 21-(2-methyl-2-butenoate), [3á,16à,21á(Z),22à]-(CAS)	367	0.14	437538.93	572	C35H56O6	Wiley9

Faten-212 #14750 RT: 55.16 AV: 1 RF: 6.00, 3 NL: 6.97E4
F: {0,0} + c EI Full ms [40.00-800.00]

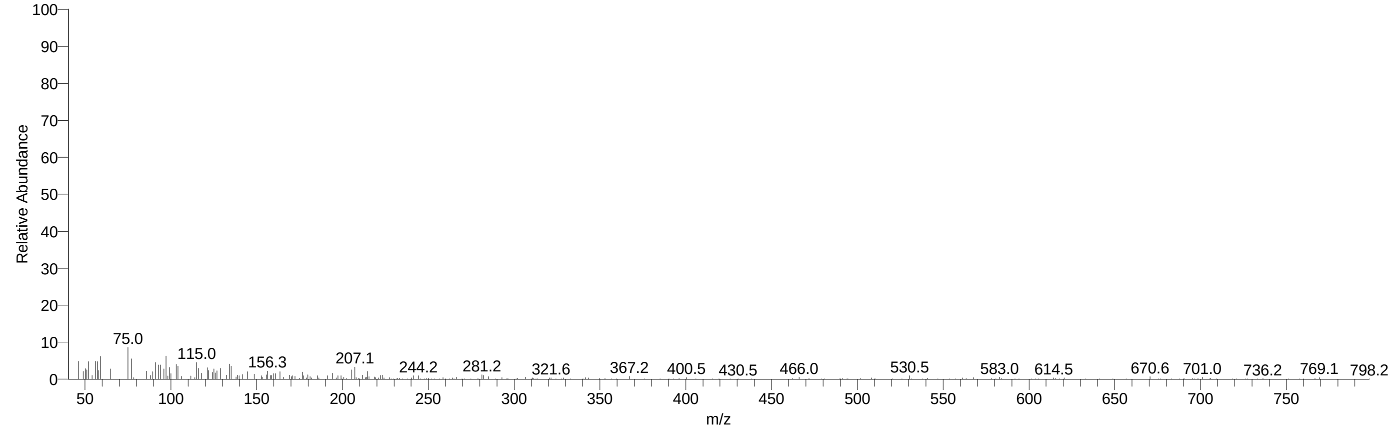

Hit Spectrum | Delta | Compound Structure

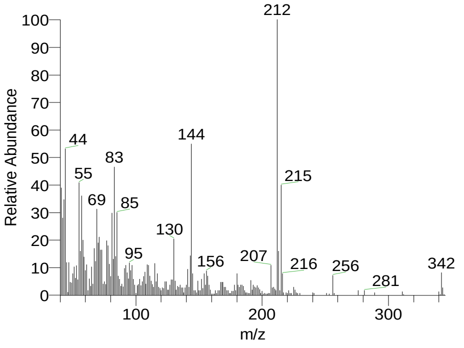

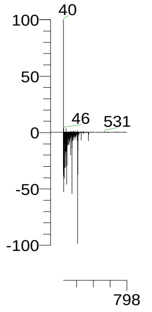

2,16,19,20-TETRAHYDROMOSSAMBINE
Formula C20H26N2O3, MW 342, CAS# NA, Entry# 458234

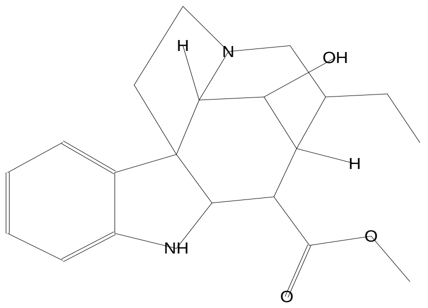

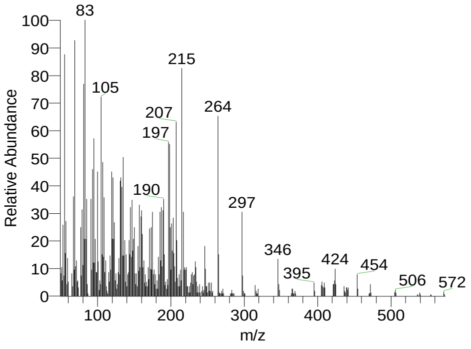

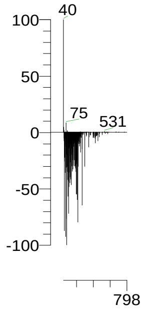

Olean-12-ene-3,16,21,22,28-pentol, 21-(2-methyl-2-butenoate), [3á,16à,21á(Z),22à]-
Formula C35H56O6, MW 572, CAS# 20089-98-9, Entry# 47874
Olean-12-ene-3á,16à,21á,22à,28-pentol, 16-(2-methylcrotonate), (Z)-

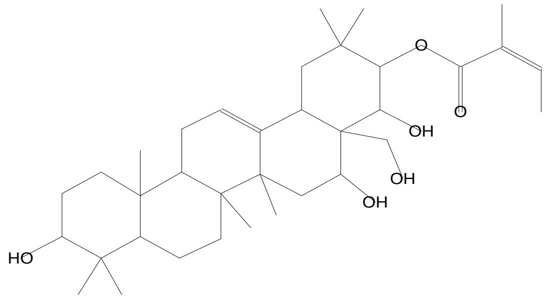

There is no signature data to report.

Library Search Report

Hit Spectrum	Delta	Compound Structure

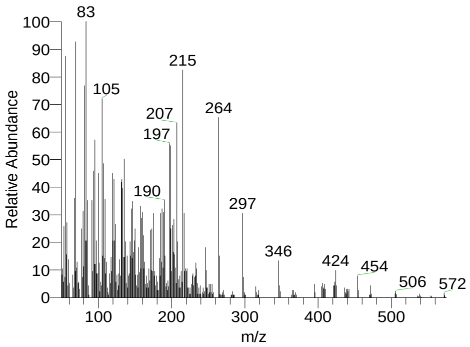

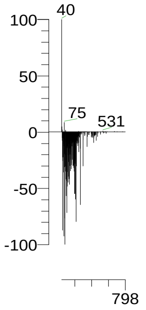

Olean-12-ene-3,16,21,22,28-pentol, 21-(2-methyl-2-butenoate), [3á,16à,21á(Z),22à]- (CAS)
Formula C35H56O6, MW 572, CAS# 20089-98-9, Entry# 639219
3á,21á,22à,28-TETRAHYDROXY-16à-ANGELOYLOXY-OLEAN-12-ENE

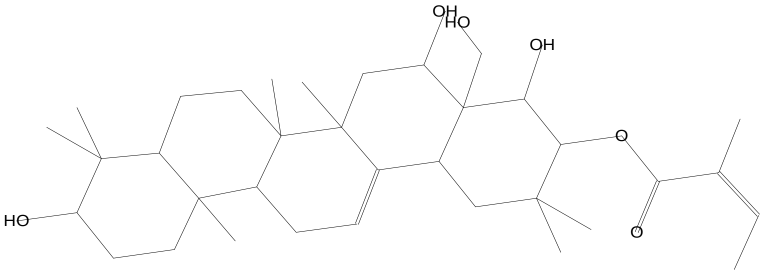

There is no signature data to report.

Library Search Report

RT	Probability	Compound Name	SI	Area %	Area	Molecular Weight	Molecular Formula	Library
56.50	12.14	Tertbutyloxyformamide, N-methyl-N-[4-(1-pyrrolidinyl)-2-butynyl]-	442	0.13	417210.87	252	C14H24N2O2	mainlib
56.50	8.57	Paromomycin	432	0.13	417210.87	615	C23H45N5O14	mainlib
56.50	7.91	Bupropion	430	0.13	417210.87	239	C13H18ClNO	Wiley9

Faten-212 #15145 RT: 56.50 AV: 1 RF: 6.00, 3 NL: 4.85E4
F: {0,0} + c EI Full ms [40.00-800.00]

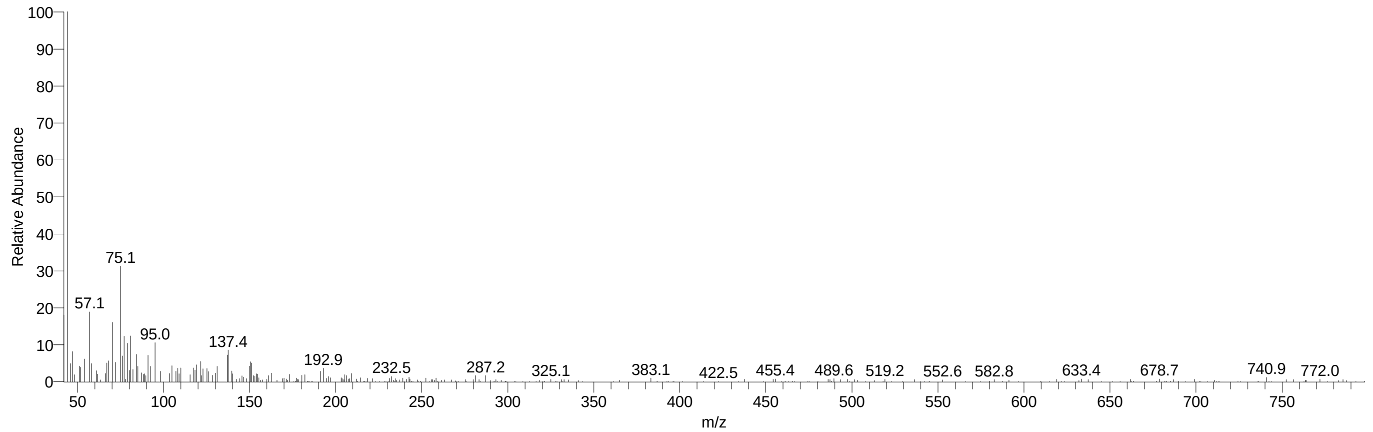

Hit Spectrum	Delta	Compound Structure

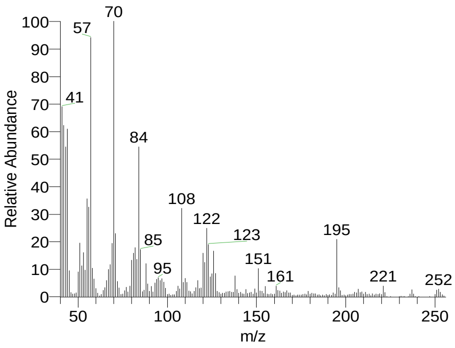

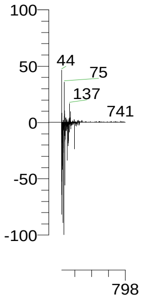

Tertbutyloxyformamide, N-methyl-N-[4-(1-pyrrolidinyl)-2-butynyl]-
Formula C14H24N2O2, MW 252, CAS# 124045-68-7, Entry# 34145
t-Butoxyformamid, N-methyl-N-[4-(1-pyrrolidinyl)-2-butynyl]-

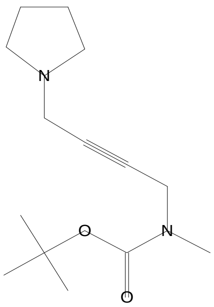

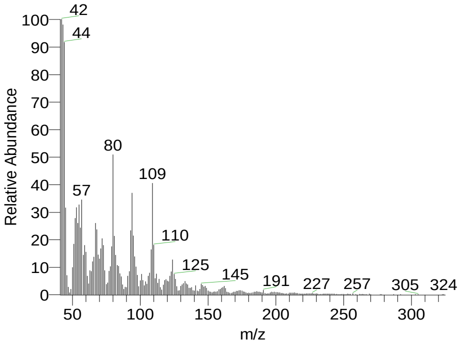

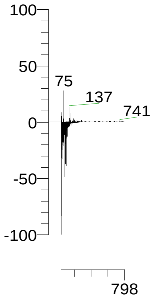

Paromomycin
Formula C23H45N5O14, MW 615, CAS# 7542-37-2, Entry# 4301

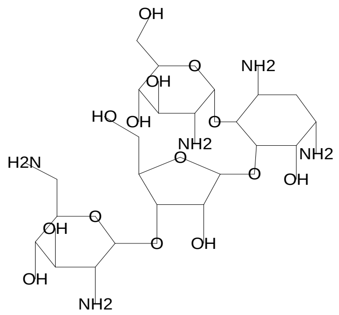

There is no signature data to report.

Library Search Report

Hit Spectrum	Delta	Compound Structure

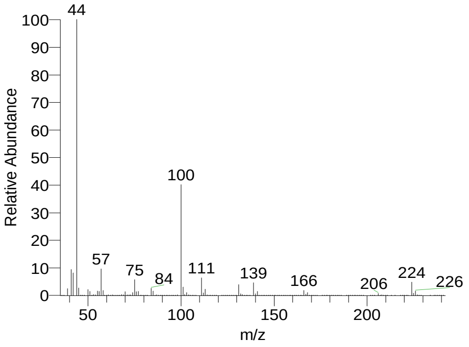

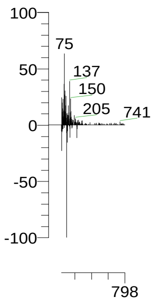

Bupropion
Formula C13H18ClNO, MW 239, CAS# 34911-55-2, Entry# 233783
1-Propanone, 1-(3-chlorophenyl)-2-((1,1-dimethylethyl)amino)-

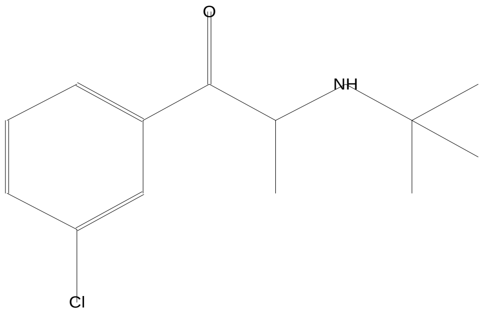

There is no signature data to report.

Library Search Report

RT	Probability	Compound Name	SI	Area %	Area	Molecular Weight	Molecular Formula	Library
59.25	7.54	Dotriacontane (CAS)	468	0.14	432444.98	450	C32H66	Wiley9
59.25	5.78	1-Heptatriacotanol	461	0.14	432444.98	536	C37H76O	mainlib
59.25	5.33	DI-2-BENZOTHIAZOLE DISULFANE	459	0.14	432444.98	332	C14H8N2S4	Wiley9

Faten-212 #15952 RT: 59.25 AV: 1 RF: 6.00, 3 NL: 5.62E4
F: {0,0} + c EI Full ms [40.00-800.00]

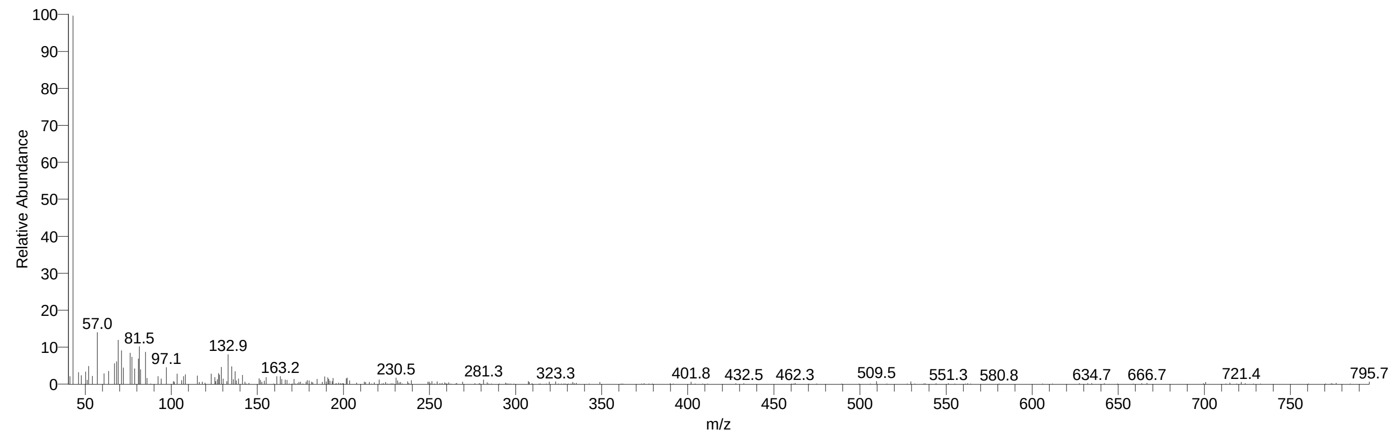

Hit Spectrum	Delta	Compound Structure

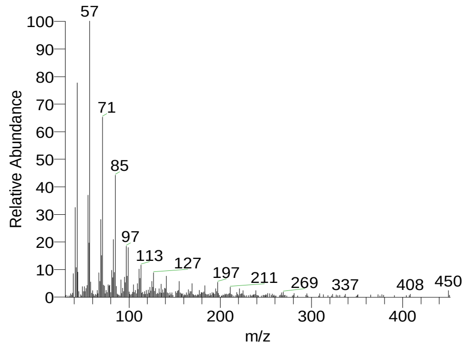

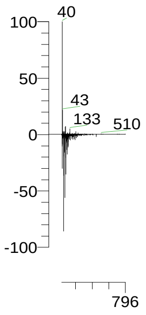

Dotriacontane (CAS)
Formula C32H66, MW 450, CAS# 544-85-4, Entry# 589615
n-Dotriacontane

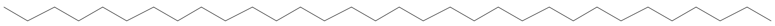

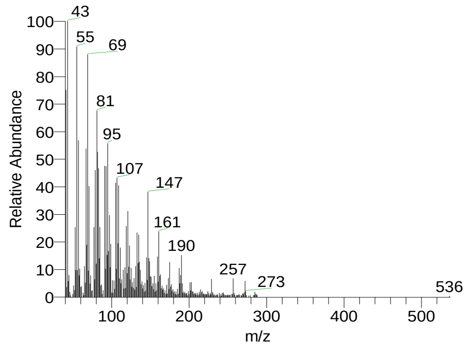

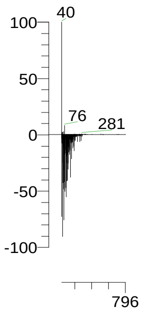

1-Heptatriacotanol
Formula C37H76O, MW 536, CAS# 105794-58-9, Entry# 6904
1-Heptatriacontanol #

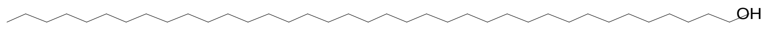

There is no signature data to report.

Library Search Report

Hit Spectrum	Delta	Compound Structure

DI-2-BENZOTHIAZOLE DISULFANE
Formula C14H8N2S4, MW 332, CAS# NA, Entry# 438926

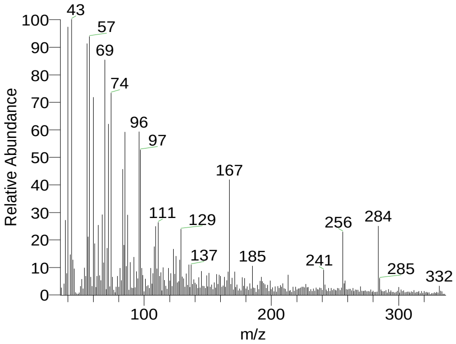

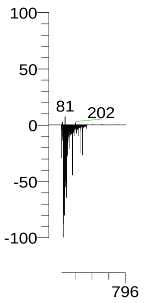

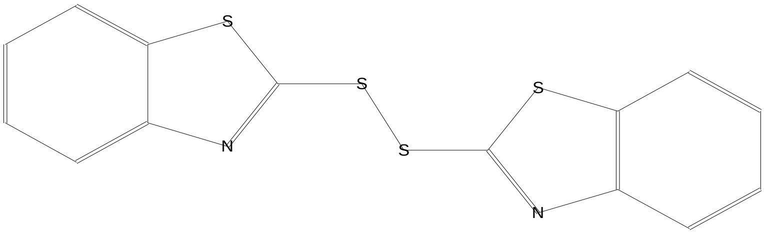

There is no signature data to report.

Library Search Report

RT	Probability	Compound Name	SI	Area %	Area	Molecular Weight	Molecular Formula	Library
62.95	12.55	2-(4-Chloro-1-naphthyloxy)-N'-(2-thenylidene) acethydrazide	445	0.21	677632.11	344	C17H13ClN2O2S	Wiley9
62.95	12.06	3',8,8'-Trimethoxy-3-piperidyl-2,2'-binaphthalene-1,1',4,4'-tetrone	444	0.21	677632.11	487	C28H25NO7	mainlib
62.95	10.19	nickel(II) octaethylbenzochlorin acetoxy derivative	440	0.21	677632.11	686	C41H48N4NiO2	Wiley9

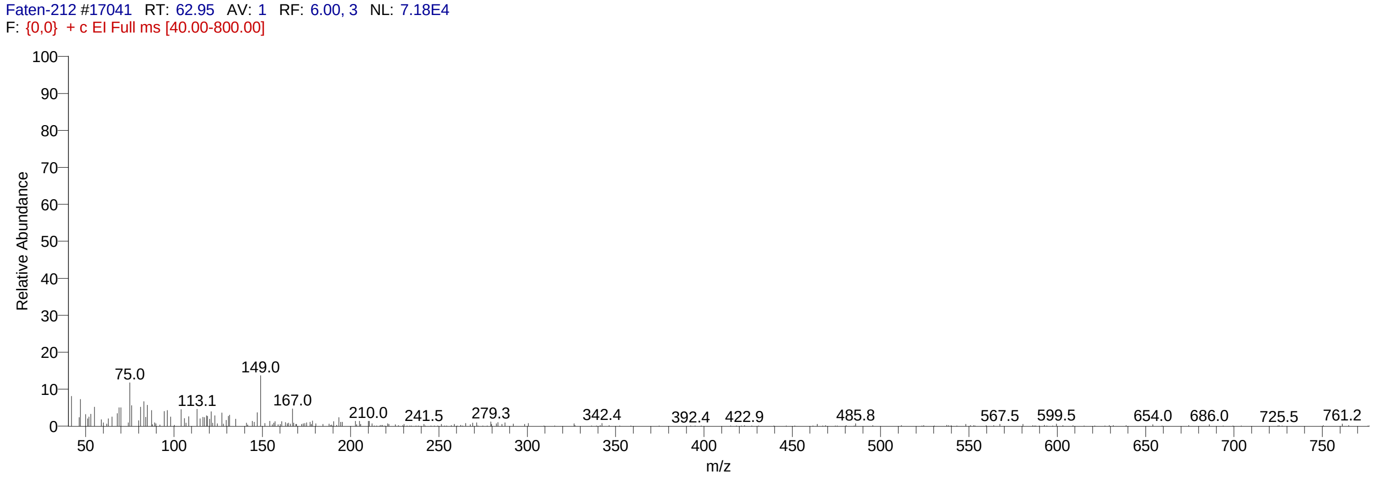

Hit Spectrum | Delta | Compound Structure

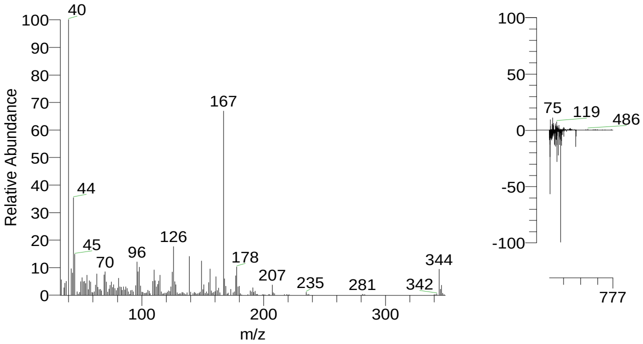

2-(4-Chloro-1-naphthyloxy)-N'-(2-thenylidene)acethydrazide
Formula C17H13ClN2O2S, MW 344, CAS# NA, Entry# 460897
2-[(4-Chloro-1-naphthyl)oxy]-N'-[(E)-2-thienylmethylidene]acetohydrazide

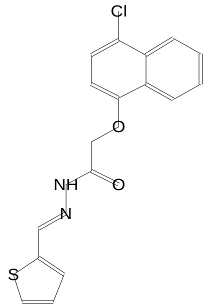

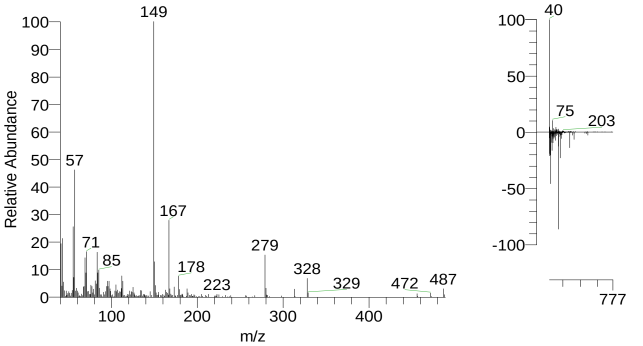

3',8,8'-Trimethoxy-3-piperidyl-2,2'-binaphthalene-1,1',4,4'-tetrone
Formula C28H25NO7, MW 487, CAS# 127611-84-1, Entry# 121431
3',8,8'-Trimethoxy-3-piperidin-1-yl-2,2'-binaphthyl-1,1',4,4'-tetrone

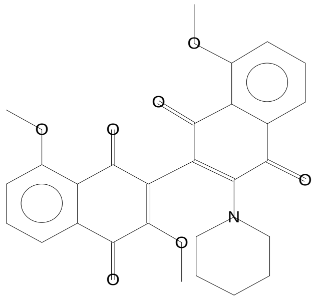

There is no signature data to report.

Library Search Report

Hit Spectrum	Delta	Compound Structure

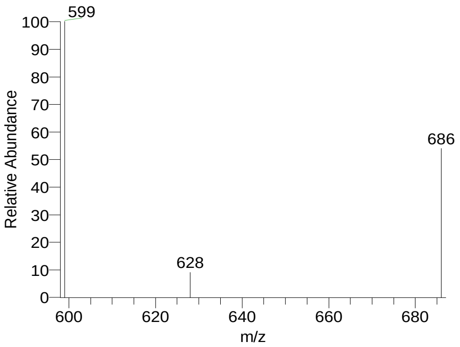

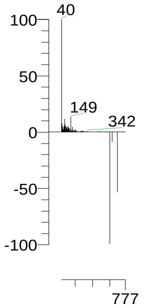

nickel(II) octaethylbenzochlorin acetoxy derivative
Formula C41H48N4NiO2, MW 686, CAS# NA, Entry# 653661
3-[(Acetoxy)ethylidene]-2,3,7,8,12,13,17,18-octaethylbenzochlorin

There is no signature data to report.

Library Search Report

RT	Probability	Compound Name	SI	Area %	Area	Molecular Weight	Molecular Formula	Library
64.42	43.04	1-(2-trimethylsiloxy-1,1-dideuteriovinyl)-4-trimethylsiloxy-benzene	531	0.13	413671.87	280	C14H22D2O2Si2	Wiley9
64.42	10.80	1-[(2-trimethylsiloxy)vinyl]-4-trimethylsiloxy-2,6-dideuteriobenzene	496	0.13	413671.87	280	C14H22D2O2Si2	Wiley9
64.42	9.96	1-(2-trimethylsiloxyvinyl)-4-trimethylsiloxy-3,5-dideuteriobenzene	494	0.13	413671.87	280	C14H22D2O2Si2	Wiley9

Faten-212 #17472 RT: 64.42 AV: 1 RF: 6.00, 3 NL: 1.83E4
F: {0,0} + c EI Full ms [40.00-800.00]

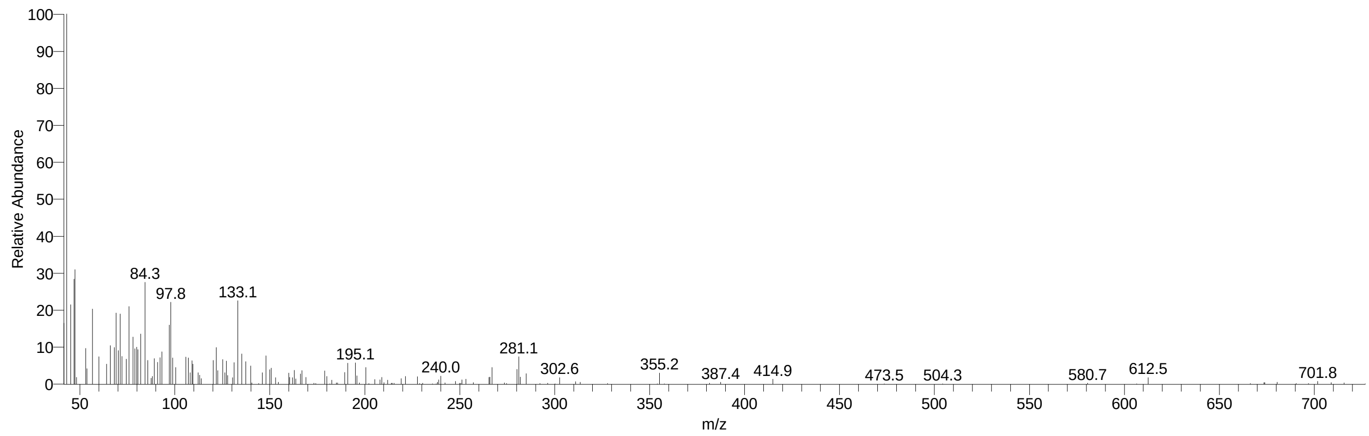

Hit Spectrum	Delta	Compound Structure

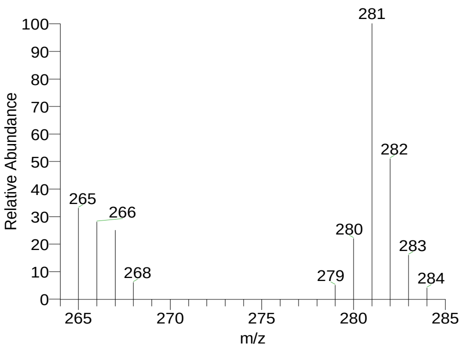

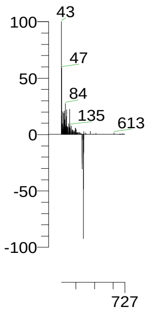

1-(2-trimethylsiloxy-1,1-dideuteriovinyl)-4-trimethylsiloxy-benzene
Formula C14H22D2O2Si2, MW 280, CAS# 126210-55-7, Entry# 328880
Silane, trimethyl[4-[1-[(trimethylsilyl)oxy]ethenyl-2,2-d2]phenoxy]- (CAS)

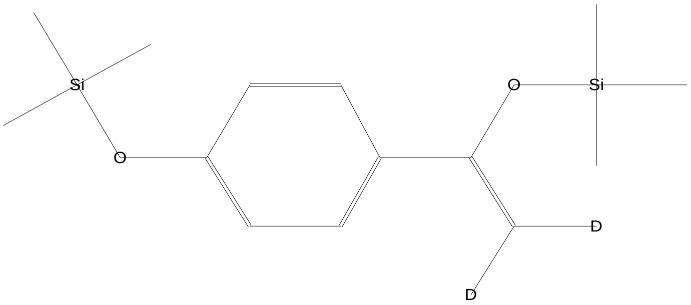

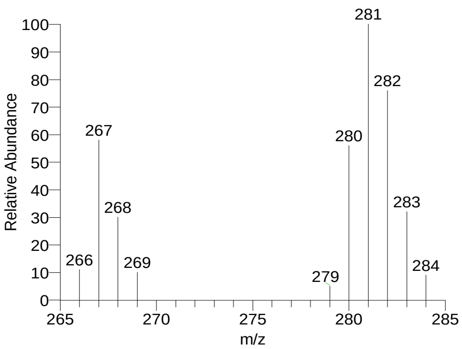

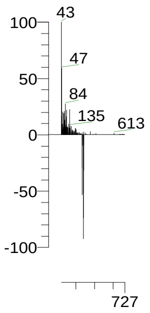

1-[(2-trimethylsiloxy)vinyl]-4-trimethylsiloxy-2,6-dideuteriobenzene
Formula C14H22D2O2Si2, MW 280, CAS# 126210-57-9, Entry# 328881
Silane, trimethyl[4-[1-[(trimethylsilyl)oxy]ethenyl]phenoxy-2,6-d2]- (CAS)

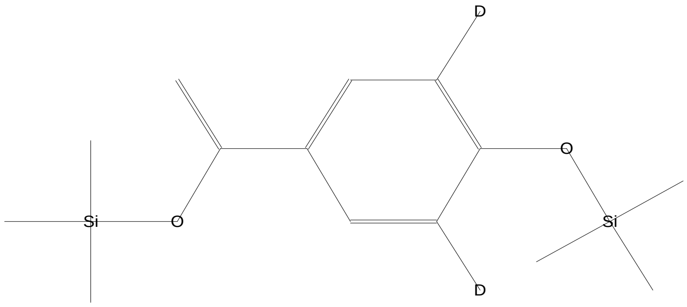

There is no signature data to report.

Library Search Report

Hit Spectrum	Delta	Compound Structure

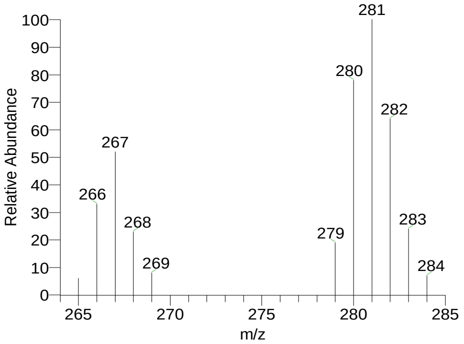

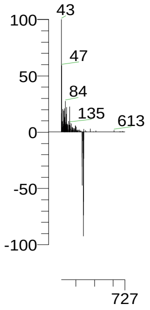

1-(2-trimethylsiloxyvinyl)-4-trimethylsiloxy-3,5-dideuteriobenzene
Formula C14H22D2O2Si2, MW 280, CAS# 126210-56-8, Entry# 328882
Silane, trimethyl[4-[1-[(trimethylsilyl)oxy]ethenyl]phenoxy-3,5-d2]- (CAS)

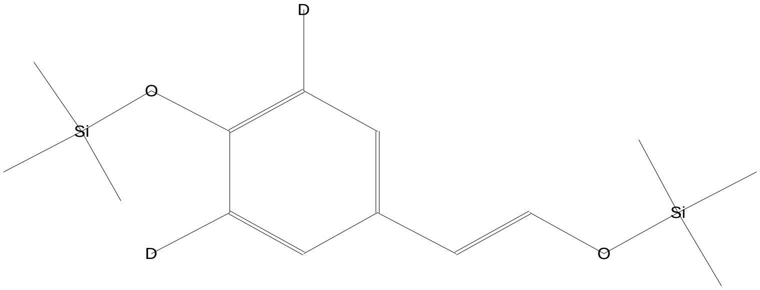

There is no signature data to report.

Library Search Report

RT	Probability	Compound Name	SI	Area %	Area	Molecular Weight	Molecular Formula	Library
67.94	9.55	N,N'-Bis(Carbobenzyloxy)-lysine methyl(ester)	405	0.13	400555.23	428	C23H28N2O6	mainlib
67.94	5.50	8,11,14-Eicosatrienoic acid, methyl ester, (Z,Z,Z)-	389	0.13	400555.23	320	C21H36O2	mainlib
67.94	5.50	8,11,14-Eicosatrienoic acid, methyl ester (CAS)	389	0.13	400555.23	320	C21H36O2	Wiley9

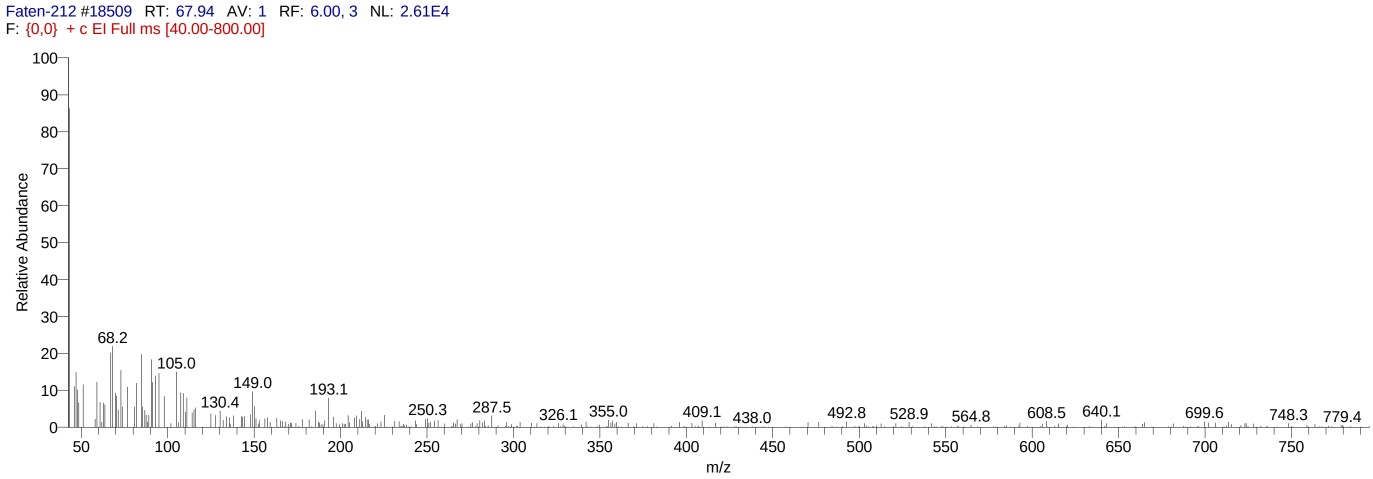

Hit Spectrum	Delta	Compound Structure

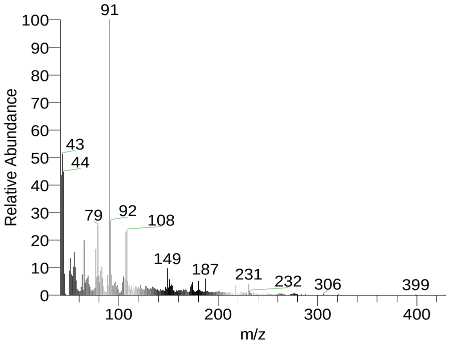

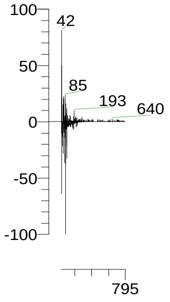

N,N'-Bis(Carbobenzyloxy)-lysine methyl(ester)
Formula C23H28N2O6, MW 428, CAS# NA, Entry# 53669
Methyl 2,6-bis([(benzyloxy)carbonyl]amino)hexanoate #

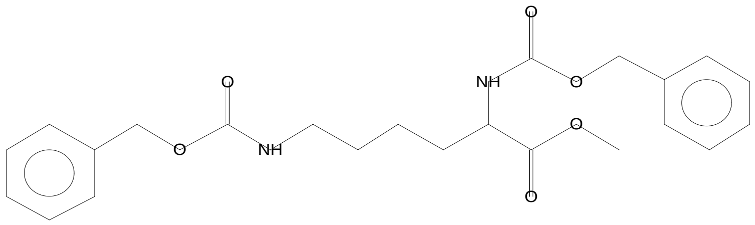

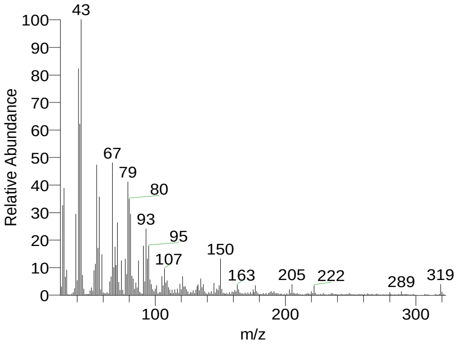

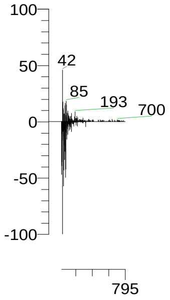

8,11,14-Eicosatrienoic acid, methyl ester, (Z,Z,Z)-
Formula C21H36O2, MW 320, CAS# 21061-10-9, Entry# 5561
Methyl dihomo-ç-linolenate

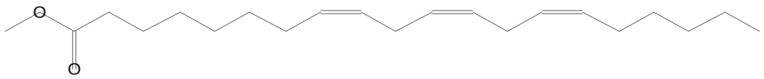

There is no signature data to report.

Library Search Report

Hit Spectrum	Delta	Compound Structure

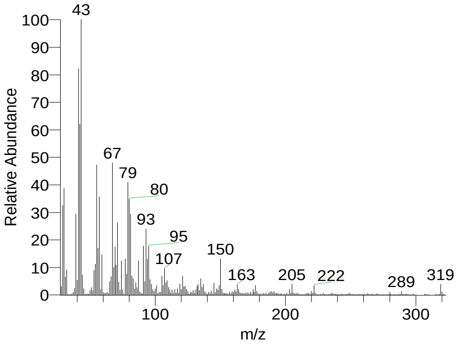

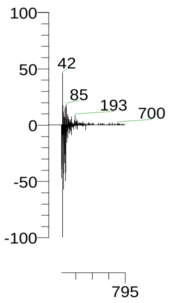

8,11,14-Eicosatrienoic acid, methyl ester (CAS)
Formula C21H36O2, MW 320, CAS# 17364-32-8, Entry# 417539
METHYL 8,11,14-EICOSATRIENOATE

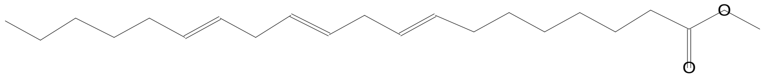

There is no signature data to report.

Library Search Report

RT	Probability	Compound Name	SI	Area %	Area	Molecular Weight	Molecular Formula	Library
79.44	7.23	Bufa-20,22-dienolide, 14,15-epoxy-3,11-dihydroxy-, (3á,5á,11à,15á)-	396	0.16	522078.03	400	C24H32O5	mainlib
79.44	7.23	11à-HYDROZYRESIBUFOGENIN	394	0.16	522078.03	400	C24H32O5	Wiley9
79.44	6.67	GLYCERYL TRIDOCASAHEXAENOATE	394	0.16	522078.03	942	C69H98O	Wiley9

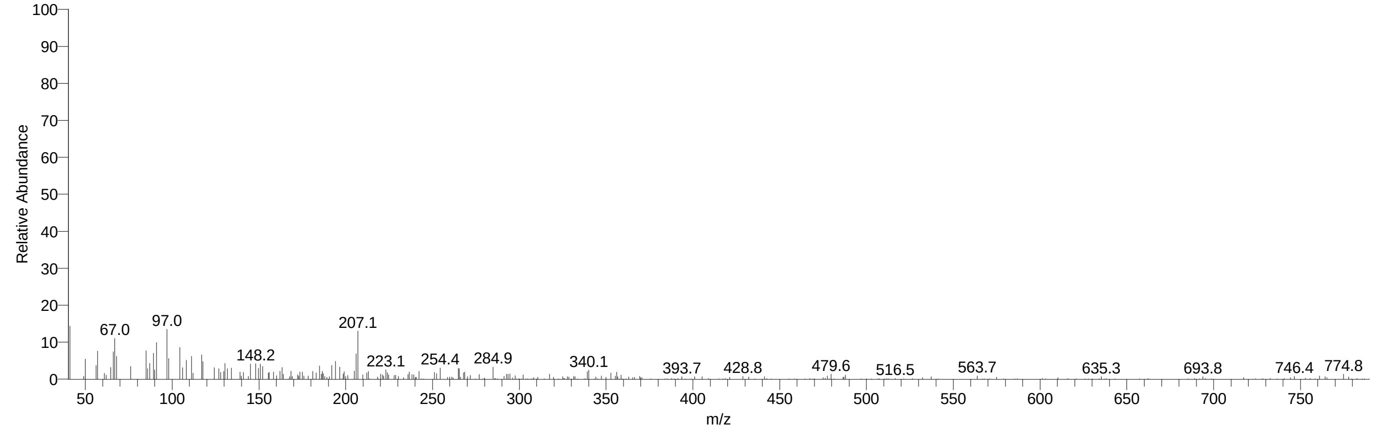

Hit Spectrum	Delta	Compound Structure

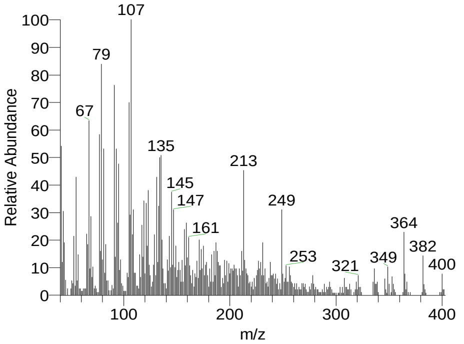

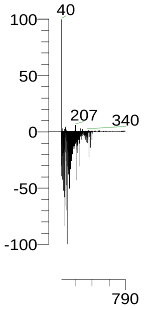

Bufa-20,22-dienolide, 14,15-epoxy-3,11-dihydroxy-, (3á,5á,11à,15á)-
Formula C24H32O5, MW 400, CAS# 39005-15-7, Entry# 75442
11à-Hydroxyresibufogenin

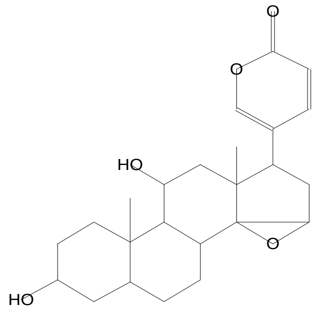

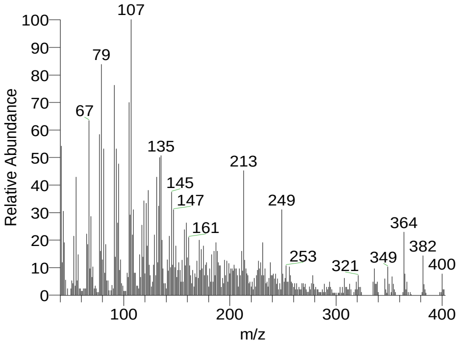

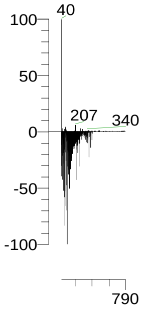

11à-HYDROZYRESIBUFOGENIN
Formula C24H32O5, MW 400, CAS# 39005-15-7, Entry# 542688
Bufa-20,22-dienolide, 14,15-epoxy-3,11-dihydroxy-, (3á,5á,11à,15á)- (CAS)

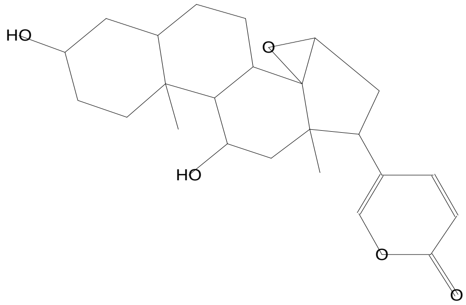

There is no signature data to report.

Library Search Report

Hit Spectrum	Delta	Compound Structure
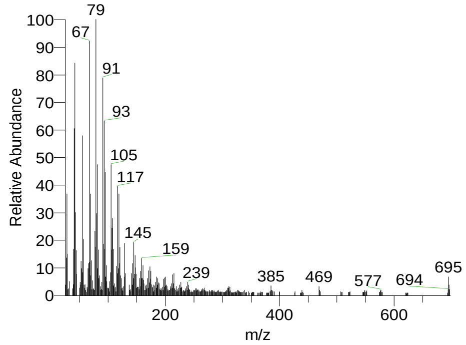	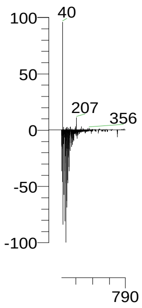	GLYCERYL TRIDOCASAHEXAENOATE<br>Formula C69H98O, MW 942, CAS# NA, Entry# 660874

There is no signature data to report.

Library Search Report

RT	Probability	Compound Name	SI	Area %	Area	Molecular Weight	Molecular Formula	Library
79.62	10.41	Betamethasone acetate	463	0.16	511915.05	434	C24H31FO6	mainlib
79.62	8.18	Beclomethasone	457	0.16	511915.05	408	C22H29ClO5	mainlib
79.62	6.91	Cholan-24-oic acid, 3-(acetyloxy)-7,12-dioxo-, methyl ester, (3à,5á)-	453	0.16	511915.05	460	C27H40O6	mainlib

Faten-212 #21941 RT: 79.62 AV: 1 RF: 6.00, 3 NL: 7.29E4
F: {0,0} + c EI Full ms [40.00-800.00]

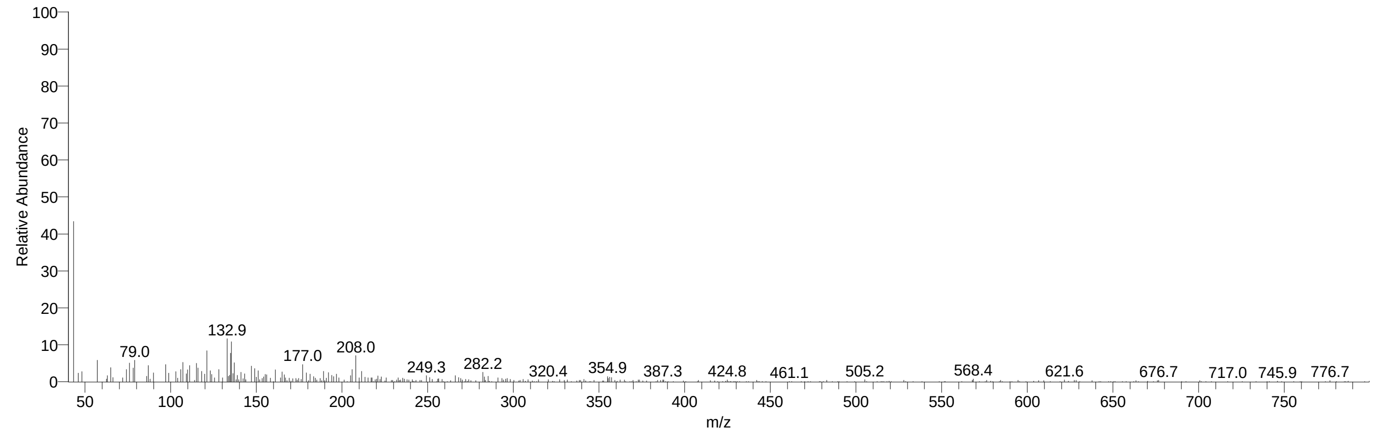

Hit Spectrum | Delta | Compound Structure

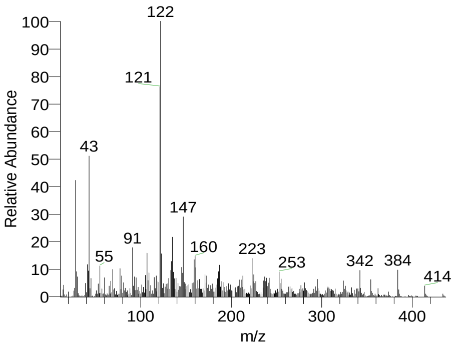

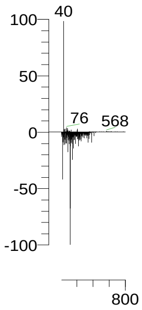

Betamethasone acetate
Formula C24H31FO6, MW 434, CAS# 987-24-6, Entry# 93589
Pregna-1,4-diene-3,20-dione, 21-(acetyloxy)-9-fluoro-11,17-dihydroxy-16-methyl-, (11á,16á)-

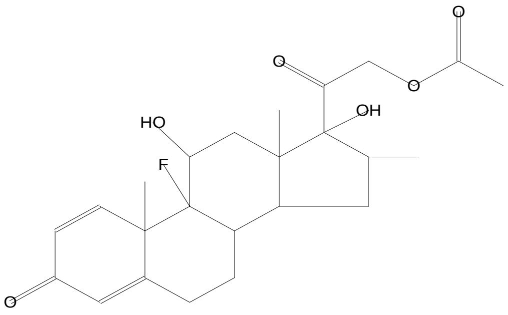

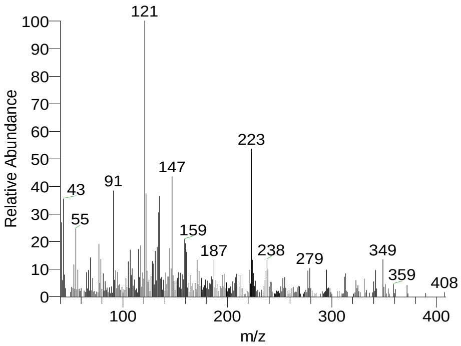

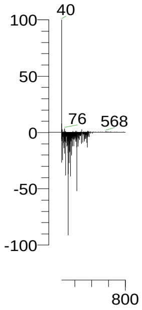

Beclomethasone
Formula C22H29ClO5, MW 408, CAS# 4419-39-0, Entry# 92887
Pregna-1,4-diene-3,20-dione, 9-chloro-11,17,21-trihydroxy-16-methyl-, (11á,16á)-

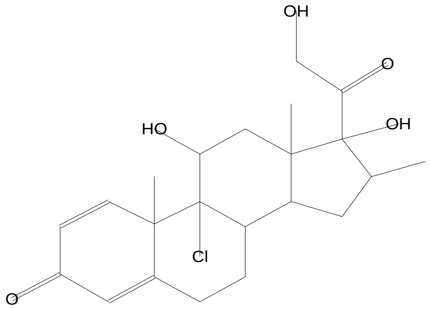

There is no signature data to report.

Library Search Report

Hit Spectrum	Delta	Compound Structure

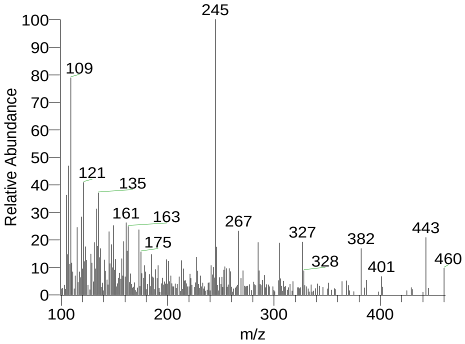

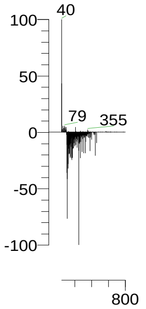

Cholan-24-oic acid, 3-(acetyloxy)-7,12-dioxo-, methyl ester, (3à,5á)-
Formula C27H40O6, MW 460, CAS# 7753-73-3, Entry# 182651
Methyl 3-(acetyloxy)-7,12-dioxocholan-24-oate #

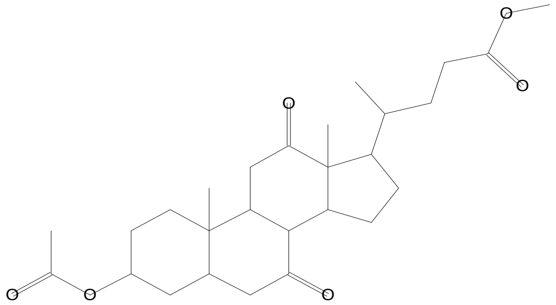

There is no signature data to report.

Library Search Report

RT	Probability	Compound Name	SI	Area %	Area	Molecular Weight	Molecular Formula	Library
80.43	9.62	1-(2-Acetoxyethyl)-3,6-diazahomoadamantan-9-one oxime	428	0.25	788606.97	267	C13H21N3O3	mainlib
80.43	7.76	4,4,6a,6b,8a,11,11,14b-Octamethyl-docosahydropicen-3-ol	423	0.25	788606.97	428	C30H52O	mainlib
80.43	7.76	Lupanol	423	0.25	788606.97	428	C30H52O	Wiley9

Faten-212 #22179 RT: 80.43 AV: 1 RF: 6.00, 3 NL: 4.48E4
F: {0,0} + c EI Full ms [40.00-800.00]

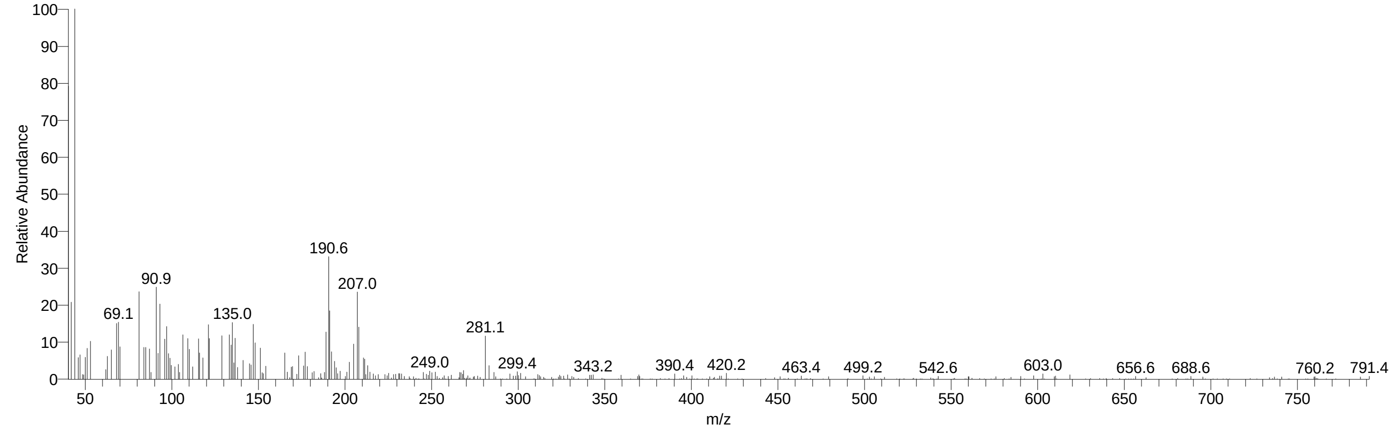

Hit Spectrum | Delta | Compound Structure

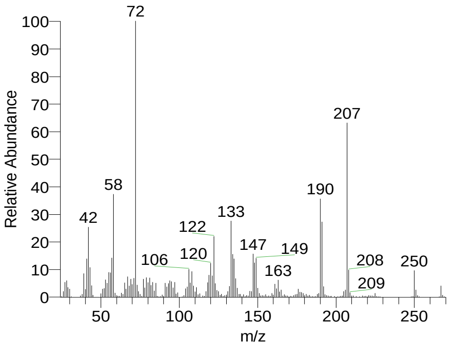

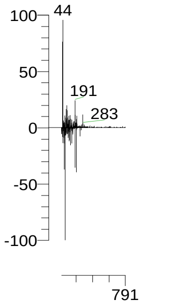

1-(2-Acetoxyethyl)-3,6-diazahomoadamantan-9-one oxime
Formula C13H21N3O3, MW 267, CAS# NA, Entry# 36750

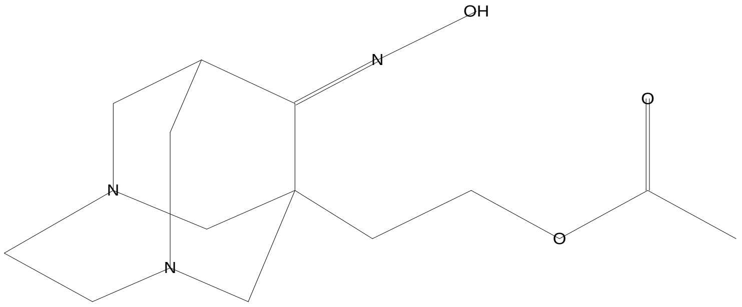

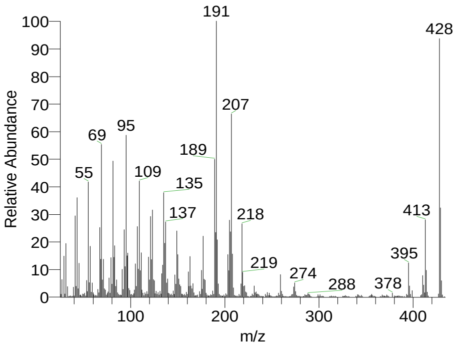

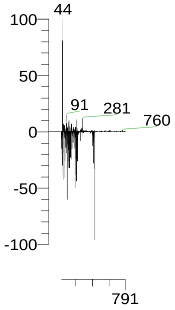

4,4,6a,6b,8a,11,11,14b-Octamethyl-docosahydropicen-3-ol
Formula C30H52O, MW 428, CAS# NA, Entry# 157640
Oleanan-3-ol #

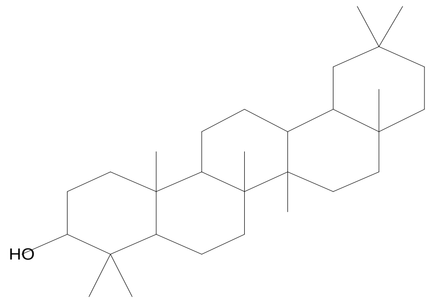

There is no signature data to report.

Library Search Report

Hit Spectrum	Delta	Compound Structure

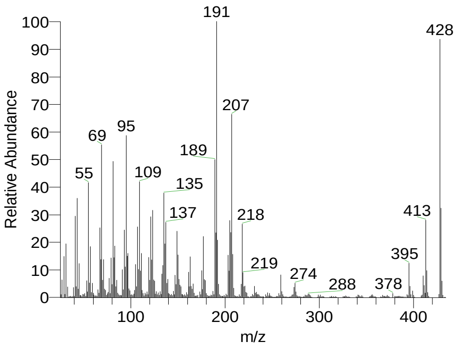

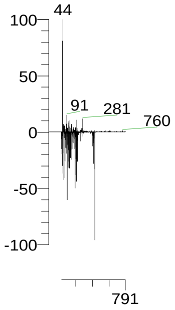

Lupanol
Formula C30H52O, MW 428, CAS# 3186-86-5, Entry# 572155
Lupan-3-ol, (3á)- (CAS)

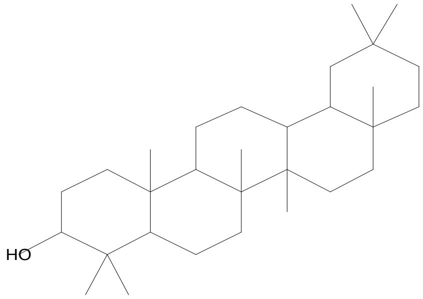

There is no signature data to report.

Library Search Report

RT	Probability	Compound Name	SI	Area %	Area	Molecular Weight	Molecular Formula	Library
80.50	7.68	Ethyl iso-allocholate	411	0.14	442753.53	436	C26H44O5	mainlib
80.50	7.68	Ethyl iso-allocholate	411	0.14	442753.53	436	C26H44O5	Wiley9
80.50	3.96	1-Amino-1-ortho-chlorophenyl-2-(2-quinoxalinyl)ethene	393	0.14	442753.53	281	C16H12ClN3	Wiley9

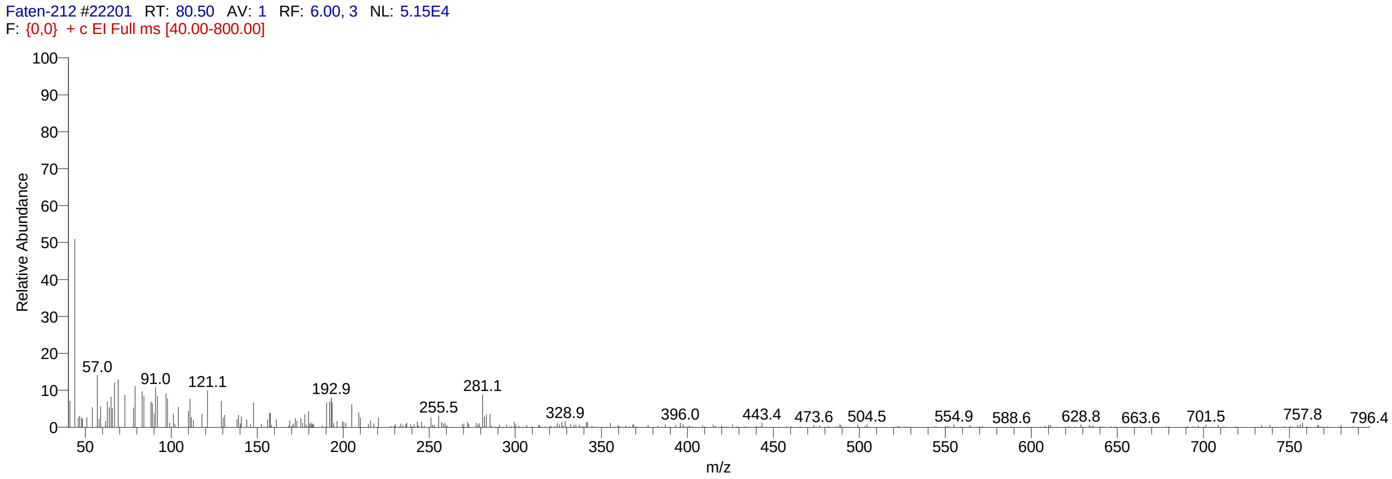

Hit Spectrum	Delta	Compound Structure

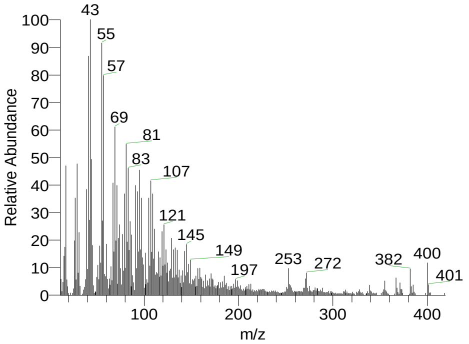

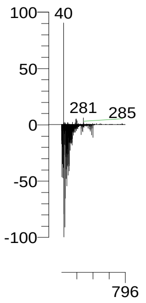

Ethyl iso-allocholate
Formula C26H44O5, MW 436, CAS# NA, Entry# 6654
Ethyl 3,7,12-trihydroxycholan-24-oate #

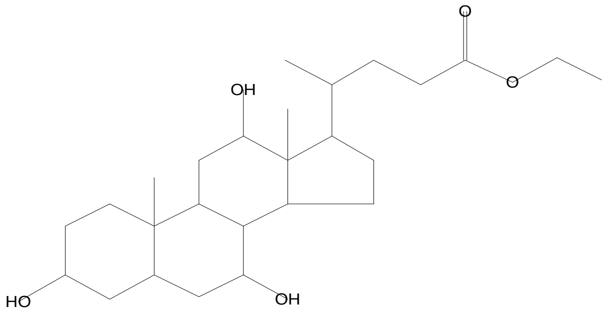

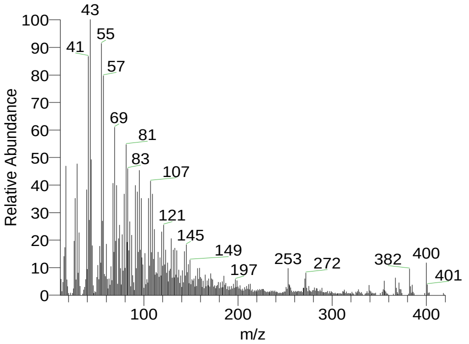

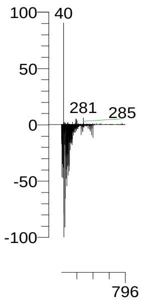

Ethyl iso-allocholate
Formula C26H44O5, MW 436, CAS# NA, Entry# 578772
Ethyl 3,7,12-trihydroxycholan-24-oate

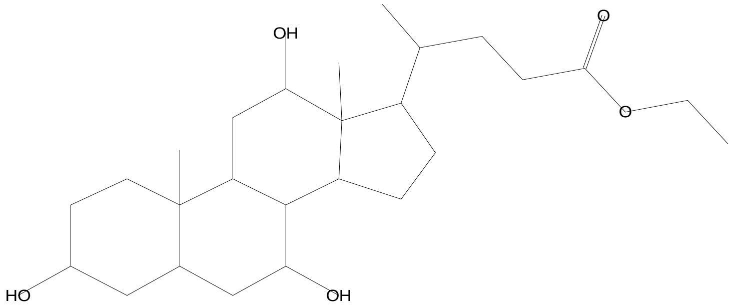

There is no signature data to report.

Library Search Report

Hit Spectrum | Delta | Compound Structure

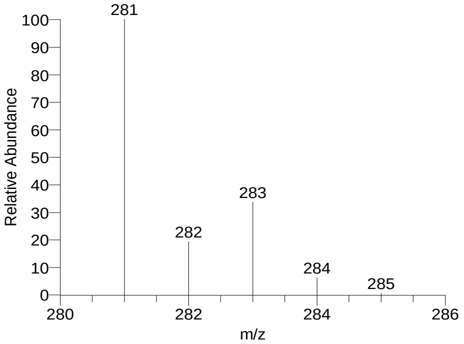

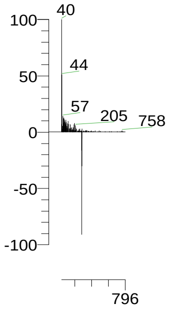

1-Amino-1-ortho-chlorophenyl-2-(2-quinoxalinyl)ethene
Formula C16H12ClN3, MW 281, CAS# 69737-10-6, Entry# 331820
Benzenemethanamine, 2-chloro-à-(2-quinoxalinylmethylene)- (CAS)

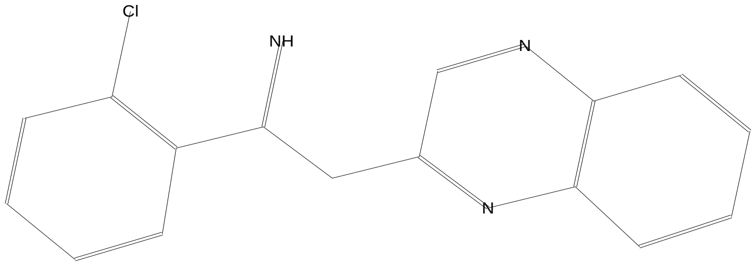

There is no signature data to report.

Library Search Report

RT	Probability	Compound Name	SI	Area %	Area	Molecular Weight	Molecular Formula	Library
81.37	17.62	1H-Indole, 2-(4-chlorobutyl)-3-phenyl-	385	0.14	433851.46	283	C18H18ClN	Wiley9
81.37	13.85	Benzeneacetic acid, 3-methoxy-4-[(trimethylsilyl)oxy]-, trimethylsilyl ester (CAS)	379	0.14	433851.46	326	C15H26O4Si2	Wiley9
81.37	6.30	Pregna-1,4-diene-3,11,20-trione, 17,21-dihydroxy- (CAS)	359	0.14	433851.46	358	C21H26O5	Wiley9

Faten-212 #22457 RT: 81.37 AV: 1 RF: 6.00, 3 NL: 4.32E4
F: {0,0} + c EI Full ms [40.00-800.00]

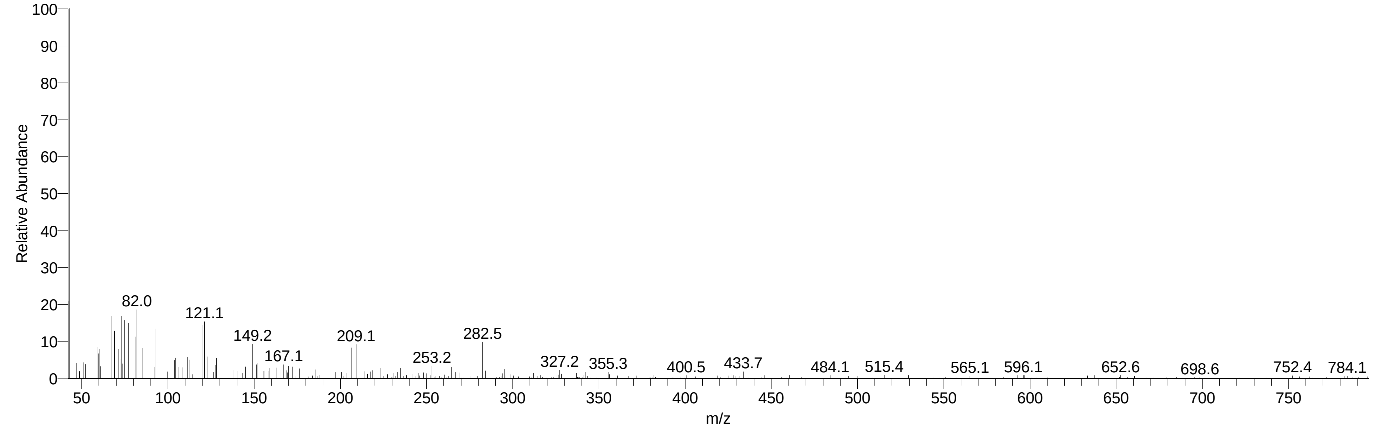

Hit Spectrum | Delta | Compound Structure

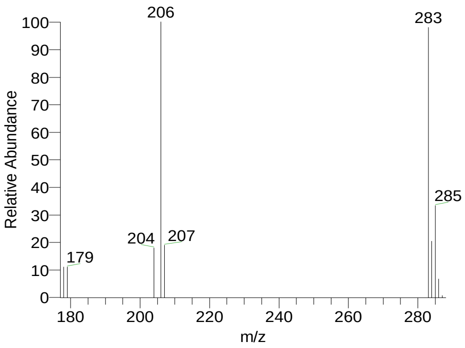

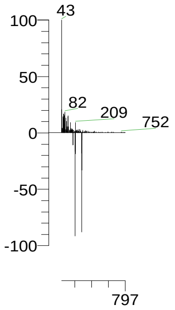

1H-Indole, 2-(4-chlorobutyl)-3-phenyl-
Formula C18H18ClN, MW 283, CAS# 163064-75-3, Entry# 336939
1H-INDOL, 2-(4-CHLOROBUTYL)-3-PHENYL-

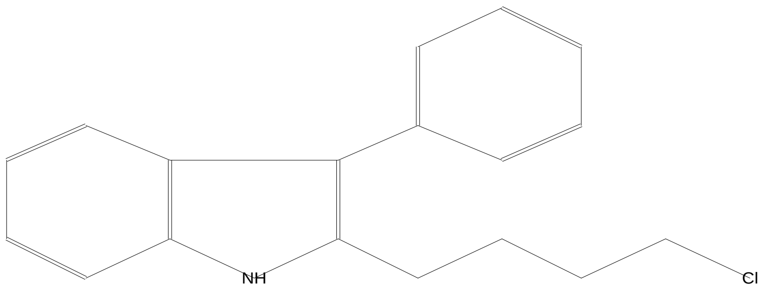

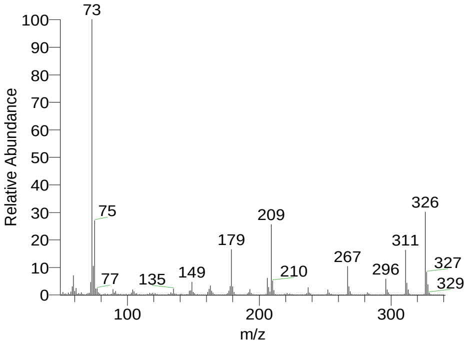

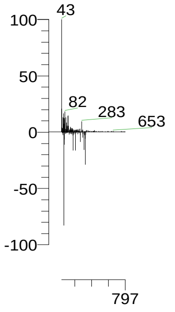

Benzeneacetic acid, 3-methoxy-4-[(trimethylsilyl)oxy]-, trimethylsilyl ester (CAS)
Formula C15H26O4Si2, MW 326, CAS# 37148-61-1, Entry# 427550
HOMOVANILLIC ACID-DITMS

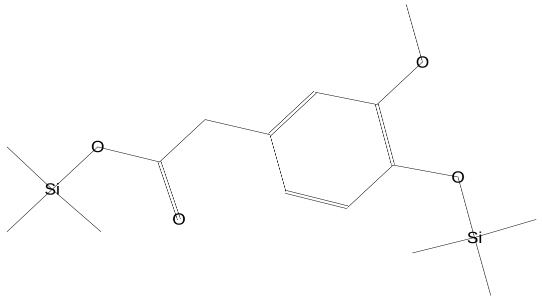

There is no signature data to report.

Library Search Report

Hit Spectrum	Delta	Compound Structure

Pregna-1,4-diene-3,11,20-trione, 17,21-dihydroxy- (CAS)
Formula C21H26O5, MW 358, CAS# 53-03-2, Entry# 485035
Deltra

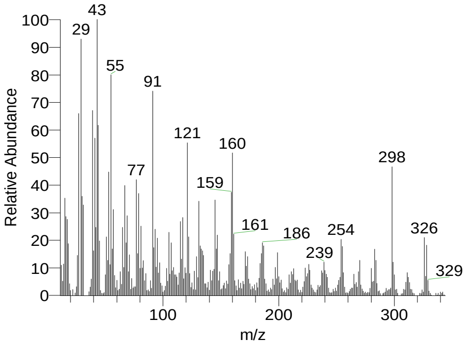

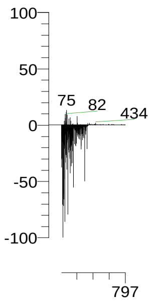

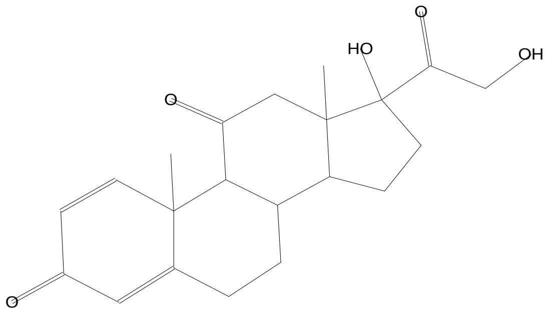

There is no signature data to report.

Library Search Report

RT	Probability	Compound Name	SI	Area %	Area	Molecular Weight	Molecular Formula	Library
81.91	7.88	Benz[e]azulen-3(3aH)-one, 4,6a,7,8,9,10,10a,10b-octahydro-3a,8,10a-trihydroxy-5-(hydroxymethyl)-2,10-dimethyl-, [3aR-(3aà,6aà,8á,10á,10aá,10bá)]-	415	0.19	606472.79	308	C17H24O5	mainlib
81.91	7.88	Benz[e]azulen-3(3aH)-one, 4,6a,7,8,9,10,10a,10b-octahydro-3a,8,10a-trihydroxy-5-(hydroxymethyl)-2,10-dimethyl-, [3aR-(3aà,6aà,8á,10á,10aá,10bá)]-	415	0.19	606472.79	308	C17H24O5	Wiley9
81.91	7.27	7H-Isobenzofuro[4,5-b][1,4]benzodioxepin-11-carboxaldehyde, 1,3-dihydro-1,4-dihydroxy-10-methoxy-5,8-dimethyl-3,7-dioxo-	413	0.19	606472.79	386	C19H14O9	mainlib

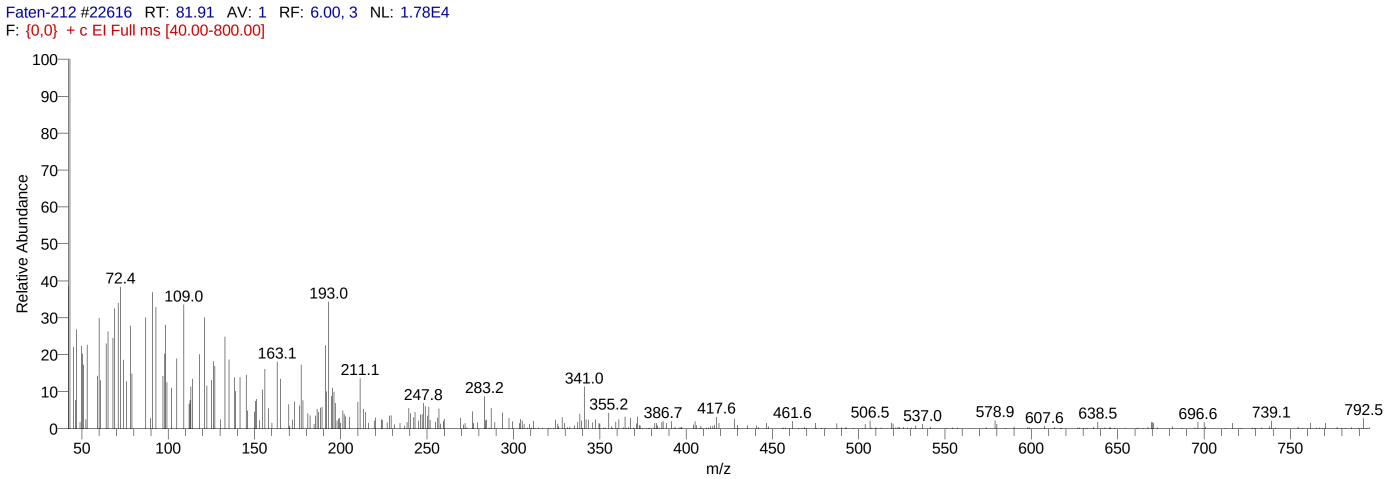

Hit Spectrum	Delta	Compound Structure

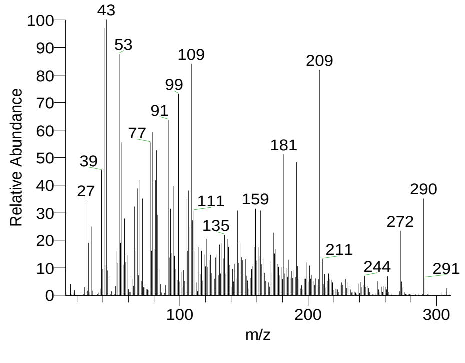

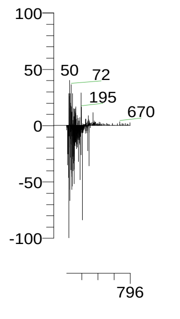

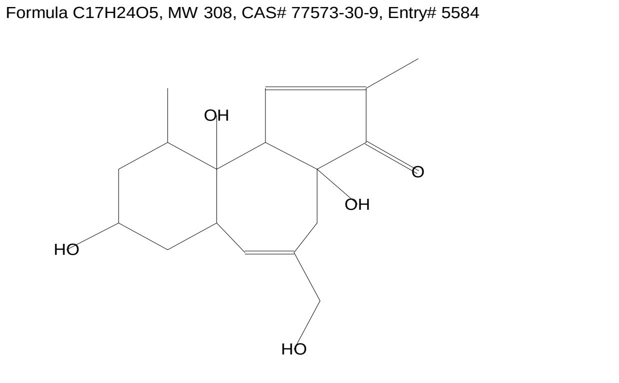

There is no signature data to report.

Library Search Report

Hit Spectrum	Delta	Compound Structure

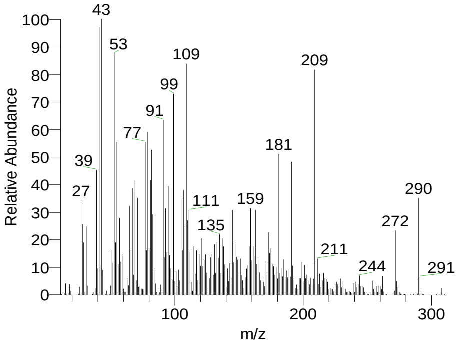

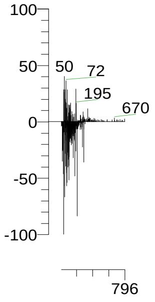

Formula C17H24O5, MW 308, CAS# 77573-30-9, Entry# 391273

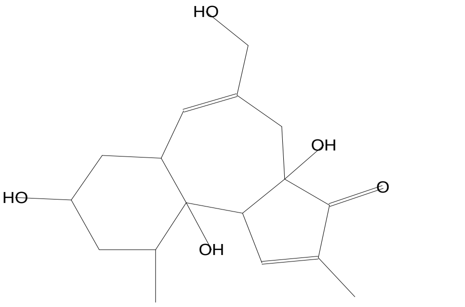

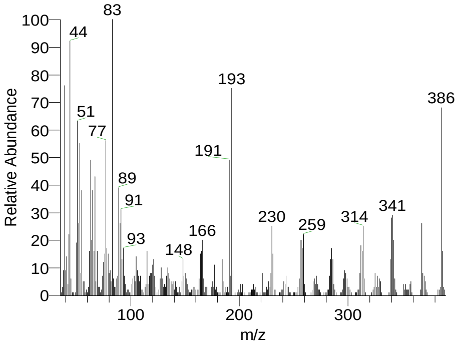

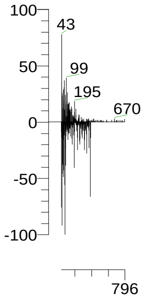

Formula C19H14O9, MW 386, CAS# 20426-13-5, Entry# 47366

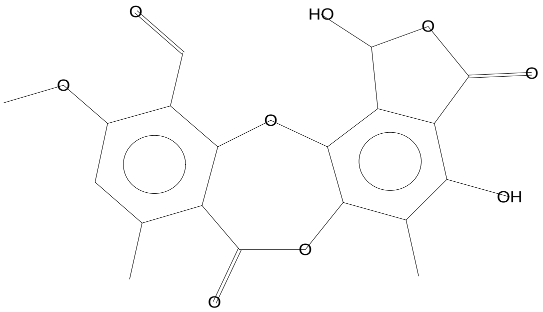

There is no signature data to report.

Library Search Report

RT	Probability	Compound Name	SI	Area %	Area	Molecular Weight	Molecular Formula	Library
82.21	6.18	4-Ethyl-acridan	313	0.16	499798.94	209	C15H15N	mainlib
82.21	6.18	Dibenzo[a,d]bicyclo[3,2,1]-8-azaoctane, N-[diethoxy-cyanomethyl]-	313	0.16	499798.94	392	C23H24N2O4	mainlib
82.21	5.46	Tetradecanoic acid, (3,3a,4,6a,7,8,9,10,10a,10b-decahydro-3a,8,10a-trihydroxy-2,10-dimethyl-3-oxobenz[e]azulen-5-yl)methyl ester, [3aR-(3aà,6aà,8á,10á,10aá,10bá)]-	310	0.16	499798.94	518	C31H50O6	mainlib

Faten-212 #22705 RT: 82.21 AV: 1 RF: 6.00, 3 NL: 4.93E4
F: {0,0} + c EI Full ms [40.00-800.00]

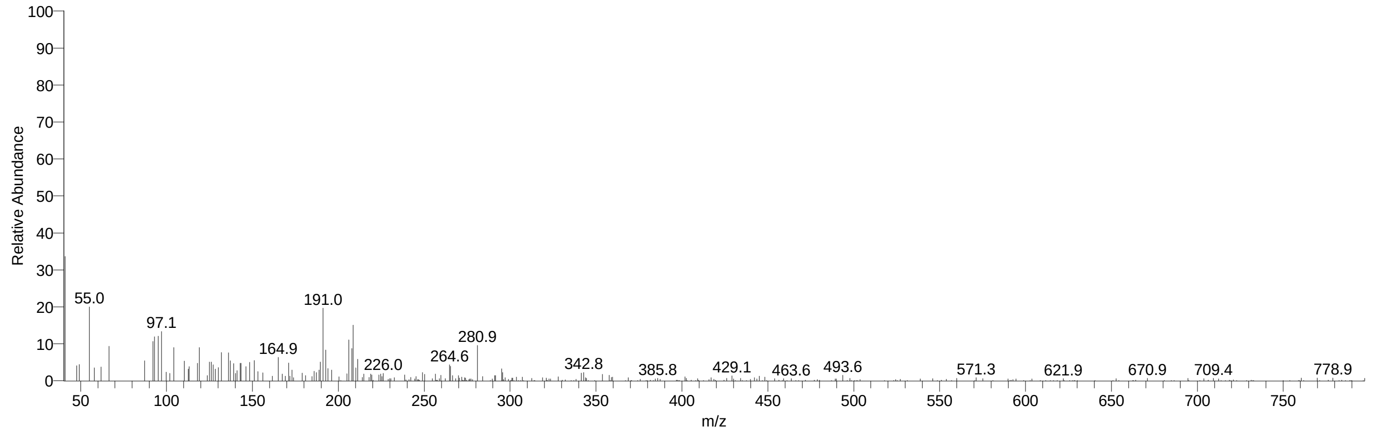

Hit Spectrum	Delta	Compound Structure

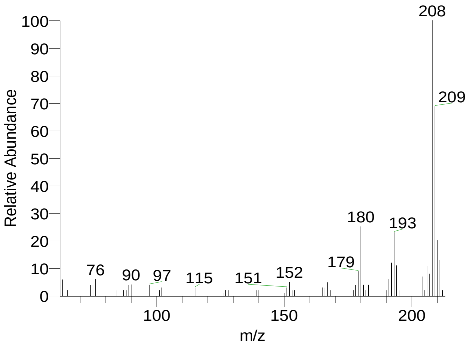

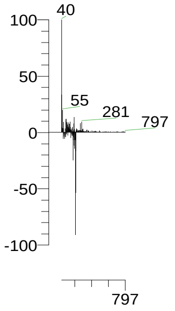

4-Ethyl-acridan
Formula C15H15N, MW 209, CAS# 65753-72-2, Entry# 166988
4-Ethyl-9,10-dihydroacridine #

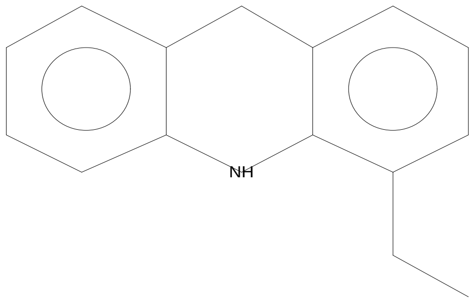

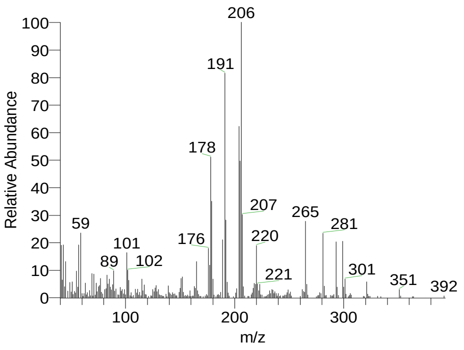

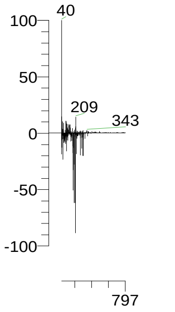

Dibenzo[a,d]bicyclo[3,2,1]-8-azaoctane, N-[diethoxy-cyanomethyl]-
Formula C23H24N2O4, MW 392, CAS# NA, Entry# 166003

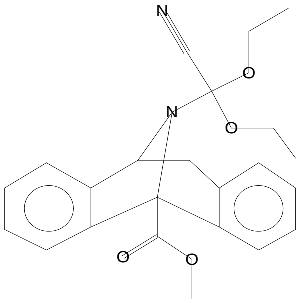

There is no signature data to report.

Library Search Report

Hit Spectrum	Delta	Compound Structure

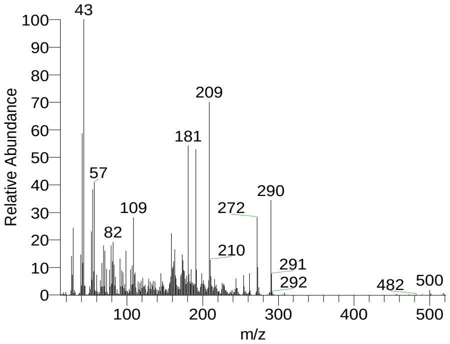

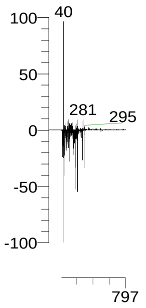

Formula C31H50O6, MW 518, CAS# 77573-28-5, Entry# 12815

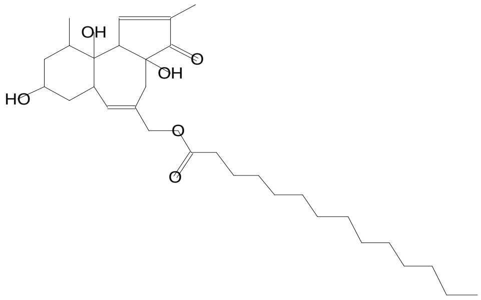

There is no signature data to report.

Library Search Report

RT	Probability	Compound Name	SI	Area %	Area	Molecular Weight	Molecular Formula	Library
82.88	9.68	Stigmast-5-en-3-ol, (3á,24S)- (CAS)	469	0.15	473026.84	414	C29H50O	Wiley9
82.88	5.86	Bufa-20,22-dienolide, 14,15-epoxy-3,11-dihydroxy-, (3á,5á,11à,15á)-	454	0.15	473026.84	400	C24H32O5	mainlib
82.88	5.86	11à-HYDROZYRESIBUFOGENIN	454	0.15	473026.84	400	C24H32O5	Wiley9

Faten-212 #22901 RT: 82.88 AV: 1 RF: 6.00, 3 NL: 9.97E3
F: {0,0} + c EI Full ms [40.00-800.00]

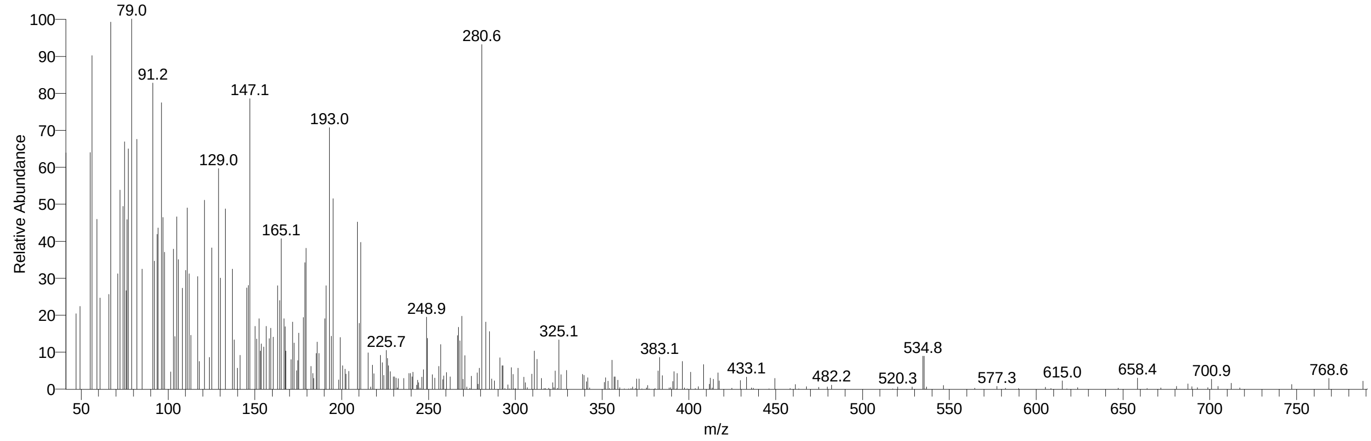

Hit Spectrum	Delta	Compound Structure

Stigmast-5-en-3-ol, (3á,24S)- (CAS)
Formula C29H50O, MW 414, CAS# 83-47-6, Entry# 558546
Clionasterol (CAS)

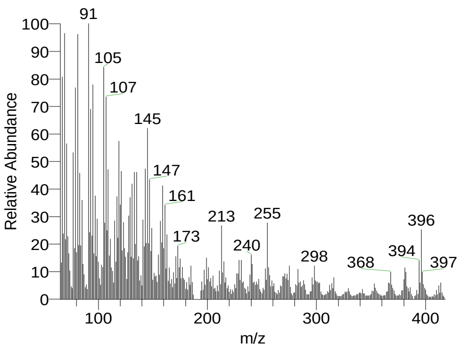

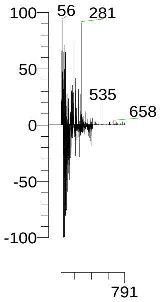

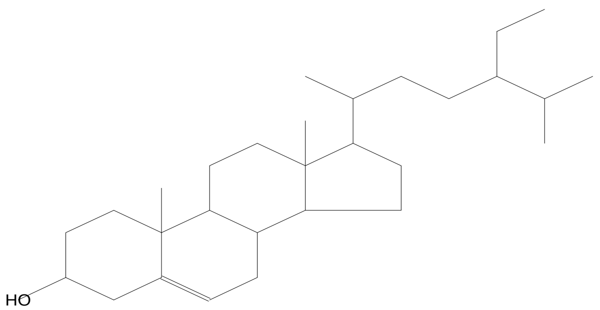

Bufa-20,22-dienolide, 14,15-epoxy-3,11-dihydroxy-, (3á,5á,11à,15á)-
Formula C24H32O5, MW 400, CAS# 39005-15-7, Entry# 75442
11à-Hydroxyresibufogenin

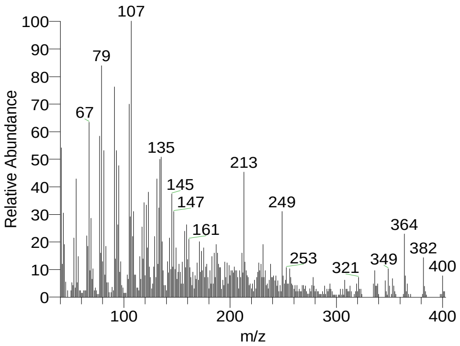

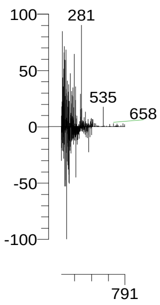

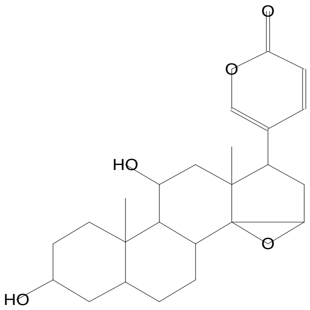

There is no signature data to report.

Library Search Report

Hit Spectrum | Delta | Compound Structure

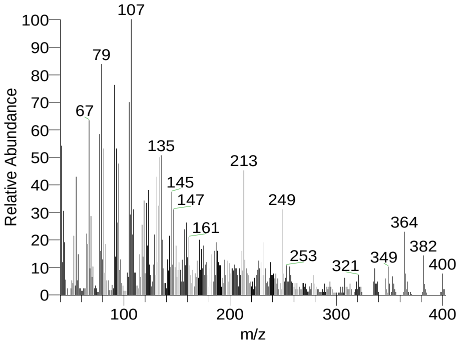

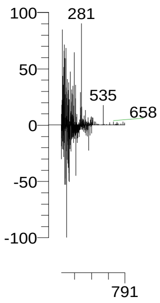

11à-HYDROZYRESIBUFOGENIN
Formula C24H32O5, MW 400, CAS# 39005-15-7, Entry# 542688
Bufa-20,22-dienolide, 14,15-epoxy-3,11-dihydroxy-, (3á,5á,11à,15á)- (CAS)

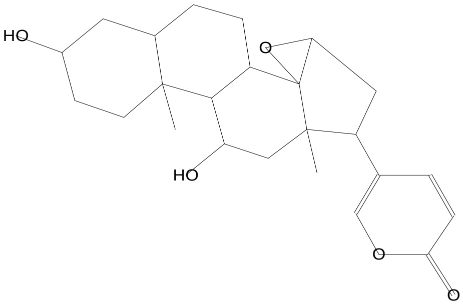

There is no signature data to report.

Library Search Report

RT	Probability	Compound Name	SI	Area %	Area	Molecular Weight	Molecular Formula	Library
83.31	5.46	Benzothiophene-2-carboxamide, 4,5,6,7-tetrahydro-3-acetamido-N-allyl-	360	0.15	474520.07	278	C14H18N2O2S	Wiley9
83.31	5.25	Benzothiophene-2-carboxamide, 4,5,6,7-tetrahydro-3-acetamido-N-allyl-	359	0.15	474520.07	278	C14H18N2O2S	mainlib
83.31	4.43	2,3,5-Tri-O-acetyl-4,6-di-O-methyl-D-mannonitrile	355	0.15	474520.07	331	C14H21NO8	Wiley9

Faten-212 #23028 RT: 83.31 AV: 1 RF: 6.00, 3 NL: 6.05E4
F: {0,0} + c EI Full ms [40.00-800.00]

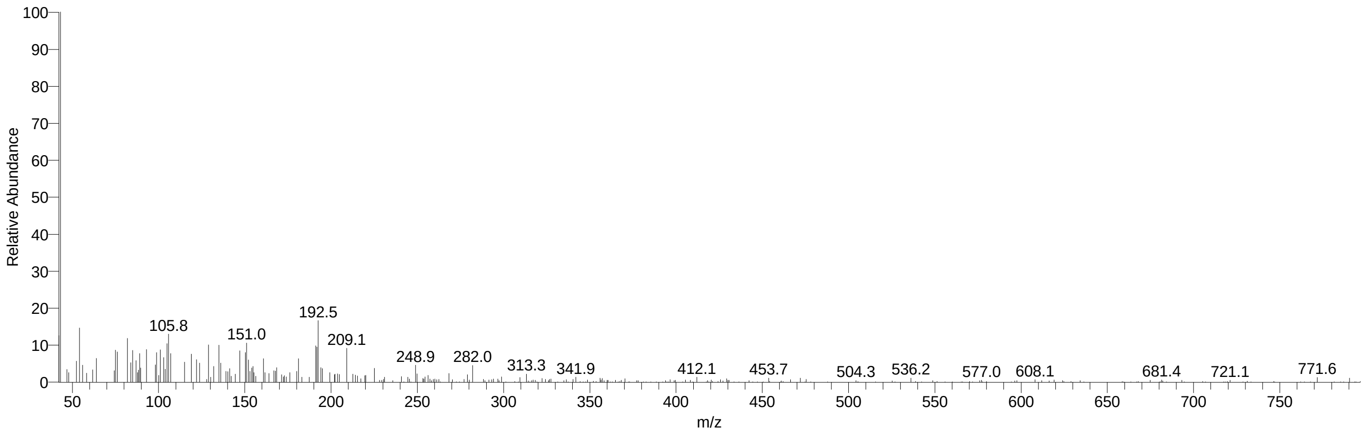

Hit Spectrum	Delta	Compound Structure

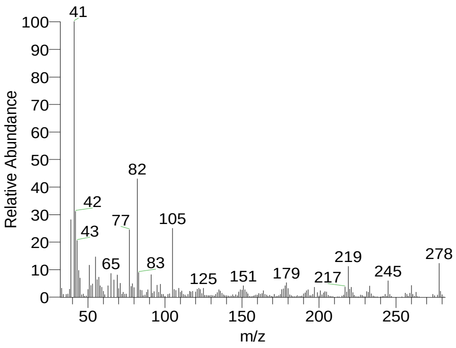

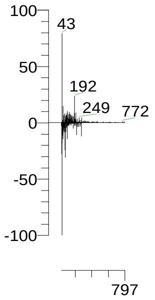

Benzothiophene-2-carboxamide, 4,5,6,7-tetrahydro-3-acetamido-N-allyl-
Formula C14H18N2O2S, MW 278, CAS# NA, Entry# 324179
3-(Acetylamino)-N-allyl-4,5,6,7-tetrahydro-1-benzothiophene-2-carboxamide

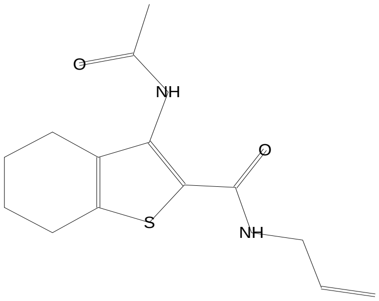

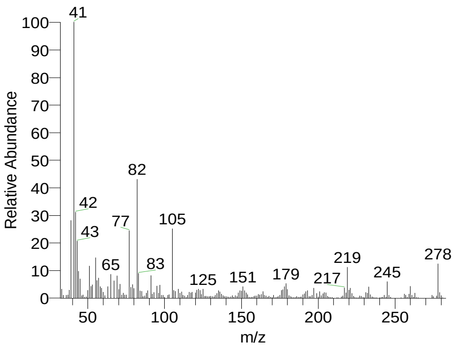

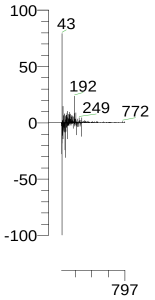

Benzothiophene-2-carboxamide, 4,5,6,7-tetrahydro-3-acetamido-N-allyl-
Formula C14H18N2O2S, MW 278, CAS# NA, Entry# 3473
3-(Acetylamino)-N-allyl-4,5,6,7-tetrahydro-1-benzothiophene-2-carboxamide #

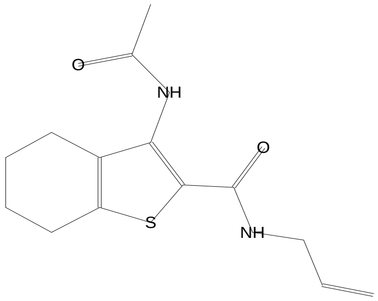

There is no signature data to report.

Library Search Report

Hit Spectrum	Delta	Compound Structure

2,3,5-Tri-O-acetyl-4,6-di-O-methyl-D-mannonitrile
Formula C14H21NO8, MW 331, CAS# 58720-12-0, Entry# 437458

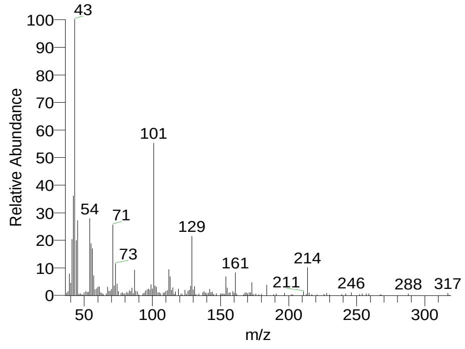

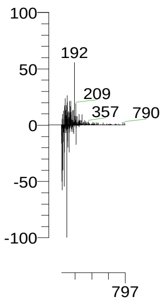

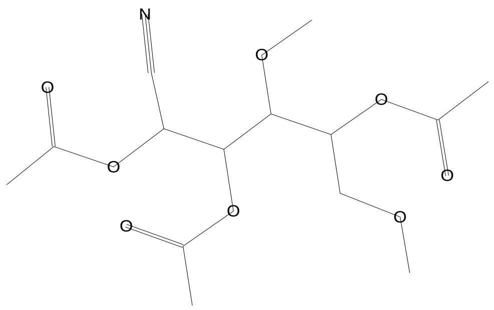

There is no signature data to report.

Library Search Report

RT	Probability	Compound Name	SI	Area %	Area	Molecular Weight	Molecular Formula	Library
83.47	10.71	Stearic acid, 3-(octadecyloxy)propyl ester (CAS)	387	0.13	411201.87	594	C39H78O3	Wiley9
83.47	7.34	Hexasiloxane, 1,1,3,3,5,5,7,7,9,9,11,11-dodecamethyl-	376	0.13	411201.87	430	C12H38O5Si6	mainlib
83.47	7.34	1,1,3,3,5,5,7,7,9,9,11,11-DODECAMETHYL-HEXASILOXANE	376	0.13	411201.87	430	C12H38O5Si6	Wiley9

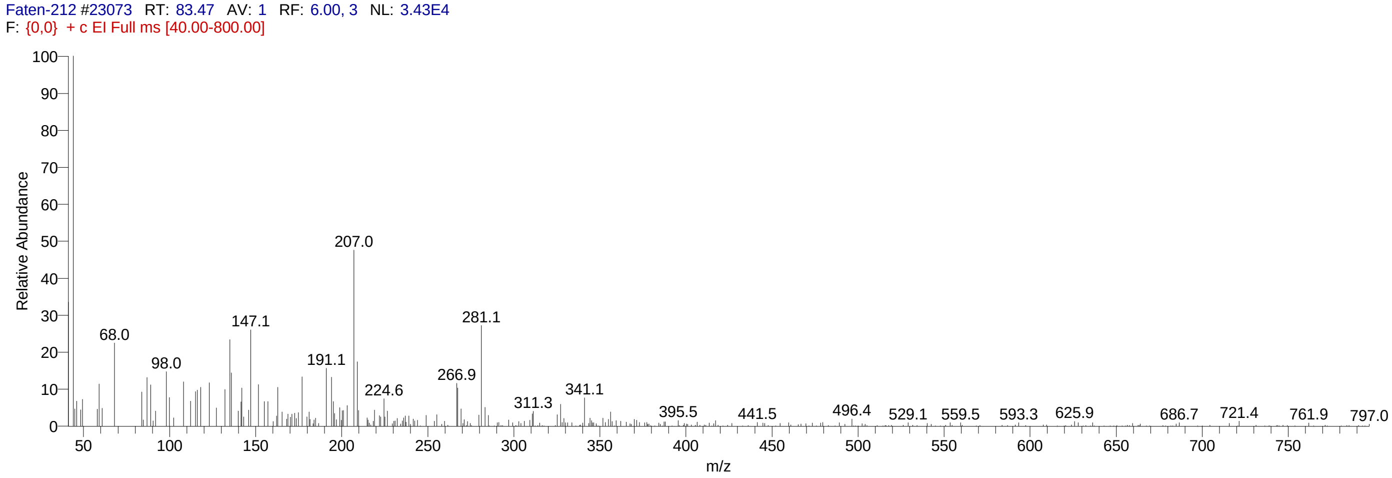

Hit Spectrum	Delta	Compound Structure

Stearic acid, 3-(octadecyloxy)propyl ester (CAS)
Formula C39H78O3, MW 594, CAS# 17367-40-7, Entry# 643308
3-Octadecyloxy-1-O-octadecanoylpropanol

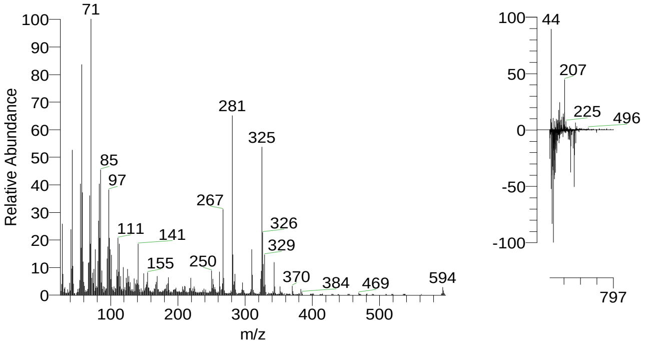

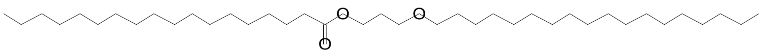

Hexasiloxane, 1,1,3,3,5,5,7,7,9,9,11,11-dodecamethyl-
Formula C12H38O5Si6, MW 430, CAS# 995-82-4, Entry# 39868
1,1,3,3,5,5,7,7,9,9,11,11-Dodecamethylhexasiloxane #

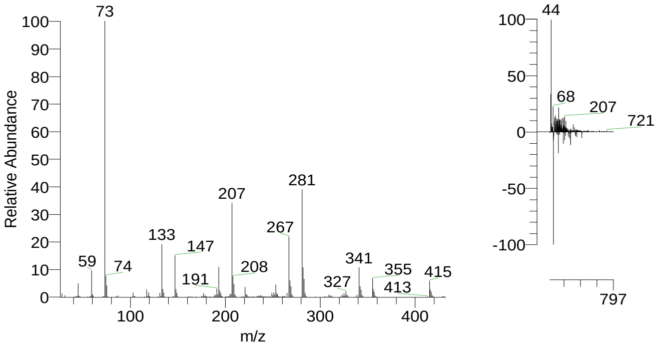

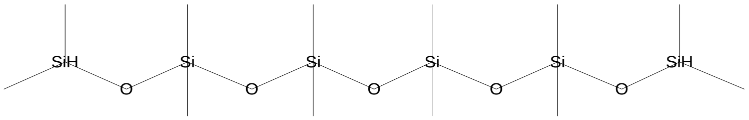

There is no signature data to report.

Library Search Report

Hit Spectrum	Delta	Compound Structure

1,1,3,3,5,5,7,7,9,9,11,11-DODECAMETHYL-HEXASILOXANE
Formula C12H38O5Si6, MW 430, CAS# NA, Entry# 572959

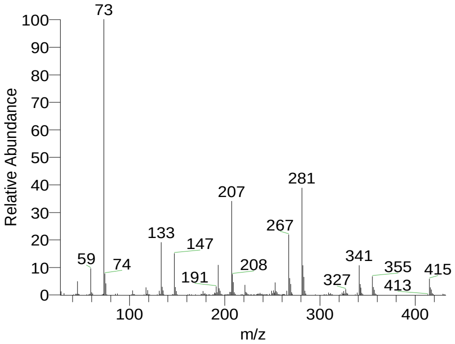

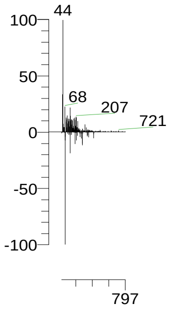

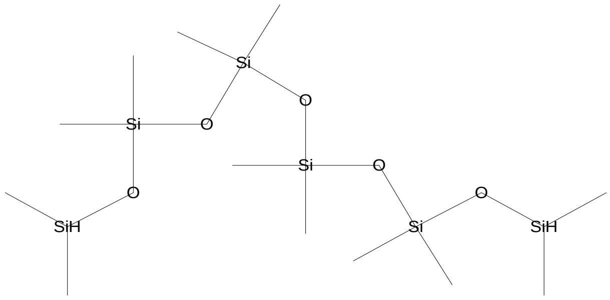

There is no signature data to report.

Library Search Report

RT	Probability	Compound Name	SI	Area %	Area	Molecular Weight	Molecular Formula	Library
84.43	6.04	3,9-Epoxypregn-16-en-20-one, 3-methoxy-7,11,18-triacetoxy-	448	0.15	463104.06	518	C28H38O9	mainlib
84.43	6.04	3,9-Epoxypregn-16-en-20-one, 3-methoxy-7,11,18-triacetoxy-	448	0.15	463104.06	518	C28H38O9	Wiley9
84.43	5.33	Astaxanthin	445	0.15	463104.06	596	C40H52O4	mainlib

Faten-212 #23358 RT: 84.43 AV: 1 RF: 6.00, 3 NL: 1.87E4
F: {0,0} + c EI Full ms [40.00-800.00]

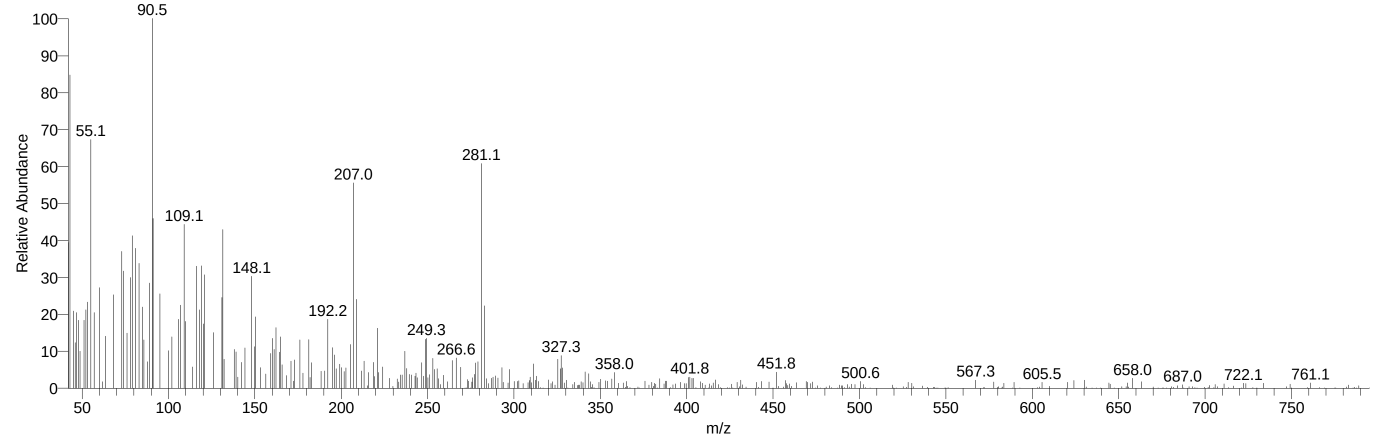

Hit Spectrum	Delta	Compound Structure

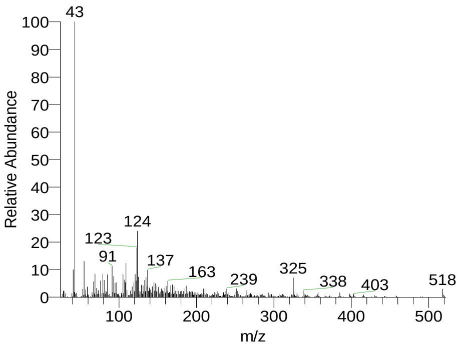

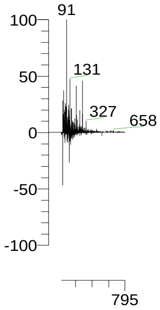

3,9-Epoxypregn-16-en-20-one, 3-methoxy-7,11,18-triacetoxy-
Formula C28H38O9, MW 518, CAS# NA, Entry# 11070

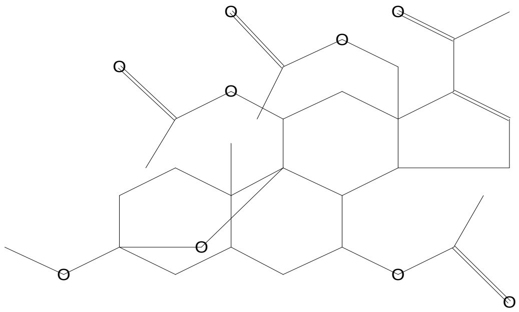

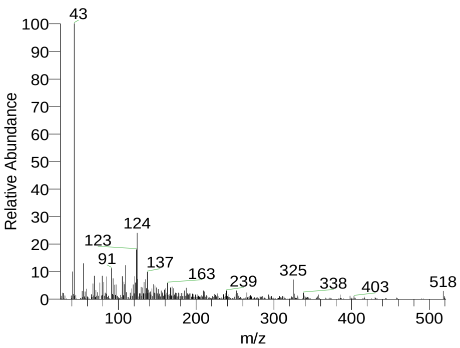

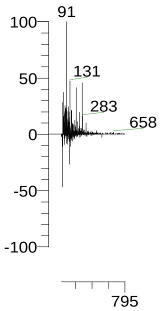

3,9-Epoxypregn-16-en-20-one, 3-methoxy-7,11,18-triacetoxy-
Formula C28H38O9, MW 518, CAS# NA, Entry# 624770

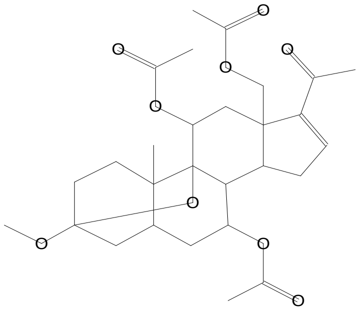

There is no signature data to report.

Library Search Report

Hit Spectrum	Delta	Compound Structure

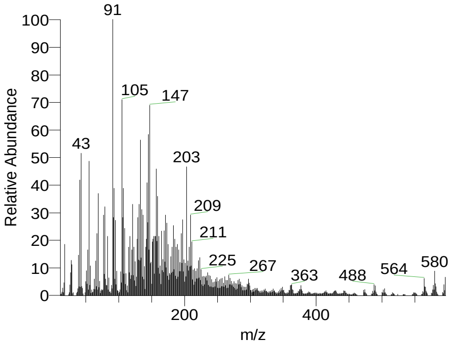

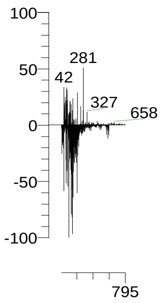

Astaxanthin
Formula C40H52O4, MW 596, CAS# 472-61-7, Entry# 55468
á,á-Carotene-4,4'-dione, 3,3'-dihydroxy-, (3S,3'S)-

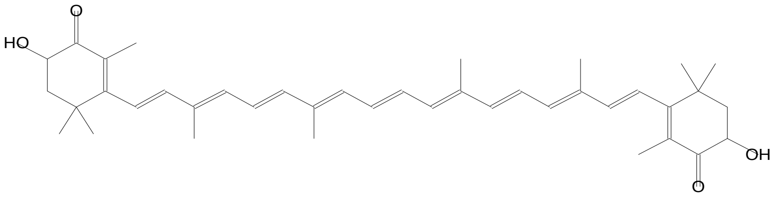

There is no signature data to report.

Library Search Report

RT	Probability	Compound Name	SI	Area %	Area	Molecular Weight	Molecular Formula	Library
84.77	31.85	2-Methoxy-4-trimethylstannyl-3-pyridinecarboxaldehyde	411	0.16	503266.16	301	C10H15NO2Sn	Wiley9
84.77	8.51	Aspidospermidine-3-carboxylic acid, 6,7-didehydro-3,4-dihydroxy-16-methoxy-, methyl ester, (2á,3á,4á,5à,12á,1 9à)-	379	0.16	503266.16	400	C22H28N2O5	mainlib
84.77	8.51	Aspidospermidine-3-carboxylic acid, 6,7-didehydro-3,4-dihydroxy-16-methoxy-, methyl ester, (2á,3á,4á,5à,12á,1 9à)-	379	0.16	503266.16	400	C22H28N2O5	Wiley9

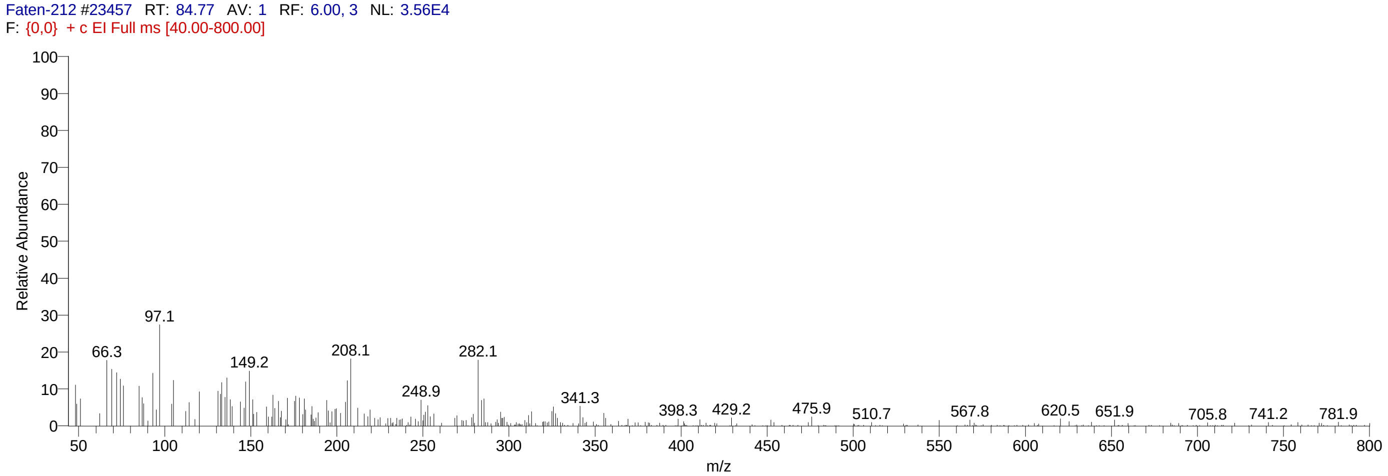

Hit Spectrum | Delta | Compound Structure

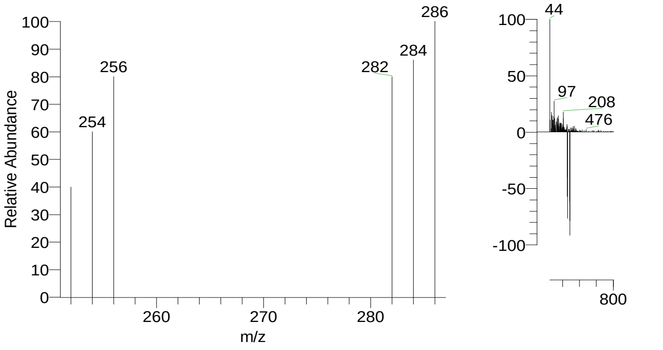

2-Methoxy-4-trimethylstannyl-3-pyridinecarboxaldehyde
Formula C10H15NO2Sn, MW 301, CAS# NA, Entry# 375852

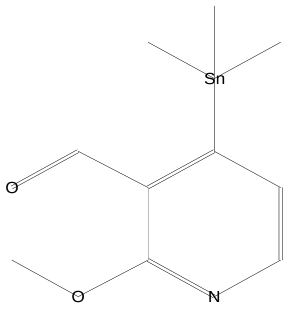

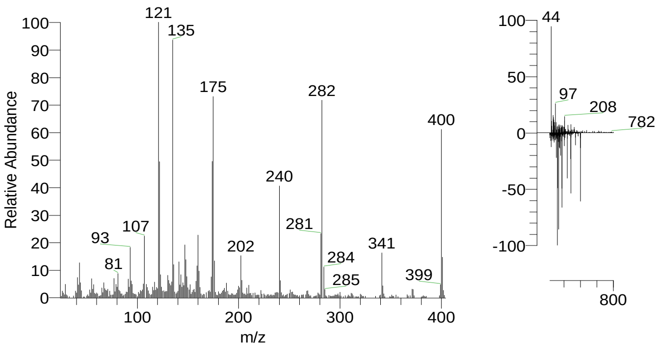

Formula C22H28N2O5, MW 400, CAS# 101043-53-2, Entry# 92267
Methyl 3,4-dihydroxy-16-methoxy-6,7-didehydroaspidospermidine-3-carboxylate #

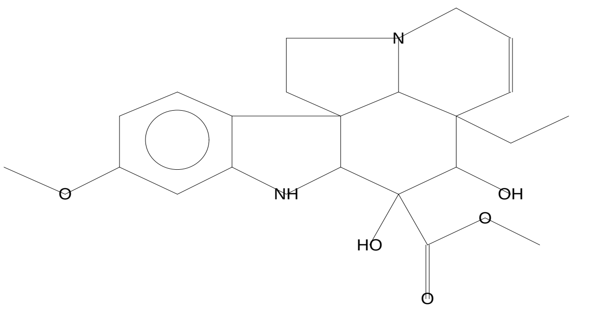

There is no signature data to report.

Library Search Report

Hit Spectrum	Delta	Compound Structure

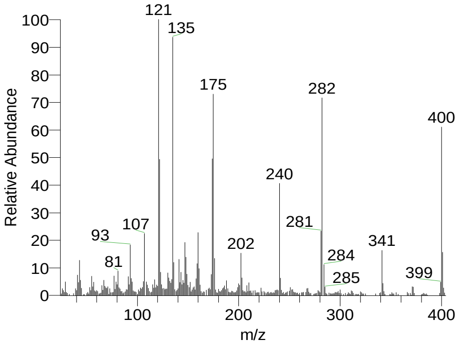

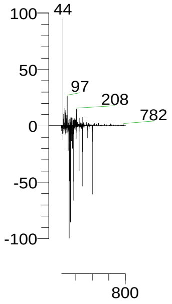

Formula C22H28N2O5, MW 400, CAS# 101043-53-2, Entry# 542424

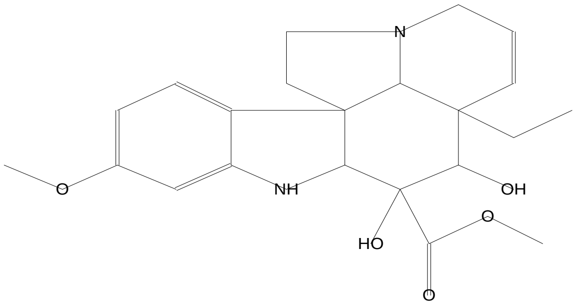

There is no signature data to report.

Library Search Report

RT	Probability	Compound Name	SI	Area %	Area	Molecular Weight	Molecular Formula	Library
85.77	10.67	Endosulfan II	354	0.13	421419.64	404	C9H6Cl6O3S	Wiley9
85.77	8.38	3-Isopropyl-6a,7,10b-trimethyl-8-(2-oxo-2-phenylethyl)dodecahydrobenzo[f]chromene-7-carboxylic acid, methyl ester	348	0.13	421419.64	454	C29H42O4	mainlib
85.77	8.38	3-Isopropyl-6a,7,10b-trimethyl-8-(2-oxo-2-phenylethyl)dodecahydrobenzo[f]chromene-7-carboxylic acid, methyl ester	348	0.13	421419.64	454	C29H42O4	Wiley9

Faten-212 #23752 RT: 85.77 AV: 1 RF: 6.00, 3 NL: 7.79E4
F: {0,0} + c EI Full ms [40.00-800.00]

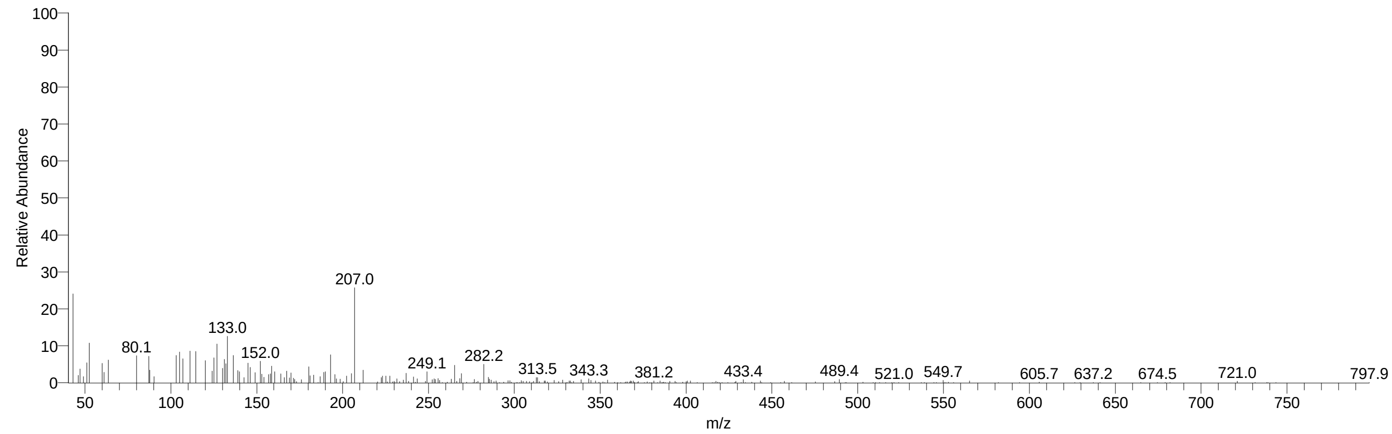

Hit Spectrum	Delta	Compound Structure

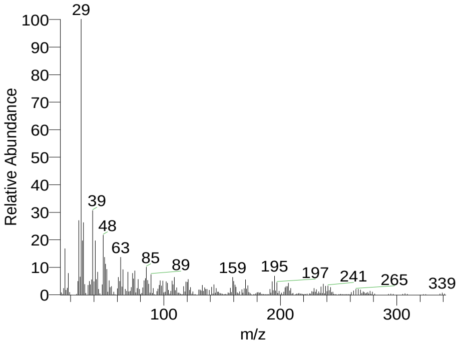

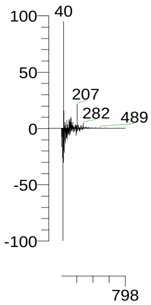

Endosulfan II
Formula C9H6Cl6O3S, MW 404, CAS# 33213-65-9, Entry# 546404

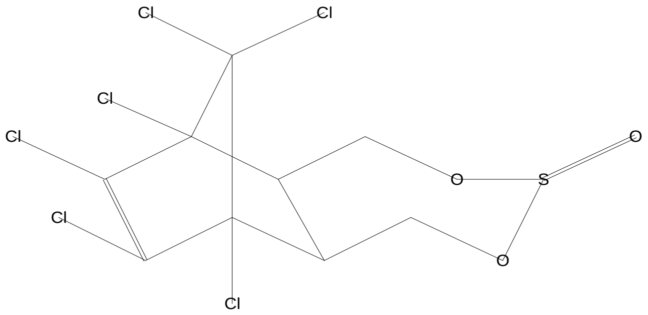

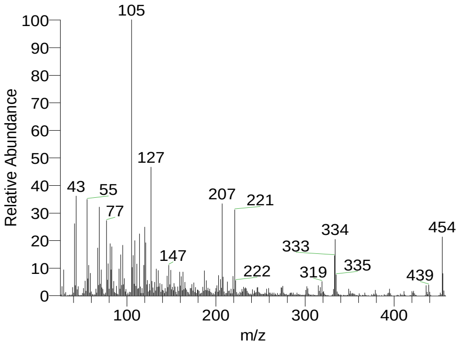

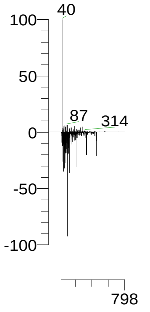

Formula C29H42O4, MW 454, CAS# NA, Entry# 73057

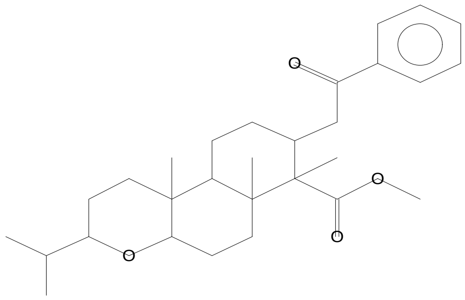

There is no signature data to report.

Library Search Report

Hit Spectrum	Delta	Compound Structure

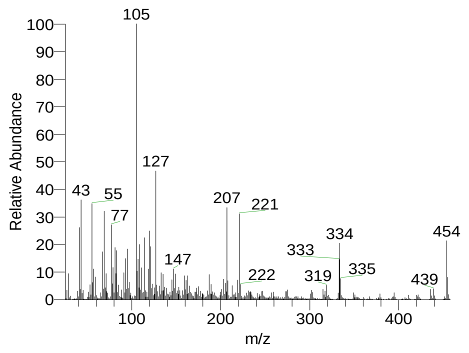

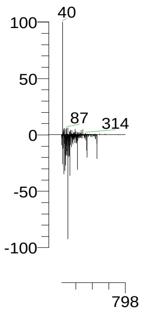

Formula C29H42O4, MW 454, CAS# NA, Entry# 592155

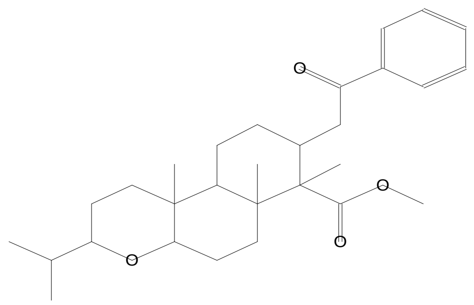

There is no signature data to report.

Library Search Report

RT	Probability	Compound Name	SI	Area %	Area	Molecular Weight	Molecular Formula	Library
87.46	14.37	2,7-Diphenyl-1,6-dioxopyridazino[4,5:2',3']pyrrolo[4',5'-d]pyridazine	435	0.15	484867.97	355	C20H13N5O2	mainlib
87.46	14.37	2,7-Diphenyl-1,6-dioxopyridazino[4,5-2',3']pyrrolo[4',5'-D]pyridazine	435	0.15	484867.97	355	C20H13N5O2	Wiley9
87.46	14.37	1,2-Bis(1-methyl-3,6-diazahomoadamantantylidene-9)hydrazine	435	0.15	484867.97	356	C20H32N6	mainlib

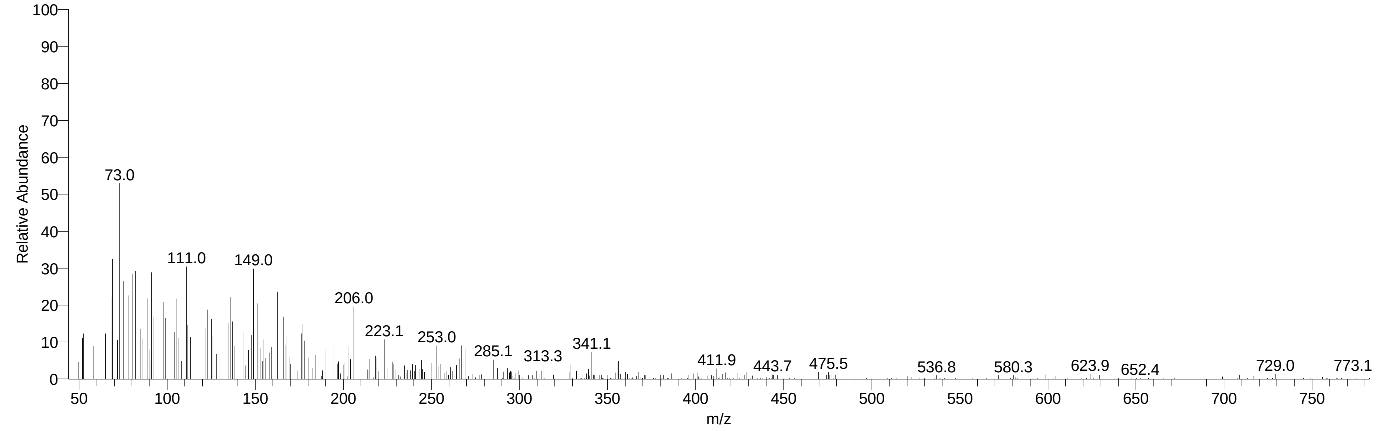

Hit Spectrum | Delta | Compound Structure

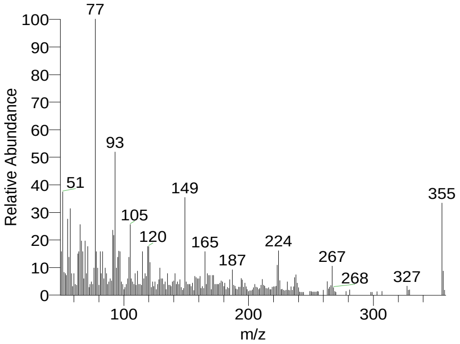

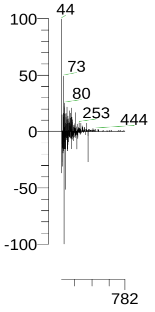

2,7-Diphenyl-1,6-dioxopyridazino[4,5:2',3']pyrrolo[4',5'-d]pyridazine
Formula C20H13N5O2, MW 355, CAS# 91757-06-1, Entry# 42692

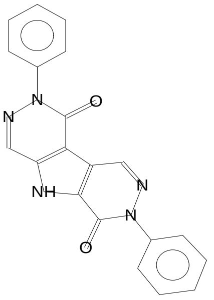

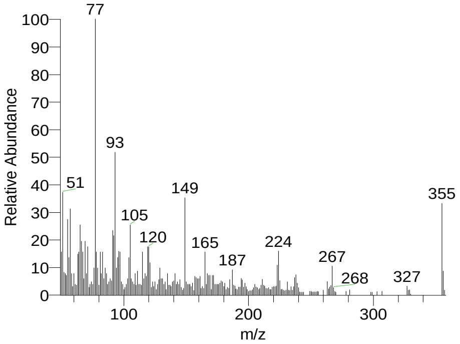

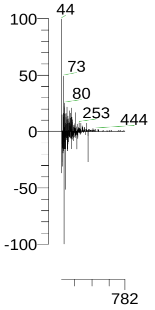

2,7-Diphenyl-1,6-dioxopyridazino[4,5-2',3']pyrrolo[4',5'-D]pyridazine
Formula C20H13N5O2, MW 355, CAS# 91757-06-1, Entry# 480072
2,7-Diphenyl-1,6-dioxopyridazino[4,5:2',3']pyrrolo[4',5'-d]pyridazine

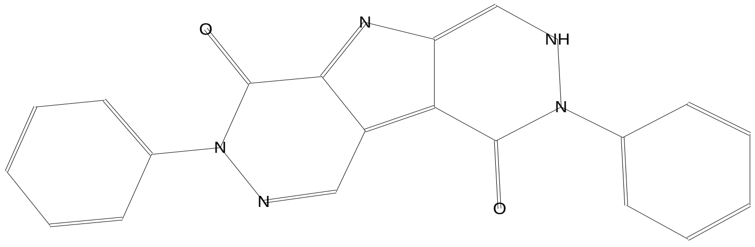

There is no signature data to report.

Library Search Report

Hit Spectrum	Delta	Compound Structure

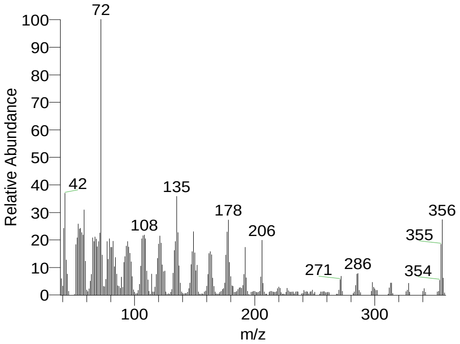

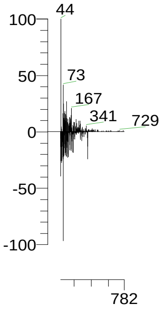

1,2-Bis(1-methyl-3,6-diazahomoadamantantylidene-9)hydrazine
Formula C20H32N6, MW 356, CAS# NA, Entry# 36119

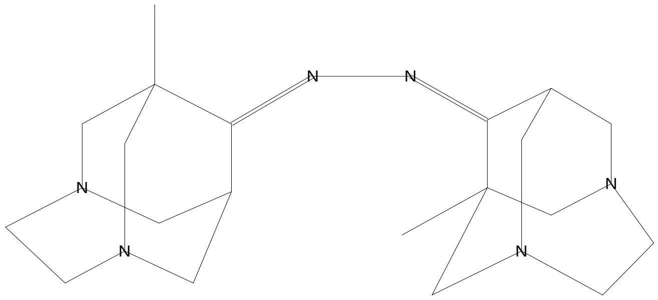

There is no signature data to report.

Library Search Report

RT	Probability	Compound Name	SI	Area %	Area	Molecular Weight	Molecular Formula	Library
87.68	11.74	1H-Cyclopropa[3,4]benz[1,2-e]azulene-5,7b,9,9a-tetrol, 1a,1b,4,4a,5,7a,8,9-octahydro-3-(hydroxymethyl)-1,1,6,8-tetramethyl-, 9,9a-diacetate, [1aR-(1aà,1bá,4aá,5á,7aà,7bà,8à,9á,9aà)]-	416	0.14	454109.06	434	C24H34O7	mainlib
87.68	11.74	1H-Cyclopropa[3,4]benz[1,2-e]azulene-5,7b,9,9a-tetrol, 1a,1b,4,4a,5,7a,8,9-octahydro-3-(hydroxymethyl)-1,1,6,8-tetramethyl-, 9,9a-diacetate,[1aR-(1aà,1bá,4aá,5á,7aà,7bà,8à,9á,9aà)]-	416	0.14	454109.06	434	C24H34O7	Wiley9
87.68	8.28	Pregnan-20-one, 3,11-dihydroxy-17,21-bis[(trimethylsilyl)oxy]-, O-methyloxime, (3à,5á,11á)-	406	0.14	454109.06	539	C28H53NO5Si2	mainlib

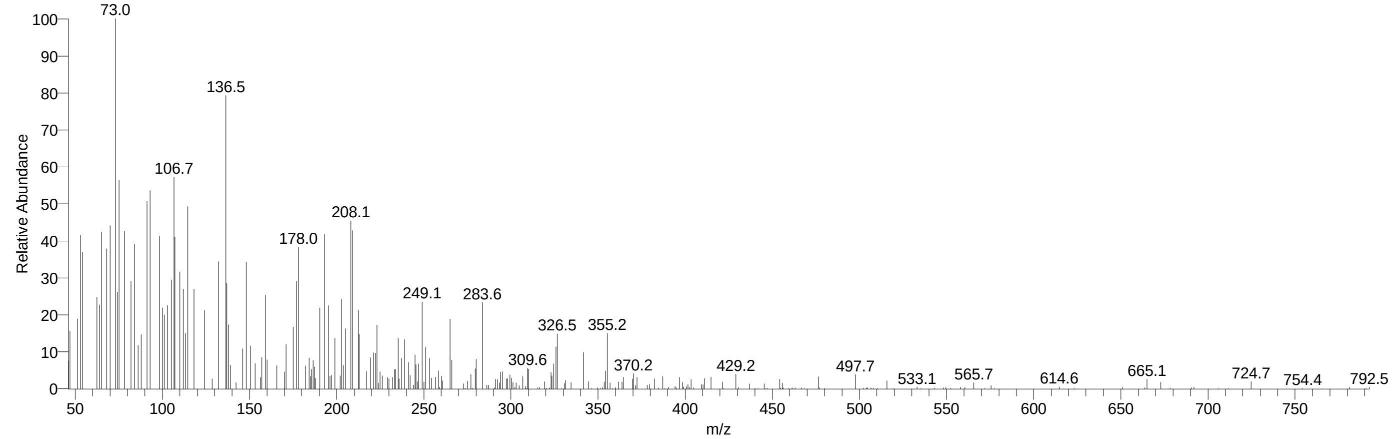

Hit Spectrum	Delta	Compound Structure

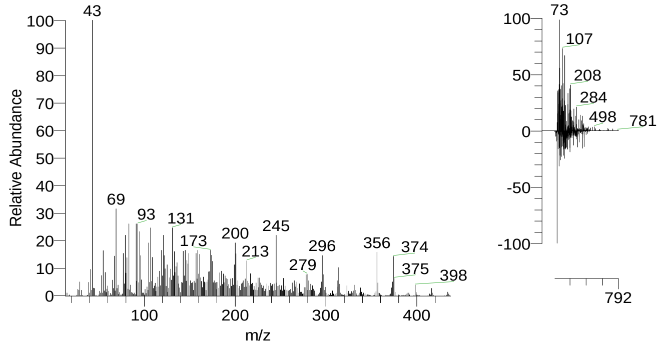

Formula C24H34O7, MW 434, CAS# 77508-65-7, Entry# 8103

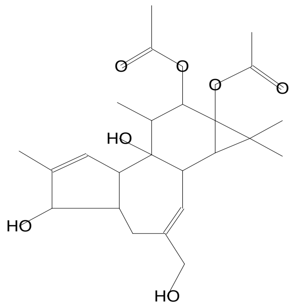

There is no signature data to report.

Library Search Report

Hit Spectrum	Delta	Compound Structure

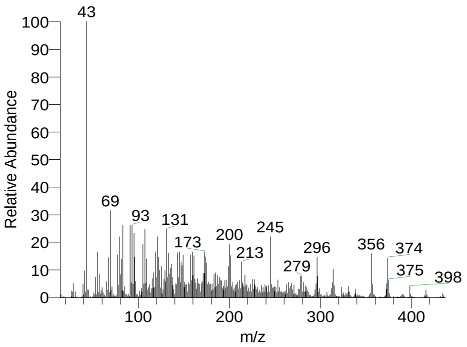

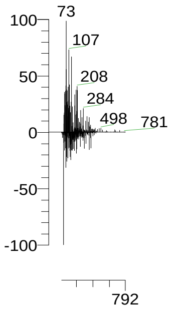

Formula C24H34O7, MW 434, CAS# 77508-65-7, Entry# 577059

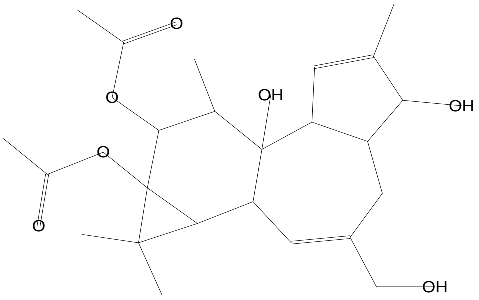

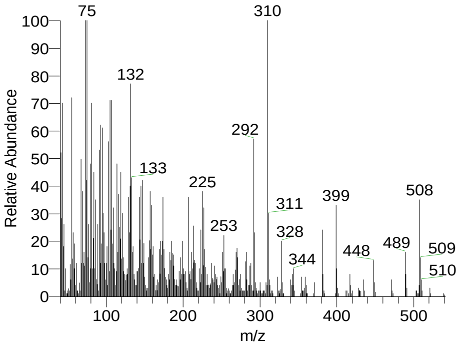

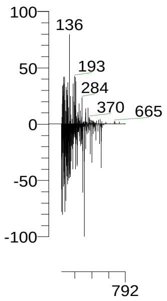

Pregnan-20-one, 3,11-dihydroxy-17,21-bis[(trimethylsilyl)oxy]-, O-methyloxime, (3à,5á,11á)-
Formula C28H53NO5Si2, MW 539, CAS# 57305-36-9, Entry# 37867
3,11-Dihydroxy-17,21-bis[(trimethylsilyl)oxy]pregnan-20-one o-methyloxime #

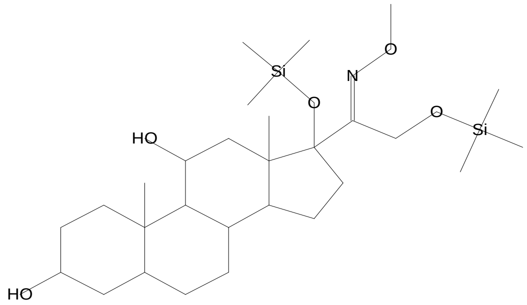

There is no signature data to report.

Library Search Report

RT	Probability	Compound Name	SI	Area %	Area	Molecular Weight	Molecular Formula	Library
88.69	17.59	Spherodenon	370	0.16	497594.00	582	C41H58O2	Wiley9
88.69	13.47	3,5-Dimethyl-2,6-bis(trimethylsiloxy)pyridine	363	0.16	497594.00	283	C13H25NO2Si2	Wiley9
88.69	17.59	.psi.,.psi.-Carotene, 3,4-didehydro-1,2,7',8'-tetrahydro-1-methoxy-2-oxo-	362	0.16	497594.00	582	C41H58O2	mainlib

Faten-212 #24608 RT: 88.69 AV: 1 RF: 6.00, 3 NL: 2.38E4
F: {0,0} + c EI Full ms [40.00-800.00]

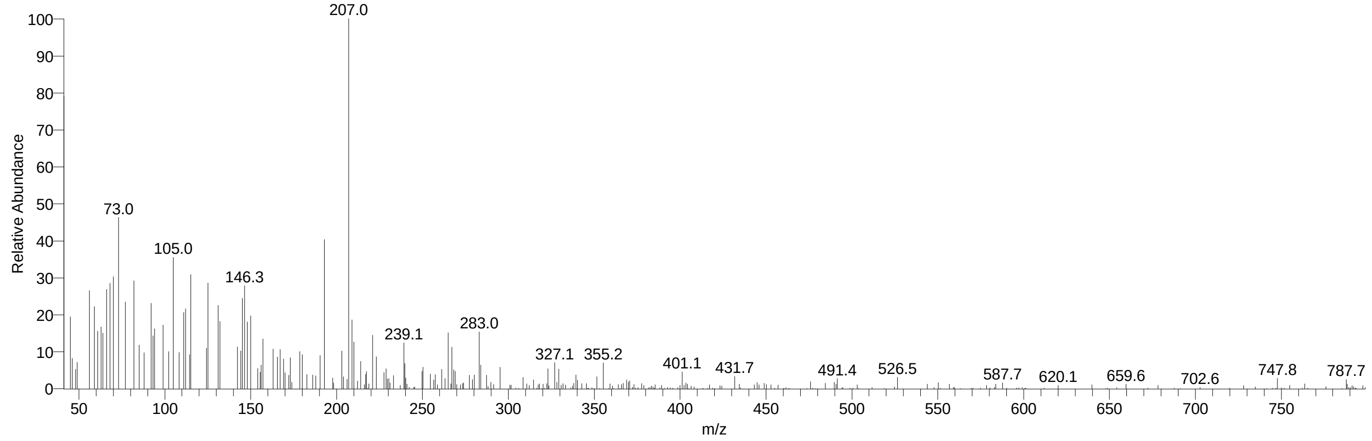

Hit Spectrum | Delta | Compound Structure

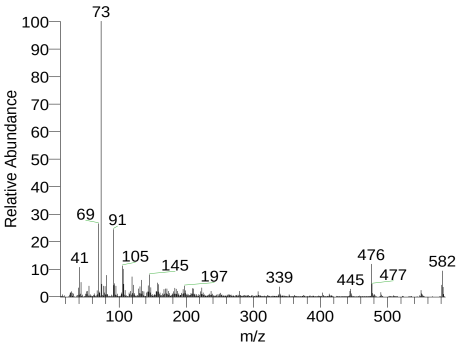

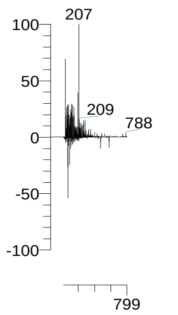

Spherodenon
Formula C41H58O2, MW 582, CAS# 13836-70-9, Entry# 641148
.psi.,.psi.-Carotene, 3,4-didehydro-1,2,7',8'-tetrahydro-1-methoxy-2-oxo- (CAS)

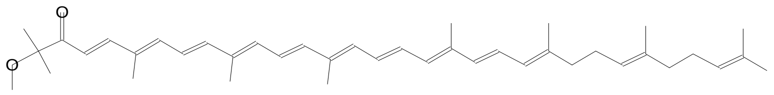

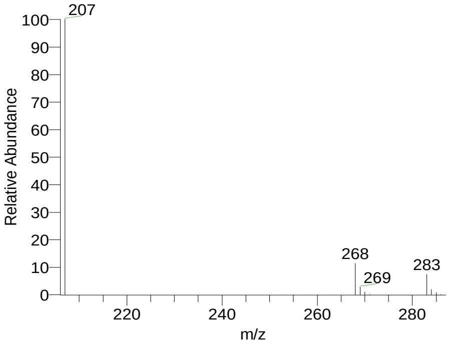

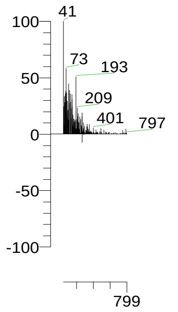

3,5-Dimethyl-2,6-bis(trimethylsiloxy)pyridine
Formula C13H25NO2Si2, MW 283, CAS# NA, Entry# 336114

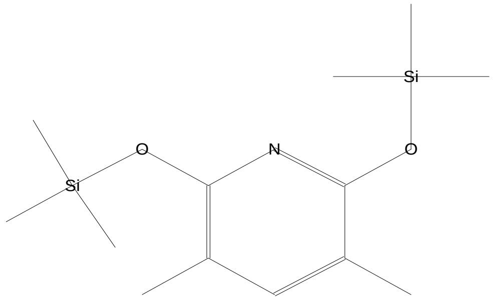

There is no signature data to report.

Library Search Report

Hit Spectrum	Delta	Compound Structure

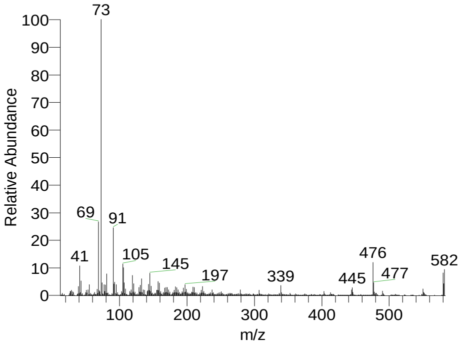

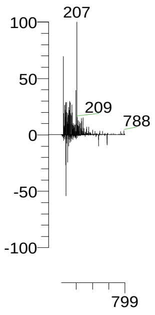

.psi.,.psi.-Carotene, 3,4-didehydro-1,2,7',8'-tetrahydro-1-methoxy-2-oxo-
Formula C41H58O2, MW 582, CAS# 13836-70-9, Entry# 37545
Lycopene, 3,4-didehydro-1,2,7',8'-tetrahydro-1-methoxy-2-oxo-, all-trans-

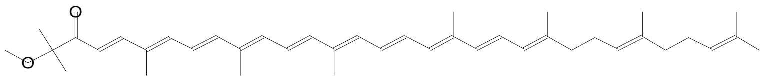

There is no signature data to report.

Library Search Report

RT	Probability	Compound Name	SI	Area %	Area	Molecular Weight	Molecular Formula	Library
88.82	21.17	Butanoic acid, 1a,2,5,5a,6,9,10,10a-octahydro-5a-hydroxy-4-(hydroxymethyl)-1,1,7,9-tetramethyl-6,11-dioxo-1H-2,8a-methanocyclopenta[a]cyclopropa[e]cyclodecen-5-yl ester, [1aR-(1aà,2à,5á,5aá,8aà,9à,10aà)]-	428	0.18	580591.14	416	C24H32O6	Wiley9
88.82	14.52	Dodecanoic acid, 1a,2,5,5a,6,9,10,10a-octahydro-5a-hydroxy-4-(hydroxymethyl)-1,1,7,9-tetramethyl-6,11-dioxo-1H-2,8a-methanocyclopenta[a]cyclopropa[e]cyclodecen-5-yl ester, [1aR-(1aà,2à,5á,5aá,8aà,9à,10aà)]-	417	0.18	580591.14	528	C32H48O6	mainlib
88.82	14.52	Dodecanoic acid, 1a,2,5,5a,6,9,10,10a-octahydro-5a-hydroxy-4-(hydroxymethyl)-1,1,7,9-tetramethyl-6,11-dioxo-1H-2,8a-methanocyclopenta[a]cyclopropa[e]cyclodecen-5-yl ester, [1aR-(1aà,2à,5á,5aá,8aà,9à,10aà)]-	416	0.18	580591.14	528	C32H48O6	Wiley9

Faten-212 #24646 RT: 88.82 AV: 1 RF: 6.00, 3 NL: 4.31E4
F: {0,0} + c EI Full ms [40.00-800.00]

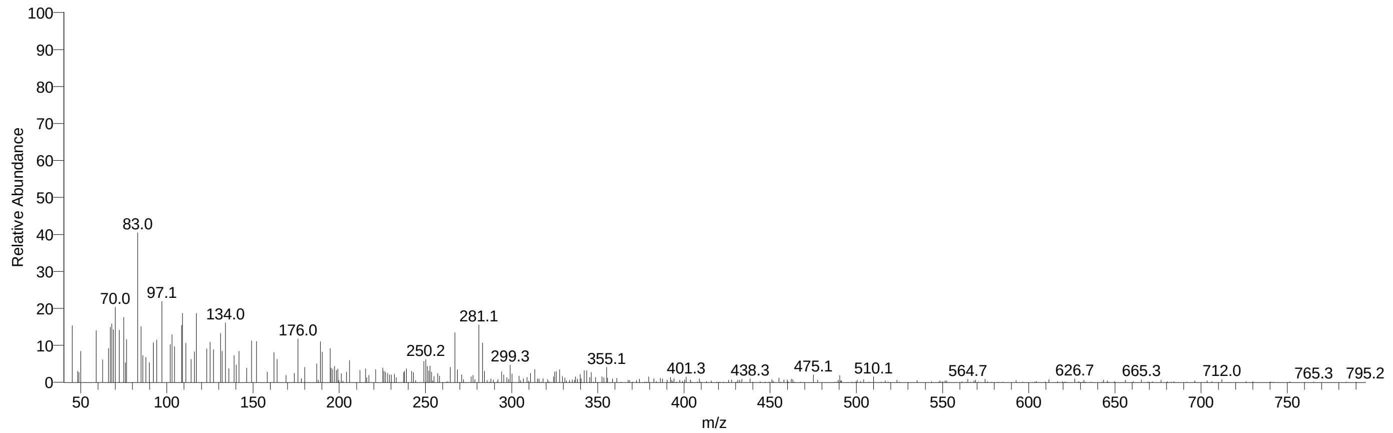

Hit Spectrum | Delta | Compound Structure

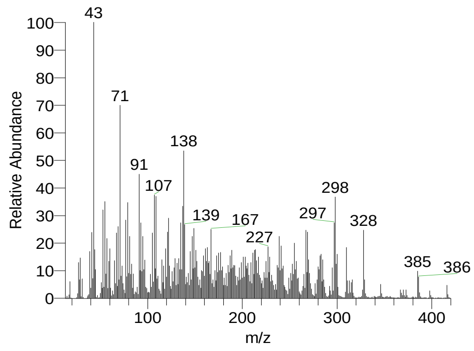

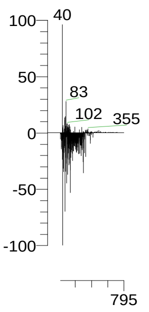

Formula C24H32O6, MW 416, CAS# 77508-70-4, Entry# 560211

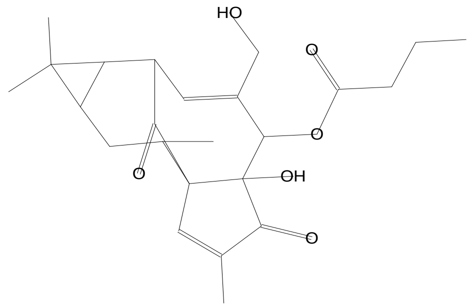

There is no signature data to report.

Library Search Report

Hit Spectrum	Delta	Compound Structure

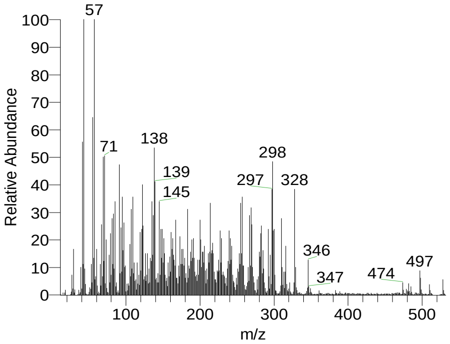

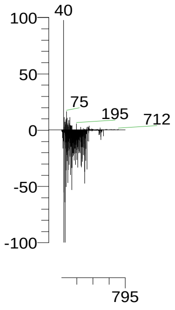

Formula C32H48O6, MW 528, CAS# 77508-68-0, Entry# 7337

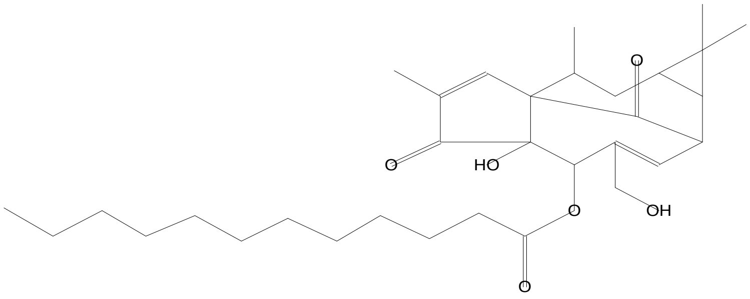

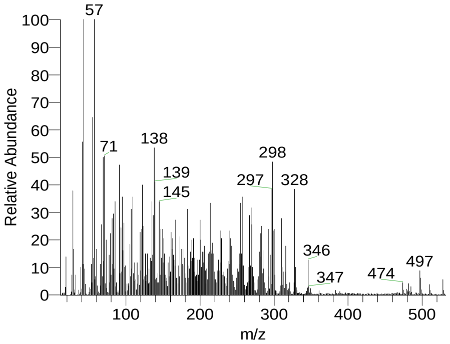

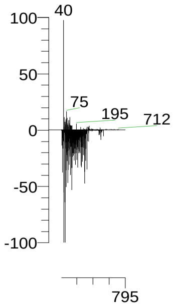

Formula C32H48O6, MW 528, CAS# 77508-68-0, Entry# 628121

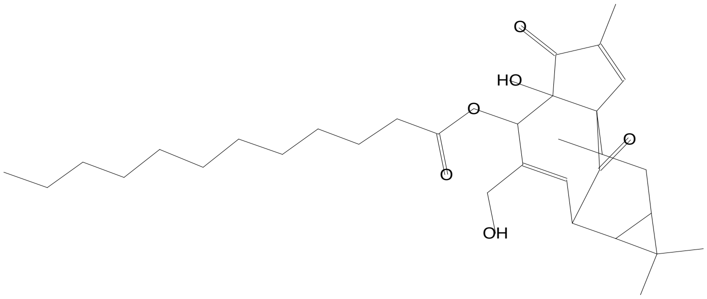

There is no signature data to report.

Library Search Report

RT	Probability	Compound Name	SI	Area %	Area	Molecular Weight	Molecular Formula	Library
89.34	10.00	QUERCETIN 7,3',4'-TRIMETHOXY	426	0.20	628546.27	344	C18H16O7	Wiley9
89.34	7.66	DI-2-BENZOTHIAZOLE DISULFANE	419	0.20	628546.27	332	C14H8N2S4	Wiley9
89.34	4.94	Propane-1,1,2,2-tetracarbonitrile, 3-(4-acetyl-2,5-dimethyl-3-furanoyl)-	406	0.20	628546.27	308	C16H12N4O3	mainlib

Faten-212 #24801 RT: 89.34 AV: 1 RF: 6.00, 3 NL: 4.56E4
F: {0,0} + c EI Full ms [40.00-800.00]

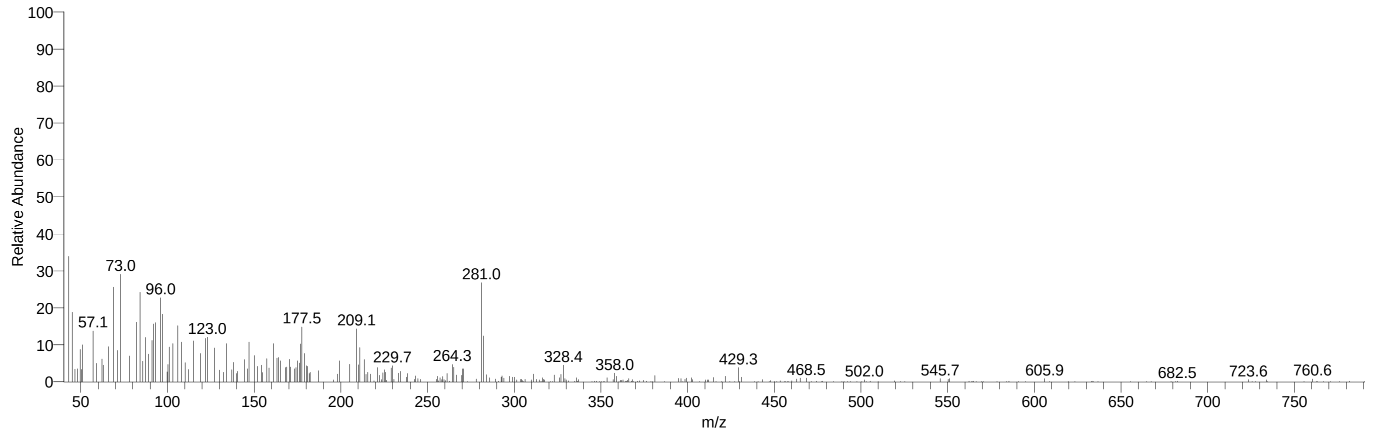

Hit Spectrum | Delta | Compound Structure

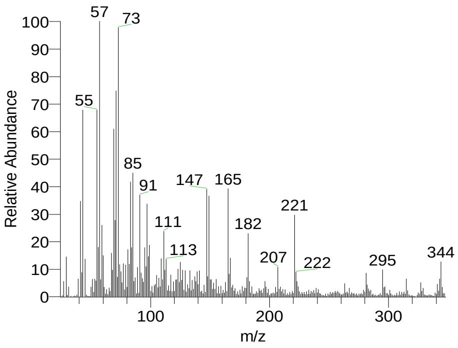

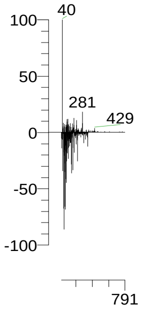

QUERCETIN 7,3',4'-TRIMETHOXY
Formula C18H16O7, MW 344, CAS# 6068-80-0, Entry# 461110
4H-1-Benzopyran-4-one, 2-(3,4-dimethoxyphenyl)-3,5-dihydroxy-7-methoxy- (CAS)

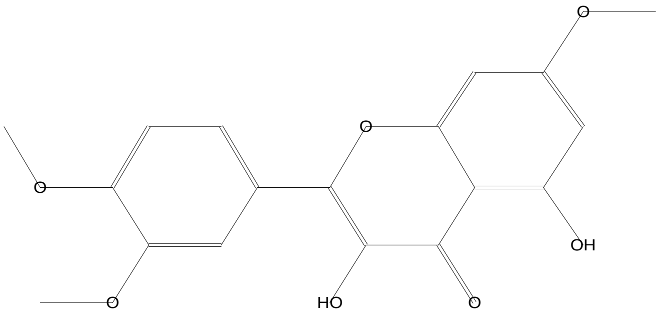

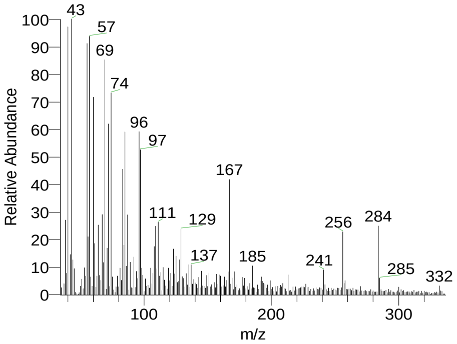

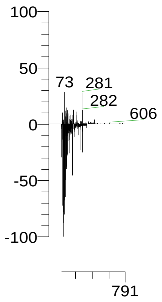

DI-2-BENZOTHIAZOLE DISULFANE
Formula C14H8N2S4, MW 332, CAS# NA, Entry# 438926

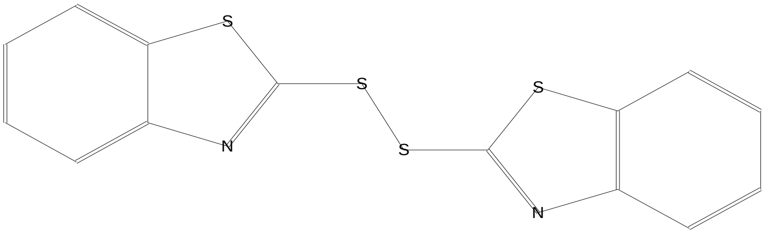

There is no signature data to report.

Library Search Report

Hit Spectrum	Delta	Compound Structure

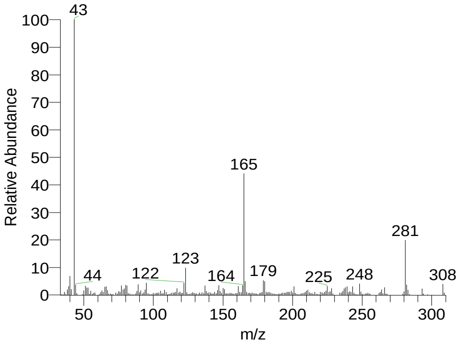

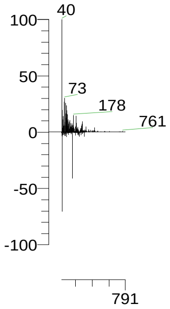

Propane-1,1,2,2-tetracarbonitrile, 3-(4-acetyl-2,5-dimethyl-3-furanoyl)-
Formula C16H12N4O3, MW 308, CAS# NA, Entry# 12141
4-(4-Acetyl-2,5-dimethyl-3-furyl)-4-oxo-1,1,2,2-butanetetracarbonitrile #

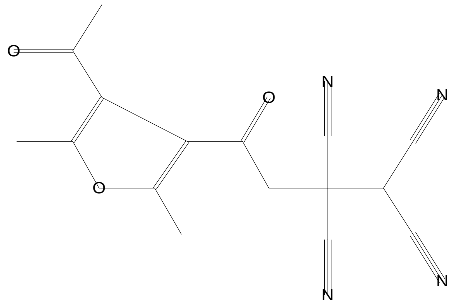

There is no signature data to report.

Library Search Report

RT	Probability	Compound Name	SI	Area %	Area	Molecular Weight	Molecular Formula	Library
89.74	11.82	Gigantine	402	0.18	577882.00	237	C13H19NO3	Wiley9
89.74	10.91	DI-2-BENZOTHIAZOLE DISULFANE	400	0.18	577882.00	332	C14H8N2S4	Wiley9
89.74	7.48	1,4-bis(Chloromethyl)-2,5-dioctyloxybenzene	389	0.18	577882.00	430	C24H40Cl2O2	Wiley9

Faten-212 #24919 RT: 89.74 AV: 1 RF: 6.00, 3 NL: 5.79E4
F: {0,0} + c EI Full ms [40.00-800.00]

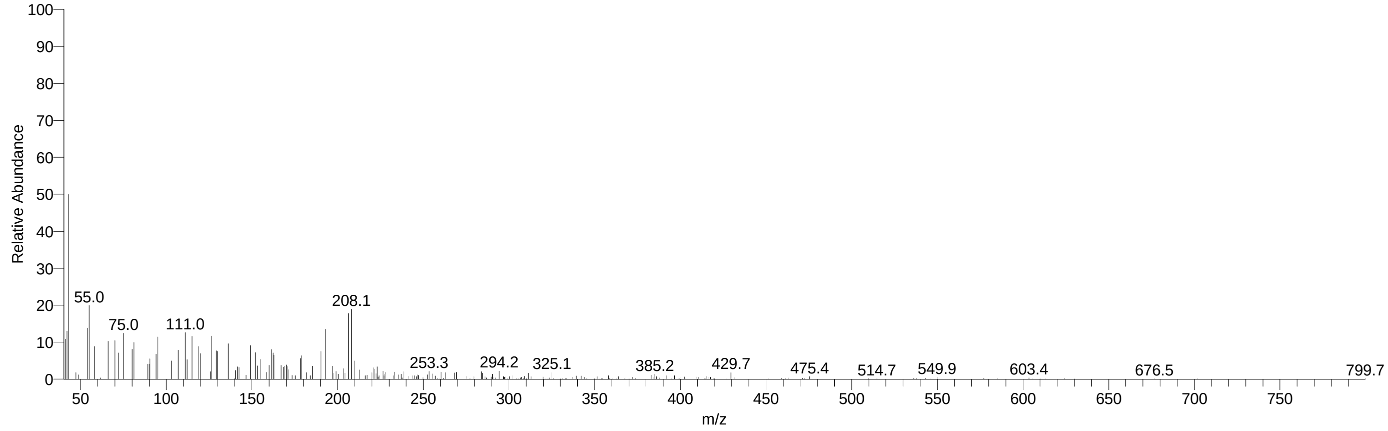

Hit Spectrum | Delta | Compound Structure

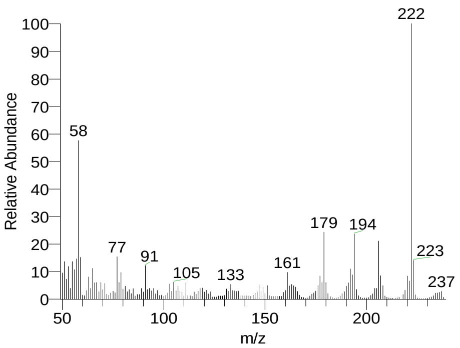

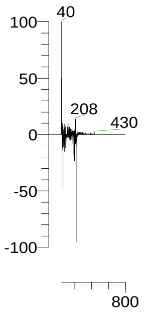

Gigantine
Formula C13H19NO3, MW 237, CAS# 32829-58-6, Entry# 228663
5-Isoquinolinol, 1,2,3,4-tetrahydro-6,7-dimethoxy-1,2-dimethyl-, (s)- (CAS)

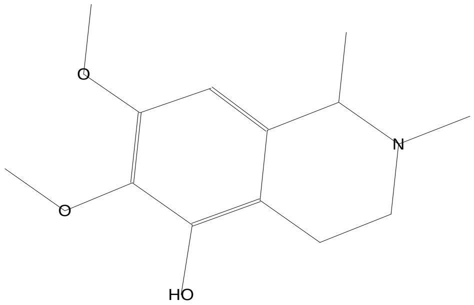

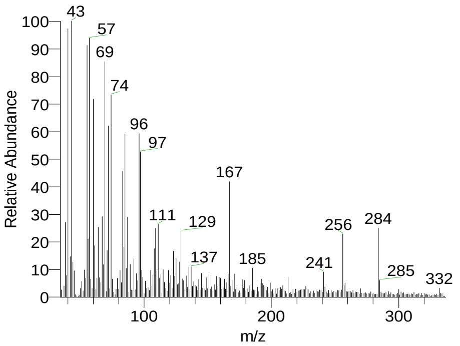

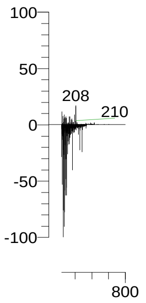

DI-2-BENZOTHIAZOLE DISULFANE
Formula C14H8N2S4, MW 332, CAS# NA, Entry# 438926

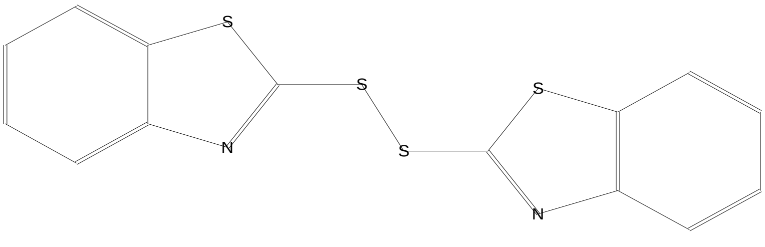

There is no signature data to report.

Library Search Report

Hit Spectrum | Delta | Compound Structure

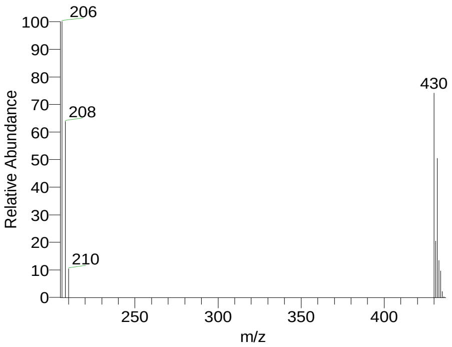

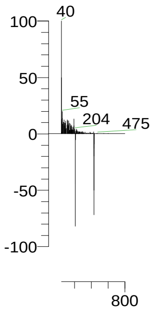

1,4-bis(Chloromethyl)-2,5-dioctyloxybenzene
Formula C24H40Cl2O2, MW 430, CAS# NA, Entry# 573598

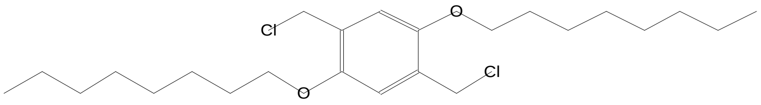

There is no signature data to report.

Library Search Report

RT	Probability	Compound Name	SI	Area %	Area	Molecular Weight	Molecular Formula	Library
89.94	10.32	Sparteine	392	0.15	478353.71	234	C15H26N2	Wiley9
89.94	7.90	N,N'-Pentamethylenebis [s-3-aminopropyl thiosulfuric acid]	385	0.15	478353.71	410	C11H26N2O6S4	mainlib
89.94	6.68	Pregan-20-one, 2-hydroxy-5,6-epoxy-15-methyl-	381	0.15	478353.71	346	C22H34O3	Wiley9

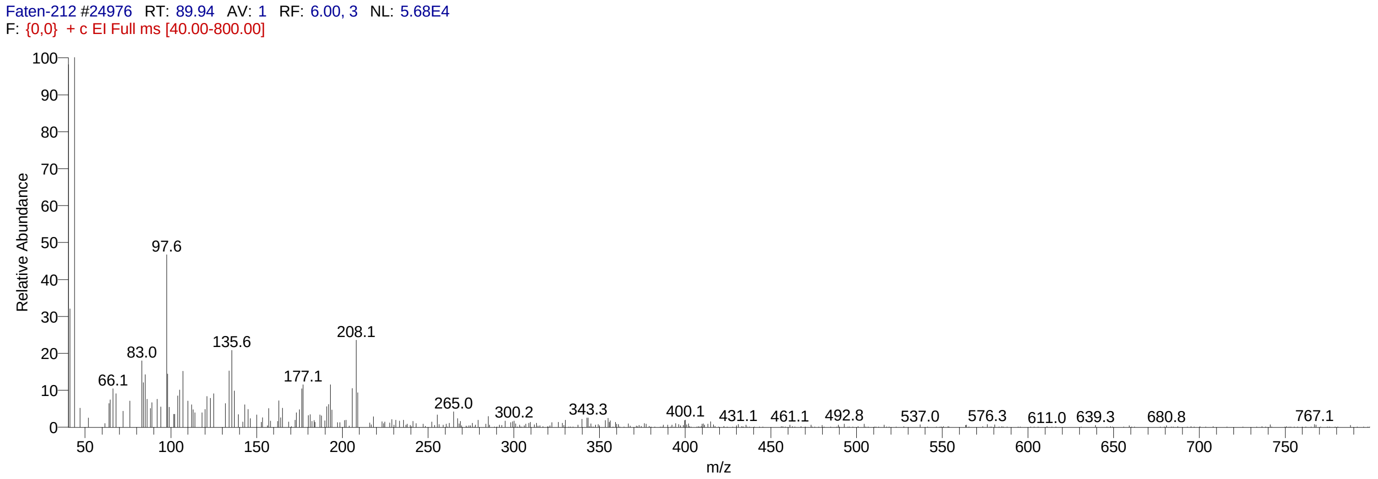

Hit Spectrum | Delta | Compound Structure

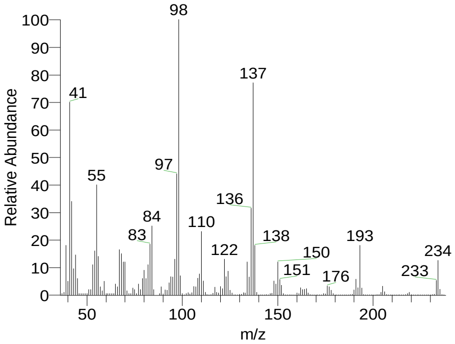

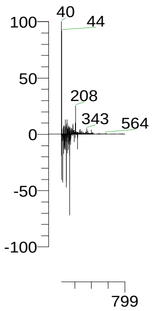

Sparteine
Formula C15H26N2, MW 234, CAS# 90-39-1, Entry# 222243

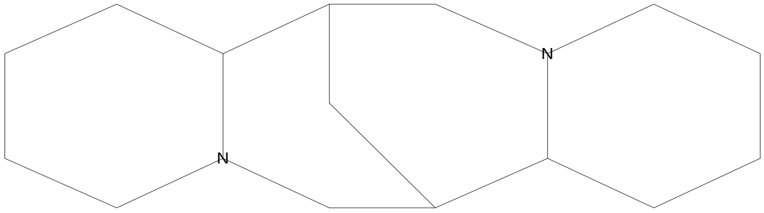

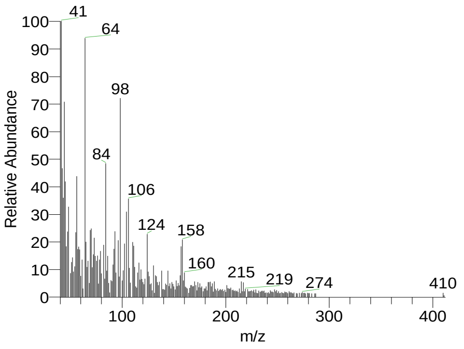

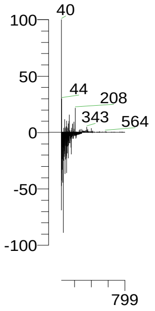

N,N'-Pentamethylenebis[s-3-aminopropyl thiosulfuric acid]
Formula C11H26N2O6S4, MW 410, CAS# 35871-54-6, Entry# 2954

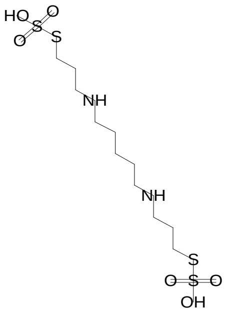

There is no signature data to report.

Library Search Report

Hit Spectrum	Delta	Compound Structure

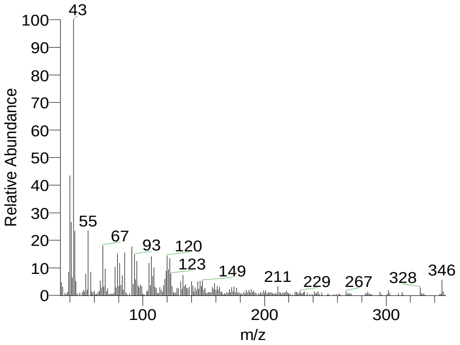

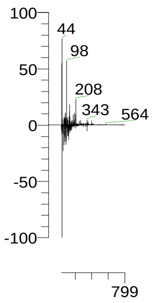

Pregan-20-one, 2-hydroxy-5,6-epoxy-15-methyl-
Formula C22H34O3, MW 346, CAS# NA, Entry# 465849
1-(2-Hydroxy-7,9a,11b-trimethylhexadecahydrocyclopenta[1,2]phenanthro[8a,9-b]oxiren-9-yl)ethanone

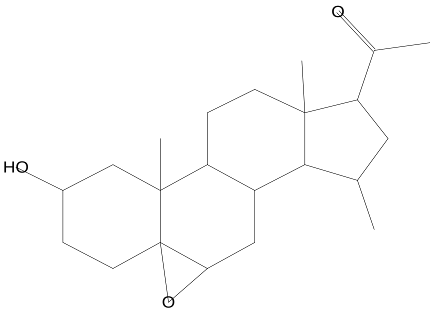

There is no signature data to report.
